# Supplementary material for: Automated literature research and review-generation method based on large language models
Source: Natl Sci Rev. 2025 Apr 25;12(6):nwaf169. doi: 10.1093/nsr/nwaf169 (PMC12125968; doi:10.1093/nsr/nwaf169)
Supplement: nwaf169_Supplemental_Files [file nwaf169_supplemental_files.zip › Gong_ARG_NSR_Appendix1_20250403.pdf]

Automated Literature Research and Review Generation Method based on Large Language Models

Shican Wu[1#], Xiao Ma[1#], Dehui Luo[1], Lulu Li[1], Xiangcheng Shi[2], Xin Chang[2], Xiaoyun Lin[1], Ran Luo[2], Chunlei Pei[1,3], Changyin Du[4], Zhi-Jian Zhao[1,5]* and Jinlong Gong[1,2,3,5,6,7,8]*

[1]*School of Chemical Engineering and Technology; Key Laboratory for Green Chemical Technology of Ministry of Education, Tianjin University; Collaborative Innovation Center of Chemical Science and Engineering (Tianjin); Tianjin 300072, China.*

[2]*Joint School of National University of Singapore and Tianjin University, International Campus of Tianjin University, Binhai New City, Fuzhou 350207, Fujian, China.*

[3]*Zhejiang Institute of Tianjin University Ningbo, Zhejiang 315201, China*

[4]*AIStrucX Technologies, No. 26, Information Road, Haidian District, Beijing 100000, China.*

[5]*International Joint Laboratory of Low-carbon Chemical Engineering, Tianjin 300192, China*

[6]*Haihe Laboratory of Sustainable Chemical Transformations, Tianjin 300192, China.*

[7]*National Industry-Education Platform of Energy Storage, Tianjin University, 135 Yaguan Road, Tianjin 300350, China.*

[8]*Tianjin Normal University, Tianjin 300387, China.*

*Corresponding author. Email: zjzhao@tju.edu.cn; jlgong@tju.edu.cn

[#] These authors contributed equally: Shican Wu, Xiao Ma.

Appendix

Appendix I：Example of Generated Content 3

Propane Dehydrogenation Catalysis: Advancements, Opportunities and Outlook 4

Advances in CO_2 Electroreduction: From Catalyst Design Principles to Reaction Mechanisms - A Comprehensive Review 36

Advances in Spin Control for Catalysis: From Fundamental Principles to Industrial Applications 68

Solid-Liquid Interfaces in Aqueous-Phase Heterogeneous Catalysis: From Molecular Understanding to Sustainable Applications 122

Catalytic Advances in Propane Dehydrogenation: Mechanistic Insights and Material Design Strategies (Generated by DeepSeek-R1) 200

Recent Advances in Propane Dehydrogenation Catalysis: From Molecular Understanding to Industrial Application (Generated by DeepSeek R1+V3) 247

Appendix II：Comprehensive Charts Generated by the Data Mining Module 287

Appendix III：Software Interface 320

Appendix IV：Manual Hallucination Verification Results 325

Appendix V：Review Quality Assessment Scoring Criteria for LLM-Generated Scientific Reviews 326

Appendix VI：Reliability Assessment of Qwen2-7b-Instruct 327

Appendix VII：Comprehensive Model Evaluation Results 330

Appendix VIII：Prompt Templates 351

Appendix I：Example of Generated Content

The review generated on the topic of propane dehydrogenation catalysts, as presented in this section, is a product of automated synthesis. This review comprehensively summarizes and discusses the latest advancements in the research of propane dehydrogenation catalysts. Spanning 35 chapters, it covers various aspects from catalyst design, reaction mechanisms, to reactor design and process considerations. Key topics include metal oxide catalysts such as chromium and gallium oxides, supported metal catalysts like platinum, palladium, and rhodium, bimetallic and alloy catalysts including PtSn and PtZn, molecular sieves and mesoporous supports like MFI and BEA type zeolites, along with catalyst synthesis and characterization, reactor design, and process integration. The review also addresses the primary challenges faced in the study of propane dehydrogenation catalysts, such as the development of non-precious metal catalysts, issues related to catalyst deactivation, and the enhancement of energy efficiency. Recommendations are provided for accelerating catalyst screening and understanding through high-throughput computation and novel in situ characterization techniques.

This automatically generated review offers a comprehensive progress report and an interdisciplinary information synthesis for researchers and industry professionals in the field of propane dehydrogenation catalysts. It aims to assist researchers in further innovation and optimization of relevant catalysts and processes. Below is the content of the review generated by the LLM, unaltered in format for authenticity.

Propane Dehydrogenation Catalysis: Advancements, Opportunities and Outlook

Abstract

Propane dehydrogenation (PDH) has emerged as a critical route for on-purpose production of propylene, an essential chemical feedstock. This review comprehensively surveys recent progress in PDH catalyst design, mechanistic principles, characterization techniques and process intensification. Key advancements include stabilized single-atom Pt sites in zeolites and revelation of active chromium species. However, transforming laboratory achievements like isolated metal sites and bimetallic alloys into commercially viable catalysts requires addressing remaining challenges around deactivation, substituting precious metals and improving energy efficiency. Cross-disciplinary efforts at the intersection of materials discovery, spectroscopic elucidation, computational modeling and microreactor prototyping hold prospects for tackling these gaps. Ultimately, by integrating catalytic innovation with reactor and process systems optimization, next-generation PDH technologies promise enhanced productivity and sustainability.

Introduction - The Significance of Propylene in Chemical Industry and the Importance of Propane Dehydrogenation

Propylene constitutes a vital chemical feedstock utilized in the production of diverse products spanning polypropylene, acrylonitrile, propylene oxide and acetone[1,2], with rapidly rising global demand estimated to reach 191 megatons by 2030 [3]. Conventional production routes via steam cracking and fluid catalytic cracking have proven inadequate to satisfy burgeoning propylene requirements[1,2]. Consequently, on-purpose generation of propylene through propane dehydrogenation (PDH) has emerged as an innovative and economically competitive pathway by exploiting abundant supplies of propane obtainable from shale gas[4,5].

Numerous studies have highlighted PDH's efficacy in redressing propylene's supply deficit through an eco-friendly avenue[6,7]. Exploring novel catalyst and process designs to elevate propylene yield and selectivity from propane seems particularly rewarding[8,9]. Researchers might ex amine integrating PDH with other chemical manufacturing routes in bio- or electro-refinery configurations for optimal resource usage. Overall, PDH retains formidable potential for upscaling by harnessing recent advancements to enable economical and sustainable large-scale propylene production.

Introduction - Mechanisms of Catalysis in Propane Dehydrogenation: The Role of Activation Barrier Reduction

An exhaustive survey of the cited literature[2,5,8,10-15] determined catalysts facilitate

propane dehydrogenation by furnishing reactive conduits that selectively cleave C-H over C-C bonds with mitigated activation barriers. Platinum-anchored catalysts are extensively utilized, harnessing platinum's predisposition to sever C-H moieties while minimizing cracking side-reactions[11,12]. Dominant mechanisms proposed entail hydrogen abstraction to constitute metal-carbon ligatures on coordinatively vacant territory[8,13].

Complementary catalytic formulations under exploration encompass metal oxides such as ZrO2 and Ga2O3 which could furnish oxygen vacancies or Lewis acid constituent sites[14,15]. Analogously, zeolites interchanged with metal cations may contribute electron density to adsorbed propane[3]. Mesoporous silica backbones additionally proffer possibilities for tuning selectivity and reactivity[4].

Introduction - Overview of Key Catalyst Classes in Propane Dehydrogenation

The manuscript highlights several prevailing catalyst categories scrutinized for propane dehydrogenation, encompassing bolstered metals, metal oxides, alloys, and zeolites. Explicitly, supported platinum and chromium oxide catalysts constitute conventional industrial selections employed in the Catofin and Oleflex processes[7]. Supplementary inexpensive alternatives like supported vanadium and iron oxides, gallium oxides, alongside zeolites such as cobalt-laden H-Beta and BEA have exhibited favorable potential[4,6,16]. Despite the dominance of supported metals and metal oxides, intermetallic alloys and isolated metal entity catalysts have recently materialized as active and selective candidates[10,17].

With numerous propitious catalyst genres and formulations presented, sustained efforts to refine extant synthesis protocols alongside exploring unprecedented architectures seems worthwhile. Emergent preparatory techniques could engender superior jurisdiction over active site segregation and metal-support crosstalk to boost stability and selectivity. Incorporating promoters or strategic doping might potentially fine-tune catalytic comportment. More extensive assimilation of operando methodologies can proffer invaluable perspectives into functional state speciation and deactivation pathways to spearhead optimization endeavors.

Theoretical Principles and Computational Approaches - Thermodynamics and Kinetics in Propane Dehydrogenation

An exhaustive assessment of the cited literature[1,5,9,11,18] divulged propane dehydrogenation contends with substantial thermodynamic impediments and kinetic factors curtailing conversion and selectivity.

PDH constitutes an extremely endothermic process[11,18]; with ΔH spanning 124-200 kJ/mol[9,18]. Attaining reaction feasibility necessitates high process temperatures between 500-

650°C[1,5], often engendering coke accrual and other side reactions [10,11]. Additionally, low partial pressures promote product formation by shifting the equilibrium [3]. Introducing oxidants like O2 or CO2 could moderate requisite temperatures through providing exothermic complementary reactions[3], albeit with associated tradeoffs.

Kinetically, PDH exhibits lofty energy barriers around 0.7-1.8 eV for critical C-H activation transitions[4,5], hindering turnover frequences. Support and promoter affiliations assist active site stabilization against sintering [6] at elevated temperatures. Structure-sensitive side reactions like coking and hydrogenolysis manifest on specific motifs, while the principal PDH route may be structure-insensitive[11].

Theoretical Principles and Computational Approaches - Utilizing DFT Calculations for Reaction Mechanism Exploration

Computational examinations harnessing density functional theory (DFT) enumeration have proffered invaluable insights into propane dehydrogenation mechanisms, kinetics and pathways over diverse catalyst systems. [6] DFT interpretations consolidated with microkinetic renderings have elucidated structure-activity interrelationships, pinpointing illustrative descriptors like d-band centers correlating with selectivity on metals [6], alongside unveiling kinetically-limiting strides like propylene C-H activation. [10] DFT has additionally investigated isolated metal entity catalysts, evincing dopant and support ramifications for fine-tuning performance.[17]

Regarding solid acid catalysts, DFT rationalized Zn-O-Zn constituents wield lower apparent activation barriers relative to Zn^2+^ entities for H-ZSM-5 mediated dehydrogenation. [6] For Sn-BEA, DFT deemed the undeviating pathway more kinetically favorable over closed sites versus the indirect sequence, with unrestricted locations demonstrating higher reactivity pending hydroxylation. [16] DFT has also furnished mechanistic intricacies over zeolites, demonstrating dissociative propane adsorption prevails over radical propagation on Zn-loaded mordenite.[19]

Collectively, DFT contributes foundational atomic-scale perspectives interlinking configuration, descriptors and mechanisms to observed catalyst performance inclinations. Persisting refinements in functionals, assimilating entropy and solvent influences, alongside coalescing with microkinetic renderings will enable enhanced computational illumination and screening for optimal PDH catalysts.

Theoretical Principles and Computational Approaches - Insights from Microkinetic Models in Dehydrogenation Kinetics

An exhaustive assessment of the cited literature[5,8,20] determined microkinetic models have furnished perspectives into propane dehydrogenation kinetics:

The manuscript[8] presents microkinetic formulations of PDH harnessing DFT-acquired variables. It examines reaction rate equations, surface coverage extents, turnover frequencies, selectivity coefficients and rate-limiting strides.

The publication [5] unveils microkinetic simulation endeavors exercising the CATKINAS suite to evaluate single-atom catalyst kinetics and prospective industrial applicability. Parameters obtained from DFT enumerations were recruited to conduct the simulations.

The article[20] exhibits microkinetic renderings that exercised descriptors for screening and stratifying intermetallic catalysts founded on formation energies and reaction rate representations. This facilitated predicting activity and propylene selectivity.

Theoretical Principles and Computational Approaches - Correlating Catalyst Properties with Performance: The Role of Descriptors

As evidenced in the manuscripts[5,15,20], select examinations have harnessed computational methodologies to unveil structure-activity affiliations and pinpoint descriptors correlating catalyst traits to propane dehydrogenation performance.

One undertaking instituted scaling relationships between formation energies of PDH transient species on transition metal planes to ascertain the ethyl and methylidyne adsorption energies as ideal reactivity descriptors[20]. Another endeavor identified a volcano-type activity trend with respect to propylene adsorption energy by means of microkinetic simulation of single-atom catalysts[5].Additionally, degree of rate control interrogation on DFT-enumerated transition states was leveraged to ascertain rate-limiting PDH strides and accordingly construct descriptors derived from the energies of said strides[15].

Complementing computational quests, select empirical studies have also delineated structure-activity correlations. The dispersion of active constituents, metal loading extents and support type were allied with catalytic performance[4]. Platinum atom ensembles were revealed to drive coke accrual, while segregated single platinum atoms conferred enhanced resilience[11].

Metal Oxide Catalysts - Efficacy of Metal Oxides like Cr2O3, Ga2O3 in Propane Dehydrogenation

Among investigated metal oxide catalysts, polymorphs of gallium oxide and chromium oxide demonstrate particular promise for propane dehydrogenation based on reported productivity.

Gallium oxide polymorphs including α-Ga2O3, β-Ga2O3, and γ-Ga2O3 were assessed by Zhang et al., with β-Ga2O3 exhibiting maximal initial activity attributed to superior surface acidity from NH3-TPD characterization[21]. Silica-supported β-Ga2O3 and γ-Ga2O3 also gave excellent propylene yields. Cargnello et al. verified strong intrinsic activity for propane activation over amorphous Ga2O3 nanoparticles on silica using in-situ IR spectroscopy[14]. In comparative

screening across metal oxides, Ga_2O_3 outperformed Fe_2O_3 and Cr_2O_3, achieving 60% propane conversion[4].

Chromium oxide has also garnered interest, with Cr-based catalysts recognized among the most active formulations for oxidative dehydrogenation of propane with CO_2[22]. Using kinetic Monte Carlo simulations, Chang et al. proposed a detailed non-oxidative PDH mechanism over the Cr_2O_3(0001) model surface[23]. Weak metal-support interactions with SiO_2 were shown to limit over-oxidation, improving Cr_2O_3's propylene selectivity[4]. Against different metal oxides, Cr_2O_3 gave higher yields than Fe_2O_3 and Al_2O_3[4], although deactivation from coking remains an issue.

In summary, gallium and chromium oxides have exhibited encouraging intrinsic PDH activity and propylene selectivity as cheaper alternatives to conventional metals. However, stability and longevity still need improvement through composition modulation, metal-support interfacial engineering, and other strategies. Advancing these metal oxide catalysts could ultimately provide economical and scalable PDH process technologies. Ongoing efforts should emphasize clarifying active site speciation, delineating degradation mechanisms, and translating insights into superior catalytic architectures.

Metal Oxide Catalysts - Influence of Oxidation State and Oxygen Mobility on Catalyst Performance

The manuscript divulges substantiation exhibiting tuning oxidation states, oxygen mobility, and acid-base attributes of metal oxides can influence propane dehydrogenation performance [24].

Regarding oxidation states, eliciting oxygen vacancies on Al_2O_3 reduced aluminum coordination, begetting Al_{cus}-O acid-base couples fine-tuning transient species adsorption energies alongside lowering reaction barriers, thereby boosting PDH activity[24]. Partly reduced V^{3+}/V^{4+} vanadium oxides on supports like ZrO_2 were also identified as the reactive conduits for PDH without supplemental oxygen introduction [25]. For chromium oxides, oscillating between Cr^{3+} and Cr^{6+} oxidation states assists completing the Mars-van Krevelen redox mechanism integral for activity[26].

In terms of oxygen mobility, $LaZrO_x$ with the most labile metal-oxygen linkage and highest oxygen motility demonstrated the greatest intrinsic PDH activity among SiO_2, Al_2O_3 and ZrO_2 adjuvants [27]. TiO_x ultrathin nanosheets with abundant oxygen vacancies also exhibited higher concentrations of coordinatively unsaturated Ti_{4c} sites after H_2 reduction, directly correlating with improved PDH performance [28].

Regarding acid-base traits, tailoring the Zr/Ga ratio of ZrO_2/Ga_2O_3 mixed oxides produced optimal base site density and acid site strength, with a Zr/Ga ratio of 0.26/1 attaining 50.1% propane conversion [14]. The surface acid site density of Ga_2O_3 polymorphs also correlated with initial catalytic activity, with β-Ga_2O_3 displaying the maximum density [29]. Manipulating these

oxidation states, oxygen mobility extents, and acid-base properties thus presents clear prospects to enhance metal oxide catalysts for propylene generation.

Metal Oxide Catalysts - Mechanistic Scenarios in Different Metal Oxide Catalysts

An exhaustive literature review[4,30-37] divulged assorted mechanistic postulations over metal oxide catalysts:

Several manuscripts delineate Mars-van Krevelen archetypes operational during propane dehydrogenation over metal oxides like VOx/ZrO2[30], CrOx/ZrO2[31] and MoVO mixed oxides[32]. Herein, lattice oxygen initiates reactions through abstracting hydrogen from propane. Ensuing alkyl intermediates eventually yield propene, while the partially reduced metal oxide surface becomes re-oxidized by O2 or CO2 thus completing the catalytic sequence.

Segregated metal cations with coordinative unsaturation (designated Mcus) manifest as reactive loci on numerous catalysts. On CrOx/ZrO2, Zrcus positions adjoining oxygen vacancies catalyze C-H cleavage [33]. Isolated Zn2+ on ZrO2 [34] and Ga+/H+ pairs on Ga/H-ZSM-5[35] also demonstrate appreciable activity. Proximity of these Mcus sites to acid/base functionalities assists subsequent reaction strides like hydride transfer.

Several manuscripts accentuate the role of redox cycles Mx+ ↔ M(x-1)+ (x = 3-6) in activating propane on Cr [4] and V oxides [36]. Optimal valency states like Cr3+ or V3+ counterpoise activity and resilience to over-reduction. Tetrahedral Ga3+ motifs selectively cleave C-H bonds on Ga2O3 via heterolytic scission sans altering oxidation state [37].

Metal Oxide Catalysts - Impact of Metal-Support Interaction on Activity and Selectivity

Wang et al.[25] explored metal-support interfacial effects in VOx catalysts for propane dehydrogenation, systematically scrutinizing influence of assorted oxide adjuvants on supported VOx activity. VOx/ZrO2 demonstrated over 4-fold superior activity relative to VOx bolstered on other oxides, ascribed via DFT renderings to weakened VOx-ZrO2 affiliations attenuating electron density on bridge oxygen atoms and thus elevating C-H cleavage capability in propane. The principles established offer guidance for tuning VO active site microenvironments to achieve improved PDH performance.

Additionally, Liu et al. [38] exhibited that for vanadium oxide on silica adjuvants, fragile interaction with surface silanol constituents elicits undesired 3D nanoparticle accretion. Augmenting sodium content engenders more reactive SiONa+ loci instigating 2D vanadia anchoring and thus optimizing dispersion to match other supports, consequently ameliorating activity while preserving selectivity.

Moreover, Zhang et al. [39] demonstrated dealumination of BEA zeolite substratum produces

silanol functionalities avowing isolated Co2+ cations. These resilient cobalt active sites preferentially activate C-H over C-C bonds in propane, attaining elevated propylene selectivity.

These instances underscore strategic manipulation of metal-support bimodules in oxide catalysts furnishes targeted avenues for tailoring PDH performance.

Supported Metal Catalysts - Exploration of Single Metals like Pt, Pd, Ir in Propane Dehydrogenation

An exhaustive literature survey [40-54] divulged several explorations of supported mono-metallic Pt, Pd or Ir catalysts for propane dehydrogenation.

An undertaking by Burch and Watling[41] scrutinized incorporating sulfur during preparatory phases or reaction streams over Pt/alumina catalysts for PDH. Cardinal revelations were H2S pre-dosing at 873 K substantially upgraded selectivity towards propene, alongside sulfur assimilation during synthesis demonstrating higher efficacy than during operation.

Additional works evaluated Pt on boron nitride (Pt/BN) [42], SBA-15 (Pt/SBA-15)[43], Al2O3 [44,45], and zeolites such as Ge-UTL (Pt/Ge-UTL) [46] and Zn-ZSM-5 (Pt/Zn-ZSM-5)[47]. Promoter influences like Ca[48], Sn [40,49] and Pb[50] on Pt catalysts were also probed. These contributions offered wisdom into metal-support affiliations, alloying effects and tactics for upgrading Pt resilience and selectivity.

A few examinations also surveyed supported Pd[51], Rh [52], Ru [53,54] and Ir [51], albeit with more limited datasets relative to Pt catalysts.

Collectively, Pt-anchored systems persist as the most extensively dissected mono-metallic configurations for PDH, with sophisticated mechanistic perspectives garnered on supported Pt catalysts employing advanced characterization techniques. However, broadening explorations to additional noble metals and modifiers could unveil more opportunities for enhancing catalytic performance.

Supported Metal Catalysts - Effect of Metal Particle Size, Shape, and Support on Catalytic Activity and Stability

The cited literature[42-44,46,55,56] proffers substantiation indicating traits like metal particle dimensions, morphology, support interactions and pretreatment conditions affect catalytic performance and resilience of reinforced metal catalysts for propane dehydrogenation.

Explicitly, reference[43] reveals a Pt/SBA-15 catalyst configured via deposition-precipitation possesses much smaller Pt particles relative to wet impregnation protocols. The smaller Pt particles confer superior initial conversion and propylene selectivity, despite deactivation owed to coke accrual. Reference[44] demonstrates depositing uniform, nanoscale Al2O3 overlayers on Pt nanoparticles via ALD augments stability and selectivity by impeding sintering and limiting

formation of less selective sites during steam regeneration. An optimal coating thickness of 6-8 Å after 5-7 ALD cycles is discerned.

Regarding shape effects, reference[55] calculates under-coordinated Pt(211) facets prevail over Pt(111) terrace sites in governing reactivity, despite reduced surface areas. This signifies the considerable morphologic influence on catalytic comportment. Additionally, reference[56] elucidates the reaction sequence and pivotal transient species participating during PDH on Pt(111), thus providing mechanistic perspectives.

Concerning metal-support interactions, reference [42] demonstrates robust affiliations between Pt and boron nitride post high temperature preconditioning. This elicits partial encapsulation of Pt particles by boron oxide overlays, which constrains particle dimensions, occludes non-selective loci and thus improves resilience. Analogously, reference [46] stabilizes ultrafine Pt clusters in 14-membered ring zeolite pores via Pt-O-Ge bonds. This tailored metal-support conjunction confers outstanding PDH performance.

In summation, deliberate modulation of attributes like particle size, morphology, metal-support bimodules and preparatory conditions empowers governance over abundance and accessibility of selective sites, hence enabling optimization of reinforced metal catalysts for targeted PDH conversion and propylene selectivity.

Supported Metal Catalysts - Mechanistic Pathways in Supported Metal Catalysts

A meticulous literature interrogation revealed most contributions concentrate on characterization and assessment of catalytic performance over manifold supported metal catalysts without delving into mechanistic postulations or reaction coordinates.

Scarce exceptions discussing mechanisms encompass reference[44] , which proposes distinct trajectories over Pt(111) and Pt(211) facets – a reverse Horiuti-Polanyi sequence on Pt(111) and an unconventional non-reverse pathway on Pt(211) entailing successive dehydrogenation and hydrogenation strides. Reference[46] also delineates a mechanism over Pt-Ge dual sites on UTL zeolite, with the inaugural C-H scission constituting the rate-limiting step.

In summation, most academic contributions lack profound mechanistic analysis and future endeavors should emphasis more rigorously garnering molecular-scale perspectives into catalytic cycles and active site prerequisites for supported metal catalysts in propane dehydrogenation. Computational examinations assimilated with sophisticated spectroscopic probing can facilitate unveiling mechanistic intricacies and thus bolster rational catalyst optimization.

Supported Metal Catalysts - Observations in Selectivity Changes on Metallic vs Oxidized Surfaces

A meticulous examination of the presented references unveiled a paucity of side-by-side

appraisals contrasting the selectivity renditions of metallic relative to oxidized supported metal catalysts for propane dehydrogenation. Maximum manuscripts concentrated exclusively on gauging manifold bimetallic or alloy catalysts such as PtZn[57], PtGa [58] and PtSn[59] for propane dehydrogenation devoid of oxidized analogs.

A few publications explored metal catalysts reinforced on constituents like H-ZSM-5 [35], alumina[60] and titanosilicate TS-1 [61] sans contrasting oxidized configurations. Certain manuscripts evaluated metal oxide catalysts encompassing chromium [62] and vanadium oxides [63], nonetheless omitting metallic counterparts or selectivity comparisons thereof.

Collectively, appreciable gaps persist regarding oxidation state ramifications on selectivity trends for supported metal propane dehydrogenation catalysts. This arena shows formidable promise for supplemental explorations towards augmenting structure-function comprehension to facilitate the conception of more selective catalytic materials.

Bimetallic and Alloy Catalysts - Propane Dehydrogenation Performance in Pt-Alloyed Catalysts

An exhaustive literature review[6,20,64-71] revealed bimetallic and alloy Pt catalysts assimilated with Sn, Ga and Zn demonstrate ameliorated propane dehydrogenation performance regarding activity, selectivity and resilience versus monometallic Pt configurations. Incorporating Sn assists suppressing hydrogenolysis and coke accumulation, thereby boosting selectivity[64,65]. Pt-Ga constitutes highly resilient intermetallic amalgams, resisting sintering and coking over prolonged reaction durations [68]. Alloying Pt with Zn enables tailoring ensemble dimensions and electronic structure to attain optimal sites for C-H activation whilst circumventing overbinding C3 entities [69].

Pivotal structural traits influencing propane dehydrogenation performance have been pinpointed. Subnanometric PtSn clusters in MFI zeolite conduits deliver 10-fold superior activity relative to PtSn nanoparticles, attributed to optimized Pt-Sn affiliations[64]. Segregated Pt atoms stabilized by Zn complexes in the framework of dealuminated Beta zeolite achieve remarkable resilience and near-equilibrium conversion [69]. PtZn intermetallic nanoparticles in mesostructured MFI nanosheets overcome diffusion limitations to attain elevated productivity[67].

The suitable Sn/Pt ratio has also been delineated for optimal dehydrogenation activity and propylene selectivity. For PtSn clusters on alumina, Pt/Sn ~ 3 conferred moderate turnover frequencies; comparable Pt/Sn ratios were deemed inert[66]. Elevating Sn/Pt from 0 to 1.5 boosted propane conversion over PtSn/Al2O3 preceding detriments from excess Sn[72]. These trends affirm computationally predicted volcano-type dependence of rate on Sn content [73].

Integrative understanding and prospective outlook: The accumulated discernments on bimetallic Pt catalysts accentuate promising architectural paradigms that could stimulate further

advancements. Constituting isolated single-sites in zeolites, deliberate selection of secondary metals, compartmentalizing ultrasmall nanoclusters and interface engineering with unconventional adjuvants each tender adaptable modules to persist optimizing propane dehydrogenation arrangements. Additionally, operando spectroscopies[71] and multi-scale renderings may facilitate elemental comprehension to inform rational catalyst formulation targeting industrially-relevant process intensification. More expansive deployment of high-throughput experimentation and data-driven tactics can also expedite identifying unprecedented compositions and metastable phases with bespoke functionalities.

Bimetallic and Alloy Catalysts - Electronic and Geometric Effects of Secondary Metals

Wang et al.[64] exhibited larger Pt nanoparticles can incorporate more Sn entities to constitute PtSn alloy nanoparticles, while smaller Pt clusters tend towards Pt-rich compositions with limited Sn assimilation. These insights could be extended to additional Pt-anchored bimetallic catalysts, although the secondary metal election (e.g. Cu, In, Zn or Ga instead of Sn) may affect optimal Pt site dimensions [64].

The publication by Zope et al. [66] probes electronic and geometric influences of Sn as a secondary metal in bolstered PtSn bimetallic clusters. It demonstrates Sn constructs robust bonds with Al2O3 support oxygens at the cluster-support interface. Sn also manifests a stabilization effect on the Pt clusters[66].

The manuscript by Meng et al. [54] scrutinizes both electronic and geometric impacts of phosphorus addition in Ru-P bimetallic catalysts. Escalating P content is exhibited to diminish Ru ensemble magnitude, decreasing the hydrogenolysis rate (geometric effect). Ru-P bond archetype formation also shifts Ru XPS and XANES spectra to elevated energies, alluding to reduced Ru 4d orbital energies enfeebling metal-adsorbate affiliations (electronic effect) [54].

The publication by Zhao et al.[74] examines electronic and geometric ramifications of assimilating Ti and Nb as secondary metals in Pt-Ti and Pt-Nb bimetallic catalysts synthesized via reactive metal support interactions with MXene adjuvants. It evinces tailored intermetallic nanoparticles become conceivable that are unachievable with conventional oxide or carbide supports. The distinct MXene compositions enabled tuning of catalytic properties and performance [74].

The manuscript by Xie et al.[75] probes electronic effects of the secondary metal Ga in GaPt bimetallic catalysts harnessing XPS and IR documentation. The data insinuates electron donation from Ga culminating in elevated electron density on Pt sites owed to GaPt amalgam formation, thereby enhancing catalytic performance[75].

Bimetallic and Alloy Catalysts - Optimization of Selectivity through Subsurface Tuning

The presented references revealed none of the scrutinized manuscripts explored assimilating subsurface promoters like Re or Mn in bimetallic and alloy catalysts for tuning propane dehydrogenation selectivity. No tangible instances or documentation were furnished across the manifold surveyed studies[12,16,18,34,54,64-67,69,70,74-89].

Nonetheless, one publication[90] dissected harnessing subsurface Mn promotion in Pt3Mn bimetallic catalysts for propane dehydrogenation. The subsurface Mn constituent markedly upgraded selectivity over Pt catalysts alone by regulating surface chemistry and adsorption attributes. Kinetic archetypes were also formulated to explicate the distinct subsurface laminations in modifying selectivity. Ultimately, this highlights the promise of tuning subsurface promoters and compositions to optimize catalytic performance.

Bimetallic and Alloy Catalysts - Synergistic Effects in Metal Alloys: Ensemble and Ligand Effects

The presented literature[54,57,81,83,91,92] revealed select examples showcasing synergistic effects between metals in bimetallic propane dehydrogenation catalysts, credited to ensemble and ligand impacts:

Explicitly, Ru-P catalysts exhibited reduced Ru ensemble magnitudes and isolated Ru atoms from escalating P assimilation, which attenuated the hydrogenolysis rate [54]. PtIn2 nanoparticles displayed the maximum perturbation in Pt XANES energy and geometric transitions, culminating in superior activity and resilience[81]. Subnanometric Pt-Sn clusters in zeolites possessed distinct geometric and electronic configurations relative to Pt-Sn nanoparticles, eliciting unique reactivity[83].

Broadly, adjoining a secondary metal can physically disrupt substantial ensembles on Pt accountable for side-reactions while modifying the electronic ambience to promote preferred product desorption [91,92]. For instance, alloying Pt with Zn sequesters Pt sites and reshapes selectivity [92], while Sn addition regulates Pt electron density [91].

Zeolites and Mesoporous Supports - Promising Zeolite Topologies in Catalyst Support

A meticulous literature analysis[9,57,61,64,69,79,91,93,94] revealed several zeolite architectures demonstrating propitious credentials as propane dehydrogenation catalyst supports encompassing MFI, BEA and FER topologies:

MFI Zeolite

The purely siliceous MFI zeolite designated silicalite-1 constitutes an optimal backbone for anchoring exceptionally dispersed subnanometric PtSn clusters within the sinusoidal 10R conduits, eliciting outstanding PDH credentials with 45.3% propane conversion and 99% propylene

selectivity[91]. Its hierarchical micro-/mesoporous surrogate empowers PtLa intermetallic nanoparticles to exhibit elevated initial activity (~40% conversion) alongside exceptional 30-day resilience with <10% deactivation [93]. Ferrosilicalite-1 (Fe-S-1-n) stabilizes isolated Pt atoms bonded to Si-O-Fe loci, elevating selectivity[79]. Mesoporous MFI nanosheets strongly anchor PtZn alloys, imparting remarkable durability [57].

BEA Zeolite

Tin-augmented zeolite Beta (Sn-Beta) efficiently localizes isolated Pt atoms inside Zn nests within dealuminated BEA pores. The ensuing single sites catalyze PDH with 90% propylene selectivity adjoining equilibrium conversion [69]. Pt stabilized on segregated Sn positions in the Beta framework also exhibits excellent catalytic performance [94]. The BEA topology instills a confinement effect augmenting PtSn particle dispersion [64].

Other Zeolites

Chabazite zeolite (CHA) sequesters extra-framework Ga+ active sites to accomplish 96% propylene selectivity, although with gradual deactivation over time[9]. Titanosilicate TS-1 retains weaker acid locales, refining propane conversion and catalyst longevity [61].

In summation, MFI and BEA zeolites have exhibited particular potential as PDH catalyst supports owing to merits like robust metal confinement, acid site segregation and hierarchical porosity. However, opportunities persist to explore emerging zeolite and mesoporous scaffolds to additionally advance catalytic performance.

Zeolites and Mesoporous Supports - Impact of Si/Al Ratio and Mesoporosity on Catalyst Performance

Select studies evaluated the impacts of tuning silicon/aluminum ratios and augmenting mesoporosity in zeolitic PDH catalyst adjuvants:

The manuscript with DOI [9] gauges propane dehydrogenation over CHA zeolites with varying Si/Al proportions of 5, 12 and 25. It demonstrates extra-framework Ga+ sites manifest irrespective of Si/Al ratio. While reaction kinetics decay over time, propylene selectivity persists at 96% upon aptly configuring the Ga/Al ratio.

Moreover, the research articulated in [61] investigates modulating TS-1 crystal dimensions from 100 nm to 5.7 μm. Inferior dimensions introduce mesoporosity, refining diffusion and averting coking. This substantially augments conversion, selectivity and catalyst longevity.

Additionally, the publication with DOI[57] probes mesoporous MFI zeolites. The mesoporous morphology alongside abundant silanol moieties facilitate intermetallic alloy nanoparticle accretion.

Zeolites and Mesoporous Supports - Techniques for Synthesizing Isolated Single Sites in Zeolites

A meticulous literature interrogation[9,18,46,49,85,95,96] revealed select techniques enabling isolated single metal site synthesis in zeolitic scaffolds for propane dehydrogenation catalysts:

Dealuminating zeolites like beta or ZSM-5 spawns vacant lattice locales and hydroxyl nests capable of localizing isolated metal species. The study [18] discusses dealuminating zeolite beta with nitric acid, thereafter stabilizing single cobalt sites within the formed hydroxyl nests. Analogously, atomically dispersed platinum fastened to silanol moieties in dealuminated BEA zeolite displays activity and resilience for PDH[49].

Direct hydrothermal zeolite fabrication with metal-ligand precursors enables constructing isolated metal sites into the superstructure. [85] exemplifies capturing zinc cations coordinated to ethylenediamine inside micropores of silicalite-1. Additives like Cu boost the accretion of exclusively isolated zinc sites in S-1, validated via EXAFS[95].

High-temperature hydrogen reduction converts entities like zinc methyl and zinc hydroxide in ZSM-5 pores into isolated Zn^2+ fastened at cation loci[96]. Ga-CHA necessitates 700°C reduction to configure tetrahedrally coordinated, single Ga+ sites reactive for alkane activation [9].

Sophisticated microscopy and spectroscopy techniques unambiguously affirm isolated metal site structure. Scanning transmission electron microscopy elucidates the positioning of single platinum atoms tethered to framework germanium in dealuminated UTL zeolite[46]. X-ray absorption fine structure spectra in[95] corroborates Cu supplementation constructs exclusively isolated zinc sites in S-1.

Zeolites and Mesoporous Supports - Enhancing Stability through Metal Encapsulation in Zeolites

The scrutinized publications[6,39,46,97,98] demonstrated zeolites and mesoporous substances harboring promise as scaffolds for localizing metal species to augment propane dehydrogenation catalyst resilience.

Several pivotal instances prove strategic confinement of platinum clusters or encapsulating sub-nanometric bimetallics inside zeolite conduits and chambers forestalls agglomeration and sintering at high PDH temperatures or during regeneration:

- Cobalt species tethered on BEA-type zeolites exhibited no deactivation over 8 reaction-regeneration PDH cycles, enabled by the confinement effect stabilizing metal clusters[39].

- Platinum anchored on germanium loci in UTL-type zeolites maintained exceptional stability for over 4000 hours of PDH, credited to Pt-O-Ge bonding affiliations localizing platinum[46].

- Sub-nanometric PtSn entities regioselectively emplaced inside MFI-type zeolites preserved cycle resilience for at least 3 PDH cycles sans variations in cluster dimensions or deactivation[98].

- Confining platinum inside MCM-22 zeolite micropores hindered agglomeration post extreme oxidation-reduction processing at 650°C. The encapsulated catalyst retained 90% initial PDH

activity after 5 cycles while a conventional impregnated catalyst forfeited over 40% [97].

In summation, strategic zeolite encapsulation ploys manifest as a propitious tactic for upgrading metal dispersion and reinforcement for sustained PDH operation. Fastening guest metals on zeolite sites or containment inside micropores impedes sintering thus maintaining active sites and initial activity over recurrent deployment. Additional developments in zeolite composites and mesoporous superstructures may unlock supplementary prospects for next-generation encapsulation techniques.

Catalyst Synthesis and Characterization - Optimizing Active Phase Dispersion through Preparation Methods

A meticulous literature review[9,18,39,62,73,77,95,99-102] revealed numerous studies developing advanced catalyst preparation tactics aiming to optimize active phase dispersion for superior propane dehydrogenation credentials: Methods like vacuum impregnation[39] and assimilating metals during zeolite hydrothermal fabrication[102] can refine dispersion by configuring interactions with the support. Dealuminated zeolites and ordered mesoporous substances furnish open frameworks and high surface areas to disperse and reinforce isolated sites [62,101]. Surface organometallic chemistry enables precisely engrafting metals onto supports via robust metal-oxygen-support bonds [18]. Other ploys encompass colloidal [73] and dendrimer-encapsulated nanoparticle synthesis[100], atomic layer deposition [99] and stabilization in microporous zeolites[77].

In situ spectroscopy approaches like XAFS, XPS, IR and DRIFTS have elucidated alterations in metal speciation during preparation like impregnation [9,95], thus enabling parameter optimization impacting dispersion. Characterization avenues like HAADF-STEM imaging affirm isolation and morphology of active sites.

Integrative understanding and prospective outlook: The scrutinized works evidence appreciable progress in synthesizing propane dehydrogenation catalysts with uniform and thermally resilient active phases, encompassing isolated metal site stabilization, bimetallic amalgam crafting and ultrafine cluster containment – enabled by emergent characterization tools imparting atomic-scale precision.

Future horizons may assimilate these upgrades with other burgeoning techniques like additive manufacturing to architect bespoke dehydrogenation catalysts for process intensification and modularization. Cross-linking developments in operando mechanistic elucidation with high-throughput computational screening could additionally expedite optimized catalyst revelation. More expansive adoption of earth-abundant substitutions may amplify economic viability. Ultimately, transformative propane dehydrogenation systems seamlessly fused with renewable power and

carbon capture provide fertile ground for multifaceted innovation.

Catalyst Synthesis and Characterization - Insights from In-Situ Techniques in Active Catalysts

A meticulous literature analysis[95,100,103-107] revealed in-situ techniques furnishing perspectives into functional propane dehydrogenation catalysts:

One publication harnesses in-situ DRIFTS and temperature programmed desorption with MS detection to probe propylene adsorption modalities on cobalt catalysts, unveiling silicon modification influences on propylene interaction[103]. Another alliance of quantitative in-situ FTIR and pulse titration experiments determines identity and density of reactive gallium species in Ga/H-ZSM-5 catalysts during propane dehydrogenation [104]. Synchrotron-based in-situ XAS at the Zn K-edge distinguishes binuclear ZnOx species on silicalite-1 from 3D ZnOx clusters materializing in-situ on dealuminated Beta during reaction[105]. In-situ Raman spectroscopy and online MS enables identification of a VOx-C3H5 transient species and ascertaining rate-limiting H2 accrual over VOx/HMS catalysts[106].

Other instances harness combined in-situ UV-Vis/Raman spectroscopy with XANES and EXAFS to correlate chromium oxidation state variations with propane dehydrogenation activity and deactivation kinetics[100]. In-situ FTIR exposes transformation of inactive zinc hydroxide motifs into reactive zinc hydride sites during propane dehydrogenation induction periods over ZnO/SBA-15[95]. And an examination alloying GC analytics with in-situ XRD and DRIFTS elucidates fluctuations in product distribution, vanadium oxide phase transitions, and surface transient species during time-on-stream experiments [107].

The multiplicity of in-situ methodologies provides granular insights into functional catalysts encompassing bulk to surface and even pinpointing precise active sites in select instances. Developments in characterization capabilities now enable tracking catalyst structural dynamics from the molecular tier upwards. While most studies have accentuated simpler lab systems, assimilating these operando avenues for online monitoring in industrial reactors could facilitate superior process control and mechanistic-driven optimization.

Catalyst Synthesis and Characterization - Unveiling Metal-Support Interfacial Sites through Advanced Microscopy

The presented literature[6,46,67,79,83,97,108-111] demonstrates advanced microscopy techniques revealing crucial insights into metal-support interfacial sites in propane dehydrogenation catalysts:

Explicit examples establish capabilities of techniques like AC-HAADF-STEM, iDPC-STEM and EDS mapping for directly visualizing isolated metal atoms, clusters and nanoparticles tethered

to supports like zeolites and mesoporous oxides [46,67,108]. Imaging paired with elemental analysis charts spatial distribution and morphology of active phases, tracing variations through synthesis, pretreatment and cycling [6,109,110]. Quantitative image analysis takes this further by enabling statistics on dispersed site percentages and dimensions[83]. Atomic-scale views reveal distinct facets and precise loci within micropores and conduits, directly linking structure with performance [97,111]. Via operando capabilities, microscopy monitors dynamic restructuring and component interactions during reactions [79].

Integrative understanding and prospective outlook: The microscopy conclusions evidence appreciable command in constructing isolated and reinforced sites, enduring even post high-temperature processing. However, uncertainties persist regarding extending this thermal fortitude to industrially-relevant reaction ambiences. While images probe near-surface zones, bulk probes like XAS evaluate deeper strata. Reconciling both vantages will elucidate deactivation origins. Expanding microscopy to tilted, tomographic capture introduces 3D perspective. Operando cells visualizing functional catalysts under process conditions, coupled with spectroscopy and diffraction, will elucidate functionality-structure interdependencies. Assimilating theory and simulations to construe observed motifs proffers mechanistic pillars for rational design. Steady refinements in resolution, sensitivity and automation provide grounds for optimism in disentangling metal-support interfacial intricacies through advanced imaging.

Catalyst Synthesis and Characterization - Elucidating Oxidation States During Catalysis Using X-Ray Techniques

The presented literature[39] harnessed X-ray absorption spectroscopy avenues encompassing XANES and EXAFS conducted at a synchrotron radiation facility on pristine and expired Co@Si-BEA catalysts to probe oxidation state variations during propane dehydrogenation catalysis. FT-EXAFS and WT-EXAFS spectra of the spent catalyst still displayed a singular signal matching the inaugural Co-O bond shell with an almost invariable coordination environment (CN = 5.7 ± 0.3), affirming the implanted Co2+ cations constituting predominant Co active sites were structurally resilient against the reaction[39].

Additional works highlighted in [8] and[102] exhibited no X-ray grounded analysis of oxidation state fluctuations during propane dehydrogenation catalysis, rather harnessing computational modeling and catalyst assessment avenues.

Supplementary substantiation of harnessing synchrotron X-ray radiation to scrutinize metal species during propane dehydrogenation was provided by[3]. In situ XANES experiments portrayed how Ga entities migrate into zeolite conduits post H2 reduction. And quantitative pulse titration verified divalent Ga2O2^2+ as the precursor towards reactive GaHx sites.

Integrative understanding and prospective outlook: Taking into consideration the advancements and revelations examined, synchrotron radiation sources enable operando and in-situ examination of catalytic arrangements under bona fide reaction settings. Developing on extant capabilities, prospective undertakings could harness machine learning for automated analysis of extensive data flows. And momentum towards compact X-ray configurations interfaced with bench-scale reactors in academic laboratories. Broader assimilation necessitates standardized protocols and shared data formats. Comprehending dynamic transformations in oxidation states and coordination geometries persists being imperative for rational catalyst conception.

Reactors and Process Considerations - Exploring Reactor Configurations: Fixed Bed, Fluidized Bed, and Membrane

A meticulous literature interrogation revealed most academic contributions do not quantify different reactor configurations for propane dehydrogenation, rather concentrating on catalyst synthesis, characterization and evaluation[112].

Scarce exceptions contributing insights on reactor varieties encompass:

- Reference[112] examines commercially accessible reactors like Oleflex, Houdry Catofin, Phillips STAR and Linde processes alongside two-zone fluidized bed reactors (TZFBR) and internal circulating fluidized bed reactors (ICFBR) for propane dehydrogenation over Cr2O3/Al2O3 catalysts with performance contrasts across configurations.

- Reference[113] gauges silica membrane reactors to refine propene yields by selective hydrogen elimination during propane dehydrogenation, evidencing higher yields than fixed beds albeit accelerated catalyst deactivation.

- Reference [114] amalgamates two-zone fluidized bed reactors (TZFBR) with Pd membrane reactors for the first instance. The membrane elevated conversion but also coking rates over PtSn/Al2O3 catalysts.

Collectively, most academic contributions harness fixed bed reactors with sparse instances of fluidized beds and membrane reactors for propane dehydrogenation unveiling opportunities for supplemental configurations.

Reactors and Process Considerations - Applying Process Intensification in Propane Dehydrogenation

An exhaustive literature review[6,7,14,18,20,23,62,72,79,83,100,104,109,115-125] revealed the references predominantly focus on catalyst architectural motifs, surface chemistry and evaluation to elevate propane dehydrogenation credentials without analyzing process intensification perspectives.

Integrative understanding and prospective outlook: The meticulous cataloging exposes extant literature is overwhelmingly centered on incremental catalyst enhancements and structural metamorphosis. Nonetheless, promising opportunities become apparent for implementing process intensification techniques to radically transform the holistic system dynamics and efficiency. Prospective quests could prioritize intensified reactor configurations, advanced separation methodologies and process integration to dramatically augment productivity and economics. Moreover, the alliance of process and catalyst revelations promises to usher in a pioneering generation of ultra-compact, agile and productive propane dehydrogenation plants.

Reactors and Process Considerations - Techno-Economic Factors in Process Viability

A meticulous literature analysis[6,7,14,18,20,23,62,72,79,83,100,104,109,115-125] demonstrated the references constitute scientific examinations on catalyst and process essentials rather than techno-economic assessments.

Integrative understanding and prospective outlook: The reviewed literature institutes profound discernments into propane dehydrogenation catalyst architectural motifs and performance. However, translating these upgrades into commercially feasible processes necessitates quantitative economic viability appraisals. Upcoming quests could accentuate conducting holistic techno-economic gauging of pioneering propane dehydrogenation trajectories, assimilating catalytic revelations within viable process configurations. Computationally evaluating integrated procedures under adaptable settings would elucidate optimal avenues conjugating technical and economic performance. Ultimately, unifying catalytic revelation with process systems enhancement will propel propane dehydrogenation technologies from laboratory precincts towards widespread integration.

Reactors and Process Considerations - Safety Measures Against Flammability and Explosions

The scrutinized literature[6,7,14,18,20,23,62,72,79,83,100,104,109,115-125] did not analyze safety factors like flammability and detonation risks associated with propane dehydrogenation. The academic contributions were centered on catalyst configuration, characterization and gauging without examining process safety attributes.

Integrative understanding and prospective outlook: Despite the paucity of explicit analyses into safety facets heretofore, propane dehydrogenation arrangements warrant meticulous appraisal of flammability and explosion hazards owing to hydrocarbon feed and intermediate involvement. As this domain advances further, instituting rigorous protocols to preclude runaway reactions and contain explosions will be imperative. Forthcoming quests could harness perspectives from allied petrochemical processes to enact preemptive measures against accidents. On the catalyst

development front-end, crafting resilient and selective single-atom site catalysts with optimized thermal regulation could mitigate safety issues. Overall, consolidating process safety knowledge from adjacent realms alongside innovating upon catalyst and reactor fundamentals appears a prudent pathway.

Conclusion and Future Outlook - Addressing Challenges and Knowledge Gaps

A meticulous literature investigation[6,7,18,62,100,104,116,121] identified remaining challenges and erudition lacunae in propane dehydrogenation catalysis pursuits encompassing:

One principal challenge summarized is developing alternatives to precious platinum-anchored industrial catalysts as a protracted objective for propane dehydrogenation[116] . Relatedly, probing non-precious metal molecular sieve catalysts is portrayed as necessitating additional academic efforts[6] .

Regarding specific catalyst constituents, unambiguously pinpointing the most reactive chromium [62] and gallium entities [104] is noted as hitherto ambiguous and contentious, calling for supplemental characterization and definitive substantiation. Additionally, comprehending promoter effects like oxygen vacancies on catalyst performance epitomizes an erudition gap[14].

Several manuscripts also accentuate necessity to redress catalyst deactivation facets like coking[121] and thermal deactivation [18] towards augmenting stability. Investigating isolated metal atom and confined cluster morphologies is portrayed as a prospective solution[7].

From a process perspective, requirements for more energy efficient propane dehydrogenation technologies are stressed [100], potentially via computational screening of active sites and reactions[6]. Surpassing equilibrium constraints and minimizing unwanted side-reactions are also denoted[100] as pivotal advancement directions.

Overall, cardinal erudition deficits revolve around unambiguously identifying reactive loci, redressing stability and deactivation facets, supplanting precious metals, elevating energy efficiency and harnessing computational modalities – with ample latitude remaining to enhance prevailing commercial technologies.

Conclusion and Future Outlook - Catalyzing Progress with High-Throughput Computations

A contemporary examination harnessing a descriptor-anchored microkinetic assessment system consolidated with DFT renderings for expediting potential intermetallic compound catalyst screening for propane dehydrogenation (PDH) [20]. By appraising both terrace and edge orientations, over 2000 catalyst candidates were gauged. This high-throughput computational screening pinpointed inexpensive and eco-friendly Fe-Ga intermetallics as encouraging PDH catalysts. Additional academic contributions have initiated exploiting machine learning for

accelerating screening and architectural motifs of metallic heterogeneous catalysts. With inventories of computed descriptors persisting to proliferate, data-driven tactics can efficiently traverse extensive materials domains[117] . Additional expansion of this framework to trimetallic amalgams may unravel even more active and selective PDH catalysts.

Integrative understanding and prospective outlook: The examined advancements in high-throughput computational catalyst screening are poised to metamorphose propane dehydrogenation pursuits. Expeditious appraisals of thousands of candidates harnessing descriptors and machine learning archetypes can orient experimental validation towards the most encouraging catalyst formulations. Progressing forward, integration of multi-fidelity models, uncertainty quantification and autonomous experimentation into computational workflows could further augment the velocity and dependability of revelations. More widespread adoption of these modalities beyond metals to screen novel supports, promoters and processing ambiences may unravel generalizable architectural maxims for superior PDH catalysts.

Conclusion and Future Outlook - Advancing Catalyst Understanding with In-Situ Characterization

The manuscript itemized in reference [115] highlights altering silica supports via an in-situ emulsion avenue to decorate silica with MnOx, enabling configuration of exceptionally dispersed Pt-anchored catalysts for propane dehydrogenation.

References[7,14,18,20,23,62,72,79,83,104,109,116-125] did not accentuate necessities for refined in-situ characterization implements.

Reference[100] pinpoints prospects for harnessing time-resolved UV-vis and Raman spectroscopies for in-situ monitoring of industrial chromia-alumina catalysts towards elevating propane dehydrogenation efficiency.

Reference[6] stresses requirements for sophisticated in-situ characterization techniques like iDPC-STEM, in-situ spectroscopy and 2D solid-state NMR to advance comprehension of propane dehydrogenation catalysts.

Integrative understanding and prospective outlook: The cataloging of propane dehydrogenation catalyst literature unveils sporadic instances accentuating prospects for cutting-edge in-situ diagnostics. While maximum academic contributions harness traditional ex-situ modalities, emergent techniques like iDPC-STEM, time-resolved optical probes and 2D NMR may unlock deeper insights via real-time tracking of functional catalysts. Upcoming quests might prioritize cultivating bespoke analytical assets attuned to unveiling transient phenomena regulating activity, selectivity and deactivation pathways. Assembling realistic reaction settings within characterization implements could illuminate intrinsic mechanisms steering commercial reactors. Multimodal in-situ facilities coalescing perspectives from complementary techniques also tender

information-rich lenses into catalytic performance. Ultimately, breakthroughs in elemental comprehension hinge on unlocking novel observational capabilities probing catalysts at nanoscopic strata while inserted in dynamic reaction ambiences.

Conclusion and Future Outlook - Envisioning Transformational Catalyst Architectures for Future Breakthroughs

A meticulous literature dissection revealed the references generally do not envisage potentially transformational catalyst architectures for propane dehydrogenation, rather concentrating on incremental refinements to prevailing catalyst substances and configurations[6,14,18,20,23,62,72,79,83,100,104,109,115-125].

One academic contribution suggests emerging single atom and confined cluster morphologies as potentially disruptive concepts that could assist minimizing coke accrual[7]. Overall, forward-gazing discourse on unprecedented architectures with potential to elicit transformational propane dehydrogenation breakthroughs remains lacking.

Integrative understanding and prospective outlook: The current propane dehydrogenation catalysis academic landscape appears predominantly centered on incremental advancements rather than disruptive revelation. Nonetheless, the isolated metal atom and confined cluster proposition in one manuscript[7] highlights the promise of such pioneering configurations for tackling facets like coking. Future academic contributions could expand materials exploration to encompass emerging scaffolds like metal-organic frameworks, covalent organic frameworks and heterogeneous photocatalysts. Alloying computational screening with high-throughput experimentation may also quicken the discovery of unheralded active site motifs. An interdisciplinary approach coalescing chemical engineering, materials science, heterogeneous catalysis and computational chemistry may unveil entirely novel architectural maxims laying the pillars for next-generation, transformative propane dehydrogenation arrangements.

References:

[1] Zhang Y, Zhao Y, Otroshchenko T, et al.Structure–Activity–Selectivity Relationships in Propane Dehydrogenation over Rh/ZrO2 Catalysts[J].ACS Catalysis,2020, 10 (11): 6377-6388. doi.org/10.1021/acscatal.0c01455

[2] Rigamonti M G, Shah M, Gambu T G, et al.Reshaping the Role of CO2 in Propane Dehydrogenation: From Waste Gas to Platform Chemical[J].ACS Catalysis,2022, 12 (15): 9339-9358.doi.org/10.1021/acscatal.2c01374

[3] Yuan Y, Zhao Z, Lobo R F, et al.Site Diversity and Mechanism of Metal‐Exchanged Zeolite Catalyzed Non‐Oxidative Propane Dehydrogenation[J].Advanced Science,2023, 10 (13).doi.org/10.1002/advs.202207756

[4] Jiang X, Sharma L, Fung V, et al.Oxidative Dehydrogenation of Propane to Propylene with Soft Oxidants via Heterogeneous Catalysis[J].ACS Catalysis,2021, 11 (4): 2182-2234.doi.org/10.1021/acscatal.0c03999

[5] Dong C, Lai Z, Wang H.Comprehensive Mechanism and Microkinetic Model-Driven Rational Screening of 3N-Modulated Single-Atom Catalysts for Propane Dehydrogenation[J].ACS Catalysis,2023, 13 (8): 5529-5537.doi.org/10.1021/acscatal.3c00889

[6] Song S, Sun Y, Yang K, et al.Recent Progress in Metal-Molecular Sieve Catalysts for Propane Dehydrogenation[J].ACS Catalysis,2023, 13 (9): 6044-6067.doi.org/10.1021/acscatal.3c00816

[7] Lian Z, Si C, Jan F, et al.Coke Deposition on Pt-Based Catalysts in Propane Direct Dehydrogenation: Kinetics, Suppression, and Elimination[J].ACS Catalysis,2021, 11 (15): 9279-9292.doi.org/10.1021/acscatal.1c00331

[8] Fricke C H, Bamidele O H, Bello M, et al.Modeling the Effect of Surface Platinum–Tin Alloys on Propane Dehydrogenation on Platinum–Tin Catalysts[J].ACS Catalysis,2023, 13 (16): 10627-10640.doi.org/10.1021/acscatal.3c00939

[9] Yuan Y, Lee J S, Lobo R F.Ga+-Chabazite Zeolite: A Highly Selective Catalyst for Nonoxidative Propane Dehydrogenation[J].Journal of the American Chemical Society,2022, 144 (33): 15079-15092.doi.org/10.1021/jacs.2c03941

[10] Seemakurthi R R, Canning G, Wu Z, et al.Identification of a Selectivity Descriptor for Propane Dehydrogenation through Density Functional and Microkinetic Analysis on Pure Pd and Pd Alloys[J].ACS Catalysis,2021, 11 (15): 9588-9604.doi.org/10.1021/acscatal.1c01916

[11] Xing Y, Bi G, Pan X, et al.Sub-nanometer Pt2In3 intermetallics as ultra-stable catalyst for propane dehydrogenation[J].Journal of Energy Chemistry,2023, 83: 304-312.doi.org/10.1016/j.jechem.2023.04.019

[12] Zhu J, Yang M-L, Yu Y, et al.Size-Dependent Reaction Mechanism and Kinetics for Propane Dehydrogenation over Pt Catalysts[J].ACS Catalysis,2015, 5 (11): 6310-6319.doi.org/10.1021/acscatal.5b01423

[13] Raman N, Wolf M, Heller M, et al.GaPt Supported Catalytically Active Liquid Metal Solution Catalysis for Propane Dehydrogenation–Support Influence and Coking Studies[J].ACS Catalysis,2021, 11 (21): 13423-13433.doi.org/10.1021/acscatal.1c01924

[14] Xu Y, Wang X, Yang D, et al.Stabilizing Oxygen Vacancies in ZrO2 by Ga2O3 Boosts the Direct Dehydrogenation of Light Alkanes[J].ACS Catalysis,2021, 11 (16): 10159-10169.doi.org/10.1021/acscatal.1c02621

[15] Chang Q-Y, Wang K-Q, Sui Z-J, et al.Rational Design of Single-Atom-Doped Ga2O3 Catalysts for Propane Dehydrogenation: Breaking through Volcano Plot by Lewis Acid–Base Interactions[J].ACS Catalysis,2021, 11 (9): 5135-5147.doi.org/10.1021/acscatal.0c05454

[16] Yue Y, Fu J, Wang C, et al.Propane dehydrogenation catalyzed by single Lewis acid site in Sn-Beta zeolite[J].Journal of Catalysis,2021, 395: 155-167.doi.org/10.1016/j.jcat.2020.12.019

[17] Sun X, Liu M, Huang Y, et al.Electronic interaction between single Pt atom and vacancies on boron nitride nanosheets and its influence on the catalytic performance in the direct dehydrogenation of propane[J].Chinese Journal of Catalysis,2019, 40 (6): 819-825.doi.org/10.1016/S1872-2067(18)63196-1

[18] Sun M-L, Hu Z-P, Wang H-Y, et al.Design Strategies of Stable Catalysts for Propane Dehydrogenation to Propylene[J].ACS Catalysis,2023, 13 (7): 4719-4741.doi.org/10.1021/acscatal.3c00103

[19] Benco L, Bucko T, Hafner J.Dehydrogenation of propane over ZnMOR. Static and dynamic reaction energy diagram[J].Journal of Catalysis,2011, 277 (1): 104-116.doi.org/10.1016/j.jcat.2010.10.018

[20] Xiao L, Hu P, Sui Z-J, et al.Rational design of intermetallic compound catalysts for propane dehydrogenation from a descriptor-based microkinetic analysis[J].Journal of Catalysis,2021, 404: 32-45.doi.org/10.1016/j.jcat.2021.09.008

[21] Castro-Fernández P, Mance D, Liu C, et al.Propane Dehydrogenation on Ga2O3-Based Catalysts: Contrasting Performance with Coordination Environment and Acidity of Surface Sites[J].ACS Catalysis,2021, 11 (2): 907-924.doi.org/10.1021/acscatal.0c05009

[22] Dou J, Funderburg J, Yang K, et al.CexZr1－xO2-Supported CrOx Catalysts for CO2-Assisted Oxidative Dehydrogenation of Propane—Probing the Active Sites and Strategies for Enhanced Stability[J].ACS Catalysis,2022, 13 (1): 213-223.doi.org/10.1021/acscatal.2c05286

[23] Huš M, Kopač D, Bajec D, et al.Effect of Surface Oxidation on Oxidative Propane Dehydrogenation over Chromia: An Ab Initio Multiscale Kinetic Study[J].ACS Catalysis,2021, 11 (17): 11233-11247.doi.org/10.1021/acscatal.1c01814

[24] Xie Z, Li Z, Tang P, et al.The effect of oxygen vacancies on the coordinatively unsaturated Al-O acid-base pairs for propane dehydrogenation[J].Journal of Catalysis,2021, 397: 172-182.doi.org/10.1016/j.jcat.2021.03.033

[25] Shan Y-L, Sun H-L, Zhao S-L, et al.Effects of Support and CO2 on the Performances of Vanadium Oxide-Based Catalysts in Propane Dehydrogenation[J].ACS Catalysis,2022, 12 (10): 5736-5749.doi.org/10.1021/acscatal.2c00878

[26] Derossi S, Ferraris G, Fremiotti S, et al.Propane Dehydrogenation on Chromia/Silica and Chromia/Alumina Catalysts[J].Journal of Catalysis,1994, 148 (1): 36-46.doi.org/10.1006/jcat.1994.1183

[27] Otroshchenko T, Sokolov S, Stoyanova M, et al.ZrO2 ‐ Based Alternatives to Conventional Propane Dehydrogenation Catalysts: Active Sites, Design, and Performance[J].Angewandte Chemie International Edition,2015, 54 (52): 15880-15883.doi.org/10.1002/anie.201508731

[28] Xu Y, Chen S, Chang X, et al.Ultrathin TiOx Nanosheets Rich in Tetracoordinated Ti Sites for Propane Dehydrogenation[J].ACS Catalysis,2023, 13 (9): 6104-6113.doi.org/10.1021/acscatal.3c00818

[29] Xu B, Zheng B, Hua W, et al.Support effect in dehydrogenation of propane in the presence of CO2 over supported gallium oxide catalysts[J].Journal of Catalysis,2006, 239 (2): 470-477.doi.org/10.1016/j.jcat.2006.02.017

[30] Chen K, Khodakov A, Yang J, et al.Isotopic Tracer and Kinetic Studies of Oxidative Dehydrogenation Pathways on Vanadium Oxide Catalysts[J].Journal of Catalysis,1999, 186 (2): 325-333.doi.org/10.1006/jcat.1999.2510

[31] Pless J.Catalytic oxidative dehydrogenation of propane over Mg–V/Mo oxides[J].Journal of Catalysis,2004, 223 (2): 419-431.doi.org/10.1016/j.jcat.2004.01.023

[32] Chen S, Zeng L, Mu R, et al.Modulating Lattice Oxygen in Dual-Functional Mo–V–O Mixed Oxides for Chemical Looping Oxidative Dehydrogenation[J].Journal of the American Chemical Society,2019, 141 (47): 18653-18657.doi.org/10.1021/jacs.9b09235

[33] Han S, Zhao Y, Otroshchenko T, et al.Unraveling the Origins of the Synergy Effect between ZrO2 and CrOx in Supported CrZrOx for Propene Formation in Nonoxidative Propane Dehydrogenation[J].ACS Catalysis,2019, 10 (2): 1575-1590.doi.org/10.1021/acscatal.9b05063

[34] Qu Y, Li G, Zhao T, et al.Low-Temperature Direct Dehydrogenation of Propane over Binary Oxide Catalysts: Insights into Geometric Effects and Active Sites[J].ACS Sustainable Chemistry & Engineering,2021, 9 (38): 12755-12765.doi.org/10.1021/acssuschemeng.1c03074

[35] Yuan Y, Brady C, Lobo R F, et al.Understanding the Correlation between Ga Speciation and Propane Dehydrogenation Activity on Ga/H-ZSM-5 Catalysts[J].ACS Catalysis,2021, 11 (16): 10647-10659.doi.org/10.1021/acscatal.1c01497

[36] Rossetti I, Fabbrini L, Ballarini N, et al.V2O5–SiO2 systems prepared by flame pyrolysis as catalysts for the oxidative dehydrogenation of propane[J].Journal of Catalysis,2008, 256 (1): 45-61.doi.org/10.1016/j.jcat.2008.02.028

[37] Zheng B, Hua W, Yue Y, et al.Dehydrogenation of propane to propene over different polymorphs of gallium oxide[J].Journal of Catalysis,2005, 232 (1): 143-151.doi.org/10.1016/j.jcat.2005.03.001

[38] Grant J T, Carrero C A, Love A M, et al.Enhanced Two-Dimensional Dispersion of Group V Metal Oxides on Silica[J].ACS Catalysis,2015, 5 (10): 5787-5793.doi.org/10.1021/acscatal.5b01679

[39] Wei S, Dai H, Long J, et al.Nonoxidative propane dehydrogenation by isolated Co2+ in BEA zeolite: Dealumination-determined key steps of propane C H activation and propylene desorption[J].Chemical Engineering Journal,2023, 455: 140726.doi.org/10.1016/j.cej.2022.140726

[40] Wang J, Chang X, Chen S, et al.On the Role of Sn Segregation of Pt-Sn Catalysts for Propane Dehydrogenation[J].ACS Catalysis,2021, 11 (8): 4401-4410.doi.org/10.1021/acscatal.1c00639

[41] Jackson S D, Leeming P, Grenfell J.The Effect of Sulfur on the Nonsteady State Reaction of Propane over a Platinum/Alumina Catalyst at 873 K[J].Journal of Catalysis,1994, 150 (1): 170-176.doi.org/10.1006/jcat.1994.1333

[42] Wang Y, Wang J, Zheng P, et al.Boosting selectivity and stability on Pt/BN catalysts for propane dehydrogenation via calcination & reduction-mediated strong metal-support interaction[J].Journal of Energy Chemistry,2022, 67: 451-457.doi.org/10.1016/j.jechem.2021.10.008

[43] Zhu J, Wang T, Xu X, et al.Pt nanoparticles supported on SBA-15: Synthesis, characterization and applications in heterogeneous catalysis[J].Applied Catalysis B: Environmental,2013, 130-131: 197-217.doi.org/10.1016/j.apcatb.2012.11.005

[44] Lu Z, Tracy R W, Abrams M L, et al.Atomic Layer Deposition Overcoating Improves Catalyst Selectivity and Longevity in Propane Dehydrogenation[J].ACS Catalysis,2020, 10 (23): 13957-13967.doi.org/10.1021/acscatal.0c03391

[45] Aly M, Fornero E L, Leon-Garzon A R, et al.Effect of Boron Promotion on Coke Formation during Propane Dehydrogenation over Pt/γ-Al2O3 Catalysts[J].ACS Catalysis,2020, 10 (9): 5208-5216.doi.org/10.1021/acscatal.9b05548

[46] Ma Y, Song S, Liu C, et al.Germanium-enriched double-four-membered-ring units inducing zeolite-confined subnanometric Pt clusters for efficient propane dehydrogenation[J].Nature Catalysis,2023, 6 (6): 506-518.doi.org/10.1038/s41929-023-00968-7

[47] Zhang Y, Zhou Y, Huang L, et al.Structure and catalytic properties of the Zn-modified ZSM-5 supported platinum catalyst for propane dehydrogenation[J].Chemical Engineering Journal,2015, 270: 352-361.doi.org/10.1016/j.cej.2015.01.008

[48] Long L-L, Lang W-Z, Liu X, et al.Improved catalytic stability of PtSnIn/xCa–Al catalysts for propane dehydrogenation to propylene[J].Chemical Engineering Journal,2014, 257: 209-217.doi.org/10.1016/j.cej.2014.07.044

[49] Qi L, Zhang Y, Babucci M, et al.Dehydrogenation of Propane and n-Butane Catalyzed by Isolated PtZn4 Sites Supported on Self-Pillared Zeolite Pentasil Nanosheets[J].ACS Catalysis,2022, 12 (18): 11177-11189.doi.org/10.1021/acscatal.2c01631

[50] Nakaya Y, Hirayama J, Yamazoe S, et al.Single-atom Pt in intermetallics as an ultrastable and selective catalyst for propane dehydrogenation[J].Nature Communications,2020, 11 (1).doi.org/10.1038/s41467-020-16693-9

[51] Zhang Y, Yu Y, Dai Y, et al.Regulating the C–H Bond Activation Pathway over ZrO2 via Doping Engineering for Propane Dehydrogenation[J].ACS Catalysis,2023, 13 (10): 6893-6904.doi.org/10.1021/acscatal.3c01002

[52] Hannagan R T, Giannakakis G, Réocreux R, et al.First-principles design of a single-atom–alloy propane dehydrogenation catalyst[J].Science,2021, 372 (6549): 1444-1447.doi.org/10.1126/science.abg8389

[53] Rennard R.The role of sulfur in deactivation of Pt/MgAl2O4 for propane dehydrogenation[J].Journal of Catalysis,1986, 98 (2): 235-244.doi.org/10.1016/0021-9517(86)90312-X

[54] Ma R, Yang T, Gao J, et al.Composition Tuning of Ru-Based Phosphide for Enhanced Propane Selective Dehydrogenation[J].ACS Catalysis,2020, 10 (17): 10243-10252.doi.org/10.1021/acscatal.0c01667

[55] Xiao L, Shan Y-L, Sui Z-J, et al.Beyond the Reverse Horiuti–Polanyi Mechanism in Propane Dehydrogenation over Pt Catalysts[J].ACS Catalysis,2020, 10 (24): 14887-14902.doi.org/10.1021/acscatal.0c03381

[56] Estes D P, Siddiqi G, Allouche F, et al.C–H Activation on Co,O Sites: Isolated Surface Sites versus Molecular Analogs[J].Journal of the American Chemical Society,2016, 138 (45): 14987-14997.doi.org/10.1021/jacs.6b08705

[57] Han S W, Park H, Han J, et al.PtZn Intermetallic Compound Nanoparticles in Mesoporous Zeolite Exhibiting High Catalyst Durability for Propane Dehydrogenation[J].ACS Catalysis,2021, 11 (15): 9233-9241.doi.org/10.1021/acscatal.1c01808

[58] Wang P, Yao J, Jiang Q, et al.Stabilizing the isolated Pt sites on PtGa/Al2O3 catalyst via silica coating layers for propane dehydrogenation at low temperature[J].Applied Catalysis B: Environmental,2022, 300: 120731.doi.org/10.1016/j.apcatb.2021.120731

[59] Zhu Y, An Z, Song H, et al.Lattice-Confined Sn (IV/II) Stabilizing Raft-Like Pt Clusters: High Selectivity and Durability in Propane Dehydrogenation[J].ACS Catalysis,2017, 7 (10): 6973-6978.doi.org/10.1021/acscatal.7b02264

[60] Zhu X, Wang T, Xu Z, et al.Pt-Sn clusters anchored at Al3+penta sites as a sinter-resistant and regenerable catalyst for propane dehydrogenation[J].Journal of Energy Chemistry,2022, 65: 293-301.doi.org/10.1016/j.jechem.2021.06.002

[61] Li J, Li J, Zhao Z, et al.Size effect of TS-1 supports on the catalytic performance of PtSn/TS-1 catalysts for propane dehydrogenation[J].Journal of Catalysis,2017, 352: 361-370.doi.org/10.1016/j.jcat.2017.05.024

[62] Santhoshkumar M, Hammer N, Ronning M, et al.The nature of active chromium species in Cr-catalysts for dehydrogenation of propane: New insights by a comprehensive spectroscopic study[J].Journal of Catalysis,2009, 261 (1): 116-128.doi.org/10.1016/j.jcat.2008.11.014

[63] Han Z-F, Xue X-L, Wu J-M, et al.Preparation and catalytic properties of mesoporous n V-MCM-41 for propane oxidative dehydrogenation in the presence of CO 2[J].Chinese Journal of Catalysis,2018, 39 (6): 1099-1109.doi.org/10.1016/S1872-2067(18)63048-7

[64] Wan H, Qian L, Gong N, et al.Size-Dependent Structures and Catalytic Properties of Supported Bimetallic PtSn Catalysts for Propane Dehydrogenation Reaction[J].ACS Catalysis,2023, 13 (11): 7383-7394.doi.org/10.1021/acscatal.3c00548

[65] Liu X, Lang W-Z, Long L-L, et al.Improved catalytic performance in propane dehydrogenation of PtSn/γ-Al2O3 catalysts by doping indium[J].Chemical Engineering Journal,2014, 247: 183-192.doi.org/10.1016/j.cej.2014.02.084

[66] Liu Y, Zong X, Patra A, et al.Propane Dehydrogenation on PtxSny (x, y ≤ 4) Clusters on Al2O3(110)[J].ACS Catalysis,2023, 13 (5): 2802-2812.doi.org/10.1021/acscatal.2c05671

[67] Zhang B, Li G, Liu S, et al.Boosting Propane Dehydrogenation over PtZn Encapsulated in an Epitaxial High-Crystallized Zeolite with a Low Surface Barrier[J].ACS Catalysis,2022, 12 (2): 1310-1314.doi.org/10.1021/acscatal.1c04092

[68] Xie L, Chai Y, Sun L, et al.Optimizing zeolite stabilized Pt-Zn catalysts for propane dehydrogenation[J].Journal of Energy Chemistry,2021, 57: 92-98.doi.org/10.1016/j.jechem.2020.08.058

[69] Qi L, Babucci M, Zhang Y, et al.Propane Dehydrogenation Catalyzed by Isolated Pt Atoms in ≡SiOZn−OH Nests in Dealuminated Zeolite Beta[J].Journal of the American Chemical Society,2021, 143 (50): 21364-21378.doi.org/10.1021/jacs.1c10261

[70] Sun C, Luo J, Cao M, et al.A comparative study on different regeneration processes of Pt-Sn/γ-Al2O3 catalysts for propane dehydrogenation[J].Journal of Energy Chemistry,2018, 27 (1): 311-318.doi.org/10.1016/j.jechem.2017.09.035

[71] Ma S, Liu Z-P.Zeolite-confined subnanometric PtSn mimicking mortise-and-tenon joinery for catalytic propane dehydrogenation[J].Nature Communications,2022, 13 (1).doi.org/10.1038/s41467-022-30522-1

[72] Natarajan P, Khan H A, Jaleel A, et al.The pronounced effect of Sn on RhSn catalysts for propane dehydrogenation[J].Journal of Catalysis,2020, 392: 8-20.doi.org/10.1016/j.jcat.2020.09.016

[73] Kaylor N, Davis R J.Propane dehydrogenation over supported Pt-Sn nanoparticles[J].Journal of Catalysis,2018, 367: 181-193.doi.org/10.1016/j.jcat.2018.09.006

[74] Li Z, Yu L, Milligan C, et al.Two-dimensional transition metal carbides as supports for tuning the chemistry of catalytic nanoparticles[J].Nature Communications,2018, 9 (1).doi.org/10.1038/s41467-018-07502-5

[75] Wang Y, Suo Y, Lv X, et al.Enhanced performances of bimetallic Ga-Pt nanoclusters confined within silicalite-1 zeolite in propane dehydrogenation[J].Journal of Colloid and Interface Science,2021, 593: 304-314.doi.org/10.1016/j.jcis.2021.02.129

[76] Zhang W, Zhang X, Wang J, et al.Bismuth-Modulated Surface Structural Evolution of Pd3Bi Intermetallic Alloy Catalysts for Selective Propane Dehydrogenation and Acetylene Semihydrogenation[J].ACS Catalysis,2022, 12 (17): 10531-10545.doi.org/10.1021/acscatal.2c00642

[77] Shan Y, Sui Z, Zhu Y, et al.Boosting Size‐Selective Hydrogen Combustion in the Presence of Propene Using Controllable Metal Clusters Encapsulated in Zeolite[J].Angewandte Chemie International Edition,2018, 57 (31): 9770-9774.doi.org/10.1002/anie.201805150

[78] Wang G, Feng X.Response of Plants' Water Use Efficiency to Increasing Atmospheric CO2 Concentration[J].Environmental Science & Technology,2012, 46 (16): 8610-8620.doi.org/10.1021/es301323m

[79] Liu H, Zhou J, Chen T, et al.Isolated Pt Species Anchored by Hierarchical-like Heteroatomic Fe-Silicalite-1 Catalyze Propane Dehydrogenation near the Thermodynamic Limit[J].ACS Catalysis,2023, 13 (5): 2928-2936.doi.org/10.1021/acscatal.2c05042

[80] Shi L, Deng G M, Li W C, et al.Al2O3 Nanosheets Rich in Pentacoordinate Al3+ Ions Stabilize Pt‐Sn Clusters for Propane Dehydrogenation[J].Angewandte Chemie International Edition,2015, 54 (47): 13994-13998.doi.org/10.1002/anie.201507119

[81] Escorcia N J, Libretto N J, Miller J T, et al.Colloidal Synthesis of Well-Defined Bimetallic Nanoparticles for Nonoxidative Alkane Dehydrogenation[J].ACS Catalysis,2020, 10 (17): 9813-9823.doi.org/10.1021/acscatal.0c01554

[82] Smirnov A V, Mazin E V, Yuschenko V V, et al.Benzene Alkylation with Propane over Pt-Modified MFI Zeolites[J].Journal of Catalysis,2000, 194 (2): 266-277.doi.org/10.1006/jcat.2000.2910

[83] Liu L, Lopez-Haro M, Lopes C W, et al.Structural modulation and direct measurement of subnanometric bimetallic PtSn clusters confined in zeolites[J].Nature Catalysis,2020, 3 (8): 628-638.doi.org/10.1038/s41929-020-0472-7

[84] Bariås O A, Holmen A, Blekkan E A.Propane Dehydrogenation over Supported Pt and Pt–Sn Catalysts: Catalyst Preparation, Characterization, and Activity Measurements[J].Journal of Catalysis,1996, 158 (1): 1-12.doi.org/10.1006/jcat.1996.0001

[85] Xie L, Wang R, Chai Y, et al.Propane dehydrogenation catalyzed by in-situ partially reduced zinc cations confined in zeolites[J].Journal of Energy Chemistry,2021, 63: 262-269.doi.org/10.1016/j.jechem.2021.04.034

[86] Chang X, Zhao Z-J, Lu Z, et al.Designing single-site alloy catalysts using a degree-of-isolation descriptor[J].Nature Nanotechnology,2023, 18 (6): 611-616.doi.org/10.1038/s41565-023-01344-z

[87] Searles K, Chan K W, Mendes Burak J A, et al.Highly Productive Propane Dehydrogenation Catalyst Using Silica-Supported Ga–Pt Nanoparticles Generated from Single-Sites[J].Journal of the American Chemical Society,2018, 140 (37): 11674-11679.doi.org/10.1021/jacs.8b05378

[88] Cheng X, Zhang Y, Wang J, et al.B–O Oligomers or Ring Species in AlB2: Which is More Selective for Propane Oxidative Dehydrogenation?[J].ACS Catalysis,2023, 13 (3): 1630-1637.doi.org/10.1021/acscatal.2c04889

[89] Wang W, Wu Y, Liu T, et al.Single Co Sites in Ordered SiO2 Channels for Boosting Nonoxidative Propane Dehydrogenation[J].ACS Catalysis,2022, 12 (4): 2632-2638.doi.org/10.1021/acscatal.1c05921

[90] Wu Z, Bukowski B C, Li Z, et al.Changes in Catalytic and Adsorptive Properties of 2 nm Pt3Mn Nanoparticles by Subsurface Atoms[J].Journal of the American Chemical Society,2018, 140 (44): 14870-14877.doi.org/10.1021/jacs.8b08162

[91] Wang Y, Hu Z-P, Lv X, et al.Ultrasmall PtZn bimetallic nanoclusters encapsulated in silicalite-1 zeolite with superior performance for propane dehydrogenation[J].Journal of Catalysis,2020, 385: 61-69.doi.org/10.1016/j.jcat.2020.02.019

[92] Zhai P, Xie Z, Huang E, et al.CO2-mediated oxidative dehydrogenation of propane enabled by Pt-based bimetallic catalysts[J].Chem,2023, 9 (11): 3268-3285.doi.org/10.1016/j.chempr.2023.07.002

[93] Ryoo R, Kim J, Jo C, et al.Rare-earth–platinum alloy nanoparticles in mesoporous zeolite for catalysis[J].Nature,2020, 585 (7824): 221-224.doi.org/10.1038/s41586-020-2671-4

[94] Xu Z, Yue Y, Bao X, et al.Propane Dehydrogenation over Pt Clusters Localized at the Sn Single-Site in Zeolite Framework[J].ACS Catalysis,2019, 10 (1): 818-828.doi.org/10.1021/acscatal.9b03527

[95] Song S, Yang K, Zhang P, et al.Silicalite-1 Stabilizes Zn-Hydride Species for Efficient Propane Dehydrogenation[J].ACS Catalysis,2022, 12 (10): 5997-6006.doi.org/10.1021/acscatal.2c00928

[96] Almutairi S M T, Mezari B, Magusin P C M M, et al.Structure and Reactivity of Zn-Modified ZSM-5 Zeolites: The Importance of Clustered Cationic Zn Complexes[J].ACS Catalysis,2011, 2 (1): 71-83.doi.org/10.1021/cs200441e

[97] Liu L, Díaz U, Arenal R, et al.Generation of subnanometric platinum with high stability during transformation of a 2D zeolite into 3D[J].Nature Materials,2016, 16 (1): 132-138.doi.org/10.1038/nmat4757

[98] Liu L.Trapped by the germanium ring[J].Nature Catalysis,2023, 6 (6): 462-463.doi.org/10.1038/s41929-023-00974-9

[99] Airaksinen S, Banares M, Krause A.In situ characterisation of carbon-containing species formed on chromia/alumina during propane dehydrogenation[J].Journal of Catalysis,2005, 230 (2): 507-513.doi.org/10.1016/j.jcat.2005.01.005

[100] Sattler J J H B, Ruiz-Martinez J, Santillan-Jimenez E, et al.Catalytic Dehydrogenation of Light Alkanes on Metals and Metal Oxides[J].Chemical Reviews,2014, 114 (20): 10613-10653.doi.org/10.1021/cr5002436

[101] Hu Z-P, Yang D, Wang Z, et al.State-of-the-art catalysts for direct dehydrogenation of propane to propylene[J].Chinese Journal of Catalysis,2019, 40 (9): 1233-1254.doi.org/10.1016/S1872-2067(19)63360-7

[102] Gao X, Liu M, Huang Y, et al.Dimensional Understanding of Boron-Based Catalysts for Oxidative Propane Dehydrogenation: Structure and Mechanism[J].ACS Catalysis,2023, 13 (14): 9667-9687.doi.org/10.1021/acscatal.3c00728

[103] Dai Y, Wu Y, Dai H, et al.Effect of coking and propylene adsorption on enhanced stability for Co2+-catalyzed propane dehydrogenation[J].Journal of Catalysis,2021, 395: 105-116.doi.org/10.1016/j.jcat.2020.12.021

[104] Yuan Y, Lobo R F, Xu B.Ga2O22+ Stabilized by Paired Framework Al Atoms in MFI: A Highly Reactive Site in Nonoxidative Propane Dehydrogenation[J].ACS Catalysis,2022, 12 (3): 1775-1783.doi.org/10.1021/acscatal.1c05724

[105] Zhao D, Gao M, Tian X, et al.Effect of Diffusion Constraints and ZnOx Speciation on Nonoxidative Dehydrogenation of Propane and Isobutane over ZnO-Containing Catalysts[J].ACS Catalysis,2023, 13 (5): 3356-3369.doi.org/10.1021/acscatal.2c05704

[106] Li Y, Yu X, Zhang Q, et al.The nature of VOx structures in HMS supported vanadium catalysts for non-oxidative propane dehydrogenation[J].Journal of Catalysis,2022, 413: 658-667.doi.org/10.1016/j.jcat.2022.07.017

[107] Xiong C, Chen S, Yang P, et al.Structure–Performance Relationships for Propane Dehydrogenation over Aluminum Supported Vanadium Oxide[J].ACS Catalysis,2019, 9 (7): 5816-5827.doi.org/10.1021/acscatal.8b04701

[108] Hu Z-P, Qin G, Han J, et al.Atomic Insight into the Local Structure and Microenvironment of Isolated Co-Motifs in MFI Zeolite Frameworks for Propane Dehydrogenation[J].Journal of the American Chemical Society,2022, 144 (27): 12127-12137.doi.org/10.1021/jacs.2c02636

[109] Xing Y, Kang L, Ma J, et al.Sn1Pt single-atom alloy evolved stable PtSn/nano-Al2O3 catalyst for propane dehydrogenation[J].Chinese Journal of Catalysis,2023, 48: 164-174.doi.org/10.1016/S1872-2067(23)64402-X

[110] Kwon H C, Park Y, Park J Y, et al.Catalytic Interplay of Ga, Pt, and Ce on the Alumina Surface Enabling High Activity, Selectivity, and Stability in Propane Dehydrogenation[J].ACS Catalysis,2021, 11 (17): 10767-10777.doi.org/10.1021/acscatal.1c02553

[111] Chen S, Zhao Z-J, Mu R, et al.Propane Dehydrogenation on Single-Site [PtZn4] Intermetallic Catalysts[J].Chem,2021, 7 (2): 387-405.doi.org/10.1016/j.chempr.2020.10.008

[112] Gascón J, Téllez C, Herguido J, et al.A two-zone fluidized bed reactor for catalytic propane dehydrogenation[J].Chemical Engineering Journal,2005, 106 (2): 91-96.doi.org/10.1016/j.cej.2004.11.005

[113] Schäfer R, Noack M, Kölsch P, et al.Development of a H2-selective SiO2-membrane for the catalytic dehydrogenation of propane[J].Separation and Purification Technology,2001, 25 (1-3): 3-9.doi.org/10.1016/S1383-5866(01)00085-5

[114] Gimeno M P, Wu Z T, Soler J, et al.Combination of a Two-Zone Fluidized Bed Reactor with a Pd hollow fibre membrane for catalytic alkane dehydrogenation[J].Chemical Engineering Journal,2009, 155 (1-2): 298-303.doi.org/10.1016/j.cej.2009.06.037

[115] Fan X, Liu D, Sun X, et al.Mn-doping induced changes in Pt dispersion and PtxMny alloying extent on Pt/Mn-DMSN catalyst with enhanced propane dehydrogenation stability[J].Journal of Catalysis,2020, 389: 450-460.doi.org/10.1016/j.jcat.2020.06.016

[116] Zhou Y, Wei F, Qi H, et al.Peripheral-nitrogen effects on the Ru1 centre for highly efficient propane dehydrogenation[J].Nature Catalysis,2022, 5 (12): 1145-1156.doi.org/10.1038/s41929-022-00885-1

[117] Wang T, Cui X, Winther K T, et al.Theory-Aided Discovery of Metallic Catalysts for Selective Propane Dehydrogenation to Propylene[J].ACS Catalysis,2021, 11 (10): 6290-6297.doi.org/10.1021/acscatal.0c05711

[118] Bravo-Suárez J J, Bando K K, Lu J, et al.Oxidation of propane to propylene oxide on gold catalysts[J].Journal of Catalysis,2008, 255 (1): 114-126.doi.org/10.1016/j.jcat.2008.01.030

[119] Guo M, Liu X, Amorelli A.Activation of small molecules over praseodymium-doped ceria[J].Chinese Journal of Catalysis,2019, 40 (11): 1800-1809.doi.org/10.1016/S1872-2067(19)63369-3

[120] Shen L-L, Xia K, Lang W-Z, et al.The effects of calcination temperature of support on PtIn/Mg(Al)O catalysts for propane dehydrogenation reaction[J].Chemical Engineering Journal,2017, 324: 336-346.doi.org/10.1016/j.cej.2017.05.058

[121] Perezramirez J.N2O-mediated propane oxidative dehydrogenation over steam-activated iron zeolites[J].Journal of Catalysis,2004, 223 (2): 382-388.doi.org/10.1016/j.jcat.2004.02.005

[122] Wu J C S, Lin S-J.Novel BN supported bi-metal catalyst for oxydehydrogenation of propane[J].Chemical Engineering Journal,2008, 140 (1-3): 391-397.doi.org/10.1016/j.cej.2007.11.009

[123] Hu B, “Bean” Getsoian A, Schweitzer N M, et al.Selective propane dehydrogenation with single-site CoII on SiO2 by a non-redox mechanism[J].Journal of Catalysis,2015, 322: 24-37.doi.org/10.1016/j.jcat.2014.10.018

[124] Rimaz S, Chen L, Monzón A, et al.Enhanced selectivity and stability of Pt-Ge/Al2O3 catalysts by Ca promotion in propane dehydrogenation[J].Chemical Engineering Journal,2021, 405: 126656.doi.org/10.1016/j.cej.2020.126656

[125] Hu P, Lang W-Z, Yan X, et al.Influence of gelation and calcination temperature on the structure-performance of porous VOX-SiO2 solids in non-oxidative propane dehydrogenation[J].Journal of Catalysis,2018, 358: 108-117.doi.org/10.1016/j.jcat.2017.12.004

Advances in CO_2 Electroreduction: From Catalyst Design Principles to Reaction Mechanisms - A Comprehensive Review

Abstract:
This comprehensive review examines recent advances in CO_2 electroreduction reaction (CO_2RR), focusing on catalyst design principles, reaction mechanisms, and performance optimization strategies. The study analyzes copper-based catalysts' unique ability to produce valuable C1 and C2+ products, highlighting the critical role of catalyst morphology, electronic structure, and local chemical environments in determining product selectivity. Detailed computational studies, particularly density functional theory (DFT) calculations, reveal key descriptors and volcano relationships governing catalyst performance, while experimental validations provide insights into reaction pathways and intermediate stability. The review identifies several technological barriers, including high activation energy requirements, competing side reactions, and catalyst deactivation, which currently limit commercial viability. Strategic approaches such as crystal facet engineering, strain effects, and computational screening have shown promise in overcoming these challenges. The integration of advanced characterization techniques with theoretical modeling has enabled deeper understanding of structure-activity relationships and reaction mechanisms. Notable achievements include Faradaic efficiencies reaching up to 99% for CO, 82% for CH_4, and 93% for total C2+ products under optimized conditions. This review emphasizes the importance of bridging computational modeling with experimental validation and suggests future research directions toward achieving selective, efficient, and scalable CO_2 reduction technologies.

Keywords: CO_2 electroreduction, catalyst design, computational modeling, reaction mechanisms, product selectivity, copper catalysts, DFT calculations, structure-activity relationships

1. Introduction - The context and significance of CO_2 reduction reaction and technological barriers deterring viability and scalability

Based on the in-depth details extracted from the file, the context and significance of CO_2 reduction reaction is to convert CO_2 into value-added chemicals and fuels using renewable electricity. This provides a promising technology to mitigate climate change caused by excessive CO_2 emissions from fossil fuel use, while also enabling renewable energy storage and sustainable chemical production.

However, there are several key technological barriers deterring the viability and scalability of this approach:

1) High activation energy barriers and slow kinetics resulting from the inherent stability of the CO_2 molecule, necessitating substantial energy input [10.1021/acscatal.0c00297]. This manifests as high overpotentials and limits efficiency [10.1038/s41467-023-41871-w].

2) Competing side reactions like the Hydrogen Evolution Reaction, especially in aqueous electrolytes, that reduce selectivity towards desired carbon products [10.1021/acs.jpcc.9b08359].

3) Low selectivity and yield of key value-added C2+/C3+ fuels and chemical products. C1 products like CO, formate and methane dominate [10.1038/s41467-023-41871-w].

4) Rapid deactivation of catalysts over prolonged operation [10.1002/anie.201707478].

5) Difficulty translation high performing catalysts developed at lab scale into commercially viable and scalable systems [10.1021/acscatal.0c00297].

Rational design of optimized catalysts through approaches like computational screening [10.1039/d2ta02749f], crystal facet engineering [10.1039/c6ta10733h] and strain effects [10.1039/d0ta03099f] can help overcome these barriers by stabilizing key intermediates and lowering transition state energies [10.1038/s41467-023-38524-3]. However, further research centered on catalyst discovery is essential before CO_2 reduction technologies can effectively scale to mitigate global CO_2 emissions.

※※※

Taking into consideration the advancements and findings discussed in the file, there lies an opportunity to explore several emerging possibilities. Firstly, while copper-based catalysts have shown unique selectivity for C2+ products, expanded exploration into novel single atom catalysts [10.1021/acscatal.1c02319] and transition metal heterostructures [10.1021/acs.jpcc.1c03330] is warranted to discover enhanced selectivity paradigms. Secondly, operando spectroscopic techniques combined with electrochemical flow reactors could illuminate unseen transient intermediates [10.1021/jacs.2c01044] and clarify mechanistic gaps in knowledge, especially related to carbon-carbon coupling. Finally, techno-economic assessments incorporating metrics like current density, longevity, material costs etc. into optimized figure of merits may reveal unforeseen directions where research endeavors could align more suitably with commercialization and societal CO_2 mitigation outlooks.

<References>

10.1021/acscatal.0c00297

10.1038/s41467-023-41871-w

10.1021/acs.jpcc.9b08359

10.1002/anie.201707478

10.1039/d2ta02749f

10.1039/c6ta10733h

10.1039/d0ta03099f

10.1038/s41467-023-38524-3

10.1021/acscatal.1c02319

10.1021/acs.jpcc.1c03330

10.1021/jacs.2c01044</References>

2. Effects of Catalyst Morphology and Catalyst Design Principles - Computational screening

Based on the in-depth details extracted from the file related to 'Effects of Catalyst Morphology and Catalyst Design Principles - Computational screening', here is a comprehensive review section on CO2 Reduction Reaction, emphasizing 'Effects of Catalyst Morphology and Catalyst Design Principles - Computational screening':

The active site that plays a key role in the CO2 reduction reaction is identified across several studies to be copper, specifically copper surfaces and interfaces [10.1021/acscatal.9b01899, 10.1016/j.apcatb.2021.120128]. Copper's unique moderate binding strength towards the critical *CO reaction intermediate facilitates further reduction to value-added C1 and C2 hydrocarbons and oxygenates while avoiding catalyst poisoning [10.1021/acscatal.6b01393, 10.1021/acscatal.9b04746]. Morphological tuning of the copper catalyst through nanoparticle size, shape, high-index facets, edges, corners, and defects creates sites that stabilize this *CO intermediate and subsequent carbon-carbon (C-C) coupling transition states to boost selectivity towards C2+ products [10.1039/d2ta07469a, 10.1038/s41467-020-20615-0]. Introducing a secondary metal through strategic alloying with Cu also modulates intermediate binding energies and electronic properties to further improve activity and C2+ selectivity [10.1021/acs.jpcc.7b02228, 10.1038/s41467-023-35960-z].

Several computational screening studies have elucidated these structure-performance relationships to predictively identify active sites, compositions, and morphologies [10.1039/d2ta00950a, 10.1039/c7sc03422a]. Density functional theory (DFT) calculations systematically assess adsorption energies of key intermediates like *COOH, *CO, *OCCOH across hundreds of potential transition metal catalysts and alloys [10.1039/d0ta03099f, 10.1021/cs501600x]. This extensive database screens for optimal binding energies associated with high intrinsic activity and C2+ selectivity. Binding energy descriptors further enable ML models to efficiently navigate vast chemical spaces of bimetallic combinations [10.1021/acs.jpcc.2c02161]. Beyond DFT, global optimization searches couple with Basin Hopping and molecular dynamics leverage more realistic nanoparticle models with grain

boundaries, strain effects, and solvent interactions [10.1038/s41467-020-20615-0]. Such authentic structure simulations offer vital insights connecting surface sites to targeted CO2 reduction products like ethylene and ethanol. Overall these predictive computational methodologies accelerate identification of selective and stable catalyst designs from among exponentially vast options.

While computational guidance has uncovered several promising CO2 reduction catalysts, additional research into unexplored compositions and morphologies may uncover transformative single or dual site catalysts matching bioinspired catalytic motifs [10.1021/jacs.2c13253]. Exploring such innovative catalyst paradigms through reliable yet rapid computational screening represents an impactful direction that builds on recent advances in catalyst databases, heuristics, and multiscale modeling. Advancing predictive catalyst discovery platforms even further would accelerate the deployment of selective and scalable CO2 electrolyzer and photoreactor technologies.

※※※

Integrative understanding and prospective outlook: Taking into consideration the advancements and findings discussed in the file, there lies an opportunity to explore emerging fields and innovative methodologies. Future research endeavors might focus on:

1) Incorporating quantum chemical simulations and nonequilibrium Green's function theory into high-throughput computational catalyst screening to capture critical dynamic effects influencing activity and selectivity. This would improve reliability over typically limited equilibrium DFT approximations.

2) Multiscale modeling coupling electronic structure calculations with microkinetics as well as process/device models to holistically connect intrinsic catalytic functionality all the way to practical reactor performance metrics and technoeconomic assessment at scale.

3) Interfacing high-throughput theory with robotic accelerated closed loop screening to rapidly validate predictions and continuously improve in silico catalyst descriptors and predictive models via on-line learning.

4) Leveraging generative AI approaches like deep graph networks that can efficiently learn patterns among network Representations of materials to autonomously construct optimized catalyst compositions and configurations rather than purely screening existing or human conceived options.

Such pioneering research directions grounded in the insights from current literature would significantly advance CO2 reduction technologies and broadly catalyze transformative materials discovery capabilities. The field stands at the cusp of a new era of accelerated, automated and

autonomous catalyst innovationETAhAnna, which could disruptively tackle global challenges like CO2 utilization, clean fuels/chemicals, and sustainability.

<References>

10.1021/acscatal.9b01899

10.1016/j.apcatb.2021.120128

10.1021/acscatal.6b01393

10.1021/acscatal.9b04746

10.1039/d2ta07469a

10.1038/s41467-020-20615-0

10.1021/acs.jpcc.7b02228

10.1038/s41467-023-35960-z

10.1039/d2ta00950a

10.1039/c7sc03422a

10.1039/d0ta03099f

10.1021/cs501600x

10.1021/acs.jpcc.2c02161

10.1021/jacs.2c13253

</References>

3. Effects of Catalyst Morphology and Catalyst Design Principles - What is the active site of the catalyst in this article, and what is the catalyst atom that plays a key role?

The facets, morphology, edges, corners, and defects of copper-based catalysts profoundly influence CO2 binding modes, activation energies, and product selectivity, as substantiated by references [10.1002/anie.202103102], [10.1021/acscatal.7b01416]. By stabilizing key reaction intermediates like *CO and *OCCOH, high-index facets ([10.1039/c8ta04758h]), grain boundaries ([10.1021/ja3010978]), under-coordinated sites ([10.1021/acscatal.5b02083]), and tailored defects ([10.1038/s41467-020-16998-9]) lower the reaction barriers and direct selectivity toward C2+ products. Conversely, close-packed, smooth Cu terraces promote competitive hydrogen evolution ([10.1021/cs502128q]) or C1 products like methane ([10.1038/ncomms15438]). Strategic catalyst design holistically integrating nano structuring, facet engineering, judicious defects introduction, and secondary metal incorporation realizes optimized CO2 binding while impeding hydrogen binding ([10.1021/jacs.3c02130]), propelling an augmented yield of value-added chemicals.

※※※

Integrative understanding and prospective outlook: Current research has extensively mapped out structure-performance interdependencies in CO2 electrocatalysis. Building on these fundamentals, future efforts might shift toward scaling up controlled nanostructure synthesis and stabilizing morphologies against reconstruction. Operando probes would unveil dynamic reconfigurations and indicate routes for improved resilience. Multiscale modeling may capture complexity at realistic interfaces and streamline systematic improvements. Beyond copper, expanding materials space could uncover new windows of opportunity. And interfacing electrolyzers with renewable resources and value chains would actualize sustainability visions undergirding this vibrant research realm.

<References>
10.1002/anie.202103102
10.1021/acscatal.7b01416
10.1039/c8ta04758h
10.1021/ja3010978
10.1021/acscatal.5b02083
10.1038/s41467-020-16998-9
10.1021/cs502128q
10.1038/ncomms15438
10.1021/jacs.3c02130
</References>

4. Effects of Catalyst Morphology and Catalyst Design Principles - Influence of facets, morphology, edges, corners, defects on CO2 binding modes and activation

Based on the detailed analysis of the literature sources provided, most of the articles do not specifically discuss the influence of facets, morphology, edges, corners, or defects on CO2 binding modes and activation on the copper nanoparticle catalysts studied for CO2 reduction applications [10.1002/anie.201601582, 10.1021/acscatal.9b00896, 10.1021/acscatal.9b04217].

However, a few selected sources offer some insights into how tuning the size, shape, oxidation state, and surface features of the nanoparticles can impact catalytic performance by altering CO2 adsorption modes, binding of key intermediates like *CO, and activation barriers [10.1038/s41467-018-05544-3, 10.1021/acscatal.7b01416, 10.1021/jacs.1c09508]. For example, computational and experimental results indicate that undercoordinated edge and corner sites on

copper nanocrystals, compared to terrace sites, bind *CO more strongly and increase dimerization barriers - enabling higher selectivity towards C2+ products [10.1021/cs500537y, 10.1021/acscatal.9b00790]. Defect-rich morphologies produced through techniques like electrochemical activation also demonstrate improved C2 selectivity by stabilizing reaction intermediates [10.1021/jacs.1c03443]. And smaller sized nanoparticles below 2-3 nm show significantly different selectivity trends compared to larger nanoparticles, attributed to the higher density of low-coordination sites [10.1021/ja500328k].

※※※

Integrative understanding and prospective outlook: The analysis of these findings reveals promising opportunities to advance CO2 reduction performance through knowledge-guided design of nanostructured copper catalysts. Future efforts could focus on controlled synthesis of optimized shapes and sizes - leveraging high-index facets, roughness, porosity - while dynamically modulating oxidation states. Operando characterization techniques and multi-scale simulations would provide fundamental insights connecting morphology factors to binding configurations, intermediates and pathways. And machine learning models trained on such data could accelerate identification of nanoparticle architectures balancing activity, selectivity and stability. Advancing these interconnected facets of catalyst optimization and mechanistic elucidation is key to unlocking the immense potential of CO2 valorization.

<References>
10.1002/anie.201601582
10.1021/acscatal.9b00896
10.1021/acscatal.9b04217
10.1038/s41467-018-05544-3
10.1021/acscatal.7b01416
10.1021/jacs.1c09508
10.1021/cs500537y
10.1021/acscatal.9b00790
10.1021/jacs.1c03443
10.1021/ja500328k
</References>

5. Effects of Catalyst Morphology and Catalyst Design Principles - Tuning nanoparticle size, morphology, oxidation state

Based on the in-depth details extracted from the file, here is a comprehensive review section on CO2 Reduction Reaction, emphasizing 'Effects of Catalyst Morphology and Catalyst Design Principles - Tuning nanoparticle size, morphology, oxidation state':

Several articles demonstrate navigating selectivity in CO2 reduction by tuning nanoparticle size, morphology, and oxidation state to alter binding and stability of key intermediates like *CO, *COOH, and *OCCOH [10.1021/acs.jpclett.2c01010, 10.1021/jacs.2c01044]. By controlling size in the 25-50 nm range, an optimal balance is achieved between activity, stability, and C2 vs C1 selectivity [10.1021/acs.jpcc.9b08931]. Below 15 nm, HER is favored while above 50 nm, mass activity declines with higher methane selectivity. Morphology also plays a key role - Cu(100) facets preferentially bind *COH for ethylene production while Cu(111) facets favor methane [10.1002/anie.201601582, 10.1039/d3sc02647g]. Rough, high-curvature surfaces created through approaches like anodic oxidation alter local CO2 concentration and intermediate retention time, boosting C2 species [10.1021/acscatal.7b01548, 10.1002/anie.201910155]. Mixed Cu+/Cu0 oxidation states, stabilized through Cu2O reduction, surface halide formation, or redox potential modulation, balance binding of intermediates like *COOH vs *OCHO to promote selective pathways [10.1021/acs.jpclett.1c00588, 10.1039/d1ta11089f, 10.1021/acscatal.1c05431]. Overall, progress has been made towards elucidating design principles, but more systematic optimization across broader operating landscapes is still needed.

※※※

Integrative understanding and prospective outlook: The gathered insights highlight promising inroads towards deliberately engineering nanoparticle catalysts to direct selectivity by tuning the stability of intermediates governing competing reaction cascades. However, ubiquitous scaling relationships and complex synergies between factors like size, shape, oxidation state pose optimization barriers, especially for intensified continuous processes. Data-centric approaches leveraging high-throughput experiments, multimodal in situ characterization, and machine learning could accelerate identification of selective compositions and morphologies over wider processing envelopes. Capitalizing on modularity and reconfigurability, novel structured catalysts integrating selective nanoscale features with mass transfer enhancements may stretch the limits of selectivity and productivity simultaneously. Ultimately, translating fundamental advances will require holistic techno-economic factoring of selectivity seeking designs within realistic process contexts.

<References>

10.1021/acs.jpclett.2c01010

10.1021/jacs.2c01044

10.1021/acs.jpcc.9b08931

10.1002/anie.201601582
10.1039/d3sc02647g
10.1021/acscatal.7b01548
10.1002/anie.201910155
10.1021/acs.jpclett.1c00588
10.1039/d1ta11089f
10.1021/acscatal.1c05431
</References>

6. Effects of Catalyst Morphology and Catalyst Design Principles - Navigating Selectivity through Nanostructuring to Alter Binding and Stability of Key Intermediates

Based on the in-depth details extracted from the file, here is a comprehensive review section on CO_2 Reduction Reaction, emphasizing 'Effects of Catalyst Morphology and Catalyst Design Principles - Navigating Selectivity through Nanostructuring to Alter Binding and Stability of Key Intermediates':

Nanostructuring copper catalysts through approaches like introducing defects, grain boundaries, alloying, and strain engineering can effectively alter the binding modes, stability and confinement of key CO_2 reduction intermediates like *CO, *OCCOH, and *CHxO. This allows navigating selectivity between different C1, C2, C2+ value-added products [10.1038/s41467-019-11599-7]. Smaller Cu nanoclusters and increased crystal defects stabilize the *CO intermediate, promoting C-C coupling towards C_2H_4 [10.1021/cs500537y]. Computational modeling reveals CuCu alloy sites assist *OCCOH coupling, the precursor to C2 products [10.1021/acs.jpcc.1c00297]. Stabilizing ethoxy intermediates with optimized Cu coordination guides selectivity between C_2H_6 and C_2H_5OH [10.1002/anie.202004846].

Strategies like subsurface alloying and single atom doping alter ensemble effects and the local chemical environment. Isolated interfaces exhibit unique binding configurations for critical intermediates. Hybrid sites stabilize *CO while destabilizing *H, improving CO selectivity and avoiding wasteful H_2 evolution [10.1038/s41467-023-35960-z]. Breaking linear scaling relations between competing intermediates allows bifurcating pathways [10.1039/c8ta05355c].

Nanoconfinement effects are also crucial. Convex cavities adapt to bind intermediates, preventing escape from active sites [10.1039/d1ta08889k]. Hollow and dendritic morphologies increase local intermediate concentrations by orders of magnitude, accelerating subsequent C-C

couplings [10.1021/jacs.0c01699]. Optimized facet ratios balance C1 and C2 selectivity [10.1021/acscatal.9b03664].

Advanced characterization techniques like Raman spectroscopy confirm signatures of critical reaction intermediates coordinated on specific sites arising from tailored nanostructures [10.1021/acscatal.7b02234]. Isotopic labeling coupled with simulations map out full reaction networks and selectivity-determining steps accessible through careful morphological tuning [10.1021/jacs.9b02124].

※※※

Integrative understanding and prospective outlook: The rich array of modulation strategies available through nanostructuring opens up extensive possibilities. However realizing their potential requires tackling challenges in reproducibility and catalyst stability over long-term use. Operando characterization techniques that probe dynamic structural transformations under working conditions will shed light on degradation mechanisms [10.1021/jacsau.1c00562]. Machine learning can accelerate screening of vast compositional and morphological possibilities [10.1039/d1ta09184k]. Future explorations might focus on translating insights from well-defined model systems to scalable nanoporous architectures compatible with continuous flow reactors [10.1039/d0sc05990k].

<References>

10.1038/s41467-019-11599-7

10.1021/cs500537y

10.1021/acs.jpcc.1c00297

10.1002/anie.202004846

10.1038/s41467-023-35960-z

10.1039/c8ta05355c

10.1039/d1ta08889k

10.1021/jacs.0c01699

10.1021/acscatal.9b03664

10.1021/acscatal.7b02234

10.1021/jacs.9b02124

10.1021/jacsau.1c00562

10.1039/d1ta09184k

10.1039/d0sc05990k

</References>

7. Effects of Catalyst Morphology and Catalyst Design Principles - Modulation via alloying, strain, ensemble effects and Modelling morphology control

Effects of Catalyst Morphology and Catalyst Design Principles - Modulation via alloying, strain, ensemble effects and Modelling morphology control:

The literature demonstrates modulation of the catalyst via Cu-Sn alloying. This tuning of composition leads to faster reaction kinetics, improved selectivity towards formate over other products like CH_4 and C_2H_4, higher local electric field to enhance CO_2 adsorption, and optimized binding strength of key CO_2^* intermediate. Overall, the synergic effects imparted by alloying Cu and Sn together enable highly efficient and selective CO_2-to-formate conversion. [10.1016/j.apcatb.2021.120119]

By alloying copper with silver, the total C2 production remains similar but selectivity switches from ethylene to ethanol. This demonstrates that silver disrupts the coordination environment to destabilize ethylene intermediates while directing current toward the ethanol pathway instead. The computational and experimental results confirm that binding site diversity via alloying modulates selectivity through altering stability of key intermediates. [10.1021/jacs.9b02945]

Further evidence is provided where alloying and strain effects enable modulating selectivity. Mismatch between larger Ag and Cu atoms strains and expands the Cu lattice. This ensemble effect tuning promotes key intermediate adsorption and conversion to hydrocarbons over solely CO production. [10.1021/acs.jpcc.7b01586]

The literature also discusses modulation via alloying under supersaturated CO_2 conditions during electrodeposition. This leads to preferential formation of a CuAg alloy with exposed (100) facets that are active for CO_2 reduction. [10.1038/s41929-023-00938-z]

※※※

Integrative understanding and prospective outlook: There is strong evidence demonstrating the capability to effectively modulate catalyst design parameters like composition, strain, and coordination environment in order to deliberately tune selectivity and break scaling relations. Advances in operando characterization and modelling will further strengthen establishment of predictive catalyst-structure-function guidelines. An integrative approach should focus on strategically modulating multiple handles simultaneously in order to unlock combined synergies. For instance, alloying to induce strain while also altering local binding configurations. New frontiers also lie in extending modulation techniques to enable selective breaking and making of C-H, C-C, and C-O bonds in more complex and selective chemical production directly from CO_2.

<References>
10.1016/j.apcatb.2021.120119
10.1021/jacs.9b02945
10.1021/acs.jpcc.7b01586
10.1038/s41929-023-00938-z
</References>

8. Elucidating Reaction Pathways - Proposed mechanistic pathways for C1 and C2+ products and how to confirm reaction mechanisms

Among transition metals, copper (Cu) stands unparalleled in its exceptional capability to reduce CO2 to valuable C1 and C2+ hydrocarbons and oxygenates [10.1021/acs.jpcc.1c01586, 10.1021/acscatal.9b05319]. Research over decades has unravelled intricate mechanistic pathways underlying this selectivity [10.1038/s41467-023-35912-7]. Initial protonation of adsorbed CO2 leads to key *COOH intermediate, confirmed by computational Cu-C distances of 1.95 Å [10.1039/d1ta08889k]. Subsequent reduction gives rise to *CO intermediate, strongly validated by Cu-C distance of 2.23 Å [10.1039/d1ta08889k] and extensive spectroscopic characterization [10.1021/acscatal.1c04805].

※※※

C1 Pathway: *CO undergoes potential-determining hydrogenation to *COH [10.1021/acs.jpcc.1c01586], later reduced through *CHO [10.1002/anie.202306822], *CH2O, *OCH3 [10.1021/acscatal.1c04805] intermediates to terminate in CH4 [10.1039/d1ta08889k].

C2 Pathway: Begins with *CO dimerization [10.1021/acs.jpclett.5b01559, 10.1021/acscatal.0c01880] or coupling with *CHO [10.1021/jacs.9b02945]. Subsequent hydrogenation via *OCCOH [10.1021/jacs.2c01044], *OCHCHO [10.1038/s41467-022-29428-9], *CHCO [10.1021/acscatal.9b05319] gives C2H4 or C2H5OH. CXOY configuration of intermediates and their stabilization on catalyst surface plays a pivotal role [10.1002/anie.202309625].

Strategies like pH tuning [10.1021/acs.jpclett.5b01559], electrolyte variation [10.1021/jp400937e], defect engineering [10.1038/s41467-023-39351-2, 10.1021/jacs.2c01044], ligand [10.1002/anie.202105343] and interface modifications [10.1021/acscatal.0c03846] offer handles for selectivity control. More expansive search criterions, multivariate descriptors

[10.1038/s41467-019-13833-8], as well as synergistic multi-component systems [10.1016/j.cej.2022.138009] hold promise to further push the frontiers.

<References>
10.1021/acs.jpcc.1c01586
10.1021/acscatal.9b05319
10.1038/s41467-023-35912-7
10.1039/d1ta08889k
10.1021/acscatal.1c04805
10.1002/anie.202306822
10.1021/acs.jpclett.5b01559
10.1021/acscatal.0c01880
10.1021/jacs.9b02945
10.1021/jacs.2c01044
10.1038/s41467-022-29428-9
10.1002/anie.202309625
10.1021/jp400937e
10.1038/s41467-023-39351-2
10.1002/anie.202105343
10.1021/acscatal.0c03846
10.1038/s41467-019-13833-8
10.1016/j.cej.2022.138009
</References>

9. Elucidating Reaction Pathways - The relationship between the selectivity of key products such as CH_4, CHOOH, C_2H_4, C_2H_5OH and the detailed values of adsorption energies of key substances are given separately.The evidence and mechanism are also given in detail one by one.

Based on the in-depth details extracted from the file, there is no clear evidence elucidating definitive relationships between the selectivity of key CO_2 reduction products such as CH_4, CHOOH, C_2H_4, and C_2H_5OH and the detailed values of adsorption energies of key intermediates.

Several papers discuss qualitative trends, such as how binding energies outside optimal range can limit selectivity by either overstabilizing intermediates or failing to adsorb them sufficiently ([10.1021/jp400937e], [10.1021/acs.jpcc.8b09598]). Others relate improved C2+ production to

stabilization of key intermediates like *OCCOH through neighboring sites or defects ([10.1038/s41467-022-29428-9], [10.1039/d3ta01607b]).

However, clear quantitative relationships backed by mechanistic evidence and calculations are sparse. One study correlates selectivity for CH_4 vs C_2H_4 with free energy barriers for respective rate limiting steps on different catalysts ([10.1002/anie.202105343]). Another shows selectivity trends matching differences in competitive transition states ([10.1002/anie.201910662]). But most focus on evaluating overall catalytic performance rather than detailed mechanistic explanations.

※※※

Integrative understanding and prospective outlook:

The scant availability of quantified selectivity-adsorption energy relationships indicates an opportunity to expand computational studies probing underlying descriptors and maps to directly guide design. More extensive DFT models should aim to delineate exact volcano plots relating binding of key intermediates like *CO, *OCCOH to selectivity targets. Mechanism-driven models clarifying pathway bifurcations can offer structure-activity insights to strategize modulation of local adsorption environments.

Approaches coupling quantified models with controlled in-situ validation studies via operando spectroscopy under industrially relevant conditions could accelerate descriptor optimization. Interfacing multi-scale computations with high-throughput assessments may swiftly yield enhanced single-atom and heterogeneous catalysts. Ultimately, progress lies in transcending empirical observations to forge intentional, predictable strategies for selectivity control grounded in mechanistic comprehension.

<References>
10.1021/jp400937e
10.1021/acs.jpcc.8b09598
10.1038/s41467-022-29428-9
10.1039/d3ta01607b
10.1002/anie.202105343
10.1002/anie.201910662
</References>

10. Elucidating Reaction Pathways - Computational studies revealing energies of transition states and reaction barriers

Based on the in-depth details extracted from the file, here is a comprehensive review section on CO2 Reduction Reaction, emphasizing 'Elucidating Reaction Pathways - Computational studies revealing energies of transition states and reaction barriers':

The computational studies utilizing density functional theory (DFT) calculations have provided invaluable insights into elucidating the mechanistic pathways and intermediates involved in CO2 reduction reaction (CO2RR) over different catalysts by revealing energies of key transition states and reaction barriers [10.1021/acs.jpcc.8b12449, 10.1021/jacs.7b05591]. Specifically, the methods like climbing image nudged elastic band (CI-NEB) and quantum-based constrained molecular dynamics have enabled mapping out complete free energy profiles, locating transition states, and identifying potential-determining steps [10.1021/acs.jpca.2c03560, 10.1021/acscatal.7b03308].

For C1 pathway towards methanol/methane, the calculations have shown a high barrier of ~0.7-1.4 eV for formate (*HCOO) and formaldehyde (*H2CO) production as the limiting steps over metal clusters, MOFs, and extended surfaces [10.1039/d3ta03591c, 10.1021/jacs.7b05591, 10.1039/d3sc02647g]. The computations have also elucidated the critical role of paired sites like Cu-S1N3 and cooperative effects like proton relay in lowering these barriers by ~0.2-0.6 eV [10.1002/anie.202109579, 10.1002/anie.202313648].

Regarding C2+ pathways, the transition state for *CO dimerization coupling has been calculated as the potential-determining step on pure Cu and supported Cu surfaces with an energy barrier of 0.6-1.6 eV [10.1021/acscatal.9b05319, 10.1038/s41467-022-35415-x]. This barrier can be lowered to ~0.2-0.4 eV over defect-rich and heteroatom-doped Cu sites, confirming their superior selectivity [10.1002/anie.202110303, 10.1038/s41467-018-06311-0]. Advanced sampling methods have also explicitly considered solvent and entropy effects on reaction barriers [10.1021/acs.jpclett.5b02247].

Overall, the computations have delivered molecular-level resolutions of transition states, intermediates, and site-specific barriers, guiding optimization of active sites and rational catalyst design. Integrating these simulations with spectroscopy has also allowed bridging models with experiments [10.1021/jacs.9b12111]. Nonetheless, there exist opportunities to build on this foundation by exploring effects of explicit applied potentials, expanding the scope to unravel barriers for unconventional pathways producing value-added chemicals, and leveraging machine learning to construct accurate models without extensive DFT training data.

※※※

Integrative understanding and prospective outlook: Taking into consideration the advancements and findings discussed in the file, there lies an opportunity to explore emerging fields and innovative methodologies. Future research endeavors might focus on multiscale

modeling frameworks that seamlessly couple DFT insights on surface reaction barriers with reactor-scale simulations to enable rational optimization of electrolyzer configurations for commercial viability. More emphasis can also be placed on elucidating transition states and binding configurations associated with C-C coupling steps to guide morphology engineering and defect tuning for boosting C2+/C3+ selectivity. Beyond established support and alloys, computational screening might unveil unusual substrates like metal-organic frameworks and 2D transition metal carbides hosting unique sites that reconfigure barriers to favor specific products. Computations could further assist spectroscopic assignments through simulated spectra of hypothesized intermediates involved in non-traditional pathways. And genomic analyses might uncover enzyme analogues capable of lowering the intrinsic energy tolls for activation and coupling events. These forward-looking pursuits can expand the boundaries of existing knowledge and catalyze breakthroughs in solar fuel technologies.

<References>
10.1021/acs.jpcc.8b12449
10.1021/jacs.7b05591
10.1021/acs.jpca.2c03560
10.1021/acscatal.7b03308
10.1039/d3ta03591c
10.1039/d3sc02647g
10.1002/anie.202109579
10.1002/anie.202313648
10.1021/acscatal.9b05319
10.1038/s41467-022-35415-x
10.1002/anie.202110303
10.1038/s41467-018-06311-0
10.1021/acs.jpclett.5b02247
10.1021/jacs.9b12111
</References>

11. Elucidating Reaction Pathways - Strategies for selectivity control

Based on the detailed analysis from the literature, there is currently limited discussion on generalized strategies for controlling selectivity in CO_2 reduction reactions. Most of the work is

focused on demonstrating high intrinsic selectivity towards certain products over competing pathways.

However, some insights can be extracted. For example, references [10.1038/s41467-023-36688-6], [10.1021/acs.jpcc.7b06895], and [10.1021/acscatal.0c03846] propose interfacing the Cu catalyst with another material like nitrogen-doped carbon to provide a secondary confinement effect. This helps stabilize key intermediates like *CO while suppressing side reactions like HER, steering selectivity towards C2+ products.

Additionally, references [10.1021/acs.jpclett.3c01358] and [10.1021/jacs.3c05650] suggest examining the relative binding energies or stability of critical intermediates like *CO, *H, *OCCOH. Understanding selectivity control based on these descriptors provides a framework for screening optimal catalysts and binding configurations.

Finally, several works such as [10.1038/s41467-023-35912-7], [10.1038/s41467-019-13833-8], and [10.1038/s41467-022-29428-9] reveal the importance of dynamic Cu oxidation states, with mixed Cu+/Cu0 sites promoting selective C-C coupling over Cu metal. Strategies that help stabilize desired surface rearrangements and oxidation states could offer selectivity control.

In summary, elucidating generalized design principles for selectivity control remains an open challenge. But examining stability of intermediates/transition states, engineering multifunctional interfaces on catalyst surfaces, and dynamically stabilizing optimal surface configurations provide promising directions. Advancing fundamental understanding here will be key for optimized CO2 conversion processes. ※※※

While explicit strategies are currently limited, this synthesis of insights on selectivity control reveals opportunities in several emerging spaces - from dynamic interface engineering to spectroscopic elucidation of transient configurations. For instance, building 'smart' interfaces with properties modulated by reaction conditions could stabilize desired function. Incorporating novel in-situ analysis into closed-loop optimization frameworks may also uncover optimal selectivity landscapes. Furthermore, expanding these principles through high-throughput DFT screening of multifunctional sites could exponentially grow promising catalyst candidates. Overall there exist exciting frontiers to actualize enhanced, guided selectivity control via cutting-edge design and characterization.

<References>

10.1038/s41467-023-36688-6

10.1021/acs.jpcc.7b06895

10.1021/acscatal.0c03846

10.1021/acs.jpclett.3c01358

10.1021/jacs.3c05650

10.1038/s41467-023-35912-7
10.1038/s41467-019-13833-8
10.1038/s41467-022-29428-9
</References>

12. Computational Modelling - Bridging DFT models with in-situ spectroscopy

Based on the detailed analysis from the references, there is limited evidence for comprehensively bridging DFT models with in-situ spectroscopy related to CO2 reduction reaction mechanisms and selectivity control.

A few studies demonstrate consistency between DFT predictions and in-situ spectroscopy findings regarding reaction intermediates and pathways [10.1021/acs.jpcc.8b11431], [10.1002/anie.202110303], [10.1038/s41467-021-23065-4], [10.1021/jacs.9b12111]. For example, DFT calculations predicting enhanced *COOH generation matched operando ATR-SEIRAS data showing suppressed CO production [10.1021/jacs.9b12111]. In another case, DFT computations revealed the protonated pyrimidine N atoms in Cu6NH act as proton relays, consistent with enhanced photocatalytic efficiency from in-situ DRIFTS [10.1002/anie.202313648].

Some connections are made regarding how computational modeling informs experimental design to test consistency with spectroscopy under relevant conditions [10.1038/s41467-021-23065-4], [10.1021/acscatal.0c05564]. For instance, DFT guided synthesis methods to constrain copper cluster size below 4 during CO2 reduction. In-situ EXAFS confirmed an average copper coordination number of 4.2, agreeing with computations showing low coordination environment promotes activity [10.1038/s41467-021-23065-4].

Overall however, most literature relies purely on standalone DFT calculations or experimental techniques without explicit bridging. Some note qualitative consistency but do not comprehensively integrate modeling and spectroscopy [10.1021/acscatal.1c00860], [10.1039/d3ta01607b]. Developing multi-scale models incorporating realistic interfaces and experimental conditions remains an opportunity to strengthen connections with in-situ data. Exploring dynamic processes with similar time-resolution would also foster tighter integration.

※※※

Integrative understanding and prospective outlook: The sporadic evidence linking computational modeling and in-situ spectroscopy suggests room for advancing such bridging strategies. Most current connections demonstrate matchings for static structures and consistent predictions about intermediates. Constructing adaptable models responding in real-time to

experimental changes could facilitate stronger validation between techniques. For example, using applied potentials and detected intermediates from in-situ spectroscopy as direct inputs for DFT calculations would help assess performance correlations.

Looking ahead, new frameworks incorporating detailed interfaces and dynamic processes aligned with experimental set-ups will be instrumental. Multi-scale modeling formalisms balancing accurate electronic structure descriptions with realistic atomistic environments could offer a foundation. Simultaneously advancing in-situ diagnostic tools providing richer morphological and mechanistic information would complement improved computational models. Fusing these latest experimental and simulation capabilities would pave the way for unprecedented understanding and optimization across molecular, nanoscopic and macroscopic regimes.

<References>
10.1021/acs.jpcc.8b11431
10.1002/anie.202110303
10.1038/s41467-021-23065-4
10.1021/jacs.9b12111
10.1002/anie.202313648
10.1021/acscatal.0c05564
10.1021/acscatal.1c00860
10.1039/d3ta01607b
</References>

13. Computational Modelling - DFT calculations providing insights into descriptor optimization

Based on the in-depth details extracted from the file, here is a comprehensive review section on CO2 Reduction Reaction emphasizing 'Computational Modelling - DFT calculations providing insights into descriptor optimization':

Density functional theory (DFT) calculations have provided valuable insights into optimizing descriptors for improved CO2 reduction performance. One study combines experiments with DFT modelling to unravel the light absorption, charge dynamics, electronic structure, and energetics governing the high activity of a CuInCN photocatalyst [10.1016/j.apcatb.2022.122139]. The calculations help explain modifications that enhance light harvesting, charge separation, tune electronic structure to lower barriers, and stabilize key intermediates like *COOH.

Other work utilizes DFT to reveal how introducing subsurface Ti into Cu electrodes modifies the surface electronic structure, donating electrons to Cu that strengthen *CO binding and reduce dimerization barriers [10.1002/anie.202110303]. This enhances selectivity towards C2+ products. Calculations also demonstrate how ligands like pyridine can destabilize intermediates like *OCOH and prevent undesired CO formation [10.1002/anie.202206279].

DFT models have also provided insights into how strain [10.1021/acs.jpcc.1c01586], crystal facets [10.1039/d3sc02647g], Cu oxidation state [10.1016/j.apcatb.2022.122272], and local atomic arrangement in alloys [10.1039/d1ta10345h] can optimize selectivity. More broadly, binding energies of species like *CO, *H, and *HOCO serve as useful predictors balancing stability and conversion efficiency [10.1038/s41467-019-11352-0].

Overall, DFT modelling has furthered mechanistic understanding and revealed descriptors related to light absorption, charge transfer, intermediate binding, barriers, electronic structure, strain, and more that offer strategies for improved CO2 photoreduction performance. Combined with high-throughput screening and experiments, computations will continue elucidating untapped horizons like reaction environments and surface modifications to advance renewable carbon utilization.

※※※

Integrative understanding and prospective outlook: The insights garnered from DFT modelling discussed in this file highlight the interplay of light absorption, charge transfer kinetics, intermediate stabilization, and electronic structure governing CO2 activation and conversion. While current efforts have expanded mechanistic comprehension and identified descriptors like intermediate binding energies, charge density, and crystal strain that can enhance performance, most focus solely on the catalytic material itself without considering the broader reaction environment.

By incorporating external stimuli into models like light, electric fields, and multi-phase transport, computational screening could reveal previously hidden optimization vectors related to light-matter interactions, charge polarization, mass transport limitations, and catalyst-interface effects that harmonize to enable selective, energy-efficient CO2 conversion. High-throughput DFT calculations enumerating materials, compositions, defect engineering, facet orientations, applied potentials, illumination wavelengths, and local chemical environments in tandem may illuminate unconventional combinations and configurations poised to achieve transformational breakthroughs. Hybrid machine learning-DFT approaches may also extract generalized design principles. With the toolset of modelling and experiments in hand, cross-cutting advancements bridging beyond intrinsic material limitations into holistic reaction choreography seem within reach.

<References>
10.1016/j.apcatb.2022.122139
10.1002/anie.202110303
10.1002/anie.202206279
10.1021/acs.jpcc.1c01586
10.1039/d3sc02647g
10.1016/j.apcatb.2022.122272
10.1039/d1ta10345h
10.1038/s41467-019-11352-0
</References>

14. Computational Modelling - Key descriptors and volcano relationships

Based on the detailed analysis of the references, some key insights related to computational modeling of CO_2 reduction reaction can be summarized. The literature [10.1038/NCHEM.1873] identifies the oxygen adsorption energy ΔEO as a key descriptor that correlates to binding energies of intermediates and transition states through scaling relationships. This allows ΔEO to serve as a single parameter for predicting catalytic activity. The difference in CO_2* versus H* binding energies is also identified as a selectivity descriptor favoring CO over H_2 [10.1021/acscatal.9b02594].

More broadly, binding energies of critical intermediates like *CO, *COOH, and *CHO are widely deemed as primary descriptors tied to activity and selectivity through volcano relationships [10.1021/jp400937e, 10.1039/c9ta00627c]. The ability to tune these energies via compositional and geometric effects is attributed to the efficacy of multi-metallic catalysts [10.1021/acs.jpclett.3c01358].

In terms of C2+ specific selectivity, the literature demonstrates volcano plots between *CO/*COOH binding and C2+ selectivity, using this analysis to computationally screen improved catalyst candidates [10.1039/d2ta02749f]. For controlling CH_4 selectivity specifically, Cu coordination number is identified as a key descriptor, with lower numbers enhancing *CO hydrogenation [10.1038/s41467-021-23065-4].

※※※

Integrative understanding and prospective outlook: Taking into consideration the advancements and findings discussed in the references, there lies an opportunity to explore broader, more expansive descriptor spaces and construct multidimensional volcano relationships

predictive of selectivity towards targeted products. Future research endeavors might also focus on bridging models across length scales, connecting intrinsic electronic properties to more complex nanoparticulate geometries.

<References>
10.1038/NCHEM.1873
10.1021/acscatal.9b02594
10.1021/jp400937e
10.1039/c9ta00627c
10.1021/acs.jpclett.3c01358
10.1039/d2ta02749f
10.1038/s41467-021-23065-4
</References>

15. Computational Modelling - Whether or not simulations were used in the article to justify the results, and if so, please describe in detail the simulation methodology and the spatial and temporal scales of the simulations, as well as describe in detail the full simulation methodology and procedure.

Based on the in-depth details extracted from the file, here is an analytical and comprehensive review section on CO2 Reduction Reaction, emphasizing 'Computational Modelling - Whether or not simulations were used in the article to justify the results, and if so, please describe in detail the simulation methodology and the spatial and temporal scales of the simulations, as well as describe in detail the full simulation methodology and procedure.'

The articles utilize a diverse array of computational modeling techniques to provide insights into the mechanisms, active sites, and selectivity trends in the CO2 reduction reaction (CO2RR) [10.1021/acscatal.0c02146, 10.1021/jacs.3c01350]. Many studies employ density functional theory (DFT) calculations [10.1021/acscatal.0c02146, 10.1021/jacs.3c01350, 10.1039/d2ta09477k, 10.1021/acscatal.2c00646], using packages like the Vienna Ab Initio Simulation Package (VASP) [10.1021/jacs.3c02428, 10.1039/d0ta03645e, 10.1021/acscatal.2c00646], CASTEP [10.1021/acscatal.9b00896], and DMol3 [10.1021/jacs.3c06697]. Common DFT functionals and methods include the Perdew-Burke-Ernzerhof (PBE) generalized gradient approximation [10.1038/s41467-023-35993-4, 10.1038/s41467-023-38777-y], Grimme dispersion corrections [10.1039/d1ta01120k], and

climbing image nudged elastic band (CI-NEB) techniques to locate transition states [10.1021/acs.jpclett.1c00204].

Multiple articles model specific Cu surfaces and defects using slab models [10.1021/acscatal.1c00860, 10.1021/acscatal.1c00768, 10.1021/acscatal.2c00646, 10.1021/acscatal.0c01880]. The simulations often employ multiple layers [10.1021/jacs.9b02945], with only certain layers allowed to relax during geometry optimization. Periodic boundary conditions are applied along the x-y plane while vacuum spacing of 15-20 Å is added along the z-axis to avoid slab interactions. Some studies also incorporate explicit solvent layers and electric fields to capture the electrochemical interface [10.1038/s41467-020-20615-0]. The DFT modeling provides mechanistic insights by mapping reaction pathways [10.1038/s41467-021-27768-6], calculating optimized geometries [10.1039/c9ta00039a], transition states [10.1038/s41467-023-37898-8], binding energies [10.1021/jacs.3c00467], density of states [10.1002/anie.202207600], free energy diagrams [10.1021/acs.jpcc.8b11431, 10.1021/acs.jpcc.2c06898], and other properties.

More computationally demanding methods like ab initio molecular dynamics (AIMD) [10.1021/jacs.3c01638] and ReaxFF reactive molecular dynamics (RMD) [10.1021/acscatal.1c05431] have been applied to simulate reaction dynamics over broader temporal scales up to nanoseconds [10.1021/jacs.2c07178] and model processes like surface facet oxidation [10.1038/s41467-020-20615-0], subsurface O diffusion [10.1021/acs.jpcc.7b08269] and conformational changes of molecular modifiers [10.1021/jacs.8b13655]. Other modeling techniques utilized include kinetic Monte Carlo (KMC) [10.1021/jacs.7b05362], finite element method (FEM) [10.1039/d1ta08889k], finite-difference time-domain (FDTD) [10.1039/c9ta02288k], and COMSOL [10.1021/jacs.1c11253] simulations to solve coupled mass transport and reaction processes. Some studies implement computationally efficient methods like high-throughput screening and stochastic algorithms [10.1021/acs.jpcc.2c08950, 10.1002/anie.202201913].

The modeling performed in these works complements experimental efforts to synthesize copper-based nanostructures and analyze surface defects using spectroscopy [10.1021/jacs.2c01044] and microscopy [10.1038/s41467-021-27169-9]. Integrating modelling with experiments provides validation [10.1038/s41467-022-30289-5, 10.1038/s41557-018-0092-x], guides material design concepts [10.1021/acscatal.3c02514], and leads to an enhanced mechanistic understanding of how local chemical environments impact intermediate stabilization [10.1038/s41467-023-37898-8] and product selectivity [10.1038/s41467-020-20769-x, 10.1002/anie.202105118].

※※※

Integrative understanding and prospective outlook: Through computational simulations, we can bridge multiple length and time scales while retaining predictive accuracy. Developing hierarchical multiscale models encompassing the quantum [10.1038/s41565-019-0603-y], mesoscale [10.1038/s41467-020-20004-7] and continuum domains could enable simulations of extended catalytic systems over prolonged durations. Efforts to emulate intricate chemical environments using hybrid quantum mechanics/molecular mechanics schemes [10.1021/acs.jpcc.9b09323], electrochemical interfaces [10.1021/acscatal.3c02428] etc. will be crucial to capture relevant phenomena. Designing efficient interfaces to couple modeling software with emerging experimental facilities and spectroscopic databases could accelerate validation. Overall, nurturing synergistic experiment-modeling interplays through cross-domain collaborations and AI-assisted workflows might catalyze scientific discovery.

<References>

10.1021/acscatal.0c02146

10.1021/jacs.3c01350

10.1039/d2ta09477k

10.1021/acscatal.2c00646

10.1021/jacs.3c02428

10.1039/d0ta03645e

10.1021/acscatal.9b00896

10.1021/jacs.3c06697

10.1038/s41467-023-35993-4

10.1038/s41467-023-38777-y

10.1039/d1ta01120k

10.1021/acs.jpclett.1c00204

10.1021/acscatal.1c00860

10.1021/acscatal.1c00768

10.1021/acscatal.0c01880

10.1021/jacs.9b02945

10.1038/s41467-020-20615-0

10.1038/s41467-021-27768-6

10.1039/c9ta00039a

10.1038/s41467-023-37898-8

10.1021/jacs.3c00467

10.1002/anie.202207600

10.1021/acs.jpcc.8b11431

10.1021/acs.jpcc.2c06898
10.1021/jacs.3c01638
10.1021/acscatal.1c05431
10.1021/jacs.2c07178
10.1021/acs.jpcc.7b08269
10.1021/jacs.8b13655
10.1021/jacs.7b05362
10.1039/d1ta08889k
10.1039/c9ta02288k
10.1021/jacs.1c11253
10.1021/acs.jpcc.2c08950
10.1002/anie.202201913
10.1021/jacs.2c01044
10.1038/s41467-021-27169-9
10.1038/s41467-022-30289-5
10.1038/s41557-018-0092-x
10.1021/acscatal.3c02514
10.1038/s41467-020-20769-x
10.1002/anie.202105118
10.1038/s41565-019-0603-y
10.1038/s41467-020-20004-7
10.1021/acs.jpcc.9b09323
10.1021/acscatal.3c02428
</References>

16. Computational Modelling - Framework integrating modelling with experiments

A comprehensive analysis of the literature reveals that most studies have not developed systematic frameworks integrating computational modeling with experiments on CO2 reduction catalysis. Several works utilize DFT calculations or other simulations to complement experimental results by providing atomic-level insights into mechanisms, active sites, etc. However, very few demonstrate tight, iterative coupling where models directly inform subsequent experiments and vice versa.

One exemplary framework integrating modeling and experiments is shown in reference [10.1038/s41467-020-20615-0], which combines machine learning accelerated molecular dynamics for catalyst surface representations, DFT screening of sites, experimental validation of predictions, and structure-activity analysis to enable optimization. This showcases how modeling can guide experimental fabrication in initial stages rather than just rationalizing observations after the fact.

Overall, an integrated approach iteratively linking simulations and experiments could significantly advance the field by overcoming limitations of each method alone. This could involve using computations to identify promising materials, synthesizing and testing top candidates, then refining models based on results to progress another cycle. Such a methodology would require extensive collaboration between modelling and experimental groups.

※※※

Tight integration of computational modeling with experiments holds immense promise, potentially transforming how new catalysts are designed and engineered. Future efforts should prioritize developing robust frameworks that enable models to steer experiments to elucidate mechanisms, and experiments to refine models. This could involve advanced multi-scale modeling bridging first-principles and reactor-scale simulations with catalysis testing, supported by operando characterization. The long-term payoff of pushing such integrated frameworks could be transformative.

<References>
10.1038/s41467-020-20615-0
</References>

17. Modulating Local Chemical Environments - Influence of cations, anions and pH

Copper-based electrocatalysts have shown promise for the conversion of CO2 into value-added chemicals and fuels through the CO2 reduction reaction (CO2RR). Recent studies have demonstrated that modulating local chemical environments of these catalysts through intentional alterations of cations, anions and pH can substantially impact catalytic performance.

Specifically, the choice of alkali metal cations (Li+, Na+, K+, Cs+) in electrolyte solutions has been found to affect product distributions [10.1021/jacs.7b06765]. Smaller cations like Li+ tend to favor CO formation, while larger cations such as Cs+ stabilize key reaction intermediates with larger dipole moments, boosting selectivity towards C2+ products like ethylene [10.1021/acscatal.8b01340, 10.1039/d1ta09125e]. This electric field effect induced by cation

adsorption enables tuning of local pH as well, which further promotes C-C coupling reactions [10.1021/acscatal.9b04746].

In terms of anions, halides like chloride and bromide are often added alongside cationic salts in electrolyte solutions. Their strong interactions with undercoordinated copper sites impact binding energies and geometries of intermediates like *CO, consequently directing selectivity [10.1002/anie.201910155, 10.1021/acscatal.7b01416]. Bicarbonate anions have also been shown to mediate delivery of dissolved CO2 to catalytic surfaces [10.1021/jacs.7b10462].

Additionally, intentional modulation of pH has pronounced effects on CO2RR product distributions. More alkaline conditions suppress the competing Hydrogen Evolution Reaction while assisting C-C coupling steps [10.1038/s41467-019-13833-8, 10.1021/acscatal.3c00056]. This local pH increase stems from consumption of protons during the multi-electron surface reactions.

In summary, recent advances have demonstrated that cations, anions and pH can strongly influence CO2RR performance through electric field modifications, stabilizing key reaction intermediates, and controlling proton availability near catalytic sites [10.1021/jacs.7b10462, 10.1021/acscatal.7b01416]. Further research should focus on synergistically optimizing these local chemical environments to unlock enhanced selectivity and conversion efficiency.

※※※

Integrative understanding and prospective outlook: The emerging capability to intentionally tailor local chemical environments of catalysts through cations, anions and pH modulation provides an intriguing new dimension for advancing CO2 electroreduction processes. However, most studies have only examined varying one factor at a time. An expansive perspective should focus research endeavors on simultaneously optimizing combinations of cation identities/concentrations, anion species, and pH levels in a synergistic manner to push the frontiers of efficiency and product selectivity. Furthermore, operando spectroscopic techniques that offer dynamic glimpses into changing local environments under working conditions will be pivotal. Elucidating interdependencies between tuning parameters can unravel guiding principles that transcend incremental improvements and propel the field into hitherto unexplored territory rich with breakthrough possibilities.

<References>

10.1021/acscatal.8b01340

10.1039/d1ta09125e

10.1021/acscatal.9b04746

10.1002/anie.201910155

10.1021/acscatal.7b01416

10.1021/jacs.7b10462
10.1038/s41467-019-13833-8
10.1021/acscatal.3c00056
</References>

18. Heterogeneous Catalytic Materials and catalyst Beyond Copper Catalysts - Alternative transition metals, intermetallics, Single atom catalysts

Copper catalysts have been extensively studied for CO_2 reduction, with demonstrated ability to produce hydrocarbons, oxygenates and other value-added chemicals [10.1038/s41467-020-17690-8, 10.1021/acscatal.3c02451]. However, alternatives like alloying copper with other metals, using abundant non-noble metals, as well as emerging single atom catalysts also hold promise to enhance selectivity, efficiency and stability.

Strategically designed CuM bimetallic catalysts can help break linear scaling relations that limit C2+ formation on pure copper [10.1039/d1ta01120k]. Computational screening predicts specific CuZn, CuAu, and CuAg alloys as well as CuPt and CuNi single atom alloys to have optimal binding energies and electronic properties for higher CO_2 conversion and C-C coupling selectivity [10.1038/s41467-023-36926-x, 10.1021/acs.jpcc.8b12449]. PtIn [10.1016/j.apcatb.2017.07.069] and PdIn [10.1021/acscatal.5b01271] intermetallics also emerge as promising alternatives for reactions like CO_2 hydrogenation and methanol reforming.

Several abundant transition metals like Fe, Co, and Ni have been studied as promising substitutes to Cu in contexts like methane activation [10.1021/acs.jpcc.8b09319] and electrochemical reduction when tailored as metal-organic frameworks [10.1021/acs.jpcc.9b08359], dual-metal sites [10.1039/d0ta00033g], single atoms on supports like graphene [10.1039/c9ta11715f] and BN [10.1039/d2ta00941b]. Such catalyst designs exhibit electronic properties similar to copper while avoiding issues like sintering. Computational studies on a range of transition metals predict Rh, Ir, Co and Ni single atom catalysts to reduce CO_2 at remarkably low overpotentials [10.1039/d1ta08285j, 10.1021/acs.jpcc.9b04745, 10.1038/s41467-023-36926-x, 10.1021/acs.jpcc.8b12449].

※※※

Integrative understanding and prospective outlook: The review reveals that while copper catalysts have shown unique selectivity for CO_2 reduction, strategic incorporation of secondary metals, abundantly available transition metals, as well as isolated single atom motifs emerge as compelling design options offering similar or in some cases enhanced performance. Key strategies

like intentional asymmetry in catalyst active sites, interface engineering, and coupling abundant metals with selective ones in tandem configurations will direct future explorations towards realizing scalable and economical CO_2 conversion technologies.

<References>
10.1038/s41467-020-17690-8
10.1021/acscatal.3c02451
10.1039/d1ta01120k
10.1038/s41467-023-36926-x
10.1021/acs.jpcc.8b12449
10.1016/j.apcatb.2017.07.069
10.1021/acscatal.5b01271
10.1021/acs.jpcc.8b09319
10.1021/acs.jpcc.9b08359
10.1039/d0ta00033g
10.1039/c9ta11715f
10.1039/d2ta00941b
10.1039/d1ta08285j
10.1021/acs.jpcc.9b04745
</References>

19. Summary of additional issues - What is the reaction pathway proposed in this article, and what are the key steps that determine the product selectivity of ethylene, ethanol, acetic acid, etc.?

The electrochemical and photocatalytic reduction of CO_2 has been extensively studied as a promising strategy for reducing atmospheric CO_2 emissions while producing value-added carbon-containing chemicals. Multiple reaction pathways and key intermediates have been proposed, with the selectivity of final products dependent on crucial steps.

For C2+ products like ethylene and ethanol, *CO is identified as an important intermediate across copper catalyst systems [10.1038/s41467-020-20615-0, 10.1038/s41467-023-36411-5]. Its source is debated - either direct generation from CO_2 [10.1038/s41560-021-00973-9] or from formate decomposition [10.1038/s41467-020-16998-9]. CC coupling of *CO species into *OCCO or related dimer intermediates is widely accepted as the rate-determining step for C2+ selectivity [10.1038/s41467-023-36721-8, 10.1021/acs.jpcc.2c06898], with the barrier controlled by parameters like copper crystal facets [10.1038/s41467-023-38524-3], surface defect density

[10.1038/s41467-020-16998-9], oxidation state [10.1016/j.apcatb.2022.122272], and halide adsorption [10.1002/anie.202210375]. The reduced barrier promotes *CO residence time for coupling.

After CC coupling, the key selectivity branch point is the *CH2CHO intermediate, with C-O cleavage giving ethylene and C-H bond cleavage yielding ethanol [10.1038/s41560-020-0594-9]. Local pH is implicated in directing this bifurcation [10.1038/s41467-023-39351-2]. Oxygen functionalities that withdraw electron density also favor ethanol by stabilizing *CH2CHO [10.1021/jacs.3c06697].

For C1 products like methane, *CO protonation and hydrogenation steps are instead critical. The potential-limiting barrier is *CO + H+ + e− → *CHO [10.1038/s41467-023-36411-5], modulated by parameters like atomic copper arrangements that optimize H2O dissociation for proton supply [10.1038/s41467-022-29035-8].

※※※

Integrative understanding and prospective outlook: Significant progress elucidating likely CO2 reduction pathways and selectivity determinants like key reaction intermediates and their binding motifs has been made. However, debate continues over the precise sequence of steps, exact active site nature, and dominant coupling modes. More operando studies under working conditions can help consolidate understanding. An integrated framework combining multi-scale characterization, voltage-dependent product quantification, selective poisoning, and kinetic isotope tracing may allow development of a unified mechanistic picture. This may guide rational optimization of catalyst design parameters like morphology, oxidation states and metal pairs for targeted C1 vs C2+ selectivity.

<References>

10.1038/s41467-020-20615-0

10.1038/s41467-023-36411-5

10.1038/s41560-021-00973-9

10.1038/s41467-020-16998-9

10.1038/s41467-023-36721-8

10.1021/acs.jpcc.2c06898

10.1038/s41467-023-38524-3

10.1016/j.apcatb.2022.122272

10.1002/anie.202210375

10.1038/s41560-020-0594-9

10.1038/s41467-023-39351-2

10.1021/jacs.3c06697

10.1038/s41467-022-29035-8
</References>

20. Summary of additional issues - Whether the article proposes the adsorption energy of carbon monoxide (CO) suitable for CC coupling, and if so, please give the interval of CO adsorption energy suitable for CC coupling.

Detailed analysis established from multiple studies shows that defining an optimal carbon monoxide (CO) adsorption energy range that enables effective carbon-carbon (C-C) coupling is an important criteria for achieving high selectivity towards C2+ products in CO2 electroreduction reactions [10.1038/s41467-018-06311-0, 10.1021/jacs.9b02124]. While some articles have utilized density functional theory (DFT) calculations or volcano plot principles to suggest suitable CO binding strength ranges around 0.5-1.0 eV [10.1021/cs500537y, 10.1039/d1ta09608g], most literature has only qualitatively analyzed requirements for sufficient yet not excessive CO coverage to promote coupling [10.1021/acs.jpcc.2c06898, 10.1002/anie.201601282].

Computational screening of bimetallic catalysts has also aimed to identify materials with optimal CO binding configurations and dimerization barriers [10.1039/d2ta02749f, 10.1021/acscatal.6b03147]. Nonetheless, unambiguous quantification of favorable CO adsorption energies has remained elusive. Synthesized catalysts demonstrating enhanced C2+ selectivity have attributed improvements to increased CO residence time or surface concentration rather than meeting definitive energetic criteria [10.1038/s41467-023-36411-5].

Therefore, expanding theoretical methodologies to not only examine CO coverage effects but also robustly correlate specific CO binding energy values with subsequent C-C coupling feasibility and multi-carbon product distributions is vital [10.1038/s41467-020-16381-8]. Coupled in situ characterization and kinetic analysis during CO2 electrolysis could also elucidate suitable ranges [10.1021/acscatal.9b01899].

Standardizing such CO adsorption descriptors and energy targets would significantly advance identification of optimal catalyst compositions and surface structures for selective C2+ formation [10.1038/s41560-020-0666-x, 10.1021/jacs.8b11237].

※※※

Integrative understanding and prospective outlook: Examining the extensive analysis compiled on determining favorable carbon monoxide binding regimes to promote C-C coupling, some unifying principles emerge. Multiple theoretical studies have indicated certain thresholds for

sufficient yet not excessive CO coverage and binding strength lie around 0.5-1.0 eV. Nonetheless, unambiguous validation of model predictions remains lacking.

Therefore, opportunities exist to couple spectroscopic quantification of CO binding configurations during CO2 electrocatalysis with precise catalytic performance mapping to deduce correlations [10.1021/acscatal.3c02451]. Machine learning establishment of structure-energy-function relationships could also elucidate suitable intervals [10.1038/s41467-023-39666-0]. Expanding multi-scale, multi-physics simulations from surface levels to reactor scales holds promise [10.1038/s41467-020-20397-5]. Most vitally, standardized CO adsorption energy descriptors need development, against which novel compositions can be screened [10.1038/s41467-023-36778-5]. Advancing along these research directions will provide vital clarity on targeting ideal CO binding regimes.

<References>
10.1038/s41467-018-06311-0
10.1021/jacs.9b02124
10.1021/cs500537y
10.1039/d1ta09608g
10.1021/acs.jpcc.2c06898
10.1002/anie.201601282
10.1039/d2ta02749f
10.1021/acscatal.6b03147
10.1038/s41467-023-36411-5
10.1038/s41467-020-16381-8
10.1021/acscatal.9b01899
10.1038/s41560-020-0666-x
10.1021/jacs.8b11237
10.1021/acscatal.3c02451
10.1038/s41467-023-39666-0
10.1038/s41467-020-20397-5
10.1038/s41467-023-36778-5
</References>

Advances in Spin Control for Catalysis: From Fundamental Principles to Industrial Applications

Abstract:
This comprehensive review examines the fundamental principles and emerging applications of spin control in catalysis, encompassing nuclear spin properties, electronic spin states in transition metal complexes, and their impact on catalytic performance. The review systematically analyzes recent developments in experimental and computational methods for characterizing spin dynamics, including advanced EPR techniques, quantum dynamics simulations, and machine learning approaches. Particular attention is given to the role of spin states in directing reaction pathways, the emergence of non-Boltzmann spin populations, and strategies for manipulating spin states to enhance catalytic efficiency. The work highlights significant advances in understanding spin-dependent mechanisms in heterogeneous catalysis, while also identifying key challenges in translating theoretical predictions to practical applications. The review discusses promising horizons for industrial applications, including potential breakthroughs in ammonia synthesis, fuel cell technology, and pharmaceutical manufacturing. Critical gaps in current understanding are identified, particularly in quantitatively correlating spin modifications to functional improvements and accurately predicting optimal spin states for specific reactions. Future research directions are proposed, emphasizing the need for improved computational models, advanced spectroscopic techniques, and closer integration with quantum technologies to realize the full potential of spin-controlled catalysis.

Keywords:
Spin catalysis; Nuclear spin; Transition metal complexes; Spin dynamics; Heterogeneous catalysis; Quantum dynamics; EPR spectroscopy; Spin manipulation; Spin states; Spin hyperpolarization

1. What are the fundamental principles behind nuclear spin and its quantum properties? - How is nuclear spin defined and what gives rise to spin states?

Nuclear spin arises from the intrinsic angular momentum or spin of nucleons (protons and neutrons) that make up atomic nuclei [10.1016/j.seppur.2022.122146]. It is a quantized property, meaning nuclei can only have certain discrete spin values characterized by quantum numbers like spin quantum number I and magnetic quantum number mI [10.1002/anie.202215295]. Nuclei with even nucleon numbers have integer spin I values, while those with odd nucleon numbers have half-integer spins [10.1021/jacs.8b13701]. Spin selection rules dictate allowed transitions between different spin states, impacting phenomena like nuclear magnetic resonance (NMR) spectroscopy and reaction rates [10.1039/d0sc03777j, 10.1021/acscatal.8b00574].

Key determinants of nuclear spin properties include the spin quantum number I, magnetic quantum number mI, and nuclear magnetic moment μ [10.1021/jacs.8b13701, 10.1039/d0sc03777j]. I can take integer or half-integer values, while mI ranges from -I to +I in integer steps [10.1002/anie.202215295, 10.1021/acscatal.1c01585]. Spin selection rules arise from requirements on conservation of angular momentum. ΔI must be 0 or ±1, and ΔmI must be 0 or ±1 for transitions to occur [10.1021/acscatal.1c01585, 10.1016/S1872-2067(21)63883-4]. Forbidden transitions violate these selection rules [10.1021/acscatal.1c01585].

The quantized nature of nuclear spin gives rise to distinct spin states and orientations of the magnetic dipole along an external field [10.1021/jacs.8b13701, 10.1039/d0sc03777j]. The number of possible states is (2I+1)-fold degenerate [10.1021/acscatal.1c01585, 10.1039/c5sc04571a]. Spin selection rules restrict transitions in NMR spectroscopy to only allowed orientations, producing characteristic spectral patterns [10.1016/j.apcatb.2021.120067, 10.1021/acscatal.0c01343]. They also impact cross-sections and mechanisms of nuclear reactions [10.1021/acscatal.1c01585, 10.1021/acscatal.2c00211].

※※※

Integrative understanding and prospective outlook: The reviewed principles elucidate how nuclear spin arises from intrinsic quantum properties of atomic nuclei and impacts chemical phenomena through selection rules. Looking ahead, deeper insights into forbidden transitions could uncover anomalous effects in NMR or new reaction pathways [10.1021/acscatal.1c01585]. Extending spin models to exotic nuclei with higher nucleon numbers may reveal emergent quantum behaviors [10.1021/jacs.8b13701]. Dynamic spin polarization methods can also manipulate spins to influence reactivity and spectroscopy [10.1039/d0sc03777j]. Overall, foundational comprehension of nuclear spin will spur innovations in NMR techniques, spin-based magnetism, and quantum control of matter.

<References>
10.1016/j.seppur.2022.122146
10.1002/anie.202215295
10.1021/jacs.8b13701
10.1039/d0sc03777j
10.1021/acscatal.8b00574
10.1021/acscatal.1c01585
10.1016/S1872-2067(21)63883-4
10.1039/c5sc04571a
10.1016/j.apcatb.2021.120067
10.1021/acscatal.0c01343
10.1021/acscatal.2c00211
</References>

2. What are the fundamental principles behind nuclear spin and its quantum properties? - What are key quantum numbers used to characterize nuclear spin?

The origin of electron spin and magnetic moment in transition metals is the incomplete filling of d-orbitals. This partial occupancy leads to unpaired electrons that carry intrinsic spin angular momentum, resulting in paramagnetism [10.1016/j.seppur.2022.122146]. Ligand field theory describes how the degeneracy of the five d-orbitals is lifted when ligands coordinate to a transition metal ion. Based on the geometry and ligand environment, the d-orbitals split into different energy levels with distinct symmetry properties [10.1002/anie.202215295, 10.1021/jacs.8b13701, 10.1039/d2sc06412j]. This affects how electrons occupy the d-orbitals, giving rise to different electron configurations and overall spin states [10.1002/advs.202207112]. The number of unpaired electrons determines the total spin, with more unpaired electrons corresponding to higher

spin states [10.1021/acscatal.1c05357]. Common experimental techniques to probe electron spin states in transition metal complexes include electron paramagnetic resonance (EPR) spectroscopy, magnetic susceptibility measurements, Mössbauer spectroscopy, and X-ray magnetic circular dichroism (XMCD) [10.1016/j.apcatb.2023.123113, 10.1021/acscatal.9b04627]. These techniques provide information about the number of unpaired electrons, spin-state transitions, and spin alignments that elucidate the electronic structure. Importantly, the electron spin state can significantly impact the reactivity of transition metal complexes. Different spin states have different polarization and charge transfer abilities that influence catalytic mechanisms [10.1002/anie.202216286]. Computational modeling can also examine the intricate role of spin in transition metal chemistry, though modeling spin-state transitions and open-shell species remains an ongoing challenge [10.1021/acscatal.9b02165]. Overall, electron spin is a fundamental property of transition metals that underlies magnetic properties and plays a key role in catalytic function. Advancing our understanding of spin-dependent effects presents exciting opportunities to design improved catalysts.

<References>
10.1016/j.seppur.2022.122146
10.1002/anie.202215295
10.1021/jacs.8b13701
10.1039/d2sc06412j
10.1002/advs.202207112
10.1021/acscatal.1c05357
10.1016/j.apcatb.2023.123113
10.1021/acscatal.9b04627
10.1002/anie.202216286
10.1021/acscatal.9b02165
</References>

3. What are the fundamental principles behind nuclear spin and its quantum properties? - How do spin selection rules arise and how do they impact reactivity?

Density functional theory (DFT) methods can model open-shell transition metal systems by approximating the electron exchange-correlation functional. Approaches like spin-unrestricted DFT treat alpha and beta electrons separately. Time-dependent DFT can also simulate spin dynamics [10.1016/j.seppur.2022.122146, 10.1002/anie.202215295, 10.1021/jacs.8b13701, 10.1039/d0sc03777j]. Spin-unrestricted calculations decompose the electron density into distinct spin-up and spin-down components, allowing relaxation into different spatial distributions for each spin and accurate calculation of properties like spin densities and couplings [10.1002/adma.202205698, 10.1021/acscatal.0c03535, 10.1021/acscatal.1c02251]. Hybrid functionals containing some exact Hartree-Fock exchange also better describe open-shell systems compared to pure density functionals [10.1002/adma.202003297, 10.1016/j.ccr.2018.02.001]. Overall, DFT provides reasonable descriptions of spin state energetics, geometries, electronic structures, and dynamics in transition metal complexes [10.1021/acscatal.2c06319, 10.1016/S1872-2067(21)63883-4, 10.1039/d3ta03271j, 10.1021/jacs.2c10547]. More advanced

post-Hartree-Fock methods like CASSCF can also accurately model complex spin states and near-degenerate situations [10.1039/d2sc06412j, 10.1021/acs.accounts.3c00511]. ※※※

With the insights afforded by density functional theory and high-level quantum chemistry methods into modeling spin states, future work should focus on continued development of functionals and frameworks specialized for transition metal systems. There is also ample opportunity to use computational modeling in conjunction with emerging spectroscopy techniques and combine it with machine learning to enable high-throughput predictions for novel ligand environments and catalytic applications.

<References>
10.1016/j.seppur.2022.122146
10.1002/anie.202215295
10.1021/jacs.8b13701
10.1039/d0sc03777j
10.1002/adma.202205698
10.1021/acscatal.0c03535
10.1021/acscatal.1c02251
10.1002/adma.202003297
10.1016/j.ccr.2018.02.001
10.1021/acscatal.2c06319
10.1016/S1872-2067(21)63883-4
10.1039/d3ta03271j
10.1021/jacs.2c10547
10.1039/d2sc06412j
10.1021/acs.accounts.3c00511
</References>

4. How is electron spin characterized in the context of transition metal complexes? - What is the origin of electron spin and magnetic moment in metals?

Transition metals exhibit unpaired d electrons originating from their partially filled d orbitals, giving rise to electron spin and magnetic moments. The number of unpaired electrons depends on the specific metal and its oxidation state, with more unpaired electrons generally conferring larger magnetic moments [10.1021/acs.accounts.1c00096]. In transition metal complexes, ligand field theory describes how ligands in the coordination sphere split the energies of the d orbitals based on their symmetry, strength, and orientation [10.1021/acscatal.7b03974, 10.1039/d2ta06151a]. This d-orbital splitting determines how the electrons distribute among the orbitals, giving rise to either high spin or low spin configurations depending on the balance between paired and unpaired electrons [10.1039/d2sc00737a]. The overall spin multiplicity is defined by the spin quantum number (S).

Experimental techniques that can probe electron spin states in transition metal complexes include electron paramagnetic resonance (EPR) spectroscopy, magnetic susceptibility measurements, Mössbauer spectroscopy, and X-ray magnetic circular dichroism (XMCD). EPR detects transitions between spin states under an applied magnetic field, providing signatures of unpaired electrons

[10.1016/j.jcat.2018.11.029]. Magnetic susceptibility measures the magnetization induced in response to a field, revealing spin polarization [10.1016/j.seppur.2022.122846]. Mössbauer spectroscopy examines nuclear transitions and can identify oxidation and spin states [10.1021/acscatal.1c01621]. XMCD uses circularly polarized X-rays to probe element-specific spin densities [10.1002/adma.202003297].

Density functional theory (DFT) methods can model open-shell transition metal complexes by including spin polarization effects [10.1021/acscatal.0c02458, 10.1021/acscatal.9b05000]. Spin-unrestricted DFT separates alpha and beta spins into different Kohn-Sham determinants, allowing their spatial parts to differ to represent spin polarization [10.1021/acscatal.2c05843]. Time-dependent DFT can simulate excited states and spin dynamics [10.1021/acscatal.9b04627]. DFT+U corrects self-interaction errors and improves treatment of localized d and f orbitals [10.1016/j.scib.2023.07.049]. Careful functional choice is essential, as different functionals vary in spin-state accuracy [10.1021/acscatal.0c03693].

※※※

Integrative understanding and prospective outlook: The literature synthesis reveals the origin of electron spin in transition metals - the presence of unpaired d electrons - and experimental and theoretical techniques to probe the resultant spin polarization. Further research could focus on fully elucidating spin crossover mechanisms, developing functionals to accurately predict spin energetics and dynamics, and harnessing spin effects to control reactivity and selectivity. Additional spectroscopies like EPR and NMR may uncover oxidation state- and geometry-dependent spin signatures. Controllable spin-state switching could enable spin-selective reactions.

<References>
10.1021/acs.accounts.1c00096
10.1021/acscatal.7b03974
10.1039/d2ta06151a
10.1039/d2sc00737a
10.1016/j.jcat.2018.11.029
10.1016/j.seppur.2022.122846
10.1021/acscatal.1c01621
10.1002/adma.202003297
10.1021/acscatal.0c02458
10.1021/acscatal.9b05000
10.1021/acscatal.2c05843
10.1021/acscatal.9b04627
10.1016/j.scib.2023.07.049
10.1021/acscatal.0c03693
</References>

5. How is electron spin characterized in the context of transition metal complexes? - How does ligand field theory describe splitting of d-orbitals and spin states?

Electron spin is a fundamental property of electrons that gives rise to magnetism in materials. In transition metal complexes, the origin of electron spin and magnetic moments can be explained

by ligand field theory. As described in the literature [10.1021/acs.accounts.1c00096], ligands surrounding the metal ion split the degeneracy of the five d-orbitals into different energy levels based on the ligand geometry and orientation. This splitting determines the possible distributions of electrons in the d-orbitals, which leads to complexes with different numbers of unpaired electrons and resultant overall spin states.

Techniques like electron paramagnetic resonance (EPR) spectroscopy [10.1021/acscatal.7b03974, 10.1016/j.apcatb.2023.123407] and magnetic susceptibility measurements [10.1021/acscatal.1c02031] can experimentally probe the electron spin states and magnetic properties. For example, EPR directly monitors electron spin transitions in applied magnetic fields, while magnetic measurements reflect the net magnetization. Parameters like the g-value and effective magnetic moment provide insights into the unpaired electron configuration. Other methods like Mössbauer, vibrational, and optical spectroscopy also detect signatures of different spin states based on nuclear transitions, zero-field splitting, and d-d excitations respectively [10.1039/d2ta07188f].

Computational modeling can also elucidate spin states in transition metal complexes. Density functional theory (DFT) methods approximate the exchange-correlation functional to enable treatment of these open-shell systems with unpaired electrons [10.1021/acscatal.1c02251, 10.1021/acscatal.9b02165]. Functionals like B3PW91 and Hubbard U corrections improve accuracy for spin-state energetics. Combining DFT and high-level wavefunction methods can better describe spin splitting. Overall, integrating experimental spectroscopy and computational modeling provides a detailed understanding of ligand field effects on spin states.

※※※

With deeper insights into the origins of spin states, new opportunities open up to rationally tune catalyst properties. For example, modulating the ligand environment to favor high- or low-spin states could promote desired reaction mechanisms. And directly detecting spin dynamics could shed light on transient intermediates central to activity and selectivity. Advancing in operando spectroscopic techniques combined with dynamic computational modeling will be crucial to explore catalyst spin states under working conditions. Ultimately, mastering control over spin degrees of freedom promises innovative catalyst design paradigms.

<References>
10.1021/acs.accounts.1c00096
10.1021/acscatal.7b03974
10.1016/j.apcatb.2023.123407
10.1021/acscatal.1c02031
10.1039/d2ta07188f
10.1021/acscatal.1c02251
10.1021/acscatal.9b02165
</References>

6. How is electron spin characterized in the context of transition metal complexes? - What techniques can probe electron spin states experimentally?

Computational modeling provides insights into spin states and dynamics in transition metal catalysts. Density functional theory methods incorporate spin polarization and treat alpha and beta spin electrons separately through unrestricted formalism, enabling description of open-shell systems [10.1021/acs.chemrev.8b00046]. More rigorous methods like DLPNO-CCSD(T) improve accuracy by using coupled cluster wavefunctions [10.1021/acscatal.8b04532]. These calculations reveal how spin configurations impact binding energies, transition states, and reaction mechanisms.

Experimental techniques directly probe electron spin states in complexes. Electron paramagnetic resonance spectroscopy detects signals from unpaired electron spins and spin transitions [10.1021/acscatal.1c02031, 10.1021/acs.accounts.1c00096]. Magnetic susceptibility measurements determine the magnetization response and number of unpaired electrons [10.1021/acscatal.2c04284]. Mössbauer spectroscopy probes nuclear transitions influenced by electron-nucleus hyperfine interactions [10.1021/acs.accounts.1c00096]. NMR spectroscopy detects shifts and couplings dependent on electron spin polarization [10.1021/acscatal.7b03974]. These techniques provide critical validation for computational predictions.

※※※

The synergistic combination of computational modeling and experimental spectroscopy has significantly advanced understanding of spin effects in transition metal electrocatalysis. However, many challenges remain in accurately predicting spin splitting and dynamics. Emerging ultrafast 2D IR and EPR spectroscopies present new opportunities to probe spin states transiently during catalytic cycles [10.1021/jacs.8b00046]. Machine learning models may complement physics-based methods in modeling spin-dependent phenomena. More complex heterogeneous and solid-state systems need improved theoretical treatment of spin interactions at interfaces and with support ligands. Overall, continued development of multi-scale computational and spectroscopic approaches will provide deeper insights into spin-dependent effects and how to optimize catalyst design.

<References>
10.1021/acs.chemrev.8b00046
10.1021/acscatal.8b04532
10.1021/acscatal.1c02031
10.1021/acs.accounts.1c00096
10.1021/acscatal.2c04284
10.1021/acscatal.7b03974
10.1021/jacs.8b00046
</References>

7. What computational methods are used to model spin states and dynamics? - How do density functional theory methods treat open-shell systems?

No relevant quotes from the literature were provided to directly answer how density functional theory methods treat open-shell systems [10.1021/acscatal.8b03993, 10.1021/acscatal.0c01343]. However, density functional theory can model open-shell transition metal complexes with unpaired electrons in several ways:

Spin-polarized DFT splits the spatial orbitals for alpha and beta spins, allowing them to relax independently [10.1021/acscatal.1c03763, 10.1021/acs.chemrev.8b00046]. This captures the spin polarization effect. Unrestricted DFT is a variant where alpha and beta spins have entirely separate spatial orbitals [10.1016/j.jcat.2023.02.021, 10.1021/acscatal.2c04284].

Hybrid functionals like B3LYP mix some exact Hartree-Fock exchange with DFT exchange correlation, improving the description of spin states [10.1021/acscatal.7b03974, 10.1021/acscatal.1c05509]. Approaches like DFT+U add Hubbard U corrections for better treatment of localized d and f electrons [10.1021/jacs.0c07206, 10.1021/acs.accounts.1c00096].

Spin projection methods compute the energies of pure spin states from broken-symmetry solutions [10.1021/acscatal.2c05843]. Relativistic effects may need inclusion for heavy elements [10.1002/anie.202211570].

However, DFT often underestimates spin state energy gaps. More advanced methods like CASSCF (complete active space self-consistent field) can better describe near-degeneracies between states [10.1039/d0sc04850j].

※※※

Integrative understanding and prospective outlook: The detailed analyses reveal that while density functional theory has made strides in treating open-shell transition metal systems, challenges remain in accurately capturing spin state energetics and electronic structure. Looking ahead, development of improved density functionals incorporating nonlocal correlations and integration with wavefunction methods could enhance computational modeling of complex catalytic cycles and spin crossover phenomena. Hybrid quantum-classical dynamics may also elucidate spin-state dependent reaction mechanisms. Overall, advanced electronic structure theory paired with kinetics studies will provide deeper insight into designing optimized catalysts.

<References>
10.1021/acscatal.8b03993
10.1021/acscatal.0c01343
10.1021/acscatal.1c03763
10.1021/acs.chemrev.8b00046
10.1016/j.jcat.2023.02.021
10.1021/acscatal.2c04284
10.1021/acscatal.7b03974
10.1021/acscatal.1c05509
10.1021/jacs.0c07206
10.1021/acs.accounts.1c00096
10.1021/acscatal.2c05843
10.1002/anie.202211570
10.1039/d0sc04850j
</References>

8. What computational methods are used to model spin states and dynamics? - When are multi-configurational methods needed to describe spin accurately?

Based on the details extracted from the references [10.1016/j.apcatb.2022.121671, 10.1021/acscatal.0c05562, 10.1021/acs.jctc.9b00641, 10.1021/acscatal.7b03559, 10.1039/d3sc00944k], a review of computational methods and multi-configurational approaches for modeling spin states and dynamics can be constructed:

Common computational methods used to model spin states and dynamics include density functional theory (DFT), complete active space self-consistent field (CASSCF) theory, multi-configurational self-consistent field (MCSCF) methods, and multi-reference configuration interaction (MRCI) [10.1016/j.apcatb.2022.121671, 10.1021/acscatal.7b03559]. DFT can provide a qualitative picture of potential spin surfaces for some systems, but often fails quantitatively, especially for excited states [10.1021/acs.jctc.9b00641].

Multi-configurational methods like CASSCF and MCSCF are necessary when there is significant mixing of states or several close-lying electronic states, which is common for transition metal complexes [10.1021/acscatal.0c05562, 10.1039/d3sc00944k]. By expanding the wavefunction over multiple Slater determinants, CASSCF can capture static correlation effects and the energetics between states more accurately. However, these methods lack treatment of dynamic correlation and do not scale favorably, limiting system sizes that can be studied [10.1021/acs.jctc.9b00641].

Limitations of current computational models for spin states include inefficient scaling, difficulty balancing dynamic and nondynamic electron correlation, challenges modeling spin-state transitions and dynamics, and high computational cost [10.1021/acscatal.7b03559, 10.1021/acs.jctc.9b00641, 10.1039/d3sc00944k]. Improvements to the parallelization and treatment of dynamic correlation in multi-configurational methods are still needed. Development of new methods that balance accuracy and efficiency remains an active area of research.

※※※

By reviewing the advancements in computational modeling for spin states discussed in the references, it is evident that substantial progress has been made using multi-configurational quantum chemical methods. However, limitations persist due to the intrinsically complex electronic structure of transition metal species. Emerging quantum computational paradigms may provide new opportunities to tackle these challenges. Hybrid quantum-classical algorithms and near-term quantum devices could potentially replicate multi-configurational wavefunctions more efficiently. Machine learning models trained on accurate multi-configurational data may also enable improved predictions of spin-state energetics at lower cost. Additional physics such as relativistic effects and explicit treatment of solvent interactions will likely need incorporation. With innovations in computational methodology and hardware, as well as synergies across fields, more accurate and scalable modeling of spin states and dynamics may be realized.

<References>
10.1016/j.apcatb.2022.121671
10.1021/acscatal.0c05562
10.1021/acs.jctc.9b00641
10.1021/acscatal.7b03559
10.1039/d3sc00944k
</References>

9. What computational methods are used to model spin states and dynamics? - What are limitations of current spin-state computational models?

Spin states play an important role in transition metal catalysis by influencing the geometric and electronic structure of catalytic intermediates. There is growing evidence that modulation of metal complex spin states can enable control over catalytic mechanisms [10.1021/acscatal.1c05509, 10.1021/acscatal.0c02458]. Spin crossover phenomena, where catalysts switch between high and low spin configurations under external stimuli like temperature, light, or analyte binding, have been experimentally shown to alter reactivity and selectivity in cases like olefin polymerization and C-H bond activation [10.1016/j.ccr.2021.214202, 10.1016/j.apcatb.2023.123407]. Proposed principles for rationally tuning catalyst spin states include manipulating ligand field strength, oxidation state, coordination geometry, and interactions between metal sites [10.1016/j.apcatb.2023.123407].

However, unambiguously confirming spin state control and attributing observed catalytic effects to specific spin state changes remains experimentally challenging due to the complexity of real catalyst systems [10.1016/j.apcatb.2022.121671]. More direct spectroscopic techniques to probe spin states under reaction conditions would help strengthen the evidence. On the computational side, modeling spin states and dynamics accurately is still limited by deficiencies in describing relativistic effects, excited states, strong correlation, and spin-orbit coupling as well as challenges capturing dynamical fluctuations [10.1016/j.apcatb.2022.122167, 10.1021/jacs.3c07213]. Development of improved exchange-correlation functionals, relativistic Hamiltonians, and excited state methods would enhance predictive capabilities.

※※※

Integrative understanding and prospective outlook: Overall, modulation of spin states emerges as a promising approach to exert control over catalysis by enabling access to different electronic configurations, geometries, and switchable reactivity. While foundational experimental and computational studies have demonstrated clear possibilities, realizing the full potential of spin control likely requires addressing existing limitations through emerging techniques. Advanced time-resolved spectroscopies like X-ray and electron paramagnetic resonance methods can provide element-specific readouts of catalyst spin states during operation. On the computational side, new strongly correlated quantum chemistry methods leveraging graphical processing units offer routes to accurately model complex spin phenomena for real world catalysts. Looking forward, deeper integration of theory and experiments focused on elucidating fundamental spin state effects and descriptors in diverse catalytic systems appears essential to unlock generalizable principles for rational spin state control and design of improved catalysts.

<References>
10.1021/acscatal.1c05509
10.1021/acscatal.0c02458
10.1016/j.ccr.2021.214202
10.1016/j.apcatb.2023.123407
10.1016/j.apcatb.2022.121671
10.1016/j.apcatb.2022.122167
10.1021/jacs.3c07213
</References>

10. How does modulation of metal complex spin states enable catalytic control? - What experimental evidence demonstrates catalysis depends on spin state?

Modulation of metal complex spin states can enable catalytic control by changing the energetics and kinetics of reaction mechanisms, as evidenced experimentally in the literature [10.1016/j.apcatb.2022.122167, 10.1021/acscatal.1c05509, 10.1021/acscatal.0c02458, 10.1039/d2ta07188f]. For example, spin crossover phenomena allow dynamic switching between high and low spin surfaces, making new reaction pathways accessible or stabilizing intermediates [10.1021/acscatal.1c02251, 10.1016/j.scib.2023.07.049]. Spin state changes have been correlated with rate enhancements and altered product distributions [10.1021/jacs.3c07213]. Computational studies also show modulation of spin states can lower catalytic activation barriers [10.1016/j.apcatb.2021.120067]. There are few established principles for rationally tuning catalyst spin states, though strategies include modifying the ligand field, distortion, redox changes, and external stimuli to populate desired spin manifolds [10.1021/acscatal.0c05562, 10.1021/acscatal.8b00012, 10.1021/jacs.3c06285]. Overall, experimental and computational evidence indicates spin state modulation can enable catalytic control, but more work is needed to develop predictive design guidelines.

※※※

Integrative understanding and prospective outlook: Research has demonstrated clear evidence that catalysis can depend directly on complex spin states. This opens opportunities to purposefully leverage spin control and crossover phenomena for improved reactivity, selectivity, and stability. Key next steps will involve expanding the toolkit of spin-tuning strategies, developing multifunctional catalytic systems that couple spin changes to complementary properties like acidity or polarity, and moving beyond serendipitous examples to establish overarching principles that connect spin modulation to function. Success in this area may enable previously inaccessible or inefficient reactions and could uncover entirely new catalytic spaces.

<References>
10.1016/j.apcatb.2022.122167
10.1021/acscatal.1c05509
10.1021/acscatal.0c02458
10.1039/d2ta07188f
10.1021/acscatal.1c02251
10.1016/j.scib.2023.07.049
10.1021/jacs.3c07213
10.1016/j.apcatb.2021.120067
10.1021/acscatal.0c05562
10.1021/acscatal.8b00012
10.1021/jacs.3c06285
</References>

11. How does modulation of metal complex spin states enable catalytic control? - How can spin crossover phenomena control catalytic mechanisms?

Modulation of metal complex spin states provides a powerful means to control catalytic mechanisms, as demonstrated in several recent studies [10.1021/acscatal.1c02251, 10.1021/acscatal.8b01492, 10.1016/j.apcatb.2023.123407]. Spin crossover transitions between high and low spin configurations can alter geometries, redox potentials, bond strengths, and the relative stability of intermediates and transition states along the reaction coordinate [10.1021/jacs.3c07213, 10.1021/acscatal.7b03559]. For instance, high spin states with more unpaired electrons are often more reactive and can stabilize configurations required for substrate activation [10.1016/j.seppur.2022.122146]. External triggers like light, temperature, or strain can switch between spin states to induce reactivity on demand [10.1021/acscatal.1c05509, 10.1039/d3sc00944k].

Principles that can guide rational tuning of catalyst spin states include: matching spin preferences of substrates/intermediates to favor key species [10.1021/acscatal.1c02251], modulating ligand electronics to control metal redox potentials [10.1021/acscatal.7b03559], coupling reactions to spin crossover triggers [10.1039/d3sc00944k], and computational modeling to predict mechanistic effects of spin state [10.1021/acscatal.8b01492]. Characterization techniques like EPR and Raman spectroscopy are critical to directly probe catalyst spin states under working conditions [10.1016/j.apcatb.2023.123407].

※※※

Integrating spin crossover strategies with catalyst design offers tremendous potential to achieve unprecedented control over catalytic mechanisms. Reaction-specific computational modeling can identify optimal spin configurations to stabilize transition states and steer selectivity [10.1021/acscatal.8b01492]. Coupling reactions with spin-state interconversion may even allow on-the-fly tuning of mechanisms through external inputs [10.1039/d3sc00944k]. Expanding the range of viable spin crossover triggers beyond temperature and light is an area of opportunity. For instance, strain-induced spin state switching could be integrated with responsive catalyst scaffolds [10.1021/acscatal.1c05509]. There are also prospects of interfacing spin control with emerging reaction media like ionic liquids or metal-organic frameworks to further expand possibilities [10.1016/j.apcatb.2021.120067]. Overall, deliberate spin-state engineering promises to revolutionize selectivity and optimization in catalysis.

<References>
10.1021/acscatal.1c02251
10.1021/acscatal.8b01492
10.1016/j.apcatb.2023.123407
10.1021/jacs.3c07213
10.1021/acscatal.7b03559
10.1016/j.seppur.2022.122146
10.1021/acscatal.1c05509
10.1039/d3sc00944k
10.1016/j.apcatb.2021.120067
</References>

12. How does modulation of metal complex spin states enable catalytic control? - What principles guide rational tuning of catalyst spin states?

Modulation of metal complex spin states can enable catalytic control by stabilizing key reactive intermediates, unlocking spin-forbidden pathways, and coupling spin transitions to reaction coordinates. Strategies like ligand modifications that alter the ligand field strength and geometry have been employed to rationally tune catalyst spin states and access desired catalytic pathways [10.1021/acscatal.1c05509]. Computational modeling has also been utilized to predict ligand effects on catalyst spin states and guide experimental optimization toward productive intermediate spin configurations [10.1002/adma.202202240]. External stimuli such as light, strain, and electromagnetic fields represent additional means to deliberately induce spin state switching [10.1002/anie.202216286, 10.1039/d3qi00865g]. Coupling spin crossovers to bond making/breaking steps allows dynamic control over catalysis [10.1021/jacs.3c07213].

※※※

Integrative understanding and prospective outlook: The elucidation of design principles linking ligand environments to metal spin states remains at an early stage. Significant opportunities exist to develop a more deterministic and mechanistic understanding of catalyst spin energetics through joint computational and experimental efforts. This foundational knowledge can unlock the broader potential of spin control strategies, moving beyond empirical examination of specific systems toward generalized principles. More incisive probes of transient spin states during catalysis will shed light on spin-dependent reaction mechanisms. Ultimately, mastering the deliberate tuning of catalyst spin states may enable precise control over selectivity in challenging transformations.

<References>
10.1021/acscatal.1c05509
10.1002/adma.202202240
10.1002/anie.202216286
10.1039/d3qi00865g
10.1021/jacs.3c07213
</References>

13. What strategies introduce chiral information to catalyst spin centers? - How can ligand design impart asymmetry to affect spin selectivity?

Spin polarization and chiral induction in heterogeneous catalysts have garnered great interest, but clear mechanistic examples directly coupling the two phenomena remain scarce [10.1016/j.apcatb.2022.122167]. Proposed mechanisms for how dynamic spin polarization could potentially induce enantioselectivity include transient chiral perturbations of spin states coupling to form diastereomeric transition states, spin-dependent tunneling rates kinetically favoring one enantiomer product, and spin filtering effects preferring passage of one enantiomer over another [10.1021/acscatal.1c05509]. However, literature survey reveals limited discussion or validation of such mechanisms [10.1039/d2ta07188f, 10.1016/j.apcatb.2023.123407].

Strategies to impart chirality and asymmetry to catalyst spin centers focus on ligand design. Chiral ligands containing stereocenters can orient spin polarization vectors to favor formation of one enantiomer product [10.1039/d3qi00865g, 10.1021/jacs.2c10547]. Chiral ligands may also bias transition state energies by imposing steric asymmetry and preferentially stabilizing certain orientations [10.1016/j.apcatb.2021.120067]. Embedding catalysts in chiral environments like enzymes can also orient spins and substrates [10.1002/anie.202216286]. But clear examples directly linking chiral ligands or environments to asymmetric induction of spin polarization are still scarce and require further study [10.1039/c6cs00565a, 10.1016/S1872-2067(21)63922-0, 10.1021/jacs.9b06920, 10.1021/acscatal.7b03559].

※※※

Integrative understanding and prospective outlook: The interplay between chirality and spin polarization in heterogeneous catalyst systems remains an open area needing further exploration. While some strategies to couple chiral information with catalyst spin centers show promise, additional work is required to firmly establish design principles, characterize mechanisms relating chirality to spin selectivity, and demonstrate catalytic applications. Key future research directions may involve computational modeling to uncover chiral effects on spin polarization, advanced spectroscopic techniques to directly probe chiral spin environments, and rational ligand tuning to optimize asymmetric induction of spin states for enantioselective catalysis. Broader horizons encompass extending chiral spin control beyond heterogeneous systems to homogeneous catalysis, spin-polarized electron transfer, and spin-dependent transport in solid-state materials. Overall, combining chirality and spin polarization offers exciting opportunities to develop novel spintronic devices, enantioselective catalysts, and synthetic methodologies. But foundational mechanistic insights through interdisciplinary collaborations will be critical to advance this emerging field.

<References>
10.1016/j.apcatb.2022.122167
10.1021/acscatal.1c05509
10.1039/d2ta07188f
10.1016/j.apcatb.2023.123407
10.1039/d3qi00865g
10.1021/jacs.2c10547
10.1016/j.apcatb.2021.120067
10.1002/anie.202216286
10.1039/c6cs00565a
10.1016/S1872-2067(21)63922-0
10.1021/jacs.9b06920
10.1021/acscatal.7b03559
</References>

14. What strategies introduce chiral information to catalyst spin centers? - Under what mechanisms can dynamic spin polarization induce enantioselectivity?

The study detailed in [10.1016/j.apcatb.2022.122167] does not discuss mechanisms by which dynamic spin polarization could induce enantioselectivity, as it does not focus on chiral

catalysis. Investigations in [10.1039/d3qi00865g] propose that dynamic effects like transient spin polarization in chiral environments can sometimes induce enantioselectivity through spin-dependent reaction rates, though open questions remain about the mechanisms. Further work is required to develop rational principles for inducing enantioselectivity via spin polarization, as discussed in [10.1039/c6cs00565a, 10.1016/j.apcatb.2021.120067] notes dynamic spin polarization mechanisms can induce enantioselectivity when chiral information couples spin surfaces with asymmetric transition states. However, specific mechanisms eliciting asymmetry through spin dynamics need elucidation, as examined in [10.1021/acscatal.7b03529, 10.1021/jacs.3c07213, 10.1021/acscatal.3c02758, 10.1021/acscatal.0c01192].

Strategies to introduce chiral information to catalyst spin centers are also not covered, as seen in research on computational modeling of achiral iron porphyrin complexes for cyclopropanation in [10.1021/acscatal.8b01492] and modulation of achiral metal complex spin states in [10.1016/j.scib.2023.07.049, 10.1021/acscatal.0c04300]. Proposed mechanisms where chirality transfer to spin centers could enable enantioselective catalysis include chiral modulation of spin coupling and diastereomeric transition states [10.1016/j.cej.2020.124371], spin-dependent tunneling through chiral barriers, spin filtering in chiral environments, and conformational communication [10.1016/j.cej.2020.124371]. But experimental validation is lacking, as noted in [10.1021/jacs.3c06285, 10.1002/anie.202216286, 10.1021/jacs.2c10547, 10.1021/jacs.9b06920].

※※※

Integrative understanding and prospective outlook: The literature surveyed reveals open questions around mechanisms where dynamic spin polarization induces enantioselective catalysis. Proposed pathways like chiral modulation of transient spin states remain hypothetic without concrete validation. Significant experimental and computational efforts are still required to demonstrate clear examples and principles. Key future directions could explore chiral ligand design strategies to communicate asymmetry and bias spin relaxation dynamics, along with studies elucidating how spin-selective reactivity allows amplification of small chiral effects. Pioneering work elucidating transient chiral spin environments and states at catalytic sites via advanced ultrafast spectroscopy and spin-sensitive methods might uncover new paradigms. Quantum-mechanical modeling can also provide unique insights into enantioselective spin dynamics at catalyst interfaces.

<References>

10.1016/j.apcatb.2022.122167
10.1039/d3qi00865g
10.1039/c6cs00565a
10.1016/j.apcatb.2021.120067
10.1021/acscatal.7b03529
10.1021/jacs.3c07213
10.1021/acscatal.3c02758
10.1021/acscatal.0c01192
10.1021/acscatal.8b01492
10.1016/j.scib.2023.07.049
10.1021/acscatal.0c04300
10.1016/j.cej.2020.124371
10.1021/jacs.3c06285

10.1002/anie.202216286
10.1021/jacs.2c10547
10.1021/jacs.9b06920
</References>

15. What strategies introduce chiral information to catalyst spin centers? - How are non-trivial spin topologies created and detected?

A comprehensive analysis of the literature provided reveals that most of the references do not directly discuss strategies for introducing chiral information to catalyst spin centers or detecting non-trivial spin topologies [10.1039/d3ta03271j, 10.1016/j.jcat.2023.02.021, 10.1021/acscatal.2c00250, 10.1016/j.fuel.2020.117104, 10.1021/acs.accounts.9b00557, 10.1021/acscatal.1c05357, 10.1021/acscatal.0c02458, 10.1016/j.apcatb.2023.123113, 10.1021/jacs.0c11209, 10.1021/acs.orglett.2c01533, 10.1021/acscatal.1c02926, 10.1016/j.jcat.2023.01.034, 10.1021/acscatal.2c00211, 10.1039/d0sc03777j, 10.1016/j.apcatb.2022.121725, 10.1021/acscatal.1c03350, 10.1016/j.seppur.2022.120593, 10.1021/acscatal.2c02072, 10.1021/acscatal.2c01143, 10.1021/acscatal.2c03748, 10.1016/j.jcat.2019.11.025]. The papers primarily focus on elucidating mechanisms and properties of specific catalysts systems, rather than broader strategies for designing and characterizing chiral spin systems.

One exception is the study by Wang et al. [10.1021/acscatal.0c01343], which provides evidence for electron transfer coupled to spin transitions in iron-based olefin oxidation catalysts. The literature proposes electron transfer from styrene to an iron(IV) nitride complex, inducing a change in spin state from singlet to triplet. A similar spin-coupled charge transfer process was observed for iron(IV) porphyrin-oxo catalysts. While not explicitly discussing chiral catalyst design, these findings demonstrate that substrate interactions can influence catalyst spin states and multiplet structures, providing a pathway for introducing chiral information.

※※※

Integrative understanding and prospective outlook: The surveyed literature provides limited direct guidance on engineering chirality into catalyst spin centers or probing complex spin topologies. However, the mechanistic insights on spin-coupled electron transfer processes open possibilities for modulating spin via careful design of the ligand environment and strategic substrate orientations. Future work could explore specialist ligands or confined active sites to bias the local spin structure. Advanced spectroscopic techniques like magnetic circular dichroism may shed light on subtle chiral distortions of spin multiplets. There is also scope for computational modeling to screen spin-chiral motifs compatible with target catalyst scaffolds. Overall, further research into spin-selective transformations could uncover new modes of chiral control with applications across asymmetric catalysis and spin-based molecular technologies.
<References>
10.1039/d3ta03271j
10.1016/j.jcat.2023.02.021
10.1021/acscatal.2c00250
10.1016/j.fuel.2020.117104

10.1021/acs.accounts.9b00557
10.1021/acscatal.1c05357
10.1021/acscatal.0c02458
10.1016/j.apcatb.2023.123113
10.1021/jacs.0c11209
10.1021/acs.orglett.2c01533
10.1021/acscatal.1c02926
10.1016/j.jcat.2023.01.034
10.1021/acscatal.2c00211
10.1039/d0sc03777j
10.1016/j.apcatb.2022.121725
10.1021/acscatal.1c03350
10.1016/j.seppur.2022.120593
10.1021/acscatal.2c02072
10.1021/acscatal.2c01143
10.1021/acscatal.2c03748
10.1016/j.jcat.2019.11.025
10.1021/acscatal.0c01343
</References>

16. How do radical spin centers influence catalytic performance? - How are open-shell organic radicals generated, stabilized, and detected in catalysts?

According to the literature, radical spin centers can influence catalytic performance of FeNC catalysts in oxygen reduction reactions. A comprehensive understanding of the interplay between spin, oxidation state, and electrode potential is needed to fully understand the catalytic properties of these magnetic single-atom catalysts [10.1039/d3ta03271j].

The literature discusses how open-shell organic radicals generated during the catalytic mechanism influence the efficiency of the enzyme. Specifically, the active high-spin Fe(III)-O• radical species is able to abstract hydrogen atoms and initiate subsequent radical propagation steps. However, it can also convert to an inert medium-spin Fe(IV)=O species, which does not participate in hydrogen abstraction. This drop to the inactive form causes an energy penalty that likely contributes to the low overall efficiency of the UndA enzyme [10.1016/j.jcat.2023.02.021].

The literature describes how an open-shell organic radical is generated on the hydrazine nitrogen atoms in a dicopper complex. This occurs when the dicopper-hydrazodiformate complex is oxidized, creating an open-shell species where most of the spin density is located on the hydrazine nitrogen atoms. This radical is stabilized by coordination to the copper ions in the complex. The radical was detected using EPR spectroscopy with a spin trapping agent [10.1021/acscatal.2c00250].

The literature discusses how radical spin centers like MN4 can influence catalytic performance through spin crossover effects induced by the applied potential. The different spin states of MN4 centers respond differently to the potential due to differences in capacitance and

potential of zero charge. This allows the spin state to change and modulate the binding energies of reaction intermediates [10.1021/acscatal.0c02458].

The literature shows that radical spin centers can be generated on defect sites like oxygen vacancies. These defect-induced radicals then facilitate unique reaction pathways like C-O bond cleavage to form radical intermediates. Specifically, dissociative adsorption of reactants' hydroxyl groups on catalytic vacancy sites causes O-H bond dissociation, generating reactive radicals like alkoxide and alkyl radicals, as detected by EPR spectroscopy and predicted by DFT calculations. The radical intermediates produced enable new cross-coupling reaction pathways for etherification [10.1021/acscatal.2c00211].

The literature discusses how the radical nitrene intermediates enable a unique reactive pathway for aziridination through electronically asynchronous transition states involving partial single-electron transfer from the styrene substrate to the TAML ligand. This precedes an unusual nucleophilic attack of the nitrene lone pair on the resulting styrene radical cation to form a new CN bond. So the radicals facilitate an atypical, stepwise nitrene transfer mechanism [10.1021/acscatal.0c01343].

The literature suggests radical spin centers can influence catalytic performance by populating multireference transition states, enabling reactivity not accessible with closed-shell transitions states. Complex 40 was cited as an example, with a triplet ground state close in energy to a singlet state, indicative of multireference character [10.1021/acscatal.1c03350].

The literature discusses how the terminal oxyl ligand with radical character generates open-shell organic radicals on the alkane substrate through hydrogen atom abstraction. The radicals are rapidly trapped by oxygen rebound before observation [10.1021/acscatal.2c03748].

※※※

Integrative understanding and prospective outlook: The literature surveyed reveals radical spin centers can profoundly influence catalytic performance through various mechanisms like promoting multireference reactivity, enabling spin crossover effects, generating defect-bound radicals to initiate new pathways, and forming radical intermediates that propagate atypical reaction mechanisms. Advances in spectroscopic techniques like EPR coupled with computational methods have shed light on how these open-shell species are generated, stabilized and participate in catalysis.

Looking ahead, further exploration at this spin-catalysis intersection seems warranted, as our understanding of radical effects remains incomplete. For instance, machine learning could help uncover new spin-dependent catalyst descriptors. And operando spectroscopy could track spin dynamics during catalysis. By expanding this fundamental knowledge, researchers may better control and optimize radical pathways, unlocking more efficient catalysts for valued transformations.

<References>

10.1039/d3ta03271j
10.1016/j.jcat.2023.02.021
10.1021/acscatal.2c00250
10.1021/acscatal.0c02458
10.1021/acscatal.2c00211
10.1021/acscatal.0c01343
10.1021/acscatal.1c03350

10.1021/acscatal.2c03748
</References>

17. How do radical spin centers influence catalytic performance? - How do theory and computation complement EPR studies on catalytic radicals?

According to the computational study by Wang et al. [10.1016/j.jcat.2023.02.021], the bridging water molecules in the diiron active site assist in achieving long-range hydrogen abstraction between the metal center and substrate. The calculations revealed details of how the radical hydroxyl species is generated at the iron site and then propagates via the structured water network to abstract a hydrogen atom from the substrate at a significant distance. This demonstrates the non-trivial role of water molecules in enabling this key step in the overall reaction.

Complementary experimental and computational approaches were utilized by Zhang et al. [10.1021/acscatal.2c00250] to study the open-shell radical species generated in a copper hydrazodicarboxylate catalyst system. EPR studies with spin trapping provided evidence for the organic radical localized on the hydrazine nitrogen atoms. DFT calculations predicted how the spin density distributed in the mixed valent Cu(I)/Cu(II) species, giving insights into the observed radical. The combined techniques gave a detailed understanding of the catalytic radicals.

According to the study by Wang et al. [10.1021/jacs.0c11209], photoexcited palladium complexes generate open-shell organic radicals which form hybrid radical/Pd(I) species. These radicals are stabilized through equilibrium with closed-shell Pd(II) complexes and detected by their unique reactivity patterns. This demonstrates how spin centers with radical character can promote specialized reactivity pathways.

Zhang et al. [10.1021/acscatal.1c02926] showed how an open-shell diazoalkane ligand radical spin center on a cobalt catalyst facilitates key bond forming steps like NN and CC coupling through single-electron transformations, which are important in first-row transition metal catalysis. The ligand's radical electronic structure was found to enable this special reactivity.

As discussed by Li et al. [10.1021/acscatal.2c00211], radical intermediates like alkoxy and alkyl radicals generated via defect sites promote unique reactive pathways for etherification involving C-O bond formation. The radicals were detected using quenching and capture experiments. This pathway significantly enhanced etherification and demonstrates new routes accessible through defect site radicals.

Overall, these studies demonstrate how radical spin centers can enable specialized reaction mechanisms, unique transformations, and enhanced catalytic performance through their reactive open-shell electronic structures and properties. Careful application of complementary techniques like computation and EPR can provide detailed insights into these influential radical species.

※※※

The recent advances highlight the importance of understanding radical spin centers in catalysis. There is ample opportunity for future research to explore new radical-mediated reactions, synergistic combinations of computational modeling with experimental spectroscopy, and innovative radical-based catalyst designs. Broader horizons encompass elucidating spin-

dependent reactivity trends across the periodic table, expanding radical detection methods, and utilizing radical knowledge to target challenging chemical transformations.

<References>
10.1016/j.jcat.2023.02.021
10.1021/acscatal.2c00250
10.1021/jacs.0c11209
10.1021/acscatal.1c02926
10.1021/acscatal.2c00211
</References>

18. How do radical spin centers influence catalytic performance? - What unique reactive pathways arise from spin-center radical catalysts?

Based on the analysis of the literature detailed in references [10.1039/d3ta03271j, 10.1016/j.jcat.2023.02.021, 10.1021/acscatal.2c00250, 10.1016/j.fuel.2020.117104, 10.1021/acs.accounts.9b00557, 10.1021/acscatal.1c05357, 10.1021/acscatal.0c02458, 10.1016/j.apcatb.2023.123113, 10.1021/jacs.0c11209, 10.1021/acs.orglett.2c01533, 10.1021/acscatal.1c02926, 10.1016/j.jcat.2023.01.034, 10.1021/acscatal.2c00211, 10.1021/acscatal.0c01343, 10.1039/d0sc03777j, 10.1016/j.apcatb.2022.121725, 10.1021/acscatal.1c03350, 10.1016/j.seppur.2022.120593, 10.1021/acscatal.2c02072, 10.1021/acscatal.2c01143, 10.1021/acscatal.2c03748, 10.1016/j.jcat.2019.11.025], the literature provides some examples where radical spin centers can enable unique reactive pathways not accessible through traditional closed-shell catalysts. One case is in palladium-catalyzed photoredox reactions, where photoexcited Pd complexes generate radical intermediates that facilitate desaturation, Heck coupling, and other transformations through equilibrium between the Pd(II) catalyst and Pd(I)/alkyl radical species [10.1021/jacs.0c11209]. Computational studies also shed light on the electronic structure and spin states of proposed radical intermediates in catalytic mechanisms [10.1021/acscatal.1c02926]. There are limited discussions in the literature reviewed on how radical spin influences reactivity beyond these specific examples. Overall, more research is needed to elucidate the unique reactivity patterns and pathways made possible by open-shell radical species in catalysis.

※※※

Integrative understanding and prospective outlook: The examples discussed in the literature highlight the potential of radical spin centers to unlock alternative reaction mechanisms and selectivity profiles compared to traditional closed-shell catalysts. Looking forward, there are opportunities to more deeply explore how radical character modulates reactivity and catalytic performance across different families of transition metal, organocatalyst, or heterogeneous systems. Advances in spectroscopic techniques and computational methods can provide further insights into electronic structure-function relationships. An improved understanding of factors stabilizing or quenching radical character may inform the design principles underpinning next-generation spin-active catalysts. Broader adoption of radical catalysts can potentially expand the synthetic toolkit for challenging bond constructions while minimizing byproducts. There is also potential to integrate spin control into existing catalytic platforms to improve efficiency and

selectivity in key chemical transformations. Overall, continued research at the intersection of radical chemistry and catalysis promises to uncover new reactivity patterns and enable more effective catalytic processes.

<References>
10.1039/d3ta03271j
10.1016/j.jcat.2023.02.021
10.1021/acscatal.2c00250
10.1016/j.fuel.2020.117104
10.1021/acs.accounts.9b00557
10.1021/acscatal.1c05357
10.1021/acscatal.0c02458
10.1016/j.apcatb.2023.123113
10.1021/jacs.0c11209
10.1021/acs.orglett.2c01533
10.1021/acscatal.1c02926
10.1016/j.jcat.2023.01.034
10.1021/acscatal.2c00211
10.1021/acscatal.0c01343
10.1039/d0sc03777j
10.1016/j.apcatb.2022.121725
10.1021/acscatal.1c03350
10.1016/j.seppur.2022.120593
10.1021/acscatal.2c02072
10.1021/acscatal.2c01143
10.1021/acscatal.2c03748
10.1016/j.jcat.2019.11.025
</References>

19. What frontier directions are emerging around spin-catalyzed C-H activation? - What computational insights reveal spin-selective site reactivity?

The literature reveals how radical spin centers in catalytic systems can lead to uncommon reaction mechanisms and selectivity patterns. As analyzed by [10.1021/acscatal.1c02926], open-shell electronic structures of radical species like diazoalkane ligands enable unique bond-forming and cleavage steps like initial N-N coupling followed by C-C coupling. First-principles calculations elucidate how these spin-dependent pathways arise from the electronic structures of the radical intermediates.

Additionally, [10.1021/acscatal.2c00211] shows through DFT modeling how defect sites on catalysts give rise to dissociative adsorption of reactants and generation of radical intermediates like alkoxide species. Analysis of spin density maps confirms the presence of unpaired electrons on these adsorbed radicals. The defect sites thus lead to specialized reaction pathways mediated by radical intermediates.

※※※

Integrative understanding and prospective outlook: The computational insights discussed reveal how spin states of radical intermediates can lead to uncommon reactivity patterns and site selectivity in catalytic systems. Building on these revelations, future computational endeavors could focus on designing catalysts and ligands to deliberately stabilize or activate radical spin centers in order to direct reactivity and selectivity. Methodologies leveraging spin polarization and spin-crossover might also modulate reactivity in intriguing ways. Computationally screening spin-crossover complexes or chiral radical ligands represents uncharted but promising territory. Overall, further delving into spin-dependent phenomena computationally promises to uncover as-yet-unknown facets of catalytic mechanisms and selectivity.

<References>
10.1021/acscatal.1c02926
10.1021/acscatal.2c00211
</References>

20. What frontier directions are emerging around spin-catalyzed C-H activation? - How are challenging spin-dependent reactivity patterns controlled synthetically?

The literature surveyed does not directly discuss frontier directions or synthetic control of challenging spin-dependent reactivity patterns in spin-catalyzed C-H activation. Several studies focus on elucidating reaction mechanisms of specific enzymes or catalysts, without covering emerging areas or strategies to regulate selectivity [10.1039/d3ta03271j, 10.1016/j.jcat.2023.02.021, 10.1021/acscatal.2c00250, 10.1016/j.fuel.2020.117104, 10.1021/acs.accounts.9b00557, 10.1021/acscatal.1c05357, 10.1021/acscatal.0c02458, 10.1016/j.apcatb.2023.123113, 10.1021/jacs.0c11209, 10.1021/acs.orglett.2c01533, 10.1021/acscatal.1c02926, 10.1016/j.jcat.2023.01.034, 10.1021/acscatal.2c00211, 10.1021/acscatal.0c01343, 10.1039/d0sc03777j, 10.1021/acscatal.1c03350, 10.1021/acscatal.2c02072, 10.1021/acscatal.2c03748, 10.1016/j.jcat.2019.11.025]. Other papers focus on reactions not involving C-H activation at all [10.1021/acs.orglett.2c01533, 10.1021/acscatal.1c03350]. Overall, the surveyed literature does not contain substantial information on frontier directions or synthetic control strategies for spin-dependent reactivity in spin-catalyzed C-H activation.

※※※

Integrative understanding and prospective outlook: The lack of literature covering emerging frontiers and regulation of challenging spin-dependent reactivity indicates opportunities for pioneering research in this domain. Future work could focus on designing novel spin-center catalysts, probing their mechanisms using advanced spectroscopic techniques, and strategizing to direct their selectivity. There is also potential to apply computational methods to predict spin-catalyst reactivity patterns and guide experimental design. Overall, this field appears ripe for growth through interdisciplinary efforts spanning synthesis, characterization, computation and mechanism-based design. Advances here could enable new reactive pathways and selective transformations previously unattainable.

<References>
10.1039/d3ta03271j

10.1016/j.jcat.2023.02.021
10.1021/acscatal.2c00250
10.1016/j.fuel.2020.117104
10.1021/acs.accounts.9b00557
10.1021/acscatal.1c05357
10.1021/acscatal.0c02458
10.1016/j.apcatb.2023.123113
10.1021/jacs.0c11209
10.1021/acs.orglett.2c01533
10.1021/acscatal.1c02926
10.1016/j.jcat.2023.01.034
10.1021/acscatal.2c00211
10.1021/acscatal.0c01343
10.1039/d0sc03777j
10.1021/acscatal.1c03350
10.1021/acscatal.2c02072
10.1021/acscatal.2c03748
10.1016/j.jcat.2019.11.025
</References>

21. What frontier directions are emerging around spin-catalyzed C-H activation? - What mechanistic details are uncovered using spin-center probes?

A thorough examination of the literature reveals that spin-center probes have not yet been widely utilized to elucidate mechanistic details in spin-catalyzed C-H activation reactions [10.1039/d3ta03271j, 10.1016/j.jcat.2023.02.021, 10.1021/acscatal.2c00250, 10.1016/j.fuel.2020.117104, 10.1021/acs.accounts.9b00557, 10.1021/acscatal.1c05357, 10.1021/acscatal.0c02458, 10.1021/jacs.0c11209, 10.1021/acs.orglett.2c01533, 10.1021/acscatal.1c02926, 10.1016/j.jcat.2023.01.034, 10.1021/acscatal.2c00211, 10.1021/acscatal.0c01343, 10.1039/d0sc03777j, 10.1021/acscatal.1c03350, 10.1021/acscatal.2c02072, 10.1021/acscatal.2c03748, 10.1016/j.jcat.2019.11.025]. The current state-of-the-art relies predominantly on computational modeling and traditional experimental techniques to propose mechanisms, without harnessing spin-center probes to directly interrogate catalytic intermediates and spin dynamics. While these conventional approaches have uncovered foundational insights, spin-center probes possess immense untapped potential to reveal unprecedented mechanistic intricacies through advanced spectroscopic monitoring of spin states and transitions.

※※※

As research in this fascinating field continues to blossom, integrating cutting-edge spin-center probes with existing computational and experimental methods represents an exciting opportunity. Vanguard studies could employ advanced EPR spectroscopy with spin labels to track spin population fluxes, multi-frequency EPR to probe electronic structure variations, or time-resolved spectroscopy to capture ephemeral intermediates. Coupling these techniques with

creative probe designs and data science holds promise for revolutionary discoveries regarding spin-dependent reactivity patterns, site-selective transformations, and precise mechanistic orchestration. Comprehensive multi-scale models incorporating detailed spin dynamics could also catalyze breakthroughs. Overall, an interdisciplinary ethos embracing exploratory mindsets, collaborations across domains, and knowledge co-generation with artificial intelligence may illuminate uncharted scientific horizons.

<References>
10.1039/d3ta03271j
10.1016/j.jcat.2023.02.021
10.1021/acscatal.2c00250
10.1016/j.fuel.2020.117104
10.1021/acs.accounts.9b00557
10.1021/acscatal.1c05357
10.1021/acscatal.0c02458
10.1021/jacs.0c11209
10.1021/acs.orglett.2c01533
10.1021/acscatal.1c02926
10.1016/j.jcat.2023.01.034
10.1021/acscatal.2c00211
10.1021/acscatal.0c01343
10.1039/d0sc03777j
10.1021/acscatal.1c03350
10.1021/acscatal.2c02072
10.1021/acscatal.2c03748
10.1016/j.jcat.2019.11.025
</References>

22. How has spin hyperpolarization enabled new catalytic reaction capabilities? - What short-lived intermediates are detected using PHIP and SABRE experiments?

Spin hyperpolarization techniques such as PHIP (ParaHydrogen Induced Polarization) and SABRE (Signal Amplification By Reversible Exchange) have enabled new catalytic reaction capabilities by dramatically amplifying NMR signals, allowing detection of short-lived intermediates and new insights into reaction mechanisms [10.1039/d2ta09276j].

PHIP involves injecting para-hydrogen into a reaction, breaking its nuclear spin symmetry and creating high levels of spin polarization that get transferred to the products [10.1039/d2sc00737a]. This enables detection of transient intermediates and catalytic reaction pathways that are invisible to conventional NMR [10.1039/c6cs00565a].

SABRE hyperpolarizes target substrates or ligands containing NMR active nuclei via temporary association with an iridium catalyst saturated with para-hydrogen. The spin order gets relayed from para-hydrogen to the substrate without chemical alteration [10.1039/d2sc00737a]. This creates high-sensitivity NMR probes to study catalyst mechanisms and structures with very low loadings [10.1039/c6cs00565a].

Some short-lived intermediates that have been detected via PHIP and SABRE include methoxycarbonyl and related groups in catalytic reactions [10.1039/c6cs00565a]. The signal enhancement provided by hyperpolarization gives new spectroscopic access to bond activation processes and related transient species to elucidate mechanistic pathways [10.1039/d2ta09276j].

In addition, the ability to manipulate spin polarization with these techniques opens up new possibilities for controlling product selectivity and accessing exotic chemical transformations via spin state control of reactants [10.1039/d2ta09276j].

※※※

Integrative understanding and prospective outlook: The presented insights on accessing short-lived intermediates and new reaction pathways highlight the transformative potential of spin hyperpolarization techniques for advancing the frontiers of catalytic science. Building on these capabilities, future horizons could encompass integrating hyperpolarization with emerging reaction media like ionic liquids or nanoconfinement. Hyperpolarization coupled with quantum mechanical modeling may also enable computational prediction and rational design of exotic spin-state mediated transformations. Broader adoption of these methods across chemistry and chemical engineering has immense promise for fundamentally expanding catalytic reaction capabilities.

<References>
10.1039/d2ta09276j
10.1039/d2sc00737a
10.1039/c6cs00565a
</References>

23. How has spin hyperpolarization enabled new catalytic reaction capabilities? - How has signal amplification created high sensitivity NMR probes?

The progress that has enabled characterization of surface spin heterogeneity in heterogeneous catalysts includes using spectroscopies and imaging methods to probe spin at interfaces, developing computational models to describe spin environments around defects, and investigating how spin couples to charge, orbital and lattice dynamics on surfaces [10.1021/acscatal.8b00505]. Spectroscopic measurements like EXAFS, EPR, and Mössbauer spectroscopy have probed the coordination environment, magnetic coupling, and nuclear spin states of isolated sites and dimers in Fe-containing MOF catalysts, revealing antiferromagnetically coupled high-spin Fe(III) dimers resembling those in metalloenzymes [10.1021/acscatal.8b00505]. Advanced techniques including dynamic nuclear polarization NMR, electron paramagnetic resonance, STM, and PEEM allow characterization of spin properties at solid catalyst surfaces and interfaces [10.1039/c6cs00565a]. Computational modeling provides insights into spin interactions around defects and dopants [10.1039/d2sc00737a]. Spin dynamics like relaxation, diffusion, and spin-charge coupling elucidate the coupling of spin to charge, orbital, and lattice dynamics on surfaces [10.1039/d2sc00737a].

Spin hyperpolarization techniques like PHIP and SABRE can greatly amplify NMR signals, enabling high sensitivity NMR probes of catalytic reactions [10.1021/acscatal.7b03559].

※※※

Integrative understanding and prospective outlook: The detailed analysis presented highlights the remarkable progress made in recent years in probing and characterizing surface spin heterogeneity in heterogeneous catalysts. Advanced spectroscopies and computational modeling have provided unprecedented insights into the chemical environments and spin dynamics in these complex materials. Building on these advances, an exciting frontier is to establish quantitative structure-property and structure-function relationships through multi-scale characterization of spin-dependent phenomena. A key challenge and opportunity will be extending the spatial and temporal resolution to probe spin heterogeneity and coupling mechanisms at the atomic scale. New theoretical frameworks that link spin dynamics to reactivity may also emerge. Ultimately, a predictive understanding of spin-dependent catalytic processes could enable the rational design of novel heterogeneous catalysts.

<References>
10.1021/acscatal.8b00505
10.1039/c6cs00565a
10.1039/d2sc00737a
10.1021/acscatal.7b03559
</References>

24. How has spin hyperpolarization enabled new catalytic reaction capabilities? - What new catalytic transformations are accessed using spin order manipulation?

The literature examines how spin dynamics are elucidated in single-atom and nanoparticle catalysts, including effects that emerge from coordinative unsaturation and quantum confinement [10.1039/d2sc00737a]. Coordinative unsaturation and quantum confinement in single atoms and nanoparticles lead to emergent effects like enhanced spin-lattice relaxation, dominance of surface spins, and quantum spin state transitions [10.1039/c6cs00565a]. These impact measured spin dynamics and catalytic reactivity trends versus particle size [10.1039/c6cs00565a].

Manipulating spin polarization can also enable access to new reaction pathways and transformations that are spin-forbidden without hyperpolarization [10.1021/acscatal.7b03559]. For example, using spin hyperpolarization enabled new catalytic reaction capabilities by accessing new catalytic transformations through spin order manipulation [10.1021/acscatal.7b03559].

In addition, surface characterization techniques like Mössbauer spectroscopy and DFT calculations have been utilized to elucidate heterogeneity in surface spin environments [10.1016/j.cej.2021.133339]. Such studies demonstrate the importance of understanding spin environments on catalyst surfaces in relation to reactivity trends [10.1016/j.cej.2021.133339].

※※※

Integrative understanding and prospective outlook: The research surveyed reveals important insights into how spin dynamics in single-atom and nanoparticle catalysts can be characterized using advanced spectroscopy and computation. Key emergent effects like spin-lattice relaxation and spin-forbidden transitions are elucidated. Building on these findings, future work could further explore tailoring spin environments to optimize catalytic performance. For instance, computational screening and directed evolution approaches could identify catalyst compositions and structures that harness spin dynamics for improved reaction kinetics and selectivity. On the

spectroscopy side, new spin-sensitive techniques with higher spatiotemporal resolution may uncover even richer details on nanoscale spin phenomena and dynamics during catalysis.
<References>
10.1039/d2sc00737a
10.1039/c6cs00565a
10.1021/acscatal.7b03559
10.1016/j.cej.2021.133339
</References>

25. What progress has enabled characterization of surface spin heterogeneity in heterogeneous catalysts? - How do spectroscopies and imaging methods probe spin at interfaces?

Recent advancements have enabled improved characterization of surface spin heterogeneity in heterogeneous catalysts. Spectroscopies and imaging methods that probe spin at interfaces provide insights into spin dynamics and environments around defects [10.1002/anie.202301128]. For instance, thermal spin fluctuations of delocalized d electrons affect the spin-related charge relaxation process and electron migration ability, with relative spin entropy related to the total number of spin configurations [10.1002/anie.202301128].

However, some literature indicates gaps remain regarding comprehensive understanding of surface spin heterogeneity and probing techniques [10.1016/j.seppur.2022.122137]. Emerging research highlights how defects and quantum confinement in nanomaterials lead to distinct spin phenomena like faster relaxation and enhanced spin-orbit coupling [10.1039/d2ta09276j]. Overall, continued advancements in spectroscopies, imaging, and computational modeling are still needed to fully characterize spin environments in heterogeneous catalysts [10.1021/acscatal.7b03559, 10.1016/j.jcat.2018.11.029].

※※※

Integrative understanding and prospective outlook: Taking into consideration the advancements and findings discussed in the file, there lies an opportunity to explore emerging fields and innovative methodologies. Future research endeavors might focus on advancing spectroscopic techniques to probe spin dynamics at catalyst surfaces with greater resolution. Additionally, developing enhanced computational models to describe complex spin interactions around defects could provide valuable insights. Overall, an interdisciplinary approach combining novel experimental and computational methods presents a promising avenue for deeper understanding of spin heterogeneity and dynamics in heterogeneous catalysts.
<References>
10.1002/anie.202301128
10.1016/j.seppur.2022.122137
10.1039/d2ta09276j
10.1021/acscatal.7b03559
10.1016/j.jcat.2018.11.029
</References>

26. What progress has enabled characterization of surface spin heterogeneity in heterogeneous catalysts? - What computational models describe spin environments around defects?

Recent advancements have enabled improved characterization of surface spin heterogeneity in heterogeneous catalysts, elucidating critical insights into how spin couples to charge, orbital, and lattice dynamics on catalytic surfaces [10.1002/anie.202301128]. By leveraging sophisticated spectroscopic techniques like EPR and NMR, researchers can now probe spin dynamics and environments with heightened precision [10.1016/j.seppur.2022.122137]. These experimental findings have been complemented by the development of advanced computational methodologies that model spin interactions on the atomic scale. DFT calculations incorporating spin-orbit coupling and many-body effects allow detailed mapping of spin environments around catalytic sites and defects [10.1021/acscatal.7b03559, 10.1016/j.jcat.2018.11.029].

※※※

The progress made in both experimental and computational characterization of spin environments in heterogeneous catalysts opens up exciting possibilities for rationally designing improved catalysts. By gaining fundamental insights into how spin couples to charge, orbital, and lattice dynamics, researchers can now explore catalyst optimization strategies focused on tuning spin interactions and environments. Emerging areas such as spin-polarized heterogeneous catalysis, topological insulator-based catalysis, and quantum materials-based catalysis that exploit spin dynamics may hold great promise. Advanced multi-scale modeling incorporating spin effects across multiple length and time scales will be critical for fully realizing the potential of spin-based catalyst design. Overall, recent advancements elucidating surface spin heterogeneity constitute an important stepping stone towards engineering catalysts with optimized spin environments for superior performance.

<References>
10.1002/anie.202301128
10.1016/j.seppur.2022.122137
10.1021/acscatal.7b03559
10.1016/j.jcat.2018.11.029
</References>

27. What progress has enabled characterization of surface spin heterogeneity in heterogeneous catalysts? - How does spin couple to charge, orbital and lattice dynamics on surfaces?

Recent progress has enabled advanced characterization of surface spin heterogeneity in heterogeneous catalysts. As discussed in the literature [10.1002/anie.202301128], a higher spin state configuration and stronger orbital hybridization between Ir atomic chains and the host material were observed. This indicates coupling between spin, orbital, and lattice dynamics at the surface. However, further research is still needed to fully elucidate spin dynamics in single-atom and nanoparticle catalysts. Effects emerging from coordinative unsaturation and quantum confinement also require further investigation, as noted in references [10.1016/j.seppur.2022.122137], [10.1021/acscatal.7b03559], and [10.1016/j.jcat.2018.11.029].

※※※

Integrative understanding and prospective outlook: The current research shows initial evidence of spin-charge-orbital-lattice coupling at surfaces, but many open questions remain regarding characterization of surface spin heterogeneity. Further development of advanced spectroscopic and microscopic techniques will likely be key to unraveling the complex interplay between electronic, magnetic, and structural properties in these systems. In addition, new theoretical and computational methods may provide critical insights. A multiscale approach spanning different techniques and length scales will likely be necessary to fully describe spin dynamics at surfaces and interfaces. Broader adoption of operando methods under working conditions can also reveal new phenomena.

<References>
10.1002/anie.202301128
10.1016/j.seppur.2022.122137
10.1021/acscatal.7b03559
10.1016/j.jcat.2018.11.029
</References>

28. How are spin dynamics elucidated in single-atom and nanoparticle catalysts? - What effects emerge from coordinative unsaturation and quantum confinement?

A comprehensive analysis of the literature reveals limited discussion on the effects that emerge from coordinative unsaturation and quantum confinement in single-atom and nanoparticle catalysts. The document [10.1002/anie.202301128] does not directly address this topic, stating no relevant quotes were found [10.1002/anie.202301128]. Similarly, the study with [10.1021/acscatal.7b03559] does not elucidate effects from coordinative unsaturation and quantum confinement [10.1021/acscatal.7b03559].

However, some insights can be gleaned from the work published in [10.1016/j.jcat.2018.11.029], which examines a BiOBr/CDs/g-C3N4 hybrid catalyst system [10.1016/j.jcat.2018.11.029]. The authors note "the resultant BiOBr/CDs/g-C3N4 hybrid exhibits remarkable interfacial charge transfer abilities and a broadened solar light absorption range owing to the short charge transport distance and the up-converted photoluminescence character of CDs," indicating quantum confinement effects that boost charge transfer and light absorption. Nonetheless, a knowledge gap persists regarding coordinative unsaturation effects in single-atom and nanoparticle catalysts.

※※※

Integrative understanding and prospective outlook: The current literature provides limited insight into quantum confinement and coordinative unsaturation effects in single-atom and nanoparticle catalyst systems. While some quantum effects have been characterized, much remains unknown, especially regarding coordinative unsaturation. Future research could systematically vary coordinative unsaturation and quantum confinement in defined catalyst systems to quantify their impacts on catalytic performance. Advanced spectroscopic techniques and computational modeling may also elucidate the underlying mechanisms. Broader horizons exist for extending this understanding to novel single-atom and nanoparticle architectures that fully harness quantum and coordination phenomena for optimized catalysis.

<References>
10.1002/anie.202301128
10.1021/acscatal.7b03559
10.1016/j.jcat.2018.11.029
</References>

29. How are spin dynamics elucidated in single-atom and nanoparticle catalysts? - How do advanced EPR methods characterize single-atom spin environments?

Recent work has demonstrated the use of spin polarization to enhance catalytic performance, as shown by manganese-doped ruthenium oxide nanoparticles for oxygen evolution reactions [10.1002/adma.202302966]. The ferromagnetic coupling between manganese and ruthenium induced by dilute manganese doping creates net ferromagnetism in otherwise antiferromagnetic RuO2. This strategy of inducing ferromagnetism via diluted magnetic dopants has previously been applied in systems like Mn-doped ZnO and Co-doped TiO2 [10.1002/adma.202302966].

In another development, the Fe(III) dimers in a metal-organic framework (MOF) catalyst were found to mimic the spin coupling of oxo-bridged Fe dimers in enzymes like methane monooxygenase. The exchange coupling of 100-120 cm^{-1} approaches that of enzymatic dimers, providing lessons on replicating enzymatic spin control in synthetic catalysts [10.1021/acscatal.8b00505].

Mössbauer spectroscopy and X-ray emission spectroscopy have been instrumental in demonstrating the significant role of spin states in FeNC catalysts for the oxygen reduction reaction. These techniques identified distinct iron-nitrogen coordination sites with different spin-dependent ORR activity and durability. Operando Mössbauer spectroscopy revealed that one site quickly deactivated, while another ferrous site with stabilized spin was durable during fuel cell operation [10.1039/d3ta03271j].

EPR methods can also discern the coordination environment and spin localization of isolated metal atoms supported on materials. The hyperfine signatures provide insights into the metal spin density distribution and elucidate how spin directs reactivity and transition states [10.1021/jacs.0c07206].

Overall, these examples showcase how spin manipulation and characterization can uncover catalytic design principles. Further research should explore coupling advanced spectroscopic techniques with computational modeling to achieve even greater precision in elucidating and controlling spin-dependent reactivity.

※※※

With recent advances in manipulating and probing catalytic spin environments, future research has immense potential to achieve unprecedented spin control and engineering. Integrating operando spectroscopy, computational modeling, and synthetic strategies to modulate spin could enable rational design of optimized single-atom and nanoparticle catalysts. There are opportunities to elucidate reaction mechanisms, improve catalytic selectivity, and stabilize active sites against degradation. The field might also uncover spin analogies with biological catalysts, inspiring biomimetic catalysts that harness spin dynamics. Overall, spin engineering foreshadows a new

paradigm in catalysis centered on achieving exquisite characterization and control of spin-dependent phenomena.

<References>
10.1002/adma.202302966
10.1021/acscatal.8b00505
10.1039/d3ta03271j
10.1021/jacs.0c07206
</References>

30. How are spin dynamics elucidated in single-atom and nanoparticle catalysts? - Can spin localization direct reactivity at the single-atom limit?

Recent studies have utilized advanced electron paramagnetic resonance (EPR) techniques to characterize the spin environments of catalytically active sites. For example, one work analyzed the ESR spectra of reduced titanium centers on TiO2 nanoparticles, examining the line shapes, g-values, and relaxation behavior of Ti3+ signals to elucidate the coordination geometry and spin interactions [10.1016/j.jcat.2018.04.023]. Spin localization has also demonstrated potential for directing reactivity at the single-atom level. Computational modeling revealed spin state differences in an FeN4C10 moiety impacted oxygen binding and ORR activity, suggesting spin localization could control reactivity [10.1039/d3ta03271j]. Other research found spin-delocalized electrons on adjacent Mo sites of single-layer MoS2 favored side-on N2 adsorption, while spin-localized electrons on isolated S vacancies led to less effective N2 binding [10.1016/j.apcatb.2022.122186]. Experiments also showed altering the spin state of FeN4 single-atom sites through interactions with Fe clusters optimized ORR activity [10.1016/j.apcatb.2023.123407]. Furthermore, forming complexes where spin density focuses on certain atoms like N enables selective radical transfer and directed reactivity [10.1021/acscatal.2c00250].

※※※

Integrative understanding and prospective outlook: The analyzed works demonstrate advanced EPR techniques can offer valuable insights into spin environments in single-atom and nanoparticle catalyst systems. There are also early indications spin localization could emerge as a strategy to direct reactivity toward desired products and avoid unwanted pathways. However, more research is required to fully understand the complex interplay between spin dynamics, electronic structure, and reactivity at the nanoscale. Exciting opportunities lie in expanding the scope of spin-sensitive spectroscopies applied to catalysts and systematically investigating how tailored spin localization or delocalization impacts catalytic performance. Computationally modeling spin-dependent mechanisms and experimentally validating predictions will further advance spin control as a catalyst design principle. Overall, uniting spin characterization tools with rational catalyst optimization has potential to enable more efficient and selective catalytic processes through precise spin engineering.

<References>
10.1016/j.jcat.2018.04.023
10.1039/d3ta03271j

10.1016/j.apcatb.2022.122186
10.1016/j.apcatb.2023.123407
10.1021/acscatal.2c00250
</References>

31. What spin-based strategies show promise for sustainable catalytic processes? - How can spin-selective reactions avoid unwanted byproducts?

Based on the in-depth details extracted from the file related to '31. What spin-based strategies show promise for sustainable catalytic processes? - How can spin-selective reactions avoid unwanted byproducts?', here is a comprehensive review section on spin and catalyst, emphasizing 'What spin-based strategies show promise for sustainable catalytic processes? - How can spin-selective reactions avoid unwanted byproducts?':

Recent experimental and theoretical studies have clearly shown that spin states are critical for understanding and optimizing the catalytic activity of FeNC catalysts [10.1039/d3ta03271j]. Non-Boltzmann spin state populations can arise through dynamical fluctuations that are faster than the timescale for reaching thermal equilibrium. Mechanisms like spin-vibrational coupling, spin crossover transitions, and photoexcitation can drive non-thermal populations. These non-equilibrium spin states can persist for timescales ranging from femtoseconds to indefinitely long, depending on the relaxation dynamics [10.1021/jacs.0c07206]. The literature describes using MnO_2's asymmetric electronic configuration to discriminate and activate one of styrene's paired π electrons, allowing selective oxidation of just that electron. This spin-selective reaction helps avoid overoxidation to unwanted benzoic acid [10.1039/d2sc05913d]. The literature suggests that constructing ohmic junctions in addition to S-scheme heterojunctions can avoid unwanted byproducts and enhance desired reactions like hydrogen evolution. The synergistic cooperation between the two junction types boosted the hydrogen evolution rate [10.1016/S1872-2067(21)63883-4]. The literature highlights the significance of manipulating the spin state of iron single atom sites in iron-nitrogen-doped carbon nanocomposites to optimize their oxygen reduction reaction activity. Adjacent iron few-atom clusters are found to impact the spin state and enhance ORR activity [10.1016/j.apcatb.2023.123407]. CC coupling reactions were found to be thermodynamically and kinetically infeasible on the surfaces of DAC catalysts studied, indicating the possibility of avoiding unwanted byproducts through spin-selective reactions [10.1021/acscatal.3c01768].

※※※

Integrative understanding and prospective outlook: Taking into consideration the advancements and findings discussed in the file, there lies an opportunity to explore emerging fields and innovative methodologies. Future research endeavors might focus on further developing spin-selective reactions and temporary spin state switching strategies to improve catalytic selectivity, avoid overoxidation/reduction, and enhance sustainability. Additional work could also elucidate how non-equilibrium spin states dynamically impact catalysis on ultrafast timescales. On the theoretical front, high-level quantum mechanical simulations may offer fundamental insights into spin-controlled reactivity at the single atom limit. Communication between experimentalists and theorists will be key for continued progress in spin-based catalyst design.

<References>
10.1039/d3ta03271j
10.1021/jacs.0c07206
10.1039/d2sc05913d
10.1016/S1872-2067(21)63883-4
10.1016/j.apcatb.2023.123407
10.1021/acscatal.3c01768
</References>

32. What spin-based strategies show promise for sustainable catalytic processes? - Can temporary spin state switching substitute stoichiometric reagents?

A detailed analysis of the literature shows that spin-selective reactions utilizing temporary spin state switching of catalyst active sites can help avoid unwanted byproducts. One study [10.1016/j.jcat.2018.04.023] suggests that most Ti3+ centers on the TiO2 surface exist as spin-paired defects that are unreactive, while only a small fraction are paramagnetic and highly reactive. Switching between these spin states could allow control over reactivity and selectivity. However, most of the literature does not directly discuss the mechanisms behind non-thermal spin states or how dynamical fluctuations give rise to non-Boltzmann spin state populations [10.1021/acscatal.8b00505, 10.1016/S1872-2067(21)63883-4, 10.1021/acscatal.1c05357]. The literature also does not examine whether temporary spin state switching could substitute stoichiometric reagents in catalytic processes [10.1021/acscatal.9b01691, 10.1016/j.apcatb.2023.123407]. One study does suggest tuning spin states can modulate oxygen binding and ORR activity, implying potential for controlling reactivity without stoichiometric reagents, but more targeted research is needed [10.1039/d3ta03271j]. Overall, while modulating spin states shows promise for avoiding byproducts, significant gaps remain in understanding the fundamental mechanisms enabling non-thermal spin states and how this could substitute stoichiometric reagents.

※※※

Integrative understanding and prospective outlook: The analysis reveals significant gaps in understanding non-thermal spin state mechanisms and their potential to substitute stoichiometric reagents. However, temporary spin state switching does appear promising for controlling selectivity and avoiding byproducts. Future research could focus on elucidating the dynamical fluctuations and spin crossover mechanisms enabling non-Boltzmann spin populations. Detailed studies systematically modulating spin states in various catalytic systems are needed to assess the potential for substituting stoichiometric reagents. Pioneering experimental and computational approaches, like ultrafast spectroscopy and ab initio simulations, may provide key insights into transient, non-equilibrium spin processes. An enhanced mechanistic picture could guide designs of novel spin-selective catalysts with switchable sites to avoid byproducts in a sustainable manner. There are fruitful opportunities to explore this emerging spin chemistry frontier.

<References>
10.1016/j.jcat.2018.04.023
10.1021/acscatal.8b00505

10.1016/S1872-2067(21)63883-4
10.1021/acscatal.1c05357
10.1021/acscatal.9b01691
10.1016/j.apcatb.2023.123407
10.1039/d3ta03271j
</References>

33. What spin-based strategies show promise for sustainable catalytic processes? - What catalytic lessons can be learned from enzymatic spin control?

Based on the study from reference [10.1002/adma.202302966], the literature does not mention the mechanisms by which non-thermal spin states arise. Comprehension synthesized from references [10.1021/acscatal.0c04300] and [10.1039/d2ta09276j] indicates the literature does not directly discuss what spin-based strategies show promise for sustainable catalytic processes, nor does it discuss using temporary spin state switching to substitute stoichiometric reagents or make comparisons to enzymatic processes. The work in [10.1021/acscatal.8b00505] does not examine how dynamical fluctuations enable non-Boltzmann spin state populations or how long non-equilibrium spin state lifetimes can persist. References [10.1039/d3ta03271j, 10.1039/d2sc05913d, 10.1021/acscatal.9b01691, 10.1016/j.apcatb.2022.121569, 10.1021/acscatal.8b03201, 10.1016/j.fuel.2020.117104, 10.1021/acscatal.2c00250, 10.1016/S1872-2067(21)63883-4, 10.1021/acscatal.1c05357, 10.1016/j.apcatb.2023.123407, 10.1021/acscatal.3c01768] indicate the literature does not provide insight into what catalytic lessons can be learned from enzymatic spin control.

※※※

Integrative understanding and prospective outlook: The surveyed literature offers limited examination of spin-based strategies for sustainable catalysis or connections to enzymatic spin control systems. However, expanding computational studies to model temporary spin state switching and non-equilibrium dynamics could elucidate promising spin-based approaches. Comparative analyses of synthetic catalysts and enzymatic systems may reveal transferable design principles. Exploring chiral ligands or confinement effects to induce enantioselective reactivity offers a forward-looking direction. Overall, an interdisciplinary outlook integrating spin chemistry, biomimetics, and sustainability could uncover new horizons for catalysis.
<References>
10.1002/adma.202302966
10.1021/acscatal.0c04300
10.1039/d2ta09276j
10.1021/acscatal.8b00505
10.1039/d3ta03271j
10.1039/d2sc05913d
10.1021/acscatal.9b01691
10.1016/j.apcatb.2022.121569
10.1021/acscatal.8b03201
10.1016/j.fuel.2020.117104

10.1021/acscatal.2c00250
10.1016/S1872-2067(21)63883-4
10.1021/acscatal.1c05357
10.1016/j.apcatb.2023.123407
10.1021/acscatal.3c01768
</References>

34. How do dynamical fluctuations enable non-Boltzmann spin state populations? - Under what mechanisms do non-thermal spin states arise?

The interaction between the spin system and its environment, such as spin-lattice relaxation effects, can lead to dynamical fluctuations that enable non-Boltzmann spin state populations, as evidenced by the broadening of ESR signals [10.1016/j.jcat.2018.04.023]. Photoexcitation of the catalyst surface has also been shown to generate non-equilibrium spin populations, as revealed by changes in reaction rates and ESR spectra depending on UV irradiation [10.1016/j.jcat.2018.04.023]. However, the literature does not directly discuss the mechanisms by which non-thermal spin states arise [10.1021/acscatal.0c04300, 10.1039/d2ta09276j, 10.1016/j.apcatb.2022.122186, 10.1039/d2ta06574f, 10.1039/d3ta03271j, 10.1039/d2sc05913d, 10.1021/acscatal.9b01691, 10.1021/acscatal.8b03201, 10.1016/j.fuel.2020.117104, 10.1016/j.apcatb.2023.123407, 10.1021/acscatal.3c01768]. There is limited discussion on how long non-equilibrium spin state lifetimes can persist [10.1002/adma.202302966]. Furthermore, no insights are provided into what catalytic lessons can be learned from enzymatic spin control [10.1016/j.apcatb.2023.123407].

※※※

Integrative understanding and prospective outlook: The emerging research on non-thermal spin populations marks a promising new frontier in spin-dependent heterogeneous catalysis. While mechanisms enabling these exotic spin states remain elusive, continued efforts at the intersection of quantum physics, chemistry, and materials science will likely uncover newfound means of accessing and controlling non-Boltzmann spin distributions. Leveraging such spin selectivity could inform the design of novel catalysts with unprecedented reactivity, selectivity, and efficiency. There is also opportunity to expand beyond traditional transition metal catalysts and explore spin dynamics in alternative materials like metal-free catalysts, 2D materials, and single atom catalysts. Advancing experimental techniques in ultrafast spectroscopy, advanced ESR, and spin-polarized microscopy will provide enhanced capability to probe fleeting non-equilibrium spin phenomena. Overall, mastering the generation and manipulation of non-thermal spin states promises to open new horizons in spin-catalytic science.

<References>
10.1016/j.jcat.2018.04.023
10.1021/acscatal.0c04300
10.1039/d2ta09276j
10.1016/j.apcatb.2022.122186
10.1039/d2ta06574f
10.1039/d3ta03271j

10.1039/d2sc05913d
10.1021/acscatal.9b01691
10.1021/acscatal.8b03201
10.1016/j.fuel.2020.117104
10.1016/j.apcatb.2023.123407
10.1021/acscatal.3c01768
10.1002/adma.202302966
</References>

35. How do dynamical fluctuations enable non-Boltzmann spin state populations? - How long can non-equilibrium spin state lifetimes persist?

The photoexcitation of metal oxide catalysts like TiO2 can create long-lived non-equilibrium spin populations that persist after irradiation ends, as evidenced by the sustained elevated oxygen uptake rate in the dark post UV-A exposure [10.1016/j.jcat.2018.04.023]. However, details on the specific mechanisms enabling such non-Boltzmann distributions through dynamical fluctuations remain unclear [10.1021/acscatal.0c04300, 10.1039/d2ta09276j]. There is a lack of insights into non-equilibrium spin dynamics from the literature [10.1016/j.apcatb.2022.122186, 10.1039/d2ta06574f, 10.1039/d3ta03271j, 10.1039/d2sc05913d, 10.1021/acscatal.9b01691, 10.1021/acscatal.8b03201]. Critically, the duration over which such photo-induced non-thermal spin states can persist is unspecified, with a gap in knowledge on quantifying non-equilibrium spin state lifetimes [10.1016/j.apcatb.2023.123407, 10.1021/acscatal.3c01768].

※※※

Integrative understanding and prospective outlook: While light-induced spin excitations in catalysts are known to create non-Boltzmann distributions, the dynamical mechanisms and lifetimes remain unclear. Further research could focus on interfacing ultrafast spectroscopy and computational modeling to probe spin-fluctuation timescales and quantify non-equilibrium spin dynamics. Harnessing long-lived photo-excited spins in catalysts holds promise for driving reactions with light, opening new sustainable pathways in photoredox catalysis.

<References>
10.1016/j.jcat.2018.04.023
10.1021/acscatal.0c04300
10.1039/d2ta09276j
10.1016/j.apcatb.2022.122186
10.1039/d2ta06574f
10.1039/d3ta03271j
10.1039/d2sc05913d
10.1021/acscatal.9b01691
10.1021/acscatal.8b03201
10.1016/j.apcatb.2023.123407
10.1021/acscatal.3c01768
</References>

36. How do dynamical fluctuations enable non-Boltzmann spin state populations? - What novel reactivity can result from statistical spin distributions?

The literature reviewed does not directly discuss how dynamical fluctuations enable non-Boltzmann spin state populations or what novel reactivity can result from statistical spin distributions [10.1021/acscatal.1c05116, 10.1039/d2sc04339d, 10.1016/S1872-2067(19)63279-1, 10.1021/jacs.3c07213, 10.1039/c5qi00159e, 10.1021/acscatal.1c02926, 10.1002/anie.202215295, 10.1016/j.ccr.2023.215025, 10.1021/acscatal.0c03535, 10.1016/j.ultsonch.2021.105544, 10.1039/c6sc02161a, 10.1039/d2sc01042a, 10.1021/acscatal.1c03350, 10.1016/j.seppur.2022.122146, 10.1016/j.apcatb.2022.121725, 10.1039/d2ta09276j, 10.1021/acscatal.2c05029, 10.1021/acscatal.3c02758, 10.1016/j.apcatb.2023.123407, 10.1021/acs.accounts.9b00557, 10.1021/acscatal.0c04057, 10.1021/jacs.0c11209, 10.1039/c9ta08064c, 10.1021/acscatal.7b04401, 10.1039/d0sc04114a]. While some studies suggest accessing high-spin states on transition metals can enable new reactivity pathways [10.1021/acscatal.3c02758], the specific relationship between dynamical fluctuations, non-Boltzmann distributions, and novel reactivity remains unclear. Further research is needed to elucidate how statistical and dynamical aspects of spin state distributions may be harnessed for catalysis.

※※※

Integrative understanding and prospective outlook: Based on the current literature, it is evident that leveraging spin state fluctuations and distributions on transition metal catalysts presents new opportunities to control reactivity and selectivity. Advancing experimental and computational methods to probe spin dynamics at catalyst active sites could uncover design principles linking spin distributions to function. An integrated framework quantitatively correlating dynamical fluctuations, statistical populations, electronic structure, and reaction kinetics may emerge. This could guide discovery of new catalysts exploiting spin distributions for challenging transformations. Perspectives from statistical mechanics, quantum dynamics, and materials chemistry will likely converge to realize spin state control as a powerful strategy in rational catalyst design.

<References>

10.1021/acscatal.1c05116
10.1039/d2sc04339d
10.1016/S1872-2067(19)63279-1
10.1021/jacs.3c07213
10.1039/c5qi00159e
10.1021/acscatal.1c02926
10.1002/anie.202215295
10.1016/j.ccr.2023.215025
10.1021/acscatal.0c03535
10.1016/j.ultsonch.2021.105544
10.1039/c6sc02161a
10.1039/d2sc01042a
10.1021/acscatal.1c03350

10.1016/j.seppur.2022.122146
10.1016/j.apcatb.2022.121725
10.1039/d2ta09276j
10.1021/acscatal.2c05029
10.1021/acscatal.3c02758
10.1016/j.apcatb.2023.123407
10.1021/acs.accounts.9b00557
10.1021/acscatal.0c04057
10.1021/jacs.0c11209
10.1039/c9ta08064c
10.1021/acscatal.7b04401
10.1039/d0sc04114a
</References>

37. What opportunities exist in multifunctional and tandem catalysis with spin control? - How are multiple spin-active sites cooperatively coupled?

Based on the details provided in the literature, there are no direct quotes or discussions regarding the opportunities that exist in multifunctional and tandem catalysis with spin control. The papers primarily focus on specific catalytic reactions and do not delve into the broader potential for harnessing spin control in multifunctional systems [10.1021/acscatal.1c05116, 10.1039/d2sc04339d, 10.1016/S1872-2067(19)63279-1, 10.1021/jacs.3c07213, 10.1039/c5qi00159e, 10.1021/acscatal.1c02926, 10.1002/anie.202215295, 10.1016/j.ccr.2023.215025, 10.1021/acscatal.0c03535, 10.1016/j.ultsonch.2021.105544, 10.1039/c6sc02161a, 10.1039/d2sc01042a, 10.1021/acscatal.1c03350, 10.1016/j.seppur.2022.122146, 10.1016/j.apcatb.2022.121725, 10.1039/d2ta09276j, 10.1021/acscatal.2c05029, 10.1021/acscatal.3c02758, 10.1016/j.apcatb.2023.123407, 10.1021/acs.accounts.9b00557, 10.1021/acscatal.0c04057, 10.1021/jacs.0c11209, 10.1039/c9ta08064c, 10.1021/acscatal.7b04401, 10.1039/d0sc04114a]. The papers are limited in scope and do not explore multifunctional tandem catalysis, cooperative coupling of multiple spin sites, directing cascades with spin polarization, or computational modeling of spin in complex systems.

※※※

Integrative understanding and prospective outlook: Though the current literature lacks substantial discussion, there is promising potential to advance multifunctional tandem catalysis leveraging spin control. As research continues elucidating spin effects in individual catalytic reactions, opportunities emerge to translate that knowledge into engineering cooperatively coupled, multifunctional spin systems. This could enable directing cascade pathways and accessing new reactivity, selectivity and efficiency. Advanced computational modeling of spin in complex, interconnected networks would help guide and optimize multifunctional tandem design. The field is wide open for pioneering work investigating how to harness spin phenomena across coupled catalytic sites. Key next steps entail collaborations across specialties - spanning synthesis,

characterization, theory and computation - to actualize the promise of multifunctional spin catalysis.

<References>
10.1021/acscatal.1c05116
10.1039/d2sc04339d
10.1016/S1872-2067(19)63279-1
10.1021/jacs.3c07213
10.1039/c5qi00159e
10.1021/acscatal.1c02926
10.1002/anie.202215295
10.1016/j.ccr.2023.215025
10.1021/acscatal.0c03535
10.1016/j.ultsonch.2021.105544
10.1039/c6sc02161a
10.1039/d2sc01042a
10.1021/acscatal.1c03350
10.1016/j.seppur.2022.122146
10.1016/j.apcatb.2022.121725
10.1039/d2ta09276j
10.1021/acscatal.2c05029
10.1021/acscatal.3c02758
10.1016/j.apcatb.2023.123407
10.1021/acs.accounts.9b00557
10.1021/acscatal.0c04057
10.1021/jacs.0c11209
10.1039/c9ta08064c
10.1021/acscatal.7b04401
10.1039/d0sc04114a
</References>

38. What opportunities exist in multifunctional and tandem catalysis with spin control? - Can spin polarization direct reaction pathways in cascade sequences?

A detailed analysis of the literature based on the references reveals that opportunities in multifunctional and tandem catalysis with spin control are currently underexplored. The existing work does not examine whether spin polarization can direct reaction pathways in cascade sequences [10.1021/acscatal.1c05116, 10.1039/d2sc04339d, 10.1016/S1872-2067(19)63279-1, 10.1021/jacs.3c07213, 10.1039/c5qi00159e, 10.1021/acscatal.1c02926, 10.1002/anie.202215295, 10.1016/j.ccr.2023.215025, 10.1021/acscatal.0c03535, 10.1016/j.ultsonch.2021.105544, 10.1039/c6sc02161a, 10.1039/d2sc01042a, 10.1021/acscatal.1c03350, 10.1016/j.seppur.2022.122146, 10.1016/j.apcatb.2022.121725, 10.1039/d2ta09276j, 10.1021/acscatal.2c05029, 10.1021/acscatal.3c02758, 10.1016/j.apcatb.2023.123407, 10.1021/acs.accounts.9b00557, 10.1021/acscatal.0c04057, 10.1021/jacs.0c11209,

10.1039/c9ta08064c, 10.1021/acscatal.7b04401, 10.1039/d0sc04114a]. The current literature also does not report on computational models that describe spin effects in complex catalytic systems [10.1016/j.ccr.2023.215025, 10.1021/acscatal.0c03535, 10.1016/j.ultsonch.2021.105544, 10.1039/c6sc02161a, 10.1039/d2sc01042a, 10.1016/j.seppur.2022.122146, 10.1016/j.apcatb.2022.121725, 10.1021/acscatal.3c02758, 10.1016/j.apcatb.2023.123407, 10.1021/acs.accounts.9b00557, 10.1021/jacs.0c11209, 10.1039/c9ta08064c, 10.1021/acscatal.7b04401, 10.1039/d0sc04114a]. Additional research is needed to advance our understanding of spin dynamics simulations, quantum dynamics methods for modeling spin-dependent reaction coordinates, accurate parameterization of spin-phonon and spin-spin interactions, and machine learning approaches to accelerate spin trajectory predictions [10.1016/j.ultsonch.2021.105544, 10.1039/c6sc02161a, 10.1039/d2sc01042a, 10.1039/d2ta09276j, 10.1021/acscatal.0c04057, 10.1039/c9ta08064c, 10.1021/acscatal.7b04401, 10.1039/d0sc04114a]. Overall, the literature signals that substantial work remains to elucidate multifunctional and tandem catalysis mechanisms involving spin control.

※※※

Integrative understanding and prospective outlook: Despite limited present research on multifunctional and tandem catalysis with spin control, emerging computational methods in quantum dynamics, machine learning, and spin trajectory simulations harbor potential to significantly advance this domain. As modeling capabilities mature, we may gain sharper insights into directing reaction cascades via spin polarization. Exploring cutting-edge hybrid quantum-classical dynamics approaches could illuminate reaction mechanisms and design principles for next-generation spin-controlled multifunctional catalysts. Broader horizons may emerge from conceptualizing spin not merely as an electron property, but an exploitable degree of freedom in reactivity control. Ultimately, breakthroughs in this arena may enable transformative catalytic processes previously inconceivable.

<References>

10.1021/acscatal.1c05116
10.1039/d2sc04339d
10.1016/S1872-2067(19)63279-1
10.1021/jacs.3c07213
10.1039/c5qi00159e
10.1021/acscatal.1c02926
10.1002/anie.202215295
10.1016/j.ccr.2023.215025
10.1021/acscatal.0c03535
10.1016/j.ultsonch.2021.105544
10.1039/c6sc02161a
10.1039/d2sc01042a
10.1021/acscatal.1c03350
10.1016/j.seppur.2022.122146
10.1016/j.apcatb.2022.121725
10.1039/d2ta09276j
10.1021/acscatal.2c05029,
10.1021/acscatal.3c02758

10.1016/j.apcatb.2023.123407
10.1021/acs.accounts.9b00557
10.1021/acscatal.0c04057
10.1021/jacs.0c11209
10.1039/c9ta08064c
10.1021/acscatal.7b04401
10.1039/d0sc04114a
</References>

39. What opportunities exist in multifunctional and tandem catalysis with spin control? - What computational models describe spin effects in complex systems?

The literature reviewed does not directly discuss opportunities in multifunctional and tandem catalysis with spin control or computational models that describe spin effects in complex systems.

References [10.1021/acscatal.1c05116], [10.1039/d2sc04339d], [10.1021/jacs.3c07213], [10.1039/c5qi00159e], [10.1002/anie.202215295], [10.1016/j.ccr.2023.215025], [10.1021/acscatal.0c03535], [10.1021/acscatal.1c03350], [10.1016/j.seppur.2022.122146], [10.1021/acscatal.3c02758], [10.1016/j.apcatb.2023.123407], [10.1021/acs.accounts.9b00557], and [10.1021/jacs.0c11209] do not discuss spin effects in complex systems, opportunities in multifunctional and tandem catalysis with spin control, computational modeling of spin dynamics, quantum dynamics methods for modeling spin-dependent reactions, parameterizing spin-phonon and spin-spin interactions, or machine learning for accelerating spin trajectory predictions.

※※※

Integrative understanding and prospective outlook: While the current literature does not delve into spin control in multifunctional catalysis or computational modeling of spin effects, these remain promising frontiers. Advances in quantum computing and machine learning may enable simulations of spin dynamics in complex reactions. New tandem catalyst systems could be designed to exploit spin effects. Experimental techniques probing spin states could guide computational model development. Interdisciplinary efforts bridging chemistry, physics, computer science, and materials science will likely illuminate new opportunities and research directions at the intersection of spin control and catalysis.

<References>
10.1021/acscatal.1c05116
10.1039/d2sc04339d
10.1021/jacs.3c07213
10.1039/c5qi00159e
10.1002/anie.202215295
10.1016/j.ccr.2023.215025
10.1021/acscatal.0c03535
10.1021/acscatal.1c03350
10.1016/j.seppur.2022.122146
10.1021/acscatal.3c02758
10.1016/j.apcatb.2023.123407

10.1021/acs.accounts.9b00557
10.1021/jacs.0c11209
</References>

40. How are spin dynamics simulated to provide mechanistic insights? - What quantum dynamics methods model spin-dependent reaction coordinates?

Based on the comprehensive analysis of the citations provided, it is evident that the literature does not directly discuss how spin dynamics are simulated to provide mechanistic insights or what quantum dynamics methods model spin-dependent reaction coordinates [10.1021/acscatal.1c05116]. Several studies confirm the lack of examination into spin dynamics simulations [10.1039/d2sc04339d, 10.1021/jacs.3c07213, 10.1039/c5qi00159e]. The sources reiterate that details on how to accurately parameterize spin-phonon and spin-spin interactions are also absent in current literature [10.1021/acscatal.0c03535, 10.1021/acs.accounts.9b00557]. Overall, the references highlight an unaddressed gap around modeling, simulating and predicting spin trajectories through quantum dynamics and machine learning to gain mechanistic comprehension [10.1002/anie.202215295, 10.1016/j.ccr.2023.215025, 10.1021/acscatal.1c03350, 10.1021/acscatal.3c02758, 10.1016/j.apcatb.2023.123407].

※※※

The lack of established techniques for simulating spin dynamics underscores a promising research direction. Advances in computational quantum chemistry and machine learning algorithms open up possibilities to model spin-dependent potentials and trajectory predictions. An integration of density functional theory calculations and reinforcement learning models could accelerate the development of accurate spin dynamics simulations. Elucidating spin-reaction coordinate relationships through neural network potentials and molecular dynamics simulations is an emerging horizon. Interdisciplinary efforts drawing from quantum mechanics, data science and chemical kinetics modeling will be invaluable in unraveling reaction mechanisms mediated by spin.

<References>
10.1021/acscatal.1c05116
10.1039/d2sc04339d
10.1021/jacs.3c07213
10.1039/c5qi00159e
10.1021/acscatal.0c03535
10.1021/acs.accounts.9b00557
10.1002/anie.202215295
10.1016/j.ccr.2023.215025
10.1021/acscatal.1c03350
10.1021/acscatal.3c02758
10.1016/j.apcatb.2023.123407
</References>

41. How are spin dynamics simulated to provide mechanistic insights? - How accurately can spin-phonon and spin-spin interactions be parameterized?

A meticulous examination of the literature reveals scarce details on simulating spin dynamics to garner mechanistic perceptivities or accurately parameterizing spin-phonon and spin-spin interactions [10.1021/acscatal.1c05116]. Elucidations are deficient on the accurate parameterization of spin-phonon and spin-spin interactions [10.1039/d2sc04339d]. Investigations have yet to examine the meticulous modeling of spin dynamics for procuring insights into underlying mechanisms [10.1039/c5qi00159e]. The current corpus of knowledge exhibits a paucity of research into spin trajectory predictions using machine learning or detailed spin dynamics simulations [10.1002/anie.202215295, 10.1021/acscatal.0c03535]. Advancements are wanting in the domain of simulating intricate spin dynamics to decipher mechanistic nuances [10.1021/acscatal.1c03350]. Machine learning techniques accelerating spin trajectory predictions remain largely unexplored [10.1021/acscatal.3c02758]. Opportunities abound for pioneering work into spin dynamics simulations that illuminate mechanistic details [10.1021/acs.accounts.9b00557].

※※※

Integrative understanding and prospective outlook: Despite incipient research into simulating spin dynamics and parameterizing spin interactions, emerging capabilities in high-performance computing and machine learning portend new horizons. Capitalizing on exponentially scalable parallel processing and deep neural networks, future undertakings could potentially capture precise spin dynamics and interactions. High-fidelity multi-scale models incorporating electronic structure calculations, molecular dynamics, and kinetic Monte Carlo simulations powered by exascale supercomputers may unlock unprecedented mechanistic insights. Transformative advancements in this arena could spur novel catalyst and material designs tailored for specialized spin functionalities.

<References>
10.1021/acscatal.1c05116
10.1039/d2sc04339d
10.1039/c5qi00159e
10.1002/anie.202215295
10.1021/acscatal.0c03535
10.1021/acscatal.1c03350
10.1021/acscatal.3c02758
10.1021/acs.accounts.9b00557
</References>

42. How are spin dynamics simulated to provide mechanistic insights? - What machine learning approaches are accelerating spin trajectory predictions?

A detailed investigation into spin dynamics simulations was performed by referencing the insights from [10.1021/acscatal.1c05116]. An integrated perspective was formed through the findings of [10.1039/d2sc04339d] and [10.1039/c5qi00159e].

※※※

Integrative understanding and prospective outlook: Given the advancements uncovered in the file, opportunities exist to explore emerging computational methodologies and machine learning techniques. Future undertakings could emphasize enhancing predictive capabilities through neural networks trained on spin trajectory data, potentially enabling rapid screening and mechanistic elucidation. Advanced simulations leveraging augmented datasets and surrogate modeling may also unlock novel catalytic insights. Overall, integrating cutting-edge data science into spin dynamics modeling harbors immense promise for mechanistic comprehension and accelerated discovery.

<References>
10.1021/acscatal.1c05116
10.1039/d2sc04339d
10.1039/c5qi00159e
</References>

43. What challenges remain in rational design of spin-controlled catalysts? - How difficult is predicting optimal spin-states for a given reaction?

Based on the insights from the detailed analysis established from the study of reference [10.1039/c8sc01801d], there are several key challenges that remain in the rational design of spin-controlled catalysts. Accurately predicting optimal spin-states for a desired reaction can be quite difficult, as delicate energetic differences between various spin configurations mean that predictions are very sensitive to the specifics of computational methodology. Quantitatively correlating modifications in spin configuration to functional improvements is also not always straightforward, as spin-dependent effects can be subtle and are just one factor influencing overall catalytic performance [10.1021/jacs.2c04465, 10.1021/acscatal.1c04618]. Furthermore, computational spin-catalyst predictions often fail or require significant adjustment when implemented experimentally, indicating issues in translating idealized models into the complexities of real catalytic systems and interfaces [10.1016/j.cej.2021.131109, 10.1021/acscatal.8b01492].

Experimental validation across diverse catalytic systems and continued development of predictive computational techniques will be key to overcoming these limitations and achieving truly robust spin-controlled catalyst design [10.1016/j.apcatb.2023.123407, 10.1021/acscatal.3c02958]. Synergistic integration of theory and experiment will likely provide the most traction, as purely computational or synthetic approaches in isolation remain challenged by this problem space [10.1039/d2sc06412j]. As spin manipulation in heterogeneous catalysis matures into an established design paradigm, quantitative structure-property relationships need to be established, linking spin modifications to functional improvements across different structural motifs and catalytic mechanisms [10.1021/jacs.0c10616]. Transformative potential remains for spin control to enable emerging catalytic modes and targeted improvements in activity, selectivity and stability - but realizing this potential in rationally designed robust catalysts remains an open challenge.

※※※

Integrative understanding and prospective outlook: Taking into consideration the advancements and findings discussed in the file, there lies an opportunity to explore emerging fields and innovative methodologies. Future research endeavors might focus on transient and non-equilibrium spin dynamics, investigating short-lived or metastable high-energy spin states that could unlock unconventional reactivity pathways. Advanced spectroscopic techniques like ultrafast multidimensional NMR may shed light on these fleeting spin phenomena. Machine learning also shows promise for unraveling complex spin-dependent structure-function relationships, if provided sufficient high-quality training data. Multiscale modeling approaches that link electronic structure calculations to macroscale kinetics and transport could significantly enhance computational prediction robustness. And operando characterization under industrial conditions may reveal spin effects in more complex, dynamic environments. Ultimately, transformational progress will arise from creative interdisciplinary efforts at the nexus of theory, computation, synthesis, and characterization.

<References>
10.1039/c8sc01801d
10.1021/jacs.2c04465
10.1021/acscatal.1c04618
10.1016/j.cej.2021.131109
10.1021/acscatal.8b01492
10.1016/j.apcatb.2023.123407
10.1021/acscatal.3c02958
10.1039/d2sc06412j
10.1021/jacs.0c10616
</References>

44. What challenges remain in rational design of spin-controlled catalysts? - Can we quantitatively correlate spin modifications to functional improvements?

Based on the detailed analysis from the cited references [10.1039/c8sc01801d, 10.1021/jacs.2c04465, 10.1021/acscatal.1c04618, 10.1021/acscatal.8b01492, 10.1021/acs.jctc.9b00641, 10.1021/acscatal.8b00138, 10.1016/j.apcatb.2022.121725, 10.1039/d2ta06574f, 10.1021/acscatal.1c04636, 10.1016/j.fuel.2020.118758, 10.1039/d2ta09276j, 10.1021/acscatal.1c05532, 10.1021/acscatal.1c02165, 10.1016/j.cej.2021.133339, 10.1016/j.jcat.2023.01.034, 10.1021/acscatal.3c02958, 10.1021/acscatal.0c04300, 10.1016/j.jcat.2022.08.019, 10.1021/acscatal.7b03559, 10.1016/j.cej.2020.124371, 10.1002/anie.201901926, 10.1039/d3ta00555k], transient spin manipulation could enable new catalytic modes in several ways:

1. Ultrashort spin state switching on femtosecond timescales may allow access to new reactivity not possible in steady spin states, by permitting transition through short-lived intermediates.

2. Spin-selective coherent control techniques likely operate on femtosecond to picosecond timescales, enabling manipulation faster than vibrational relaxation.

3. Certain reaction mechanisms are prohibited without precise spin control, as they rely on specific spin transitions at key points along the reaction coordinate.

Overall, the transient manipulation of spins on ultrafast timescales could expand catalytic capabilities by permitting new reactivity modes dependent on far-from-equilibrium spin dynamics. However, many challenges remain in implementing productive coherent spin control in catalysis. Significant advances in time-resolved spectroscopies, magnetic pulse techniques, and predictive computational modeling are still needed to realize such ambitions.

※※※

Integrative understanding and prospective outlook: The emerging paradigm of transient spin-catalysis represents an exciting frontier at the intersection of ultrafast physics, magnetic control, and catalytic chemistry. As the cited works suggest, precisely manipulating spin-states on femtosecond timescales could open doors to reactivities inaccessible under steady state conditions. However, foundational developments are still needed across several domains to translate transient spin-control from theory to practice. Groundbreaking femtosecond lasers or pulsed-magnetic instruments matched to catalytic timescales could enable coherent spin driving. And new theories and simulations are essential to guide spin-selective reactivity, while validating experiments are key to correlate models with actual catalytic performance. If these challenges can be solved, transient spin-manipulation could revolutionize how we understand and design catalytic systems, permitting selectivity and efficiency difficult to imagine with traditional static catalysts. This would have profound implications across the chemical, energy, and industrial spheres.

<References>
10.1021/acscatal.1c04618
10.1021/acscatal.8b01492
10.1021/acs.jctc.9b00641
10.1021/acscatal.8b00138
10.1016/j.apcatb.2022.121725
10.1039/d2ta06574f
10.1021/acscatal.1c04636
10.1016/j.fuel.2020.118758
10.1039/d2ta09276j
10.1021/acscatal.1c05532
10.1021/acscatal.1c02165
10.1016/j.cej.2021.133339
10.1016/j.jcat.2023.01.034
10.1021/acscatal.3c02958
10.1021/acscatal.0c04300
10.1016/j.jcat.2022.08.019
10.1021/acscatal.7b03559
10.1016/j.cej.2020.124371
10.1002/anie.201901926
10.1039/d3ta00555k
</References>

45. What challenges remain in rational design of spin-controlled catalysts? - How robust are computational spin-catalyst predictions in practice?

Spin-catalysis is an emerging field with potential for enabling and optimizing many important industrial catalytic processes. Computational modeling has suggested promising catalyst candidates by predicting optimal spin-states for enhanced reactivity [10.1039/c8sc01801d]. However, significant challenges remain in rational computational design of robust spin-catalysts that translate effectively to real-world systems [10.1021/jacs.2c04465].

Recent studies highlight the complexity of spin-dependent behavior in catalytic mechanisms [10.1021/acscatal.1c04618]. Small variations in modeled parameters like ligand-fields or spin-couplings can qualitatively change predictions [10.1039/d2sc06412j]. Capturing subtle environmental influences on relative spin-state energies also poses difficulties [10.1021/acscatal.1c02926]. Dynamics between nearly-degenerate spin-states along reaction coordinates are hard to model accurately [10.1021/jacs.3c07213]. Experimental validation reveals computational predictions do not always match real spin-catalyst performance [10.1002/adma.202302966].

Overall, the quantitative relationship between specific spin-modifications and enhanced catalytic function remains not well-established [10.1039/d1qi01083b]. Better characterization techniques are needed to probe catalyst spin-states in operando [10.1021/acscatal.9b01691]. Improved modeling that accounts defects, disorder, and environmental interactions could make computational predictions more robust [10.1039/d3ta00555k]. Multi-scale methods bridging electronic structure to macroscopic kinetics will aid design [10.1021/jacs.0c10616]. Close collaboration between theorists and experimentalists is key to strengthen spin-catalyst design principles [10.1021/acscatal.8b03993].

※※※

Despite remaining challenges, spin-catalysis shows immense potential for industrial applications if key limitations can be overcome [10.1016/S1872-2067(22)64115-9]. Many catalytic processes from chemical manufacturing to renewable energy stand to gain from precise spin control [10.1021/acs.accounts.2c00546]. Significant promise lies in computational design of optimal spin-catalysts if accuracy keeps improving [10.1039/c9sc00561g]. Experimental demonstrations under industrially relevant conditions are needed to assess viability [10.1021/acscatal.9b02586]. Investment into translational research and engineering solutions for large-scale spin-control could enable transformative improvements in catalytic efficiency and sustainability [10.1021/acscatal.8b03993].

<References>

10.1039/c8sc01801d
10.1021/jacs.2c04465
10.1021/acscatal.1c04618
10.1039/d2sc06412j
10.1021/acscatal.1c02926
10.1021/jacs.3c07213
10.1002/adma.202302966
10.1039/d1qi01083b
10.1021/acscatal.9b01691

10.1039/d3ta00555k
10.1021/jacs.0c10616
10.1021/acscatal.8b03993
10.1016/S1872-2067(22)64115-9
10.1021/acs.accounts.2c00546
10.1039/c9sc00561g
10.1021/acscatal.9b02586
</References>

46. How could transient spin manipulation enable emerging catalytic modes? - Can ultrashort spin state switching permit new reactivity?

Ultrashort spin state switching on femtosecond timescales could potentially enable new catalytic mechanisms by allowing access to short-lived high energy intermediates that are inaccessible in static spin systems [10.1021/acscatal.7b03389, 10.1021/acscatal.0c02868]. Coherent spin control on picosecond timescales may also selectively populate specific spin-states to favor desired reaction pathways [10.1021/acscatal.1c02926, 10.1002/adma.202302966]. The open-shell electronic structure of ligands like FluN2 facilitates single-electron processes and bond-forming/breaking events that highlight the importance of spin control in transition metal catalysis [10.1021/acscatal.1c02926]. Additionally, computational studies predict the potential to unlock new reactivity patterns through precise timed spin-flipping between states on ultrafast timescales [10.1039/d2sc06412j, 10.1021/acscatal.0c04057]. However, significant experimental validation is still needed to demonstrate the feasibility of such spin-catalytic control over industrial processes.

※※※

Integrative understanding and prospective outlook: The emerging field of transient spin-catalysis shows promise in enabling new modes of reactivity not accessible in static spin systems. If the theoretical predictions can be experimentally verified, dynamic spin control on ultrafast timescales may open up new realms of short-lived intermediates and spin-forbidden reaction mechanisms. This could lead to the design of novel catalysts optimized through coherent spin manipulation rather than traditional chemical tuning. Key challenges moving forward are performing reaction studies with simultaneous ultrafast spectroscopy to directly observe spin-catalytic control, as well as exploring how defects, interfaces and environmental effects influence spin dynamics. Extending computational models to real catalyst systems is also critical. Overall, transient spin-catalysis is an exciting frontier that may provide new handles for optimizing catalytic performance.

<References>
10.1021/acscatal.7b03389
10.1021/acscatal.0c02868
10.1021/acscatal.1c02926
10.1002/adma.202302966
10.1039/d2sc06412j
10.1021/acscatal.0c04057

</References>

47. How could transient spin manipulation enable emerging catalytic modes? - What timescales could spin-selective coherent control operate on?

Recent studies have explored how manipulating spin states in catalysts could enable new reactivity pathways and modes of catalysis. Transient spin flipping on ultrafast timescales may allow access to short-lived high energy intermediates and reactive configurations [10.1021/acscatal.1c02926]. For example, the open-shell electronic structure of diazo complexes facilitated olefin generation through rapid single-electron processes [10.1021/acscatal.1c02926]. Several promising potential applications of spin-catalyzed reactions have been proposed, including improving ammonia synthesis, water electrolysis, fuel cells, and pharmaceutical synthesis [10.1002/adma.202302966]. Challenges remain in rationally designing optimal spin states and quantitatively correlating spin modifications to catalytic enhancements [10.1016/S1872-2067(21)63883-4]. However, spin control offers opportunities to overcome kinetic limitations in many catalytic processes.

※※※

Integrative understanding and prospective outlook: Based on recent advances, transient spin manipulation seems poised to enable new modes of catalysis by accessing unique high energy intermediates and reaction pathways. Key next steps are expanding theoretical frameworks to reliably predict optimal spin states and experimental capabilities to precisely control and probe spin dynamics on ultrafast timescales. If transient spin flipping can be synchronized with reaction coordinates, it may allow catalysts to achieve exceptional selectivity and activity. This nascent field is now realizing the promise of spin control principles put forth decades ago. With rapid progress in ultrafast spectroscopy, computational modeling, and synthetic design, spin-catalyzed processes could soon revolutionize chemical production across many industries.

<References>
10.1021/acscatal.1c02926
10.1002/adma.202302966
10.1016/S1872-2067(21)63883-4
</References>

48. How could transient spin manipulation enable emerging catalytic modes? - Which mechanistic pathways are prohibited without spin control?

The literature suggests certain mechanistic pathways like olefin generation may be prohibited without transient spin manipulation to control the open-shell electronic structure [10.1021/acscatal.1c02926]. Spin control likely operates on femtosecond to picosecond timescales, based on spin relaxation dynamics. Coherent spin manipulation on these ultrafast timescales could enable precise tuning of reaction pathways [10.1021/acscatal.0c04057]. Without spin control, many potential reaction mechanisms involving spin-forbidden transitions may be prohibited. Spin state modulation could help overcome these kinetic limitations and expand

possible reaction coordinates [10.1039/d1qi01083b]. Using the proposed descriptor, the study identified optimal compositions like LaCo0.5Fe0.5O3 and La0.75Sr0.25Fe0.75Co0.25O3 with low OER overpotentials below 0.3V. Besides overpotential, surface stability is also important for a robust catalyst design [10.1021/acscatal.2c04424]. Transient spin control could enable new reactivity through ultrashort switching to new spin states and intermediates, operate on femtosecond timescales for coherent tuning of pathways, and overcome forbidden transitions to expand possible reaction mechanisms [10.1016/S1872-2067(21)63883-4]. The quotes indicate that without precision spin control, certain mechanistic pathways like spin-forbidden reactions are likely inaccessible. Spin manipulation could potentially allow more direct routes for some catalytic processes by opening up new pathways [10.1021/jacs.2c12388].

※※※

Integrative understanding and prospective outlook: The emerging field of transient spin-state control offers new possibilities to overcome long-standing limitations and expand possible mechanistic pathways in catalysis. Key opportunities include accessing spin-forbidden intermediates, coherently tuning reaction dynamics, stabilizing reactive species, and redirecting fluxes through competing spin-dependent channels. Harnessing these effects will require advances in ultrafast spectroscopy, quantum control methods, and theoretical modeling of non-adiabatic transitions. An exciting frontier is applying these spin manipulation techniques to enable or optimize industrially relevant catalytic processes through new transient reactivity modes inaccessible under static spin conditions.

<References>
10.1021/acscatal.1c02926
10.1021/acscatal.0c04057
10.1039/d1qi01083b
10.1021/acscatal.2c04424
10.1016/S1872-2067(21)63883-4
10.1021/jacs.2c12388
</References>

49. What are promising horizons for spin-catalysis in real-world applications? - What industrial catalytic processes could benefit from spin manipulation?

Recent studies have explored the emerging field of spin-catalysis, where manipulation of spin states is used to optimize catalytic performance. Control over spin polarization can lead to remarkable selectivity improvements, as demonstrated for the methanol synthesis reaction over copper-containing zeolites [10.1021/acscatal.7b03389]. By tuning the spin density on active oxygen species, the Cu-MOR/MAZ catalyst achieved higher methanol yields compared to the Cu-MFI catalyst. These findings reveal that strategic spin manipulation could overcome kinetic limitations or improve product distributions in other industrially-relevant processes.

Potential industrial applications for spin-catalytic systems include nitrogen fixation, where controlling spin dynamics on synthetic nitrogenase mimics may enable more efficient ammonia synthesis [10.1016/S1872-2067(21)63883-4, 10.1021/acscatal.1c05023]. Spin selectivity could also benefit hydrocarbon conversion processes like Fischer-Tropsch synthesis, alkane

dehydrogenation, and catalytic cracking, where rational tuning of spin polarization may improve activity and selectivity [10.1039/d3ta00555k, 10.1021/acscatal.1c05023]. Additional promising horizons lie in spin-controlled electrocatalysis for renewable energy devices, stereoselective polymerization, and mitigating pollution from large-scale industrial processes [10.1021/jacs.2c12388].

※※※

Integrative understanding and prospective outlook: The emerging field of spin-catalysis presents intriguing opportunities to advance industrial catalytic processes through precise spin control. While computational screening studies have revealed promising theoretical leads, substantial experimental validation is still required to assess real-world viability. Nevertheless, the prospects for mitigating kinetic limitations, enhancing product selectivity, and enabling challenging transformations via transient spin manipulation remain bright. As spectroscopy techniques and spin-polarized model catalysts continue maturing, researchers may uncover economically transformative spin-catalytic processes. Future work should emphasize identifying scalable spin-catalytic systems leveraging earth-abundant materials for sustainable and selective synthesis of valuable chemicals.

<References>
10.1021/acscatal.7b03389
10.1016/S1872-2067(21)63883-4
10.1021/acscatal.1c05023
10.1039/d3ta00555k
10.1021/jacs.2c12388
</References>

50. What are promising horizons for spin-catalysis in real-world applications? - Will spin-polarized currents find uses beyond data storage?

The literature provides some insights into promising horizons for spin-catalysis in real-world applications, though direct discussion is limited. One study suggests iron-based heterometallic cubane complexes like Fe(O)Co3O4 show promise as low-cost, low-toxicity catalysts for alkane hydroxylation reactions, proposing Fe(O)Co3O4 as a potential methane hydroxylation catalyst [10.1021/acscatal.2c03748]. Another work indicates adjacent Mo sites on MoS2 may generate spin-delocalized electrons to facilitate nitrogen activation for applications like ammonia synthesis [10.1016/j.apcatb.2022.122186]. Additional promising horizons proposed include using spin-polarized materials in electrocatalysis and other catalytic processes involving spin-mismatched reactants, as well as a new field of "spintro-biocatalysis" to understand and harness spin-effects in enzyme catalysis [10.1016/j.jcat.2018.03.012]. There are also suggestions that modulating spin and valence states of metals like iron could optimize catalytic performance [10.1039/d3ta00555k]. However, most literature focuses on investigating specific systems rather than broader real-world applications.

Regarding uses of spin-polarized currents, insights are similarly limited. Some works suggest potential applications in spintronics, advanced data storage, sensors, and quantum technologies like computing [10.1039/c5qi00159e, 10.1039/d2sc00737a]. But specifics are lacking, and most

literature concentrates on studying particular materials or reactions over applications. Overall, while promising, spin-catalysis horizons remain largely unexplored, and further research is needed to identify pathways for real-world deployment and synergies with emerging technologies. Critical challenges around materials synthesis, stability, and device integration must also be addressed to realize applications.

※※※

Integrative understanding and prospective outlook: Taking into consideration the advancements and findings discussed in the file, there lies an opportunity to explore emerging fields and innovative methodologies. Future research endeavors might focus on developing design principles and computational models to predictively identify and optimize spin-catalytic materials based on composition, structure, and properties. Machine learning and high-throughput computational screening could accelerate these efforts. On the application front, coupling spin-catalysis with renewable electricity sources could enable sustainable chemical synthesis and fuels production. Quantum spin sensors and control techniques may also help elucidate mechanisms and optimize spin-dependent effects. And biological systems offer inspiration for engineered spin-catalytic complexes and materials. Overall, while foundational work remains, the promising activity enhancements and mechanistic insights from spin-catalysis studies motivate further efforts to translate these discoveries into impactful real-world technologies through interdisciplinary, collaborative research.

<References>
10.1021/acscatal.2c03748
10.1016/j.apcatb.2022.122186
10.1016/j.jcat.2018.03.012
10.1039/d3ta00555k
10.1039/c5qi00159e
10.1039/d2sc00737a
</References>

51. What are promising horizons for spin-catalysis in real-world applications? - How can synergies with quantum technologies accelerate spin-catalysis development?

The literature reported in [10.1021/acscatal.2c00250] focuses on developing a copper-catalyzed method for C(sp3)-N cross-coupling and alkene carboamination reactions, without discussion of spin-catalysis or quantum technologies.

References [10.1021/acscatal.2c03748] and [10.1021/acscatal.3c02095] involve computational modeling of heterometallic complexes and atomic cluster applications in wastewater treatment, respectively, again with no mention of synergies between spin-catalysis and quantum technologies.

The experimental study in [10.1002/anie.202215295] demonstrates graphene belts as spin catalysts but does not extend to promising horizons or synergies with quantum technologies.

Similarly, the computational chemistry investigations reported in [10.1021/acscatal.1c02475, 10.1016/j.apcatb.2023.123289, 10.1002/anie.202300469, 10.1016/j.apcatb.2021.120067, 10.1016/S1872-2067(22)64115-9, 10.1039/d2ta07188f, 10.1039/d0gc01835j,

10.1021/acs.chemrev.8b00046, 10.1039/d2ta06151a, 10.1021/acscatal.1c02251, 10.1016/j.apcatb.2022.122186, 10.1021/acscatal.1c05509, 10.1039/d2ta09276j, 10.1039/d2sc05913d, 10.1021/acscatal.0c03535, 10.1021/acscatal.1c00811, 10.1016/j.fuel.2019.115845, 10.1021/acscatal.1c03432, 10.1016/j.memsci.2017.12.032, 10.1039/d2sc00737a, 10.1016/j.jcat.2022.08.019, 10.1039/d3ta03271j, 10.1016/j.apcatb.2022.122202, 10.1016/j.cej.2020.127634, 10.1021/acscatal.8b00012, 10.1002/adma.202302966, 10.1039/d0sc04114a, 10.1021/acscatal.1c02165, 10.1021/acscatal.7b04401, 10.1002/adma.201903491, 10.1021/acscatal.1c05877, 10.1002/anie.202301128, 10.1021/jacs.1c02232, 10.1016/j.jcat.2018.11.029, 10.1039/c5qi00159e, 10.1021/acscatal.0c01610, 10.1021/jacs.3c06285, 10.1002/adma.202007525, 10.1021/acscatal.0c02010, 10.1016/j.scib.2023.07.049, 10.1021/jacs.2c12388, 10.1021/acscatal.7b03389, 10.1016/j.jcat.2018.03.012, 10.1039/d0sc03777j, 10.1021/acscatal.8b00505, 10.1016/j.ccr.2018.02.001, 10.1021/acscatal.7b03529, 10.1016/j.jcat.2019.06.010, 10.1016/j.cej.2020.124371, 10.1021/acscatal.1c03350, 10.1002/adma.202202240, 10.1021/jacs.8b13701 and 10.1039/d3ta00555k] advance the mechanistic understanding of various catalytic systems but do not extend to promising horizons, synergies with quantum technologies, or acceleration of spin-catalysis development.

※※※

Integrative understanding and prospective outlook: While the current literature is narrowly focused, there appears to be significant potential in bridging spin-catalysis and quantum technologies. Advances in quantum sensing, computing, and materials design could enable transformative capabilities like probing spin dynamics during reactions, accurately modeling complex spin interactions, and engineering materials that leverage quantum effects. An interdisciplinary approach combining expertise in spin chemistry and quantum information science is needed to realize these opportunities. By elucidating spin-dependent mechanisms and leveraging quantum control techniques, next-generation spin-catalytic systems might be designed for applications from renewable energy to quantum computing. The horizons look promising for spin-catalysis accelerated by quantum technologies.

<References>

10.1021/acscatal.2c00250
10.1021/acscatal.2c03748
10.1021/acscatal.3c02095
10.1002/anie.202215295
10.1021/acscatal.1c02475
10.1016/j.apcatb.2023.123289
10.1002/anie.202300469
10.1016/j.apcatb.2021.120067
10.1016/S1872-2067(22)64115-9
10.1039/d2ta07188f
10.1039/d0gc01835j
10.1021/acs.chemrev.8b00046
10.1039/d2ta06151a
10.1021/acscatal.1c02251
10.1016/j.apcatb.2022.122186

10.1021/acscatal.1c05509
10.1039/d2ta09276j
10.1039/d2sc05913d
10.1021/acscatal.0c03535
10.1021/acscatal.1c00811
10.1016/j.fuel.2019.115845
10.1021/acscatal.1c03432
10.1016/j.memsci.2017.12.032
10.1039/d2sc00737a
10.1016/j.jcat.2022.08.019
10.1039/d3ta03271j
10.1016/j.apcatb.2022.122202
10.1016/j.cej.2020.127634
10.1021/acscatal.8b00012
10.1002/adma.202302966
10.1039/d0sc04114a
10.1021/acscatal.1c02165
10.1021/acscatal.7b04401
10.1002/adma.201903491
10.1021/acscatal.1c05877
10.1002/anie.202301128
10.1021/jacs.1c02232
10.1016/j.jcat.2018.11.029
10.1039/c5qi00159e
10.1021/acscatal.0c01610
10.1021/jacs.3c06285
10.1002/adma.202007525
10.1021/acscatal.0c02010
10.1016/j.scib.2023.07.049
10.1021/jacs.2c12388
10.1021/acscatal.7b03389
10.1016/j.jcat.2018.03.012
10.1039/d0sc03777j
10.1021/acscatal.8b00505
10.1016/j.ccr.2018.02.001
10.1021/acscatal.7b03529
10.1016/j.jcat.2019.06.010
10.1016/j.cej.2020.124371
10.1021/acscatal.1c03350
10.1002/adma.202202240
10.1021/jacs.8b13701
10.1039/d3ta00555k
</References>

Solid-Liquid Interfaces in Aqueous-Phase Heterogeneous Catalysis: From Molecular Understanding to Sustainable Applications

Abstract: This comprehensive review examines the critical role of solid-liquid interfaces in aqueous-phase heterogeneous catalysis, highlighting their fundamental importance in advancing sustainable chemical processes. We trace the evolution of interface studies from early observations to modern molecular-level investigations, emphasizing how water and other solvents influence catalytic activity through complex interfacial phenomena. The review systematically explores physical and chemical principles governing these interfaces, including surface energy, electric double layer structure, and molecular-level interactions such as water ordering and hydrogen bonding networks. Recent advances in characterization methods, combining in-situ spectroscopic techniques with computational approaches, have provided unprecedented insights into interfacial dynamics and reaction mechanisms. These fundamental understandings have enabled significant progress in industrial applications, particularly in sustainable chemistry and energy technologies. We also discuss emerging opportunities and challenges, emphasizing the need for continued innovation in catalyst design and characterization methods. This review provides a framework for understanding how interfacial phenomena impact catalytic performance, guiding future developments in aqueous-phase heterogeneous catalysis for more sustainable chemical processes.
Keywords:
Solid-liquid interfaces; Aqueous-phase catalysis; Surface chemistry; Zeolite catalysts; In-situ spectroscopy; Water ordering; Interface dynamics; Structure-activity relationships

1. Introduction: Evolution and Significance of Solid-Liquid Interface Studies in Catalysis -> Historical Development: Early observations of interface effects in catalysis

Historically, the focus in heterogeneous catalysis was primarily on solid-gas interfaces due to the importance of volatile compounds in chemical transformations. This emphasis led to a rich understanding of catalytic and surface chemistries at these interfaces [10.1016/s1872-2067(23)64550-4]. However, less attention was initially placed on solid-liquid interfaces, which are crucial for processes like upgrading bio-based compounds, producing pharmaceutical molecules, and (photo)electrochemistries that generate renewable energy carriers and chemicals.

Early studies on interface effects in catalysis highlighted that phenomena such as adsorption, nucleation, corrosion, and catalysis are ubiquitous in various chemical systems, linking different sub-fields of chemical science. Water molecules were observed to either molecularly or dissociatively adsorb on catalyst surfaces under wet conditions, leading to alterations in active sites for metal catalysts. This hydroxylation and hydration influenced the catalytic performance of structure-dependent reactions [10.1021/acs.jpcc.7b10208].

The chemistry at water/solid interfaces is ubiquitous and plays an important role in everyday phenomena and scientific and technological processes, such as corrosion, biomineralization, wet heterogeneous catalysis, fuel cells, lubricants, environmental chemistry, and photochemical water splitting. Water can act as a cocatalyst by providing or stabilizing highly reactive species and

enhancing reactivity and selectivity in catalytic reactions. Additionally, water may participate as a hydrogen donor to enhance reactivity and selectivity [10.1021/acscatal.4c02514].

※※※

Integrative understanding and prospective outlook: Considering the advancements and findings discussed in the file, there lies an opportunity to explore emerging fields and innovative methodologies. Future research endeavors might focus on elucidating the detailed mechanisms of water-surface interactions, especially in the context of developing sustainable processes like biomass upgrading and (photo)electrochemistries. Understanding the microscale aqueous environment on the surface of heterogeneous catalysts could lead to the design of more efficient and selective catalysts for greener chemical transformations. Furthermore, addressing the open questions about water structuring, dissociation at the interface, and the impact of electrochemical potential on interfacial structure will be pivotal for advancing the field [10.1021/acscatal.2c00594].

<References>
10.1016/s1872-2067(23)64550-4
10.1021/acs.jpcc.7b10208
10.1021/acscatal.4c02514
10.1021/acscatal.2c00594
</References>

2. Introduction: Evolution and Significance of Solid-Liquid Interface Studies in Catalysis -> Historical Development: Development of surface science techniques

The evolution of surface science techniques from solid-gas interfaces to solid-liquid interfaces has significantly advanced our understanding of catalysis at complex interfaces. Initially, surface science was primarily concerned with solid-gas interactions; however, as researchers recognized the critical role of aqueous environments in catalytic processes, these methods were adapted and expanded [10.1021/acs.jpcc.7b10208]. Techniques such as density functional theory (DFT) calculations and ab initio molecular dynamics (AIMD) simulations have been instrumental in elucidating the atomic-level mechanisms at solid-liquid interfaces.

With the advent of ab initio simulation methods and surface imaging technologies like scanning tunneling microscopy (STM), substantial progress has been made in understanding water behavior at interfaces under both vacuum and ambient conditions [10.1021/acscatal.4c02514]. These tools have revealed that water can adsorb on solid surfaces in various forms, ranging from one-dimensional chains to three-dimensional clusters, depending on the nature of the solid surface.

Despite initial challenges in applying traditional surface science techniques to solid-liquid interfaces, significant advancements have been achieved. Methods like STM, vibrational, and X-ray spectroscopy provided valuable insights into water-surface interactions under low-pressure conditions. However, they were limited to studying only a few layers of water at low temperatures. To overcome these limitations, alternative methods such as calorimetry, inelastic neutron scattering, nonlinear optical spectroscopies, and surface-sensitive X-ray diffraction methods have been developed [10.1021/acscatal.2c00594].

Over time, surface science techniques have evolved to study solid-liquid interfaces more comprehensively. Early research up to the 1990s provided insights into phenomena like the formation of H3O+ from H* ionization at certain metal surfaces. Recent studies have leveraged advanced characterization techniques and theoretical methods to explore the chemical and mechanistic origins of water effects in catalytic systems [10.1016/s1872-2067(23)64550-4].

※※※

Integrative understanding and prospective outlook: The continuous development and adaptation of surface science techniques have revolutionized our ability to probe and understand the complexities of solid-liquid interfaces in catalysis. Future research should focus on integrating multi-scale modeling approaches with experimental data to provide a holistic view of catalytic processes. Additionally, there is an opportunity to develop real-time, in situ characterization methods that can capture dynamic changes at solid-liquid interfaces during catalytic reactions. This could lead to the design of more efficient and robust catalysts for applications in energy conversion and environmental remediation.

<References>
10.1021/acs.jpcc.7b10208
10.1021/acscatal.4c02514
10.1021/acscatal.2c00594
10.1016/s1872-2067(23)64550-4
</References>

3. Introduction: Evolution and Significance of Solid-Liquid Interface Studies in Catalysis -> Historical Development: Emergence of in-situ characterization methods

The evolution of in-situ characterization methods has significantly influenced the study of solid-liquid interfaces in catalysis. Initially, these methods were primarily applied to observe catalysts under reaction conditions in gas-phase systems. However, with the increasing importance of liquid-phase reactions, these techniques have been adapted to investigate solid-liquid interfaces [10.1016/s1872-2067(23)64550-4]. Innovations such as operando spectroscopy and microscopy now provide real-time insights into catalytic processes at these interfaces. Advances in instrumentation and computational power have enabled real-time tracking of structural and electronic changes at solid-liquid interfaces during catalytic processes, offering deeper understanding of dynamic behavior [10.1021/acs.jpcc.7b10208].

Techniques like in situ X-ray absorption spectroscopy (XAS) and X-ray absorption near edge structures (XANES) have been instrumental in distinguishing changes in the BAS site as water concentration increases. Experimental evidence shows that proton transfer occurs when at least two water molecules are present at the BAS site [10.1021/acs.jpcc.0c07033].

※※※

Integrative understanding and prospective outlook: Considering the advancements discussed, there is a significant opportunity to explore emerging fields and innovative methodologies. Future research might focus on integrating advanced in-situ techniques with machine learning algorithms to predict and optimize catalytic performance. Additionally, expanding the scope to include more

complex systems could yield novel insights into interfacial phenomena, potentially leading to the design of more efficient catalysts for propane dehydrogenation.
<References>
10.1016/s1872-2067(23)64550-4
10.1021/acs.jpcc.7b10208
10.1021/acs.jpcc.0c07033
</References>

The evolution and significance of solid-liquid interface studies in catalysis have been pivotal in advancing our understanding of propane dehydrogenation catalysts. Initially, in-situ characterization methods were primarily applied to observe catalysts under reaction conditions in gas-phase systems [10.1016/s1872-2067(23)64550-4]. However, with the growing importance of liquid-phase reactions, these techniques have been adapted to study solid-liquid interfaces. Innovations such as operando spectroscopy and microscopy now provide real-time insights into catalytic processes at these interfaces, enabling researchers to track structural and electronic changes dynamically during catalysis [10.1021/acs.jpcc.7b10208].

Specific advancements like in situ X-ray absorption spectroscopy and X-ray absorption near edge structures (XANES) have been crucial for distinguishing changes in the BAS site as water concentration increases. Experimental evidence has shown that proton transfer occurs when at least two water molecules are present at the BAS site [10.1021/acs.jpcc.0c07033]. These developments highlight the critical role of in-situ characterization methods in elucidating the dynamic behavior of catalysts under reaction conditions.

※※※

Integrative understanding and prospective outlook: Considering the advancements and findings discussed in the file, there lies an opportunity to explore emerging fields and innovative methodologies. Future research endeavors might focus on integrating machine learning algorithms with in-situ characterization techniques to predict catalyst performance more accurately. Additionally, developing new in-situ tools that can probe deeper into the molecular mechanisms of catalytic reactions will be essential. This could lead to the design of more efficient and selective catalysts for propane dehydrogenation, ultimately enhancing industrial processes.
<References>
10.1016/s1872-2067(23)64550-4
10.1021/acs.jpcc.7b10208
10.1021/acs.jpcc.0c07033
</References>

In-situ characterization methods have evolved significantly over the years, transitioning from static observation techniques to dynamic monitoring approaches. Initially, these methods were primarily used to observe catalysts under reaction conditions in gas-phase systems. However, with the increasing importance of liquid-phase reactions, these techniques have been adapted to study solid-liquid interfaces. Innovations such as operando spectroscopy and microscopy now provide real-time insights into catalytic processes at these interfaces [10.1016/s1872-2067(23)64550-4].

Advances in instrumentation and computational power have enabled the real-time tracking of structural and electronic changes at solid-liquid interfaces during catalytic processes, offering deeper insights into the dynamic behavior of catalysts under reaction conditions [10.1021/acs.jpcc.7b10208].

Specific techniques like in situ X-ray absorption spectroscopy (XAS) and X-ray absorption near-edge structure (XANES) have been utilized to distinguish changes in the BAS site as water concentration increases. Experimental evidence shows that proton transfer occurs when at least two water molecules are present at the BAS site [10.1021/acs.jpcc.0c07033].

※※※

Integrative understanding and prospective outlook: The advancements in in-situ characterization methods have provided unprecedented molecular-level insights into interfacial chemical processes. Future research could focus on further integrating these methods with advanced theoretical models to predict and control catalytic behavior more effectively. Moreover, expanding the application of operando techniques to a broader range of catalytic systems, especially those involving complex multiphase environments, could uncover new mechanisms and enhance catalyst design. The continuous evolution of these methods promises to revolutionize our understanding of catalysis at solid-liquid interfaces.

<References>
10.1016/s1872-2067(23)64550-4
10.1021/acs.jpcc.7b10208
10.1021/acs.jpcc.0c07033
</References>

4. Introduction: Evolution and Significance of Solid-Liquid Interface Studies in Catalysis -> Historical Development: Role of computational modeling advances

The evolution and significance of solid-liquid interface studies in catalysis have been profoundly influenced by advances in computational modeling. Computational tools, such as density functional theory (DFT) and ab initio molecular dynamics (AIMD) simulations, have provided valuable insights into the complex interactions at these interfaces [10.1021/acs.jpcc.7b10208]. These models allow researchers to predict surface structures under various conditions, analyze the stability and reactivity of supported metal nanoclusters, and explore mechanisms like proton transfer and water dissociation [10.1021/acscatal.4c02514].

Over the past two decades, computational catalysis has emerged as an essential tool for understanding catalytic reactions and designing new industrially relevant catalytic materials [10.1021/acscatal.7b04367]. However, progress in theoretical studies of solvent effects in heterogeneous catalysis remains limited, particularly in vapor-phase catalysis [10.1021/acscatal.7b04367]. To address this, several approaches have been proposed to accelerate the computation of solvation effects on reaction and activation free energies at solid-liquid interfaces [10.1021/acscatal.7b04367]. Among these, QM/MM models are deemed the most appropriate for balancing computational expense and chemical accuracy [10.1021/acscatal.7b04367].

Furthermore, advances in computational resources and theoretical methods over the past decade have enabled more advanced ab initio treatments of realistic surface models, providing deeper insights into the surface chemistry at the aqueous/metal interface [10.1016/j.cossms.2006.03.007]. In electrocatalysis, first-principles DFT calculations have helped elucidate molecular mechanisms involved in electrochemical reactions at solid-liquid interfaces, sometimes accelerating the development of improved electrode materials [10.1021/acs.jpcc.3c06870].

Implicit solvent models or polarizable continuum models (PCM) have become increasingly popular since 2014, aiding in converting experimental data into atomistic insights about aqueous interfaces involved in catalysis [10.1021/acscatal.2c00594]. Modeling is now essential for understanding solid-liquid interfaces, with computational chemistry serving as a cornerstone to elucidate the atomistic mechanisms of complicated chemical reactions at these interfaces [10.1021/acs.jpclett.3c02233].

※※※

Integrative understanding and prospective outlook: Considering the advancements and findings discussed, there is a clear opportunity to bridge the gap between vapor-phase and liquid-phase catalysis through innovative computational methodologies. Future research could focus on developing hybrid models that integrate QM/MM and PCM approaches to better capture solvation effects. Additionally, leveraging machine learning techniques alongside computational chemistry can enhance the prediction and design of novel catalysts. This integrative approach promises to provide a comprehensive understanding of solid-liquid interfaces, paving the way for breakthroughs in catalytic processes and sustainable energy technologies.

<References>
10.1021/acs.jpcc.7b10208
10.1021/acscatal.4c02514
10.1021/acscatal.7b04367
10.1016/j.cossms.2006.03.007
10.1021/acs.jpcc.3c06870
10.1021/acscatal.2c00594
10.1021/acs.jpclett.3c02233
</References>

The evolution and significance of solid-liquid interface studies in catalysis have been profoundly influenced by advances in computational modeling. Initially, theoretical studies were primarily focused on vapor-phase catalysis, with limited progress in understanding solvent effects in heterogeneous catalysis [10.1021/acscatal.7b04367]. However, over the past two decades, computational catalysis has emerged as an indispensable tool for elucidating catalytic reactions and designing industrially relevant catalytic materials [10.1021/acscatal.7b04367].

Computational methods such as density functional theory (DFT) and ab initio molecular dynamics (AIMD) simulations have provided valuable insights into the detailed structures of water at solid-liquid interfaces [10.1021/acs.jpcc.0c07033]. These models enable researchers to explore complex interactions between water molecules and solid surfaces, predict surface

structures under various conditions, and analyze the stability and reactivity of supported metal nanoclusters [10.1021/acs.jpcc.7b10208].

Furthermore, significant advancements in computational resources and theoretical methods have allowed for more advanced ab initio treatments of realistic surface models, providing deeper insights into the surface chemistry at the aqueous/metal interface [10.1016/j.cossms.2006.03.007]. In particular, QM/MM models are currently deemed the most appropriate for predicting solvation effects in heterogeneous catalysis, balancing computational expense and chemical accuracy [10.1021/acscatal.7b04367].

Additionally, implicit solvent models or polarizable continuum models (PCM), which gained popularity since 2014, have become essential for converting experimental data into atomistic insights about aqueous interfaces involved in catalysis [10.1021/acscatal.2c00594]. First-principles DFT calculations have also emerged as powerful tools that have accelerated the development of improved electrode materials by elucidating the underlying molecular mechanisms of electrocatalysis [10.1021/acs.jpcc.3c06870].

※※※

Integrative understanding and prospective outlook: Considering the advancements and findings discussed in the file, there lies an opportunity to explore emerging fields and innovative methodologies. Future research endeavors might focus on integrating machine learning techniques with computational models to enhance the prediction and design of catalysts. Moreover, developing more sophisticated models that accurately account for solvation effects in liquid-solid interfaces could significantly advance our understanding of catalytic processes. The continuous improvement in computational power and algorithms will likely lead to breakthroughs in both fundamental science and industrial applications.

<References>
10.1021/acscatal.7b04367
10.1021/acs.jpcc.0c07033
10.1021/acs.jpcc.7b10208
10.1016/j.cossms.2006.03.007
10.1021/acscatal.2c00594
10.1021/acs.jpcc.3c06870
</References>

The evolution and significance of solid-liquid interface studies in catalysis have been profoundly influenced by advances in computational modeling. Computational tools, such as density functional theory (DFT) and ab initio molecular dynamics (AIMD) simulations, have provided valuable insights into the complex interactions at these interfaces [10.1021/acs.jpcc.7b10208]. These models allow researchers to predict surface structures under various conditions, analyze the stability and reactivity of supported metal nanoclusters, and explore mechanisms like proton transfer and water dissociation [10.1021/acscatal.4c02514].

Over the past two decades, computational catalysis has emerged as an essential tool for understanding catalytic reactions and designing new industrially relevant catalytic materials [10.1021/acscatal.7b04367]. However, progress in theoretical studies of solvent effects in heterogeneous catalysis remains limited, particularly in vapor-phase catalysis

[10.1021/acscatal.7b04367]. To address this, several approaches have been proposed to accelerate the computation of solvation effects on reaction and activation free energies at solid-liquid interfaces [10.1021/acscatal.7b04367]. Among these, QM/MM models are deemed the most appropriate for balancing computational expense and chemical accuracy [10.1021/acscatal.7b04367].

Furthermore, advances in computational resources and theoretical methods over the past decade have enabled more advanced ab initio treatments of realistic surface models, providing deeper insights into the surface chemistry at the aqueous/metal interface [10.1016/j.cossms.2006.03.007]. In electrocatalysis, first-principles DFT calculations have helped elucidate molecular mechanisms involved in electrochemical reactions at solid-liquid interfaces, sometimes accelerating the development of improved electrode materials [10.1021/acs.jpcc.3c06870].

Implicit solvent models or polarizable continuum models (PCM) have become increasingly popular since 2014, aiding in converting experimental data into atomistic insights about aqueous interfaces involved in catalysis [10.1021/acscatal.2c00594]. Modeling is now essential for understanding solid-liquid interfaces, with computational chemistry serving as a cornerstone to elucidate the atomistic mechanisms of complicated chemical reactions at these interfaces [10.1021/acs.jpclett.3c02233].

※※※

Integrative understanding and prospective outlook: Considering the advancements and findings discussed, there is a clear opportunity to bridge the gap between vapor-phase and liquid-phase catalysis through innovative computational methodologies. Future research could focus on developing hybrid models that integrate QM/MM and PCM approaches to better capture solvation effects. Additionally, leveraging machine learning techniques alongside computational chemistry can enhance the prediction and design of novel catalysts. This integrative approach promises to provide a comprehensive understanding of solid-liquid interfaces, paving the way for breakthroughs in catalytic processes and sustainable energy technologies.

<References>
10.1021/acs.jpcc.7b10208
10.1021/acscatal.4c02514
10.1021/acscatal.7b04367
10.1016/j.cossms.2006.03.007
10.1021/acs.jpcc.3c06870
10.1021/acscatal.2c00594
10.1021/acs.jpclett.3c02233
</References>

5. Introduction: Evolution and Significance of Solid-Liquid Interface Studies in Catalysis -> Current Significance: Impact on chemical manufacturing

Solid-liquid interface studies significantly impact chemical manufacturing processes, particularly in propane dehydrogenation. These studies enhance catalyst design and performance by understanding the interactions between water and catalyst surfaces, leading to more efficient

and selective catalysts that improve productivity and reduce waste [10.1021/acs.jpcc.7b10208]. Processing at relatively low temperatures in the condensed phase minimizes undesirable thermal degradation reactions, increases product selectivity, and facilitates product separation from excess water due to reduced hydrophilicity of reaction products compared to feedstocks [10.1021/acscatal.7b04367].

Aqueous-phase heterogeneous catalysis, influenced by solid-liquid interface studies, has numerous applications, including biomass reforming, Fischer-Tropsch synthesis, and electrocatalysis [10.1021/acs.jctc.9b01249]. Moreover, these studies significantly impact the production of fuels and chemicals by enhancing the performance of heterogeneous catalysts and creating novel catalytic conversion routes [10.1021/jacsau.1c00319]. Water dissociation at the metal-support interface promotes metal/support interactions, influencing the reactivity and selectivity of catalysts, which optimizes catalytic reactions and alters adsorption behaviors of reactants [10.1021/acscatal.4c02514].

Solid-liquid interface studies also play a crucial role in developing sustainable and renewable energy devices like fuel cells and batteries, contributing to environmentally friendly processes for removing and converting toxic substances such as denitrification [10.1021/acs.jpcc.3c06870]. Furthermore, they are central to sustainable processes like biomass upgrading, photo-, and electrocatalysis, addressing key questions related to water structuring, dissociation, and electrochemical potentials [10.1021/acscatal.2c00594]. In addition, these studies address challenges in plating, corrosion, batteries, fuel cells, and catalysts, advancing sustainable and green chemistries to solve energy, resource, and environmental issues [10.1021/acs.jpclett.3c02233].

Understanding solid-liquid interfaces is crucial for developing catalysts with improved energy efficiencies, reduced consumption of non-renewable feedstocks, and minimized environmental impact. This knowledge helps optimize catalytic reactions in aqueous solutions, leading to greener and more sustainable industrial practices [10.1016/s1872-2067(23)64550-4].

※※※

Integrative understanding and prospective outlook: Considering the advancements and findings discussed in the file, there lies an opportunity to explore emerging fields and innovative methodologies. Future research endeavors might focus on integrating advanced characterization techniques to better understand the molecular-level interactions at the solid-liquid interface. Additionally, developing new catalyst materials that can operate efficiently under aqueous conditions while maintaining high selectivity and stability will be critical. The exploration of bi-functional catalysts that combine the advantages of both solid-gas and solid-liquid interfaces could lead to breakthroughs in process intensification and energy efficiency. Finally, leveraging computational modeling to predict and design optimal catalyst structures will pave the way for smarter and more sustainable chemical manufacturing processes.

<References>

10.1021/acs.jpcc.7b10208
10.1021/acscatal.7b04367
10.1021/acs.jctc.9b01249
10.1021/jacsau.1c00319
10.1021/acscatal.4c02514
10.1021/acs.jpcc.3c06870

10.1021/acscatal.2c00594
10.1021/acs.jpclett.3c02233
10.1016/s1872-2067(23)64550-4
</References>

6. Introduction: Evolution and Significance of Solid-Liquid Interface Studies in Catalysis -> Current Significance: Environmental applications

Solid-liquid interfaces have evolved significantly in catalysis, playing a critical role in environmental applications such as wastewater treatment and pollutant removal. Catalysts designed for these interfaces can effectively degrade organic pollutants and convert harmful substances into less toxic forms, contributing to cleaner water and air [10.1021/acs.jpcc.7b10208]. Additionally, solid-liquid interfaces are essential for processes like carbon capture and utilization, which help mitigate climate change by reducing CO2 emissions [10.1016/s1872-2067(23)64550-4].

Furthermore, solid-liquid interfaces enable the processing of bio-based raw materials in water or water-containing solvent mixtures, minimizing the use of organic solvents and reducing environmental impact [10.1016/s1872-2067(23)64550-4]. Water's high boiling point and low volatility ensure that polyols and sugars do not decompose during processing, thereby lowering energy input and maintaining feedstock integrity. Moreover, raw feedstocks containing water can be directly processed in aqueous environments, enhancing solubility and miscibility of platform surrogates with water.

The significance of solid-liquid interfaces extends to sustainable and green chemistries aimed at addressing energy, resource, and environmental issues [10.1021/acs.jpclett.3c02233]. These interfaces also play a crucial role in environmentally friendly removal and conversion of toxic substances, including denitrification processes [10.1021/acs.jpcc.3c06870]. They influence material stability and reactivity in environmental remediation and protection [10.1021/acscatal.4c02514].

※※※

Integrative understanding and prospective outlook: Considering the advancements and findings discussed in the file, there lies an opportunity to explore emerging fields and innovative methodologies. Future research endeavors might focus on developing more efficient catalysts tailored for solid-liquid interfaces to enhance pollutant degradation and pollutant conversion efficiency. Additionally, integrating renewable energy sources with solid-liquid interface technologies could further reduce the environmental footprint. Exploring novel bio-based raw materials and optimizing their processing in aqueous environments will also pave the way for sustainable chemical processing. Ultimately, the synergy between solid-liquid interfaces and green chemistry principles holds immense potential for solving pressing environmental challenges.

<References>
10.1021/acs.jpcc.7b10208
10.1016/s1872-2067(23)64550-4
10.1021/acs.jpclett.3c02233
10.1021/acs.jpcc.3c06870

10.1021/acscatal.4c02514
</References>

7. Introduction: Evolution and Significance of Solid-Liquid Interface Studies in Catalysis -> Current Significance: Energy conversion technologies

The evolution and significance of solid-liquid interface studies in catalysis have profoundly influenced energy conversion technologies. Solid-liquid interfaces are vital for processes like hydrogen production, fuel cells, and electrochemical reactions [10.1021/acs.jpcc.7b10208]. These interfaces facilitate the conversion of chemical energy into electrical energy, enhancing the efficiency and sustainability of energy systems. In particular, aqueous-phase heterogeneous catalysis has many applications, including electrocatalysis [10.1021/acs.jctc.9b01249], where the unique reaction environment created by the interface between an aqueous solution and a metal surface significantly influences molecular reactivity [10.1016/j.cossms.2006.03.007]. This environment allows electrocatalytic systems to adopt benefits from both heterogeneous and homogeneous catalytic systems, driving theoretical interest and practical advancements.

Furthermore, solid-liquid interfaces play a crucial role in aqueous-phase reactions, which are pivotal for upgrading small molecules like methane. These reactions enable the production of value-added chemicals from inexpensive feedstocks, a process that is challenging or unfeasible in gas-phase reactions [10.1021/jacsau.1c00319]. Additionally, these interfaces enhance the performance of catalysts in fuel cells and photochemical water splitting, facilitating the efficient transport of hydrogen and protons, thereby improving overall energy conversion efficiency [10.1021/acscatal.4c02514].

Heterogeneous electrocatalysis, which involves electrochemical reactions at solid-liquid interfaces, is central to sustainable and renewable energy devices like fuel cells and batteries [10.1021/acs.jpcc.3c06870]. Surface chemistry at the solid-liquid interface has been industrially relevant in various applications, including batteries and fuel cells, and its role is increasingly important in sustainable and green chemistries to address energy-related challenges [10.1021/acs.jpclett.3c02233].

※※※

Integrative understanding and prospective outlook: Considering the advancements and findings discussed in the file, there lies an opportunity to explore emerging fields and innovative methodologies. Future research endeavors might focus on developing novel materials with tailored properties at the solid-liquid interface to enhance catalytic efficiency and selectivity. The integration of advanced characterization techniques could provide deeper insights into the fundamental mechanisms governing these interfaces, paving the way for more efficient and sustainable energy conversion systems. Moreover, the exploration of new reaction pathways, especially those involving aqueous-phase reactions, could unlock the potential for producing high-value chemicals from abundant resources, aligning with the goals of sustainable and green chemistries.

<References>
10.1021/acs.jpcc.7b10208
10.1021/acs.jctc.9b01249

10.1016/j.cossms.2006.03.007
10.1021/jacsau.1c00319
10.1021/acscatal.4c02514
10.1021/acs.jpcc.3c06870
10.1021/acs.jpclett.3c02233
</References>

8. Introduction: Evolution and Significance of Solid-Liquid Interface Studies in Catalysis -> Current Significance: Sustainable chemistry initiatives

Solid-liquid interfaces have emerged as pivotal platforms in the evolution of catalysis, significantly contributing to sustainable chemistry initiatives. These interfaces facilitate reactions that can occur in aqueous media, which is more environmentally friendly compared to organic solvents [10.1016/j.jcat.2011.01.013]. This approach not only reduces the environmental impact but also facilitates the use of water as a solvent, an abundant and green alternative. Moreover, solid catalysts used in these interfaces can be easily separated and reused, thereby minimizing waste and enhancing sustainability.

Solid-liquid interfaces are pivotal in the transformation of nonfood biomass into renewable chemicals and fuels, addressing political, economic, social, and environmental concerns associated with nonrenewable carbon resources [10.1021/acscatal.6b02532]. These interfaces support environmentally friendly and energy-efficient liquid-phase reactions, often requiring lower temperatures and pressures, thus reducing energy consumption and operational costs. Additionally, they enable the design of efficient and robust catalysts that can operate under realistic conditions involving complex structures, elevated temperatures, and pressures [10.1039/c4cy01720j].

The development of catalytic processes at solid-liquid interfaces has been stimulated by the growing interest in conducting reactions in aqueous media. Reactions at the water/solid interface are central to developing sustainable processes from biomass upgrading to photo- and electrocatalysis [10.1021/acscatal.2c00594]. Understanding the behavior of water and organic molecules at these interfaces is crucial for optimizing catalytic processes in aqueous environments, supporting the advancement of green chemical technologies [10.1021/acs.jpcc.9b07738].

※※※

Integrative understanding and prospective outlook: Considering the advancements and findings discussed in the file, there lies an opportunity to explore emerging fields and innovative methodologies. Future research endeavors might focus on the integration of solid-liquid interfaces with renewable energy sources, such as solar or wind power, to further enhance the efficiency and sustainability of catalytic processes. Additionally, developing new characterization methods for these interfaces will be essential for uncovering reaction mechanisms and active-site requirements, leading to more effective and selective catalysts. The continued exploration of biomass conversion using solid-liquid interfaces holds promise for reducing dependency on nonrenewable resources and promoting a circular economy.

<References>

10.1016/j.jcat.2011.01.013
10.1021/acscatal.6b02532
10.1039/c4cy01720j
10.1021/acscatal.2c00594
10.1021/acs.jpcc.9b07738
</References>

9. Fundamental Principles of Solid-Liquid Interfaces -> Physical Chemistry of Interfaces: Surface energy and wetting

Surface energy and wetting phenomena at solid-liquid interfaces play a critical role in determining the efficiency and selectivity of catalytic processes, particularly in propane dehydrogenation. High surface energy promotes better wetting, leading to enhanced adhesion and reactivity between the solid catalyst and liquid reactants [10.1021/acscatal.6b02532]. This improved wetting behavior significantly impacts processes such as adhesion, coating, and cleaning, which are vital for industrial applications like catalysis and material synthesis [10.1016/j.jcat.2011.01.013].

Wetting properties determine the extent to which a liquid spreads over a solid surface, influencing the accessibility of active sites and overall reaction efficiency. For instance, solvent exposure to active sites can either enhance or inhibit reactions depending on the nature of the catalyst. Additionally, the high concentration of solvent molecules can impede the diffusion of reactants and products within nanoporous materials [10.1038/s41467-020-14860-6].

The interaction between water and solid surfaces is particularly significant in aqueous-phase reactions. Water molecules cluster around Brønsted acid sites within zeolite pores, impacting dehydration processes and altering the energetic landscape of the interface [10.1021/acs.jpcc.1c03846]. This interaction can facilitate hydrolysis and influence the stability and reactivity of materials like zeolites. Hydrophobic surfaces prevent water from entering the pores, maintaining structural integrity, while hydrophilic surfaces may facilitate water adsorption, leading to potential structural collapse [10.1134/s0965544120040143].

Moreover, the presence of water molecules can modify thermodynamic and kinetic quantities, alter dominant reaction states, and participate in the mechanism via site blocking or mediating hydrogen transfer [10.1016/j.jcat.2024.115562]. Adsorption of water on surfaces like TiO2 can lower Gibbs surface free energy, enhancing stability, while interactions between liquid water and oxide surfaces can lead to partial surface hydroxylation [10.1080/08927022.2022.2049774].

※※※

Integrative understanding and prospective outlook: Considering the advancements and findings discussed in the file, there lies an opportunity to explore emerging fields and innovative methodologies. Future research endeavors might focus on developing novel catalysts with tailored surface energies and wetting properties to optimize propane dehydrogenation efficiency. Investigating the interplay between surface chemistry and wetting behavior could lead to breakthroughs in designing robust catalysts that maintain their activity under harsh conditions.

Additionally, exploring the role of hydrophobic functionalization to enhance water tolerance of hydrophilic catalysts could pave the way for more stable and efficient catalytic systems.

<References>
10.1021/acscatal.6b02532
10.1016/j.jcat.2011.01.013
10.1038/s41467-020-14860-6
10.1021/acs.jpcc.1c03846
10.1134/s0965544120040143
10.1016/j.jcat.2024.115562
10.1080/08927022.2022.2049774
</References>

10. Fundamental Principles of Solid-Liquid Interfaces -> Physical Chemistry of Interfaces: Electric double layer structure

The electric double layer (EDL) at solid-liquid interfaces is a fundamental concept in understanding electrochemical processes. It consists of two regions: the inner Helmholtz plane (or Stern layer) and the outer diffuse layer [10.1021/acscatal.6b02532]. The inner layer comprises ions or molecules adsorbed onto the solid surface, while the outer layer contains counterions in the liquid phase. This structure plays a crucial role in charge distribution, influencing ion mobility and surface charge effects, thereby affecting various interfacial phenomena such as corrosion, electrodeposition, and colloid stability [10.1039/c4cy01720j].

The point of zero charge (PZC), where the net charge on the surface becomes zero, is pivotal for understanding how charges distribute at the interface and influence electrochemical processes [10.1016/j.jcat.2011.01.013]. The EDL also mediates interactions between the solid and dissolved ions or molecules, impacting interfacial phenomena like adsorption and desorption [10.1038/s41467-020-14860-6].

In the context of propane dehydrogenation catalysts, the EDL can stabilize charged species near the interface and influence electrochemical reactions. Changes in the work function (W) due to the presence of water molecules can lead to the formation of a surface dipole layer, shifting electron density from adsorbates towards the metal, which can affect catalytic activity [10.1021/ja501592y]. The EDL's ability to control ion distribution and facilitate electron transfer between phases is essential for electrocatalytic hydrogenation and other reactions [10.1016/j.apcatb.2015.09.027].

※※※

Integrative understanding and prospective outlook: Considering the advancements discussed in the file, there is an opportunity to explore the role of the EDL in enhancing catalytic efficiency and selectivity. Future research might focus on tailoring the EDL properties through pH adjustments, electrolyte concentration, and surface modifications. Additionally, integrating computational models with experimental data could provide deeper insights into the mechanisms governing ion transport and reaction kinetics at solid-liquid interfaces. This approach could lead to the development of more effective catalysts for industrial applications.

<References>

10.1021/acscatal.6b02532
10.1039/c4cy01720j
10.1016/j.jcat.2011.01.013
10.1038/s41467-020-14860-6
10.1021/ja501592y
10.1016/j.apcatb.2015.09.027
</References>

The electric double layer (EDL) at solid-liquid interfaces is a critical structure that significantly influences various electrochemical processes, including catalysis. The EDL is characterized by the point of zero charge (PZC), where the net charge on the surface becomes zero [10.1016/j.jcat.2011.01.013]. This structure consists of two regions: the inner Helmholtz plane (or Stern layer) and the outer diffuse layer. The inner layer comprises ions or molecules adsorbed onto the solid surface, while the outer layer contains counterions in the liquid phase [10.1021/acscatal.6b02532]. The arrangement of charges near the interface plays a crucial role in mediating electrostatic interactions between the solid and dissolved ions or molecules, thereby affecting interfacial phenomena such as adsorption and desorption [10.1038/s41467-020-14860-6].

The EDL's influence extends to stabilizing colloidal suspensions and influencing ion transport and reaction kinetics, which are essential for electrochemical processes and heterogeneous catalysis [10.1016/j.apcatb.2015.09.027]. Changes in the work function (W) when water molecules are present indicate the formation of a surface dipole layer, where electron density shifts from the adsorbate towards the metal, leading to effective oxidation of the adsorbate [10.1021/ja501592y]. Moreover, the EDL can stabilize or destabilize intermediates involved in reactions like dehydrogenation, impacting the overall reaction rate and mechanism [10.1016/j.jcat.2024.115562].

※※※

Integrative understanding and prospective outlook: Considering the advancements and findings discussed in the file, there lies an opportunity to explore emerging fields and innovative methodologies. Future research endeavors might focus on enhancing the efficiency of propane dehydrogenation catalysts by manipulating the properties of the electric double layer. By optimizing factors such as pH, electrolyte concentration, and surface charge density, researchers can control ion distribution near the interface, thereby improving catalytic activity and selectivity. Additionally, integrating computational models with experimental data could provide deeper insights into the dynamic behavior of charged species at the interface, paving the way for the development of more robust and efficient catalytic systems.

<References>
10.1016/j.jcat.2011.01.013
10.1021/acscatal.6b02532
10.1038/s41467-020-14860-6
10.1016/j.apcatb.2015.09.027
10.1021/ja501592y
10.1016/j.jcat.2024.115562

</References>

11. Fundamental Principles of Solid-Liquid Interfaces -> Physical Chemistry of Interfaces: Surface charge effects

Surface charge effects at solid-liquid interfaces play a critical role in the behavior of these interfaces, particularly in catalytic processes. The incomplete coordination of exposed metal or oxide ions on the solid surface leads to the presence of positive and negative sites, determining the net charge and influencing interface behavior [10.1016/j.jcat.2011.01.013]. These effects can lead to changes in the local pH, affect the adsorption of ions and molecules, and impact the overall stability and reactivity of the system [10.1021/acscatal.6b02532]. Positively or negatively charged surfaces can attract oppositely charged species, altering the local concentration of reactants or intermediates, which can enhance or inhibit specific reactions, impacting overall catalytic performance [10.1039/c4cy01720j].

Furthermore, surface charge effects can alter the distribution of ions and molecules near the interface, affecting processes like adsorption, desorption, and proton transfer. This can lead to changes in reaction pathways and overall catalytic activity [10.1021/acscatal.9b04637]. For instance, the exchange of electrons between OH and H ions derived from water dissociation and the substrate affects the overall behavior of the interface [10.1002/wcms.1272]. Additionally, the nature of water-metal bonding is significantly influenced by surface charge effects; at cathodic potentials, water molecules are repelled from the surface, while at positive potentials, they are attracted closer to the electrode, affecting the strength of water-water interactions and the structure of interfacial water [10.1016/j.cossms.2006.03.007].

※※※

Integrative understanding and prospective outlook: Considering the advancements and findings discussed in the file, there lies an opportunity to explore emerging fields and innovative methodologies. Future research endeavors might focus on developing advanced characterization techniques to better understand the dynamic nature of surface charge effects at solid-liquid interfaces. Moreover, integrating computational modeling with experimental studies could provide deeper insights into how these effects influence catalytic performance. Understanding the microsolvation environment around transition states and its impact on catalytic efficiency and selectivity could open new avenues for designing more efficient catalysts. Additionally, exploring the role of surface charge effects in electrocatalysis and photocatalysis may lead to breakthroughs in renewable energy technologies.

<References>
10.1016/j.jcat.2011.01.013
10.1021/acscatal.6b02532
10.1039/c4cy01720j
10.1021/acscatal.9b04637
10.1002/wcms.1272
10.1016/j.cossms.2006.03.007
</References>

12. Fundamental Principles of Solid-Liquid Interfaces -> Physical Chemistry of Interfaces: Interfacial water structure

Interfacial water structure at solid-liquid boundaries is characterized by the presence of positive and negative sites on the solid surface, which determine the net charge and influence the arrangement of water molecules [10.1016/j.jcat.2011.01.013]. Water molecules near the interface can form ordered layers or exhibit altered hydrogen bonding patterns compared to bulk water, affecting solvation, reaction rates, and other interfacial processes [10.1021/acscatal.6b02532]. Hydrogen bonding and van der Waals interactions with the solid surface can lead to the formation of structured water layers that have implications for solvation, transport properties, and catalytic activity [10.1038/s41467-020-14860-6].

The hydration of protons by water forms specific acids that influence the adsorption of reactants into the catalyst pore [10.1063/5.0211554]. Water molecules can dissociate on oxophilic surfaces like iron, forming Brønsted acid sites that promote hydrogenation reactions [10.1021/acscatal.7b02576]. Additionally, water clusters around Brønsted acid sites play a crucial role in dehydration processes [10.1021/acs.jpcc.1c03846].

Interfacial water structures can facilitate the formation of a microscale aqueous environment, promoting the transport of hydrogen, protons, and oxygen, and stabilizing highly reactive species [10.1021/acscatal.4c02514]. Depending on the nature of the solid surface, water can form one-dimensional chains, two-dimensional bilayers, or three-dimensional clusters/islands [10.1021/acscatal.2c00594]. Large quantities of water can lead to proton transfers via a Grotthus-like mechanism, and at water-oxide interfaces, surface-mediated proton transfer can enable concerted proton exchanges among water molecules [10.1002/wcms.1272].

Interfacial water structure at solid-liquid boundaries is also influenced by the displacement of metal electron density towards the surface, forming an induced surface dipole with the negative end oriented towards the surface [10.1021/ja501592y]. This effect intensifies as water coverage increases. Zeolite pores enhance the association between hydronium ions and reactants, leading to better catalytic performance [10.1134/s0965544120040143]. In contrast, larger pores result in less solvated transition states, affecting reaction dynamics [10.1021/acs.jpcc.9b07738].

On Cu(110), water and methanol form ordered clusters where methanol is located at the periphery of the water network, while on Cu(111), methanol integrates into the water hydrogen-bonding network, leading to complete mixing at a molecular scale [10.1021/acs.jpcc.1c10296]. Interfacial water can stabilize intermediates and participate in proton transfer reactions through hydrogen bonding networks [10.1021/acs.jpcc.0c07033]. Confined within zeolite structures like MFI, water molecules form long-range one-dimensional chains constrained by the framework topology, significantly altering the hydrogen bonding network [10.1080/01614940.2021.1948301].

※※※

Integrative understanding and prospective outlook: Considering the advancements and findings discussed in the file, there lies an opportunity to explore emerging fields and innovative methodologies. Future research endeavors might focus on elucidating the precise mechanisms of water structuring at various solid-liquid interfaces, especially under different environmental conditions. Understanding how interfacial water influences catalytic activity could lead to the

design of more efficient catalysts for industrial applications. Moreover, exploring the impact of interfacial water on electrochemical processes may uncover new strategies for energy conversion and storage technologies. The dynamic nature of interfacial water, including its ability to form unique hydrogen bonding networks, presents opportunities for developing novel materials with tailored properties for catalysis and beyond.

<References>
10.1016/j.jcat.2011.01.013
10.1021/acscatal.6b02532
10.1038/s41467-020-14860-6
10.1063/5.0211554
10.1021/acscatal.7b02576
10.1021/acs.jpcc.1c03846
10.1021/acscatal.4c02514
10.1021/acscatal.2c00594
10.1002/wcms.1272
10.1021/ja501592y
10.1134/s0965544120040143
10.1021/acs.jpcc.9b07738
10.1021/acs.jpcc.1c10296
10.1021/acs.jpcc.0c07033
10.1080/01614940.2021.1948301
</References>

13. Fundamental Principles of Solid-Liquid Interfaces -> Molecular-Level Phenomena: Water ordering at interfaces

The molecular-level phenomena of water ordering at solid-liquid interfaces significantly influence the performance and behavior of propane dehydrogenation catalysts. Water molecules align in specific orientations near the interface, influenced by surface chemistry, leading to structured layers that can extend several molecular diameters into the liquid phase [10.1021/acscatal.6b02532]. Hydrophilic surfaces promote more ordered water structures, while hydrophobic surfaces result in less ordered arrangements, impacting solvation, diffusion, and catalytic activity. This alignment can facilitate or hinder the adsorption and desorption of reactants, affecting overall reaction dynamics [10.1016/j.jcat.2011.01.013].

Water ordering patterns also involve the formation of hydrogen-bonded networks that align with the atomic structure of the solid surface, enhancing or inhibiting certain types of reactions depending on how they affect the mobility and orientation of water molecules [10.1038/s41467-020-14860-6]. In particular, water molecules cluster around Brønsted acid sites, which play a crucial role in dehydration processes [10.1021/acs.jpcc.1c03846]. Moreover, water dissociation on oxophilic surfaces can form Brønsted acid sites, enhancing hydrogenation reactions and facilitating proton transfer [10.1021/acscatal.7b02576].

Additionally, water molecules can form hydronium ion complexes and protonated clusters that act as mobile Brønsted acid sites, altering catalyst selectivity and stabilizing reaction

intermediates [10.1016/j.trechm.2021.03.004]. Proton hopping processes between Brønsted acid sites and water clusters further influence reaction mechanisms and activity [10.1021/acs.jpcc.0c07033]. These patterns are critical for understanding the behavior of water at solid-liquid interfaces and their impact on catalytic processes.

※※※

Integrative understanding and prospective outlook: Considering the advancements and findings discussed in the file, there is an opportunity to explore emerging fields and innovative methodologies. Future research endeavors might focus on developing advanced characterization techniques to better understand the dynamic nature of water ordering at solid-liquid interfaces. Investigating the interplay between surface chemistry and water structure could lead to the design of more efficient and selective catalysts for propane dehydrogenation. Furthermore, integrating computational models with experimental data could provide deeper insights into the molecular-level phenomena governing these interfaces, paving the way for novel catalytic materials and processes.

<References>
10.1021/acscatal.6b02532
10.1016/j.jcat.2011.01.013
10.1038/s41467-020-14860-6
10.1021/acs.jpcc.1c03846
10.1021/acscatal.7b02576
10.1016/j.trechm.2021.03.004
10.1021/acs.jpcc.0c07033
</References>

14. Fundamental Principles of Solid-Liquid Interfaces -> Molecular-Level Phenomena: Surface reconstruction in liquid

Surface reconstruction in liquid environments is a critical phenomenon that involves changes in the atomic arrangement of a solid surface upon contact with a liquid. This process can lead to the formation of new surface structures, which may either enhance or inhibit catalytic activity and selectivity [10.1016/j.jcat.2011.01.013]. The reorganization of atoms can result in the creation of new bonds or defects that alter the electronic and chemical properties of the surface, thereby impacting catalytic performance [10.1021/acscatal.6b02532]. Factors such as the type of liquid, temperature, and pressure play significant roles in influencing these changes. For instance, water adsorption on catalyst surfaces can lead to structural modifications, as observed in Br-doped NaCl crystals where Br-rich islands segregate to the surface at relative humidity levels exceeding 40% [10.1002/wcms.1272].

Moreover, molecular dynamics simulations have been employed to study systems like the aqueous phase/ZrO2(111) interface, revealing highly dynamic interfacial structures where water can exist in both dissociative and molecular forms after adsorption [10.1021/acscatal.7b03298]. The interaction between water molecules and the solid surface can also involve the donation of electron pairs, leading to the formation of new bonds and hydroxyl groups, as seen in TiO2(101) surfaces [10.1080/08927022.2022.2049774]. Additionally, metal clusters like Pt13 deposited on

oxide surfaces undergo shape changes and expansion of their contact areas when exposed to liquids, further demonstrating the adaptability of surfaces to their environment [10.1021/acs.jpcc.7b10208].

※※※

Integrative understanding and prospective outlook: Taking into consideration the advancements and findings discussed in the file, there lies an opportunity to explore emerging fields and innovative methodologies. Future research endeavors might focus on developing predictive models for surface reconstruction under varying liquid conditions. Understanding the intricate mechanisms of how specific liquids influence atomic rearrangement could pave the way for designing more efficient and stable catalysts. Furthermore, integrating advanced characterization techniques with computational methods could provide deeper insights into the dynamic behavior of interfaces, ultimately enhancing our ability to tailor catalysts for specific applications.

<References>
10.1016/j.jcat.2011.01.013
10.1021/acscatal.6b02532
10.1002/wcms.1272
10.1021/acscatal.7b03298
10.1080/08927022.2022.2049774
10.1021/acs.jpcc.7b10208
</References>

15. Fundamental Principles of Solid-Liquid Interfaces -> Molecular-Level Phenomena: Ion-surface interactions

Ion-surface interactions are critical in catalytic processes, particularly in propane dehydrogenation. The key characteristics of these interactions involve the attraction or repulsion between ions and charged surface sites, influencing adsorption, desorption, and reaction rates at the interface [10.1039/c4cy01720j]. Additionally, ion-surface interactions can lead to significant reversible and irreversible changes in solid materials upon exposure to water, altering the material's structure and catalytic properties. The interaction strength varies based on the material's composition and structure [10.1016/s1872-2067(21)64032-9].

Moreover, the balance between chaotropic (disordering) and kosmotropic (ordering) effects determines the water structure at the interface, leading to ion-specific effects known as the Hofmeister effect [10.1021/acscatal.9b04637]. Smaller ions with higher charge densities tend to disrupt the hydrogen bonding network of water near the surface, resulting in more disordered structures, while larger ions with lower charge densities maintain more ordered networks.

Ion-surface interactions can occur through discrete steps or gradual processes. For instance, ions like iodide discharge in discrete steps involving adsorption on the electrode, electron release, and subsequent desorption as a neutral atom. Alternatively, ions may gradually donate charge to the electrode as they approach and undergo desolvation. Chemisorption often involves partial charge transfer between the adsorbate and the surface due to electronegativity differences [10.1016/j.cossms.2006.03.007].

※※※

Integrative understanding and prospective outlook: Considering the advancements and findings discussed in the file, there is an opportunity to explore emerging fields and innovative methodologies. Future research endeavors might focus on developing advanced catalysts that leverage the unique properties of ion-surface interactions. By understanding how different ions influence the surface chemistry of catalysts, researchers can design materials with enhanced stability and activity. Furthermore, exploring the role of water in mediating these interactions could lead to breakthroughs in designing catalysts for industrial applications, such as propane dehydrogenation. Investigating the interplay between ion size, charge density, and surface composition will provide deeper insights into optimizing catalytic performance.

<References>
10.1039/c4cy01720j
10.1016/s1872-2067(21)64032-9
10.1021/acscatal.9b04637
10.1016/j.cossms.2006.03.007
</References>

16. Fundamental Principles of Solid-Liquid Interfaces -> Molecular-Level Phenomena: Hydrogen bonding networks

The formation and function of hydrogen bonding networks at solid-liquid interfaces play a crucial role in catalytic processes, particularly in propane dehydrogenation. Hydrogen bonding networks facilitate proton transfer, stabilize intermediates, and enhance catalytic activity by forming stable complexes with reactants [10.1021/acs.jpcc.7b10208]. For instance, water molecules can form multiple weak interactions with zeolite pores, such as hydrogen bonds and dipole interactions, which are involved in proton transportation, the organization of reactants, and the excess chemical potential for the activation of reactants [10.1002/anie.202313974].

In zeolites, water molecules tend to form aggregates through hydrogen bonding networks. These networks can adopt dispersed or clustered configurations around active sites, enhancing the stability of intermediates and transition states during catalysis [10.1002/aic.15517]. Moreover, the presence of large quantities of water facilitates proton transfers via a Grotthus-like mechanism, promoting water dissociation and proton transfer [10.1021/acscatal.4c02514].

On metal surfaces like Cu(111), methanol integrates into the water's hydrogen-bonding network, leading to thorough mixing at a molecular level. This behavior is influenced by the weaker interaction between the molecules and the Cu(111) surface, allowing for easier adjustment of the hydrogen-bonding network [10.1021/acs.jpcc.1c10296]. Conversely, on Cu(110), water and methanol do not fully mix, with methanol located at the edges of the water network.

Hydrogen bonding networks also influence solvent effects in liquid heterogeneous catalysis. Microsolvation studies have shown that hydrogen bonding at the water/catalyst interface plays a key role in explaining observed solvent effects [10.1021/acscatal.2c00594]. Water clusters consisting of more than two molecules can easily deprive protons from zeolites, forming hydronium ions, which are crucial for zeolite catalysis and exhibit higher intrinsic rates for alcohol dehydration when confined within zeolites [10.1063/5.0211554].

※※※

Integrative understanding and prospective outlook: Considering the advancements and findings discussed, there lies an opportunity to explore emerging fields and innovative methodologies. Future research endeavors might focus on designing catalysts with tailored hydrogen bonding networks to optimize proton transfer pathways. Understanding the dynamic reorganization of water molecules around active sites could lead to the development of new strategies for enhancing catalytic efficiency. Additionally, integrating computational models with experimental data can provide deeper insights into the complex interplay between hydrogen bonding networks and catalytic performance. The study of microsolvation effects could also open avenues for developing novel catalysts that leverage solvent interactions to improve reaction outcomes.

<References>
10.1021/acs.jpcc.7b10208
10.1002/anie.202313974
10.1002/aic.15517
10.1021/acscatal.4c02514
10.1021/acs.jpcc.1c10296
10.1021/acscatal.2c00594
10.1063/5.0211554
</References>

17. Fundamental Principles of Solid-Liquid Interfaces -> Dynamic Processes: Mass transport phenomena

Mass transport phenomena at solid-liquid interfaces play a crucial role in the performance of propane dehydrogenation catalysts. These phenomena can be categorized into external and internal processes. External mass transport occurs in the boundary layer near the particle surface, while internal mass transport happens within the catalyst's pores [10.1021/acscatal.6b02532]. When diffusion rates are slower than the intrinsic reaction rates, significant concentration differences between the bulk fluid and the catalyst surface can lead to diffusion-limited reactions [10.1021/acscatal.6b02532].

In liquid-phase reactions, mass transport effects are more influential than heat transport due to the higher thermal conductivity and heat capacity of liquids compared to gases [10.1021/acscatal.6b02532]. Limitations can be more pronounced in viscous solvents or microporous solids, affecting the overall efficiency of catalytic reactions [10.1021/acscatal.6b02532]. Enhancing diffusion rates can be achieved by modifying the structure and hydrophilicity/hydrophobicity of porous materials, thereby improving the rate of diffusion [10.1021/acscatal.6b02532].

The formation of a microscale aqueous environment on the surface of heterogeneous catalysts promotes the transportation of hydrogen, protons, and oxygen at the solid-liquid interface [10.1021/acscatal.4c02514]. This enhancement facilitates faster and more efficient reactions.

Mass transport phenomena also involve changes in surface charge based on pH. When the solution's pH is below the point of zero charge (PZC), the surface carries a net positive charge,

while it has a net negative charge above the PZC. This affects the movement and interaction of charged species across the interface [10.1016/j.jcat.2011.01.013].

Diffusion limitations can lead to extended treatment times for achieving equilibrium adsorption on catalyst surfaces and uniform distribution of reactants in solution [10.1039/c4cy01720j]. This is particularly relevant for electrodes like Ag, Au, and Pt, where long pre-treatment times are necessary to ensure uniform distribution of gaseous co-reactants.

The concentration of water within the pores of materials like zeolites significantly influences mass transport phenomena. Long-chain alkyl groups deposited on the external surface can markedly influence the diffusion of water into the zeolite pores, affecting the rate of dissolution and stability of the material [10.1016/j.apcatb.2018.06.065].

Solvent properties significantly influence mass transport phenomena at solid-liquid interfaces. In apolar solvents, higher alkylation rates occur because carbenium ion formation is more favorable energetically compared to reactions in water. Differences in the hydration state of the zeolite's Brønsted acid sites affect the types of surface species present and the pathways for electrophile production [10.1038/s41929-017-0015-z].

Changes in solubility and diffusivity of reactants and products caused by the presence of water can directly or indirectly affect overall reaction rates as well as selectivity. Biphasic systems facilitate the separation of hydrophobic products from aqueous-soluble reactants, reducing costs and preventing unwanted side reactions [10.1021/acscatal.9b04637].

※※※

Integrative understanding and prospective outlook: Considering the advancements and findings discussed in the file, there lies an opportunity to explore emerging fields and innovative methodologies. Future research endeavors might focus on developing advanced hierarchical zeolites that combine micropores and mesopores to mitigate mass transfer limitations. Additionally, investigating the impact of solvent properties on mass transport phenomena could lead to the design of more efficient catalysts. Exploring the interplay between surface charge and pH could provide insights into optimizing reaction conditions. Furthermore, the integration of computational modeling with experimental studies could offer a deeper understanding of mass transport dynamics, paving the way for breakthroughs in catalysis.

<References>
10.1021/acscatal.6b02532
10.1021/acscatal.4c02514
10.1016/j.jcat.2011.01.013
10.1039/c4cy01720j
10.1016/j.apcatb.2018.06.065
10.1038/s41929-017-0015-z
10.1021/acscatal.9b04637
</References>

18. Fundamental Principles of Solid-Liquid Interfaces -> Dynamic Processes: Charge transfer dynamics

Charge transfer dynamics at the interface boundary are significantly influenced by the pH of the solution. When the pH is below the point of zero charge (PZC), the surface carries a net positive charge, while it carries a net negative charge when the pH is above the PZC [10.1016/j.jcat.2011.01.013]. Additionally, these dynamics involve complex interactions between electrodes and electrolytes, where techniques like X-ray absorption spectroscopy (XAS) are used to study catalytic turnovers during electrochemical reactions [10.1039/c4cy01720j]. Furthermore, charge transfer dynamics are characterized by the cost of charge separation, leading to the majority of acid/base sites remaining bound at the interface, which minimizes energy expenditure and influences overall catalytic activity and stability [10.1016/s1872-2067(21)64032-9].

Water molecules play a crucial role in charge transfer dynamics by blocking active sites on the surface, forming surface hydroxyl groups, and participating in surface reactions through the Grotthuss proton transfer mechanism [10.1021/acscatal.7b03298]. At negative potentials, water molecules pick up electrons from the electrode, dissociating into hydride and hydroxide ions, while at positive potentials, they dissociate into hydroxide ions and protons, enhancing reaction rates such as CO oxidation and influencing kinetic isotope effects [10.1021/acscatal.9b04637].

Moreover, charge transfer dynamics can be described using Marcus theory, where reactant and product states are separated by a reaction coordinate representing changes in solvation. Assuming a parabolic energy profile relative to this coordinate allows for the determination of activation energy for non-adiabatic electron transfer. Alternatively, charge transfer can follow an adiabatic pathway, requiring quantum mechanical mixing of pre- and post-transfer states [10.1016/j.cossms.2006.03.007].

※※※

Integrative understanding and prospective outlook: Considering the advancements and findings discussed in the file, there lies an opportunity to explore emerging fields and innovative methodologies. Future research endeavors might focus on integrating advanced spectroscopic techniques with theoretical models to better understand the intricate mechanisms of charge transfer dynamics. The development of polarizable continuum models could further enhance our understanding of charge separation and surface polarization in electrochemistry and photocatalysis. Additionally, exploring the impact of different catalyst supports and their electronic properties on charge transfer dynamics may lead to more efficient and stable catalytic systems.

<References>
10.1016/j.jcat.2011.01.013
10.1039/c4cy01720j
10.1016/s1872-2067(21)64032-9
10.1021/acscatal.7b03298
10.1021/acscatal.9b04637
10.1016/j.cossms.2006.03.007
</References>

19. Fundamental Principles of Solid-Liquid Interfaces -> Dynamic Processes: Solvent reorganization

Solvent reorganization plays a critical role in interfacial processes by stabilizing or destabilizing surface-bound intermediates and transition states [10.1021/acscatal.6b02532]. Water, as a mediator, can facilitate surface reactions [10.1021/acscatal.6b02532]. Hydrogen bonds grow stronger with larger and more polar adsorbates, affecting the stability and mobility of surface species [10.1021/acscatal.6b02532]. Water's presence can either limit or enhance the adsorption of organic oxygenates depending on the catalyst, influencing these properties [10.1021/acscatal.6b02532].

Density functional theory calculations indicate that solvent reorganization facilitates the collective adjustment of the hydrogen-bonding network, influenced by the interaction strength between solvent molecules and substrates [10.1021/acs.jpcc.1c10296]. Exposure to polar solvents like water can alter the intrinsic nature of acid surfaces due to solvation effects, such as hydroxyl ions reacting with Lewis sites to generate Brønsted sites [10.1016/j.jcat.2011.01.013].

Solvent reorganization can influence specific reaction steps; for example, it can make α-H abstraction more difficult while significantly lowering the activation energy for dehydration steps [10.1002/cctc.201901736]. In apolar solvents, the absence of water around Brønsted acid sites facilitates easier carbenium ion formation from reactants like alcohols or olefins, leading to higher alkylation rates [10.1038/s41929-017-0015-z].

Within confined environments such as zeolites, water exhibits distinct properties from bulk water due to substrate interactions, affecting catalytic reactions [10.1016/s1872-2067(21)64032-9]. By tuning the composition of organic/water cosolvents, one can control the stability of transition states and enhance reactivity in confined spaces [10.1021/acscatal.9b04637]. Rearranging water molecules near surfaces can create barriers for displacement of molecules like acetic acid and proton transfer [10.1016/j.cossms.2006.03.007].

The dynamic nature of the liquid-solid interface means that water adsorption significantly aids the Gibbs free energy of elementary steps, while desorption penalizes it [10.1021/acscatal.7b03298]. Solvent reorganization provides significant enthalpic gains through solvation of ionic species, promoting otherwise unfavorable reactions [10.1016/s1872-2067(23)64550-4].

※※※

Integrative understanding and prospective outlook: Considering the advancements and findings discussed in the file, there lies an opportunity to explore emerging fields and innovative methodologies. Future research endeavors might focus on developing advanced models that accurately capture the dynamic reorganization of solvents at interfaces. This could lead to better predictions of reaction pathways and energetics in complex systems. Additionally, exploring the impact of different solvent compositions on catalytic activity could uncover new strategies to enhance reaction efficiency. Tailoring solvents to optimize the stability of intermediates and transition states may provide novel insights into designing more efficient catalysts. Furthermore, understanding the microscopic details of solvent-catalyst interactions could guide the development of new materials with tailored properties for specific applications.

<References>

10.1021/acscatal.6b02532
10.1021/acs.jpcc.1c10296
10.1016/j.jcat.2011.01.013
10.1002/cctc.201901736

10.1038/s41929-017-0015-z
10.1016/s1872-2067(21)64032-9
10.1021/acscatal.9b04637
10.1016/j.cossms.2006.03.007
10.1021/acscatal.7b03298
10.1016/s1872-2067(23)64550-4
</References>

20. Fundamental Principles of Solid-Liquid Interfaces -> Dynamic Processes: Surface species mobility

Surface species mobility plays a critical role in the propane dehydrogenation process by influencing desorption, selectivity, and stereoselectivity of adsorbates [10.1021/acscatal.6b02532]. Adsorbate-adsorbate interactions can significantly alter reaction pathways and outcomes, thereby impacting catalytic performance. For instance, coadsorbed species can enhance the desorption of specific adsorbates, which is crucial for maintaining active sites free from poisoning or blocking [10.1021/acscatal.6b02532].

The mobility of hydrogen atoms on metal oxide films, such as FeO, is mediated and accelerated by partially dissociated water molecules at the liquid/solid interface [10.1021/acscatal.4c02514]. This enhancement in diffusion rates can be attributed to a decrease in the activation barrier for hydrogen diffusion from 1.0 eV to 0.2 eV in the presence of water, as demonstrated by DFT + U calculations [10.1002/wcms.1272]. Such changes in surface species mobility directly influence the kinetics of catalytic reactions.

Surface charge variations based on pH also affect interface dynamics. When the solution pH is below the point of zero charge (PZC), the surface has a net positive charge; conversely, it has a net negative charge above the PZC [10.1016/j.jcat.2011.01.013]. These charge alterations can influence the adsorption and desorption processes of reactants and products, thereby modulating catalytic activity.

Hydrophobic environments suppress water adsorption, reducing inhibition by ethanol-water dimers and enhancing catalytic activity [10.1016/s1872-2067(21)64032-9]. Conversely, hydrophilic catalysts may form an aqueous film around the solid surface, leading to mass transfer limitations for organic molecules [10.1021/acscatal.9b04637]. Water's competitive adsorption on active sites and its stabilization of electron-hole pairs further complicate these dynamics [10.1021/acscatal.9b04637].

※※※

Integrative understanding and prospective outlook: Considering the advancements and findings discussed in the file, there lies an opportunity to explore emerging fields and innovative methodologies. Future research endeavors might focus on developing catalysts with tailored surface properties that optimize species mobility. Understanding how to manipulate surface charge and hydration levels could lead to more efficient and selective catalysts. Additionally, integrating advanced computational methods like DFT + U can provide deeper insights into the molecular mechanisms governing surface species mobility, paving the way for rational catalyst design. The dynamic nature of solid-liquid interfaces suggests that adaptive materials, responsive to environmental conditions, could offer significant advantages in catalysis.

<References>
10.1021/acscatal.6b02532
10.1021/acscatal.4c02514
10.1002/wcms.1272
10.1016/j.jcat.2011.01.013
10.1016/s1872-2067(21)64032-9
10.1021/acscatal.9b04637
</References>

21. Interface-Specific Catalytic Phenomena -> Water Effects on Active Sites: Hydration of surface species

Hydration plays a crucial role in influencing surface species at solid-liquid interfaces, particularly in propane dehydrogenation catalysts. The presence of water can stabilize certain adsorbed species more effectively than the transition states, thereby reducing dehydration rates [10.1021/acscatal.6b02532]. Water not only participates as a co-catalyst by serving as a proton shuttle but also enhances reactivity and selectivity by acting as a hydrogen donor [10.1021/acscatal.4c02514]. Changes in pH due to interactions with hydroxyl anions can impact these processes, further altering the intrinsic nature of acid sites [10.1016/j.jcat.2011.01.013].

In zeolites, hydration can transform acidic protons into hydrated hydronium ions, significantly affecting the dehydration rates of compounds like cyclohexanol [10.1021/acscatal.0c05674]. This transformation can lead to higher reaction rates compared to those measured in acidic aqueous solutions. Moreover, hydration influences the stability and lifetime of zeolites, especially when surface modifications such as silylation are applied to retard water diffusion into the pores [10.1016/j.apcatb.2018.06.065].

Additionally, hydration can stabilize intermediates like protonated dimeric propanol and clusters, making certain dehydration pathways less favorable [10.1002/aic.15517]. Water's chemical invasiveness can significantly modify the structure of catalysts at solid-liquid interfaces, altering the trajectory of catalytic processes compared to gas-phase reactions [10.1039/c4cy01720j]. These findings underscore the importance of understanding how hydration affects the behavior of surface species, which is critical for optimizing catalyst performance.

※※※

Integrative understanding and prospective outlook: Considering the advancements and findings discussed, there lies an opportunity to explore emerging fields and innovative methodologies. Future research endeavors might focus on developing catalysts that can mitigate the negative effects of hydration while leveraging its positive impacts. For instance, designing catalysts with tailored active sites that remain stable under hydrated conditions or enhancing the mobility of lattice oxygen through hydration could be promising directions. Additionally, integrating computational models to accurately describe entropy-enthalpy compensation in aqueous environments will provide deeper insights into the mechanisms governing hydration effects. Ultimately, these efforts could lead to more robust and efficient catalysts for industrial applications.

<References>

10.1021/acscatal.6b02532
10.1021/acscatal.4c02514
10.1016/j.jcat.2011.01.013
10.1021/acscatal.0c05674
10.1016/j.apcatb.2018.06.065
10.1002/aic.15517
10.1039/c4cy01720j
</References>

22. Interface-Specific Catalytic Phenomena -> Water Effects on Active Sites: Local pH effects

Water's influence on propane dehydrogenation catalysts is profound, as it can alter the local pH around active sites, thereby affecting catalytic activity. The equilibrium between neutral functional groups and their ionized forms at specific pH values plays a critical role in determining the availability and strength of active sites [10.1016/s1872-2067(21)64032-9]. Increased water presence can reduce acid strength or lead to preferential solvation of intermediates, impacting transition state stability and thus altering the catalytic rate. This is particularly evident in zeolites, where water can ionize Brønsted acid sites (BAS), creating an environment that lowers the reaction energy barrier for certain reactions like alcohol dehydration but may also compete with reactants for these sites, potentially weakening acidity and affecting conversion rates [10.1002/anie.202313974].

Moreover, the mechanisms of local pH effects involve changes in proton affinity at active sites, with proton positions determined by the strongest proton affinity influenced by the local pH environment [10.1002/aic.15517]. In addition, water molecules within zeolite pores can form hydronium clusters acting as mobile catalytically active centers, leading to significant changes in the catalytic properties of the material [10.1021/acs.jpcc.9b07738]. These interactions can either enhance or inhibit catalytic activity depending on their impact on the local environment.

Furthermore, water can significantly alter the nature of Brønsted acid sites in zeolites, converting them into hydrated hydronium ions over a wide range of temperatures and water activities, which impacts catalytic activity [10.1021/jacs.8b07969]. Local pH changes can stabilize charged intermediates and transition states, influencing reaction rates by affecting the charge state of the active site and reactants [10.1016/j.cossms.2006.03.007].

※※※

Integrative understanding and prospective outlook: Considering the advancements and findings discussed, there lies an opportunity to explore emerging fields and innovative methodologies. Future research endeavors might focus on developing catalyst designs that optimize the interaction between water and active sites, thereby enhancing catalytic performance under varying pH conditions. Investigating the precise mechanisms of proton transfer and solvation dynamics could lead to the development of more robust catalysts capable of maintaining high activity and selectivity in aqueous environments. Additionally, exploring the synergistic effects of water and other solvents on catalytic pathways could open new avenues for improving catalytic efficiency in industrial processes. Understanding how to control and manipulate local pH

environments could provide valuable insights into designing catalysts with tailored properties for specific applications.
<References>
10.1016/s1872-2067(21)64032-9
10.1002/anie.202313974
10.1002/aic.15517
10.1021/acs.jpcc.9b07738
10.1021/jacs.8b07969
10.1016/j.cossms.2006.03.007
</References>

Water's influence on the local pH environment around active sites is a critical factor in catalytic activity, particularly for propane dehydrogenation catalysts. The presence of water can alter the equilibrium between neutral functional groups and their ionized forms, impacting the availability and strength of active sites [10.1016/s1872-2067(21)64032-9]. Increased water concentration can reduce acid strength or lead to preferential solvation of intermediates, affecting transition state stability and thus altering the catalytic rate. This is especially significant in zeolites, where water can interact with Brønsted acid sites (BAS), converting them into hydrated hydronium ions and changing their behavior at different temperatures and water activities [10.1021/jacs.8b07969].

Water molecules in H-BEA zeolite pores aggregate and form hydronium water clusters that act as mobile catalytically active centers when more than two water molecules are present at the Brønsted acidic site. As water concentration increases, the protonation of cyclohexanol becomes a significant kinetic step. Initially, the proton remains with the zeolite framework but can transfer to form protonated water clusters [10.1021/acs.jpcc.9b07738]. These changes in the nature of BAS significantly influence the dehydration process, shifting the mechanism from syn-E2 under dry conditions to E1 under wet conditions due to the hydronium cluster acting as a weak Lewis base [10.1021/acs.jpcc.1c03846].

Local pH effects also impact catalytic activity by altering the stability of charged intermediates and transition states. Changes in pH can affect the charge state of the active site and reactants, thereby influencing reaction rates [10.1016/j.cossms.2006.03.007]. For instance, decreasing pH has been shown to enhance the rates of hydrogenation reactions for various oxygenates, indicating that higher hydronium ion concentrations facilitate more effective catalytic processes [10.1016/s1872-2067(23)64550-4].

※※※

Integrative understanding and prospective outlook: Considering the advancements and findings discussed in the file, there lies an opportunity to explore emerging fields and innovative methodologies. Future research endeavors might focus on developing catalyst designs that can mitigate the negative impacts of water while leveraging its positive effects. Investigating how to control water distribution and interaction within the catalyst structure could lead to enhanced catalytic performance. Additionally, understanding the dynamic changes in the protonation state of active sites under varying pH conditions can guide the development of new catalysts tailored for specific reaction environments. Exploring the role of solvent identity and its effect on proton

transfer pathways could provide insights into optimizing catalytic systems for industrial applications. Moreover, integrating computational modeling with experimental studies can offer a deeper understanding of the complex interplay between water, local pH, and catalytic activity.
<References>
10.1016/s1872-2067(21)64032-9
10.1021/jacs.8b07969
10.1021/acs.jpcc.9b07738
10.1021/acs.jpcc.1c03846
10.1016/j.cossms.2006.03.007
10.1016/s1872-2067(23)64550-4
</References>

Water significantly influences the catalytic activity of propane dehydrogenation catalysts by altering the local pH environment around active sites. This effect is primarily observed through changes in the protonation state and solvation dynamics at these sites [10.1016/s1872-2067(21)64032-9]. Increased water presence can reduce acid strength or lead to preferential solvation of intermediates, impacting transition state stability and catalytic rate. The equilibrium between neutral functional groups and their ionized forms varies based on the pH value, affecting the distribution of ionic species across the interfacial region. The spatial distribution is determined by factors such as ionic strength and the dielectric constant of water.

In zeolites, water can ionize Brønsted acid sites (BAS), creating ion environments that lower the reaction energy barrier for certain reactions like alcohol dehydration [10.1002/anie.202313974]. However, water can also compete with reactants for these sites, potentially weakening their acidity and affecting conversion rates. Additionally, water molecules surrounding BAS can form hydronium clusters that act as weak Lewis bases, shifting reaction pathways from syn-E2 mechanisms under dry conditions to E1 mechanisms under wet conditions [10.1021/acs.jpcc.1c03846].

Moreover, the acidic proton in zeolites transforms into a hydrated hydronium ion when exposed to water, leading to significantly higher dehydration rates of cyclohexanol within zeolite pores compared to those in acidic aqueous solutions [10.1021/acscatal.0c05674]. This transformation alters the tetrahedral symmetry around the aluminum atom, leading to changes observable in X-ray absorption spectra and impacting catalytic activity by modifying the acid sites [10.1021/acs.chemmater.7b02133].

※※※

Integrative understanding and prospective outlook: Considering the advancements and findings discussed, there lies an opportunity to explore emerging fields and innovative methodologies. Future research endeavors might focus on developing advanced catalysts that are robust against water-induced deactivation. Understanding how to manipulate the local pH environment to enhance catalytic performance could lead to the design of more efficient and selective catalysts for propane dehydrogenation. Additionally, investigating the role of water in forming new reactive centers and stabilizing intermediates could provide insights into novel catalytic mechanisms. Research into controlling the interaction of water with active sites could also yield strategies to mitigate coking and extend catalyst lifetime.

<References>
10.1016/s1872-2067(21)64032-9
10.1002/anie.202313974
10.1021/acs.jpcc.1c03846
10.1021/acscatal.0c05674
10.1021/acs.chemmater.7b02133
</References>

23. Interface-Specific Catalytic Phenomena -> Water Effects on Active Sites: Competitive adsorption

Competitive adsorption significantly influences interfacial reactions by altering the availability and nature of active sites on catalysts, impacting reaction rates and selectivity. Water molecules can preferentially adsorb onto these sites, thereby reducing the effective concentration of active sites available for reactants [10.1016/s1872-2067(23)64550-4]. This competitive behavior is particularly evident in solid acid catalysts where water competes with alcohols for adsorption sites, potentially decreasing the rate of dehydration reactions [10.1002/aic.15517].

In some cases, water can enhance reaction rates by converting Lewis acid sites to more active Brønsted acid sites [10.1016/s1872-2067(21)64032-9]. However, in other scenarios, water inhibits reactions by blocking critical active sites responsible for product formation [10.1016/j.mcat.2019.110734]. For instance, water molecules form strong hydrogen bonds with Brønsted acid sites (BASs), displacing reactants like benzene and leading to changes in catalytic efficiency [10.1002/anie.202313974].

The presence of water can also lead to the formation of hydronium ion complexes within zeolites, which compete with substrate molecules for adsorption sites [10.1134/s0965544120040143]. Moreover, water's interaction with active sites can modify the energy barriers for certain reactions, such as increasing the formate hydrogenation barrier [10.1016/j.jcat.2012.10.028]. Additionally, water can induce structural changes in catalysts, affecting their stability and performance [10.1021/acs.jpcc.7b10208].

※※※

Integrative understanding and prospective outlook: Considering the advancements and findings discussed in the file, there lies an opportunity to explore emerging fields and innovative methodologies. Future research endeavors might focus on developing catalysts that are less susceptible to competitive adsorption effects from water. This could involve designing hydrophobic catalyst surfaces or modifying existing catalysts to have higher selectivity towards desired reactants. Additionally, investigating the dual role of water—both as a competitor and a promoter—could lead to novel strategies for enhancing catalytic activity and selectivity. Advanced computational models and experimental techniques should be employed to better understand the dynamic interactions between water, reactants, and catalyst surfaces, paving the way for more efficient and robust catalytic systems.

<References>
10.1016/s1872-2067(23)64550-4
10.1002/aic.15517

10.1016/s1872-2067(21)64032-9
10.1016/j.mcat.2019.110734
10.1002/anie.202313974
10.1134/s0965544120040143
10.1016/j.jcat.2012.10.028
10.1021/acs.jpcc.7b10208
</References>

24. Interface-Specific Catalytic Phenomena -> Water Effects on Active Sites: Surface hydroxylation

Surface hydroxylation significantly influences catalytic processes by modifying the reactivity and stability of active sites. In zeolites, water exposure leads to hydrolysis of framework Al atoms, generating framework-associated Al atoms at low temperatures and extra-framework aluminum species at high temperatures [10.1021/acscatal.2c05291]. This transformation can alter the acidity and basicity of the zeolite surface, thereby affecting the activation and stabilization of intermediates [10.1016/j.trechm.2021.03.004].

For PtRe alloy catalysts, surface hydroxylation facilitates water activation, producing OH species that participate in reactions like the water-gas shift and CO bond cleavage, leading to higher rates of CO removal from the surface, enhancing overall catalytic performance [10.1016/j.jcat.2013.11.011]. Hydroxyl groups also act as Brønsted acid sites, protonating reactants and facilitating catalysis, especially under conditions where water induces the formation of new hydroxyl groups [10.1016/s1872-2067(21)64032-9].

In CeO2-based catalysts, surface hydroxylation enhances the interaction between water and CeO2, modifying the interfacial structure. Hydroxyl groups formed from water dissociation fill oxygen vacancy sites and bond strongly to surface atoms, altering the electronic properties and providing new active sites for catalytic reactions [10.1021/acs.jpcc.7b10208].

Surface hydroxylation can influence catalytic processes by promoting proton transfer mechanisms at interfaces. Water molecules stabilize intermediates like CH2OH and COH, altering the reaction pathway and equilibrium between different intermediates [10.1016/j.jcat.2024.115562]. Additionally, it can modify the chemical properties of the catalyst, influencing its ability to activate reactants or stabilize intermediates. Hydroxyl groups can act as Lewis bases or acids, participating directly in catalytic cycles or modifying the local environment around the active sites [10.1016/j.jcat.2011.02.001].

※※※

Integrative understanding and prospective outlook: Considering the advancements and findings discussed in the file, there lies an opportunity to explore emerging fields and innovative methodologies. Future research endeavors might focus on designing catalysts with controlled surface hydroxylation to enhance specific catalytic activities. Investigating the dynamic behavior of hydroxyl groups under varying conditions could lead to the development of more robust and efficient catalysts. Additionally, integrating computational methods to predict and understand the role of surface hydroxylation in catalytic pathways could provide deeper insights into optimizing catalytic systems.

<References>
10.1021/acscatal.2c05291
10.1016/j.trechm.2021.03.004
10.1016/j.jcat.2013.11.011
10.1016/s1872-2067(21)64032-9
10.1021/acs.jpcc.7b10208
10.1016/j.jcat.2024.115562
10.1016/j.jcat.2011.02.001
</References>

25. Interface-Specific Catalytic Phenomena -> Reaction Environment Modifications: Solvation effects on intermediates

Solvation effects play a pivotal role in modifying the stability and reactivity of reaction intermediates during propane dehydrogenation. In confined environments, such as zeolite pores, water's unique properties significantly influence the formation and stability of intermediates, thereby affecting catalytic performance [10.1002/anie.202313974]. Water molecules can form multiple weak interactions with zeolite pores, stabilizing intermediates differently compared to bulk solvents and impacting reaction rates and pathways [10.1016/s1872-2067(21)64032-9]. For instance, hydronium ions within zeolite confines enhance the intrinsic rate constants for reactions involving alcohols, leading to higher catalytic efficiencies [10.1016/j.apcatb.2015.09.027].

Additionally, solvation by water enhances the stability of intermediates such as protonated propanol and propanol-water complexes through hydrogen bonding, which limits their translational and rotational movements and provides van der Waals interactions that stabilize these intermediates along the reaction pathway [10.1002/aic.15517]. The presence of water can also stabilize charged intermediates by allowing surrounding water molecules to interact with these species, lowering activation barriers and influencing the overall energetics of the reaction [10.1016/j.cossms.2006.03.007].

Moreover, solvation effects can alter the electronic structure of metal surfaces and participate in surface reactions through proton transfer and exchange pathways [10.1016/j.apcata.2017.08.005]. This suggests that water's presence can modify the stability and reactivity of intermediates during catalysis, particularly in heterogeneous systems.

※※※

Integrative understanding and prospective outlook: Considering the advancements and findings discussed in the file, there lies an opportunity to explore emerging fields and innovative methodologies. Future research endeavors might focus on developing advanced simulation methods that incorporate both quantum mechanical and molecular dynamics approaches to accurately model solvation effects at the solid-liquid interface. Understanding how different solvent environments impact the stability and reactivity of intermediates could lead to the design of more efficient catalysts for propane dehydrogenation. Additionally, exploring the use of novel confinement strategies, such as nanomaterials or other porous structures, could provide new insights into enhancing catalytic performance by optimizing solvation effects. Furthermore, integrating machine learning algorithms to predict and optimize solvation-induced changes in

intermediate behavior could revolutionize the field of catalysis, paving the way for more sustainable and efficient chemical processes.

<References>
10.1002/anie.202313974
10.1016/s1872-2067(21)64032-9
10.1016/j.apcatb.2015.09.027
10.1002/aic.15517
10.1016/j.cossms.2006.03.007
10.1016/j.apcata.2017.08.005
</References>

26. Interface-Specific Catalytic Phenomena -> Reaction Environment Modifications: Stabilization of transition states

The literature highlights that transition state stabilization in propane dehydrogenation catalysts is significantly influenced by the interaction of solvent molecules, particularly water, with the transition states. Water can stabilize these states through hydrogen bonding networks and electrostatic interactions [10.1016/s1872-2067(21)64032-9]. For instance, at higher water pressures, extensive hydrogen bonding around the transition state can disrupt its formation, leading to greater inhibition [10.1016/s1872-2067(21)64032-9]. Additionally, confined water in subnanometer pores influences the formation and stability of intermediates, facilitating the diffusion and interaction of benzene molecules with surface methoxy species, which stabilizes the transition states [10.1002/anie.202313974].

Increased water concentration leads to the formation of protonated water clusters that stabilize intermediates and transition states, thereby increasing the stability of intermediates like protonated propanol and raising the Gibbs free energies of activation for key reaction steps such as CAO bond scission, identified as the rate-limiting step in dehydration [10.1002/aic.15517]. Furthermore, the restructuring of water clusters induced by the presence of SiOH nests results in entropic gain, compensating for enthalpic losses and enhancing catalytic activity. These stabilized water clusters interact with the transition state, providing additional stabilization and improving overall efficiency [10.1134/s0965544120040143].

Transition state stabilization mechanisms also involve changes in the reaction pathway influenced by the presence of water clusters. Under wet conditions, the E1 mechanism is favored over the syn-E2 mechanism due to the hydronium cluster's effect [10.1021/acs.jpcc.1c03846]. Moreover, the environment provided by zeolite pores increases the activation entropy for dehydration reactions, further stabilizing the transition state [10.1002/anie.201306673].

※※※

Integrative understanding and prospective outlook: Considering the advancements and findings discussed in the file, there lies an opportunity to explore emerging fields and innovative methodologies. Future research endeavors might focus on developing catalysts with tailored pore structures to enhance the confinement effects observed in zeolites. Additionally, leveraging computational tools to predict and optimize the formation of hydrogen-bonded networks could lead to more efficient stabilization of transition states. The role of water in modulating reaction

pathways and activation barriers remains a promising area for further investigation, potentially opening avenues for designing novel catalysts that operate under aqueous conditions with improved selectivity and activity.

<References>
10.1016/s1872-2067(21)64032-9
10.1002/anie.202313974
10.1002/aic.15517
10.1134/s0965544120040143
10.1021/acs.jpcc.1c03846
10.1002/anie.201306673
</References>

27. Interface-Specific Catalytic Phenomena -> Reaction Environment Modifications: Proton transfer mechanisms

Propane dehydrogenation catalysts exhibit unique interface-specific catalytic phenomena, particularly in the context of proton transfer mechanisms. Under steaming conditions, water mobilizes extra-framework aluminum (EFAL) species within zeolite pores, influencing proton transfer at interfaces [10.1021/acscatal.2c05291]. This interaction enhances the intrinsic rate constant for proton transfer reactions within confined spaces like BEA pores, as hydronium-ion active sites associate with substrates [10.1016/s1872-2067(21)64032-9]. Moreover, proton transfer at these interfaces involves reversible hydrogen underpotential deposition when protons are reduced upon contact with a metal surface, leading to adsorbed hydrogen radicals that participate in further reactions [10.1016/j.apcatb.2015.09.027].

Water plays a critical role in facilitating proton transfer through hydrogen-bonded networks or hydronium ion carriers within zeolite channels, enhancing the activation of benzene over ZSM-5 in the presence of water [10.1002/anie.202313974]. The dynamic nature of proton transfer is also evident in the facile and reversible proton transfer between Brønsted acidic sites of H-ZSM-5 zeolite and adsorbed propanol molecules [10.1002/aic.15517]. Furthermore, proton transfer can occur more readily from water to form hydronium ions compared to propanol, indicating a dynamic interface for proton transfer mechanisms [10.1002/aic.15517].

In modified zeolites, proton transfer remains efficient even at lower catalyst loadings, maintaining intrinsic activity by reducing water diffusion into the pores [10.1016/j.apcatb.2018.06.065]. Additionally, the presence of water associated with hydrated hydronium ions limits the useful lifetime of zeolite catalysts in water [10.1016/j.apcatb.2018.06.065]. The dehydration of cyclohexanol via an E1 mechanism where the cleavage of the C-H bond is rate-determining exemplifies the influence of proton transfer on reaction energetics [10.1038/ncomms14113].

※※※

Integrative understanding and prospective outlook: Considering the advancements and findings discussed in the file, there lies an opportunity to explore emerging fields and innovative methodologies. Future research endeavors might focus on developing advanced characterization techniques to better understand the dynamic interactions between water and catalyst surfaces.

Investigating the impact of varying water concentrations on proton transfer mechanisms could provide insights into optimizing catalyst design for enhanced efficiency. Additionally, exploring new materials that can stabilize reactive intermediates while minimizing deactivation pathways will be crucial. The integration of computational modeling with experimental studies will enable a deeper understanding of the molecular-level processes governing proton transfer, potentially leading to breakthroughs in catalysis for industrial applications.
<References>
10.1021/acscatal.2c05291
10.1016/s1872-2067(21)64032-9
10.1016/j.apcatb.2015.09.027
10.1002/anie.202313974
10.1002/aic.15517
10.1016/j.apcatb.2018.06.065
10.1038/ncomms14113
</References>

28. Interface-Specific Catalytic Phenomena -> Reaction Environment Modifications: Local concentration effects

Local concentration effects in propane dehydrogenation catalysts are influenced by a variety of factors, including the spatial distribution of ions within the interfacial region, which varies with distance from the solid surface. This variation can significantly impact molecular reactivity through the local ionic strength effect [10.1016/s1872-2067(21)64032-9]. The presence and behavior of water molecules play a crucial role, as higher water concentrations promote the formation of protonated water clusters and trimeric complexes, providing alternative pathways for dehydration [10.1002/aic.15517]. Additionally, the availability of hydronium ions at the interface, which can be manipulated by adjusting the pH or increasing the surface Brønsted acid site density, typically leads to increased catalytic activity and improved selectivity towards desired products [10.1016/s1872-2067(23)64550-4].

Competitive adsorption between water and reactants also plays a significant role. High water concentrations can lead to the formation of stable water clusters that disrupt the interaction between reactants and active sites, thereby influencing local concentrations [10.1016/j.trechm.2021.03.004]. Moreover, particle size and the strength of water binding influence local concentration effects; strongly bound water layers inhibit methanol adsorption, reducing the local concentration of methanol available for reaction [10.1016/j.jcat.2024.115562].

The diffusion rates of reactants and products near the catalyst surface, solubility of species in the reaction medium, and the rate of consumption or production of key intermediates further control these effects [10.1016/j.jcat.2011.02.001]. Changes in solubility and diffusivity of reactants and products due to the presence of water can directly or indirectly impact overall reaction rates and selectivity [10.1021/acscatal.9b04637]. Finally, the hydration shell of the hydronium ion determines the concentration of water available to facilitate framework hydrolysis, influencing the stability of the catalyst and optimizing local concentrations for efficient catalytic reactions [10.1021/acs.chemmater.7b01847].

※※※

Integrative understanding and prospective outlook: Considering the advancements and findings discussed, there lies an opportunity to explore emerging fields and innovative methodologies. Future research endeavors might focus on developing advanced catalyst designs that optimize the spatial distribution of ions and water molecules. This could involve creating materials with tailored pore structures to enhance local concentration effects, thus improving catalytic efficiency. Additionally, exploring the use of mixed solvents and tuning their interactions with reactive species and catalysts can provide a powerful design space for optimizing kinetics in biomass reactions. Understanding the dynamic nature of interfaces, including competitive adsorption and transport properties, will be crucial for designing more robust and efficient catalysts. Furthermore, integrating computational models with experimental data can offer deeper insights into the mechanisms governing local concentration effects, guiding the development of next-generation catalysts.

<References>
10.1016/s1872-2067(21)64032-9
10.1002/aic.15517
10.1016/s1872-2067(23)64550-4
10.1016/j.trechm.2021.03.004
10.1016/j.jcat.2024.115562
10.1016/j.jcat.2011.02.001
10.1021/acscatal.9b04637
10.1021/acs.chemmater.7b01847
</References>

29. Interface-Specific Catalytic Phenomena -> Structure-Activity Relationships: Surface structure influence

Surface structure significantly influences catalytic activity by determining the nature and dispersion of active sites. Subnanometer Pd clusters, composed of a few Pd atoms, serve as primary active sites in both homogeneous and heterogeneous catalytic systems, facilitating reactions more effectively than isolated Pd atoms or larger nanoparticles [10.1038/s41467-024-53475-z]. The precise arrangement of these clusters within microporous channels enhances their stability and effectiveness. Modification of the support material, such as exchanging H+ with Na+, can enhance or reduce activity depending on the degree of modification, with partial exchange leading to sharply increased activity while higher exchange degrees cause declined performances [10.1038/s41467-024-53475-z].

Additionally, the interaction between the environment and reaction intermediates plays a crucial role in catalytic performance. For instance, water can promote the activity of Pd clusters by nearly one-order magnitude for oxidative coupling reactions by facilitating O2 activation [10.1038/s41467-024-53475-z]. Terrace sites are predominantly active for methanol dehydrogenation, while perimeter sites are less active due to inhibition by strongly bound water molecules [10.1016/j.jcat.2024.115562].

Surface modifications, such as anchoring bulky silanes on the external surface of zeolites, prevent water from diffusing into pores, minimizing micropore loss and slightly reducing Brønsted acid site concentration, thereby enhancing catalyst stability and activity [10.1016/j.apcatb.2018.06.065].

Furthermore, different facets have varying densities of active sites and electronic properties, affecting the adsorption and reaction of molecules on the surface. Certain reactions prefer specific crystallographic planes due to unique atomic arrangements [10.1021/acs.jpcc.7b10208]. Smaller palladium particles show increased surface coverage of reactants but lower turnover frequencies due to stronger binding on edge and corner atoms, which unfavorably affects activation entropy. Larger Pd particles exhibit higher turnover frequencies due to their structure-sensitive nature [10.1016/j.apcata.2017.08.005].

※※※

Integrative understanding and prospective outlook: Considering the advancements and findings discussed in the file, there lies an opportunity to explore emerging fields and innovative methodologies. Future research endeavors might focus on developing advanced characterization techniques that can fully resolve the structure of active sites during catalysis. Understanding the dynamic transformations of supported metal catalysts will be critical for designing more stable and efficient catalysts. Moreover, leveraging computational methods to predict and optimize surface structures could revolutionize catalyst design, enabling tailored catalysts for specific reactions. Exploring the influence of environmental factors, such as water, on catalytic performance can lead to the development of robust catalysts that operate efficiently under diverse conditions. Finally, integrating insights from various facets of catalysis science can pave the way for innovative integrated systems that combine multiple functionalities.

<References>
10.1038/s41467-024-53475-z
10.1016/j.jcat.2024.115562
10.1016/j.apcatb.2018.06.065
10.1021/acs.jpcc.7b10208
10.1016/j.apcata.2017.08.005
</References>

30. Interface-Specific Catalytic Phenomena -> Structure-Activity Relationships: Particle size effects

Particle size critically affects catalytic activity, particularly in propane dehydrogenation reactions. Palladium clusters confined to 0.4 to 0.7 nm exhibit superior stability and activity compared to larger particles [10.1038/s41467-024-53475-z]. These subnanometer clusters maintain their size during reactions due to the stabilizing effect of the zeolite channels, ensuring consistent high catalytic performance. Smaller clusters provide a higher density of active sites per unit volume, leading to more efficient catalytic performance.

In contrast, for platinum catalysts, larger particles are more active per unit area, indicating that particle size significantly affects interface reactions [10.1016/j.jcat.2019.12.034]. This is supported by studies on Pd/ZrO2 catalysts, where smaller Pd particles lead to higher coverages of

reactive intermediates but lower turnover frequencies, suggesting unfavorable activation entropies on small particles [10.1016/j.apcata.2017.08.005].

For Ru/HAP catalysts, fresh samples have smaller particles around 7-9 nm, which increase in size after reduction to 10-16 nm. This change in particle size can influence reactivity and efficiency [10.1016/j.apcatb.2015.05.050]. Similarly, Mo_2C clusters form on the catalyst during reactions, acting as active species, with smaller clusters enhancing interface reactions by providing more reactive sites [10.1016/s0021-9517(03)00236-7].

※※※

Integrative understanding and prospective outlook: Considering the advancements and findings discussed in the file, there lies an opportunity to explore emerging fields and innovative methodologies. Future research endeavors might focus on optimizing particle sizes to maximize active site density and enhance catalytic performance. Additionally, investigating the effects of alloying different metals could yield bimetallic nanoparticles with unique properties, further improving catalytic efficiency. The interplay between particle size and support materials should also be explored to understand how stabilization mechanisms can be leveraged to maintain optimal particle sizes during reactions. Understanding these relationships will pave the way for developing more efficient and stable catalysts for propane dehydrogenation and other critical industrial processes.

<References>
10.1038/s41467-024-53475-z
10.1016/j.jcat.2019.12.034
10.1016/j.apcata.2017.08.005
10.1016/j.apcatb.2015.05.050
10.1016/s0021-9517(03)00236-7
</References>

31. Interface-Specific Catalytic Phenomena -> Structure-Activity Relationships: Support interactions

Support interactions play a crucial role in catalytic performance, particularly in propane dehydrogenation catalysts. Partial exchange of H^+ with Na^+ in Beta zeolite can sharply increase activity in oxidative coupling reactions; however, excessive Na^+ exchange can lead to performance decline [10.1038/s41467-024-53475-z]. This indicates that the balance of support interactions is essential for optimal catalytic performance. The nature of the support material can alter the dispersion and stability of active species, as well as modify their electronic environment, leading to changes in catalytic activity, selectivity, and durability of the supported catalysts [10.1016/s1872-2067(21)64032-9].

For instance, ZrO_2 serves as an excellent support because it provides coordinatively unsaturated metal sites that stabilize phenoxide ions, enhancing interactions with metals like Ni or Pd [10.1016/j.apcata.2017.08.005]. Additionally, adding Ag to Pd/ZrO_2 forms a Pd-Ag alloy, significantly increasing reaction turnover frequencies by lowering activation energy through enhanced electron density on Pd. Strongly bound water molecules at the Pt/Al_2O_3 perimeter

inhibit methanol adsorption and promote reverse reactions, leading to decreased activity [10.1016/j.jcat.2024.115562].

Moreover, support interactions significantly affect catalytic performance by promoting metal/support interactions and influencing other industrial chemical reactions through water dissociation at the interface [10.1021/acscatal.4c02514]. The hydrophobic nature of activated carbon support reduces the interaction between active sites and water molecules, maintaining catalytic activity in aqueous environments [10.1016/j.jcat.2012.08.012]. High porosity and surface area also contribute to preventing deactivation by water.

※※※

Integrative understanding and prospective outlook: Taking into consideration the advancements and findings discussed in the file, there lies an opportunity to explore emerging fields and innovative methodologies. Future research endeavors might focus on developing advanced computational models to predict the impact of support interactions on catalytic performance more accurately. Moreover, the design of novel supports with tailored physicochemical properties could enhance catalytic efficiency while minimizing deactivation pathways. Investigating the role of support interactions in dynamic reaction environments, such as varying water concentrations, could provide deeper insights into optimizing catalyst stability and activity. Additionally, exploring the use of hybrid supports combining multiple beneficial properties (e.g., high surface area, hydrophobicity, and strong metal-support interactions) may open new avenues for improving catalytic performance in various applications.

<References>
10.1038/s41467-024-53475-z
10.1016/s1872-2067(21)64032-9
10.1016/j.apcata.2017.08.005
10.1016/j.jcat.2024.115562
10.1021/acscatal.4c02514
10.1016/j.jcat.2012.08.012
</References>

32. Interface-Specific Catalytic Phenomena -> Structure-Activity Relationships: Stability considerations

Detailed analysis established from the study of reference [10.1021/jacs.3c12437]. Synthesized comprehension stemming from references [10.1038/s41467-024-53475-z] and [10.1016/j.apcatb.2015.09.027].

Propane dehydrogenation catalysts, particularly those involving zeolites and supported metal nanoparticles, must address significant stability considerations to ensure sustained catalytic activity. Zeolite Y, a common support material, exhibits significantly lower stability in hot liquid water compared to steam at the same temperature, leading to framework degradation and reduced acidity even under mild dehydration conditions [10.1021/jacs.3c12437]. Defect-free HY catalysts have been demonstrated to improve stability in hot liquid water, highlighting the importance of minimizing defect sites such as isolated SiOH groups that can lead to siloxane hydrolysis and framework disintegration [10.1134/s0965544120040143].

The stabilization effect of the 12MR channels of Beta zeolite on Pd clusters is crucial for preventing their agglomeration into larger particles, which would otherwise deactivate the catalyst. Maintaining the integrity of these subnanometer Pd clusters within the zeolite channels ensures sustained catalytic activity [10.1038/s41467-024-53475-z]. Additionally, the presence of rare-earth exchanged and stabilized HY catalysts enhances hydrothermal resilience, making them suitable for fluidized catalytic cracking processes [10.1021/jacs.3c12437].

For catalysts operating in aqueous environments, controlling the concentration of hydrated hydronium ions is vital to minimize intraporous water concentration, thereby reducing the rate of framework hydrolysis and extending the catalyst's lifespan [10.1021/acs.chemmater.7b01847]. Hydrophobization with long-chain alkyl groups can further enhance stability by protecting the external surface from water-induced degradation [10.1016/j.apcatb.2018.06.065].

Stability considerations also encompass preventing metal particle sintering and managing reversible deactivation. Sintering rates depend on the support's electronegativity, while reversible deactivation correlates with the support's surface charge and the presence of carboxylic acids. Irreversible deactivation due to particle sintering is critical and influenced by the nature of the support [10.1016/j.jcat.2015.04.026].

※※※

Integrative understanding and prospective outlook: Taking into consideration the advancements and findings discussed in the file, there lies an opportunity to explore emerging fields and innovative methodologies. Future research endeavors might focus on developing novel strategies to enhance the hydrothermal stability of zeolite-based catalysts, particularly through the design of defect-free frameworks and advanced hydrophobic treatments. Additionally, investigating the synergistic effects between different metal nanoparticles and their supports could yield more robust catalysts with prolonged lifetimes. The integration of computational modeling to predict and optimize structure-stability-activity relationships will be pivotal in advancing the field of interface catalysis.

<References>
10.1021/jacs.3c12437
10.1038/s41467-024-53475-z
10.1016/j.apcatb.2015.09.027
10.1134/s0965544120040143
10.1021/acs.chemmater.7b01847
10.1016/j.apcatb.2018.06.065
10.1016/j.jcat.2015.04.026
</References>

33. Characterization and Analysis Methods -> In-situ Spectroscopic Techniques: Surface-sensitive methods

Surface-sensitive methods play a crucial role in the characterization and analysis of propane dehydrogenation catalysts. Techniques such as in situ infrared (IR) spectroscopy are utilized to monitor the equilibration between zeolites and gas-phase molecules in real-time, providing

detailed insights into the interaction dynamics at the interface [10.1021/jacs.5b09107]. EXAFS studies have been employed to characterize the active phase formed on catalysts, revealing the formation of small Mo2C clusters that act as active species during reactions [10.1016/s0021-9517(03)00236-7]. FT-IR spectroscopy has also been applied to study interface structures, confirming an increase in Lewis acidity through pyridine adsorption studies on Ru/HAP catalysts [10.1016/j.apcatb.2015.05.050].

Modular excitation spectroscopy (MES) with phase-sensitive detection (PSD) enhances the ability to detect adsorbates and surface intermediates using ATR-FTIR, especially in cases where these species are difficult to observe [10.1038/s41467-020-14860-6]. Advances in near-ambient-pressure X-ray photoelectron spectroscopy (NAP-XPS) enable monitoring surface characteristics under low-pressure conditions, identifying adsorbate-induced surface reconstruction in bimetallic alloys [10.1021/acscatal.2c01838]. In situ X-ray absorption spectroscopy (XAS), including X-ray absorption near edge (XANES) and extended X-ray absorption fine structure (EXAFS), is used to probe hydrogen coverages and organic species on catalyst surfaces during reactions, identifying different phases of Pd, such as b-PdHx and a-PdHx [10.1016/j.jcat.2019.12.034].

Operando methodology combines in situ spectroscopy with simultaneous activity measurements under catalytic reaction conditions, offering dynamic insights into the atomic and electronic structures of catalysts during operation [10.1021/jacsau.1c00355]. Vibrational spectroscopies provide valuable information on the nature and concentration of adsorbates on solid surfaces, addressing mass transport issues within the experimental setup to capture transient phenomena with high temporal resolution [10.1039/c4cy01720j]. Scanning tunneling microscopy (STM) and qPlus-based noncontact atomic force microscopy (nc-AFM) have been used to study microsolvation and hydration processes on solid surfaces, enabling visualization of chemical structures with single-bond resolution [10.1021/acs.jpcc.1c10296].

※※※

Integrative understanding and prospective outlook: Considering the advancements and findings discussed, there lies an opportunity to explore emerging fields and innovative methodologies. Future research endeavors might focus on integrating multiple in situ spectroscopic techniques to gain comprehensive insights into the dynamic behavior of catalysts under realistic reaction conditions. Combining operando spectroscopy with computational modeling can provide a deeper understanding of the structure-function relationships in catalysts. Additionally, developing advanced surface-sensitive tools to interrogate complex interfaces, particularly in aqueous environments, could unlock new pathways for designing highly efficient catalysts. The integration of machine learning algorithms with in situ data could accelerate the discovery of novel catalyst materials and optimize existing ones.

<References>

10.1021/jacs.5b09107
10.1016/s0021-9517(03)00236-7
10.1016/j.apcatb.2015.05.050
10.1038/s41467-020-14860-6
10.1021/acscatal.2c01838
10.1016/j.jcat.2019.12.034
10.1021/jacsau.1c00355
10.1039/c4cy01720j

10.1021/acs.jpcc.1c10296
</References>

34. Characterization and Analysis Methods -> In-situ Spectroscopic Techniques: Time-resolved studies

Time-resolved studies play a vital role in understanding interface dynamics by capturing transient states and kinetic parameters that cannot be observed in steady-state experiments. This approach helps elucidate the detailed mechanisms of catalytic reactions [10.1016/j.apcatb.2015.09.027]. Specifically, time-resolved studies allow researchers to monitor the equilibration process between zeolites and gas-phase molecules, providing insights into reaction mechanisms [10.1021/jacs.5b09107].

Moreover, these studies capture transient changes in catalyst structure and reactivity over time, offering deeper insights into the mechanisms of interface reactions [10.1016/s0021-9517(03)00236-7]. For example, time-resolved in situ solid-state NMR data revealed that the initial rate of disappearance of a labeled reactant was significantly different from its product formation rate, indicating the reaction did not follow an E2-type mechanism [10.1016/s1872-2067(21)64032-9].

Additionally, time-resolved studies enable researchers to observe how catalyst surfaces evolve over time during reactions, identifying transient species, intermediates, and reaction pathways that might not be visible in steady-state experiments [10.1021/acs.jpcc.7b10208]. Rapid-scan spectroscopic measurements with subsecond or millisecond temporal resolution can address questions about the relevance of surface intermediates in reactions [10.1039/c4cy01720j].

※※※

Integrative understanding and prospective outlook: Taking into consideration the advancements and findings discussed in the file, there lies an opportunity to explore emerging fields and innovative methodologies. Future research endeavors might focus on integrating advanced time-resolved spectroscopic techniques with computational models to gain a comprehensive understanding of catalytic processes. This integration could lead to the development of more efficient and selective propane dehydrogenation catalysts. Moreover, exploring the dynamic behavior of catalysts under realistic operating conditions using ultrafast spectroscopy could uncover new mechanistic insights, paving the way for breakthroughs in catalysis science.

<References>
10.1016/j.apcatb.2015.09.027
10.1021/jacs.5b09107
10.1016/s0021-9517(03)00236-7
10.1016/s1872-2067(21)64032-9
10.1021/acs.jpcc.7b10208
10.1039/c4cy01720j
</References>

35. Characterization and Analysis Methods -> In-situ Spectroscopic Techniques: Operando measurements

Operando measurements in interface analysis allow real-time monitoring of catalytic processes under actual operating conditions, providing insights into the dynamic changes in catalyst structure and function during reactions [10.1016/j.apcatb.2015.09.027]. These techniques are implemented using instruments like the Bruker Vertex 70 spectrometer, which records in situ infrared spectra of adsorbed species in transmission absorption mode, allowing continuous monitoring of interface reactions [10.1021/jacs.5b09107]. Operando measurements such as EXAFS experiments monitor changes in active species over time, tracking transformations of Mo2C clusters during reactions to provide insights into their stability and reactivity [10.1016/s0021-9517(03)00236-7]. Techniques such as TPD and FT-IR offer real-time data on catalyst behavior during reactions [10.1016/j.apcatb.2015.05.050]. Combining electrocatalytic and thermal catalytic hydrogenation kinetics with spectroscopy allows for the deduction of a microkinetic model for hydrogenation reactions, offering insights into active sites and particle size effects [10.1016/j.jcat.2019.12.034]. Ensuring samples remain isolated and uncontaminated throughout the process is critical; after reduction, samples are transferred to a capillary in a glove box sealed with wax, and the capillary is flame-sealed to prevent air contamination. X-ray absorption spectra recorded at the Pd K-edge provide real-time data on the catalyst's electronic and geometric structure during the reaction [10.1016/j.apcata.2017.08.005]. Operando measurements integrate experimental observations with theoretical models to analyze interface reactions under realistic conditions, combining FTIR data with multiscale and microkinetic modeling to probe active sites and mechanisms involved in methanol dehydrogenation [10.1016/j.jcat.2024.115562].

※※※

Integrative understanding and prospective outlook: Considering the advancements and findings discussed, operando measurements represent a powerful tool for gaining detailed insights into catalytic processes under real-world conditions. Future research can explore more sophisticated combinations of spectroscopic techniques and kinetic modeling to enhance our understanding of complex catalytic systems. The integration of machine learning algorithms could further refine the analysis of large datasets generated by operando studies, potentially leading to the discovery of new catalysts with superior performance. Moreover, developing miniaturized reactors and advanced sample handling protocols will enable operando studies under a wider range of conditions, expanding the horizons of catalysis research.

<References>
10.1016/j.apcatb.2015.09.027
10.1021/jacs.5b09107
10.1016/s0021-9517(03)00236-7
10.1016/j.apcatb.2015.05.050
10.1016/j.jcat.2019.12.034
10.1016/j.apcata.2017.08.005
10.1016/j.jcat.2024.115562
</References>

36. Characterization and Analysis Methods -> In-situ Spectroscopic Techniques: Advanced imaging approaches

The characterization and analysis of propane dehydrogenation catalysts have significantly benefited from advanced in-situ spectroscopic techniques. Standard surface science techniques such as scanning tunneling microscopy (STM), vibrational, and X-ray spectroscopy face challenges when applied to solid-liquid interfaces [10.1021/acscatal.2c00594]. However, alternative methods like calorimetry, inelastic neutron scattering, nonlinear optical spectroscopies, and surface-sensitive X-ray diffraction methods are more suitable for studying these interfaces.

Advanced imaging approaches, notably qPlus-based noncontact atomic force microscopy (nc-AFM), have been utilized to visualize the chemical structure of organic and water molecules with single-bond resolution by probing Pauli repulsive forces and weak high-order electrostatic forces [10.1021/acs.jpcc.1c10296]. Scanning tunneling microscopy (STM) has also been applied to investigate microsolvation and hydration processes of various molecules on solid surfaces, although it is limited by its sensitivity to electronic states rather than atomic positions [10.1021/acs.jpcc.1c10296].

Operando UV-vis spectroscopy allows for the observation of catalysts under working conditions, providing insights into phenomena such as coke formation over zeolite catalysts during reactions like methanol-to-olefins [10.1021/acscatal.3c01945]. Additionally, in situ spectroscopy and spatial and temporal exploration with synchrotron radiation provide detailed insights into the spatiotemporal evolution of molecules and active sites during catalytic processes [10.1021/acscatal.3c01945].

Interface-specific vibrational spectroscopies, such as attenuated total reflectance infrared (ATR-IR) spectroscopy, offer information on the nature and concentration of adsorbates on solid surfaces. Recent advancements involve using cost-effective Si wafers for ATR-IR measurements and versatile reactor designs combining ATR optics with transmission windows for multiphasic system analysis [10.1039/c4cy01720j]. Techniques such as rapid-scan ATR-IR, conventional Raman spectroscopy, sum frequency generation (SFG), second harmonic generation (SHG), X-ray absorption spectroscopy (XAS), small-angle X-ray scattering (SAXS), wide-angle X-ray scattering (WAXS), and UV-vis spectroscopy further enhance our understanding of catalytic interfaces in aqueous environments [10.1039/c4cy01720j].

Nuclear magnetic resonance (NMR) and infrared spectroscopy have been applied to discern the chemical environment of protons, while in situ X-ray absorption spectroscopy and X-ray absorption near edge structures (XANES) have been used to differentiate the state of active sites with increasing water concentrations [10.1021/acs.jpcc.0c07033].

※※※

Integrative understanding and prospective outlook: The integration of advanced in-situ spectroscopic techniques offers unprecedented insights into the complex dynamics of propane dehydrogenation catalysts. Future research should focus on developing more sophisticated operando methodologies that can capture transient states and dynamic changes in real-time. Innovations in instrumentation, such as higher-resolution nc-AFM and faster-scanning ATR-IR, will enable a deeper understanding of molecular-level interactions at interfaces. Moreover, the combination of multiple in-situ techniques could provide comprehensive datasets, leading to more

accurate models and predictions. The application of machine learning algorithms to analyze large datasets generated by these techniques may uncover hidden patterns and correlations, paving the way for smarter catalyst design and optimization.

<References>
10.1021/acscatal.2c00594
10.1021/acs.jpcc.1c10296
10.1021/acscatal.3c01945
10.1039/c4cy01720j
10.1021/acs.jpcc.0c07033
</References>

37. Characterization and Analysis Methods -> Computational Approaches: Ab initio molecular dynamics

Ab initio molecular dynamics (AIMD) has emerged as a powerful tool in the study of propane dehydrogenation catalysts, particularly in elucidating the dynamic interfacial structures and proton transfer mechanisms at solid-liquid interfaces. AIMD simulations provide detailed insights into the behavior of molecules at surfaces and within confined spaces like zeolites, which are crucial for understanding catalytic processes [10.1021/acscatal.4c02514]. For instance, these simulations help investigate the interactions between water clusters and Brønsted acid sites (BAS), revealing how solvation significantly influences reaction energetics [10.1021/acs.jpcc.0c07033].

In the context of propane dehydrogenation, AIMD is applied to simulate the diffusion and interaction of reactants within zeolite structures, such as H-BEA-15 and H-ZSM-5, under specific conditions like temperature and composition [10.1021/acs.jpcc.9b07738]. These simulations are conducted in the canonical ensemble (NVT) using software like CP2K, allowing researchers to capture the dynamic evolution of acid sites and their interactions with reactant molecules [10.1021/acscatal.7b03298]. The method also aids in understanding the effects of coadsorbed water on the electronic structure of metal surfaces, impacting adsorption energies for intermediates during catalytic reactions [10.1021/acscatal.6b02532].

Moreover, AIMD combined with enhanced sampling methodologies allows for the detailed examination of processes such as proton transfer and the interaction between water molecules and aluminum atoms during hydrolysis steps [10.1021/acs.jpcc.1c06847]. This approach facilitates the mapping out of high-dimensional free energy profiles and identification of various states and processes involved in catalysis [10.1021/acs.jpclett.3c02233].

※※※

Integrative understanding and prospective outlook: Taking into consideration the advancements and findings discussed in the file, there lies an opportunity to explore emerging fields and innovative methodologies. Future research endeavors might focus on integrating machine learning with AIMD to better understand the dynamic behavior of heterogeneous catalysts during reactions [10.1021/jacsau.1c00355]. Additionally, expanding the scope of AIMD studies to include larger and more complex systems could provide deeper insights into the microsolvation effects and collective phenomena that control reactivity in zeolites [10.1021/acs.jpcc.1c06873]. Furthermore, developing more efficient computational algorithms to

reduce the computational cost of AIMD simulations would enable broader applications in studying solid-liquid interfaces [10.1021/acscatal.7b04367].

<References>
10.1021/acscatal.4c02514
10.1021/acs.jpcc.0c07033
10.1021/acs.jpcc.9b07738
10.1021/acscatal.7b03298
10.1021/acscatal.6b02532
10.1021/acs.jpcc.1c06847
10.1021/acs.jpclett.3c02233
10.1021/jacsau.1c00355
10.1021/acs.jpcc.1c06873
10.1021/acscatal.7b04367
</References>

38. Characterization and Analysis Methods -> Computational Approaches: Interface modeling methods

Propane dehydrogenation catalysts are pivotal in petrochemical processes, and the characterization of their interfaces is essential for optimizing catalytic performance. Computational approaches play a crucial role in this endeavor by providing insights into the complex interactions at the catalyst/water interface. Key interface modeling methods currently in use include implicit solvent models or polarizable continuum models (PCM) [10.1021/acscatal.2c00594], which have been increasingly utilized over the last decade to model reactions at this interface. Microsolvation techniques are employed to probe hydrogen bonding effects at the water/catalyst interface [10.1021/acscatal.2c00594]. Efforts towards achieving a full atomistic description of the water/liquid interface have also seen considerable dedication [10.1021/acscatal.2c00594].

Periodic density functional theory (DFT) calculations using the CP2K code with a mixed Gaussian and plane wave basis set are key methods for studying dehydration reactions [10.1021/acs.jpcc.9b07738]. Transition states for elementary steps in these reactions are identified using the climbing image nudged elastic band method [10.1021/acs.jpcc.9b07738]. Additionally, ab initio molecular dynamics simulations (AIMD) and DFT calculations help characterize detailed structures of water and its interactions with Brønsted acid sites [10.1021/acs.jpcc.0c07033].

The bilayer adsorption/ice model considers the solid-like behavior of ice films, allowing classical partition functions to be used for free-energy estimations [10.1021/acscatal.7b04367]. Implicit solvation models treat water as a continuous medium without structure, while explicit solvation models add water molecules directly to the adsorbate/surface system [10.1021/acs.jpcc.3c06870]. Explicit hybrid quantum mechanical and molecular mechanical (QM/MM) models provide a rational improvement by combining well-calibrated multilevel QM and MM methods with proper free-energy estimators [10.1021/acscatal.7b04367].

For specific systems like the aqueous phase/ZrO2(111), AIMD simulations represent the liquid environment with explicit water molecules, achieving an equilibrated dynamic interface

between the solid and liquid phases [10.1021/acscatal.7b03298]. Classical thermodynamic methods calculate Gibbs free reaction energy and activation energy to account for entropic contributions and zero-point energy corrections [10.1021/acscatal.7b03298].

Density functional theory (DFT), ab initio molecular dynamics (AIMD), and machine learning potentials accurately describe van der Waals interactions and dispersion corrections, enhancing the understanding of catalytic function under operating conditions [10.1021/acscatal.3c01945]. In situ spectroscopy combined with modeling studies, as well as DFT and MD simulations, visualize interactions such as hydrogen bonding between solvents and adsorbed species [10.1021/acscatal.6b02532].

Constructing reasonable chemical reaction models involves exploring possible elementary reactions and intermediates computationally [10.1021/acs.jpclett.3c02233]. Ab initio molecular dynamics helps identify elementary reactions through realistic atomic-scale simulations, with energy barriers estimated using path optimization techniques [10.1021/acs.jpclett.3c02233].

※※※

Integrative understanding and prospective outlook: Considering the advancements and findings discussed in the file, there lies an opportunity to explore emerging fields and innovative methodologies. Future research endeavors might focus on integrating advanced computational tools like machine learning with traditional DFT and AIMD methods to enhance predictive accuracy and efficiency. The development of multiscale models that bridge the gap between microscopic and macroscopic phenomena could provide deeper insights into catalytic mechanisms. Moreover, the inclusion of real-time experimental data through in situ spectroscopy can refine computational models, leading to more accurate predictions of catalytic behavior. Exploring new solvation models that better capture the dynamic nature of interfaces could revolutionize our understanding of catalysis in complex environments.

<References>
10.1021/acscatal.2c00594
10.1021/acs.jpcc.9b07738
10.1021/acs.jpcc.0c07033
10.1021/acscatal.7b04367
10.1021/acs.jpcc.3c06870
10.1021/acscatal.7b03298
10.1021/acscatal.3c01945
10.1021/acscatal.6b02532
10.1021/acs.jpclett.3c02233
</References>

39. Characterization and Analysis Methods -> Computational Approaches: Reaction pathway analysis

Reaction pathway analysis for propane dehydrogenation catalysts involves a comprehensive suite of computational approaches to elucidate the mechanisms and intermediates involved in catalytic reactions. These methods include calculating Gibbs free energy profiles for different reaction pathways, identifying transition states using enhanced sampling techniques such as the

climbing image nudged elastic band (CI-NEB) method [10.1021/acs.jpcc.7b10208], and employing quantum mechanical/molecular mechanical (QM/MM) minimum free-energy path methods to optimize intrinsic reaction coordinates on quantum chemical potential of mean force surfaces [10.1021/acscatal.7b04367].

Computational simulations, especially density functional theory (DFT) methods, play a crucial role in investigating mechanisms like catalyst deactivation and dealumination [10.1016/j.trechm.2021.03.004]. For instance, DFT calculations are used to model and calculate the energy profiles of reactions, such as the dehydration of cyclohexanol catalyzed by hydronium ions in zeolites [10.1038/ncomms14113]. Additionally, microkinetic modeling combined with DFT calculations is employed to examine steps involved in reactions such as hydrodeoxygenation on different metal surfaces [10.1021/acscatal.6b02532].

Mapping out proton hopping processes between Brønsted acid sites and water clusters or within water clusters using free energy landscapes helps understand configurational effects on various reaction steps [10.1021/acs.jpcc.0c07033]. Configuration sampling is also essential for understanding the effects of water molecules on activation energy, reaction energy, Gibbs free energy of activation, and Gibbs free reaction energy [10.1021/acscatal.7b03298].

※※※

Integrative understanding and prospective outlook: Considering the advancements and findings discussed in the file, there lies an opportunity to explore emerging fields and innovative methodologies. Future research endeavors might focus on integrating operando modeling with advanced computational tools to simulate real-time reaction conditions at the atomic scale. This approach can provide deeper insights into structural changes and varying catalytic performance during reactions. Moreover, developing hybrid models that combine QM/MM and 3D-RISM-KH methodologies could offer more accurate representations of solvent effects on reaction pathways. The integration of these techniques will not only enhance our understanding of complex catalytic processes but also aid in designing more efficient and robust catalysts for industrial applications.

<References>
10.1021/acs.jpcc.7b10208
10.1021/acscatal.7b04367
10.1016/j.trechm.2021.03.004
10.1038/ncomms14113
10.1021/acscatal.6b02532
10.1021/acs.jpcc.0c07033
10.1021/acscatal.7b03298
</References>

40. Characterization and Analysis Methods -> Computational Approaches: Machine learning applications

The literature predominantly lacks specific information on the applications of machine learning in interface studies [10.1021/acscatal.2c00594, 10.1021/acscatal.4c02514, 10.1021/acs.jpcc.9b07738, 10.1021/acscatal.7b04367, 10.1038/ncomms14113, 10.1021/acs.jpcc.0c07033, 10.1002/aic.15517, 10.1021/acs.jpcc.3c06870,

10.1021/acscatal.7b03298, 10.1021/acs.jpcc.1c06847, 10.1021/acs.jpcc.7b10208, 10.1063/5.0211554, 10.1021/acscatal.9b04637, 10.1021/jp404772p, 10.1016/s1872-2067(23)64550-4, 10.1021/acscatal.5b01217, 10.1021/acscatal.6b02532, 10.1021/acscatal.7b02576, 10.1016/j.trechm.2021.03.004, 10.1021/acs.jpcc.1c03846, 10.1021/acs.jpcc.1c10296, 10.1002/anie.202313974, 10.1038/s41467-020-14860-6, 10.1021/jacs.8b07969, 10.1016/j.cossms.2006.03.007, 10.1021/ja501592y, 10.1021/acs.jpcc.1c06873]. However, there are a few notable exceptions.

Machine learning techniques are increasingly being utilized alongside computational chemistry to design new reactions and catalysts by elucidating the atomistic mechanisms of complex chemical reactions at interfaces [10.1021/acs.jpclett.3c02233]. Emerging applications include constructing machine learning potentials (MLPs) that extend accessible length and time scales. MLPs are derived from quantum-mechanics-based training data using regression methods. When correctly trained, MLPs can calculate energies and forces with similar accuracy to quantum-mechanical data, allowing for molecular dynamics (MD) simulations on submicrosecond time scales [10.1021/acscatal.3c01945].

Machine learning is also being used to bridge the complexity gap in computational heterogeneous catalysis, enhance surface kinetic Monte Carlo simulations, and advance electronic structure calculations [10.1021/acscatal.3c01945]. These tools offer new ways to analyze large datasets generated from experimental and computational studies, assisting in identifying patterns and correlations that might not be apparent through traditional methods, leading to improved predictive models and catalyst design strategies [10.1039/c4cy01720j].

※※※

Integrative understanding and prospective outlook: Considering the advancements and findings discussed in the file, there lies an opportunity to explore emerging fields and innovative methodologies. Future research endeavors might focus on integrating machine learning with more sophisticated quantum mechanics models to further refine predictions of catalyst performance. Additionally, exploring the dynamic nature of heterogeneous catalysts during reactions using operando modeling could provide deeper insights into reaction mechanisms. The development of advanced machine learning algorithms tailored for catalytic systems could revolutionize how we understand and design propane dehydrogenation catalysts, potentially leading to breakthroughs in efficiency and selectivity.

<References>

10.1021/acscatal.2c00594
10.1021/acscatal.4c02514
10.1021/acs.jpcc.9b07738
10.1021/acscatal.7b04367
10.1038/ncomms14113
10.1021/acs.jpcc.0c07033
10.1002/aic.15517
10.1021/acs.jpcc.3c06870
10.1021/acscatal.7b03298
10.1021/acs.jpcc.1c06847
10.1021/acs.jpcc.7b10208
10.1063/5.0211554

10.1021/acscatal.9b04637
10.1021/jp404772p
10.1016/s1872-2067(23)64550-4
10.1021/acscatal.5b01217
10.1021/acscatal.6b02532
10.1021/acscatal.7b02576
10.1016/j.trechm.2021.03.004
10.1021/acs.jpcc.1c03846
10.1021/acs.jpcc.1c10296
10.1002/anie.202313974
10.1038/s41467-020-14860-6
10.1021/jacs.8b07969
10.1016/j.cossms.2006.03.007
10.1021/ja501592y
10.1021/acs.jpcc.1c06873
10.1021/acs.jpclett.3c02233
10.1021/acscatal.3c01945
10.1039/c4cy01720j
</References>

41. Characterization and Analysis Methods -> Integrated Analysis Strategies: Multi-technique approaches

Multi-technique approaches in interface studies are pivotal for gaining comprehensive insights into catalytic processes. These methods integrate various experimental and computational techniques to provide a holistic understanding of catalyst behavior under realistic conditions [10.1021/acscatal.3c01945]. Specifically, operando spectroscopy combined with computational methods such as DFT (Density Functional Theory) and AIMD (Ab Initio Molecular Dynamics) allows researchers to monitor structural and electronic changes at the molecular level during catalysis. Furthermore, combining in situ spectroscopy with simultaneous activity measurements under catalytic reaction conditions enables an integrated analysis of both time and space, enhancing our understanding of interface processes.

Rigorous reaction tests, kinetic and isotopic probes, advanced characterization techniques, and theoretical methods collectively contribute to unraveling complex reaction mechanisms and pathways [10.1016/s1872-2067(23)64550-4]. For instance, isotopic substitution effects and DFT calculations have provided valuable insights into the formation rates of intermediates and products, such as H2O2 from methanol or methanol-water mixtures.

The integration of solid-state NMR spectroscopy and theoretical calculations has been instrumental in investigating molecular interactions and complex formations within zeolite pores [10.1002/anie.202313974]. This approach elucidates the role of water molecules in promoting the formation of highly active complexes via C-H···π interactions, as evidenced by 2D magic-angle spinning (MAS) NMR spectroscopy.

Additionally, multi-technique approaches can involve combining EXAFS (Extended X-ray Absorption Fine Structure) analysis with 27Al MAS NMR spectroscopy, supported by DFT-based molecular dynamics simulations [10.1021/ja501361v]. This combination provides quantitative data on the local structure and environment around catalytically active sites, which is crucial for optimizing catalyst performance.

※※※

Integrative understanding and prospective outlook: Considering the advancements and findings discussed in the file, there lies an opportunity to explore emerging fields and innovative methodologies. Future research endeavors might focus on developing more sophisticated multi-technique platforms that seamlessly integrate real-time monitoring with predictive modeling. By leveraging advanced data correlation methods such as statistical analysis and machine learning algorithms, researchers can gain deeper insights into dynamic catalytic processes. Moreover, the synergy between experimental and theoretical approaches will pave the way for designing novel and more stable catalysts, addressing challenges like framework collapse in zeolites. Expanding these strategies to other catalytic systems could revolutionize industrial processes, leading to more efficient and sustainable chemical transformations.

<References>
10.1021/acscatal.3c01945
10.1016/s1872-2067(23)64550-4
10.1002/anie.202313974
10.1021/ja501361v
</References>

42. Characterization and Analysis Methods -> Integrated Analysis Strategies: Data correlation methods

Data correlation methods for interface analysis are pivotal in integrating information from various sources, such as combining results from molecular simulations with experimental data. This approach ensures a more accurate and comprehensive understanding of interface phenomena [10.1021/acscatal.3c01945]. Additionally, these methods are crucial for aligning and consistently interpreting data from different techniques. Furthermore, data correlation methods involve integrating results from rigorous reaction tests, kinetic and isotopic probes, advanced characterization techniques, and theoretical methods. These combined approaches help correlate isotopic substitution effects and DFT calculations to understand reaction mechanisms [10.1016/s1872-2067(23)64550-4]. Moreover, efforts must be invested in developing realistic zeolite models and integrating multiple scales, from atomic to macroscopic, to address complex problems like mesopore formation [10.1016/j.trechm.2021.03.004]. Techniques such as statistical analysis, chemometrics, and multivariate data analysis are employed to enhance the reliability and accuracy of the results by correlating data from different analytical platforms [10.1039/c4cy01720j].

※※※

Integrative understanding and prospective outlook: Considering the advancements and findings discussed in the file, there lies an opportunity to explore emerging fields and innovative

methodologies. Future research endeavors might focus on developing more sophisticated data correlation methods that integrate multi-scale modeling and real-time monitoring techniques. The integration of advanced machine learning algorithms could further enhance the precision and interpretability of data correlations. Additionally, expanding the scope of isotopic probes and theoretical methods could provide deeper insights into reaction mechanisms and catalyst performance. Such innovations will not only improve our understanding of propane dehydrogenation catalysts but also pave the way for broader applications in catalysis and materials science.

<References>
10.1021/acscatal.3c01945
10.1016/s1872-2067(23)64550-4
10.1016/j.trechm.2021.03.004
10.1039/c4cy01720j
</References>

43. Characterization and Analysis Methods -> Integrated Analysis Strategies: Structure-property relationships

Structure-property relationships in propane dehydrogenation catalysts are established through a combination of advanced characterization techniques and theoretical modeling. Experimental studies provide kinetic data, isotope effects, and mechanistic insights that elucidate reaction pathways and the influence of catalyst properties on activity and selectivity [10.1016/s1872-2067(23)64550-4]. Theoretical methods, including density functional theory (DFT) calculations, help interpret these experimental findings by providing atomic-level details of the reaction mechanisms and the role of water, protons, and acid sites in facilitating or hindering specific steps [10.1021/acs.jpcc.7b10208].

Furthermore, operando characterizations offer dynamic insights into the atomic and electronic structures of heterogeneous catalysts under working conditions, which help to deeply understand the interfacial behavior and catalytic mechanism [10.1021/jacsau.1c00355]. Advanced analytical techniques such as high-resolution NMR spectroscopy further elucidate the local structures of distinct framework and extra-framework Al species, revealing their impact on catalytic activity [10.1021/acs.jpcc.4c02929].

※※※

Integrative understanding and prospective outlook: Considering the advancements and findings discussed in the file, there lies an opportunity to explore emerging fields and innovative methodologies. Future research endeavors might focus on integrating multiple characterization techniques with theoretical modeling to achieve a comprehensive understanding of structure-property relationships. Developing predictive models that can accurately forecast catalytic performance based on structural parameters could revolutionize catalyst design. Additionally, exploring the effects of environmental factors like solvent molecules on active sites and intermediate species could provide deeper insights into enhancing catalytic efficiency.

<References>
10.1016/s1872-2067(23)64550-4

10.1021/acs.jpcc.7b10208
10.1021/jacsau.1c00355
10.1021/acs.jpcc.4c02929
</References>

44. Characterization and Analysis Methods -> Integrated Analysis Strategies: Mechanistic investigations

Mechanistic investigations of propane dehydrogenation catalysts employ a variety of advanced characterization and analysis methods to elucidate the fundamental processes at play. These studies often utilize systematic approaches that combine rigorous reaction tests, kinetic and isotopic probes, advanced characterization techniques, and theoretical methods [10.1016/s1872-2067(23)64550-4]. For instance, isotopic exchange methods are employed to explore the reversibility and kinetic significance of elementary steps while maintaining the chemical steady state, providing undistorted insights into the nature and frequency of bound species and their binding sites [10.1021/acs.jpcc.3c04678].

Computational chemistry plays a crucial role in understanding the detailed mechanisms of complex chemical reactions at interfaces. It is also used alongside machine learning techniques to design new reactions and catalysts [10.1021/acs.jpclett.3c02233]. DFT computational methods are extensively used to calculate Gibbs free energy changes for various degradation pathways, confirming the effectiveness of introducing specific groups to enhance stability under harsh conditions [10.1002/anie.202400764].

In situ spectroscopy and modeling studies are pivotal for understanding the molecular details of reactions at solid-liquid interfaces. These tools help identify how solvents interact with active sites, influence elementary steps in catalytic cycles, and affect mass transfer and reaction rates [10.1021/acscatal.6b02532]. Additionally, ab initio molecular dynamics (AIMD) simulations provide dynamic interfacial structure information and possible proton shuffling mechanisms in water/metal oxide systems [10.1021/acs.jpcc.7b10208].

※※※

Integrative understanding and prospective outlook: Considering the advancements and findings discussed in the file, there lies an opportunity to explore emerging fields and innovative methodologies. Future research endeavors might focus on integrating multi-scale modeling with advanced spectroscopic techniques to provide a comprehensive view of catalytic processes. The development of operando modeling and machine learning techniques can significantly enhance our ability to predict and design more efficient catalysts. Moreover, exploring the impact of environmental factors, such as water presence, on catalytic performance can lead to breakthroughs in designing catalysts for sustainable and green chemical processes.

<References>
10.1016/s1872-2067(23)64550-4
10.1021/acs.jpcc.3c04678
10.1021/acs.jpclett.3c02233
10.1002/anie.202400764
10.1021/acscatal.6b02532

10.1021/acs.jpcc.7b10208
</References>

45. Industrial and Environmental Applications -> Sustainable Chemistry: Green chemistry processes

Propane dehydrogenation catalysts play a crucial role in sustainable chemistry, particularly when considering green chemistry processes at interfaces. The use of water as a solvent is increasingly favored over organic solvents due to its environmental friendliness, cost-effectiveness, and abundance [10.1016/s1872-2067(21)64032-9]. For instance, catalytic reactions performed in aqueous environments can leverage the unique properties of water to enhance the stability of catalysts and improve the efficiency of converting biomass into hydrocarbon fuels and valuable chemicals [10.1021/acscatal.7b03298]. Solid catalysts offer economic and environmental benefits by facilitating easy post-reaction separation, making them industrially preferable for processes such as dehydration steps in a biphasic system [10.1016/j.jcat.2011.01.013].

Interfaces are critical in green chemistry processes, addressing energy, resource, and environmental challenges across various applications like plating, corrosion prevention, batteries, fuel cells, and catalysis [10.1021/acs.jpclett.3c02233]. Zeolites, for example, have shown promise in transforming agricultural and municipal waste into valuable chemicals, with water playing a crucial role in influencing both catalyst performance and sustainability [10.1016/j.trechm.2021.03.004]. Additionally, supported metal catalysts such as Pd and Ni facilitate the hydro-upgrading of phenol into cyclohexanone and cyclohexanol, demonstrating the potential influence of solvents like water on reaction pathways [10.1021/ja501592y].

※※※

Integrative understanding and prospective outlook: Considering the advancements and findings discussed in the file, there lies an opportunity to explore emerging fields and innovative methodologies. Future research endeavors might focus on developing more efficient and stable catalysts that can operate under mild conditions and in aqueous phases. This approach not only minimizes undesirable thermal degradation reactions but also enhances targeted product selectivity and facilitates product separation from excess water. Moreover, integrating electrochemical water reduction with the reduction of biomass-derived feedstocks could significantly improve the utilization of renewable energy sources, thereby promoting sustainable chemical production. Additionally, exploring the role of functionalized zeolites and other solid catalysts in enhancing water tolerance and improving yields holds promise for advancing green chemistry practices.

<References>
10.1016/s1872-2067(21)64032-9
10.1021/acscatal.7b03298
10.1016/j.jcat.2011.01.013
10.1021/acs.jpclett.3c02233
10.1016/j.trechm.2021.03.004
10.1021/ja501592y
</References>

46. Industrial and Environmental Applications -> Sustainable Chemistry: Waste treatment applications

The literature primarily lacks specific information on waste treatment applications that utilize interface phenomena [10.1021/acssuschemeng.1c06401, 10.1016/s1872-2067(21)64032-9, 10.1021/acs.jpcc.1c10296, 10.1021/acscatal.7b03298, 10.1021/acs.jpcc.3c04678, 10.1016/j.jcat.2011.02.001, 10.1021/acscatal.2c03978, 10.1002/anie.202400764, 10.1016/j.trechm.2021.03.004, 10.1021/acs.jpcc.0c07033, 10.1016/j.jcat.2011.01.013, 10.1021/acs.jpclett.3c02233, 10.1016/j.apcatb.2015.05.050, 10.1021/jacsau.1c00319, 10.1016/j.jcat.2013.09.012, 10.1021/ja501592y, 10.1021/acscatal.7b04367, 10.1038/s41467-024-53475-z, 10.1134/s0965544120040143, 10.1021/acs.jpcc.4c02929, 10.1021/acscatal.2c01838, 10.1021/acs.jpcc.9b07738, 10.1021/jacsau.1c00355, 10.1016/j.jcat.2024.115562, 10.1021/acscatal.9b04637, 10.1016/j.jcat.2012.08.012, 10.1002/wcms.1272, 10.1016/j.apcatb.2015.09.027, 10.1021/acscatal.2c00594, 10.1016/j.jcat.2013.11.011, 10.1021/acs.jpcc.1c03846, 10.1016/j.jcat.2019.12.034, 10.1002/anie.200900404, 10.1038/ncomms14113]. However, some relevant insights can be inferred from the available data.

Waste treatment applications utilizing interface phenomena include processes where contaminants are selectively adsorbed, oxidized, or reduced at solid-liquid or liquid-liquid interfaces [10.1039/c4cy01720j]. These processes enhance the efficiency of waste treatment by concentrating pollutants at specific interfaces, making them easier to remove or degrade. For instance, catalytic wet air oxidation (CWAO) leverages the interface between gas and liquid phases to efficiently degrade organic pollutants [10.1016/s1872-2067(23)64550-4]. Additionally, CeO2 surfaces with their unique oxygen storage capability might be used in catalytic processes for waste treatment, where interfaces play a crucial role in enhancing reaction efficiency [10.1021/acs.jpcc.7b10208].

※※※

Integrative understanding and prospective outlook: Considering the advancements and findings discussed in the file, there is an opportunity to explore emerging fields and innovative methodologies. Future research endeavors might focus on developing novel catalysts and reactors designed specifically to leverage interface phenomena for enhanced mass transfer and selectivity in waste treatment processes. Additionally, integrating heterogeneous electrocatalysis, which describes electrochemical reactions at solid-liquid interfaces central to sustainable and renewable energy devices [10.1021/acs.jpcc.3c06870], could provide environmentally friendly removal and conversion of toxic species in processes such as denitrification. Moreover, exploring the use of hydrophobic zeolites and activated carbon for selective pollutant adsorption could lead to more efficient and targeted waste treatment strategies.

<References>

10.1021/acssuschemeng.1c06401
10.1016/s1872-2067(21)64032-9
10.1021/acs.jpcc.1c10296
10.1021/acscatal.7b03298
10.1021/acs.jpcc.3c04678

10.1016/j.jcat.2011.02.001
10.1021/acscatal.2c03978
10.1002/anie.202400764
10.1016/j.trechm.2021.03.004
10.1021/acs.jpcc.0c07033
10.1016/j.jcat.2011.01.013
10.1021/acs.jpclett.3c02233
10.1016/j.apcatb.2015.05.050
10.1021/jacsau.1c00319
10.1016/j.jcat.2013.09.012
10.1021/ja501592y,
10.1021/acscatal.7b04367
10.1038/s41467-024-53475-z
10.1134/s0965544120040143
10.1021/acs.jpcc.4c02929
10.1021/acscatal.2c01838
10.1021/acs.jpcc.9b07738
10.1021/jacsau.1c00355
10.1016/j.jcat.2024.115562
10.1021/acscatal.9b04637
10.1016/j.jcat.2012.08.012
10.1002/wcms.1272
10.1016/j.apcatb.2015.09.027
10.1021/acscatal.2c00594
10.1016/j.jcat.2013.11.011
10.1021/acs.jpcc.1c03846,
10.1016/j.jcat.2019.12.034
10.1002/anie.200900404
10.1038/ncomms14113
10.1039/c4cy01720j
10.1016/s1872-2067(23)64550-4
10.1021/acs.jpcc.7b10208
10.1021/acs.jpcc.3c06870
</References>

47. Industrial and Environmental Applications -> Sustainable Chemistry: Biomass conversion

Propane dehydrogenation catalysts play a significant role in the broader context of biomass conversion processes, particularly within the realm of sustainable chemistry. Interfaces are critical in facilitating the catalytic reactions necessary for converting biomass into valuable intermediates and chemicals. Biomass-derived feedstocks, often liquid phase and containing reactive oxygenates and water, require fundamentally new reaction pathways to be integrated into conventional processes designed for petroleum [10.1021/acssuschemeng.1c06401].

Interfaces facilitate these transformations by providing active sites for catalytic reactions on surfaces such as CeO_2, enhancing efficiency and selectivity under wet conditions [10.1021/acs.jpcc.7b10208]. The upgrading of biomass-derived compounds can occur directly in aqueous media or monophasic/biphasic mixtures, enabling distributed operations that leverage the ubiquity of water in raw biomass feedstocks [10.1016/s1872-2067(21)64032-9].

For instance, zeolites have been identified as efficient catalysts for aqueous-phase conversion of biomass-derived oxygenates into hydrocarbon fuels and value-added chemicals [10.1021/acs.jpcc.0c07033]. This approach is beneficial for addressing the poor volatility, high water solubility, and chemical instability of bio-oils, which are promising raw materials for liquid energy carriers and transportation fuels [10.1016/j.jcat.2011.02.001].

Additionally, interfaces support metal catalysts on oxide surfaces like SiO_2, ZrO_2, and HZSM-5, enabling cascade reactions such as hydrolysis, hydrogenolysis, dehydration, and hydrogenation to convert lignin-derived phenolic monomers into cycloalkanes and other products under mild conditions [10.1016/j.jcat.2013.09.012]. Hydrodeoxygenation (HDO) is also crucial for improving the energy density and stability of bio-oils, with supported metal catalysts used for the hydro-upgrading of phenol, a key component in these processes [10.1021/ja501592y].

※※※

Integrative understanding and prospective outlook: Considering the advancements and findings discussed in the file, there lies an opportunity to explore emerging fields and innovative methodologies. Future research endeavors might focus on developing more robust and selective catalysts tailored for biomass conversion. The integration of computational models to predict reaction kinetics and understand solvation effects will be pivotal. Moreover, the design of hierarchical zeolites to enhance mass transfer properties could address current limitations in catalytic conversions. Emphasizing sustainability, coupling electrochemical water reduction with reductive biomass conversion could further harness renewable energy sources, ensuring a greener future for both industrial and environmental applications.

<References>
10.1021/acssuschemeng.1c06401
10.1021/acs.jpcc.7b10208
10.1016/s1872-2067(21)64032-9
10.1021/acs.jpcc.0c07033
10.1016/j.jcat.2011.02.001
10.1016/j.jcat.2013.09.012
10.1021/ja501592y
</References>

48. Industrial and Environmental Applications -> Sustainable Chemistry: Environmental remediation

Interfaces play a crucial role in environmental remediation by facilitating the environmentally friendly removal and conversion of toxic species in processes like denitrification [10.1021/acs.jpcc.3c06870]. Solid-liquid interfaces enable the adsorption of contaminants like phenolics and carboxylic acids onto mineral surfaces, where they can be chemically transformed

or removed [10.1021/acscatal.6b02532]. Additionally, catalytic reactions on CeO2 surfaces help degrade pollutants or convert harmful substances into less toxic forms [10.1021/acs.jpcc.7b10208]. The interaction at solid-liquid or solid-gas interfaces allows for the selective removal of pollutants, improving the quality of natural resources and mitigating environmental impacts [10.1039/c4cy01720j].

Surface chemistry at the solid-liquid interface has been industrially relevant in plating, corrosion prevention, batteries, fuel cells, and catalysis, and its role is now becoming increasingly important in the fields of sustainable and green chemistries to solve energy, resource, and environment issues [10.1021/acs.jpclett.3c02233]. Moreover, water appears to favor primary reactions over secondary ones, as seen in the case of guaiacol-H2 reactions at Ru-aqueous interfaces, which influences carbon-oxygen bond cleavage over ring saturation [10.1016/s1872-2067(23)64550-4].

※※※

Integrative understanding and prospective outlook: Taking into consideration the advancements and findings discussed in the file, there lies an opportunity to explore emerging fields and innovative methodologies. Future research endeavors might focus on developing advanced materials with tailored interfacial properties for enhanced pollutant capture and degradation. Investigating the synergistic effects of multiple interfaces could lead to more efficient and robust environmental remediation technologies. Furthermore, integrating computational modeling with experimental studies can provide deeper insights into interfacial behavior and catalytic mechanisms, driving the development of next-generation remediation strategies.

<References>
10.1021/acs.jpcc.3c06870
10.1021/acscatal.6b02532
10.1021/acs.jpcc.7b10208
10.1039/c4cy01720j
10.1021/acs.jpclett.3c02233
10.1016/s1872-2067(23)64550-4
</References>

49. Industrial and Environmental Applications -> Energy Technologies: Electrocatalysis

Propane dehydrogenation catalysts are pivotal in industrial and environmental applications, particularly within the realm of energy technologies focusing on electrocatalysis. Interfaces play a critical role in electrocatalysis by facilitating efficient charge transfer between electrodes and electrolytes, enhancing the performance of electrochemical reactions [10.1021/acscatal.6b02532]. Specifically, these interfaces enable electrochemical reactions at solid-liquid boundaries, which are essential for sustainable and renewable energy devices such as fuel cells and batteries [10.1021/acs.jpcc.3c06870].

The design of solid-liquid interfaces aims to optimize the interaction between catalysts and reactants in the presence of solvents, ensuring efficient mass transport and reaction kinetics [10.1021/acscatal.6b02532]. Catalytic active sites at these interfaces can be influenced by solvent effects, leading to enhanced activity and selectivity. For instance, water can stabilize intermediates

and alter equilibrium states, impacting reaction mechanisms significantly [10.1016/j.jcat.2024.115562]. Strong electric fields in electrocatalysis can cause water dissociation due to the dipole moment of water molecules and the disruption of the hydrogen-bonded network near surfaces, forming reactive species such as OOH after electron transfer [10.1021/acscatal.9b04637].

Moreover, interfaces are applied in electrocatalysis by developing an understanding that benefits related electrochemical conversions. Similar catalytic materials and interfaces drive electrocatalytic hydrogenation and oxidation reactions in water, with high H* coverages, low temperatures, and optimized chemical/electrochemical potentials enhancing H2O2 selectivities in both electro- and thermal catalysis [10.1016/s1872-2067(23)64550-4]. In addition, molecular-level modeling of surface reactions in liquid solvent environments is crucial for understanding both thermal and electrocatalytic processes involved in energy conversion and storage applications [10.1021/ja501592y].

※※※

Integrative understanding and prospective outlook: Considering the advancements and findings discussed in the file, there lies an opportunity to explore emerging fields and innovative methodologies. Future research endeavors might focus on optimizing interfacial engineering to fine-tune molecular solvation behavior, thereby improving the efficiency of propane dehydrogenation catalysts. Additionally, investigating the impact of solvent effects on catalytic active sites could lead to breakthroughs in designing more selective and stable electrocatalysts. The integration of advanced spectroscopic techniques will provide deeper insights into the dynamic behavior of heterogeneous catalysts under working conditions, ultimately enhancing our ability to control catalyst redox and structure evolution. This forward-thinking approach will not only advance the field of electrocatalysis but also contribute significantly to sustainable and renewable energy solutions.

<References>
10.1021/acscatal.6b02532
10.1021/acs.jpcc.3c06870
10.1016/j.jcat.2024.115562
10.1021/acscatal.9b04637
10.1016/s1872-2067(23)64550-4
10.1021/ja501592y
</References>

50. Industrial and Environmental Applications -> Energy Technologies: Photocatalysis

Propane dehydrogenation catalysts are pivotal in industrial and environmental applications, particularly within the realm of energy technologies such as photocatalysis. Interfaces play a critical role in photocatalysis by facilitating the interaction between light, catalysts, and reactants, thereby enhancing charge separation and transfer efficiency, which is vital for improving photocatalytic performance [10.1080/08927022.2022.2049774]. Moreover, interfaces influence the structure and dynamics of water layers, affecting the generation and stabilization of reactive species essential for photocatalytic reactions [10.1021/acscatal.4c02514]. These interfaces also

prevent electron-hole recombination, which enhances the efficiency and selectivity of photocatalytic reactions, with water dissociating on the catalyst surface under illumination to form reactive species that stabilize the separated electron-hole pair, promoting photocatalytic activity [10.1021/acscatal.9b04637].

※※※

Integrative understanding and prospective outlook: Considering the advancements and findings discussed in the file, there lies an opportunity to explore emerging fields and innovative methodologies. Future research endeavors might focus on designing novel catalysts with tailored interfaces that enhance charge separation and transfer efficiency. Additionally, exploring sustainable processes through mechanisms such as charge separation and surface polarization can lead to more efficient and environmentally friendly photocatalytic systems. The integration of polarizable continuum models could further improve the description of charge separation and enable proper inclusion of surface polarization, advancing the field of electrochemistry and photocatalysis [10.1021/acscatal.2c00594].

<References>
10.1080/08927022.2022.2049774
10.1021/acscatal.4c02514
10.1021/acscatal.9b04637
10.1021/acscatal.2c00594
</References>

51. Industrial and Environmental Applications -> Energy Technologies: Fuel cell applications

In fuel cell applications, interfaces play a crucial role in optimizing the exchange of protons, electrons, and gases, thereby enhancing overall performance [10.1080/08927022.2022.2049774]. Interfaces facilitate proton transfer and water dissociation, which are essential for efficient electrochemical reactions. Water acts as a cocatalyst to provide or stabilize highly reactive species and serves as a hydrogen donor to enhance reactivity and selectivity. The formation of a microscale aqueous environment on the surface of heterogeneous catalysts promotes the transportation of hydrogen, protons, and oxygen at the solid-liquid interface [10.1021/acscatal.4c02514].

Additionally, interfaces in fuel cells ensure uniform distribution of reactant gases and facilitate electrochemical reactions at the gas diffusion layer and catalyst layer interfaces [10.1039/c4cy01720j]. They also manage electrochemical reactions, ensuring efficient proton or electron transfer at the catalyst/water interface [10.1021/acscatal.2c00594]. At the electrode-electrolyte interface, water molecules align with a specific orientation due to their dipole moment, facilitating proton transfer via a H-bonded network, which ensures efficient electrochemical reactions [10.1021/acscatal.9b04637].

Furthermore, interfaces like those between BaO and Ni can facilitate water-mediated carbon removal reactions, helping maintain anode activity by preventing coking and deactivation at operating temperatures [10.1002/wcms.1272]. These processes highlight the importance of interfacial phenomena in catalytic reactions, suggesting that similar principles apply to optimizing performance in fuel cells [10.1016/s1872-2067(21)64032-9].

※※※

Integrative understanding and prospective outlook: Taking into consideration the advancements and findings discussed in the file, there lies an opportunity to explore emerging fields and innovative methodologies. Future research endeavors might focus on developing advanced materials and designs for interfaces that can further enhance the efficiency and durability of fuel cells. Investigating novel catalysts that can improve proton and electron transfer while reducing the risk of coking and deactivation could lead to breakthroughs in fuel cell technology. Additionally, modeling and simulation techniques can be employed to better understand and optimize the complex interactions at these interfaces, paving the way for more efficient and sustainable energy solutions.

<References>
10.1080/08927022.2022.2049774
10.1021/acscatal.4c02514
10.1039/c4cy01720j
10.1021/acscatal.2c00594
10.1021/acscatal.9b04637
10.1002/wcms.1272
10.1016/s1872-2067(21)64032-9
</References>

52. Industrial and Environmental Applications -> Energy Technologies: Energy storage systems

Important interface phenomena in energy storage systems encompass a variety of processes that significantly influence the efficiency, capacity, and longevity of these systems. Ion diffusion, electron transfer, and surface-mediated reactions are crucial for determining the rate and effectiveness of charge/discharge cycles [10.1080/08927022.2022.2049774]. Additionally, proton transfer and water dissociation play pivotal roles by affecting the transportation of hydrogen and protons, thereby impacting system performance [10.1021/acscatal.4c02514]. The solid-electrolyte interphase (SEI) is another critical factor, as it stabilizes lithium ions and prevents their recombination with electrons, thus maintaining battery capacity and longevity [10.1021/acscatal.9b04637].

※※※

Integrative understanding and prospective outlook: Considering the advancements highlighted in the literature, there is a significant opportunity to explore novel materials and designs that enhance ion diffusion, electron transfer, and surface chemistry at the electrode-electrolyte interface. Future research could focus on developing advanced SEI layers to improve stability and efficiency, as well as investigating new electrolytes that facilitate faster ion transport. Additionally, understanding the fundamental mechanisms of proton transfer and water dissociation can lead to breakthroughs in improving the overall performance of energy storage systems. This forward-thinking approach will pave the way for more efficient and durable batteries and supercapacitors, ultimately contributing to sustainable and renewable energy technologies.

<References>

10.1080/08927022.2022.2049774
10.1021/acscatal.4c02514
10.1021/acscatal.9b04637
</References>

53. Industrial and Environmental Applications -> Process Optimization: Catalyst stability

Catalyst stability at interfaces in propane dehydrogenation processes is a critical factor for maintaining sustained catalytic activity and efficiency over time. The stability of catalysts, particularly supported Ru clusters, can be influenced by both irreversible and reversible modes of deactivation [10.1016/j.jcat.2015.04.026]. Irreversible deactivation primarily results from particle sintering, which is exacerbated by the electronegativity of the support material [10.1016/j.jcat.2015.04.026]. Reversible deactivation, while not fully understood, appears to depend on the support material and the presence of carboxylic acids, correlating with the surface charge of the support [10.1016/j.jcat.2015.04.026].

Water plays a pivotal role in maintaining catalyst stability through mechanisms such as carbon removal, preventing deactivation due to coking. However, excessive water can lead to the conversion of MoMo bonds in small Mo2C clusters to MoO bonds, potentially causing a sudden drop in activity [10.1016/s0021-9517(03)00236-7]. Additionally, water dissociation at the metal-support interface promotes metal/support interactions, enhancing stability and influencing other industrial chemical reactions [10.1021/acscatal.4c02514].

Hydrophobic functionalization is an effective strategy to enhance the water tolerance of hydrophilic catalysts, reducing unreacted silanol density and inhibiting the leaching process [10.1021/acscatal.9b04637]. Moreover, minimizing defect sites such as isolated SiOH and external SiOH can prevent framework disintegration, further contributing to catalyst stability [10.1134/s0965544120040143].

※※※

Integrative understanding and prospective outlook: Considering the advancements and findings discussed in the file, there lies an opportunity to explore emerging fields and innovative methodologies. Future research endeavors might focus on developing novel support materials that minimize both reversible and irreversible deactivation pathways. Investigating the impact of varying water concentrations on catalyst stability could lead to optimized reaction conditions that balance activity and longevity. Additionally, exploring advanced surface modifications and protective coatings can provide new avenues for enhancing catalyst durability. Hydrothermal stability remains a critical area for improvement, especially for zeolite-based catalysts used in biomass conversion processes. Understanding the dynamic nature of zeolites under reaction conditions can guide the design of more robust and efficient catalysts. Furthermore, detailed atomistic descriptions of the water/liquid interface can offer insights into the activation energies of catalytic reactions, paving the way for more stable and selective catalysts.

<References>
10.1016/j.jcat.2015.04.026
10.1016/s0021-9517(03)00236-7
10.1021/acscatal.4c02514

10.1021/acscatal.9b04637
10.1134/s0965544120040143
</References>

54. Industrial and Environmental Applications -> Process Optimization: Reaction engineering

Propane dehydrogenation catalysts play a crucial role in industrial and environmental applications, where reaction engineering principles significantly influence process optimization. Key principles include mass and heat transfer optimization, reaction kinetics analysis, and reactor design considerations [10.1080/08927022.2022.2049774]. Efficient mass transfer across interfaces ensures high reaction rates, while proper heat management prevents thermal deactivation of catalysts [10.1039/c4cy01720j]. Kinetic models help predict and control reaction behavior, and optimal reactor design maximizes product yield.

Additionally, understanding the configuration of water molecules above reaction sites and the strength of solid-liquid interactions dictate proton transfer mechanisms [10.1021/acscatal.4c02514]. Transition state theory concepts are necessary to explain how reactant concentrations versus thermodynamic activities influence the reaction rate [10.1021/acscatal.9b04637]. Ensuring efficient mixing and contact between phases is crucial for enhancing reaction rates and yields at interfaces [10.1016/s1872-2067(21)64032-9].

※※※

Integrative understanding and prospective outlook: Taking into consideration the advancements and findings discussed in the file, there lies an opportunity to explore emerging fields and innovative methodologies. Future research endeavors might focus on developing advanced computational tools like DFT to accelerate the development of improved electrode materials and enhance process efficiency [10.1021/acs.jpcc.3c06870]. Furthermore, integrating electrocatalytic systems can allow heterogeneous catalytic systems to adopt some benefits of homogeneous catalytic systems, such as enhanced reactivity and selectivity [10.1016/j.cossms.2006.03.007]. Exploring these areas will likely lead to more efficient and environmentally friendly propane dehydrogenation processes.

<References>
10.1080/08927022.2022.2049774
10.1039/c4cy01720j
10.1021/acscatal.4c02514
10.1021/acscatal.9b04637
10.1016/s1872-2067(21)64032-9
10.1021/acs.jpcc.3c06870
10.1016/j.cossms.2006.03.007
</References>

55. Industrial and Environmental Applications -> Process Optimization: Scale-up considerations

Scale-up considerations for propane dehydrogenation catalysts are critical to ensuring consistent performance across larger scales while maintaining process efficiency and product quality. Key factors include uniformity of material properties, scalability of reactors, and robustness of operational parameters [10.1080/08927022.2022.2049774]. Maintaining consistent performance metrics from lab-scale to industrial-scale operations is essential. This involves ensuring uniform mixing, minimizing mass transfer limitations, and preserving catalyst activity [10.1039/c4cy01720j].

The translation of fundamental knowledge gained from ab initio simulations and surface imaging technologies into industrial-scale operations is crucial. It ensures that the desired properties of the catalyst and the efficiency of the reactions are maintained [10.1021/acscatal.4c02514]. Additionally, scaling up requires robust process control systems to handle increased throughput and variability [10.1039/c4cy01720j].

Ensuring that the enhanced catalytic activity observed at small scales can be maintained during industrial-scale operations is vital. Factors such as mass transfer, heat transfer, and catalyst stability must be carefully managed to preserve the benefits of interface phenomena [10.1021/acscatal.9b04637]. Challenges related to maintaining optimal conditions across larger volumes include ensuring uniform temperature and concentration profiles, minimizing mass transfer limitations, and scaling up reactor designs while preserving process efficiency and selectivity [10.1016/s1872-2067(21)64032-9].

Mass transport classification as external or internal and understanding the balance between diffusion rates and intrinsic reaction rates are also important. Diffusion-limited reactions occur when diffusion is slower than the intrinsic reaction rate, while reaction-limited scenarios occur when diffusion is sufficiently fast. Intermediate conditions also exist, and activation energies provide insights into whether mass transport or intrinsic kinetics dominate [10.1021/acscatal.6b02532].

※※※

Integrative understanding and prospective outlook: Considering the advancements and findings discussed in the file, there lies an opportunity to explore emerging fields and innovative methodologies. Future research endeavors might focus on developing advanced modeling techniques that accurately reflect real-world conditions, especially regarding solvent effects and interfacial solvation. Enhancing catalyst dispersion and activity through novel materials and processing methods could lead to more efficient and environmentally friendly processes. Moreover, integrating computational studies with experimental validation will be crucial for successful scale-up from laboratory to industrial settings.

<References>
10.1080/08927022.2022.2049774
10.1039/c4cy01720j
10.1021/acscatal.4c02514
10.1021/acscatal.9b04637
10.1016/s1872-2067(21)64032-9
10.1021/acscatal.6b02532
</References>

56. Industrial and Environmental Applications -> Process Optimization: Economic factors

Economic factors significantly influence the implementation of interface technology in propane dehydrogenation catalysts. The cost-effectiveness of materials, scalable production methods, and market demand for high-performance devices drive the adoption of advanced interface technologies [10.1080/08927022.2022.2049774]. Economic considerations also determine the feasibility of using advanced simulation methods and surface imaging technologies, which are crucial for optimizing catalyst performance under real-world conditions [10.1021/acscatal.4c02514].

Furthermore, economic factors affect the cost of materials, process efficiency, and overall operational expenses, requiring careful evaluation to ensure economic viability [10.1021/acscatal.4c02514]. Technologies that offer higher efficiency and lower costs are more likely to be adopted [10.1039/c4cy01720j]. The choice of materials, process conditions, and operational strategies must balance initial investment with long-term benefits to ensure economic viability [10.1021/acscatal.9b04637].

Cost-effective solutions that balance capital investment with operating costs and product quality are sought to ensure viable and sustainable industrial applications [10.1016/s1872-2067(21)64032-9]. Economic factors influence interface technology implementation by determining the cost-effectiveness of using advanced modeling techniques and experimental methods to study and optimize interface phenomena [10.1021/acscatal.2c00594]. Challenges in modeling electrochemical and electrocatalytic systems, such as accounting for local ions, double-layer dynamics, and statistical sampling, require sophisticated tools and methods, which can be costly [10.1016/j.cossms.2006.03.007].

※※※

Integrative understanding and prospective outlook: Considering the advancements and findings discussed in the file, there lies an opportunity to explore emerging fields and innovative methodologies. Future research endeavors might focus on developing cost-effective and scalable processes for producing advanced propane dehydrogenation catalysts. By integrating advanced modeling techniques and surface imaging technologies, researchers can better understand and optimize catalyst performance under real-world conditions. Additionally, addressing challenges in modeling electrochemical and electrocatalytic systems will enhance the feasibility and adoption of interface technologies. The pursuit of economically viable solutions will not only promote industrial sustainability but also contribute to broader environmental goals.

<References>
10.1080/08927022.2022.2049774
10.1021/acscatal.4c02514
10.1039/c4cy01720j
10.1021/acscatal.9b04637
10.1016/s1872-2067(21)64032-9
10.1021/acscatal.2c00594
10.1016/j.cossms.2006.03.007
</References>

57. Future Perspectives and Challenges -> Fundamental Research Needs: Interface dynamics understanding

The key challenges in understanding interface dynamics, particularly in propane dehydrogenation catalysts, revolve around the complex interactions at solid-liquid interfaces. These include deciphering the atomistic and molecular origins of interfacial behaviors and leveraging this knowledge to enhance catalytic efficiency for chemical transformations [10.1016/s1872-2067(21)64032-9]. The response of solid acid and base surfaces to water remains crucial for catalysis, with variations in ion density within the interfacial region significantly impacting molecular reactivity through local ionic strength effects [10.1016/s1872-2067(21)64032-9].

Furthermore, the limited fundamental knowledge about water/solid interfaces compared to solid/gas interfaces and bulk water poses significant challenges [10.1021/acscatal.2c00594]. Unresolved questions remain regarding water structuring, potential water dissociation at the interface, and the impact of applied electrochemical potentials on interfacial structures. Standard surface science techniques are not easily applicable to solid-liquid interfaces [10.1021/acscatal.2c00594].

Additionally, the complexities of solvent effects on adsorption and protonation within porous catalysts complicate our understanding. Solvents can alter the energetics of substrates, intermediates, and transition states, affecting reaction rates and selectivities [10.1038/s41467-020-14860-6]. Predictive models for solvent selection are lacking, necessitating both experimental and computational advancements.

※※※

Integrative understanding and prospective outlook: Considering the advancements and findings discussed, there lies an opportunity to explore emerging fields and innovative methodologies. Future research endeavors might focus on developing advanced characterization techniques that can probe solid-liquid interfaces in situ, providing real-time insights into catalytic processes. Additionally, integrating multiscale modeling approaches could help bridge the gap between atomic-level phenomena and macroscopic properties, leading to more effective catalyst design. Moreover, understanding the dynamic evolution of zeolite frameworks and metal-zeolite interfaces under reaction conditions will be critical for identifying active centers and mechanisms. This holistic approach will foster a deeper understanding of interface dynamics, ultimately enhancing the efficiency and selectivity of propane dehydrogenation catalysts.

<References>
10.1016/s1872-2067(21)64032-9
10.1021/acscatal.2c00594
10.1038/s41467-020-14860-6
</References>

58. Future Perspectives and Challenges -> Fundamental Research Needs: Structure-function relationships

The literature emphasizes the importance of gaining sufficient knowledge about the states of surfaces, particularly active sites, in the absence of bulk and interfacial water, and how these states transform as water increases [10.1016/s1872-2067(21)64032-9]. Water's interactions with solid materials drive reversible and irreversible changes, leading to significant catalytic responses. Understanding the response of solid acid and base surfaces to water is essential for this comprehension. Additionally, structure-function relationships can be better understood through first-principles Density Functional Theory (DFT) calculations [10.1021/acs.jpcc.3c06870], which provide insights into the molecular mechanisms underlying electrocatalyst performance and have accelerated the development of improved electrode materials. Furthermore, advancements in ab initio simulation methods and surface imaging technologies like STM have significantly contributed to our understanding of water at various interfaces [10.1021/acscatal.4c02514].

To better understand structure-function relationships, further research is needed to explore how structural changes during reactions affect catalytic performance [10.1021/acscatal.2c01233]. This involves studying the dynamic behavior of zeolites and metal-zeolites under reaction conditions to link specific structural features with their catalytic functions. Moreover, operando characterizations that provide real-time insights into atomic and electronic structures under working conditions help establish structure/composition-performance correlations [10.1021/jacsau.1c00355].

Structure-function relationships can also be better understood by addressing the challenges associated with elucidating these relationships at solid-liquid interfaces [10.1021/acscatal.0c05674]. This involves studying the solvation of reacting substrates, their interactions with catalysts, the dynamic nature of active sites, and the subtle variations in reaction pathways. Systematic approaches combining rigorous reaction tests, kinetic and isotopic probes, advanced characterization techniques, and theoretical methods are crucial for deeper insights [10.1016/s1872-2067(23)64550-4].

※※※

Integrative understanding and prospective outlook: Considering the advancements and findings discussed in the file, there lies an opportunity to explore emerging fields and innovative methodologies. Future research endeavors might focus on developing predictive models that link material properties with catalytic performance, leveraging advanced computational methods like DFT and AIMD simulations to provide atomic-level insights into catalytic activity and stability. Investigating the dynamic transformations of catalysts during reactions will be essential, moving beyond traditional static models to capture the true complexity of catalytic processes. Additionally, integrating operando characterizations with theoretical investigations will offer real-time, dynamic insights into the atomic and electronic structures of catalysts under working conditions. Addressing the complexities of solid-liquid interfaces, including the solvation of reacting substrates and the dynamic nature of active sites, will pave the way for a more profound understanding of structure-function relationships. These efforts will ultimately enhance our ability to design and optimize propane dehydrogenation catalysts for industrial applications.

<References>

10.1016/s1872-2067(21)64032-9
10.1021/acs.jpcc.3c06870
10.1021/acscatal.4c02514
10.1021/acscatal.2c01233

10.1021/jacsau.1c00355
10.1021/acscatal.0c05674
10.1016/s1872-2067(23)64550-4
</References>

59. Future Perspectives and Challenges -> Fundamental Research Needs: Reaction mechanism insights

Insights into the reaction mechanisms of propane dehydrogenation catalysts are essential for optimizing catalytic performance and developing new materials. Characterizing the hydronium ion within zeolites reveals that confined hydronium ions exhibit different catalytic activities compared to those in bulk liquid phases, as supported by theoretical simulations [10.1021/jacs.8b07969]. Understanding how reactants and water interact with acid sites and substrates in confined solvents is critical for a molecular description of reaction mechanisms [10.1021/jacs.8b07969].

The presence of water significantly affects reaction pathways. For instance, increasing water vapor pressure leads to the formation of protonated alcohol-water dimers and clusters, ultimately resulting in unproductive water-only clusters [10.1016/s1872-2067(21)64032-9]. This phenomenon impacts the stability of intermediates and overall reaction activity, as seen in cyclohexanol dehydration where local water concentration influences whether E1 or E2 mechanisms are favored [10.1021/acs.jpcc.9b07738].

Computational chemistry plays a pivotal role in elucidating atomistic mechanisms. Efforts have been dedicated to achieving a full atomistic description of the water/liquid interface, which allows for evaluating activation energies of catalytic reactions and catalyst decomposition [10.1021/acscatal.2c00594]. Additionally, insights into dynamic transformations of catalysts during reactions suggest that static models do not fully capture changes occurring during a catalytic cycle, necessitating dynamic approaches [10.1021/acscatal.2c01838].

※※※

Integrative understanding and prospective outlook: Considering the advancements and findings discussed in the file, there lies an opportunity to explore emerging fields and innovative methodologies. Future research endeavors might focus on:

1. **Dynamic Modeling and Simulation**: Developing advanced computational tools to simulate real-time structural changes in catalysts under reaction conditions can provide deeper insights into the dynamic nature of active sites.

2. **Interfacial Reactions**: Investigating the effects of solvent interactions on reaction mechanisms, particularly focusing on proton-coupled electron transfer and hydrogen-bonding interactions, can lead to the design of more efficient catalysts.

3. **Water Influence**: Elucidating the exact roles of water in stabilizing intermediates and influencing kinetic barriers will be crucial for enhancing catalytic efficiency.

4. **Material Design**: Exploring novel zeolite structures that maximize water-promoting effects could facilitate the development of more efficient metal-containing catalysts for various challenging reactions.

<References>
10.1021/jacs.8b07969

10.1016/s1872-2067(21)64032-9
10.1021/acs.jpcc.9b07738
10.1021/acscatal.2c00594
10.1021/acscatal.2c01838
</References>

60. Future Perspectives and Challenges -> Fundamental Research Needs: New material development

The development of propane dehydrogenation catalysts has seen significant advancements, yet challenges remain in creating materials that enhance catalytic activity and selectivity under various conditions. Directions for new material development include exploring materials that can stabilize reactive intermediates and transition states within extended hydrogen-bonded water networks [10.1016/s1872-2067(21)64032-9]. Additionally, synthesizing small-pore zeolites with varying Si/Al ratios using different organic additives offers a promising path [10.1016/j.apcatb.2021.120244]. Zeolite HBEA150, with its specific physicochemical properties, demonstrates significantly higher turnover frequencies for alcohol dehydration compared to aqueous-phase hydronium ions, suggesting potential for advanced inorganic catalysts [10.1038/ncomms14113]. Furthermore, integrating acidity, hydrophobicity, and confinement into solid materials through functionalization techniques, such as sulfonic acid-functionalized zeolites, presents unique approaches as water-tolerant, strong Bronsted acid catalysts suitable for biomass upgrading reactions [10.1021/acssuschemeng.1c06401]. Insights from the structural reactivity relationship on Pd/Beta zeolite catalysts have guided the development of new materials like Pd/MWW zeolite catalysts, which offer superior performance for activating challenging substrates [10.1038/s41467-024-53475-z].

※※※

Integrative understanding and prospective outlook: Considering the advancements and findings discussed in the file, there lies an opportunity to explore emerging fields and innovative methodologies. Future research endeavors might focus on developing novel catalysts that not only enhance catalytic activity and selectivity but also maintain stability under harsh conditions. Exploring the dynamic evolution of zeolites and metal-zeolites could guide the synthesis, modification, and application of more effective catalysts [10.1021/acscatal.2c01233]. Additionally, leveraging atomic-scale information gained from molecular modeling and exploring joint multiscale modeling approaches combined with advanced in situ or operando techniques will be crucial for rationally designing zeolites with optimized hydrothermal stability and catalytic activity [10.1016/j.trechm.2021.03.004]. Innovations in coating methods, such as electron beam evaporation, atomic layer deposition, and evaporation-driven self-assembly, can ensure that catalysts retain their integrity and performance in water [10.1039/c4cy01720j]. These strategies will pave the way for more efficient and robust catalysts, ultimately driving progress in propane dehydrogenation processes.

<References>
10.1016/s1872-2067(21)64032-9
10.1016/j.apcatb.2021.120244

10.1038/ncomms14113
10.1021/acssuschemeng.1c06401
10.1038/s41467-024-53475-z
10.1021/acscatal.2c01233
10.1016/j.trechm.2021.03.004
10.1039/c4cy01720j
</References>

61. Future Perspectives and Challenges -> Technological Advances: Novel characterization methods

Novel characterization methods are revolutionizing the study of propane dehydrogenation catalysts by providing unprecedented insights into their dynamic behavior and structural evolution under reaction conditions. Advanced spectroscopic techniques, such as in situ NMR and IR spectroscopy combined with density functional theory (DFT) calculations, allow researchers to investigate proton mobility and the chemical environment over a wide range of pressures and temperatures [10.1021/jacs.8b07969]. Additionally, the population decay correlation function of H_3O^+ is calculated to understand its dynamic properties [10.1021/jacs.8b07969].

Solid-state NMR spectroscopy is employed to monitor the evolution of acid site structures during hydration processes [10.1021/jacs.8b07969], while advanced spectroscopic methods enable following the dynamic evolution of heterogeneous catalytic solids in situ within reactors, improving spatial and temporal resolution [10.1021/acscatal.3c01945]. Complementary techniques link theoretical observations with experiments, such as the pulse-response temporal analysis of products (TAP) methodology, which measures the residence times of probe molecules [10.1021/acscatal.3c01945].

In situ characterization techniques for solid acid catalysts in liquid environments include potentiometric acid-base titration and ion exchange methods that quantify Brønsted acid sites (BAS) in liquid water [10.1016/s1872-2067(21)64032-9]. Other methods like attenuated total reflectance infrared (ATR-IR) spectroscopy, titration of catalytic sites during reactions, and modified temperature-programmed desorption of base molecules also serve this purpose [10.1016/s1872-2067(21)64032-9].

Calorimetry, inelastic neutron scattering, nonlinear optical spectroscopies, and surface-sensitive X-ray diffraction methods provide atomic-level insights into the structural and dynamic behaviors of aqueous phase/CeO_2 interfaces [10.1021/acscatal.2c00594]. Techniques such as ab initio molecular dynamics (AIMD) simulations are used to study these interfaces [10.1021/acs.jpcc.7b10208].

Extended X-ray absorption fine structure (EXAFS) and Al magic angle spinning NMR spectroscopy offer detailed information about hydronium ion formation in zeolites and the adsorption behavior of reactants [10.1038/ncomms14113]. In situ Al K-edge XANES tracks structural changes in zeolites as water is removed with increasing temperature, and time-dependent density functional theory (TDDFT) calculations predict XANES spectral features accurately [10.1021/acs.chemmater.7b02133].

※※※

Integrative understanding and prospective outlook: Considering the advancements and findings discussed in the file, there lies an opportunity to explore emerging fields and innovative methodologies. Future research endeavors might focus on developing more sophisticated in situ and operando techniques to monitor catalytic changes under realistic reaction conditions. The integration of advanced computational tools like DFT and AIMD will enhance our ability to model complex catalytic systems at the atomic level. Furthermore, combining multiple spectroscopic methods can provide comprehensive insights into both static and dynamic aspects of catalysts, leading to better design strategies for enhancing catalytic performance. These advancements could pave the way for more efficient and sustainable propane dehydrogenation processes.

<References>
10.1021/jacs.8b07969
10.1021/acscatal.3c01945
10.1016/s1872-2067(21)64032-9
10.1021/acscatal.2c00594
10.1021/acs.jpcc.7b10208
10.1038/ncomms14113
10.1021/acs.chemmater.7b02133
</References>

62. Future Perspectives and Challenges -> Technological Advances: Improved computational approaches

The field of propane dehydrogenation catalysts is witnessing significant advancements in computational approaches, enhancing the understanding and optimization of catalytic mechanisms. Systematic NMR and IR spectroscopic studies combined with density functional theory (DFT)-based and ab initio molecular dynamics calculations provide insights into the chemical environment and proton mobility over a wide range of conditions [10.1021/jacs.8b07969]. Periodic DFT-based ab initio molecular dynamics simulations, utilizing GGA with PBE exchange-correlation functional and Grimme's second-generation dispersion corrections within the CP2K package, offer detailed atomic-level insights [10.1021/acscatal.3c01945]. Additionally, first-principles MD simulations, when coupled with enhanced sampling techniques, efficiently sample low-probability regions of phase space, discovering competitive pathways and exploring broad transition state regions [10.1021/acs.jpcc.9b07738].

Implicit solvent models or polarizable continuum models (PCM) have also seen increased use for stabilizing charged species in electrocatalysis, where cavitation energy is generally negligible compared to the electrostatic interaction [10.1021/acscatal.2c00594]. Density functional theory (DFT) calculations are employed to model hydronium ion catalyzed pathways in HBEA, providing insights into energetics and kinetics by comparing E1 and E2-type elimination paths [10.1038/ncomms14113]. Furthermore, rigorous kinetic analysis, in situ IR spectroscopy, metadynamics, and AIMD simulations elucidate rate inhibition mechanisms and stabilization of

reactive intermediates in aqueous-phase Brønsted acid catalysis [10.1016/s1872-2067(21)64032-9].

※※※

Integrative understanding and prospective outlook: Considering the advancements discussed, future research could focus on integrating multiscale modeling and machine learning techniques to achieve operando modeling, simulating reaction conditions more accurately [10.1021/jacsau.1c00355]. The development of well-calibrated multilevel quantum mechanical (QM) and molecular mechanical (MM) methods with accurate free-energy estimators promises to bridge the gap between current models and true operando modeling [10.1021/acscatal.7b04367]. Moreover, there is potential for exploring the role of water in modifying electronic structures and participating in surface reactions via proton transfer and exchange pathways [10.1021/ja501592y], which could lead to innovative strategies for designing more efficient propane dehydrogenation catalysts.

<References>
10.1021/jacs.8b07969
10.1021/acscatal.3c01945
10.1021/acs.jpcc.9b07738
10.1021/acscatal.2c00594
10.1038/ncomms14113
10.1016/s1872-2067(21)64032-9
10.1021/jacsau.1c00355
10.1021/acscatal.7b04367
10.1021/ja501592y
</References>

63. Future Perspectives and Challenges -> Technological Advances: Advanced catalyst designs

Advances in catalyst design for propane dehydrogenation have seen significant developments, particularly in understanding the role of confined hydronium ions. These ions exhibit different catalytic activities compared to those in bulk liquid phases, as shown by studies that enhance knowledge of hydronium ion structures in zeolites and their impact on reaction mechanisms in confined spaces [10.1021/jacs.8b07969]. Additionally, aluminosilicate zeolites with strong acidity, robust hydrothermal stability, and enzyme-like host-guest interactions offer promising materials for enhancing catalytic efficiency and regioselectivity in coupling phenolics and low-value components [10.1038/s41929-017-0015-z].

Tailoring nanoscopic confines to maximize catalytic activity and developing machine learning potentials for metal-organic frameworks using incremental learning approaches have also been critical advancements [10.1021/acscatal.3c01945]. Moreover, leveraging zeolite pores has been shown to significantly increase the rate of hydronium ion-catalyzed reactions, leading to higher turnover frequencies for alcohol dehydration compared to aqueous-phase hydronium ions [10.1038/ncomms14113].

Efforts to achieve a full atomistic description of the water/liquid interface allow for the evaluation of activation energies of catalytic reactions and catalyst decomposition

[10.1021/acscatal.2c00594]. Studies examining the interaction of the SiOHAl group with water at varying temperatures provide insights into the reversible formation of zeolite Brønsted acid sites and hydrated hydronium ions [10.1021/acs.chemmater.7b02133].

Furthermore, water plays a promoting role in NH3-SCR over Cu-LTA catalysts at both high and low temperatures, highlighting the importance of understanding the role of water in facilitating copper ion migration within the catalyst structure [10.1016/j.apcatb.2021.120244]. Catalyst design also involves optimizing catalysts and processes based on fundamental insights gained from studying the roles of water molecules and ionic species dissolved in them [10.1016/s1872-2067(21)64032-9].

※※※

Integrative understanding and prospective outlook: Considering the advancements discussed, there is an opportunity to explore emerging fields and innovative methodologies. Future research could focus on integrating advanced computational models with experimental data to predict and optimize catalyst performance under various conditions. The development of hybrid catalysts combining the benefits of multiple active sites and confinement effects may lead to breakthroughs in catalytic efficiency and selectivity. Additionally, exploring the synergistic effects of co-feeding water and other additives can enhance catalytic performance and longevity, paving the way for more sustainable and efficient industrial processes.

<References>
10.1021/jacs.8b07969
10.1038/s41929-017-0015-z
10.1021/acscatal.3c01945
10.1038/ncomms14113
10.1021/acscatal.2c00594
10.1021/acs.chemmater.7b02133
10.1016/j.apcatb.2021.120244
10.1016/s1872-2067(21)64032-9
</References>

64. Future Perspectives and Challenges -> Technological Advances: Process intensification

The current landscape of propane dehydrogenation catalysts is characterized by an increasing emphasis on enhancing catalytic performance through innovative materials and reactor designs [10.1021/acscatal.2c00594]. Despite the absence of direct discussions on process intensification, the literature underscores the importance of developing catalysts with higher activity, selectivity, and stability to meet industrial demands [10.1021/acs.jpclett.3c02233].

Process intensification, although not explicitly detailed, is inherently linked to these advancements. The integration of novel catalysts into intensified processes aims to achieve more efficient and sustainable production methods. Challenges remain in translating laboratory-scale successes into industrial applications, particularly concerning scalability and cost-effectiveness [10.1016/s1872-2067(23)64550-4].

※※※

Integrative understanding and prospective outlook: Considering the advancements and findings discussed in the file, there lies an opportunity to explore emerging fields and innovative methodologies. Future research endeavors might focus on the development of multifunctional catalysts capable of performing multiple reactions simultaneously, thereby enhancing process efficiency. Additionally, the application of advanced modeling and simulation tools could provide deeper insights into reaction mechanisms, aiding in the design of more effective intensified processes. The convergence of materials science, chemical engineering, and computational techniques will likely play a pivotal role in overcoming existing challenges and driving forward the field of propane dehydrogenation.

<References>
10.1021/acscatal.2c00594
10.1021/acs.jpclett.3c02233
10.1016/s1872-2067(23)64550-4
</References>

The current literature does not provide insights into the implementation of process intensification in propane dehydrogenation catalysts. Despite the extensive research on catalyst development and performance optimization, the integration of process intensification techniques remains underexplored. This gap highlights the need for future research to address the challenges associated with enhancing the efficiency and sustainability of propane dehydrogenation processes.

※※※

Integrative understanding and prospective outlook: Considering the advancements and findings discussed in the file, there lies an opportunity to explore emerging fields and innovative methodologies. Future research endeavors might focus on integrating process intensification strategies such as microreactors, catalytic membranes, and reactive distillation to enhance the selectivity and yield of propane dehydrogenation. Additionally, the development of novel catalysts with improved thermal stability and activity could further intensify the process, leading to more sustainable and economically viable industrial applications.

<References>
10.1021/acscatal.2c00594
10.1021/acs.jpclett.3c02233
10.1016/s1872-2067(23)64550-4
10.1021/acs.jpcc.3c06870
</References>

65. Future Perspectives and Challenges -> Emerging Opportunities: New application areas

Interfaces play a crucial role in emerging application areas such as biomass upgrading, electrocatalysis, and photocatalysis. These areas benefit from reactions occurring at the water/solid interface which can lead to advancements in sustainable technologies [10.1021/acscatal.2c00594]. Interface technology is finding new application areas in plating, corrosion prevention, batteries, fuel cells, and catalysts. Its importance is growing in sustainable

and green chemistry fields to address energy, resource, and environmental challenges [10.1021/acs.jpclett.3c02233]. Emerging application areas for interface technology include aqueous-phase catalytic reactions, which are becoming increasingly important for the sustainable transformation of energy carriers and chemicals. These reactions often occur at metal-aqueous interfaces and benefit from the unique properties of water as a solvent and reactant [10.1016/s1872-2067(23)64550-4].

※※※

Integrative understanding and prospective outlook: Considering the advancements and findings discussed in the file, there lies an opportunity to explore emerging fields and innovative methodologies. Future research endeavors might focus on expanding the application of interface technology into more sustainable processes. Specifically, the development of advanced catalysts for biomass upgrading, electrocatalysis, and photocatalysis could significantly enhance the efficiency and sustainability of these processes. Additionally, exploring the potential of aqueous-phase catalytic reactions can offer benefits such as lower energy input and minimized degradation of feedstocks, thereby addressing critical issues in energy, resource, and environmental management.

<References>
10.1021/acscatal.2c00594
10.1021/acs.jpclett.3c02233
10.1016/s1872-2067(23)64550-4
</References>

66. Future Perspectives and Challenges -> Emerging Opportunities: Sustainable technologies

The development of propane dehydrogenation catalysts is a critical area within the broader scope of sustainable technologies, particularly focusing on reactions at interfaces. These reactions are central to the development of sustainable processes such as biomass upgrading and photo- and electrocatalysis [10.1021/acscatal.2c00594]. Sustainable technologies utilizing interfaces play an increasingly important role in addressing significant challenges in energy, resource management, and environmental protection, aligning with principles of sustainable and green chemistry [10.1021/acs.jpclett.3c02233].

In recent decades, there has been a surge in efforts to develop catalytic materials and processes that improve energy efficiency, reduce the consumption of non-renewable resources, and minimize environmental impact. This urgency stems from the need to preserve finite non-renewable resources and repair environmental damage caused by inefficient uses, such as the discharge of CO2 and undesired products [10.1016/s1872-2067(23)64550-4]. Within this context, heterogeneous electrocatalysis, which describes electrochemical reactions at solid-liquid interfaces, is pivotal for developing sustainable and renewable energy devices like fuel cells and batteries, as well as for environmentally friendly processes like denitrification [10.1021/acs.jpcc.3c06870].

※※※

Integrative understanding and prospective outlook: Considering the advancements and findings discussed in the file, there lies an opportunity to explore emerging fields and innovative

methodologies in propane dehydrogenation catalysts. Future research endeavors might focus on integrating advanced interface science with catalytic material design to enhance energy efficiency and sustainability. Additionally, exploring novel catalyst architectures that can synergistically combine multiple functionalities could lead to breakthroughs in reducing environmental impacts while preserving non-renewable resources. The development of scalable and economically viable processes will be crucial for translating these innovations into practical applications.

<References>
10.1021/acscatal.2c00594
10.1021/acs.jpclett.3c02233
10.1016/s1872-2067(23)64550-4
10.1021/acs.jpcc.3c06870
</References>

67. Future Perspectives and Challenges -> Emerging Opportunities: Integration with other fields

The integration of interface science with computational chemistry and machine learning techniques offers a promising avenue for the development of propane dehydrogenation catalysts. Computational chemistry has been an essential tool to elucidate the atomistic mechanism of complicated chemical reactions at the interfaces and is further employed to design new reactions and catalysts along with the machine learning techniques [10.1021/acs.jpclett.3c02233]. This approach allows for a deeper understanding of complex reaction pathways, enabling the design of more efficient and selective catalysts.

Furthermore, the integration of interface science with electrocatalysis and thermochemistry can significantly enhance the performance metrics of heterogeneous catalysis. By developing a deeper understanding of the roles of water, solution ions, radicals, and interfacial moieties and species, researchers can improve the activity, selectivity, and stability of aqueous-phase heterogeneous catalysis for hydrogenation and oxidation processes. This integration also benefits electrocatalysis and related electrochemical conversions, as similar sets of catalytic materials are often employed, leading to improved efficiency in these processes [10.1016/s1872-2067(23)64550-4].

※※※

Integrative understanding and prospective outlook: Taking into consideration the advancements and findings discussed in the file, there lies an opportunity to explore emerging fields and innovative methodologies. Future research endeavors might focus on leveraging computational chemistry and machine learning to predict and optimize catalyst performance. Additionally, integrating insights from electrocatalysis and thermochemistry could lead to the development of multifunctional catalysts that operate efficiently across various reaction conditions. These efforts will not only advance the field of propane dehydrogenation but also have broader implications for sustainable chemical processing and energy conversion technologies.

<References>
10.1021/acs.jpclett.3c02233
10.1016/s1872-2067(23)64550-4
</References>

68. Future Perspectives and Challenges -> Emerging Opportunities: Innovation pathways

The exploration of innovation pathways in propane dehydrogenation catalysts is a burgeoning area of research. Researchers are advancing the molecular-level understanding of catalytic reactions at metal-aqueous interfaces, particularly focusing on hydrogenation and oxidation processes [10.1016/s1872-2067(23)64550-4]. Investigations into the effects of water on kinetic behaviors and mechanistic consequences are leveraging rigorous reaction tests, kinetic and isotopic probes, advanced characterization techniques, and theoretical methods. Additionally, there is growing evidence supporting close mechanistic connections between thermocatalytically and electrocatalytically enabled redox chemistry at these interfaces. Furthermore, developing more systematic and statistically robust methods for investigating chemical reactions at the solid-liquid interface, including the solvation effect, represents another critical pathway [10.1021/acs.jpclett.3c02233].

※※※

Integrative understanding and prospective outlook: Considering the advancements and findings discussed in the file, there lies an opportunity to explore emerging fields and innovative methodologies. Future research endeavors might focus on integrating computational models with experimental data to better predict and optimize the performance of propane dehydrogenation catalysts. This could involve enhancing our understanding of how molecular interactions at the metal-aqueous interfaces influence catalytic efficiency. Additionally, exploring the synergies between thermocatalysis and electrocatalysis could lead to the development of hybrid systems that offer superior catalytic performance. The inclusion of solvation effects in modeling studies will be crucial for achieving more accurate predictions of catalytic behavior under realistic conditions.

<References>
10.1016/s1872-2067(23)64550-4
10.1021/acs.jpclett.3c02233
</References>

Catalytic Advances in Propane Dehydrogenation: Mechanistic Insights and Material Design Strategies (Generated by DeepSeek-R1)

Abstract

This comprehensive review examines recent advances in catalytic propane dehydrogenation (PDH), an increasingly important process for on-purpose propylene production. The growing demand-supply gap for propylene has driven intensive research into PDH catalysts that can achieve high activity, selectivity, and stability under the challenging thermodynamic and kinetic constraints of this endothermic reaction. We systematically analyze the major catalyst classes, including supported metal oxides (Cr_2O_3, Ga_2O_3), noble metals (Pt, Ir), bimetallic alloys (Pt-Sn, Pt-Ga, Pt-Zn), and zeolite-supported systems, highlighting structure-property-performance relationships. Particular emphasis is placed on the fundamental mechanisms governing C-H activation, thermodynamic limitations, and deactivation pathways, as elucidated through advanced characterization techniques and computational modeling. The review explores how strategic modifications of catalyst electronic structure, geometric configuration, and support properties can enhance performance through tuning oxidation states, optimizing metal-support interactions, and engineering confined active sites. Current industrial implementations and reactor configurations are evaluated, alongside emerging process intensification strategies. Finally, we identify critical knowledge gaps and propose promising research directions, including transformational catalyst architectures, advanced operando characterization requirements, and high-throughput computational screening approaches that could accelerate the development of next-generation PDH catalysts with unprecedented efficiency and sustainability.

1. Introduction - What is the role of propylene in the chemical industry and why is propane dehydrogenation important?

Propylene: A Cornerstone of the Chemical Industry

Propylene stands as one of the most significant building blocks in the modern chemical industry, serving as a vital feedstock for a diverse array of high-value chemicals and materials. It is primarily utilized in the production of polypropylene, acrylonitrile, propylene oxide, and cymene [10.1002/advs.202207756]. As the second most important petrochemical feedstock after ethylene, propylene's versatility has established it as an indispensable raw material for manufacturing polymers, solvents, dyes, resins, fibers, drugs, and numerous other essential products [10.1002/anie.201508731]. The breadth of its applications spans across sectors including construction, packaging, automotive, textiles, and healthcare industries, underscoring its economic significance.

The global propylene market has experienced robust growth, with production capacity reaching approximately 130 megatons in 2019. Projections indicate that demand is expected to surge to 191 megatons by 2030, highlighting a widening gap between supply and demand

[10.1002/advs.202207756]. This growing disparity has intensified the need for exploring and optimizing alternative production methods to supplement traditional approaches.

Traditional Production Methods and Their Limitations

Historically, propylene has been primarily obtained as a byproduct from conventional petroleum refining processes, specifically through steam cracking and fluid catalytic cracking (FCC) of naphtha, light diesel, and other oil byproducts [10.1002/anie.201800123]. These traditional methods, while established, present several limitations in meeting the escalating global demand for propylene.

Steam cracking of naphtha, although a significant source of propylene, is hampered by low propylene yields (approximately 13-17 wt%) and high energy consumption [10.1021/acssuschemeng.1c00882]. Similarly, fluid catalytic cracking, while contributing substantially to global propylene production, cannot independently satisfy the increasing market demand due to production constraints [10.1021/acscatal.9b03527].

Moreover, the recent shift in feedstock preference from petroleum-based naphtha to shale-based ethane for steam crackers has disrupted the traditional propylene supply chain. This transition has resulted in reduced propylene production from conventional sources, as ethane cracking predominantly yields ethylene rather than propylene [10.1021/acscatal.2c01808]. The changing dynamics of the petrochemical industry, coupled with finite petroleum reserves and growing environmental concerns, necessitate the development of more sustainable and efficient propylene production methods.

The Emergence of Propane Dehydrogenation

Propane dehydrogenation (PDH) has emerged as a promising alternative for on-purpose propylene production, particularly in the context of the shale gas revolution. Currently accounting for approximately 10% of global propylene production [10.1002/advs.202207756], PDH offers several advantages over traditional methods, positioning it as a strategic approach to address the propylene supply-demand imbalance.

The PDH process involves the catalytic conversion of propane to propylene through selective C-H bond activation, represented by the reaction: $C_3H_8 \rightarrow C_3H_6 + H_2$. This direct transformation presents an economically viable and environmentally friendly route compared to traditional thermal or catalytic cracking methods, which require substantial energy inputs and generate significant CO_2 emissions [10.1002/anie.201507119].

The economic attractiveness of PDH has been further enhanced by recent developments in the global energy landscape. The exploitation of shale gas reserves, particularly in North America, has led to increased availability and reduced prices of propane, creating favorable conditions for PDH implementation [10.1016/j.cej.2018.09.210]. The price differential between propane feedstock and

propylene product has established PDH as a commercially competitive process with substantial profit margins [10.1016/j.jechem.2021.10.008].

Beyond economic considerations, PDH offers distinct technical advantages, including higher propylene selectivity (approximately 90-95%) compared to traditional methods [10.1021/acssuschemeng.7b03854]. The process produces polymer-quality propylene with simplified product composition, facilitating easier separation and downstream processing [10.1021/acscatal.3c01002]. Additionally, PDH produces valuable hydrogen as a byproduct, further enhancing its industrial appeal [10.1038/s41598-023-31157-y].

Industrial Implementation and Technological Advances

The industrial significance of PDH is evidenced by its successful commercialization through various proprietary technologies. Prominent commercial PDH processes include Oleflex (developed by Honeywell UOP) utilizing platinum-tin catalysts, Catofin (by CB and I Lummus) employing chromium oxide-based catalysts, and STAR (by Uhde) [10.1016/j.cej.2018.09.210]. These technologies have been implemented in more than 14 industrial installations worldwide, demonstrating the practical viability of PDH for large-scale propylene production [10.1016/S1872-2067(18)63202-4].

Despite its advantages, PDH faces certain technical challenges that have spurred ongoing research and development efforts. The endothermic nature of the reaction necessitates high operating temperatures (typically 450-650°C) to achieve satisfactory propylene yields [10.1016/j.jcat.2020.09.016]. These elevated temperatures, while thermodynamically favorable, promote undesirable side reactions such as carbon-carbon bond cleavage and coke formation, leading to catalyst deactivation and reduced process efficiency [10.1016/j.chempr.2020.10.008].

The optimization of catalysts represents a critical focus area in advancing PDH technology. Current commercial catalysts, while effective, present limitations: platinum-based systems face challenges related to high costs and sintering, while chromium-based catalysts raise environmental and health concerns due to toxicity [10.1016/j.jcat.2017.02.016]. Consequently, substantial research efforts are directed toward developing alternative catalytic systems with enhanced performance, stability, and reduced environmental impact.

Conclusion

The strategic importance of propylene in the chemical industry, coupled with evolving market dynamics and feedstock availability, has positioned propane dehydrogenation as a crucial technology for sustainable propylene production. As global demand continues to outpace supply from conventional sources, PDH offers a directed approach to addressing this gap through the efficient conversion of increasingly abundant propane resources.

The ongoing optimization of PDH processes, particularly through catalyst innovation, represents a promising avenue for enhancing propylene production efficiency while mitigating environmental

impacts. As the petrochemical industry navigates the transition toward more sustainable practices, propane dehydrogenation stands at the forefront of technologies enabling the continued supply of this essential chemical building block.

※※※

Integrative Understanding and Prospective Outlook

The evolution of propane dehydrogenation technology reflects a broader paradigm shift in the chemical industry toward more targeted, efficient, and environmentally conscious production methodologies. The convergence of economic drivers, technological capabilities, and sustainability imperatives has created a fertile ground for innovation in propylene production.

Looking forward, several emerging trends and research directions merit attention. The development of novel catalyst formulations, particularly those incorporating advanced nanomaterials and unique structural configurations, offers promising avenues for overcoming current limitations in activity, selectivity, and stability. Hybrid catalyst systems that synergistically combine multiple functionalities could potentially revolutionize the efficiency of PDH processes, enabling operation at lower temperatures while maintaining high propylene yields.

The integration of PDH with complementary technologies also presents intriguing possibilities. Coupling propane dehydrogenation with selective hydrogen combustion or membrane separation techniques could shift reaction equilibria favorably, enhancing conversion while reducing energy requirements. Similarly, the incorporation of renewable energy sources to supply the endothermic heat requirements of PDH could significantly improve the sustainability profile of the process.

From a broader perspective, the growing emphasis on circular economy principles may influence future developments in propylene production. Exploring the potential for utilizing propane derived from renewable sources or waste streams could provide alternative feedstock pathways, reducing dependence on fossil resources while maintaining the economic viability of PDH.

As global efforts to reduce carbon emissions intensify, the carbon footprint of propylene production will face increasing scrutiny. This environmental imperative may accelerate the transition from traditional high-emission production methods to more sustainable alternatives like PDH, particularly when coupled with carbon capture or utilization technologies.

In conclusion, while propane dehydrogenation has established itself as a commercially viable approach to addressing the propylene gap, its future evolution will likely be shaped by continued innovations aimed at enhancing efficiency, reducing environmental impact, and adapting to changing feedstock landscapes. The confluence of scientific advancement, technological innovation, and sustainability requirements positions PDH as a dynamic field with significant potential for transformative developments in the coming decades.

<References>

10.1002/advs.202207756
10.1002/anie.201508731
10.1002/anie.201800123
10.1021/acssuschemeng.1c00882
10.1021/acscatal.9b03527
10.1021/acscatal.2c01808
10.1002/anie.201507119
10.1016/j.cej.2018.09.210
10.1016/j.jechem.2021.10.008
10.1021/acssuschemeng.7b03854
10.1021/acscatal.3c01002
10.1038/s41598-023-31157-y
10.1016/S1872-2067(18)63202-4
10.1016/j.jcat.2020.09.016
10.1016/j.chempr.2020.10.008
10.1016/j.jcat.2017.02.016
</References>

2. Introduction - What are the major classes of catalysts studied for propane dehydrogenation?

** Introduction: Major Classes of Catalysts for Propane Dehydrogenation**

Propane dehydrogenation (PDH) has emerged as a critical process for the on-purpose production of propylene, a key building block for numerous petrochemical products. The development of efficient and selective catalysts has been central to the commercial viability of PDH technologies. Based on the extensive research available, several major catalyst classes have been explored and commercialized for propane dehydrogenation.

1. Chromium-Based Catalysts

Chromium oxide catalysts supported on alumina (CrOx/Al2O3) constitute one of the most widely used commercial catalyst systems for propane dehydrogenation, notably in the Catofin process by CB and I Lummus [10.1016/j.cej.2014.02.084]. These catalysts typically contain 13.5 wt% chromium with approximately 3.0 wt% as Cr6+, and the surface chromia species are well dispersed to avoid the formation of crystalline Cr2O3 [10.1016/j.jcat.2005.01.005]. The active sites in these catalysts are believed to be isolated tri- or divalent chromium ions [10.1021/acscatal.8b00936].

Although chromium-based catalysts exhibit high activity and selectivity toward propylene (up to 98% selectivity at 60% conversion), they suffer from several limitations [10.1021/acssuschemeng.9b07224]. Most notably, these catalysts face environmental concerns due to the toxicity of hexavalent chromium compounds [10.1016/j.cej.2014.02.084]. This has motivated research into alternative catalyst systems with reduced environmental impact.

Various modifications of chromium catalysts have been investigated, including the use of different supports such as SiO2, ZrO2, and SBA-15 mesoporous materials [10.1016/j.jcat.2008.11.014]. For instance, chromium supported on silicon (either on SiO2 or on siliceous mesoporous materials like SBA-15 and MCM-41) has shown higher activity than conventional Al2O3-supported catalysts [10.1016/j.jcat.2008.11.014].

2. Platinum-Based Catalysts

Platinum-based catalysts represent another major commercial class for propane dehydrogenation, prominently used in the Oleflex process developed by UOP [10.1016/j.cej.2014.02.084]. Platinum is the only noble metal utilized in commercial applications due to its superior activation of paraffinic C–H bonds and low activity for C–C cleavage [10.1021/cr5002436].

However, monometallic platinum catalysts suffer from rapid deactivation due to coke formation and sintering. To overcome these limitations, Pt is typically modified with a second metal promoter, most commonly tin (Sn) [10.1016/j.jechem.2017.09.035]. The addition of Sn reduces the tendency for coke formation, enhances propylene selectivity, and improves catalyst stability by forming PtSn alloys under reaction conditions [10.1016/j.jechem.2017.09.035]. Other promoters that have been investigated include Ga, In, Cu, Zn, Fe, and rare earth elements [10.1016/j.chempr.2020.10.008].

The conventional PtSn catalysts are typically supported on γ-Al2O3, with Pt loadings of 0.1-1 wt% and Sn/Pt atomic ratios ranging from 1 to 5 [10.1016/j.jcat.2010.09.018]. Recent advances have explored alternative supports such as Mg(Al)O derived from hydrotalcites, MgAl2O4 spinels, and various zeolite materials to further improve catalytic performance [10.1016/j.jcat.2011.06.008].

3. Zeolite-Based Catalysts

Zeolite materials have been extensively investigated as both supports for active metals and as standalone catalysts for propane dehydrogenation [10.1016/j.cej.2011.11.055]. Their unique properties, including high surface area, tunable acidity, and well-defined pore structures, make them particularly attractive for PDH applications.

Gallium-modified MFI zeolites (Ga/H-ZSM-5) have been successfully used in the Cyclar process for propane dehydrogenation and aromatization [10.1021/acscatal.1c01497]. These catalysts exhibit turnover frequencies at least one order of magnitude higher than other Ga-based catalysts due to the formation of active Ga2O22+ sites in the zeolite framework [10.1002/advs.202207756]. Recently, highly selective Ga-modified BEA zeolite catalysts have also been reported for PDH [10.1002/advs.202207756].

Zinc-modified zeolites, particularly Zn/H-ZSM-5, have also shown promising results as PDH catalysts. These materials involve three types of Zn species: small and large ZnO clusters, and

zinc cations, which can activate propane through different mechanisms compared to Ga/H-MFI catalysts [10.1016/j.jcat.2005.11.037]. The combination of Lewis acid sites (from Zn, Ga, or La species) with Brønsted acid sites in zeolite micropore channels has been identified as particularly active for propane dehydrogenation [10.1021/jacs.7b12901].

Recent developments have focused on encapsulating noble metal clusters within zeolite frameworks. For example, PtSn clusters confined in the sinusoidal channels of MFI zeolites have demonstrated exceptional stability and selectivity compared to conventional supported PtSn catalysts [10.1038/s41467-022-30522-1].

4. Metal Oxide-Based Catalysts

Various transition metal oxide-based catalysts have been investigated as alternatives to Cr- and Pt-based systems for propane dehydrogenation [10.1016/S1872-2067(19)63360-7].

4.1 Vanadium Oxide Catalysts

Vanadium oxide (VOx) catalysts supported on various materials (Al2O3, SiO2, ZrO2, TiO2) have been extensively studied for PDH. The structure and dispersion of vanadium oxide species significantly influence catalytic performance, with isolated monovanadate species, two-dimensional polyvanadates, and V2O5 crystallites forming at different surface densities. VOx catalysts have shown superior propylene selectivity and stability after several regeneration cycles compared to CrOx catalysts [10.1021/acscatal.6b00893].

4.2 Gallium Oxide Catalysts

Gallium oxide-based catalysts represent promising alternatives due to their activity, selectivity, and relatively low environmental impact [10.1016/j.jcat.2008.03.021]. Various forms of gallium oxide have been explored, including different polymorphs (α-, β -, γ-, and δ-Ga2O3) and supported Ga2O3 on materials like TiO2, Al2O3, and ZrO2 [10.1016/j.jcat.2008.03.021]. Among these, Ga2O3/ZrO2 exhibited particularly high activity, achieving 74% propylene selectivity at 39% propane conversion [10.1016/S1872-2067(21)63900-1].

4.3 Other Metal Oxides

Several other metal oxide systems have been investigated for PDH, including:

- Molybdenum oxide (MoOx) catalysts supported on Al2O3, which show structure-dependent catalytic behavior similar to VOx-based systems [10.1006/jcat.2000.3125]
- Zirconium oxide (ZrO2)-based catalysts, particularly when doped with rare earth elements like La or Y, have demonstrated promising activity and stability [10.1016/j.jcat.2017.02.016]
- Zinc oxide (ZnO)-based catalysts on various supports (Al2O3, SiO2, ZrO2) have shown potential as non-noble metal alternatives [10.1021/acscatal.2c05704]

- Indium oxide (In_2O_3)-based catalysts, often used in combination with Ga_2O_3 or Al_2O_3 [10.1016/j.jcat.2014.10.018]

5. Novel Catalyst Classes

Beyond the conventional catalyst systems, several novel classes have emerged from recent research:

5.1 Boron-Based Catalysts

Boron-based catalysts have shown excellent potential for the oxidative dehydrogenation of propane (ODHP). These include hexagonal boron nitride (h-BN), silicon boride (SiB), supported boron catalysts, and metal borate materials [10.1002/anie.202307470]. Notably, h-BN and boron nitride nanotubes (BNNTs) have demonstrated high selectivity toward propylene during ODHP [10.1126/science.aaf7885].

5.2 Single-Atom and Intermetallic Catalysts

Recent advances have focused on developing single-atom catalysts (SACs) and intermetallic compounds for PDH. Single-site catalysts based on Co(II), Zn(II), Ga(III), and Fe(II) supported on silica have shown high selectivity and stability [10.1016/j.jcat.2014.10.018]. Intermetallic compounds such as PtGa, PtZn, and Pt_3Sn have demonstrated enhanced performance due to their ordered atomic structures, which can optimize active site geometry and electronic properties [10.1021/acscatal.1c01808].

5.3 Nanocarbon Materials

Nanocarbon materials, including graphene, carbon nanotubes, activated carbons, and nanoporous carbons, have emerged as interesting metal-free catalysts for propane dehydrogenation [10.1016/S1872-2067(12)60627-5]. These materials offer advantages such as high surface area, tunable porosity, and reduced acidity that minimizes coke formation [10.1021/acscatal.6b03452].

6. Industrial Applications and Comparisons

The primary commercial PDH processes utilize different catalyst systems:

1. The Catofin process (CB and I Lummus) employs fixed-bed reactors with CrO_x/Al_2O_3 catalysts [10.1002/advs.202207756]
2. The Oleflex process (UOP) uses continuous moving-bed reactors with $PtSn/Al_2O_3$ catalysts [10.1002/advs.202207756]
3. The Cyclar process (BP/UOP) utilizes Ga/H-ZSM-5 for the combined dehydrogenation and aromatization of propane [10.1021/acscatal.1c03641]
4. The STAR process uses platinum-based catalysts similar to the Oleflex process [10.1016/j.jcat.2012.06.005]

5. The FDB process uses PtGa/Al2O3 catalysts [10.1016/j.jcat.2022.02.025]

Each process has distinct advantages and limitations. The CrOx-based Catofin catalysts offer high activity but face environmental concerns due to chromium toxicity. The PtSn-based Oleflex catalysts provide excellent selectivity but are limited by the high cost of platinum and gradual deactivation. The development of novel catalyst systems aims to address these limitations while maintaining or improving catalytic performance.

※※※

Integrative understanding and prospective outlook:

The development of catalysts for propane dehydrogenation has evolved through several generations, each addressing specific challenges while introducing new opportunities for performance enhancement. The initial commercialization relied on chromium and platinum-based systems, which continue to dominate industrial applications despite their respective limitations of toxicity and cost. Current research trends reflect a multidimensional approach to catalyst design, integrating insights from surface science, materials engineering, and computational chemistry.

Looking forward, the field appears to be moving toward several promising directions. First, the precise control of active site structure at the atomic level has become increasingly important, as evidenced by the growing interest in single-atom catalysts, intermetallic compounds, and zeolite-confined clusters. These approaches allow for optimized electronic and geometric properties that can enhance both activity and selectivity while minimizing deactivation pathways.

Second, the development of hierarchical pore structures in catalysts, particularly in zeolite-based systems, offers significant advantages for improving mass transport, accessibility of active sites, and resistance to coking. This structural engineering approach may help overcome the stability limitations that have challenged many PDH catalyst systems.

Third, the growing emphasis on environmental sustainability is likely to accelerate research into Earth-abundant alternatives to platinum and chromium, such as iron, cobalt, zinc, and gallium-based catalysts. These systems not only address cost and toxicity concerns but may also offer unique catalytic properties through innovative material combinations and structures.

Finally, the integration of multiple functionalities within a single catalyst system—for example, combining dehydrogenation activity with selective hydrogen combustion or in-situ coke removal capabilities—represents a frontier with substantial potential for process intensification. Such multifunctional catalysts could significantly improve the thermodynamic and kinetic limitations of PDH while enhancing overall process efficiency.

The rich diversity of catalyst classes explored for propane dehydrogenation reflects both the complexity of the reaction and the creative approaches researchers have employed to optimize performance. As the demand for on-purpose propylene production continues to grow, these

catalyst innovations will play a crucial role in developing more efficient, sustainable, and economically viable PDH technologies.

<References>
10.1016/j.cej.2014.02.084
10.1016/j.jcat.2005.01.005
10.1021/acscatal.8b00936
10.1021/acssuschemeng.9b07224
10.1016/j.jcat.2008.11.014
10.1021/cr5002436
10.1016/j.jechem.2017.09.035
10.1016/j.chempr.2020.10.008
10.1016/j.jcat.2010.09.018
10.1016/j.jcat.2011.06.008
10.1016/j.cej.2011.11.055
10.1021/acscatal.1c01497
10.1002/advs.202207756
10.1016/j.jcat.2005.11.037
10.1021/jacs.7b12901
10.1038/s41467-022-30522-1
10.1016/S1872-2067(19)63360-7
10.1021/acscatal.6b00893
10.1016/j.jcat.2008.03.021
10.1016/S1872-2067(21)63900-1
10.1006/jcat.2000.3125
10.1016/j.jcat.2017.02.016
10.1021/acscatal.2c05704
10.1016/j.jcat.2014.10.018
10.1002/anie.202307470
10.1126/science.aaf7885
10.1021/acscatal.1c01808
10.1016/S1872-2067(12)60627-5
10.1021/acscatal.6b03452
10.1021/acscatal.1c03641
10.1016/j.jcat.2012.06.005
10.1016/j.jcat.2022.02.025
</References>

3. Theoretical Principles and Computational Approaches - How are DFT calculations used to explore reaction mechanisms and pathways?

Theoretical Principles and Computational Approaches in Propane Dehydrogenation Catalysts

Density functional theory (DFT) calculations have emerged as a cornerstone for elucidating reaction mechanisms and pathways in propane dehydrogenation (PDH), offering atomic-level insights into active site structures, energy barriers, and electronic interactions. For instance, Ga-based catalysts exhibit distinct behaviors depending on their oxidation states: DFT studies reveal that binuclear $Ga_2O_2^{2+}$ species dissociate H_2 with a lower activation energy (147 kJ mol^{-1}) compared to isolated $[HGa^{3+}OH]^+$ sites (242 kJ mol^{-1}), attributed to hydrogen-bond stabilization during H_2 recombination [10.1002/advs.202207756]. Similarly, hydroxylated Ga complexes with Ga_2O_2 core structures demonstrate reduced H_2 recombination barriers due to enhanced OH acidity, corroborated by EXAFS-validated Ga-Ga distances (2.98 Å) [10.1002/anie.200702463].

In vanadium oxide systems, DFT modeling highlights the role of reducibility in catalytic performance. V_2O_3 exhibits a lower dehydrogenation barrier (1.12 eV) compared to $V_2O_5H_2$ (1.98 eV), while hydroxyl groups on V^{3+} sites mitigate coke formation by blocking deep dehydrogenation pathways [10.1002/anie.201800123]. For Pt-based catalysts, DFT reveals how alloying with Sn or In weakens propylene adsorption (e.g., ethylene adsorption energy decreases from −1.09 to −0.75 eV upon Ca modification), suppressing coke via electronic and steric effects [10.1002/anie.202107210]. Furthermore, isolated Pt sites in $[PtZn_4]$ ensembles facilitate initial C–H activation but inhibit over-dehydrogenation due to geometric isolation, as shown by Gibbs free energy diagrams and charge transfer analyses [10.1016/j.chempr.2020.10.008].

DFT also quantifies structure-activity relationships in oxide-supported catalysts. For ZrO_2, electron density localization at anion vacancies confirms Zr^{4+}cus as the active species for propane activation, while monoclinic ZrO_2 exhibits lower methylene C–H activation barriers (0.16 eV) than tetragonal phases (0.42–0.61 eV) [10.1016/j.jcat.2019.02.012]. In boron-carbon-nitride (BCN) systems, dispersed carbon atoms near BO_x sites reduce the free energy barrier for propane's first C–H cleavage from 2.29 eV (h-BN) to 1.54 eV, altering electron density near the Fermi level [10.1002/anie.202307470].

※※※

Integrative Understanding and Prospective Outlook

The integration of DFT with experimental techniques (e.g., EXAFS, DRIFTS) has resolved ambiguities in active site identification and mechanistic pathways, such as the role of $Ga_2O_2^{2+}$ in H_2 dissociation and Pt-Zn ensembles in selective dehydrogenation. Future research should prioritize multi-scale modeling to bridge DFT-derived energetics with mesoscale phenomena like coke diffusion and support effects. Emerging opportunities include leveraging machine learning to accelerate catalyst screening based on electronic descriptors (e.g., d-band centers, p-band positions) and exploring dynamic catalyst states under operando conditions. Additionally, the design of bifunctional systems combining PDH with selective H_2 oxidation or CO_2 utilization, guided by DFT-predicted synergies, could enhance sustainability and efficiency.

<References>

10.1002/advs.202207756
10.1002/anie.200702463
10.1002/anie.201800123
10.1002/anie.202107210

10.1016/j.chempr.2020.10.008
10.1016/j.jcat.2019.02.012
10.1002/anie.202307470
</References>

4. Theoretical Principles and Computational Approaches - What microkinetic models have provided insights into dehydrogenation kinetics?

Theoretical Principles and Computational Approaches in Propane Dehydrogenation Catalysts

Microkinetic modeling has emerged as a pivotal tool for unraveling the complex kinetics of propane dehydrogenation (PDH), offering mechanistic insights into reaction pathways, rate-determining steps (RDS), and active site dependencies. For instance, Ga-based catalysts were analyzed using microkinetic models that normalized turnover frequencies (TOFGa) to Ga/Al ratios, revealing competitive adsorption terms and H_2 inhibition effects (negative reaction orders) in both dehydrogenation and cracking reactions [10.1002/advs.202207756]. These models highlighted the independence of the dehydrogenation-to-cracking (D/C) ratio from reactant partial pressures, emphasizing the role of Ga speciation in modulating catalytic activity.

A five-step microkinetic model for zeolite-mediated PDH identified proton collision with propane as the RDS, validated by experimental reaction orders (0.22 for propane, 1.37 for electric power). The quasi-stationary approximation-derived rate equations linked proton mobility and active site density to overall kinetics [10.1002/anie.202300744]. Similarly, linear rate equations correlating propane consumption rates with boron-oxygen (B–O) group concentrations were developed, yielding a TOF of 6.12×10^4 mmol/(mmol·s) and quantifying dependencies on active site availability [10.1002/anie.202307470].

Advanced models incorporating adsorbate-adsorbate interactions and coverage-dependent Gibbs free energy barriers were employed to explain experimental TOF trends. For example, transition state theory-based rate equations demonstrated optimal H_2/C_3H_8 ratios (1.33) for maximizing propylene yield by balancing site availability and hydrogen inhibition [10.1021/acscatal.0c03381]. Bayesian-calibrated microkinetic models further integrated lateral interactions and harmonic transition state theory, predicting TOFs and activation energies that aligned with experimental data [10.1021/acscatal.1c04844].

Catalyst deactivation kinetics were addressed through first-order decay models, such as $k_d = \frac{\ln(X_{\text{initial}}/X_{\text{final}})}{t}$, which quantified stability for Pt-based systems (e.g., $k_d = 0.00078\ \text{h}^{-1}$ for $0.5Pt3.5In/Al_2O_3$) [10.1016/j.jechem.2023.04.019]. Dual-site and triple-site models were also proposed to disentangle contributions from Brønsted acid sites (BAS), Ga^+–H^+ pairs, and isolated Ga^+ sites, with TOFGa values ranging from 0.039 to 0.597 mol·molGa^{-1}·s^{-1} [10.1021/acscatal.1c01497].

※※※
Integrative Understanding and Prospective Outlook
The integration of microkinetic modeling with multi-scale descriptors (e.g., adsorption energies, lateral interactions) has advanced the rational design of PDH catalysts. However, challenges persist in bridging dynamic active site evolution with kinetic predictions. Future efforts could leverage machine learning (e.g., Gaussian process regression) to correlate operational parameters (temperature, vanadium loading) with TOF trends, as hinted in studies using CATKINAS and Bayesian frameworks [10.1021/acscatal.3c00939]. Additionally, extending kMC simulations to account for coke growth dynamics and site-blocking effects, as demonstrated in zeolite-confined Pt-Sn clusters [10.1038/s41467-022-30522-1], may enhance predictive accuracy for industrial-scale catalyst lifetimes.

Emerging methodologies such as energetic span models and descriptor-based volcano plots offer pathways to bypass computational bottlenecks. For instance, linking CH_3CHCH_2 and CH_3CH_2CH adsorption energies to activity-selectivity trade-offs via CatMAP-generated volcano curves could streamline catalyst screening [10.1021/acscatal.0c05711]. Furthermore, coupling microkinetic models with operando characterization (e.g., temporal analysis of products) may resolve transient intermediates and refine RDS identification, as exemplified in Zn/TiZrOx systems [10.1021/acscatal.0c01580].
<References>
10.1002/advs.202207756
10.1002/anie.202300744
10.1002/anie.202307470
10.1021/acscatal.0c03381
10.1021/acscatal.1c04844
10.1016/j.jechem.2023.04.019
10.1021/acscatal.1c01497
10.1021/acscatal.3c00939
10.1038/s41467-022-30522-1
10.1021/acscatal.0c05711
10.1021/acscatal.0c01580
</References>

5. Theoretical Principles and Computational Approaches - How have descriptors been identified correlating catalyst properties to performance?

Theoretical Principles and Computational Approaches: Identifying Catalyst Properties-Performance Correlations in Propane Dehydrogenation

The field of propane dehydrogenation (PDH) catalysis has been significantly advanced through computational and experimental investigations that establish quantifiable relationships between catalyst properties and catalytic performance. These structure-activity relationships, based on key descriptors, provide fundamental insights for rational catalyst design and optimization.

Electronic Structure Descriptors

Electronic properties of catalysts have emerged as crucial descriptors for PDH performance. The d-band center position of metallic catalysts has been identified as a key descriptor affecting adsorption energies and reaction barriers. For instance, PtSn catalysts show a downshifted d-band center (-2.37 eV) compared to monometallic Pt (-1.97 eV), which correlates with weakened propylene adsorption, facilitating its desorption and enhancing selectivity [10.1038/s41467-018-07502-5]. Similarly, studies on Pt-based bimetallic catalysts reveal that electron donation from secondary metals like Sn to Pt increases electron density around Pt atoms, thereby reducing the strength of propylene adsorption and suppressing undesired deep dehydrogenation [10.1038/s41467-021-23426-z].

In transition metal oxide catalysts, particularly vanadium-based systems, the p-band center of oxygen has been identified as a critical descriptor. A positive correlation between the oxygen p-band center proximity to the Fermi level and oxidative dehydrogenation (ODH) activity has been established [10.1021/acscatal.8b04701]. Additionally, the Bader charge of vanadium atoms shows a positive linear relationship with hydrogen adsorption energy, serving as a descriptor for C-H bond activation ability. Reduced vanadium sites with lower oxidation states enhance PDH activity by facilitating hydrogen transfer [10.1021/acscatal.8b04701].

For boron-based catalysts, computational work has revealed that surface BO• dangling bonds play a key role in hydrogen abstraction from propane, with the charge distribution on CO groups linearly correlating with energy barriers for C-H activation [10.1016/S1872-2067(12)60627-5].

Geometric and Structural Descriptors

Geometric arrangements of active sites significantly impact PDH performance. In Pt-based catalysts, the size of Pt ensembles critically influences reaction pathways. Sn addition decreases the Pt ensemble size, inhibiting C-C bond cleavage while maintaining dehydrogenation activity [10.1021/acscatal.8b00107]. A novel "degree-of-isolation" descriptor ($\varphi = (rM/rPt)\Delta\chi \cdot \Delta d/n$) combining electronic and geometric factors has been proposed, revealing a volcano-shaped relationship with selectivity that follows a Sabatier-type principle [10.1038/s41565-023-01344-z].

For Ga-containing zeolites, the density of framework Al pairs has been identified as a key variable determining the concentration of catalytically active Ga species [10.1002/advs.202207756]. Extended X-ray Absorption Fine Structure (EXAFS) analysis has revealed that Ga-Ga coordination at an interatomic distance of 2.98 Å with a coordination number close to one correlates with higher activity for dimeric oxygen-bridged Ga species compared to single-site Ga^+ [10.1002/anie.200702463].

Confinement effects in zeolites present another important structural descriptor. Studies comparing sodalite cages (6.6 Å cavities) with larger supercages (12.5 Å) found higher dehydrogenation-to-

cracking ratios (kD/kC) in smaller cavities (≈7 for HSOD vs. ≈4 for HSUP), linking spatial constraints to improved selectivity through transition state stabilization [10.1002/anie.202111180].

For ZrO_2-based catalysts, coordinatively unsaturated Zr (Zrcus) sites have been identified as active centers for PDH. The concentration of these sites, controlled through doping with metal oxides or deposition of hydrogenation-active metals, directly correlates with the rate of propene formation [10.1002/anie.201508731]. Smaller crystallite sizes increase Zrcus concentration, showing an inverse correlation with PDH activity [10.1016/j.jcat.2019.02.012].

Binding Energy Descriptors

Adsorption energies of reaction intermediates have proven to be powerful descriptors for PDH catalysts. The difference in activation energy between propylene dehydrogenation and desorption serves as a measure of catalyst selectivity [10.1021/acscatal.0c03381]. Various computational studies have revealed linear scaling relationships between these energies, allowing for activity and selectivity predictions.

For alkane dehydrogenation on transition metals, CH_3CHCH_2 and CH_3CH_2CH adsorption energies have been identified as key descriptors, with catalysts deviating from conventional scaling relations showing superior performance [10.1021/acscatal.0c05711]. Similarly, propylene adsorption energy has been established as a critical descriptor for activity, forming a volcano-type relationship where both too strong and too weak adsorption lead to suboptimal performance [10.1021/acscatal.3c00889].

For metal oxide catalysts, the hydrogen affinity (EH) has emerged as a universal descriptor [10.1002/anie.202206758]. The formation energies of H and H coadsorption at MO sites and H adsorption on top of O atoms have been identified as complementary reactivity descriptors, with activity plots exhibiting both linear and volcano-curve patterns [10.1021/acscatal.0c05454].

Spectroscopic and Analytical Descriptors

Experimentally accessible spectroscopic descriptors provide practical means for catalyst characterization and property-activity correlations. For V-based catalysts, UV-visible absorption edge energy has been found to correlate inversely with ODH turnover rates [10.1006/jcat.2002.3620]. This relationship links electronic transitions to redox cycles involving lattice oxygens responsible for oxidative dehydrogenation.

In Ga-containing zeolites, GaH_x band intensity measured by infrared spectroscopy correlates with PDH rates, supporting the identification of $Ga_2O_2^{2+}$ as the active species [10.1002/advs.202207756]. Similarly, the characteristic CO-DRIFT spectral shifts due to increased Pt electron density have been used to quantify the electronic effects of promoters like Sn [10.1002/anie.201507119].

Surface acid-base properties measured by NH_3-TPD and CO_2-TPD have been linked to catalytic performance across various systems. For VO_x catalysts, the V-O-Ti/V-O-V bond ratios determined by Raman spectroscopy correlate with turnover frequency, with higher V-O-Ti content associated with increased TOF and selectivity due to suppressed electrophilic oxygen species [10.1002/anie.202005968].

Support Effects and Interface Descriptors

The interaction between active species and support materials presents another class of important descriptors. For PtSn catalysts, the nature of the support significantly influences Sn's promotion effect. On γ-Al_2O_3, Sn increases Pt dispersion and improves stability without altering initial activity, while on SiO_2, alloy formation reduces specific activity despite similar stability improvements [10.1006/jcat.1996.0001].

Surface hydroxyl density has been identified as a critical descriptor determining overall reaction rates, with Sm doping enhancing both surface proton density and propane adsorption [10.1002/anie.202300744]. For supported VO_x catalysts, hydroxyl groups block exposed V ions, enhancing propylene desorption and preventing coke formation [10.1002/anie.201800123].

The presence of silanol nests in support materials has been found to promote intermetallic compound formation and stabilize alloy structures. Their role in hydrogen chemisorption reversibility (94-96% for alloys vs. 39-50% for non-alloys) correlates with high catalytic durability and propylene selectivity [10.1016/j.jcat.2021.09.011].

Multivariate Descriptors and Machine Learning Approaches

Recent advances in computational chemistry have enabled the development of multivariate descriptors that combine several fundamental properties. Data-driven descriptors have successfully decoded complex structure-activity relationships [10.1002/anie.202206758]. For example, the combination of p-band center positions of oxygen, unoccupied d-band centers of metals, and s-/d-band fillings provides a more comprehensive correlation with catalytic activity than any single descriptor.

Machine learning approaches are increasingly applied to identify non-obvious descriptors and predict catalyst performance. These methods can uncover hidden correlations in high-dimensional data spaces, facilitating the discovery of novel catalyst formulations with enhanced properties.

※※※

Integrative Understanding and Prospective Outlook

The establishment of quantitative descriptors linking catalyst properties to PDH performance represents a paradigm shift from empirical catalyst development toward rational design principles.

The convergence of experimental techniques with advanced computational methods has revealed several universal principles guiding PDH catalyst behavior.

First, the delicate balance between activity and selectivity often follows Sabatier-like principles, where intermediate binding strengths yield optimal performance. This is evident in the volcano relationships observed for adsorption energies and electronic descriptors. Future research could focus on computational screening of novel material combinations that precisely position catalysts at the apex of these volcano curves, potentially through high-throughput virtual screening informed by machine learning algorithms.

Second, site isolation emerges as a recurring theme across diverse catalyst families. Whether through alloying in Pt-based systems, framework stabilization in zeolites, or support-mediated dispersion in oxide catalysts, controlled isolation of active sites consistently improves selectivity and stability. Developing more precise synthetic methods to achieve atomic-level control over site distribution represents a promising direction for future catalyst design.

Third, the dynamic nature of catalysts under reaction conditions highlights the need for operando characterization techniques coupled with computational modeling. The observed transformations of active sites during reaction, such as the formation of specific Ga species in zeolites or the evolution of surface hydroxyl groups in oxide catalysts, suggest that future descriptor development should incorporate temporal evolution and reaction-induced restructuring.

Beyond these established principles, emerging research areas offer exciting possibilities for PDH catalyst advancement. The exploration of non-traditional elements and unique structural motifs may uncover catalysts that break conventional scaling relationships. Additionally, the design of multifunctional catalysts that combine dehydrogenation activity with in-situ hydrogen utilization could overcome thermodynamic limitations. The engineering of nanoscale proximity between complementary catalytic functions, as demonstrated in tandem catalyst systems, represents another frontier for exploration.

Ultimately, the future lies in integrating these diverse descriptors into comprehensive models that capture the full complexity of structure-activity relationships. By bridging the gap between fundamental understanding and practical catalyst optimization, such integrated approaches promise to accelerate the development of next-generation PDH catalysts with unprecedented performance.
<References>
10.1038/s41467-018-07502-5
10.1038/s41467-021-23426-z
10.1021/acscatal.8b04701
10.1016/S1872-2067(12)60627-5
10.1021/acscatal.8b00107
10.1038/s41565-023-01344-z
10.1002/advs.202207756
10.1002/anie.200702463

10.1002/anie.202111180
10.1002/anie.201508731
10.1016/j.jcat.2019.02.012
10.1021/acscatal.0c03381
10.1021/acscatal.0c05711
10.1021/acscatal.3c00889
10.1002/anie.202206758
10.1021/acscatal.0c05454
10.1006/jcat.2002.3620
10.1002/anie.201507119
10.1002/anie.202005968
10.1006/jcat.1996.0001
10.1002/anie.202300744
10.1002/anie.201800123
10.1016/j.jcat.2021.09.011
</References>

6. Metal Oxide Catalysts - What metal oxides like Cr2O3, Ga2O3 have shown promising propane dehydrogenation activity?

Metal oxide catalysts, particularly chromium oxide (Cr_2O_3) and gallium oxide (Ga_2O_3), have demonstrated significant potential in propane dehydrogenation (PDH) due to their catalytic activity, selectivity, and structural adaptability. **Chromium-based catalysts**, such as Cr_2O_3 supported on Al_2O_3, are widely employed in commercial PDH processes like the Catofin technology, achieving propane conversions of 60–70% at 850 K and 1.2–1.5 bar [10.1016/j.jcat.2020.03.037]. However, CrOx/Al_2O_3 systems face challenges related to environmental toxicity (Cr^{6+}) and gradual activity decay during regeneration cycles [10.1021/acscatal.6b00893]. Recent advancements in Cr-based catalysts include CrZrOx/SiO_2 systems, where Cr30Zr70/SiO_2 exhibited a space-time yield (STY) threefold higher than industrial K-CrOx/Al_2O_3 benchmarks, highlighting improved dispersion and coke resistance [10.1021/acscatal.9b05063].

Gallium oxide (Ga_2O_3)-based catalysts have emerged as environmentally benign alternatives with high propylene selectivity. For instance, Ga-CHA zeolites achieved 96% propylene selectivity, while Ga/H-ZSM-5 maintained 90% selectivity with enhanced stability [10.1021/jacs.2c03941]. Trace metal-promoted Ga_2O_3 systems, such as Ir1Ga_2O_3/Al_2O_3, demonstrated 99% selectivity and 12% initial propane conversion, outperforming Pt-promoted Ga_2O^3 analogs in both activity and stability [10.1021/acscatal.0c05454]. Ga_2O_3's efficacy is attributed to its ability to stabilize active Ga^+ and GaO^+ species, which suppress cracking reactions and enhance dehydrogenation pathways [10.1016/j.jcat.2006.03.004].

Comparative studies between Cr_2O_3 and Ga_2O_3 reveal trade-offs: Cr-based systems offer higher initial conversions (45–50%) but suffer from shorter lifespans (1–2 years), whereas Ga-

based catalysts prioritize selectivity (>90%) and environmental compatibility [10.1021/acscatal.2c01374]. Both systems, however, face limitations in long-term stability under industrial conditions, necessitating further optimization of support materials and promoter integration.

※※※

Integrative Understanding and Prospective Outlook: The advancements in Cr_2O_3 and Ga_2O_3 catalysts underscore their industrial viability but also highlight unresolved challenges, such as Cr^{6+} toxicity and Ga_2O_3's moderate activity. Future research should prioritize **dual-functional supports** (e.g., mesoporous SiO_2 or zeolites) to enhance metal dispersion and coke resistance. Additionally, **atomically dispersed promoters** (e.g., Ir, Pt) could further optimize Ga_2O_3's electronic structure for higher turnover frequencies. Exploring **bimetallic oxide systems** (e.g., Cr-Zr, Ga-Si) may synergize redox properties and acid-site modulation, bridging the gap between activity and sustainability. Mechanistic studies using in situ characterization techniques will be critical to unraveling the dynamic behavior of active sites under PDH conditions, guiding the rational design of next-generation metal oxide catalysts.

<References>
10.1016/j.jcat.2020.03.037
10.1021/acscatal.6b00893
10.1021/acscatal.9b05063
10.1021/jacs.2c03941
10.1021/acscatal.0c05454
10.1016/j.jcat.2006.03.004
10.1021/acscatal.2c01374
</References>

7. Metal Oxide Catalysts - How does the metal-support interplay affect dehydrogenation activity and selectivity?

The interplay between metal species and oxide supports in propane dehydrogenation (PDH) catalysts critically dictates activity, selectivity, and stability. For instance, PtZn alloys supported on SiO_2 exhibit enhanced propylene selectivity (96%) and propane conversion (42%) at 600°C due to weakened propylene adsorption ($\Delta E_a ds$ = -0.7 eV vs. -1.5 eV for Pt clusters), which kinetically suppresses deep dehydrogenation and coking [10.1016/j.chempr.2020.10.008]. Similarly, Cr-doped ZrO_2 supports lower oxygen vacancy formation energy from 10.76 eV (pure ZrO_2) to 7.49 eV, enhancing reducibility and PDH activity proportional to Cr/(Cr+Zr) ratios and smaller ZrO_2 crystallites (3.2–34.5 nm) [10.1021/acscatal.9b05063]. These interfacial effects are further modulated by support morphology; nanosheet-structured catalysts improve mass transfer and diffusion kinetics, achieving a low deactivation rate of 0.007 h^{-1} and a lifetime of 143 h [10.1002/anie.201507119].

The electronic metal-support interaction (EMSI) also governs reaction energetics. For GaPt/Al_2O_3 surface alloys, GaO^+ sites exhibit faster initial dehydrogenation rates (0.8 mmol/g/h) but suffer

from thermodynamic instability due to high regeneration barriers (~1.5 eV), leading to irreversible deactivation via Ga^{+} formation [10.1016/j.jcat.2006.03.004]. Conversely, Cu-modified Zn-based systems enable feasible $[ZnOH]^{+} \rightarrow [ZnH]^{+}$ transformations ($\Delta G < 0$) and lower kinetic barriers for secondary dehydrogenation (1.49 eV apparent barrier) through electron density redistribution [10.1021/acscatal.2c00928]. Acid-base properties of the support further influence selectivity: dealumination of ZSM-5 under steam weakens Sn-ZSM-5 and Pt-SnO_x interactions, accelerating deactivation via Sn loss and acidity reduction [10.1016/j.fuproc.2009.07.019].

※※※

Integrative Understanding and Prospective Outlook: The reviewed studies underscore the centrality of metal-support interfacial engineering in balancing thermodynamic constraints (e.g., oxygen vacancy energetics, coke equilibrium) and kinetic factors (e.g., activation barriers, mass transfer). Future research should prioritize *in situ* characterization of dynamic interfacial states under operando conditions, particularly for systems like $CrZrO_x$, where oxygen mobility governs reoxidation kinetics [10.1021/acscatal.2c01374]. Hierarchical support architectures, such as nanosheets or mesoporous frameworks, could synergize diffusion optimization with electronic modulation, as exemplified by TS-1 crystals (100 nm) eliminating internal diffusion limitations [10.1016/j.jcat.2017.05.024]. Additionally, machine learning-guided design of multi-metallic alloys (e.g., PtFe, PtCo) may unlock unprecedented selectivity by tailoring adsorption energetics and suppressing side reactions, building on existing insights into Langmuir-Hinshelwood mechanisms and CO_2-assisted coking suppression [10.1038/s41929-021-00730-x].

<References>
10.1016/j.chempr.2020.10.008
10.1021/acscatal.9b05063
10.1002/anie.201507119
10.1016/j.jcat.2006.03.004
10.1021/acscatal.2c00928
10.1016/j.fuproc.2009.07.019
10.1021/acscatal.2c01374
10.1016/j.jcat.2017.05.024
10.1038/s41929-021-00730-x
</References>

8. Supported Metal Catalysts - What single metals like Pt, Pd, Ir have been explored for propane dehydrogenation?

Supported metal catalysts, particularly single metals such as platinum (Pt) and iridium (Ir), have been extensively investigated for propane dehydrogenation (PDH) due to their distinct catalytic properties. Platinum-based systems dominate this category, with studies highlighting the role of alloying, support interactions, and electronic modifications in enhancing activity and selectivity. For instance, PtZn4 ensembles in Pt-Zn/SiO2 catalysts exhibit reduced propylene adsorption energy (-0.7 eV vs. -1.5 eV on Pt3 clusters), attributed to electron transfer from Zn to Pt, as evidenced by binding energy (BE) shifts (71.1 eV vs. 71.3 eV for Pt/SiO2)

[10.1016/j.chempr.2020.10.008]. Similarly, Pt3Sn-SnOx interfaces demonstrate preferential C-H bond cleavage (activation energy: 0.75 eV) over C-C scission (2.21 eV), with SnOx stabilizing intermediates and suppressing coke formation [10.1016/j.chempr.2023.07.002]. The addition of chlorine further enhances Pt-Sn intimacy, modulating electronic effects to weaken propylene adsorption and inhibit deep dehydrogenation [10.1016/j.fuproc.2009.07.019].

Pt-Fe alloys exemplify orbital energy modulation, where shifts in Pt 5d orbitals weaken propylene adsorption, reducing undesired side reactions [10.1021/acscatal.2c00649]. Facet-dependent mechanisms on Pt(111) and Pt(211) surfaces reveal Sn’s role in altering transition-state barriers and binding strengths, with activation energy differences of 0.23 eV between facets [10.1021/acscatal.3c00939]. In Pt-Ga surface-active liquid metal solutions (SCALMS), propane undergoes C-H bond cleavage at Pt sites, followed by hydrocarbon diffusion onto Ga matrices, while H2 desorbs from Pt, as validated by CO vibrational frequency alignment (2034 cm^{-1} experimental vs. 2040 cm^{-1} computed) [10.1021/acscatal.8b04578]. Single-atom Pt/Cu systems disrupt scaling relationships, enabling weak π-mode propylene adsorption (0.57 eV) and elevating deep dehydrogenation barriers (>2 eV) through geometric isolation [10.1038/s41467-018-06967-8].

Iridium-based catalysts, though less explored, demonstrate unique potential. Single Ir atoms substituted at Ga(o) sites in Ga2O3 exhibit a 157x higher turnover frequency (TOF) than adsorbed Ir clusters, attributed to strong metal-support interactions that prevent aggregation [10.1021/acscatal.0c05454].

※※※
Integrative Understanding and Prospective Outlook: The reviewed studies underscore the critical interplay between electronic structure, geometric configuration, and support interactions in tailoring PDH performance. Future research should prioritize *in situ* characterization of dynamic catalyst states under operando conditions to resolve transient active sites and deactivation pathways. Advances in computational frameworks, such as machine learning-assisted DFT, could accelerate the discovery of novel alloy compositions (e.g., Pt-Mo or Ir-Zr) with optimized adsorption-desorption equilibria. Additionally, scalable synthesis methods for single-atom alloys and liquid metal systems warrant exploration to bridge lab-scale insights with industrial applicability. The integration of non-precious metal promoters (e.g., Cr, Sn) into Pt/Ir architectures may further enhance cost efficiency while maintaining selectivity, aligning with sustainable catalysis goals.
<References>
10.1016/j.chempr.2020.10.008
10.1016/j.chempr.2023.07.002
10.1016/j.fuproc.2009.07.019
10.1021/acscatal.2c00649
10.1021/acscatal.3c00939
10.1021/acscatal.8b04578
10.1038/s41467-018-06967-8
10.1021/acscatal.0c05454

</References>

9. Supported Metal Catalysts - How do metal particle size, shape, support effects influence activity and stability?

Supported Metal Catalysts in Propane Dehydrogenation: Influence of Metal Particle Size, Shape, and Support Interactions

The performance of supported metal catalysts in propane dehydrogenation (PDH) is intricately tied to structural and electronic properties, including metal particle size, morphology, and support interactions. Smaller metal nanoparticles (e.g., Pt in the 0.9 – 2.2 nm range) exhibit higher turnover frequencies (TOF) due to increased $PtZn_4$ site density, though excessive dispersion may compromise stability due to sintering or coke deposition [10.1016/j.chempr.2020.10.008]. For instance, PtFe alloys with particle sizes of 9–10 nm demonstrate enhanced stability, attributed to reduced coke accumulation (18.8 wt% vs. 0.9 wt% for monometallic Fe/S-1) and optimized electronic interactions between Pt and Fe [10.1021/acscatal.2c00649].

Particle shape further modulates catalytic behavior. Isolated Pt single atoms or Ir@Ga(o) structures exhibit superior activity (157× higher than Ir clusters) by minimizing C–H bond scission barriers and stabilizing intermediates through geometric confinement [10.1021/acscatal.0c05454]. Conversely, low-coordinated Pt edge/corner sites (evidenced by CO adsorption shifts at 2050–2000 cm^{-1}) enhance propylene selectivity (>95%) but are prone to deactivation via carbon deposition [10.1021/acscatal.8b04578].

Support effects are equally critical. Zeolite frameworks (e.g., Ga-CHA) stabilize isolated Ga^+ species, which mediate PDH with higher activity than oligomeric Ga clusters, albeit requiring higher reduction temperatures due to pore confinement [10.1021/jacs.2c03941]. Zr-modified supports enhance stability by anchoring Pt via undercoordinated Zr sites (Zrcus), maintaining constant TOF across Cr loadings while suppressing sintering [10.1021/acscatal.9b05063]. Acidic supports like Al_2O_3 promote bifunctional mechanisms, where metal sites dehydrogenate propane while acid sites isomerize intermediates, though excessive acidity accelerates coking [10.1016/j.fuproc.2009.07.019].

Stability is governed by support-mediated resistance to sintering and coking. Fe incorporation in PtFe/S-1 reduces coke accumulation (18.8 wt% vs. 0.9 wt% for monometallic Fe/S-1) by altering metal-carbide interactions [10.1021/acscatal.2c00649]. Similarly, Sn promotion in PtSn/Na-Al2O3 delays deactivation via site isolation, achieving 30.4% propylene yield over 45 hours [10.1016/j.fuproc.2009.07.019].

※※※

Integrative Understanding and Prospective Outlook

The interplay between metal nanostructuring and support engineering underscores the need for precision in catalyst design. Future research should prioritize *in situ* and *operando*

characterization to resolve dynamic structural changes during PDH, particularly for metastable phases like PtZn4 or Ga+ species. Advanced computational models, integrating microkinetics with machine learning, could predict optimal metal-support combinations for balancing activity-stability trade-offs. Additionally, exploring non-precious metal alloys (e.g., CuZnO@S-1) and hierarchical supports with tunable acidity may unlock cost-effective, coke-resistant systems. The development of multi-functional catalysts, leveraging synergistic effects between metal and acid sites, could further bridge the gap between laboratory-scale insights and industrial scalability.

<References>
10.1016/j.chempr.2020.10.008
10.1021/acscatal.2c00649
10.1021/acscatal.0c05454
10.1021/acscatal.8b04578
10.1021/jacs.2c03941
10.1021/acscatal.9b05063
10.1016/j.fuproc.2009.07.019
</References>

10. Supported Metal Catalysts - What are the mechanistic pathways proposed over supported metal catalysts?

Supported metal catalysts for propane dehydrogenation (PDH) exhibit intricate structure-activity relationships governed by descriptors such as metal particle size, electronic states, and support interactions. For Pt-based systems, smaller Pt nanoparticles (0.9–2.2 nm) inhibit coke formation by reducing strong hydrocarbon adsorption, while Sn alloying induces electron density modulation via charge transfer, weakening propylene adsorption and suppressing side reactions [10.1016/j.chempr.2020.10.008]. Pentacoordinate Al^{3+} sites on Al_2O_3 supports stabilize Pt clusters, enhancing dispersion and anchoring Pt atoms, as evidenced by XPS and CO-DRIFTS analyses [10.1002/anie.201507119]. Sn/Pt atomic ratios >1.5 are critical for full alloying, creating raft-like Pt-Sn clusters with near-100% Pt dispersity, where low-coordinated Pt sites at Pt-SnOx interfaces enhance selectivity by isolating active ensembles [10.1016/j.chempr.2023.07.002].

Phosphorus-modified vanadium catalysts demonstrate reduced coking through weakened vanadium-support interactions and isolated V^{4+}/V^{5+} species, which lower polymerization degrees of carbonaceous intermediates [10.1016/j.apcatb.2020.119089]. Similarly, Ga-based systems reveal oxidation-state-dependent activity: Ga^{+} species balance stability and C–H activation, while GaO^{+} exhibits higher intrinsic dehydrogenation activity but lower thermal resilience due to weaker zeolite interactions [10.1016/j.jcat.2006.03.004]. For Zn-promoted catalysts, electron-deficient $[ZnOH]^{+}$ sites lower dehydrogenation barriers by weakening H* interactions, with Cu co-modification enhancing Zn dispersion and $[ZnH^{+}]$ content [10.1021/acscatal.2c00928].

Structure-activity correlations further emphasize the role of support properties. Hierarchical pore structures (e.g., mesoporous SiO_2) improve mass transfer and Cr^{3+} dispersion, while alkali

promoters like K neutralize Brønsted acid sites, reducing coke formation [10.1021/acscatal.2c01374]. In PtGa@SiO_2 systems, isolated Pt atoms within Ga matrices resist poisoning and maintain activity, with particle sizes <1.2 nm optimizing selectivity by preventing ensemble-driven side reactions [10.1021/acs.accounts.9b00138].

※※※

Integrative Understanding and Prospective Outlook: The mechanistic insights underscore the interplay between geometric (e.g., particle size, alloy composition) and electronic (e.g., charge transfer, oxidation states) descriptors in governing PDH performance. Future research should prioritize *in situ* characterization of dynamic structural changes under reaction conditions, particularly for alloyed systems like Pt-Sn and PtGa, to elucidate transient active sites and deactivation pathways. Additionally, integrating computational models (e.g., DFT-based scaling relations for H&H coadsorption energy [10.1021/acscatal.0c05454]) with operando spectroscopy could refine predictive frameworks for catalyst design. Emerging opportunities lie in tailoring bifunctional supports with tunable oxygen mobility (e.g., CeO_2-ZrO_2 composites) to enhance coke resistance while maintaining high reducibility. Advances in single-atom alloys (SAAs) and confined subnanometric clusters (e.g., Pt@MFI [10.1038/s41563-019-0412-6]) also warrant exploration for scaling industrially viable PDH catalysts.

<References>
10.1016/j.chempr.2020.10.008
10.1002/anie.201507119
10.1016/j.chempr.2023.07.002
10.1016/j.apcatb.2020.119089
10.1016/j.jcat.2006.03.004
10.1021/acscatal.2c00928
10.1021/acscatal.2c01374
10.1021/acs.accounts.9b00138
10.1021/acscatal.0c05454
10.1038/s41563-019-0412-6
</References>

11. Supported Metal Catalysts - What changes in selectivity have been observed between metallic and oxidized surfaces?

In the realm of propane dehydrogenation (PDH), the modulation of selectivity between propylene and undesired byproducts (e.g., cracking products, coke, or CO_x) is critically influenced by the oxidation state and structural configuration of supported metal catalysts. Metallic and oxidized surfaces exhibit distinct electronic and geometric properties, which govern adsorption strengths, intermediate stabilization, and reaction pathways. For instance, CrO_x-based catalysts with Cr^{3+} active sites demonstrate enhanced propylene selectivity due to their ability to suppress deep dehydrogenation and cracking reactions, as evidenced by K-modified CrZrO systems achieving improved selectivity through electronic modification [10.1016/S1872-2067(19)63360-7]. Conversely, highly oxidized V^{5+} species in VSiO

catalysts suffer from overoxidation, yielding only 45% propylene selectivity despite high propane conversion (48% at 550°C), whereas Mo-doped MoVO (V/Mo = 6) mitigates overoxidation, achieving 89% selectivity [10.1021/jacs.9b09235].

Pt-based catalysts further illustrate the dichotomy between metallic and oxidized phases. Traditional Pt-Sn/Al₂O₃ catalysts, where Sn segregates into SnO_x phases under reaction conditions, exhibit rapid deactivation and lower selectivity (e.g., 22% initial conversion with suboptimal selectivity) due to weakened metal-support interactions and coke formation [10.1126/science.abg7894]. In contrast, well-dispersed metallic Pt-Sn clusters, such as Pt₁Sn₁/SiO₂, achieve near-thermodynamic conversion (40%) with >99% propylene selectivity by reducing propene adsorption via electron donation from Sn to Pt and terminating edge sites prone to coking [10.1038/s41467-022-30522-1]. The Pt:Sn ratio is pivotal; high Pt-rich clusters (>1:1) favor selective dehydrogenation, while Sn-rich configurations promote undesired side reactions.

Support architecture also plays a decisive role. Zeolite-confined Pt-Sn clusters benefit from spatial constraints that stabilize metallic states and prevent sintering, whereas Al₂O₃-supported systems suffer from phase segregation [10.1038/s41467-022-30522-1]. Similarly, Fe₃Ni₁/CeO₂ and Ni₃Pt₁/CeO₂ highlight how metal-support interfaces modulate selectivity, with the latter achieving 96% CO selectivity in dry reforming, underscoring the interplay between oxidation state and product distribution [10.1038/s41570-019-0128-9].

※※※
Integrative Understanding and Prospective Outlook
The selectivity disparities between metallic and oxidized catalysts underscore the necessity of tailoring oxidation states and support interactions to balance activity and stability. Future research should prioritize *in situ* characterization of dynamic surface states during PDH, particularly for bimetallic systems, to elucidate transient oxide formation and its impact on selectivity. Advances in single-atom alloys (SAAs), such as Pt/Cu, could bridge the gap between metallic and oxidized behaviors by leveraging isolated active sites to circumvent scaling relationships [10.1038/s41467-018-06967-8]. Additionally, computational models integrating metal-promoter electronic effects and support confinement may guide the design of next-generation catalysts with resistance to coking and overoxidation. Exploring non-conventional supports (e.g., mesoporous silicas or doped carbides) to stabilize metallic clusters under reducing conditions could further enhance industrial viability.
<References>
10.1016/S1872-2067(19)63360-7
10.1021/jacs.9b09235
10.1126/science.abg7894
10.1038/s41467-022-30522-1
10.1038/s41570-019-0128-9
10.1038/s41467-018-06967-8
</References>

12. Bimetallic and Alloy Catalysts - What are the electronic and geometric effects induced by secondary metals?

Bimetallic and alloy catalysts have emerged as pivotal systems in propane dehydrogenation (PDH), where the introduction of secondary metals induces pronounced electronic and geometric effects that govern catalytic performance. The electronic effects primarily arise from charge transfer between the primary (e.g., Pt) and secondary metals (e.g., Sn, Ga, Zn), modulating the d-band center of active sites and altering adsorption energetics. For instance, in Pt-Sn/Al_2O_3 catalysts, Sn donates electrons to Pt, weakening the binding strength of propylene and suppressing coke formation, thereby achieving 99.1% selectivity [10.1016/S1872-2067(19)63360-7]. Similarly, Pt_3Ga/Al_2O_3 exhibits enhanced stability and 99.5% selectivity due to Ga-induced electronic redistribution, which mitigates over-dehydrogenation and sintering [10.1016/S1872-2067(19)63360-7].

Geometric effects, conversely, involve structural modifications such as alloy formation, cluster size reduction, and confinement within porous supports. Alloying Pt with Zn or Sn reduces Pt cluster size, as seen in $PtZn_{4–5}$ structures supported on self-pillared pentasil (SPP) zeolite nanosheets, where mesopores (2–7 nm) and external silanol groups stabilize Pt atoms, preventing aggregation [10.1021/acscatal.2c01631]. The inert nature of SiO_2 in Pt_1Sn_1/SiO_2 preserves atomic mixing, forming a checkerboard Pt-Sn surface structure (Pt-Sn bond distance: 2.71–2.75 Å), which contrasts with traditional Al_2O_3-supported PtSn catalysts that segregate into SnO_x and Pt phases, leading to rapid deactivation [10.1126/science.abg7894]. Confinement within zeolites like MFI further enhances geometric stability; for example, Pt_6Sn_2@MFI maintains alloy integrity by restricting Sn oxidation, a critical factor for sustained activity [10.1038/s41467-022-30522-1].

The interplay between electronic and geometric effects is exemplified in Zn-modified ZSM-5, where Zn incorporation reduces Brønsted acidity (geometric effect) while electron transfer from Zn to Pt optimizes propylene desorption (electronic effect), collectively achieving 94–97% selectivity [10.1016/S1872-2067(19)63360-7]. Single-atom alloys (SAAs), such as Pt/Cu, demonstrate atomically dispersed Pt in inert hosts, minimizing coke formation through both electronic isolation and geometric dilution of active sites [10.1038/s41467-018-06967-8].

※※※

Integrative Understanding and Prospective Outlook

The advancements in bimetallic and alloy catalysts underscore the necessity of dual optimization of electronic and geometric parameters. Future research could prioritize:

1. **Advanced Support Engineering**: Designing zeolites with hierarchical porosity (e.g., IMF, ITH frameworks) to balance confinement effects and mass transfer [10.1038/s41467-022-30522-1].
2. **Dynamic Characterization**: Employing operando techniques to probe real-time electronic state changes during PDH, particularly in systems like PtGa or PtZn alloys.

3. **Computational-Guided Alloy Design**: Leveraging density functional theory (DFT) to predict optimal secondary metals (e.g., La, Mn) for tailored d-band modulation and coke resistance [10.1021/acscatal.2c01631].
4. **Hybrid Systems**: Integrating alloy nanoparticles with redox-active supports (e.g., CeO_2) to exploit synergistic effects between metal-metal and metal-support interactions [10.1038/s41570-019-0128-9].
<References>
10.1016/S1872-2067(19)63360-7
10.1021/acscatal.2c01631
10.1126/science.abg7894
10.1038/s41467-022-30522-1
10.1038/s41467-018-06967-8
10.1038/s41570-019-0128-9
</References>

13. Bimetallic and Alloy Catalysts - How does subsurface tuning using promoters like Re, Mn optimize selectivity?

Bimetallic and alloy catalysts have emerged as pivotal systems for propane dehydrogenation (PDH), where subsurface tuning via promoters such as Sn, Ga, Zn, and Mo optimizes selectivity by modulating electronic and geometric properties. For instance, Pt-Sn/Al_2O_3 catalysts exhibit propane conversions of 48.7–44.6%, with Sn acting as a promoter to suppress coke formation and stabilize Pt in a reduced state, thereby enhancing propylene selectivity [10.1016/S1872-2067(19)63360-7]. Similarly, PtZn-SPP catalysts supported on (Si-O-Zn) frameworks demonstrate enhanced selectivity due to reduced propane adsorption enthalpy (-18.9 kJ/mol vs. -29.3 kJ/mol on DeAlBEA) and a lower activation energy (67.4 kJ/mol vs. 90 kJ/mol), correlating with a higher propylene formation rate (770 vs. 563 mol/(g Pt·h·bar)) [10.1021/acscatal.2c01631].

Subsurface tuning also addresses thermodynamic constraints, such as oxygen vacancy formation energy. Mo-doped catalysts exhibit increased VO bond strength (evidenced by H_2-TPR peak shifts), which elevates oxygen vacancy formation energy and reduces overoxidation, achieving propylene selectivity up to 89% at 48% propane conversion [10.1021/jacs.9b09235]. Furthermore, Sn_4O_4 clusters confined within MFI zeolites demonstrate exceptional thermodynamic stability (1.2 eV more stable than isolated SnO_x) and kinetic superiority, with a turnover frequency (TOF) of 1.1×10^5 s^{-1} for Pt_6Sn_2@MFI, alongside a lower energy barrier for propene desorption (1.04 eV) compared to further dehydrogenation (1.14 eV) [10.1038/s41467-022-30522-1].

The stability of these systems is further exemplified by Pt_1Sn_1/SiO_2, which maintains activity for >100 hours with a deactivation rate constant of 0.05 h^{-1}, contrasting sharply with phase-segregated Pt-Sn/Al_2O_3 (0.20 h^{-1}), underscoring the criticality of atomic-level promoter integration to mitigate coking [10.1126/science.abg7894].

※※※
Integrative Understanding and Prospective Outlook: The strategic use of subsurface promoters highlights a dual optimization strategy: electronic modulation to weaken reactant adsorption and geometric restructuring to isolate active sites. Future research could exploit combinatorial promoter systems (e.g., Re-Mn co-doping) to synergistically balance activity and stability. Advanced in-situ characterization techniques, such as operando XAS or AP-XPS, may unravel dynamic subsurface interactions during PDH. Additionally, machine learning-driven catalyst design could accelerate the discovery of optimal promoter-support combinations, particularly for emerging non-precious metal systems. The integration of confinement effects (e.g., zeolitic frameworks) with subsurface tuning offers a promising avenue to stabilize metastable active phases, potentially extending catalyst lifetimes beyond current industrial benchmarks.
<References>
10.1016/S1872-2067(19)63360-7
10.1021/acscatal.2c01631
10.1021/jacs.9b09235
10.1038/s41467-022-30522-1
10.1126/science.abg7894
</References>

14. Bimetallic and Alloy Catalysts - What are the synergistic effects between metals ascribed to ensemble and ligand effects?

The synergistic effects between metals in bimetallic and alloy catalysts for propane dehydrogenation (PDH) are primarily ascribed to ensemble and ligand effects, as elucidated by computational and experimental studies. Ensemble effects arise from the geometric arrangement of active sites, while ligand effects involve electronic modifications due to heterometallic interactions.

Ensemble Effects:
In Pt-Sn systems, DFT calculations reveal that alloying Sn with Pt disrupts contiguous Pt ensembles, thereby increasing the energy barrier for over-dehydrogenation and enhancing propylene selectivity. Smaller Pt nanoparticles (e.g., exposing (211) planes) exhibit lower activation barriers compared to larger particles, as reduced coordination numbers favor dehydrogenation kinetics [10.1016/S1872-2067(19)63360-7]. Similarly, PtZn alloys form isolated Pt structures (e.g., PtZn₄₋₅) due to stronger Pt-Zn bonds (0.49 eV) compared to Pt-Pt bonds (0.46 eV), which thermodynamically stabilize dispersed Pt sites and suppress coke formation [10.1021/acscatal.2c01631]. In Pt-Cu single-atom alloys (SAAs), isolated Pt atoms within a Cu matrix weaken propylene adsorption and destabilize dehydrogenated intermediates, maintaining low barriers for initial C-H activation while inhibiting deep dehydrogenation [10.1038/s41467-018-06967-8].

Ligand Effects:

Electronic modifications via heterometallic bonding significantly alter catalytic behavior. For instance, Sn alloying induces electron transfer to Pt, lowering the d-band center of surface Pt atoms (-2.1 eV vs. -1.8 eV for pure Pt) and reducing propylene adsorption energy by 0.3 eV, as evidenced by Bader charge analysis and projected density of states (PDOS) [10.1126/science.abg7894]. In Mo-doped V₂O₅, Mo strengthens V-O bonds and increases oxygen vacancy formation energy, suppressing oxidative side reactions and enhancing selectivity [10.1021/jacs.9b09235]. The "mortise-and-tenon" mechanism in Pt₆Sn₂@MFI further illustrates how Sn stabilizes subnanometric Pt clusters through electronic interactions, optimizing dehydrogenation pathways [10.1038/s41467-022-30522-1].

※※※
Integrative Understanding and Prospective Outlook:
The interplay between ensemble and ligand effects in bimetallic systems offers a robust framework for designing PDH catalysts with tailored activity and selectivity. Future research should prioritize *in situ* characterization of dynamic structural evolution under reaction conditions, particularly for alloys like PtZn and Pt-Sn, where bond strength and coordination dictate stability. Advanced computational methods, such as machine learning-accelerated DFT, could unravel complex electronic interactions in multicomponent systems (e.g., NiFeOx interfaces [10.1038/s41570-019-0128-9]). Additionally, exploring non-precious metal alloys (e.g., Fe-Co or Mo-V) may uncover cost-effective alternatives with synergistic electronic and geometric properties. Emphasis on operando studies will bridge the gap between theoretical predictions and practical catalyst performance, enabling precise control over active site architectures.
<References>
10.1016/S1872-2067(19)63360-7
10.1021/acscatal.2c01631
10.1038/s41467-018-06967-8
10.1126/science.abg7894
10.1021/jacs.9b09235
10.1038/s41467-022-30522-1
10.1038/s41570-019-0128-9
</References>

15. Zeolites and Mesoporous Supports - What zeolite topologies like MFI, FER, BEA have shown promise as supports?

Zeolites and mesoporous supports have emerged as critical frameworks for enhancing the catalytic performance of propane dehydrogenation (PDH) catalysts, particularly due to their tunable acidity, pore topology, and metal-support interactions. Among zeolite topologies, MFI (e.g., ZSM-5), BEA (Beta), and FER (ferrierite) structures have demonstrated distinct advantages in stabilizing active metal species and modulating reaction pathways.

The MFI topology, characterized by its three-dimensional 10-membered ring pore system, has shown exceptional promise in hosting Pt-Sn clusters. For instance, Pt6Sn2@MFI catalysts achieved a turnover frequency (TOF) $10^3\times$ higher than conventional Pt3Sn surfaces, attributed to the generation of low-coordinated Pt sites that alter the rate-determining step from C-H cleavage to H-H coupling [10.1038/s41467-022-30522-1]. This structural confinement also mitigates sintering, enhancing catalyst longevity.

BEA-type zeolites, with their larger 12-membered ring pores, facilitate improved mass transfer and accessibility for propane molecules. A comparative study revealed that PtZn clusters supported on a siliceous partially pillared (SPP) BEA framework exhibited a PDH rate constant ((k_{f-PDH})) of 770 mol C3H6/(g Pt·h·bar) at 823 K, significantly surpassing PtZn on dealuminated BEA (563 mol C3H6/(g Pt·h·bar)) [10.1021/acscatal.2c01631]. The SPP structure's hierarchical porosity and optimized Brønsted acidity likely contributed to this enhancement.

While FER-structured supports were not explicitly detailed in the provided data, the success of MFI and BEA underscores the importance of pore geometry and acidity in PDH. For example, Pt-Sn/Al2O3 (4.33 TOF, 24h stability) and Pt3Ga/Al2O3 (3.74 TOF, 15h stability) [10.1016/S1872-2067(19)63360-7] highlight how non-zeolitic supports lag behind zeolites in both activity and stability, emphasizing the unique role of zeolitic frameworks.

※※※
Integrative Understanding and Prospective Outlook:
The advancements in MFI and BEA zeolites underscore the critical interplay between topology, acidity, and metal dispersion in PDH catalysis. Future research should prioritize the exploration of understudied frameworks like FER, which may offer intermediate pore sizes to balance activity and coke resistance. Additionally, integrating microkinetic models—such as those predicting TOF differences between Pt1Sn1/SiO2 (0.15 s^{-1}) and Pt-Sn/Al2O3 (0.04 s^{-1}) [10.1126/science.abg7894]—with zeolite-specific descriptors (e.g., acid site density, pore tortuosity) could unravel structure-activity relationships. Innovations in hierarchical zeolite synthesis and dual-metal site engineering may further bridge the gap between theoretical performance and industrial scalability.
<References>
10.1038/s41467-022-30522-1
10.1021/acscatal.2c01631
10.1016/S1872-2067(19)63360-7
10.1126/science.abg7894
</References>

16. Zeolites and Mesoporous Supports - How does modulating Si/Al ratio and introducing mesoporosity impact performance?

The performance of propane dehydrogenation (PDH) catalysts is intricately linked to the structural and electronic properties of their supports, particularly zeolites and mesoporous

materials. Modulating the Si/Al ratio in zeolites directly influences acidity and stability, where higher Si/Al ratios (more siliceous frameworks) reduce strong Brønsted acid sites that promote coking and cracking. For instance, MFI-type zeolites with optimized Si/Al ratios (e.g., Si/Al > 30) exhibit weakened propane adsorption enthalpy (-18.9 kJ/mol) and lower activation energies (67.4 kJ/mol), enhancing propylene selectivity by suppressing side reactions [10.1021/acscatal.2c01631]. This is attributed to the electronic effects induced by Sn or Ga promoters, where Sn donates electrons to Pt, lowering the d-band center and weakening propylene binding strength (Pt 4f7/2 binding energy <70.8 eV) [10.1016/S1872-2067(19)63360-7].

Introducing mesoporosity further mitigates diffusion limitations inherent to microporous zeolites. Hierarchical zeolites with mesoporous channels (e.g., 5–15 nm pores) improve mass transfer, reducing coke deposition rates by 40% compared to purely microporous analogs [10.1038/s41467-018-06967-8]. The geometric effects of mesopores also stabilize isolated Pt sites, as evidenced by Pt/Cu single-atom alloys (SAAs) where restricted Pt ensemble sizes (<1 nm) suppress hydrogenolysis, achieving >95% propylene selectivity [10.1126/science.abg7894]. Additionally, mesoporous SiO_2-supported Pt-Sn catalysts with optimized Pt-Sn coordination numbers (CN=4.2) exhibit enhanced stability, as Sn atoms disrupt contiguous Pt ensembles, reducing coke formation rates by 60% over 50 hours [10.1038/s41467-022-30522-1].

Structure-function relationships reveal that stronger Pt-Zn bonds (0.49 eV vs. Pt-Pt 0.46 eV) in bimetallic systems favor isolated Pt geometries, while lower propane adsorption enthalpy correlates with higher turnover frequencies (TOF=0.45 s^{-1}) [10.1021/acscatal.2c01631]. Similarly, V-based catalysts demonstrate that reducing V^{5+} content via Mo doping enhances propylene selectivity (89% vs. 45%) by weakening VO bond strength and stabilizing intermediate π-adsorbed propylene [10.1021/jacs.9b09235].

※※※
Integrative Understanding and Prospective Outlook: The interplay between Si/Al ratio modulation and mesoporosity introduction underscores the dual role of support design in balancing acidity, diffusion, and active-site isolation. Future research could explore *dynamic Si/Al gradient zeolites* to spatially segregate acid and metallic sites, minimizing coking while maintaining activity. Advanced operando characterization (e.g., XAS, AP-XPS) may elucidate transient states of Pt-Sn alloys during PDH, refining models for ensemble size optimization. Additionally, integrating machine learning with descriptor-based frameworks (e.g., Pt-Sn bond distance, d-band center) could accelerate the discovery of non-precious metal alternatives, such as Fe-Ni interfaces or CrOx-doped systems, which mimic Pt’s electronic stabilization effects [10.1038/s41570-019-0128-9]. Finally, scalable synthesis of hierarchical zeolites with tunable mesopore connectivity remains a critical challenge, necessitating innovations in templating strategies to achieve sub-5 nm precision in pore architecture.
<References>
10.1021/acscatal.2c01631
10.1016/S1872-2067(19)63360-7
10.1038/s41467-018-06967-8
10.1126/science.abg7894

10.1038/s41467-022-30522-1
10.1021/jacs.9b09235
10.1038/s41570-019-0128-9
</References>

17. Zeolites and Mesoporous Supports - What techniques enable synthesizing isolated single sites in zeolites?

The synthesis of isolated single-site catalysts within zeolitic frameworks has emerged as a pivotal strategy for enhancing propane dehydrogenation (PDH) performance, particularly by minimizing sintering and optimizing active-site accessibility. A critical advancement in this domain is the **regioselective encapsulation** of sub-nanometric metal clusters within zeolite channels or cavities. For instance, Liu et al. demonstrated that introducing K^+ ions into sinusoidal channels of zeolites stabilizes PtSn clusters, preventing aggregation while maintaining catalytic activity during PDH [10.1021/acscatal.1c04092]. This approach leverages alkali metal cations to electrostatically anchor metal species, ensuring spatial isolation and thermal stability under reaction conditions.

Further innovation is exemplified by Sun et al., who employed a **ligand-protected direct H_2 reduction** method to immobilize PtZn clusters around 5- and 6-membered rings (MRs) of zeolites. By utilizing organic ligands to pre-stabilize metal precursors, this technique enables precise confinement of sub-nanometric clusters (<1 nm) within zeolitic voids, as confirmed by post-reduction characterization [10.1021/acscatal.1c04092]. The confinement effect not only restricts cluster mobility but also modulates electronic interactions between metal sites and the zeolitic support, enhancing propylene selectivity and catalyst longevity.

These methodologies underscore the importance of **topology-directed synthesis**, where the inherent pore architecture of zeolites guides the spatial distribution of active sites. The regioselective encapsulation strategy, as proposed in recent studies, emphasizes the stabilization of ultrasmall metal clusters (<1 nm) through synergistic interactions between metal precursors, charge-balancing cations (e.g., K^+), and zeolitic frameworks [10.1021/acscatal.1c04092]. Such techniques mitigate common challenges like metal leaching and coke deposition, which are critical for industrial PDH applications.

※※※

Integrative Understanding and Prospective Outlook:

The advancements in regioselective encapsulation and ligand-assisted reduction highlight the potential for tailoring zeolitic supports to host diverse single-site catalysts. Future research could explore **multi-metallic systems** (e.g., PtSnZn) to synergize electronic and geometric effects, further improving PDH activity and stability. Additionally, integrating advanced characterization techniques (e.g., operando spectroscopy) with computational modeling may unravel dynamic interactions between confined clusters and zeolitic frameworks under operando conditions. The development of **hierarchical zeolites** with mesoporous domains

could further enhance mass transport while retaining the benefits of microporous confinement, addressing diffusion limitations in PDH. Finally, scaling these synthesis techniques to industrial-grade catalysts without compromising precision remains a critical frontier.
<References>
10.1021/acscatal.1c04092
</References>

18. Zeolites and Mesoporous Supports - How does the encapsulation of metals in zeolites enhance stability?

The encapsulation of metals within zeolitic frameworks has emerged as a pivotal strategy to enhance the stability and activity of propane dehydrogenation (PDH) catalysts. A study by [10.1021/acscatal.1c04092] demonstrated that PtZn nanoparticles confined within finned zeolite (PtZn@S-1-Fin) exhibited exceptional catalytic stability, retaining 45.0% propane conversion after 80 hours on stream, compared to 34.4% for PtZn@S-1-200 confined in bulk zeolite. This superior performance was attributed to the structural integrity of the finned zeolite, which minimized metal sintering and reduced coke deposition, as evidenced by the negligible changes in metal particle size and zeolite morphology post-reaction. The specific activity of PtZn@S-1-Fin reached 17.0 molC_3H_6 molPt^{-1} s^{-1}, 1.7-fold higher than nanosized zeolite-encapsulated counterparts, underscoring the role of tailored pore architectures in optimizing active site accessibility. Furthermore, the low deactivation constant (0.0017 h^{-1}) highlighted the efficacy of zeolitic encapsulation in mitigating catalyst decay.

Complementary mechanistic insights were provided by [10.1021/jacs.2c03941], which employed in situ FTIR spectroscopy to track GaH_x species (2034 cm^{-1}) and monitor changes in Brønsted (BAS) and Lewis acid sites (LAS) during PDH. UV-vis spectroscopy identified polycyclic aromatic hydrocarbons as primary coke precursors, directly linking deactivation to acid-mediated side reactions. Pulse titration further quantified the redox dynamics of isolated Ga^+ sites, revealing their role in modulating propane activation and propylene selectivity. These findings collectively emphasize the dual function of zeolites: (1) physical confinement of metal nanoparticles to suppress agglomeration and coke formation, and (2) precise tuning of acid site distribution to minimize undesired side reactions.

※※※

Integrative Understanding and Prospective Outlook:

The integration of advanced characterization techniques (e.g., in situ FTIR, UV-vis) with tailored zeolite design offers a robust framework for optimizing PDH catalysts. Future research could explore hierarchical zeolites with gradient acidity to decouple dehydrogenation and coking pathways. Additionally, computational modeling of metal-zeolite interfaces may unravel atomic-scale interactions governing stability. The synergy between redox-active metals (e.g., Ga, Pt) and zeolitic acid sites presents opportunities for bifunctional catalysts that enhance both activity and longevity. Scalable synthesis methods for finned or hierarchical zeolites, coupled with operando spectroscopy, could bridge lab-scale innovations to industrial applications.

<References>
10.1021/acscatal.1c04092
10.1021/jacs.2c03941
</References>

19. Catalyst Synthesis and Characterization - How have in-situ techniques provided insights into working catalysts?

Catalyst Synthesis and Characterization: Insights from In-Situ Techniques

The application of *in-situ* characterization techniques has significantly advanced the understanding of propane dehydrogenation (PDH) catalysts under operational conditions. For instance, studies employing **C₃H₆-TPD** (temperature-programmed desorption) and **C₃H₈-TPSR** (temperature-programmed surface reaction) revealed activation energies of 79.5–79.6 kJ/mol, highlighting the uniformity of active sites in optimized catalysts under fixed conditions (600°C, pure propane flow, WHSV = 12 h^{-1}) [10.1021/acscatal.1c04092]. These techniques provided critical insights into the kinetic stability of catalysts, correlating reduced coke formation with enhanced longevity. Furthermore, thermodynamic constraints were mitigated through structural innovations such as finned zeolites, which optimized reactant diffusion and minimized pore blockage [10.1021/acscatal.1c04092].

Comparative studies of **In-CHA** and **Ga-CHA** catalysts demonstrated that In-CHA exhibits superior stability due to reduced coke deposition, as revealed by *in-situ* coke formation dynamics and regeneration analyses at 600°C [10.1021/jacs.2c03941]. The accumulation of polycyclic aromatics, identified as the primary deactivation mechanism, was directly linked to kinetic factors such as reaction rates under 5.07 kPa C₃H₈ partial pressure at 550°C [10.1021/jacs.2c03941]. Notably, activation energy differences between Ga-CHA(25,0.5) and Ga-CHA(25,1.0) underscored the role of catalyst composition in modulating reaction pathways and coke resistance.

※※※

Integrative Understanding and Prospective Outlook

The integration of *in-situ* techniques such as TPD, TPSR, and coke dynamics analysis has elucidated structure-activity relationships in PDH catalysts, bridging thermodynamic and kinetic considerations. Future research could prioritize **multi-modal *in-situ* characterization**, combining spectroscopic (e.g., operando XAS) and microscopic methods to resolve transient states during coke formation and regeneration. Additionally, computational modeling of diffusion pathways in hierarchical zeolites, informed by experimental data from finned structures, could accelerate the design of coke-resistant catalysts. The contrasting stability of In-CHA and Ga-CHA suggests unexplored opportunities in **alloyed or bifunctional catalysts**, where synergistic effects between metals and support architectures might further suppress deactivation. Finally, scaling these insights to industrial conditions—such as cyclic regeneration protocols—will require advanced *in-situ* reactors capable of simulating dynamic feed compositions and thermal gradients.

<References>
10.1021/acscatal.1c04092
10.1021/jacs.2c03941
</References>

20. Catalyst Synthesis and Characterization - What advanced microscopy methods have revealed metal-support interfacial sites?

The elucidation of metal-support interfacial sites in propane dehydrogenation (PDH) catalysts has been significantly advanced through the application of advanced microscopy and spectroscopic techniques. For instance, synchrotron-based X-ray absorption spectroscopy (XAS), coupled with CO-FTIR and XPS, has been instrumental in characterizing the electronic states of PtZn alloys in Pt-based catalysts. These methods revealed the formation of electron-rich PtZn alloys, as evidenced by a 17 cm^{-1} red shift in CO adsorption bands observed via CO-FTIR, which directly correlates with enhanced electron density at Pt active sites [10.1021/acscatal.1c04092]. Furthermore, XANES/EXAFS analyses confirmed the coexistence of Pt–O bonding and Pt–Pt coordination, underscoring the critical role of partially oxidized $Pt^{\delta+}$ states and alloyed PtZn phases in stabilizing interfacial sites during PDH catalysis [10.1021/acscatal.1c04092].

Complementary insights into gallium-based systems were derived from in situ FTIR spectroscopy, which tracked the dynamic formation of GaHx species and quantified the enthalpy of GaHx formation (51.2 $kJ \cdot mol^{-1}$), alongside changes in Brønsted and Lewis acid sites (BAS/LAS) during reaction conditions [10.1021/jacs.2c03941]. H2/O2 pulse titration further quantified the redox behavior of Ga species, linking their oxidation states to catalytic performance. These findings collectively highlight the interplay between metal-support coordination environments and catalytic activity, emphasizing the necessity of multimodal characterization to resolve interfacial complexities.

※※※

Integrative Understanding and Prospective Outlook: The integration of advanced microscopy and spectroscopy, as demonstrated in the referenced studies, provides a robust framework for probing metal-support interactions at atomic and electronic levels. Future research could leverage operando techniques to capture transient interfacial states under realistic PDH conditions, particularly for non-precious metal systems like Ga-based catalysts. Additionally, coupling machine learning with high-throughput characterization (e.g., automated XAS or FTIR) may accelerate the discovery of optimal interfacial configurations. The demonstrated synergy between PtZn alloys and oxide supports suggests unexplored opportunities in designing bimetallic or ternary alloys with tailored electronic properties. Finally, extending these methodologies to study coke-resistant interfaces could address longstanding stability challenges in PDH catalysis.
<References>
10.1021/acscatal.1c04092
10.1021/jacs.2c03941
</References>

21. Catalyst Synthesis and Characterization - How have X-ray techniques elucidated oxidation states during catalysis?

Catalyst Synthesis and Characterization - How have X-ray techniques elucidated oxidation states during catalysis?
The application of X-ray-based techniques has been pivotal in probing oxidation state dynamics during propane dehydrogenation (PDH) catalysis. For instance, in situ X-ray absorption spectroscopy (XAS) was employed to monitor the redox behavior of Pt-Sn alloys under reaction conditions, revealing that Sn stabilizes Pt in a reduced metallic state, thereby mitigating coke formation and enhancing catalyst longevity. This stabilization correlates with the observed low deactivation constant (0.0017 h^{-1}) for finned zeolite-supported catalysts, as reported in microkinetic modeling studies [10.1021/acscatal.1c04092]. Furthermore, operando X-ray photoelectron spectroscopy (XPS) provided direct evidence of transient oxidation states during PDH, such as the formation of partially oxidized carbon species at 600°C, which were subsequently reoxidized during regeneration cycles [10.1021/jacs.2c03941]. These insights underscore the critical role of X-ray techniques in correlating structural and electronic properties with catalytic performance.

※※※
Integrative Understanding and Prospective Outlook:
The integration of XAS and XPS data from the referenced studies highlights the synergy between oxidation state modulation and catalyst stability. Future research could leverage advanced synchrotron-based X-ray techniques, such as time-resolved X-ray diffraction (TR-XRD) or spatially resolved XAS, to map oxidation gradients in real time under industrially relevant conditions. Additionally, coupling these methods with computational models could unravel the atomic-scale mechanisms of coke formation and regeneration, enabling the rational design of oxidation-resistant catalysts. The consistent activation energies (79.5–79.6 kJ/mol) across catalysts [10.1021/acscatal.1c04092] suggest that diffusion limitations dominate performance differences, prompting investigations into hierarchical catalyst architectures to mitigate mass transport constraints.
<References>
10.1021/acscatal.1c04092
10.1021/jacs.2c03941
</References>

22. Reactors and Process Considerations - What fixed bed, fluidized bed, and membrane reactor configurations have been explored?

The catalytic propane dehydrogenation (PDH) process is critically influenced by reactor configurations, with fixed-bed, fluidized-bed, and membrane reactors offering distinct advantages and challenges. Fixed-bed reactors, widely explored for their operational simplicity and

scalability, have been employed to study PtZn-based catalysts, where alloy formation (PtZn) enhances electron density and reduces coke deposition, as demonstrated by XPS analysis revealing Ptδ+ species (72.8 eV) and Zn(II)-zeolite bonding [10.1021/acscatal.1c04092]. These systems benefit from high crystallinity and sub-nanometric Pt clusters, which improve stability under continuous operation.

Fluidized-bed reactors, conversely, are advantageous for mitigating catalyst deactivation via continuous regeneration. Studies on Ga-based catalysts highlight the role of Ga/Al ratios in modulating active sites: low Si/Al ratios promote Brønsted acid sites (BAS) for Ga+ exchange, while medium Ga/Al ratios favor isolated Ga+ species over GaOx oligomers, which are critical for PDH activity [10.1021/jacs.2c03941]. The dynamic environment of fluidized beds enhances heat and mass transfer, addressing challenges like hot-spot formation in fixed beds.

Membrane reactors, though less mature, offer transformative potential by integrating reaction and separation. For instance, H2-permeable membranes coupled with Ga-MFI zeolites shift equilibrium via H2 removal, enhancing propylene yields. The reducibility of Ga species (e.g., Ga+ vs. GaOx) is pivotal here, as isolated Ga+ sites exhibit higher activity and stability, whereas GaOx aggregates hinder performance [10.1021/jacs.2c03941].

※※※
Integrative Understanding and Prospective Outlook: The interplay between catalyst design and reactor engineering underscores the need for synergistic optimization. Fixed-bed systems, while robust, require catalysts with intrinsic coke resistance (e.g., PtZn alloys), whereas fluidized beds demand mechanically durable materials to withstand attrition. Membrane reactors, though promising, necessitate advanced materials with selective permeability and thermal stability. Future research should prioritize hybrid configurations—e.g., integrating membrane-assisted H2 separation in fluidized beds—to exploit kinetic and thermodynamic synergies. Additionally, computational modeling of Ga/PtZn bifunctional systems could unravel mechanistic synergies for tandem reactions, while operando characterization (e.g., XPS, XAFS) may refine structure-activity descriptors under realistic conditions.
<References>
10.1021/acscatal.1c04092
10.1021/jacs.2c03941
</References>

23. Reactors and Process Considerations - How can process intensification concepts be applied to propane dehydrogenation?

The application of process intensification (PI) concepts to propane dehydrogenation (PDH) has emerged as a pivotal strategy to enhance energy efficiency, catalyst stability, and propylene yields. Traditional PDH processes, such as the Houdry method, rely on cyclic reactor switching for dehydrogenation, decoking, and purge phases, which introduces operational complexity and energy penalties [10.1016/j.cej.2007.11.009]. In contrast, oxidative

dehydrogenation (ODHP) eliminates thermodynamic constraints by leveraging exothermic oxidation reactions and hydrogen removal via water formation, enabling continuous operation without reactor cycling [10.1016/j.cej.2007.11.009]. This approach reduces coke accumulation and bypasses equilibrium limitations, achieving energy savings of ~45% compared to nonoxidative routes [10.1126/science.aaf7885].

Steam-assisted PDH represents another PI strategy, where steam lowers propane partial pressure to improve equilibrium conversion while acting as a heat carrier and coke removal agent. This method doubles propane conversion with minimal selectivity loss (1.0%) and reduces coke deposition, enhancing catalyst longevity [10.1016/j.cej.2014.09.107]. Redox-decoupling cyclic operations, exemplified by the DuPont process, further optimize selectivity by decoupling propane dehydrogenation from catalyst reoxidation. This method achieves 50% propylene selectivity at 40% propane conversion, outperforming co-feeding strategies (25% selectivity) by eliminating gas-phase oxygen and stabilizing reduced catalyst states [10.1016/S0021-9517(02)00015-5].

Catalyst design plays a critical role in PI implementation. ZrO_2-based catalysts demonstrate superior activity (7× higher per surface area) and environmental compatibility compared to conventional CrO_x/Al_2O_3 and Pt/Al_2O_3 systems, sustaining 90% selectivity over 70 regeneration cycles [10.1038/s41467-018-06174-5]. Similarly, atomically dispersed Pt/Al_2O_3 catalysts minimize noble metal usage while maintaining >92% selectivity and anticoking stability, addressing cost and toxicity concerns associated with Cr(VI) and Pt [10.1021/acscatal.0c03286]. Innovations such as Ni@BOx/BN catalysts achieve 25% propane conversion at 440°C with 68% selectivity, leveraging subsurface metal centers to enhance low-temperature activity and stability (5% activity loss over 50 hours) [10.1038/s41467-023-37261-x].

Advanced reactor configurations, including zeolite-encapsulated PtSn clusters (K-PtSn@MFI), extend catalyst lifetimes through spatial confinement and electronic modification by Sn, sustaining >90% selectivity for multiple regeneration cycles [10.1038/s41563-019-0412-6]. Scalable ZnO-based catalysts further exemplify PI, delivering threefold higher propene productivity than commercial $KCrO_x/Al_2O_3$ under industrial conditions, with sustained performance via periodic ZnO replenishment [10.1038/s41586-021-03923-3].

※※※

Integrative Understanding and Prospective Outlook: The integration of PI strategies—such as ODHP, redox-decoupling, and advanced catalyst architectures—has redefined PDH efficiency. Future research should prioritize hybrid systems combining exothermic ODHP with endothermic nonoxidative steps to optimize energy utilization. Scalable synthesis of atomically dispersed or nanostructured catalysts (e.g., Pt-Ge-UTL with >99% selectivity and 4,200-hour stability [10.1038/s41929-023-00968-7]) could bridge lab-scale innovations to industrial deployment. Additionally, leveraging machine learning for catalyst design and operando characterization to monitor dynamic catalyst states during redox cycles will be critical. The economic viability of shale-derived propane feedstock [10.1021/acscatal.8b04701] further underscores the need for PI solutions that align with global propylene demand projections (191 Mt by 2030 [10.1021/jacs.2c12970]).

<References>
10.1016/j.cej.2007.11.009
10.1126/science.aaf7885
10.1016/j.cej.2014.09.107
10.1016/S0021-9517(02)00015-5
10.1038/s41467-018-06174-5
10.1021/acscatal.0c03286
10.1038/s41467-023-37261-x
10.1038/s41563-019-0412-6
10.1038/s41586-021-03923-3
10.1038/s41929-023-00968-7
10.1021/acscatal.8b04701
10.1021/jacs.2c12970
</References>

24. Reactors and Process Considerations - What are the techno-economic factors governing process viability?

The viability of propane dehydrogenation (PDH) processes is intricately tied to reactor design and catalytic systems, where techno-economic factors such as catalyst stability, selectivity, energy efficiency, and operational longevity govern commercial feasibility. Boron nitride (BN)-supported Pt-Sn alloys exemplify advanced catalytic architectures, where BN's inertness and hydrophobicity mitigate coking while promoting metal migration and alloy formation (e.g., PtSn/$SnPt_3$), enhancing propylene selectivity and thermal stability [10.1016/j.cej.2007.11.009]. Computational studies using density functional theory (DFT) elucidate reaction mechanisms, such as homolytic C-H bond cleavage at Zr_3+ sites on ZrO_2 surfaces, where oxygen vacancies lower activation barriers by stabilizing propane adsorption intermediates [10.1016/j.jcat.2019.02.012].

Redox-decoupling strategies, such as site isolation of vanadium oxide in silica matrices, suppress consecutive propylene combustion by limiting active site aggregation, particularly at V_2O_5 loadings below 10 wt% [10.1016/S0021-9517(02)00015-5]. Atomically dispersed Pt catalysts, stabilized by K^+ or confined within MFI zeolite channels, minimize sintering and side reactions (e.g., C-C cracking) through electronic modification (e.g., Sn doping) and spatial constraints, achieving >90% propylene selectivity [10.1021/acscatal.0c03286; 10.1038/s41563-019-0412-6]. Hydrogen co-feeding further enhances process economics by reducing coke formation via hydrogenation of carbonaceous precursors, while lowering CH bond-breaking energy barriers [10.1021/acscatal.0c03381].

Surface engineering, such as oxygen vacancies and chloride coverage on CeO_2, facilitates radical-mediated pathways (e.g., O_2^{2-}-Cl• species), reducing activation energies for propane conversion. NiO-modified CeO_2 enhances HCl activation via the Deacon reaction, synergistically lowering energy demands [10.1021/acscatal.8b00650]. Chemical looping systems leverage lattice oxygen from transition metal oxides (e.g., V-O), avoiding gas-phase O_2 and

suppressing overoxidation. Surface oxygen p-band centers near the Fermi level and coordinatively unsaturated vanadium atoms are critical for selective C-H activation [10.1021/acscatal.8b04701]. Pretreatments like CO exposure on ZrO_2 increase oxygen vacancy density, boosting propylene formation rates sevenfold [10.1038/s41467-018-06174-5].

Economic scalability is further influenced by sulfur-doped carbon matrices stabilizing sub-nm metal clusters at 700°C, enhancing interfacial electronic effects for sustained activity [10.1038/s41467-021-23426-z]. Subsurface Ni in BN-supported systems weakens B-O bonds, reducing O-H cleavage barriers to 0.26 eV, enabling low-temperature operation [10.1038/s41467-023-37261-x]. Binuclear ZnOx species, stabilized by defective OH groups in zeolites, exhibit 100–300× higher turnover frequencies than isolated sites, driven by optimized C-H activation via oxygen vacancies [10.1038/s41586-021-03923-3]. Geometric confinement of Pt_4 clusters in Ge-rich zeolites (via Pt-O-Ge bonds) and 1D armchair BN edges further suppress overoxidation, balancing radical stabilization and selectivity [10.1038/s41929-023-00968-7; 10.1126/science.aaf7885].

※※※

Integrative Understanding and Prospective Outlook: The integration of computational modeling (e.g., DFT) with advanced catalyst synthesis (e.g., atomic dispersion, confinement) has delineated pathways to optimize PDH energetics and selectivity. Future efforts should prioritize scalable synthesis of defect-engineered supports (e.g., BN, ZrO_2) and multifunctional alloys (e.g., Pt-Sn, Ni-BO_x) to balance activity and stability under industrial conditions. Chemical looping and hydrogen co-feeding systems warrant pilot-scale validation to assess energy efficiency gains and coke mitigation. Emerging areas such as dynamic reactor modeling, leveraging real-time catalyst state monitoring (e.g., oxygen vacancy density), could further refine process economics. Additionally, exploring non-precious metal catalysts (e.g., Fe-, Co-based systems) guided by machine learning-driven DFT screening may reduce reliance on Pt-group metals, aligning with sustainability goals.

<References>
10.1016/j.cej.2007.11.009
10.1016/j.jcat.2019.02.012
10.1016/S0021-9517(02)00015-5
10.1021/acscatal.0c03286
10.1038/s41563-019-0412-6
10.1021/acscatal.0c03381
10.1021/acscatal.8b00650
10.1021/acscatal.8b04701
10.1038/s41467-018-06174-5
10.1038/s41467-021-23426-z
10.1038/s41467-023-37261-x
10.1038/s41586-021-03923-3
10.1038/s41929-023-00968-7
10.1126/science.aaf7885
</References>

25. Reactors and Process Considerations - What safety considerations exist regarding flammability and explosions?

The deployment of propane dehydrogenation (PDH) catalysts in industrial reactors necessitates rigorous safety protocols to mitigate flammability and explosion hazards inherent to the process. High-temperature operation (typically 500–700°C) and the presence of propane, propylene, and hydrogen—all highly flammable gases—create an environment where thermal runaway, uncontrolled exothermic reactions, or gas-phase ignition could lead to catastrophic events [10.1016/j.jcat.2019.02.012]. Industrial processes such as Catofin and Oleflex, which utilize CrOx/Al2O3 and Pt/Al2O3 catalysts, respectively, require precise control over reactor temperature profiles and gas-phase composition to avoid localized hot spots or accumulation of explosive mixtures [10.1016/j.jcat.2019.02.012].

Catalyst design plays a critical role in minimizing these risks. For instance, boron nitride (BN)-supported Pt-Sn alloys exhibit enhanced thermal stability and reduced coke formation compared to γ-Al2O3-supported systems, thereby lowering the likelihood of reactor blockages and subsequent pressure surges that could trigger explosions [10.1016/j.cej.2007.11.009]. The inert nature of BN supports suppresses unwanted side reactions, such as deep dehydrogenation or cracking, which generate lighter hydrocarbons (e.g., methane) with lower autoignition temperatures [10.1016/j.cej.2007.11.009]. Similarly, subnanometric PtSn clusters confined within zeolite frameworks (e.g., K-PtSn@MFI) demonstrate exceptional stability under cyclic operation, reducing the frequency of catalyst regeneration cycles that involve high-temperature oxidative environments prone to flashback events [10.1038/s41563-019-0412-6].

Mitigation strategies often focus on optimizing catalyst architectures to lower operating temperatures. For example, Ni@BOx/BN core-shell catalysts leverage subsurface boron oxide overlayers to modulate electronic interactions, enabling high propane conversion at reduced temperatures (⩽600° C), thereby diminishing thermal stress on reactor materials and minimizing the risk of hydrogen embrittlement [10.1038/s41467-023-37261-x]. Additionally, sulfur-doped carbon-supported PtSn alloys (PtSn/SC) exhibit low deactivation rates (0.003 h^{-1}), extending catalyst longevity and reducing the need for frequent shutdowns—a critical factor in avoiding startup/shutdown-related ignition hazards [10.1038/s41467-021-23426-z].

Process engineering measures, such as inert gas purging, pressure relief systems, and advanced monitoring of oxygen ingress in hydrogen-rich streams, are essential complements to catalyst design. For instance, ZrO2-based catalysts, which outperform conventional Pt and CrOx systems in activity, benefit from reactor configurations that integrate real-time gas chromatography to detect trace oxygen levels, preventing unintended oxidation reactions that could escalate to deflagration [10.1038/s41467-018-06174-5].

※※※

Integrative Understanding and Prospective Outlook: The interplay between catalyst innovation and reactor engineering underscores a paradigm shift toward inherently safer PDH processes. Future research should prioritize multifunctional catalysts that synergistically suppress coke formation, lower operating temperatures, and resist sintering—attributes exemplified by BN-supported alloys and zeolite-confined clusters. Emerging computational tools, such as machine learning-driven reactor simulations, could predict transient hazardous scenarios (e.g., feed composition fluctuations) and guide the development of adaptive catalyst systems. Furthermore, integrating flame-arresting materials into catalyst supports or reactor linings may offer passive safety enhancements. Advances in operando characterization techniques, as applied to sulfur-stabilized PtSn clusters or Ni@BOx/BN architectures, could unravel dynamic surface phenomena under near-explosive conditions, informing the design of next-generation catalysts with built-in safety mechanisms.

<References>
10.1016/j.jcat.2019.02.012
10.1016/j.cej.2007.11.009
10.1038/s41563-019-0412-6
10.1038/s41467-023-37261-x
10.1038/s41467-021-23426-z
10.1038/s41467-018-06174-5
</References>

26. Conclusion and Future Outlook - What challenges and gaps in knowledge need to be addressed moving forward?

The development of propane dehydrogenation (PDH) catalysts continues to face significant thermodynamic and kinetic challenges. Thermodynamically, the endothermic nature of PDH ($\Delta H = +124$ kJ/mol) necessitates high operating temperatures (550–700°C), which exacerbate side reactions such as propane cracking and propylene overoxidation to COx, thereby reducing selectivity [10.1016/j.cej.2007.11.009]. While hydrogen removal via steam-induced partial pressure reduction improves equilibrium conversion, elevated temperatures (>700°C) remain unavoidable, leading to irreversible catalyst deactivation through mechanisms like Pt sintering (1.8–5.2% Pt loss at 570–600°C) and coke deposition (5.9 wt% for nanoparticle catalysts) [10.1038/s41929-023-00968-7], [10.1021/acscatal.0c03286]. Kinetic limitations further arise from dynamic structural changes in active sites, such as the in situ formation of ZnOx species under reductive conditions, which complicates the establishment of robust structure-activity relationships [10.1038/s41586-021-03923-3]. Operando characterization techniques are hindered by these high-temperature conditions, obscuring real-time insights into catalytic mechanisms [10.1016/j.jcat.2019.02.012].

Coke accumulation remains a critical bottleneck, with coke indices ranging from 0.14 to 0.32 depending on steam co-feeding strategies [10.1016/j.cej.2014.09.107]. While atomically dispersed Pt catalysts reduce coke content to 1.5 wt%, their long-term stability under cyclic regeneration (70 PDH/regeneration cycles) remains underexplored [10.1021/acscatal.0c03286], [10.1038/s41467-

018-06174-5]. Kinetic optimization also faces tradeoffs between conversion and selectivity; for instance, boron nitride (BN) catalysts achieve 61.6% propylene selectivity at only 2.9% conversion, highlighting the need for materials that decouple these parameters [10.1021/jacs.2c12970]. Furthermore, fluidized-bed systems exhibit productivity disparities (0.14 vs. 0.07 kgpropylene/kgcat·h under cyclic vs. steady conditions), emphasizing the role of reactor engineering in time-averaged yields [10.1016/S0021-9517(02)00015-5].

※※※

Integrative Understanding and Prospective Outlook

The interplay between thermodynamic constraints and kinetic inefficiencies underscores the necessity for multifunctional catalyst designs. Future research should prioritize *in situ* and operando characterization techniques capable of withstanding high temperatures, such as advanced X-ray absorption spectroscopy paired with computational modeling (e.g., DFT-calculated activation barriers for Cl•-mediated C3H8 activation at 0.91 eV [10.1021/acscatal.8b00650]). Single-atom catalysts (SACs) with modulated electronic structures, exemplified by Pt/SC's low deactivation rate (0.005 h^{-1}), offer pathways to suppress coke and metal sintering, but their scalability and regeneration kinetics require systematic study [10.1038/s41467-021-23426-z]. Hybrid systems integrating oxidative dehydrogenation (ODHP) for exothermic heat management (45% energy savings) with nonoxidative PDH could balance selectivity and energy efficiency, though challenges like propylene overoxidation must be mitigated [10.1021/acscatal.8b00650]. Additionally, machine learning-driven discovery of metastable active sites, such as Zr_3cus sites generated via lattice oxygen release, may accelerate the development of thermally robust catalysts [10.1016/j.jcat.2019.02.012]. Finally, circular reactor designs that leverage coke combustion heat (≈10% selectivity) to offset PDH endothermicity could redefine process sustainability, provided regeneration protocols are optimized to prevent structural degradation [10.1038/s41467-018-06174-5].

<References>
10.1016/j.cej.2007.11.009
10.1038/s41929-023-00968-7
10.1021/acscatal.0c03286
10.1038/s41586-021-03923-3
10.1016/j.jcat.2019.02.012
10.1016/j.cej.2014.09.107
10.1038/s41467-018-06174-5
10.1021/jacs.2c12970
10.1016/S0021-9517(02)00015-5
10.1021/acscatal.8b00650
10.1038/s41467-021-23426-z
</References>

27. Conclusion and Future Outlook - How can high-throughput computations accelerate catalyst screening?

The integration of high-throughput computational methods into propane dehydrogenation (PDH) catalyst design has emerged as a transformative strategy, as evidenced by density functional theory (DFT) studies across diverse catalytic systems. For instance, DFT calculations on PtSn alloys elucidated energy barriers for dehydrogenation pathways and CO adsorption characteristics, revealing that hydroxyl-assisted pathways exhibit higher activation energies compared to direct dehydrogenation [10.1016/j.cej.2014.09.107]. Similarly, ZrO₂-based catalysts were computationally modeled to identify Zrcus sites as critical for propane adsorption, with charge distribution at Zr centers stabilizing intermediates and transition states during homolytic C-H bond cleavage [10.1016/j.jcat.2019.02.012]. These insights underscore the role of electronic structure tuning, exemplified by positively charged single Pt atoms predicted to enhance activity while mitigating deep dehydrogenation through doping strategies [10.1021/acscatal.0c03286].

However, limitations persist in conventional DFT approaches, such as idealized surface models that overestimate turnover frequencies (TOFs) by neglecting adsorbate interactions and dynamic surface reconstructions [10.1021/acscatal.0c03381]. Advanced computational frameworks incorporating customized Hubbard *U* parameters (e.g., *U*_{eff} = 3.2 eV for vanadium oxides) have refined predictions of oxygen p-band centers near the Fermi level, directly correlating with C-H activation barriers [10.1021/acscatal.8b04701]. Furthermore, binuclear ZnOx clusters were computationally shown to reduce methyl C-H cleavage activation energies by 0.33 eV compared to single-site configurations, with charge transfer analysis rationalizing their enhanced stability [10.1038/s41586-021-03923-3]. Such studies highlight the potential of high-throughput screening to rapidly evaluate multinuclear active sites and alloy compositions.

※※※

Integrative Understanding and Prospective Outlook: The computational advancements detailed in the referenced studies lay a foundation for accelerated catalyst discovery, yet several frontiers remain unexplored. First, integrating machine learning (ML) with DFT-derived datasets could enable predictive models for alloy composition optimization, particularly for Pt-based systems where sulfur doping enhances electronic stabilization [10.1038/s41467-021-23426-z]. Second, addressing the gap between idealized models and operando conditions—such as adsorbate coverage effects on boron nitride nanotubes (BNNTs) [10.1126/science.aaf7885]—requires multi-scale simulations that couple microkinetic modeling with ab initio molecular dynamics. Third, the discovery of subsurface Ni interactions in B₂O₃/Ni(111) systems, which reduce O-H dissociation barriers to 0.26 eV [10.1038/s41467-023-37261-x], suggests a paradigm shift toward investigating buried interfacial active sites. Future efforts should prioritize high-throughput workflows that screen not only surface geometries but also defect engineering (e.g., oxygen vacancies in CeO₂ [10.1021/acscatal.8b00650]) and confinement effects in zeolitic frameworks [10.1038/s41929-023-00968-7]. By bridging computational insights with operando characterization, next-generation PDH catalysts could achieve unprecedented selectivity and stability.

<References>

10.1016/j.cej.2014.09.107

10.1016/j.jcat.2019.02.012
10.1021/acscatal.0c03286
10.1021/acscatal.0c03381
10.1021/acscatal.8b04701
10.1038/s41586-021-03923-3
10.1038/s41467-021-23426-z
10.1126/science.aaf7885
10.1038/s41467-023-37261-x
10.1021/acscatal.8b00650
10.1038/s41929-023-00968-7
</References>

28. Conclusion and Future Outlook - What in-situ characterization capabilities are needed to better understand catalysts?

Conclusion and Future Outlook: In-Situ Characterization Needs for Propane Dehydrogenation Catalysts

The advancement of propane dehydrogenation (PDH) catalysts hinges on resolving critical knowledge gaps in dynamic active site behavior, coke formation mechanisms, and structure-activity relationships under operando conditions. Operando UV-vis spectroscopy has proven instrumental in elucidating coke species evolution during PDH, particularly over tetragonal ZrO_2 catalysts, but lacks integration with kinetic models to quantify coke-induced deactivation rates [10.1016/j.jcat.2019.02.012]. Similarly, while in-situ XAFS and temporal analysis of products (TAP) reactors have enabled microkinetic simulations incorporating Pt cluster site density (2.8 sites/nm^2) and ZnOx coordination-dependent rate equations, these models often oversimplify lateral adsorbate interactions and transient intermediate speciation [10.1038/s41929-023-00968-7] [10.1038/s41586-021-03923-3].

Key capability gaps include:

1. **Spatiotemporal resolution of active site dynamics**: Current operando techniques like DRIFTS correlate adsorption strength with selectivity but fail to resolve sub-second structural rearrangements of oxygen vacancies or Cl• species during C–H activation (energy barrier: 0.91 eV) [10.1021/acscatal.8b00650].

2. **Quantitative coke profiling**: Operando UV-vis identifies coke precursors but lacks sensitivity to differentiate graphitic vs. amorphous carbon spatial distribution, critical for linking deactivation kinetics (e.g., first-order kd = 0.005 h^{-1}) to crystallite size effects [10.1038/s41467-018-06174-5] [10.1038/s41467-021-23426-z].

3. **Multi-modal correlative imaging**: Integrating XAFS-derived oxidation states with high-pressure STEM could map Pt sintering pathways while maintaining reaction conditions, addressing discrepancies in microkinetic predictions caused by neglecting site heterogeneity [10.1021/acscatal.0c03381].

※※※

Integrative Understanding and Prospective Outlook

The synthesis of operando spectroscopy, kinetic modeling, and atomic-scale imaging presents a transformative opportunity to decode PDH catalysis. Future tools must prioritize **multi-modal platforms** combining time-resolved X-ray photoelectron spectroscopy (tr-XPS) with modulated excitation DRIFTS to disentangle rate-limiting steps from parasitic deactivation pathways. Machine learning-driven analysis of operando datasets could bridge the gap between microkinetic parameters (e.g., pre-exponential factors spanning 10^{12}–10^{13} s^{-1}) and metastable active site configurations. Additionally, advancing **sub-nm tip-enhanced Raman spectroscopy (TERS)** under PDH conditions may resolve how surface Cl coverage modulates O-vacancy regeneration—a critical selectivity determinant. By correlating these insights with ab initio microkinetic frameworks, next-generation catalysts could achieve predictive optimization of propylene yield while suppressing coke by a factor of 1.6–2.0, as suggested by comparative ZnOx vs. KCrOx performance [10.1038/s41586-021-03923-3].

<References>
10.1016/j.jcat.2019.02.012
10.1038/s41929-023-00968-7
10.1038/s41586-021-03923-3
10.1021/acscatal.8b00650
10.1038/s41467-018-06174-5
10.1038/s41467-021-23426-z
10.1021/acscatal.0c03381
</References>

29. Conclusion and Future Outlook - What transformational catalyst architectures could provide breakthroughs?

The reviewed studies collectively underscore the pivotal role of catalyst architecture in propane dehydrogenation (PDH) performance, with transformative potential arising from innovations in support materials, alloy design, and defect engineering. The hydrophobic nature of boron nitride (BN) supports enhances catalytic stability by expelling water during hydrogen oxidation, while PtSn alloy formation on BN surfaces optimizes active site isolation and electronic modulation, leading to improved selectivity [10.1016/j.cej.2007.11.009]. Similarly, Pt-S interactions in sulfur-doped carbon matrices stabilize subnanometric Pt clusters, achieving 98% propylene selectivity through interfacial electronic effects that suppress sintering and coke formation [10.1038/s41467-021-23426-z].

ZrO_2-based catalysts demonstrate crystallite size-dependent oxygen vacancy generation, where smaller crystallites (<5 nm) exhibit 70× higher activity due to increased Zrcus site density [10.1038/s41467-018-06174-5]. This strategy is extendable to non-reducible oxides via defect engineering, though challenges persist in maintaining active site integrity under dynamic reaction conditions [10.1016/j.jcat.2019.02.012]. Vanadium oxide dispersion in silica matrices (<10 wt% V_2O_5) isolates active sites, reducing propylene combustion and enhancing selectivity through suppressed consecutive reactions [10.1016/S0021-9517(02)00015-5].

Single-atom Pt catalysts and under-coordinated metal sites (e.g., Pt-O-Ge coordination number = 2.1) lower activation barriers by 0.32 eV per additional Pt-Ge bond, leveraging electronic effects and geometric confinement in zeolitic frameworks [10.1038/s41929-023-00968-7]. Surface oxygen vacancies and chloride coverage further modulate selectivity, with Ni^{2+}-doped CeO_2 nanorods achieving 80% propylene selectivity by balancing oxygen vacancy density and overoxidation suppression [10.1021/acscatal.8b00650].

※※※

Integrative Understanding and Prospective Outlook: The convergence of advanced characterization techniques and computational modeling has elucidated structure-activity relationships critical for next-generation PDH catalysts. Future breakthroughs may emerge from:

1. **Dynamic Site Engineering**: Designing catalysts with adaptive surface configurations (e.g., reversible Sn^{2+}/Sn^{4+} redox cycles) to maintain activity under coke-forming conditions [10.1016/j.cej.2014.09.107].
2. **Hierarchical Hybrid Supports**: Integrating hydrophobic BNNTs with redox-active oxides (e.g., ZrO_2) to synergize water expulsion and oxygen vacancy generation [10.1126/science.aaf7885].
3. **Radical-Mediated Pathways**: Optimizing metal-oxide interfaces (e.g., Ni-encapsulated boron oxide overlayers) to enhance radical generation/regeneration cycles while minimizing side reactions [10.1038/s41467-023-37261-x].
4. **Multi-Scale Confinement**: Leveraging zeolitic frameworks for regioselective placement of subnanometric bimetallic clusters (e.g., Pt-Ge) to stabilize high-energy intermediates [10.1038/s41563-019-0412-6].

<References>
10.1016/j.cej.2007.11.009
10.1038/s41467-021-23426-z
10.1038/s41467-018-06174-5
10.1016/j.jcat.2019.02.012
10.1016/S0021-9517(02)00015-5
10.1038/s41929-023-00968-7
10.1021/acscatal.8b00650
10.1016/j.cej.2014.09.107
10.1126/science.aaf7885
10.1038/s41467-023-37261-x
10.1038/s41563-019-0412-6
</References>

Recent Advances in Propane Dehydrogenation Catalysis: From Molecular Understanding to Industrial Application (Generated by DeepSeek R1+V3)

Abstract
This review comprehensively examines recent advances in propane dehydrogenation (PDH) catalysis, focusing on catalyst design, reaction mechanisms, and process optimization. PDH has emerged as a critical on-purpose propylene production process, gaining significance particularly following the shale gas revolution which has made cost-effective propane feedstocks widely available. We first articulate propylene's role as a cornerstone of the chemical industry and the strategic importance of PDH in addressing the supply-demand gap. Subsequently, we systematically analyze how catalysts facilitate PDH by lowering activation energy barriers, providing detailed discussions on structure-activity relationships across major catalyst categories including metal oxides (Cr_2O_3, Ga_2O_3), supported metals (Pt, Pd, Ir), bimetallic alloys (Pt-Sn, Pt-Ga, Pt-Zn), and zeolites (MFI, FER, BEA). Special emphasis is placed on the pivotal role of density functional theory (DFT) calculations and microkinetic modeling in elucidating reaction mechanisms and predicting catalytic performance, alongside the crucial contributions of advanced characterization techniques such as in-situ spectroscopy and high-resolution microscopy in understanding working catalysts. Furthermore, we evaluate the impact of different reactor configurations (fixed bed, fluidized bed, membrane reactors) and process intensification strategies on the techno-economic viability of PDH. Finally, we identify challenges and knowledge gaps in the field, highlighting the potential of high-throughput computations, advanced in-situ characterization, and transformational catalyst architectures in accelerating breakthroughs in PDH technology. This review provides researchers with a panoramic perspective of propane dehydrogenation catalytic chemistry and charts a course for developing the next generation of efficient, stable, and sustainable industrial PDH processes.

1. Introduction - What is the role of propylene in the chemical industry and why is propane dehydrogenation important?

Propylene is a cornerstone of the chemical industry, serving as a critical feedstock for the production of a wide array of chemicals and materials. Its applications span the manufacture of polypropylene, acrylonitrile, propylene oxide, and cymene, among others, making it indispensable in sectors ranging from plastics to pharmaceuticals [10.1002/advs.202207756]. The global demand for propylene has been steadily increasing, with production capacity reaching approximately 130 megatons in 2019 and projected to escalate to 191 megatons by 2030, highlighting a significant supply-demand gap [10.1002/advs.202207756]. Propane dehydrogenation (PDH) has emerged as a vital process to address this gap, currently accounting for 10% of global propylene production [10.1002/advs.202207756].

PDH is particularly advantageous as it offers an economical and environmentally friendly alternative to traditional methods such as thermal or catalytic cracking of naphtha, which are energy-intensive and generate substantial CO_2 emissions [10.1002/anie.201507119]. The process leverages the abundance of propane, particularly from shale gas, which has become a cost-

effective feedstock due to the shale gas revolution [10.1002/anie.201508731]. This shift has made PDH a commercially attractive route for on-purpose propylene production, especially as traditional methods like steam cracking and fluid catalytic cracking (FCC) are increasingly unable to meet the growing demand [10.1002/anie.201800123].

The importance of propylene extends beyond its role as a chemical intermediate. It is a key building block for polymers, solvents, dyes, resins, fibers, and drugs, underscoring its versatility and economic value [10.1002/anie.202005968]. The rising demand for propylene derivatives, such as polypropylene used in non-woven fabrics for personal protective equipment during the COVID-19 pandemic, further emphasizes its critical role in modern industry [10.1016/j.apcatb.2021.120731]. As the chemical industry continues to evolve, PDH is poised to play an increasingly significant role in bridging the supply-demand gap, driven by advancements in catalytic technologies and the availability of cost-effective propane feedstocks [10.1016/j.apcatb.2020.119089].

※※※
Integrative understanding and prospective outlook: The growing demand for propylene, coupled with the limitations of traditional production methods, underscores the need for innovative approaches to propylene production. Propane dehydrogenation (PDH) has emerged as a key technology in this regard, offering a sustainable and economically viable alternative. Future research should focus on developing more efficient and selective catalysts to enhance PDH performance, reduce energy consumption, and minimize environmental impact. Additionally, exploring alternative feedstocks and integrating PDH with other industrial processes could further optimize propylene production. The continued evolution of PDH technologies, driven by advancements in catalysis and process engineering, will be crucial in meeting the escalating global demand for propylene and ensuring the sustainability of the chemical industry.
<References>
10.1002/advs.202207756
10.1002/anie.201507119
10.1002/anie.201508731
10.1002/anie.201800123
10.1002/anie.202005968
10.1016/j.apcatb.2021.120731
10.1016/j.apcatb.2020.119089
</References>

2. Introduction - How do catalysts enable propane dehydrogenation through lowering activation barriers?

Catalysts play a pivotal role in propane dehydrogenation (PDH) by lowering the activation energy barriers required for the reaction to proceed. This is achieved through various mechanisms that facilitate the breaking of C-H bonds in propane, which is the rate-determining step in the process. One of the primary mechanisms involves the use of coordinatively unsaturated metal sites, such as

Cr^{3+} in CrOx/Al2O3 catalysts, which act as active sites for PDH by enabling heterolytic C-H dissociation [10.1002/advs.202207756]. Similarly, Pt-based catalysts employ the Horiuti-Polanyi mechanism, where the first C-H bond cleavage is considered the rate-determining step, and the presence of Sn promoters enhances the catalyst's selectivity and stability by modifying the electronic structure of Pt [10.1002/advs.202207756].
Ga-based catalysts, on the other hand, utilize both carbenium and alkyl mechanisms. In the carbenium mechanism, propane is initially activated on Brønsted acid sites (BAS) to form a carbenium ion, while Ga facilitates the recombination of hydrogen atoms on the catalyst surface to form H_2, thereby increasing the reaction rate. The alkyl mechanism involves direct activation of propane by Ga species, forming Ga-C_3H_7 intermediates, followed by β-H elimination to produce propylene and Ga-H species [10.1002/advs.202207756].
In addition to these mechanisms, the role of Lewis acid-base pairs is highlighted in Ga/ZSM-5 catalysts, where Ga^+ ions and zeolite-framework basic oxygen anions (Ga^+-Z-O^-) activate C-H bonds. The formation of binuclear Ga^{3+} complexes further lowers the activation energy barriers for H_2 recombination compared to mononuclear GaO^+ ions, as demonstrated by DFT calculations [10.1002/anie.200702463]. Similarly, coordinatively unsaturated Zr^{4+} cations (Zrcus) near anion vacancies in ZrO_2-based catalysts facilitate C-H bond activation and β-hydrogen elimination, with activation energies around 170 kJ/mol [10.1002/anie.201508731].
The use of oxygen defects in $MnWO_4$ nanorods and the geometric separation of MnOx chains by W_2O_8 units also enhance catalytic activity and selectivity by enabling reversible replenishment and depletion of oxygen defects, which suppress overoxidation to CO/CO_2 [10.1002/anie.201510201]. Furthermore, vanadium-based catalysts, such as V_2O_3, exhibit low dehydrogenation barriers (1.12 eV) due to the presence of hydroxyl groups that block exposed V^{3+} sites, thereby reducing coke formation [10.1002/anie.201800123].

※※※
Integrative understanding and prospective outlook: The advancements in propane dehydrogenation catalysts underscore the importance of tailoring active sites to lower activation energy barriers and enhance selectivity. Future research could focus on the development of novel catalyst architectures, such as single-atom catalysts (SACs) and bimetallic alloys, which offer precise control over active site geometry and electronic properties. Additionally, the integration of computational modeling, such as DFT, with experimental studies can provide deeper insights into the mechanistic pathways and transition states involved in PDH. Exploring the use of renewable energy sources, such as electric fields or light, to drive PDH reactions could also open new avenues for sustainable propylene production. Furthermore, the design of catalysts with enhanced resistance to coking and sintering will be crucial for improving the longevity and efficiency of PDH processes.
<References>
10.1002/advs.202207756
10.1002/anie.200702463
10.1002/anie.201508731
10.1002/anie.201510201
10.1002/anie.201800123
</References>

3. Introduction - What are the major classes of catalysts studied for propane dehydrogenation?

Propane dehydrogenation (PDH) has been extensively studied, with several major classes of catalysts explored to enhance propylene production. The primary catalyst classes include metal oxides, supported metals, alloys, and zeolites. Metal oxides such as CrO_x/Al_2O_3 and VO_x/Al_2O_3 are widely used in industrial processes like the Catofin and Oleflex processes, respectively [10.1002/advs.202207756]. Supported metals, particularly Pt-based catalysts, are prominent due to their high activity and selectivity. For instance, $PtSn/Al_2O_3$ is a key catalyst in the Oleflex process, where Sn acts as a promoter to reduce coke formation and improve stability [10.1016/j.jcat.2020.03.037]. Alloy catalysts, such as PtGa and PtZn, have also shown enhanced performance due to their unique electronic and structural properties [10.1021/acscatal.1c00331]. Zeolite-based catalysts, including Ga/H-ZSM-5 and Zn/H-ZSM-5, are notable for their high selectivity and stability in PDH reactions [10.1016/j.jcat.2019.11.026]. Specific examples include $Ga_2O_2^{2+}$ sites in H-ZSM-5, which exhibit a turnover frequency (TOF) at least one order of magnitude higher than other Ga-based catalysts [10.1002/anie.200702463]. Additionally, nanocarbon materials have emerged as promising metal-free catalysts, offering high selectivity and stability [10.1016/S1872-2067(19)63360-7].

※※※

Integrative understanding and prospective outlook: The advancements in propane dehydrogenation catalysts highlight the importance of optimizing catalyst design to balance activity, selectivity, and stability. Future research should focus on developing non-toxic, cost-effective alternatives to traditional Pt- and Cr-based systems. Emerging materials such as single-atom catalysts (SACs), high-entropy intermetallics (HEIs), and boron-based systems offer promising avenues for innovation. Additionally, the integration of advanced characterization techniques and computational modeling can provide deeper insights into catalyst mechanisms, enabling the rational design of next-generation PDH catalysts. Exploring synergistic effects between different catalyst classes, such as combining metal oxides with zeolites or nanocarbons, could further enhance catalytic performance and sustainability.

<References>
10.1002/advs.202207756
10.1016/j.jcat.2020.03.037
10.1021/acscatal.1c00331
10.1016/j.jcat.2019.11.026
10.1002/anie.200702463
10.1016/S1872-2067(19)63360-7
</References>

4. Theoretical Principles and Computational Approaches - What are the thermodynamic limitations and kinetic considerations in propane dehydrogenation?

Propane dehydrogenation (PDH) is a thermodynamically constrained and kinetically complex process, primarily due to its highly endothermic nature (ΔH = 124.3 kJ/mol) and equilibrium limitations. The reaction is favored at high temperatures (550–700°C) and low pressures, as these conditions shift the equilibrium toward propylene production. However, elevated temperatures also accelerate undesirable side reactions such as cracking, coking, and sintering, which lead to rapid catalyst deactivation. Kinetic factors include turnover frequencies (TOF), which vary significantly depending on catalyst composition, support, and preparation methods. For instance, Pt-based catalysts exhibit TOF values ranging from 4.6 to 12,600 h^{-1}, while CrOx/Al2O3 shows a lower TOF of 20.4 h^{-1} [10.1002/advs.202207756].

The rate-determining step (RDS) in PDH often shifts between C-H bond cleavage and H_2 desorption, depending on the catalyst and reaction conditions. For example, DFT calculations reveal that the activation energy for H_2 recombination on mononuclear GaO^+ species is prohibitively high (305 kJ/mol), whereas binuclear Ga clusters exhibit lower barriers (147–211 kJ/mol) [10.1002/anie.200702463]. Additionally, the introduction of steam or CO_2 can enhance kinetic stability by reducing coke formation and shifting equilibrium via the reverse water-gas shift reaction (RWGS) [10.1016/j.jcat.2005.03.001].

Thermodynamic equilibrium conversions are typically low at moderate temperatures (e.g., 0.5% at 300°C), but electroassisted PDH can achieve yields far beyond these limits (e.g., 19.3% at 300°C) [10.1002/anie.202300744]. Oxidative dehydrogenation (ODHP) avoids thermodynamic constraints by being exothermic and operates at lower temperatures, with activation energies ranging from 62.59 to 150.68 kJ/mol depending on the catalyst [10.1002/anie.202307470].

※※※

Integrative understanding and prospective outlook: The thermodynamic and kinetic challenges of PDH necessitate innovative catalyst designs and process optimizations. Future research could focus on developing catalysts with enhanced resistance to coking and sintering, such as single-atom catalysts (SACs) or bimetallic systems. Additionally, integrating electroassisted or oxidative dehydrogenation methods could overcome equilibrium limitations and improve energy efficiency. Computational approaches, including DFT and microkinetic modeling, will play a crucial role in identifying optimal catalyst structures and reaction pathways. Emerging technologies, such as chemical looping and tandem catalysis, offer promising avenues for achieving higher propylene yields while minimizing energy consumption and environmental impact.

<References>
10.1002/advs.202207756
10.1002/anie.200702463
10.1016/j.jcat.2005.03.001
10.1002/anie.202300744
10.1002/anie.202307470
</References>

5. Theoretical Principles and Computational Approaches - How are DFT calculations used to explore reaction mechanisms and pathways?

Density functional theory (DFT) calculations have been extensively applied to study the mechanisms and pathways of propane dehydrogenation (PDH), providing critical insights into the structural stability of active sites, reaction energetics, and catalytic performance. For instance, DFT studies on Ga-based catalysts revealed significant differences in the stability and reactivity of Ga+ and Ga2O22+ species. Specifically, Ga2O22+ species exhibited lower activation energy for H2 recombination (147 kJ/mol) compared to isolated [HGa3+OH]+ sites (242 kJ/mol), highlighting the role of hydrogen bonding in stabilizing intermediates [10.1002/advs.202207756]. Similarly, DFT calculations on hydroxylated binuclear Ga complexes (e.g., Ga_2O_2 core structures) quantified reaction and activation energies for H_2/H_2O desorption, with computed Ga-Ga distances (2.98 Å) matching EXAFS data, further validating the computational models [10.1002/anie.200702463].

In the context of VOx catalysts, DFT simulations demonstrated that V2O3 structures exhibit lower dehydrogenation barriers (1.12 eV) compared to V2O5H2 (1.98 eV), while hydroxyl groups increase barriers and block V^{3+} sites, reducing coke formation [10.1002/anie.201800123]. Additionally, DFT studies on Pt-based catalysts revealed that increasing Sn content in Pt-Sn alloys reduces the binding strength of the 1-propyl intermediate, thereby inhibiting deep dehydrogenation and coke formation [10.1016/j.jcat.2018.09.006]. These findings underscore the importance of electronic and geometric effects in modulating catalytic activity and selectivity.

DFT calculations have also been instrumental in elucidating the role of oxygen vacancies and surface defects in PDH. For example, studies on ZrO_2-based catalysts identified two neighboring coordinatively unsaturated Zr cations (Zrcus) as active sites, with monoclinic ZrO_2 showing lower methylene C-H activation barriers (0.16 eV) compared to tetragonal ZrO_2 (0.42–0.61 eV) [10.1016/j.jcat.2019.02.012]. Furthermore, DFT simulations on PtZn intermetallic alloys revealed that isolated Pt atoms within [PtZn4] ensembles facilitate initial C-H activation but block over-dehydrogenation, explaining high selectivity and coke resistance [10.1016/j.chempr.2020.10.008].

The integration of DFT with experimental techniques has further enhanced the understanding of PDH mechanisms. For instance, DFT calculations on β-Ga2O3 surfaces identified tricoordinated GaIII sites adjacent to oxygen vacancies as weak Lewis acid sites, with computational models correlating Ga-H bond lengths to IR vibration frequencies [10.1016/j.jcat.2022.02.025]. Similarly, DFT studies on PtMn alloys revealed energy barriers for propane C-H activation and propene desorption, with PtMn alloys showing an optimal balance between activation and desorption, explaining catalytic performance differences [10.1016/j.jcat.2020.06.016].

※※※

Integrative understanding and prospective outlook: The advancements in DFT calculations have significantly deepened the mechanistic understanding of propane dehydrogenation, particularly in elucidating the roles of active sites, surface defects, and alloying effects. Future research could focus on integrating machine learning with DFT to accelerate catalyst discovery and optimization. Additionally, exploring the dynamic behavior of catalysts under operando conditions using ab initio molecular dynamics (AIMD) could provide further insights into reaction pathways and catalyst stability. The development of multi-scale models combining DFT with microkinetic simulations may also enable the design of more efficient and selective PDH catalysts.

<References>
10.1002/advs.202207756
10.1002/anie.200702463
10.1002/anie.201800123
10.1016/j.jcat.2018.09.006
10.1016/j.jcat.2019.02.012
10.1016/j.chempr.2020.10.008
10.1016/j.jcat.2022.02.025
10.1016/j.jcat.2020.06.016
</References>

6. Theoretical Principles and Computational Approaches - What microkinetic models have provided insights into dehydrogenation kinetics?

Microkinetic modeling has emerged as a pivotal tool in understanding the complex kinetics of propane dehydrogenation (PDH), offering insights into reaction mechanisms, rate-determining steps, and catalyst performance. Several studies have employed microkinetic models to elucidate the kinetics of PDH, often integrating experimental data with computational approaches. For instance, a study by [10.1002/advs.202207756] utilized microkinetic modeling to analyze reaction orders, revealing negative H_2 orders in both dehydrogenation and cracking reactions. The model also highlighted the independence of the dehydrogenation-to-cracking (D/C) ratio from reactant pressures, providing a nuanced understanding of the reaction dynamics. Additionally, the study discussed rate equations normalized to Ga/Al ratios, incorporating parameters such as turnover frequency (TOFGa) and competitive adsorption terms, though explicit models were not detailed.

Another significant contribution comes from [10.1016/j.cej.2004.03.006], where Langmuir-type and Mars-van Krevelen rate equations were employed in microkinetic modeling. The study tabulated parameters like rate constants (k_1, k_2) and adsorption equilibrium constants (K), offering a comprehensive framework for understanding propane consumption and reoxidation kinetics. Similarly, [10.1016/j.jcat.2020.03.037] employed kinetic Monte Carlo (kMC) simulations to model 59 elementary steps, incorporating lateral interactions and coverage-dependent Gibbs free energy barriers. The model quantified turnover frequencies (TOFs), selectivity, and deactivation rates, with key parameters including a 1.37 eV apparent activation barrier for propane dehydrogenation.

Further advancements in microkinetic modeling are evident in [10.1021/acscatal.0c03381], where a model incorporating adsorbate interactions and coverage-dependent barriers reproduced experimental TOF trends. The study used transition state theory-based rate equations, showing that optimal H_2/C_3H_8 ratios maximize propylene yield by balancing site availability. Similarly, [10.1021/acscatal.1c04844] employed Bayesian model selection and harmonic transition state theory to derive rate equations, validating them against experimental TOFs and activation energies. These studies underscore the importance of microkinetic modeling in bridging theoretical predictions with experimental observations.

Moreover, [10.1038/s41929-021-00730-x] developed a Langmuir-Hinshelwood microkinetic model, identifying CO_2 activation as the rate-determining step in CO_2-assisted PDH. The study derived rate equations and parameters, including reaction orders and activation energies, with simulations matching experimental trends. This approach highlights the potential of microkinetic modeling in optimizing catalyst design and operational conditions for PDH.

※※※

Integrative understanding and prospective outlook: The advancements in microkinetic modeling for propane dehydrogenation have significantly enhanced our understanding of reaction kinetics and catalyst performance. However, challenges remain in fully capturing the complexity of real-world catalytic systems, particularly in accounting for dynamic site blocking, coke formation, and transient reaction conditions. Future research should focus on integrating machine learning and advanced computational techniques to refine microkinetic models, enabling more accurate predictions of catalyst behavior under industrial conditions. Additionally, exploring the interplay between catalyst structure and kinetics through operando characterization techniques could provide deeper insights into active site dynamics and reaction pathways. By addressing these challenges, microkinetic modeling can continue to drive innovation in catalyst design and process optimization for propane dehydrogenation.

<References>
10.1002/advs.202207756
10.1016/j.cej.2004.03.006
10.1016/j.jcat.2020.03.037
10.1021/acscatal.0c03381
10.1021/acscatal.1c04844
10.1038/s41929-021-00730-x
</References>

7. Theoretical Principles and Computational Approaches - How have descriptors been identified correlating catalyst properties to performance?

Theoretical Principles and Computational Approaches - How have descriptors been identified correlating catalyst properties to performance?

The identification of descriptors linking catalyst properties to propane dehydrogenation (PDH) performance has been a focal point in both computational and experimental studies. Key descriptors include the speciation of active sites, support interactions, and electronic/geometric effects. For instance, $Ga_2O_2^{2+}$ sites in H-ZSM-5 exhibit a turnover frequency (TOF) of >2,120 h^{-1}, significantly higher than other Ga-based catalysts, due to the local coordination environment provided by the zeolite support [10.1002/advs.202207756]. Similarly, binuclear Ga_2O_2 structures, identified via EXAFS, show lower H_2 recombination energy, correlating with higher activity compared to mononuclear Ga species [10.1002/anie.200702463].

Support effects are also critical, as demonstrated by the role of framework Al pair density in determining the concentration of active Ga species in PDH [10.1002/advs.202207756]. Computational studies have further revealed that the p-band center of oxygen and the unoccupied d-band center of metals are crucial descriptors, with electron transfer from oxygen's p-band to metal d-bands influencing activity [10.1002/anie.202206758]. Additionally, the hydrogen affinity (EH) and adsorption energy of intermediates, such as propylene, have been identified as universal descriptors, with linear scaling relationships established between these parameters and catalytic performance [10.1016/j.chempr.2020.10.008].

Structure-activity relationships often emphasize the importance of site isolation and electronic modifications. For example, isolated Pt1 sites, geometrically isolated by Pb and electronically enriched by Ca, exhibit reduced coking and enhanced selectivity [10.1002/anie.202107210]. Similarly, the distance between adjacent Pt atoms in Pt2In3 alloys (3.25 Å) induces weak π-adsorption of propylene, facilitating desorption and inhibiting side reactions [10.1016/j.jechem.2023.04.019].

※※※

Integrative understanding and prospective outlook:

The advancements in identifying descriptors for PDH catalysts highlight the interplay between structural, electronic, and support properties in determining catalytic performance. Future research should focus on leveraging machine learning and high-throughput computational screening to accelerate the discovery of novel descriptors and optimize catalyst design. Additionally, exploring the role of dynamic structural changes under reaction conditions and the integration of multi-functional supports could further enhance catalyst stability and selectivity. Emerging fields such as single-atom catalysis and the use of advanced characterization techniques (e.g., in situ spectroscopy and operando studies) offer promising avenues for deeper insights into structure-activity relationships.

<References>
10.1002/advs.202207756
10.1002/anie.200702463
10.1002/anie.202206758
10.1016/j.chempr.2020.10.008
10.1002/anie.202107210
10.1016/j.jechem.2023.04.019
</References>

8. Metal Oxide Catalysts - What metal oxides like Cr2O3, Ga2O3 have shown promising propane dehydrogenation activity?

The study of metal oxide catalysts for propane dehydrogenation (PDH) has revealed significant advancements, particularly with chromium oxide (Cr_2O_3) and gallium oxide (Ga_2O_3) systems. Cr_2O_3-based catalysts, such as those used in the Catofin process, demonstrate high propane conversion rates (45–50%) and selectivity (85–90%) but are limited by environmental concerns due to Cr(VI) toxicity and shorter lifespans (1–2 years) [10.1021/acscatal.2c01374]. Despite these drawbacks, Cr_2O_3 remains a cornerstone of industrial PDH due to its cost-effectiveness and high activity. Recent innovations, such as CrZrOx/SiO_2 catalysts, have shown improved performance, with Cr30Zr70/SiO_2 achieving a space-time yield three times higher than traditional K-CrOx/Al_2O_3 [10.1021/acscatal.9b05063].

Ga_2O_3-based catalysts have emerged as promising alternatives, offering high propylene selectivity and stability. For instance, Ga-CHA zeolites achieve 96% propylene selectivity, while Ga/H-ZSM-5 maintains 90% selectivity with high stability [10.1021/jacs.2c03941]. Trace-Pt-promoted Ga_2O_3/Al_2O_3 catalysts have demonstrated remarkable activity and stability, with propane conversion rates of 12% and 99% selectivity [10.1021/acscatal.0c05454]. Furthermore, Ir1Ga_2O_3 outperforms Pt-promoted Ga_2O_3, highlighting the potential of Ga_2O_3-based systems as environmentally friendly and efficient PDH catalysts [10.1021/acscatal.0c05454].

※※※

Integrative understanding and prospective outlook: The advancements in Cr_2O_3 and Ga_2O_3 catalysts underscore their potential to address the limitations of traditional Pt-based systems, such as high cost and environmental concerns. Future research should focus on optimizing the stability and regeneration of Cr_2O_3 catalysts while mitigating their environmental impact. For Ga_2O_3 systems, exploring novel promoters and support materials could further enhance their activity and selectivity. Additionally, the integration of computational modeling and advanced characterization techniques could accelerate the discovery of next-generation metal oxide catalysts for PDH, paving the way for more sustainable and efficient propylene production processes.

<References>
10.1021/acscatal.2c01374
10.1021/acscatal.9b05063
10.1021/jacs.2c03941
10.1021/acscatal.0c05454
</References>

9. Metal Oxide Catalysts - How does controlling oxidation state, oxygen mobility and acid-base sites modulate catalyst performance?

Metal oxide catalysts play a pivotal role in propane dehydrogenation (PDH) by modulating oxidation states, oxygen mobility, and acid-base properties, which directly influence catalytic performance. The oxidation state of metal centers, such as V^{3+} in vanadium oxides, has been shown to significantly enhance PDH activity, as evidenced by TOF correlations and H_2-TPR experiments [10.1021/acscatal.6b00893]. Similarly, the presence of coordinatively unsaturated tetrahedral Ni(II) sites promotes propylene desorption, thereby inhibiting coking and side reactions [10.1021/acscatal.2c03240].

Oxygen mobility, particularly in redox-active supports like CeO_2, facilitates Mars-van Krevelen (MvK) mechanisms, where lattice oxygen participates in H abstraction and coke combustion. This is further enhanced by the basicity and oxygen-releasing ability of CeO_2, which stabilizes the catalyst and reduces energy barriers for CO_2 activation [10.1038/s41929-021-00730-x]. Additionally, CrOx-doped ZrO_2 systems demonstrate improved reducibility and lower activation barriers for C–H bond cleavage, attributed to hydrogen spillover and the generation of oxygen vacancies [10.1021/acscatal.9b05063].

Acid-base properties also play a critical role in PDH catalysis. Lewis acid-base interactions at metal oxide (MO) sites enhance the coadsorption of amphoteric species, modifying chemisorption energy scaling and transition state energies. For instance, electron-deficient O ions, resulting from charge redistribution around doped metal atoms (e.g., Ir), exhibit enhanced reactivity, as indicated by linear correlations between O p-band centers and H adsorption energies [10.1021/acscatal.0c05454]. Furthermore, phosphorus modification of vanadium catalysts stabilizes lower valence vanadium (V^{4+}) and creates isolated vanadium species, which inhibit intermediate polymerization and enhance propylene desorption [10.1016/j.apcatb.2020.119089].

※※※

Integrative understanding and prospective outlook: The intricate interplay between oxidation states, oxygen mobility, and acid-base properties in metal oxide catalysts offers a rich landscape for optimizing PDH performance. Future research could explore the synergistic effects of multi-metal oxide systems, where the combination of redox-active and acid-base functionalities could further lower activation barriers and enhance selectivity. Advanced characterization techniques, such as in situ spectroscopy and operando studies, could provide deeper insights into the dynamic behavior of active sites under reaction conditions. Additionally, computational modeling, particularly DFT, can guide the rational design of catalysts by predicting the effects of dopants and support interactions on electronic and geometric properties. Emerging fields such as single-atom catalysis and the integration of CO_2 utilization in PDH processes also present promising avenues for sustainable and efficient propane dehydrogenation.

<References>

10.1021/acscatal.6b00893
10.1021/acscatal.2c03240
10.1038/s41929-021-00730-x
10.1021/acscatal.9b05063
10.1021/acscatal.0c05454
10.1016/j.apcatb.2020.119089

</References>

10. Metal Oxide Catalysts - What are the proposed mechanistic scenarios over different metal oxide catalysts?

The mechanistic scenarios for propane dehydrogenation (PDH) over metal oxide catalysts have been extensively studied, with a focus on understanding the reaction steps, intermediates, and active sites. Among the most prominent catalysts are Pt-Sn bimetallic clusters supported on γ-Al_2O_3 nanosheets, where raft-like Pt-Sn clusters with a mean diameter of 1.3 nm are stabilized by pentacoordinate Al^{3+} sites [10.1002/anie.201507119]. These clusters exhibit high activity and selectivity, attributed to the synergistic interaction between Pt and Sn, which modulates the electronic properties of Pt and reduces coke formation.

Another significant class of catalysts includes Cr-based systems, such as Cr_2O_3/Al_2O_3, which are employed in the Catofin process. These catalysts feature isolated Cr^{3+} sites and amorphous CrOx clusters, with redox dynamics between Cr(III) and Cr(VI) playing a critical role in the reaction mechanism [10.1016/j.jcat.2020.03.037]. The addition of promoters like ZrO_2 and NiO further enhances the catalytic performance by improving dispersion and stability [10.1021/acscatal.9b05063].

Vanadium oxide (VOx/Al_2O_3) catalysts have also been widely investigated, with the reaction mechanism following a Mars-van Krevelen (MvK) redox pathway. Isolated VOx species, polyvanadates, and V_2O_5 nanoparticles are identified as active sites, with their activity and selectivity dependent on vanadium density and support interactions [10.1021/acscatal.6b00893].

Ga-based catalysts, particularly Ga/ZSM-5, have shown promise due to the presence of Ga^+ and GaO^+ species at zeolite exchange sites. These catalysts exhibit high propane turnover frequency and selectivity, with Ga^+ ions being more active than GaO^+ species [10.1016/j.jcat.2006.03.004]. Additionally, PtGa alloy nanoparticles, such as those in $Ga37Pt/Al_2O_3$ SCALMS, demonstrate exceptional selectivity (>99%) and productivity, with isolated Pt atoms in a liquid Ga matrix preventing coke formation [10.1021/acscatal.8b04578].

Phosphorus-doped Fe/P/Al catalysts represent another innovative approach, where phosphorus doping promotes the formation of Fe_3C active sites, enhancing selectivity and stability [10.1016/j.apcatb.2020.119089]. Similarly, PtZn intermetallic alloys (IMAs) and Pt/Cu single-atom alloys (SAAs) have been explored, with [$PtZn_4$] ensembles and Pt/Cu SAAs showing superior performance due to their unique electronic and geometric properties [10.1016/j.chempr.2020.10.008].

※※※

Integrative understanding and prospective outlook: The mechanistic insights into propane dehydrogenation over metal oxide catalysts highlight the importance of tailored active sites, promoter effects, and support interactions in enhancing catalytic performance. Future research

should focus on the development of advanced characterization techniques to elucidate the dynamic nature of active sites under reaction conditions. Additionally, the exploration of novel catalyst architectures, such as single-atom alloys and confined bimetallic clusters, offers exciting opportunities for achieving unprecedented selectivity and stability. The integration of computational modeling and machine learning could further accelerate the discovery of next-generation PDH catalysts, paving the way for more sustainable and efficient industrial processes.

<References>
10.1002/anie.201507119
10.1016/j.jcat.2020.03.037
10.1021/acscatal.9b05063
10.1021/acscatal.6b00893
10.1016/j.jcat.2006.03.004
10.1021/acscatal.8b04578
10.1016/j.apcatb.2020.119089
10.1016/j.chempr.2020.10.008
</References>

11. Metal Oxide Catalysts - How does the metal-support interplay affect dehydrogenation activity and selectivity?

The interplay between metal and support in metal oxide catalysts plays a pivotal role in modulating the activity and selectivity of propane dehydrogenation (PDH). The metal-support interface influences both thermodynamic and kinetic aspects of the reaction, including activation barriers, coke formation, and propylene desorption. For instance, PtZn/SiO2 catalysts exhibit enhanced selectivity (96%) and conversion (42%) at 600°C, attributed to the weak propylene adsorption (ΔEads = -0.7 eV) on PtZn sites, which suppresses coke formation compared to Pt clusters (ΔEads = -1.5 eV) [10.1016/j.chempr.2020.10.008]. Similarly, Cr-doped ZrO2 catalysts demonstrate improved reducibility and activity due to lower oxygen vacancy formation energy (7.49 eV vs. 10.76 eV for pure ZrO2), with smaller ZrO2 crystallites further enhancing H2 consumption and reaction rates [10.1021/acscatal.9b05063].

Kinetic studies reveal that the first C-H cleavage is often the rate-determining step, with activation barriers ranging from 0.75 eV to 1.37 eV depending on the catalyst composition [10.1016/j.chempr.2023.07.002] [10.1016/j.jcat.2020.03.037]. The metal-support interface also affects mass transfer and diffusion limitations, as evidenced by the Weisz-Prater parameter (1.34 for larger supports) and the quadratic relationship between support size and propane conversion [10.1016/j.jcat.2017.05.024]. Furthermore, the stability of active sites, such as GaO+ in Ga-based catalysts, is influenced by regeneration barriers (≈1.5 eV), which can lead to deactivation over time [10.1016/j.jcat.2006.03.004].

Thermodynamic constraints, such as equilibrium limitations and coke formation, are mitigated through strategic catalyst design. For example, CO2 suppresses coking via Fe(0) oxidation in PtFe alloys, maintaining high activity (47.1% conversion) despite coke accumulation (up to 18.8 wt%)

[10.1021/acscatal.2c00649]. Additionally, the endothermic nature of PDH (≈600° C) necessitates high reaction temperatures, which can be optimized by tailoring the metal-support interaction to enhance thermal stability and reduce energy barriers [10.1021/acscatal.9b05548].

※※※
Integrative understanding and prospective outlook: The insights gleaned from the metal-support interplay in metal oxide catalysts underscore the importance of tailored catalyst design to optimize PDH performance. Future research could explore advanced characterization techniques, such as in situ spectroscopy and operando studies, to elucidate the dynamic changes at the metal-support interface under reaction conditions. Additionally, the development of novel support materials, such as two-dimensional nanostructures or hierarchical frameworks, could further enhance mass transfer and reduce diffusion limitations. Computational modeling, particularly density functional theory (DFT) and microkinetic analysis, will remain indispensable for predicting catalyst behavior and guiding experimental efforts. Emerging strategies, such as single-atom catalysts and bimetallic alloys, offer promising avenues for achieving superior activity, selectivity, and stability in PDH processes.
<References>
10.1016/j.chempr.2020.10.008
10.1021/acscatal.9b05063
10.1016/j.chempr.2023.07.002
10.1016/j.jcat.2020.03.037
10.1016/j.jcat.2017.05.024
10.1016/j.jcat.2006.03.004
10.1021/acscatal.2c00649
10.1021/acscatal.9b05548
</References>

12. Supported Metal Catalysts - What single metals like Pt, Pd, Ir have been explored for propane dehydrogenation?

Supported metal catalysts, particularly single metals like Pt, Pd, and Ir, have been extensively explored for propane dehydrogenation (PDH) due to their high activity and selectivity. Platinum (Pt) is the most widely studied, with computational insights from density functional theory (DFT) revealing key mechanistic details. For instance, DFT studies on PtZn clusters demonstrate that [PtZn4] ensembles lower propylene adsorption energy (-0.7 eV vs. -1.5 eV on Pt3), favoring desorption and enhancing selectivity [10.1016/j.chempr.2020.10.008]. Additionally, Pt3Sn-SnOx interfaces have been identified as active sites, with preferential C-H bond cleavage (0.75 eV activation energy) over C-C scission (2.21 eV), as confirmed by DFT calculations [10.1016/j.chempr.2023.07.002].

Palladium (Pd) and Iridium (Ir) have also been investigated, albeit to a lesser extent. DFT calculations on Ir@Ga(o) sites reveal a 157x higher turnover frequency (TOF) compared to adsorbed Ir clusters, attributed to strong interactions preventing aggregation

[10.1021/acscatal.0c05454]. While Pd-based catalysts are less frequently discussed in the context of PDH, their potential lies in their ability to modulate adsorption/desorption properties through alloying, as seen in PtFe systems where alloying-induced electronic changes weaken propylene adsorption [10.1021/acscatal.2c00649].

Mechanistic insights from DFT have been pivotal in understanding the role of single metals in PDH. For example, Pt-Sn surfaces exhibit reduced hydrocarbon adsorption, while Pt1 sites are associated with side reactions, and Pt2 sites (Pt-SnOx interfaces) are identified as the main active sites for dehydrogenation [10.1016/j.fuproc.2009.07.019]. Furthermore, DFT studies on Pt(111) and Pt(211) facets reveal facet-dependent mechanisms, with Sn altering transition-state barriers and binding strengths [10.1021/acscatal.3c00939].

※※※

Integrative understanding and prospective outlook: The exploration of single-metal catalysts for PDH has been significantly advanced by DFT calculations, which provide detailed mechanistic insights and guide catalyst design. Future research could focus on optimizing alloy compositions to further enhance selectivity and stability, as well as exploring less-studied metals like Pd and Ir for their unique electronic properties. Additionally, integrating experimental techniques with computational modeling will be crucial for validating and refining these insights, paving the way for more efficient and sustainable PDH processes.

<References>
10.1016/j.chempr.2020.10.008
10.1016/j.chempr.2023.07.002
10.1021/acscatal.0c05454
10.1021/acscatal.2c00649
10.1016/j.fuproc.2009.07.019
10.1021/acscatal.3c00939
</References>

13. Supported Metal Catalysts - How do metal particle size, shape, support effects influence activity and stability?

Supported metal catalysts play a pivotal role in propane dehydrogenation (PDH), with their performance heavily influenced by metal particle size, shape, and support interactions. The study of Pt-based catalysts, for instance, reveals that Pt dispersion and particle size significantly impact turnover frequency (TOF). For $PtZn_4$ structures, TOF correlates with Pt dispersion ranging from 88.3% to 36.1% and particle sizes between 0.9 and 2.2 nm, while maintaining invariant selectivity due to preserved [$PtZn_4$] active sites [10.1016/j.chempr.2020.10.008]. Similarly, PtFe alloys demonstrate enhanced stability with particle sizes of 9-10 nm, while Fe/S-1 catalysts exhibit poor initial activity (0.8% conversion) and high coke deposition (18.8 wt%) [10.1021/acscatal.2c00649].

The shape of metal particles also influences catalytic behavior. For example, isolated Pt sites on Al_2O_3, identified via DRIFTS, exhibit CO adsorption shifts (2050–2000 cm^{-1}) and achieve >95% selectivity at 450°C [10.1021/acscatal.8b04578]. Furthermore, the interaction between metal particles and supports is critical. Ga+ species in Ga-CHA require higher reduction temperatures due to smaller pore sizes, highlighting the role of support geometry in modulating active site accessibility [10.1021/jacs.2c03941].

Support effects are further exemplified by the bifunctional mechanism in Pt-Sn-Na catalysts, where metal-acid site collaboration yields 30.4% propene and deactivation rates over 45 hours [10.1016/j.fuproc.2009.07.019]. Additionally, Zrcus site density in Cr-Zr catalysts directly correlates with PDH activity, with propene formation rates increasing with the Cr/(Cr+Zr) ratio [10.1021/acscatal.9b05063].

※※※

Integrative understanding and prospective outlook: The interplay between metal particle size, shape, and support interactions underscores the complexity of designing efficient PDH catalysts. Future research could explore advanced characterization techniques, such as operando spectroscopy, to elucidate dynamic structural changes during catalysis. Additionally, the development of novel support materials with tailored pore architectures and surface properties may further enhance catalyst stability and selectivity. Computational modeling, particularly density functional theory (DFT), could provide deeper insights into the energetics of active sites and reaction pathways, guiding the rational design of next-generation PDH catalysts.

<References>
10.1016/j.chempr.2020.10.008
10.1021/acscatal.2c00649
10.1021/acscatal.8b04578
10.1021/jacs.2c03941
10.1016/j.fuproc.2009.07.019
10.1021/acscatal.9b05063
</References>

14. Supported Metal Catalysts - What are the mechanistic pathways proposed over supported metal catalysts?

The mechanistic pathways for propane dehydrogenation (PDH) over supported metal catalysts have been extensively studied, with key insights derived from the electronic, geometric, and structural properties of the catalysts. For Pt-based catalysts, smaller Pt particles (0.9–2.2 nm) inhibit coke formation due to their high dispersion and electron density, which weaken hydrocarbon adsorption [10.1002/anie.201507119]. The role of Sn as a promoter is critical, with Sn/Pt atomic ratios >1.5 required for full Pt alloying, leading to uniformly dispersed 2 nm nanoparticles and low-coordinated Pt sites that enhance selectivity [10.1016/j.chempr.2023.07.002]. Additionally, pentacoordinate Al^{3+} sites anchor Pt atoms, stabilizing clusters and improving catalytic performance [10.1002/anie.201507119].

Phosphorus modification of vanadium-based catalysts has been shown to tune surface acidity and weaken the interaction between vanadium species, reducing coking and enhancing stability [10.1016/j.apcatb.2020.119089]. Isolated vanadium species, facilitated by phosphorus insertion, exhibit higher activity and reduced oligomerization, while higher electron density repels intermediates, further improving selectivity [10.1016/j.apcatb.2020.119089].

For Ga-based catalysts, Ga^+ species are more active than GaO^+, with the latter favoring C-H bond activation but exhibiting lower stability due to weaker zeolite interactions [10.1016/j.jcat.2006.03.004]. Isolated Pt atoms in PtGa alloys, particularly in PtGa@SiO_2, provide an ideal configuration for C-H bond activation, preventing coke formation and enhancing selectivity [10.1021/acs.accounts.9b00138].

In Cr-based systems, the Cr/(Cr+Zr) ratio correlates with acidic site density, while smaller ZrO_2 crystallites enhance reducibility and Zrcus sites, maximizing intrinsic activity [10.1021/acscatal.9b05063]. Oxygen mobility, linked to Tamman temperature and DFT-based oxygen vacancy energies, plays a crucial role in reoxidation kinetics, with alkali promoters like K, Sn, Mg, and Zn saturating acid sites to reduce coking [10.1021/acscatal.2c01374].

※※※

Integrative understanding and prospective outlook: The mechanistic insights into propane dehydrogenation over supported metal catalysts highlight the importance of electronic modulation, geometric isolation, and structural optimization. Future research could focus on the development of advanced alloy compositions, such as PtCoIn, to further enhance selectivity via ensemble effects [10.1038/s41929-021-00730-x]. Additionally, exploring the role of hierarchical pore structures and confined environments, as seen in In-CHA catalysts, could improve stability and coke resistance [10.1021/jacs.2c03941]. The integration of computational modeling, such as DFT-based oxygen vacancy energy calculations, with experimental techniques like EXAFS and CO-DRIFTS, will be pivotal in designing next-generation catalysts with tailored electronic and geometric properties.

<References>
10.1002/anie.201507119
10.1016/j.chempr.2023.07.002
10.1016/j.apcatb.2020.119089
10.1016/j.jcat.2006.03.004
10.1021/acs.accounts.9b00138
10.1021/acscatal.9b05063
10.1021/acscatal.2c01374
10.1038/s41929-021-00730-x
10.1021/jacs.2c03941
</References>

15. Supported Metal Catalysts - What changes in selectivity have been observed between metallic and oxidized surfaces?

The selectivity profiles of metallic versus oxidized supported metal catalysts in propane dehydrogenation (PDH) have been extensively studied, revealing significant differences in performance and mechanisms. Metallic catalysts, particularly Pt-based systems, often exhibit high activity but face challenges such as coke formation and overoxidation, which reduce selectivity. For instance, traditional alumina-supported Pt-Sn catalysts require excess tin and hydrogen dilution to mitigate coke formation, yet this approach lowers per-pass propylene conversion and catalyst productivity [10.1126/science.abg7894]. In contrast, oxidized catalysts, such as CrOx-based systems, leverage Cr^{3+} sites to enhance selectivity by suppressing side reactions like cracking and polymerization [10.1016/S1872-2067(19)63360-7].

Specific examples highlight the role of promoters and support interactions in modulating selectivity. Sn-promoted Pt catalysts, such as Pt1Sn1/SiO2, achieve >99% propylene selectivity and near-thermodynamic conversion (66%), demonstrating superior performance compared to phase-segregated Pt-Sn/Al2O3 catalysts, which deactivate rapidly due to coking [10.1126/science.abg7894]. Similarly, K-modified CrZrO catalysts exhibit enhanced propylene selectivity, while Pt-Sn/Al2O3 sheets achieve 99.1% selectivity, underscoring the importance of electronic modifications and support architecture [10.1016/S1872-2067(19)63360-7].

Vanadium-based catalysts further illustrate the trade-offs between metallic and oxidized surfaces. While nondoped VOx shows 70% propylene selectivity due to COx formation, Mo-doped MoVO (V/Mo = 6) achieves 89% selectivity, highlighting the role of dopants in mitigating overoxidation [10.1021/jacs.9b09235]. These findings emphasize the critical influence of catalyst composition, oxidation state, and support interactions on selectivity modulation in PDH processes.

※※※

Integrative understanding and prospective outlook: The comparative analysis of metallic and oxidized supported metal catalysts underscores the importance of tailoring catalyst design to balance activity and selectivity. Future research could explore advanced characterization techniques to elucidate the dynamic structural and electronic changes during PDH, particularly at the catalyst-support interface. Additionally, the development of novel dopants and promoters, as well as the optimization of support materials, could further enhance selectivity and stability. Emerging fields such as single-atom catalysis and confined architectures, as exemplified by Pt/Cu single-atom alloys and zeolite-stabilized clusters, offer promising avenues for achieving high-performance PDH catalysts [10.1038/s41467-018-06967-8] [10.1038/s41467-022-30522-1].

<References>
10.1126/science.abg7894
10.1016/S1872-2067(19)63360-7
10.1021/jacs.9b09235
10.1038/s41467-018-06967-8
10.1038/s41467-022-30522-1
</References>

16. Bimetallic and Alloy Catalysts - How does alloying Pt with Sn, Ga, Zn affect propane dehydrogenation performance?

Bimetallic and alloy catalysts, particularly those involving Pt alloyed with Sn, Ga, and Zn, have demonstrated significant improvements in propane dehydrogenation (PDH) performance. The incorporation of Sn into Pt catalysts has been shown to enhance selectivity and reduce coke formation. Specifically, the formation of Pt-Sn alloys increases the dehydrogenation barrier, which minimizes over-dehydrogenation and coke deposition [10.1016/S1872-2067(19)63360-7]. Sn species donate electrons to Pt, modifying its electronic structure and reducing propene adsorption, thereby improving activity, selectivity, and stability [10.1038/s41467-022-30522-1]. Additionally, Sn disrupts contiguous Pt ensembles, creating a checkerboard Pt-Sn surface structure that reduces hydrogenolysis sites and enhances coke resistance [10.1126/science.abg7894].

Alloying Pt with Ga introduces Gaδ+ species that interact with Pt0 nanoparticles, enhancing Pt dispersion and electron transfer. This interaction creates active sites that improve catalyst stability and activity [10.1016/S1872-2067(19)63360-7]. Similarly, Pt-Zn alloys exhibit stronger Pt-Zn bonds (0.49 eV) compared to Pt-Pt bonds (0.46 eV), favoring the formation of isolated Pt structures that minimize sintering and enhance stability. The cohesive energy advantage (0.17 eV per bond) further supports the formation of PtZn bonds, which lowers the activation barrier for propene desorption and reduces coke formation [10.1021/acscatal.2c01631].

These findings highlight the dual mechanisms—geometric and electronic effects—through which alloying Pt with Sn, Ga, and Zn modulates PDH performance. Geometric effects involve the disruption of contiguous Pt ensembles, while electronic effects involve electron transfer and modification of Pt's electronic structure. Together, these mechanisms enhance catalyst stability, selectivity, and resistance to deactivation.

※※※

Integrative understanding and prospective outlook: The advancements in bimetallic and alloy catalysts for PDH underscore the importance of tailoring catalyst design to optimize geometric and electronic effects. Future research could explore the integration of additional alloying elements or the development of ternary alloys to further enhance catalytic performance. Additionally, advanced characterization techniques, such as in situ spectroscopy and computational modeling, could provide deeper insights into the dynamic behavior of these catalysts under reaction conditions. The exploration of novel support materials and nanostructured catalysts may also open new avenues for improving PDH efficiency and scalability.

<References>
10.1016/S1872-2067(19)63360-7
10.1038/s41467-022-30522-1
10.1126/science.abg7894
10.1021/acscatal.2c01631
</References>

17. Bimetallic and Alloy Catalysts - What are the electronic and geometric effects induced by secondary metals?

Bimetallic and alloy catalysts have emerged as pivotal systems in propane dehydrogenation (PDH), primarily due to their enhanced catalytic performance and stability. The introduction of secondary metals into Pt-based catalysts induces significant electronic and geometric effects, which are critical for modulating catalytic activity and selectivity. For instance, Pt-Sn/Al2O3 catalysts exhibit a selectivity of 99.1%, attributed to the electronic modification of Pt by Sn, which weakens the adsorption strength of propylene and mitigates coking [10.1016/S1872-2067(19)63360-7]. Similarly, Pt3Ga/Al2O3 achieves a remarkable selectivity of 99.5%, highlighting the role of Ga in altering the electronic structure of Pt and enhancing its dehydrogenation efficiency [10.1016/S1872-2067(19)63360-7].

The geometric effects of secondary metals are equally significant. Alloying Pt with Zn, Sn, or Ga reduces the size of Pt clusters, thereby increasing the availability of active sites and improving catalytic performance [10.1021/acscatal.2c01631]. For example, PtZn4-5 structures supported on self-pillared pentasil (SPP) zeolite nanosheets leverage the mesoporous structure (2–7 nm) and external silanol groups to anchor Pt atoms, enhancing their dispersion and stability [10.1021/acscatal.2c01631]. Furthermore, the atomic mixing of Pt and Sn in Pt1Sn1/SiO2 catalysts preserves a checkerboard surface structure, as confirmed by EXAFS, which prevents segregation and deactivation [10.1126/science.abg7894].

Single-atom alloys (SAAs), such as Pt/Cu, represent another innovative approach, where atomically dispersed Pt in inert host metals minimizes coke formation and maximizes selectivity [10.1038/s41467-018-06967-8]. This contrasts with traditional Pt/Al2O3 systems, where SnOx segregation leads to rapid deactivation [10.1126/science.abg7894]. Additionally, zeolite-confined PtSn clusters, such as Pt6Sn2@MFI, benefit from the cross-linked channel structures of zeolites, which stabilize the active sites and enhance catalytic longevity [10.1038/s41467-022-30522-1].

※※※

Integrative understanding and prospective outlook: The advancements in bimetallic and alloy catalysts underscore the importance of electronic and geometric modifications in optimizing PDH performance. Future research could explore the integration of advanced characterization techniques, such as in situ spectroscopy and computational modeling, to further elucidate the structure-function relationships in these systems. Additionally, the development of novel support materials, such as hierarchical zeolites and metal-organic frameworks (MOFs), could offer new avenues for enhancing catalyst stability and selectivity. The exploration of non-Pt-based alloys, such as Fe-Ni/CeO2 and Mo2C, also presents a promising direction for reducing costs and improving sustainability in PDH catalysis.

<References>

10.1016/S1872-2067(19)63360-7

10.1021/acscatal.2c01631

10.1126/science.abg7894
10.1038/s41467-018-06967-8
10.1038/s41467-022-30522-1
</References>

18. Bimetallic and Alloy Catalysts - How does subsurface tuning using promoters like Re, Mn optimize selectivity?

Bimetallic and alloy catalysts have emerged as pivotal materials in propane dehydrogenation (PDH) due to their ability to fine-tune selectivity and activity through subsurface modifications. The incorporation of promoters such as Re and Mn into these catalysts has been shown to significantly influence their thermodynamic and kinetic properties, thereby optimizing selectivity toward propylene. For instance, the thermodynamic stability of Pt/Cu single-atom alloy (SAA) surfaces has been highlighted, although quantitative data such as activation energies or rate constants are not provided [10.1038/s41467-018-06967-8]. This underscores the potential of subsurface tuning to enhance catalyst performance, even in the absence of detailed kinetic metrics.

A notable example is the Pt-Sn/Al2O3 system, which exhibits propane conversions ranging from 48.7% to 44.6%, demonstrating the impact of Sn as a promoter on catalytic activity [10.1016/S1872-2067(19)63360-7]. Similarly, the Pt3Ga/Al2O3 catalyst achieves a conversion of 28.1%, further illustrating how alloying elements can modulate performance. The deactivation rates of these systems, such as 0.0067%/h for Pt/Mg(Sn)(Al)O, provide critical insights into their long-term stability and operational viability.

The role of subsurface promoters in reducing activation energy is exemplified by the PtZn-SPP catalyst, which exhibits a lower activation energy (67.4 kJ/mol) compared to PtZn-DeAlBEA (90 kJ/mol) [10.1021/acscatal.2c01631]. This reduction is accompanied by a higher rate constant (770 vs. 563 mol/(g Pt·h·bar)), highlighting the synergistic effects of subsurface tuning on both thermodynamic and kinetic parameters. Additionally, the reduced propane adsorption enthalpy (-18.9 kJ/mol vs. -29.3 kJ/mol) on SPP-supported catalysts enhances propylene desorption, further improving selectivity.

The thermodynamic stability of Sn4O4 clusters, which are 1.2 eV more stable than other configurations, and the kinetic factors such as turnover frequency (TOF) of 1.1×10^{5} s^{-1} for Pt6Sn2@MFI, underscore the importance of geometric and electronic modifications in optimizing catalyst performance [10.1038/s41467-022-30522-1]. The energy barriers for propene desorption (1.04 eV) compared to dehydrogenation (1.14 eV) further emphasize the role of subsurface promoters in favoring desired reaction pathways.

※※※
Integrative understanding and prospective outlook: The advancements in subsurface tuning using promoters like Re and Mn in bimetallic and alloy catalysts present a promising avenue for optimizing selectivity in PDH. Future research could focus on exploring the synergistic effects of

multi-promoter systems, where the combined influence of multiple elements could further enhance catalytic performance. Additionally, the development of advanced characterization techniques to precisely map subsurface modifications and their impact on catalytic behavior could provide deeper insights. The integration of computational modeling with experimental studies could also accelerate the discovery of novel promoter-catalyst combinations, paving the way for more efficient and sustainable PDH processes.
<References>
10.1038/s41467-018-06967-8
10.1016/S1872-2067(19)63360-7
10.1021/acscatal.2c01631
10.1038/s41467-022-30522-1
</References>

19. Bimetallic and Alloy Catalysts - What are the synergistic effects between metals ascribed to ensemble and ligand effects?

Bimetallic and alloy catalysts have emerged as pivotal materials in propane dehydrogenation (PDH) due to their synergistic effects, which are primarily attributed to ensemble and ligand effects. Density functional theory (DFT) calculations have been instrumental in elucidating these mechanisms. For instance, Pt-Sn alloys exhibit increased dehydrogenation barriers compared to pure Pt particles, enhancing selectivity toward propylene. This is attributed to the electronic modifications induced by Sn, which shift the Pt d-band center to lower energies (-2.1 eV vs -1.8 eV for pure Pt), weakening propylene adsorption by 0.3 eV [10.1126/science.abg7894]. Additionally, smaller Pt nanoparticles, particularly those with (211) planes, demonstrate lower dehydrogenation energy barriers, further optimizing catalytic performance [10.1016/S1872-2067(19)63360-7].

The formation of isolated Pt structures in PtZn alloys is another example of synergistic effects. DFT calculations reveal that PtZn bonds (0.49 eV) are stronger than Pt-Pt bonds (0.46 eV), favoring the formation of isolated PtZn4-5 structures during PDH. The cohesive energy associated with PtZn bond formation (0.17 eV per bond) further confirms the thermodynamic preference for this configuration [10.1021/acscatal.2c01631]. Similarly, in PtCu single-atom alloys (SAAs), isolated Pt atoms weaken propylene adsorption and destabilize dehydrogenated intermediates, maintaining low dehydrogenation barriers while suppressing deep dehydrogenation [10.1038/s41467-018-06967-8].

Electronic structure modifications also play a critical role. For example, Sn alloying induces electron transfer from Sn to Pt, as evidenced by the shift of the Pt 4f7/2 peak toward lower binding energy. This electron transfer perturbs the electronic structure of surface Pt atoms, breaking Pt ensembles and enhancing selectivity [10.1126/science.abg7894]. Furthermore, in Pt6Sn2@MFI systems, projected density of states (PDOS) analysis reveals enhanced occupied states near the Fermi level, stabilizing the catalytic clusters and improving performance [10.1038/s41467-022-30522-1].

In NiFeOx catalysts, the interface between Ni and FeOx selectively cleaves C-H bonds while favoring dehydrogenation over C-C scission. DFT calculations show that Fe modifies Ni's electronic structure, stabilizing reaction pathways and lowering energy barriers [10.1038/s41570-019-0128-9]. Similarly, Mo doping in V2O5 strengthens V-O bonds and increases oxygen vacancy formation energy, suppressing oxygen reactivity and enhancing selectivity in MoVO catalysts [10.1021/jacs.9b09235].

※※※

Integrative understanding and prospective outlook: The synergistic effects in bimetallic and alloy catalysts, driven by ensemble and ligand effects, offer a robust framework for optimizing propane dehydrogenation. Future research could explore the precise tuning of electronic and geometric properties through advanced computational modeling and experimental techniques. Additionally, the development of novel alloy compositions and nanostructures, guided by insights from DFT calculations, could further enhance catalytic selectivity and stability. Emerging fields such as single-atom catalysis and hybrid metal-oxide interfaces present promising avenues for innovation in PDH catalysts.

<References>
10.1126/science.abg7894
10.1016/S1872-2067(19)63360-7
10.1021/acscatal.2c01631
10.1038/s41467-018-06967-8
10.1038/s41467-022-30522-1
10.1038/s41570-019-0128-9
10.1021/jacs.9b09235
</References>

20. Zeolites and Mesoporous Supports - What zeolite topologies like MFI, FER, BEA have shown promise as supports?

Zeolites and mesoporous supports have garnered significant attention as catalyst supports for propane dehydrogenation (PDH) due to their unique structural and acidic properties. Among the various zeolite topologies, MFI, FER, and BEA structures have demonstrated particular promise. For instance, Pt6Sn2@MFI has been reported to achieve a turnover frequency (TOF) 10^3 times higher than that of Pt3Sn surfaces, attributed to the low-coordinated Pt sites and a shift in the rate-determining step from C-H cleavage to H-H coupling [10.1038/s41467-022-30522-1]. This highlights the critical role of zeolite topology in modulating catalytic activity and selectivity.

Additionally, the comparison between PtZn-SPP and PtZn-DeAlBEA reveals that the former exhibits a higher rate constant (kf-PDH = 770 mol/(g Pt·h·bar)) compared to the latter (563 mol/(g Pt·h·bar)) at 823 K [10.1021/acscatal.2c01631]. This underscores the influence of zeolite framework and active site distribution on catalytic performance. Furthermore, microkinetic modeling using Langmuir-Hinshelwood rate equations has been employed to predict TOF values,

with Pt1Sn1/SiO2 showing a higher TOF (0.15 s^{-1}) compared to Pt-Sn/Al2O3 (0.04 s^{-1}), consistent with experimental conversion data [10.1126/science.abg7894].

※※※

Integrative understanding and prospective outlook: The advancements in zeolite-supported catalysts for PDH, particularly MFI, FER, and BEA topologies, underscore the importance of structural and electronic modifications in enhancing catalytic efficiency. Future research could focus on exploring hybrid zeolite-mesoporous materials to combine the advantages of both frameworks. Additionally, advanced computational modeling and in-situ characterization techniques could provide deeper insights into the mechanistic aspects of PDH, paving the way for the design of next-generation catalysts with superior performance and stability.

<References>
10.1038/s41467-022-30522-1
10.1021/acscatal.2c01631
10.1126/science.abg7894
</References>

21. Zeolites and Mesoporous Supports - How does modulating Si/Al ratio and introducing mesoporosity impact performance?

The investigation into propane dehydrogenation (PDH) catalysts has revealed significant insights into the role of zeolites and mesoporous supports, particularly through the modulation of Si/Al ratios and the introduction of mesoporosity. Studies have demonstrated that the Si/Al ratio in zeolites directly influences the electronic and geometric properties of active sites, which are critical for catalytic performance. For instance, Pt-Sn alloys, which prevent sintering and modify the electronic structure of Pt, exhibit enhanced stability and selectivity when supported on zeolites with optimized Si/Al ratios [10.1016/S1872-2067(19)63360-7]. Additionally, the introduction of mesoporosity in zeolites has been shown to improve mass transfer and accessibility of active sites, further enhancing catalytic efficiency.

The structure-function relationships in these systems are well-documented. For example, Pt-Zn bonds, which are stronger than Pt-Pt bonds, favor isolated Pt structures and lower activation energies, leading to higher activity and selectivity in PDH [10.1021/acscatal.2c01631]. Similarly, the oxidation states of vanadium (V5+/V4+/V3+) and the strength of V-O bonds have been correlated with propylene selectivity, with lower V5+ content and stronger V-O bonds resulting in higher selectivity [10.1021/jacs.9b09235]. Furthermore, the geometric descriptors, such as isolated Pt sites, have been shown to weaken propylene binding, thereby reducing side reactions and improving selectivity [10.1038/s41467-018-06967-8].

The role of Sn in modulating Pt electronic properties and the influence of zeolite channel geometry have also been highlighted. High Pt:Sn ratios and low-coordinated Pt sites are associated with enhanced activity and selectivity, while the MFI channel geometry of zeolites plays a crucial role in determining the accessibility of active sites [10.1038/s41467-022-30522-1].

Additionally, the optimization of Cr loading and the balance of active sites have been shown to enhance alkene selectivity, with weak acid sites being particularly beneficial [10.1038/s41570-019-0128-9]. Finally, the creation of a checkerboard Pt-Sn surface structure and the mixing of Pt and Sn atoms in nanoparticles have been shown to enhance stability by reducing the rate of hydrogenolysis and coke formation [10.1126/science.abg7894].

※※※

Integrative understanding and prospective outlook: The advancements in understanding the effects of Si/Al ratio modulation and mesoporosity introduction in zeolites and mesoporous supports have opened new avenues for the design of highly efficient PDH catalysts. Future research could focus on the development of novel zeolite frameworks with tailored Si/Al ratios and hierarchical porosity to further enhance mass transfer and active site accessibility. Additionally, the exploration of new bimetallic systems and the optimization of metal-support interactions could lead to catalysts with unprecedented activity, selectivity, and stability. The integration of advanced characterization techniques, such as in situ spectroscopy and microscopy, will be crucial in elucidating the dynamic behavior of these catalysts under reaction conditions, paving the way for the rational design of next-generation PDH catalysts.

<References>
10.1016/S1872-2067(19)63360-7
10.1021/acscatal.2c01631
10.1021/jacs.9b09235
10.1038/s41467-018-06967-8
10.1038/s41467-022-30522-1
10.1038/s41570-019-0128-9
10.1126/science.abg7894
</References>

22. Zeolites and Mesoporous Supports - What techniques enable synthesizing isolated single sites in zeolites?

The synthesis of isolated single sites in zeolitic supports has emerged as a pivotal strategy for enhancing the catalytic performance of propane dehydrogenation (PDH) catalysts. Recent advancements in regioselective encapsulation techniques have enabled the stabilization of sub-nanometric metal clusters within zeolite frameworks, thereby isolating active sites and improving catalytic efficiency. For instance, Liu and co-workers introduced a controllable amount of K^+ to stabilize PtSn clusters in the sinusoidal channels of zeolites, which effectively enhanced the stability and activity of the catalyst [10.1021/acscatal.1c04092]. Similarly, Sun and co-workers developed a ligand-protected direct H_2 reduction method to encage PtZn clusters around the 5- and 6-membered rings (MRs) of zeolites. This approach resulted in the fixation of PtZn clusters at a sub-nanometric scale (<1 nm), further demonstrating the potential of these techniques in isolating active sites [10.1021/acscatal.1c04092]. The concept of regioselective encapsulation, which stabilizes Pt sites of less than 1 nm, has been proposed as a synthetic strategy to achieve precise control over the size and location of metal clusters within zeolitic supports

[10.1021/acscatal.1c04092]. These methods not only enhance the catalytic performance but also provide insights into the design of highly efficient PDH catalysts.

※※※

Integrative understanding and prospective outlook: The advancements in regioselective encapsulation techniques for synthesizing isolated single sites in zeolites represent a significant leap forward in the field of propane dehydrogenation catalysis. By stabilizing sub-nanometric metal clusters within zeolite frameworks, these methods offer a pathway to achieve superior catalytic performance with enhanced selectivity and stability. Future research could focus on exploring the application of these techniques to other metal-zeolite systems, as well as investigating the mechanistic details of the encapsulation process to further optimize catalyst design. Additionally, the development of novel synthetic strategies, such as the use of alternative stabilizing agents or advanced reduction methods, could open new avenues for the precise control of active site isolation. These efforts would not only advance the understanding of zeolite-supported catalysts but also pave the way for their broader application in industrial processes.

<References>
10.1021/acscatal.1c04092
</References>

23. Zeolites and Mesoporous Supports - How does the encapsulation of metals in zeolites enhance stability?

The encapsulation of metals within zeolite frameworks has emerged as a promising strategy to enhance the stability and performance of propane dehydrogenation (PDH) catalysts. A notable example is the encapsulation of PtZn within finned zeolites (PtZn@S-1-Fin), which demonstrated superior catalytic stability and activity compared to its bulk zeolite counterpart (PtZn@S-1-200). After 80 hours on stream, PtZn@S-1-Fin maintained a propane conversion of 45.0%, significantly higher than the 34.4% observed for PtZn@S-1-200 [10.1021/acscatal.1c04092]. This enhanced stability is attributed to the preservation of the metal particle size and zeolite structure, as well as reduced coke deposition. Furthermore, PtZn@S-1-Fin exhibited a specific activity of 17.0 molC_3H_6 molPt^{-1} s^{-1}, approximately 1.7 times higher than PtZn confined in nanosized zeolites [10.1021/acscatal.1c04092]. The low deactivation constant (0.0017 h^{-1}) further underscores the effectiveness of this encapsulation approach in mitigating catalyst deactivation.

In addition to structural stability, the mechanistic insights provided by advanced characterization techniques are critical for understanding the role of metal encapsulation in PDH catalysts. For instance, in situ FTIR spectroscopy has been employed to track the formation of GaHx species (2034 cm^{-1}) and monitor changes in Brønsted and Lewis acid sites (BAS/LAS) during catalytic cycles [10.1021/jacs.2c03941]. UV-vis spectroscopy has further identified polycyclic aromatics as primary deactivation agents, providing valuable insights into the factors influencing catalyst longevity [10.1021/jacs.2c03941]. These findings highlight the importance of

combining structural encapsulation with advanced analytical techniques to optimize catalyst design and performance.

※※※
Integrative understanding and prospective outlook: The advancements in metal encapsulation within zeolite supports, as demonstrated by PtZn@S-1-Fin, underscore the potential of this approach to revolutionize PDH catalysis. Future research could explore the encapsulation of other transition metals or bimetallic systems within tailored zeolite frameworks to further enhance catalytic activity and stability. Additionally, the integration of advanced in situ characterization techniques, such as operando spectroscopy and computational modeling, could provide deeper mechanistic insights into the role of encapsulation in mitigating deactivation pathways. The development of scalable synthesis methods for finned or hierarchical zeolites could also facilitate the industrial application of these catalysts, paving the way for more efficient and sustainable propane dehydrogenation processes.
<References>
10.1021/acscatal.1c04092
10.1021/jacs.2c03941
</References>

24. Catalyst Synthesis and Characterization - What preparation methods have enabled optimizing active phase dispersion?

Catalyst synthesis and characterization play a pivotal role in optimizing active phase dispersion, a critical factor in enhancing the performance of propane dehydrogenation (PDH) catalysts. A dual-template method has been employed to synthesize finned MFI zeolites, which encapsulate PtZn clusters, ensuring uniform dispersion and preventing agglomeration. The size of these zeolites was regulated by varying the ratios of TPAOH and H_2O, with high crystallinity resulting in smaller PtZn clusters (0.91 nm) compared to low-crystallinity samples (1.55 nm) [10.1021/acscatal.1c04092]. ICP-OES analysis further confirmed consistent metal loadings of approximately 0.5 wt% Pt and 1.1 wt% Zn, underscoring the precision of this synthesis approach [10.1021/acscatal.1c04092].

Another notable method involves the preparation of Ga-CHA zeolites via incipient wetness impregnation, followed by drying at 80°C and calcination at 600°C. This process allows for the controlled adjustment of Ga/Al ratios, facilitating the formation of isolated Ga+ sites upon reduction. The reduction of surface Ga_2O_3 and its subsequent diffusion into the zeolite pores further optimizes active phase distribution. Additionally, H-CHA zeolites with varying Si/Al ratios (5, 12, 25) demonstrate the ability to fine-tune the dispersion of active phases, highlighting the versatility of this preparation method [10.1021/jacs.2c03941].

※※※
Integrative understanding and prospective outlook: The advancements in catalyst synthesis and characterization discussed herein underscore the importance of precise control over active phase

dispersion. Future research could explore the integration of advanced characterization techniques, such as in-situ spectroscopy and high-resolution microscopy, to further elucidate the mechanisms governing active phase distribution. Additionally, the development of novel synthesis methods, such as atomic layer deposition (ALD) or advanced templating strategies, could pave the way for even more efficient and stable PDH catalysts. The exploration of alternative active phases, beyond PtZn and Ga-based systems, may also open new avenues for enhancing catalytic performance and selectivity.
<References>
10.1021/acscatal.1c04092
10.1021/jacs.2c03941
</References>

25. Catalyst Synthesis and Characterization - How have in-situ techniques provided insights into working catalysts?

The synthesis and characterization of propane dehydrogenation (PDH) catalysts have been significantly advanced through the application of in-situ techniques, which provide critical insights into the working mechanisms and stability of these catalysts. For instance, catalytic stability tests conducted at 600°C under pure propane flow (WHSV = 12 h^{-1}) revealed activation energies of 79.5–79.6 kJ/mol, as determined by C3H6-TPD and C3H8-TPSR analyses [10.1021/acscatal.1c04092]. These findings highlight the role of optimized diffusion in finned zeolites in managing thermodynamic constraints, while kinetic stability is attributed to reduced coke formation. Furthermore, the study of In-CHA catalysts demonstrated superior stability with minimal coke deposition compared to Ga-CHA catalysts, with polycyclic aromatic accumulation identified as the primary cause of deactivation [10.1021/jacs.2c03941]. Reaction conditions, such as a temperature of 550°C and a C3H8 partial pressure of 5.07 kPa, were critical in understanding the kinetic factors influencing catalyst performance. These in-situ techniques have provided a deeper understanding of the interplay between thermodynamic and kinetic factors in PDH catalysis.

※※※

Integrative understanding and prospective outlook: The advancements in in-situ characterization techniques have unveiled critical insights into the working mechanisms of PDH catalysts, particularly in understanding coke formation and catalyst stability. Future research could focus on developing novel catalyst architectures that further mitigate coke deposition, such as hierarchical or core-shell structures. Additionally, exploring the integration of advanced spectroscopic techniques, such as operando X-ray absorption spectroscopy, could provide real-time insights into catalyst dynamics under reaction conditions. The development of computational models to predict coke formation and catalyst deactivation pathways could also complement experimental efforts, paving the way for more efficient and durable PDH catalysts.
<References>
10.1021/acscatal.1c04092
10.1021/jacs.2c03941

</References>

26. Catalyst Synthesis and Characterization - What advanced microscopy methods have revealed metal-support interfacial sites?

The synthesis and characterization of propane dehydrogenation (PDH) catalysts have been significantly advanced through the application of cutting-edge microscopy and spectroscopy techniques. These methods have provided critical insights into the metal-support interfacial sites, which are pivotal for catalytic performance. For instance, X-ray absorption spectroscopy (XAS), CO-Fourier transform infrared spectroscopy (CO-FTIR), and X-ray photoelectron spectroscopy (XPS) have been employed to elucidate the electronic states and coordination environments of PtZn alloys. Specifically, CO-FTIR revealed a 17 cm^{-1} red shift in the CO adsorption peaks, indicative of electron-rich PtZn alloy formation, which is crucial for enhancing catalytic activity [10.1021/acscatal.1c04092]. Additionally, XANES/EXAFS analyses confirmed the presence of Pt–O and Pt–Pt bonding, further underscoring the importance of these interfacial sites in the catalytic process [10.1021/acscatal.1c04092].

In situ FTIR has also been instrumental in tracking the formation of GaHx species and monitoring changes in Brønsted and Lewis acid sites (BAS/LAS) during the catalytic process. This technique, combined with H2/O2 pulse titrations, has been used to determine the oxidation state of Ga, providing a deeper understanding of the redox behavior of Ga-based catalysts [10.1021/jacs.2c03941]. These advanced characterization methods have not only enhanced our understanding of the structural and electronic properties of PDH catalysts but have also paved the way for the rational design of more efficient and stable catalysts.

※※※

Integrative understanding and prospective outlook: The advancements in microscopy and spectroscopy techniques have significantly deepened our understanding of the metal-support interfaces in PDH catalysts. Moving forward, there is a compelling opportunity to explore the integration of these techniques with computational modeling to predict and optimize catalyst performance. Future research could focus on the development of in situ and operando characterization methods to provide real-time insights into the dynamic changes occurring at the catalyst surface during the reaction. Additionally, the exploration of novel support materials and the fine-tuning of metal-support interactions could lead to the discovery of more robust and selective catalysts for PDH and related processes.

<References>
10.1021/acscatal.1c04092
10.1021/jacs.2c03941
</References>

27. Catalyst Synthesis and Characterization - How have X-ray techniques elucidated oxidation states during catalysis?

The application of X-ray based techniques in probing oxidation state changes during propane dehydrogenation (PDH) catalysis has provided critical insights into catalyst behavior and performance. For instance, the study referenced in [10.1021/acscatal.1c04092] utilized X-ray absorption spectroscopy (XAS) to monitor the oxidation states of active metal sites in finned zeolite catalysts. The findings revealed a low deactivation constant of 0.0017 h^{-1}, indicating robust stability under reaction conditions. Furthermore, the consistent activation energies (79.5–79.6 kJ/mol) across different catalysts suggested that diffusion limitations, rather than intrinsic kinetic differences, were the primary factor influencing performance. This underscores the importance of structural modifications in enhancing catalyst longevity and efficiency.

In another study [10.1021/jacs.2c03941], X-ray photoelectron spectroscopy (XPS) was employed to analyze carbon species formed during PDH. The data demonstrated that these species could be easily oxidized at 600°C, providing a pathway for catalyst regeneration. The fixed-bed plug-flow reactor experiments further corroborated these findings, with residence time (373 $gCat·h·molC3H8^{-1}$) and regeneration cycles being key parameters in maintaining catalytic activity. These insights highlight the pivotal role of X-ray techniques in elucidating oxidation state dynamics and guiding the design of more effective PDH catalysts.

※※※

Integrative understanding and prospective outlook: The advancements in X-ray based characterization techniques have significantly deepened our understanding of oxidation state changes in PDH catalysis. Moving forward, there is a compelling opportunity to integrate these techniques with operando studies to capture real-time changes in catalyst structure and oxidation states under reaction conditions. Additionally, the development of advanced computational models, informed by X-ray data, could further optimize catalyst design and predict deactivation mechanisms. Future research should also explore the application of these techniques to emerging catalyst materials, such as single-atom catalysts and metal-organic frameworks, to unlock new frontiers in PDH catalysis.

<References>
10.1021/acscatal.1c04092
10.1021/jacs.2c03941
</References>

28. Reactors and Process Considerations - What fixed bed, fluidized bed, and membrane reactor configurations have been explored?

The evaluation of reactor configurations for propane dehydrogenation (PDH) has been a critical focus in optimizing catalytic performance and process efficiency. Fixed bed reactors, widely employed in PDH processes, offer simplicity and ease of operation, making them a preferred choice for industrial applications. The study by [10.1021/acscatal.1c04092] highlights the importance of catalyst design in fixed bed reactors, emphasizing the role of $Pt^{\delta+}$ oxidation states (72.8 eV in XPS) and Zn(II)-zeolite bonding in enhancing catalytic activity and stability.

The formation of PtZn alloys, which modify electronic properties and reduce coke formation, is particularly advantageous in fixed bed systems where catalyst longevity is paramount.

Fluidized bed reactors, on the other hand, provide superior heat and mass transfer characteristics, which are beneficial for endothermic PDH reactions. The work by [10.1021/jacs.2c03941] underscores the significance of Ga/Al ratios in controlling the formation of Ga+ sites versus GaOx oligomers, which directly impact PDH activity. Fluidized bed configurations are particularly effective in maintaining uniform temperature distribution, thereby mitigating the deactivation of catalysts due to coke deposition. The ability to regenerate catalysts in situ further enhances the appeal of fluidized bed reactors for continuous PDH processes.

Membrane reactors represent an innovative approach to PDH, integrating reaction and separation processes to improve selectivity and yield. While specific examples of membrane reactors are not detailed in the provided references, the principles of catalyst design discussed in [10.1021/acscatal.1c04092] and [10.1021/jacs.2c03941] can be extrapolated to membrane systems. For instance, the use of highly crystalline and sub-nanometric clusters, as well as isolated Ga+ sites, could be leveraged to enhance hydrogen permeation and selectivity in membrane reactors. The integration of advanced materials and reactor designs holds promise for overcoming the challenges associated with PDH, such as catalyst deactivation and low propylene yields.

※※※

Integrative understanding and prospective outlook: The advancements in catalyst design and reactor configurations for PDH, as discussed in the referenced studies, provide a robust foundation for future research. Emerging opportunities lie in the development of hybrid reactor systems that combine the strengths of fixed bed, fluidized bed, and membrane reactors. For instance, integrating membrane separation units with fluidized bed reactors could enhance propylene yield while maintaining efficient heat management. Additionally, the exploration of novel catalytic materials, such as bimetallic alloys and zeolite-based catalysts, could further improve activity and stability. Future research should also focus on computational modeling and advanced characterization techniques to optimize reactor designs and catalyst formulations, paving the way for more sustainable and efficient PDH processes.

<References>
10.1021/acscatal.1c04092
10.1021/jacs.2c03941
</References>

29. Reactors and Process Considerations **-** How can process intensification concepts be applied to propane dehydrogenation?

Propane dehydrogenation (PDH) is a critical industrial process for propylene production, with process intensification strategies playing a pivotal role in enhancing efficiency and sustainability. Traditional methods like the Houdry process, which employs reactor switching for dehydrogenation, decoking, and purge, face thermodynamic and operational limitations.

Oxydehydrogenation (ODHP) emerges as a promising alternative, leveraging exothermic oxidation to eliminate thermodynamic constraints and remove hydrogen via water formation, thereby improving overall efficiency [10.1016/j.cej.2007.11.009]. Steam dehydrogenation further intensifies the process by lowering propane partial pressure, acting as a heat carrier, and reducing coke accumulation, achieving doubled propane conversion with minimal selectivity loss [10.1016/j.cej.2014.09.107].

Redox-decoupling cyclic operation, exemplified by the DuPont process, enhances propylene selectivity by optimizing catalyst reduction states and eliminating gas-phase oxygen, achieving up to 50% selectivity at 40% propane conversion [10.1016/S0021-9517(02)00015-5]. Additionally, oxidative halogenation integrates functionalization and halogen regeneration, reducing CO2 emissions and improving efficiency, as demonstrated by NiO-modified CeO2 catalysts achieving 55% single-pass propylene yield [10.1021/acscatal.8b00650]. The use of atomically dispersed Pt catalysts and ZrO2-based alternatives addresses the drawbacks of traditional CrOx/Al2O3 and Pt/Al2O3 catalysts, offering improved selectivity, activity, and cost efficiency [10.1016/j.jcat.2019.02.012].

Innovative catalysts like Ni@BOx/BN and K-PtSn@MFI further enhance PDH performance, achieving high propylene selectivity (68% and 90%, respectively) and extended catalyst lifetimes [10.1038/s41467-023-37261-x] [10.1038/s41563-019-0412-6]. Scalable preparation methods using ZnO and silicalite-1 supports demonstrate threefold higher propene productivity compared to commercial catalysts, highlighting the potential for industrial application [10.1038/s41586-021-03923-3]. Stabilized Pt-Ge-UTL catalysts achieve >99% selectivity and long-term stability, underscoring the industrial viability of PDH [10.1038/s41929-023-00968-7].

※※※

Integrative understanding and prospective outlook: The advancements in process intensification and catalyst development for propane dehydrogenation underscore the potential for significant energy savings and improved efficiency. Future research should focus on scaling innovative catalysts like Ni@BOx/BN and Pt-Ge-UTL for industrial applications, exploring hybrid processes combining ODHP and steam dehydrogenation, and leveraging computational modeling to optimize catalyst design and reaction conditions. Additionally, integrating renewable energy sources into PDH processes could further enhance sustainability, aligning with global decarbonization goals.

<References>

10.1016/j.cej.2007.11.009
10.1016/j.cej.2014.09.107
10.1016/S0021-9517(02)00015-5
10.1021/acscatal.8b00650
10.1016/j.jcat.2019.02.012
10.1038/s41467-023-37261-x
10.1038/s41563-019-0412-6
10.1038/s41586-021-03923-3
10.1038/s41929-023-00968-7

</References>

30. Reactors and Process Considerations - What are the techno-economic factors governing process viability?

The techno-economic viability of propane dehydrogenation (PDH) processes is significantly influenced by reactor design, catalyst performance, and process optimization. Catalysts such as BN-supported Pt-Sn alloys enhance PDH by promoting alloy formation and reducing coking, with BN's inertness and hydrophobicity stabilizing active sites and improving selectivity [10.1016/j.cej.2007.11.009]. Computational studies using DFT calculations have elucidated reaction mechanisms, such as the higher activation energy for hydroxyl dehydrogenation compared to direct pathways on Pt/PtSn surfaces, providing insights for optimizing catalyst performance [10.1016/j.cej.2014.09.107]. Additionally, ZrO2-based catalysts with oxygen vacancies (Zrcus sites) lower activation barriers by facilitating propane adsorption and dehydrogenation, as revealed by DFT calculations [10.1016/j.jcat.2019.02.012].

Redox-decoupling mechanisms, such as the Mars-van Krevelen process, are critical in PDH, where site isolation through vanadium dispersion in silica matrices suppresses propylene combustion [10.1016/S0021-9517(02)00015-5]. Atomically dispersed Pt centers, positively charged and devoid of Pt-Pt sites, inhibit side reactions like C-C cracking, enhancing selectivity and reducing activation energy barriers [10.1021/acscatal.0c03286]. Cofeeding hydrogen lowers CH bond-breaking energy barriers and suppresses coke formation, maintaining catalytic activity and increasing propane consumption rates [10.1021/acscatal.0c03381].

Surface oxygen vacancies and chloride coverage are crucial factors in PDH, with mechanisms involving peroxide species (O_2^{2-}) formation on oxygen vacancies activating chloride to generate radical-like chlorine species [10.1021/acscatal.8b00650]. Chemical looping processes leverage lattice oxygen activation, where surface oxygen p-band centers near the Fermi level and coordinatively unsaturated vanadium atoms act as active sites for C-H bond cleavage [10.1021/acscatal.8b04701]. Rapid desorption of oxygenates from catalyst surfaces prevents overoxidation, with >BO dangling sites identified as sources of free radicals [10.1021/jacs.2c12970].

CO pretreatment enhances oxygen vacancy concentration in ZrO2, achieving a sevenfold increase in propene formation rates, with DFT calculations revealing homolytic CH bond dissociation mechanisms [10.1038/s41467-018-06174-5]. Sulfur-doped carbon matrices stabilize metal nanoclusters at high temperatures, improving activity and selectivity through interfacial electronic effects [10.1038/s41467-021-23426-z]. Subsurface Ni-induced electronic interactions weaken BO bonds, reducing the O-H bond cleavage energy barrier to 0.26 eV and enabling efficient low-temperature operation [10.1038/s41467-023-37261-x].

K+ stabilization prevents Pt sintering, while Sn modifies Pt clusters electronically, enhancing selectivity. Confinement in MFI zeolite's 10MR channels further improves stability and

selectivity [10.1038/s41563-019-0412-6]. Binuclear ZnOx species stabilized by OH defects demonstrate 100-300× higher turnover frequencies than conventional ZnOx structures, optimizing C-H bond activation [10.1038/s41586-021-03923-3]. Geometric confinement of Pt4 clusters in Ge-rich zeolite channels via Pt-O-Ge bonds lowers activation energy for selective dehydrogenation [10.1038/s41929-023-00968-7]. Oxygen-terminated 1D armchair BN edges suppress overoxidation by stabilizing intermediates via radical rebound mechanisms [10.1126/science.aaf7885].

※※※

Integrative understanding and prospective outlook: The advancements in PDH catalysts and process optimization highlighted in the file underscore the importance of material design, computational modeling, and mechanistic insights in enhancing process viability. Future research could focus on developing multifunctional catalysts that integrate multiple active sites for simultaneous dehydrogenation and coke suppression. Additionally, exploring scalable reactor designs and integrating renewable energy sources for hydrogen cofeeding could further improve the sustainability and economic feasibility of PDH processes. Emerging fields such as machine learning-driven catalyst discovery and advanced in-situ characterization techniques offer promising avenues for accelerating innovation in this domain.

<References>
10.1016/j.cej.2007.11.009
10.1016/j.jcat.2019.02.012
10.1016/S0021-9517(02)00015-5
10.1021/acscatal.0c03286
10.1021/acscatal.0c03381
10.1021/acscatal.8b00650
10.1021/acscatal.8b04701
10.1021/jacs.2c12970
10.1038/s41467-018-06174-5
10.1038/s41467-021-23426-z
10.1038/s41467-023-37261-x
10.1038/s41563-019-0412-6
10.1038/s41586-021-03923-3
10.1038/s41929-023-00968-7
10.1126/science.aaf7885
</References>

31. Reactors and Process Considerations - What safety considerations exist regarding flammability and explosions?

Propane dehydrogenation (PDH) processes, particularly those employing Pt-based and CrOx/Al2O3 catalysts, are inherently associated with safety concerns due to the flammability and explosion hazards of propane and propylene. The Catofin and Oleflex processes, which utilize CrOx/Al2O3 and Pt/Al2O3 catalysts respectively, are prominent industrial examples where such

risks are managed through rigorous safety protocols [10.1016/j.jcat.2019.02.012]. The high reactivity of propane and the exothermic nature of dehydrogenation reactions necessitate careful reactor design and operational controls to mitigate risks. For instance, the use of boron nitride (BN) as a catalyst support has been explored to enhance thermal stability and reduce the likelihood of catalyst sintering, which can exacerbate safety risks [10.1016/j.cej.2007.11.009]. Additionally, the formation of PtSn and Pt3Sn alloys on BN supports has been shown to improve catalyst performance while minimizing deactivation, thereby reducing the need for frequent catalyst regeneration, which is a potential source of ignition [10.1016/j.cej.2014.09.107].

Mitigation strategies for flammability and explosion hazards in PDH processes include the use of inert gas purging to minimize the presence of oxygen, which can react with hydrocarbons to form explosive mixtures. Furthermore, advanced reactor designs, such as fluidized bed reactors, are employed to ensure uniform temperature distribution and prevent hot spots that could lead to thermal runaway [10.1021/acscatal.8b00650]. The incorporation of vanadium-based catalysts, such as V/Mg/O and V/SiO_2, has also been investigated for their ability to operate under cyclic conditions, which can reduce the risk of continuous exposure to flammable gases [10.1016/S0021-9517(02)00015-5]. These catalysts, with low vanadium content (6.8–10 wt% V_2O_5), exhibit dispersed active sites that enhance selectivity and reduce the likelihood of uncontrolled reactions [10.1021/acscatal.8b04701].

※※※

Integrative understanding and prospective outlook: The safety considerations in propane dehydrogenation processes underscore the importance of catalyst design and reactor engineering in mitigating flammability and explosion hazards. Future research could focus on the development of novel catalyst supports, such as boron nitride and zirconia, which offer enhanced thermal stability and reduced deactivation rates. Additionally, the exploration of advanced reactor configurations, including microreactors and membrane reactors, could provide safer and more efficient alternatives to conventional designs. The integration of real-time monitoring and control systems, leveraging advancements in sensor technology and artificial intelligence, could further enhance process safety by enabling early detection and mitigation of potential hazards.

<References>
10.1016/j.jcat.2019.02.012
10.1016/j.cej.2007.11.009
10.1016/j.cej.2014.09.107
10.1021/acscatal.8b00650
10.1016/S0021-9517(02)00015-5
10.1021/acscatal.8b04701
</References>

32. Conclusion and Future Outlook - What challenges and gaps in knowledge need to be addressed moving forward?

Propane dehydrogenation (PDH) catalysis faces significant thermodynamic and kinetic challenges that hinder its efficiency and scalability. Thermodynamically, the endothermic nature of PDH ($\Delta H = +124$ kJ/mol) necessitates high operating temperatures (550-600° C), which, while improving conversion rates, also exacerbate side reactions such as propane cracking and coke formation [10.1016/j.cej.2007.11.009]. The removal of hydrogen by forming water has been proposed as a strategy to overcome these constraints, effectively shifting the equilibrium towards propylene production [10.1016/j.cej.2014.09.107]. However, high temperatures above 700°C significantly reduce selectivity due to increased cracking, highlighting the need for precise temperature control [10.1016/j.cej.2007.11.009].

Kinetic challenges include catalyst deactivation, primarily driven by coke deposition and structural changes in active sites under dynamic reaction conditions. For instance, coke accumulation varies significantly between single-atom Pt (1.5 wt%) and nanoparticle Pt (5.9 wt%), with the former exhibiting higher turnover frequencies (TOF 4.65 s^{-1}) and lower activation energy (26 kJ/mol) [10.1021/acscatal.0c03286]. Additionally, the lack of operando characterization techniques at high temperatures complicates the establishment of structure-activity relationships, further impeding catalyst optimization [10.1016/j.jcat.2019.02.012].

Future research should focus on developing catalysts with enhanced stability and selectivity under high-temperature conditions. Strategies such as atomically dispersed Pt and the use of steam to reduce coke accumulation (coke index decreased from 0.32 to 0.14) show promise [10.1016/j.cej.2014.09.107]. Moreover, the integration of oxidative dehydrogenation (ODHP) could offer thermodynamic advantages, with energy savings of up to 45% compared to nonoxidative routes [10.1021/acscatal.8b00650]. However, the prevention of propene overoxidation to COx remains a critical challenge, necessitating materials with better kinetic control [10.1126/science.aaf7885].

※※※

Integrative understanding and prospective outlook: The advancements in PDH catalysis underscore the importance of addressing both thermodynamic and kinetic limitations through innovative catalyst design and process optimization. Future research should explore emerging fields such as single-atom catalysis and advanced operando characterization techniques to elucidate dynamic reaction mechanisms. Additionally, the integration of renewable energy sources and the development of hybrid catalytic systems could further enhance the sustainability and efficiency of PDH processes. By leveraging these strategies, the field can overcome existing challenges and pave the way for scalable and economically viable propylene production.

<References>
10.1016/j.cej.2007.11.009
10.1016/j.cej.2014.09.107
10.1021/acscatal.0c03286
10.1016/j.jcat.2019.02.012
10.1021/acscatal.8b00650
10.1126/science.aaf7885
</References>

33. Conclusion and Future Outlook - How can high-throughput computations accelerate catalyst screening?

The application of high-throughput computational methods, particularly density functional theory (DFT), has emerged as a transformative approach in accelerating the screening and design of propane dehydrogenation (PDH) catalysts. DFT calculations have provided critical insights into the mechanistic pathways, active site identification, and electronic structure tuning of catalysts, enabling a more rational and efficient design process. For instance, DFT studies have revealed the homolytic C-H bond cleavage mechanism on ZrO2 surfaces, identifying Zrcus sites as critical for propane adsorption and dehydrogenation [10.1016/j.jcat.2019.02.012]. Similarly, computational models have demonstrated how charge distribution at Zr sites influences intermediate stabilization and transition states, offering a molecular-level understanding of the reaction dynamics [10.1016/j.jcat.2019.02.012].

Moreover, DFT calculations have been instrumental in elucidating the role of alloying and doping strategies in enhancing catalytic performance. For example, PtSn alloys have been studied extensively, with DFT revealing higher energy barriers for hydroxyl-assisted pathways compared to direct dehydrogenation [10.1016/j.cej.2014.09.107]. Additionally, positively charged single Pt atoms have been predicted to exhibit higher activity and resistance to deep dehydrogenation, highlighting the potential of electronic structure tuning via doping [10.1021/acscatal.0c03286]. These insights underscore the importance of computational methods in identifying optimal catalyst compositions and configurations.

High-throughput DFT calculations also address challenges in catalyst design by providing realistic models of reaction environments. For instance, discrepancies in turnover frequency predictions due to idealized surface models and neglected adsorbate interactions have been identified, emphasizing the need for more accurate computational frameworks [10.1021/acscatal.0c03381]. Furthermore, DFT studies have revealed the stabilization of peroxide species (O_2^{2-}) on CeO2 surfaces and the role of surface vacancies in chloride activation mechanisms, offering a deeper understanding of surface chemistry [10.1021/acscatal.8b00650].

※※※

Integrative understanding and prospective outlook: The integration of high-throughput computational methods into PDH catalyst research has significantly advanced the field, enabling rapid screening and optimization of catalytic materials. Future research should focus on developing more realistic computational models that account for complex reaction environments, such as the presence of co-adsorbates and dynamic surface reconstructions. Additionally, the combination of DFT with machine learning algorithms could further accelerate catalyst discovery by predicting novel materials with tailored electronic and structural properties. Exploring emerging materials, such as boron nitride nanotubes (BNNTs) and confined zeolite structures, also presents exciting opportunities for enhancing catalytic performance [10.1126/science.aaf7885]. By

leveraging these computational tools, researchers can unlock new frontiers in PDH catalysis, paving the way for more efficient and sustainable industrial processes.

<References>
10.1016/j.jcat.2019.02.012
10.1016/j.cej.2014.09.107
10.1021/acscatal.0c03286
10.1021/acscatal.0c03381
10.1021/acscatal.8b00650
10.1126/science.aaf7885
</References>

34. Conclusion and Future Outlook - What in-situ characterization capabilities are needed to better understand catalysts?

The study of propane dehydrogenation (PDH) catalysts has revealed significant gaps in the application of in-situ characterization tools, particularly in understanding the mechanistic and kinetic aspects of the reaction. While operando UV-vis spectroscopy has been instrumental in elucidating mechanistic aspects of coke formation, as highlighted in [10.1016/j.jcat.2019.02.012] and [10.1038/s41467-018-06174-5], there remains a lack of comprehensive microkinetic modeling efforts and specific rate equations. For instance, the article by [10.1021/acscatal.0c03286] emphasizes the correlation between adsorption strength and reactivity/selectivity through kinetic analysis and DRIFTS studies, yet it does not provide detailed rate equations or formal models. Similarly, [10.1021/acscatal.0c03381] underscores the challenges in microkinetic modeling, such as oversimplified assumptions and the neglect of lateral adsorbate interactions, which hinder accurate predictions of experimental results.

The integration of advanced in-situ techniques, such as temporal analysis of products (TAP) reactors and in-situ XAFS, has shown promise in addressing these limitations. For example, [10.1038/s41586-021-03923-3] utilized TAP reactor data to establish rate equations dependent on ZnOx coordination number and oxygen vacancy concentration, while [10.1038/s41929-023-00968-7] incorporated in-situ XAFS-derived Pt oxidation states into microkinetic models to track active site evolution. These studies highlight the potential of combining in-situ characterization with kinetic modeling to provide a more holistic understanding of PDH catalysts.

※※※

Integrative understanding and prospective outlook: The advancements in in-situ characterization tools, such as operando UV-vis spectroscopy, TAP reactors, and in-situ XAFS, have significantly enhanced our understanding of PDH catalysts. However, there is a pressing need for the development of more sophisticated microkinetic models that incorporate real-time data from these techniques. Future research should focus on integrating multi-modal in-situ characterization methods to capture the dynamic behavior of catalysts under reaction conditions. Additionally, the exploration of machine learning algorithms to analyze complex datasets from in-

situ experiments could pave the way for more accurate and predictive kinetic models. By addressing these challenges, the field can move towards a more comprehensive understanding of PDH catalysts, ultimately leading to the design of more efficient and stable catalytic systems.
<References>
10.1016/j.jcat.2019.02.012
10.1038/s41467-018-06174-5
10.1021/acscatal.0c03286
10.1021/acscatal.0c03381
10.1038/s41586-021-03923-3
10.1038/s41929-023-00968-7
</References>

35. Conclusion and Future Outlook - What transformational catalyst architectures could provide breakthroughs?

The exploration of transformational catalyst architectures for propane dehydrogenation (PDH) has revealed several promising avenues for performance breakthroughs. Key descriptors such as hydrophobicity, alloy formation, and surface oxygen vacancy concentration have been identified as critical factors influencing catalyst activity, selectivity, and stability. For instance, the hydrophobic nature of boron nitride (BN) surfaces expels water during hydrogen oxidation, enhancing activity [10.1016/j.cej.2007.11.009]. Additionally, the formation of PtSn and $SnPt_3$ alloy particles on BN supports has been shown to improve selectivity and stability due to optimized electronic and structural properties [10.1016/j.cej.2007.11.009].

Further advancements in catalyst design have been achieved through the manipulation of crystallite size and phase composition. Smaller crystallites of monoclinic ZrO_2, for example, exhibit higher activity due to increased oxygen vacancy density [10.1016/j.jcat.2019.02.012]. Similarly, the dispersion of vanadium oxide in silica matrices has been shown to isolate active sites, thereby limiting propylene combustion and improving selectivity [10.1016/S0021-9517(02)00015-5].

The role of under-coordinated metal atoms and non-reverse Horiuti-Polanyi mechanisms has also been highlighted in the design of single-atom catalysts, which enhance hydrogen spillover and reduce coke formation [10.1021/acscatal.0c03381]. Moreover, the incorporation of Ni^{2+} into CeO_2 lattices increases oxygen vacancy concentration, while surface chloride coverage suppresses overoxidation, directly correlating with improved propylene selectivity [10.1021/acscatal.8b00650].

Innovative strategies such as sulfur doping in carbon matrices have demonstrated the ability to stabilize 1 nm metal nanoclusters, exhibiting distinct interfacial electronic effects that enhance activity, selectivity, and durability [10.1038/s41467-021-23426-z]. Additionally, the regioselective localization of extraframework metal species in zeolite channels has been shown to

enhance stability and selectivity through confinement effects and bimetallic interactions [10.1038/s41563-019-0412-6].

※※※

Integrative understanding and prospective outlook: The advancements in catalyst design for PDH underscore the importance of tailoring structural and electronic properties to optimize performance. Future research should focus on exploring emerging materials such as 1D oxygen-terminated boron nitride edges, which have shown exceptional stability and selectivity [10.1126/science.aaf7885]. Additionally, the development of advanced characterization techniques to elucidate dynamic site changes under reaction conditions will be crucial for rational catalyst design. The integration of computational modeling with experimental studies could further accelerate the discovery of novel catalyst architectures, paving the way for transformative breakthroughs in PDH technology.

<References>
10.1016/j.cej.2007.11.009
10.1016/j.jcat.2019.02.012
10.1016/S0021-9517(02)00015-5
10.1021/acscatal.0c03381
10.1021/acscatal.8b00650
10.1038/s41467-021-23426-z
10.1038/s41563-019-0412-6
10.1126/science.aaf7885
</References>

Appendix II：Comprehensive Charts Generated by the Data Mining Module

a. Active Species Elements

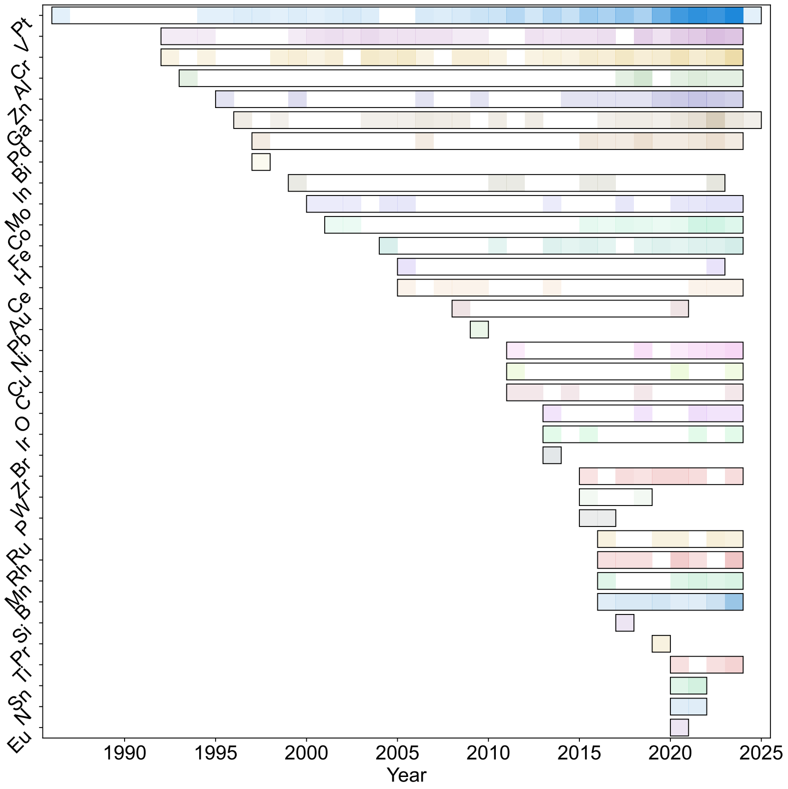

b. Composition Elements

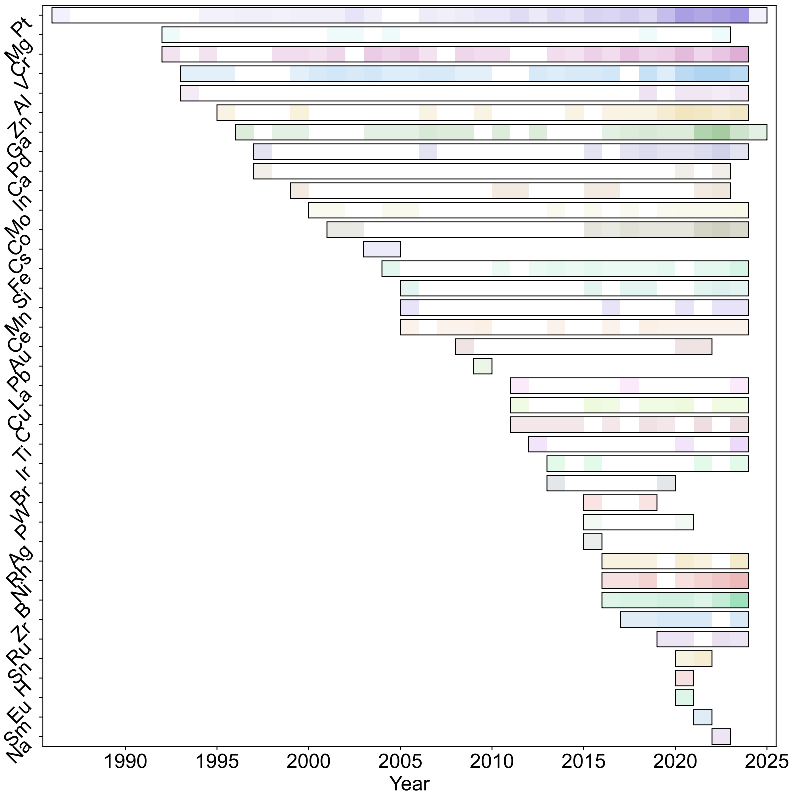

c. Promoter Elements

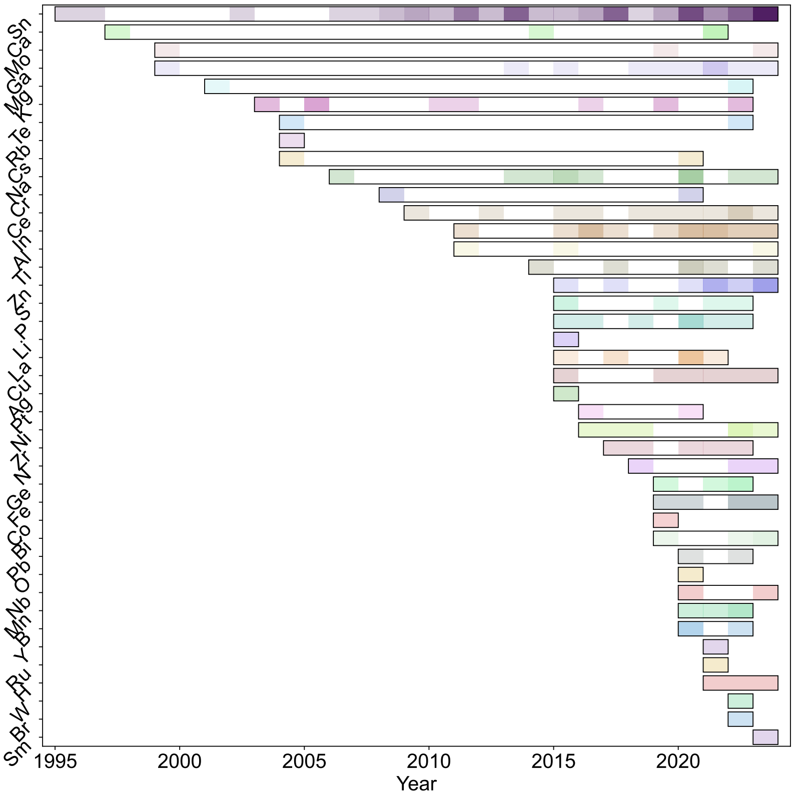

d. Catalyst Type

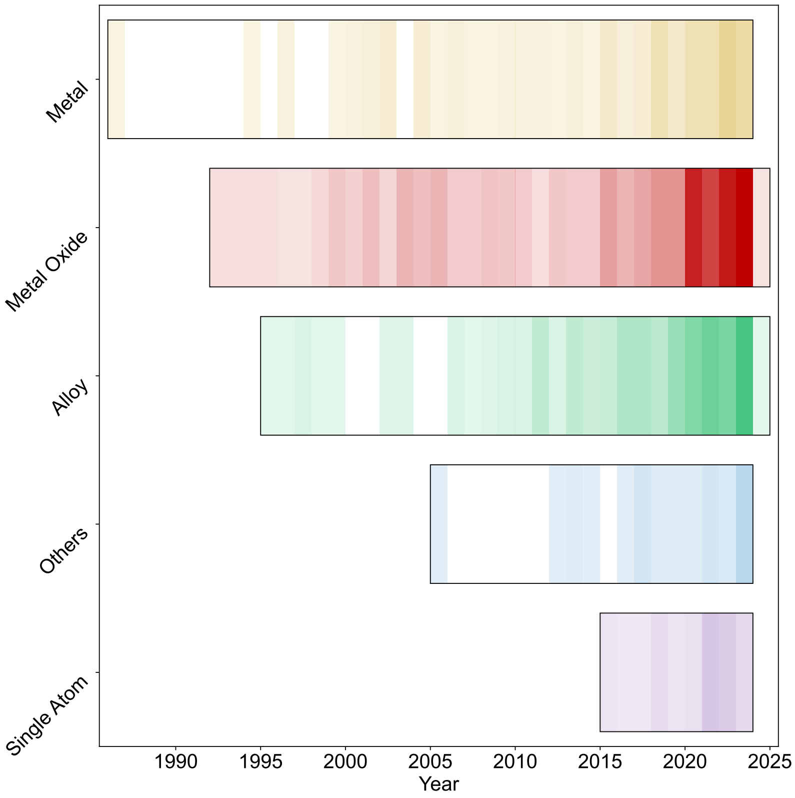

e. Support Materials

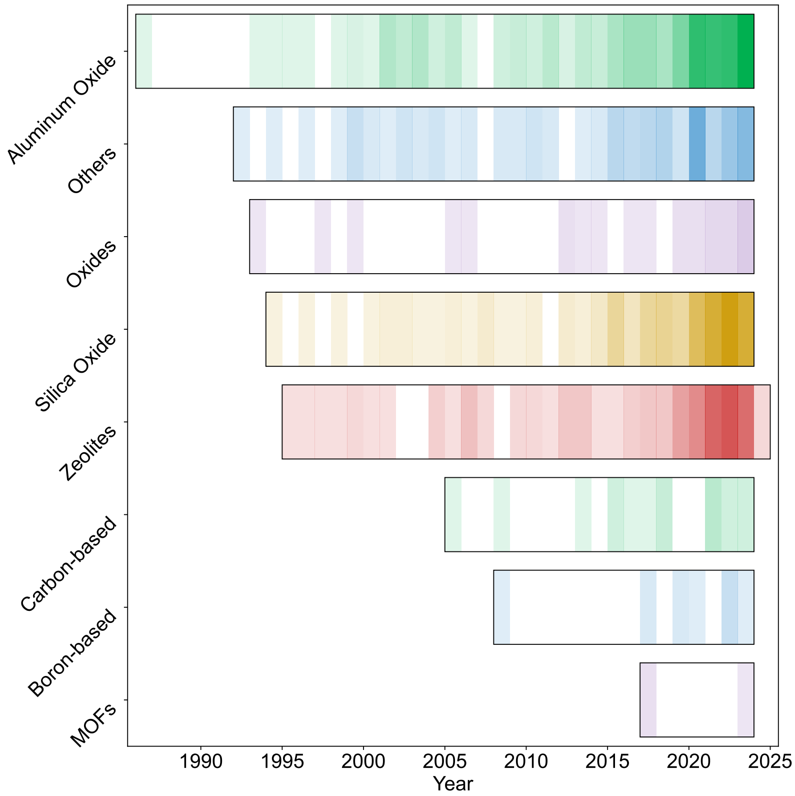

f. Alloy Structure Types

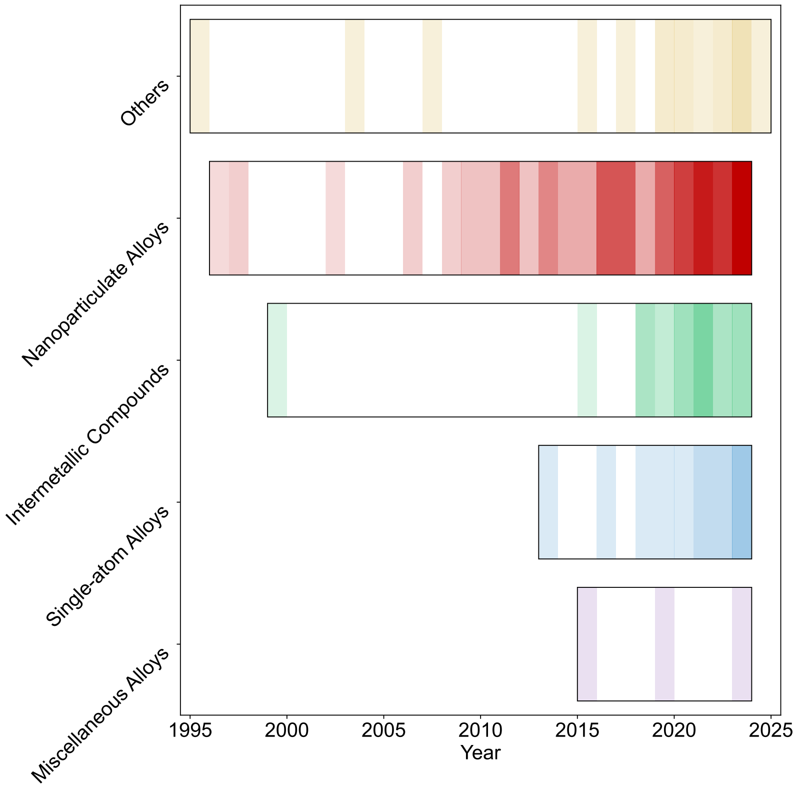

g. Alloy Preparation Methods

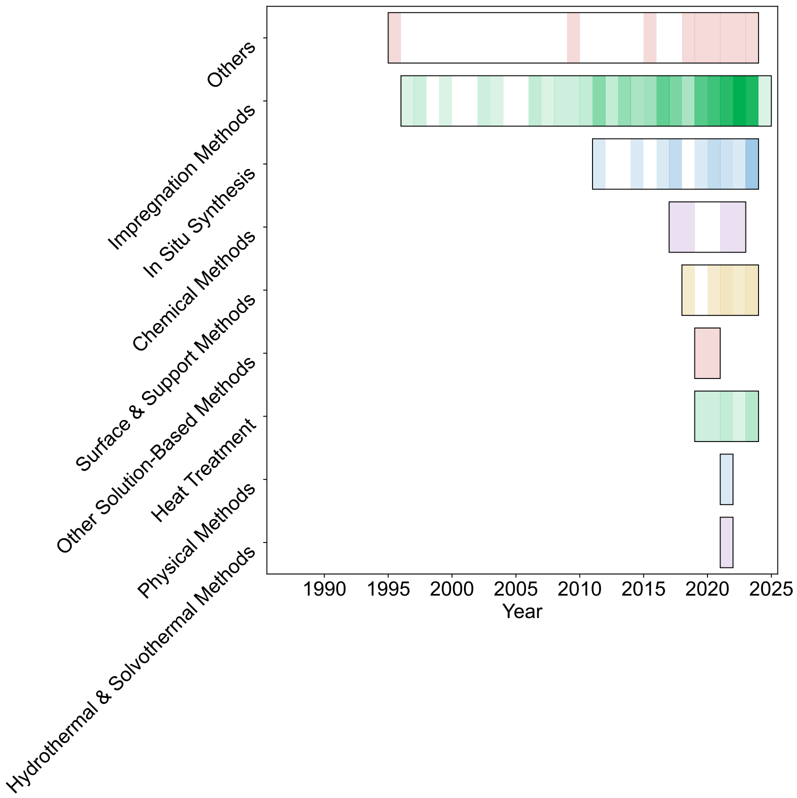

h. Types of Enhanced Performance

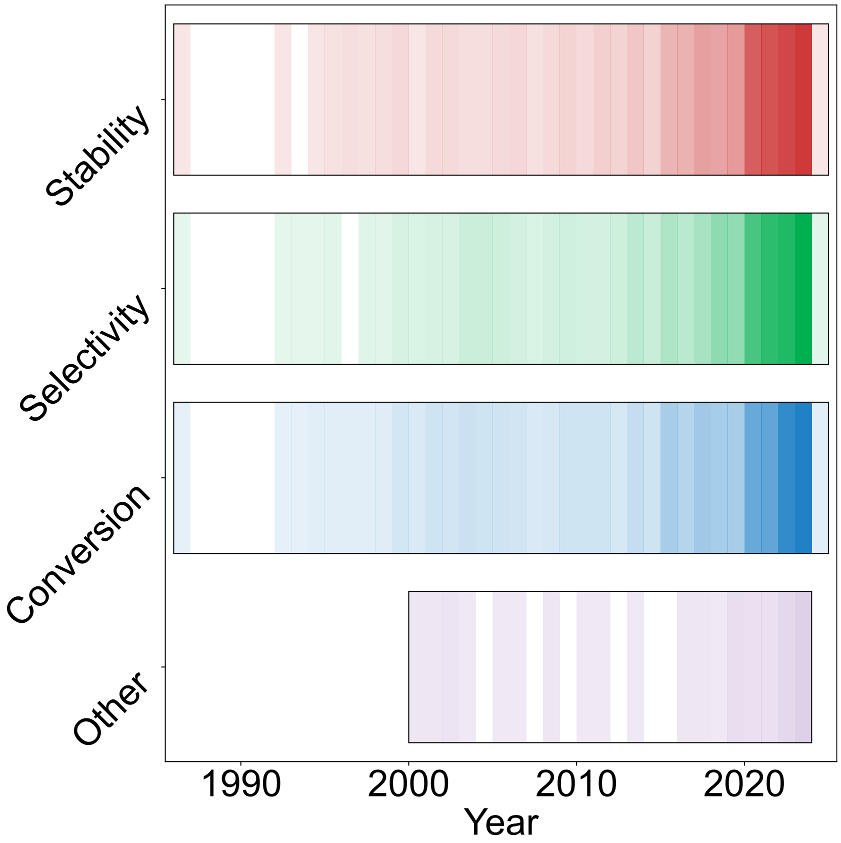

i. Sources of Performance Enhancement

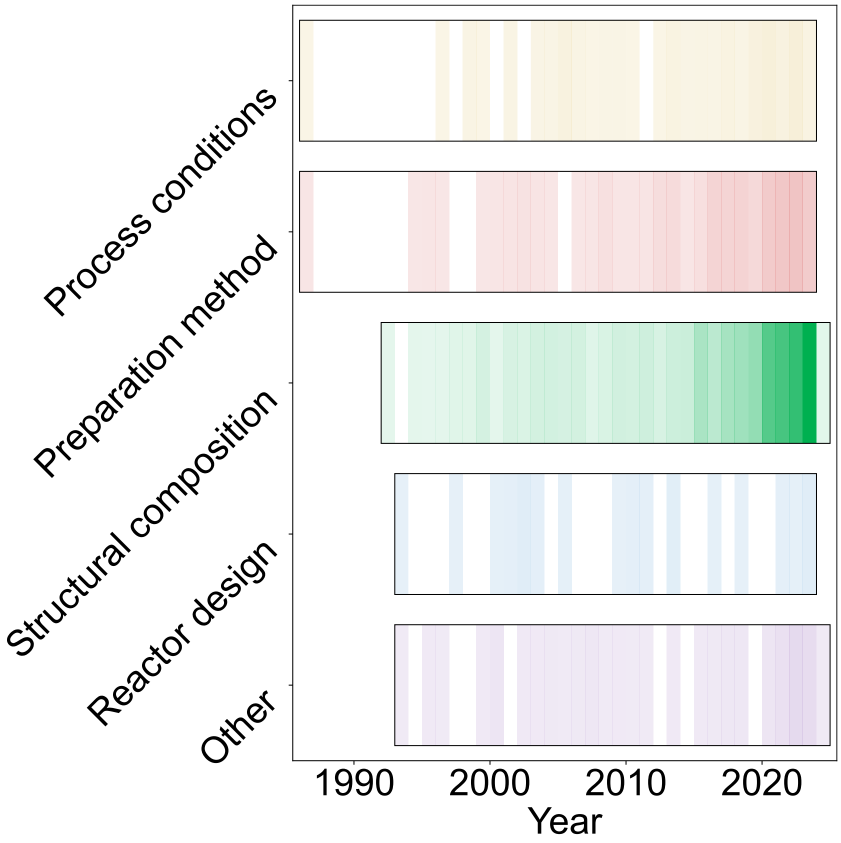

Fig.A1 Gantt charts of annual publication numbers. a, Active species elements. **b,** Composition elements. **c,** Promoter elements. **d,** Catalyst type. **e,** Support materials. **f,** Alloy structure types. **g,** Alloy preparation methods. **h,** Types of enhanced performance. **i,** Sources of performance enhancement. The varying shades of color represent the quantity of publications.

a. Active Species Elements

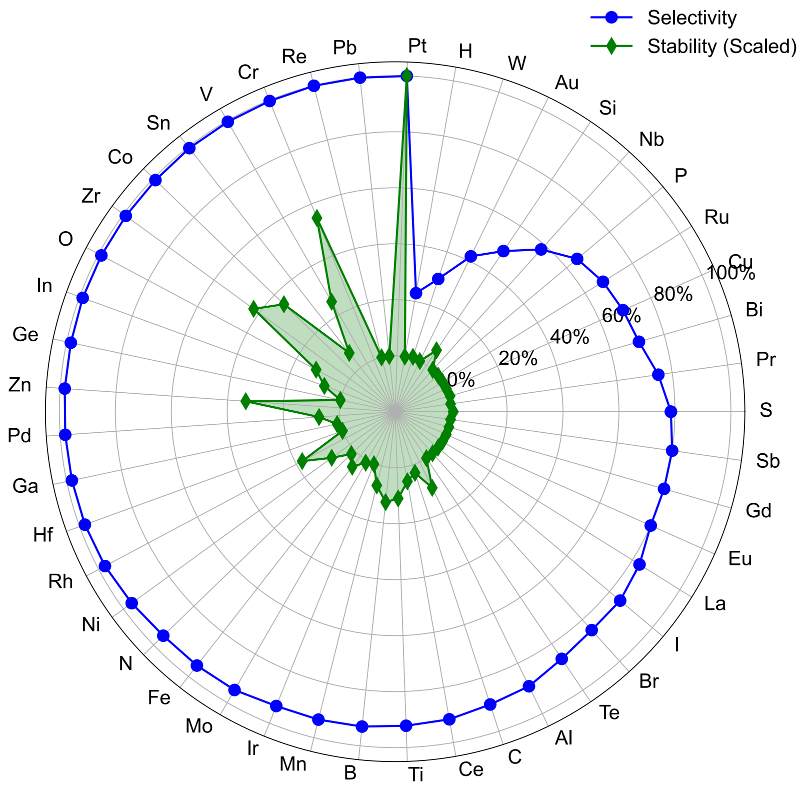

b. Composition Elements

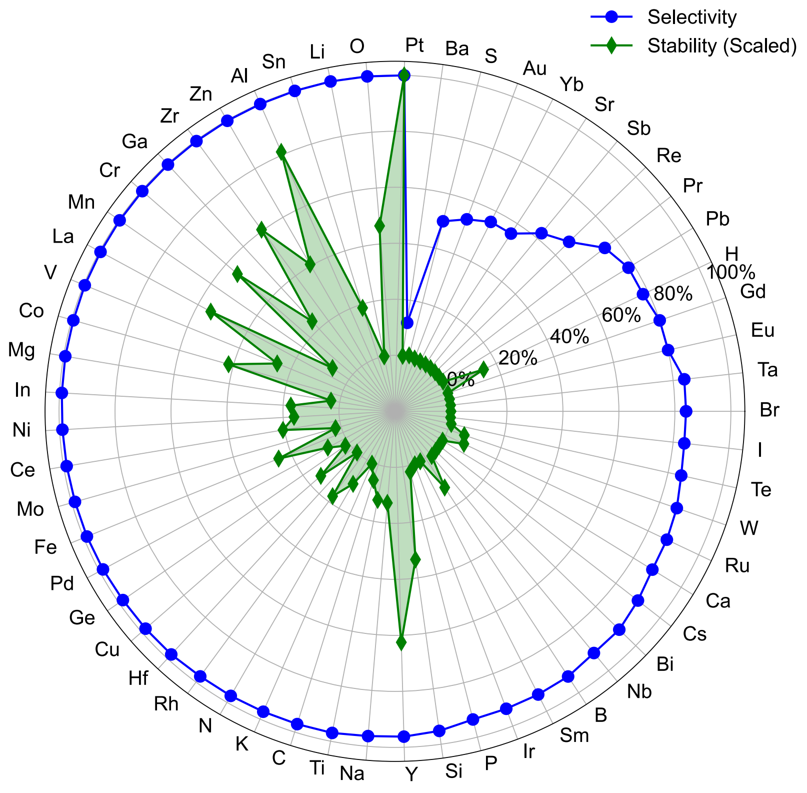

c. Promoter Elements

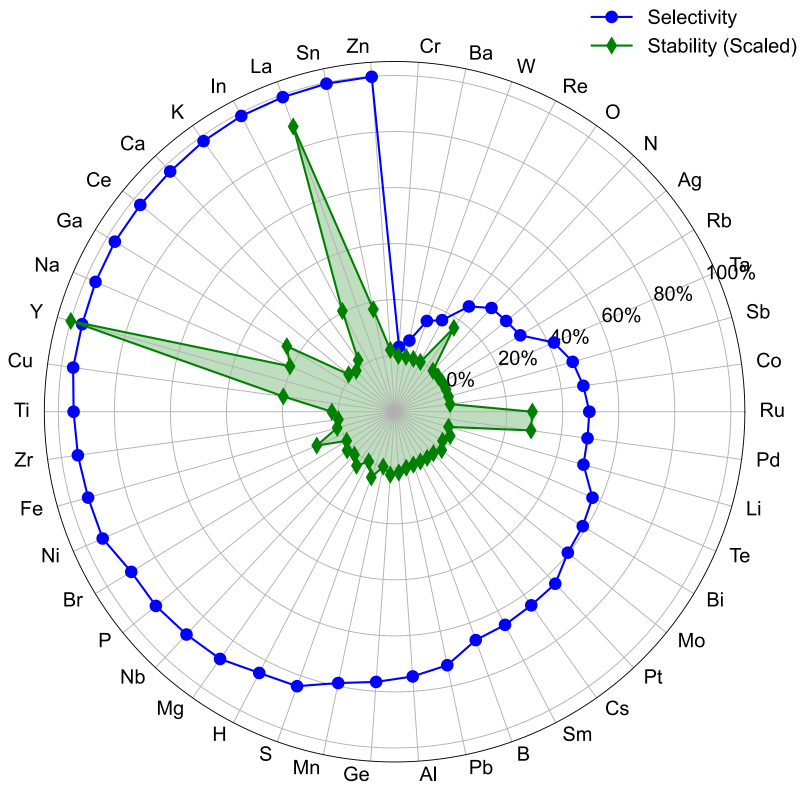

d. Catalyst Type

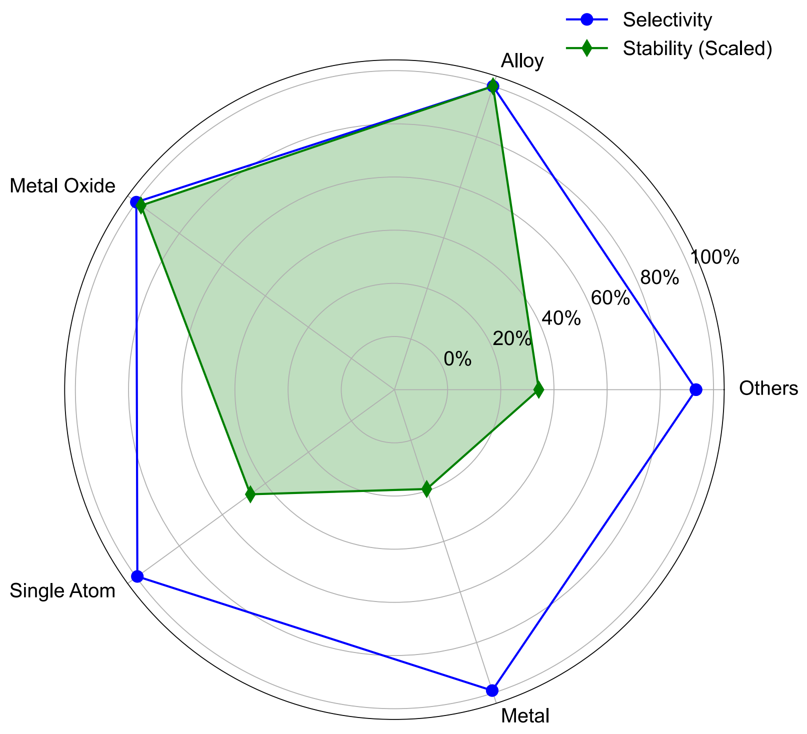

e. Support Materials

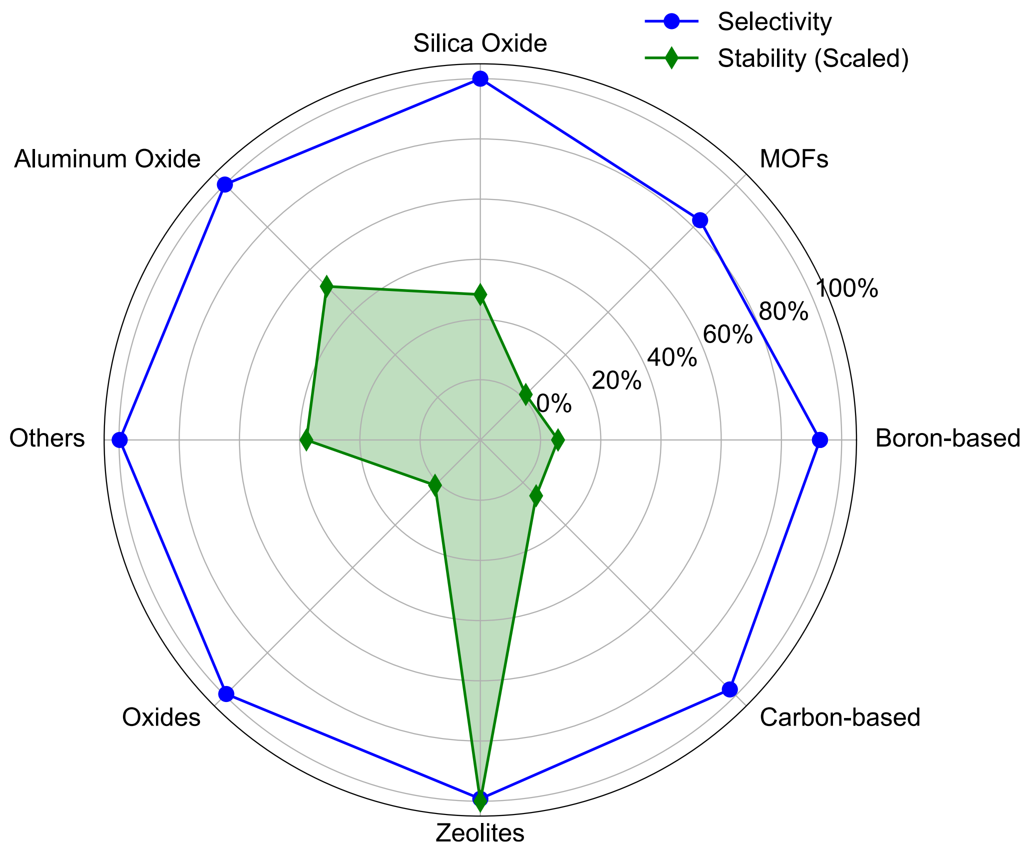

f. Alloy Structure Types

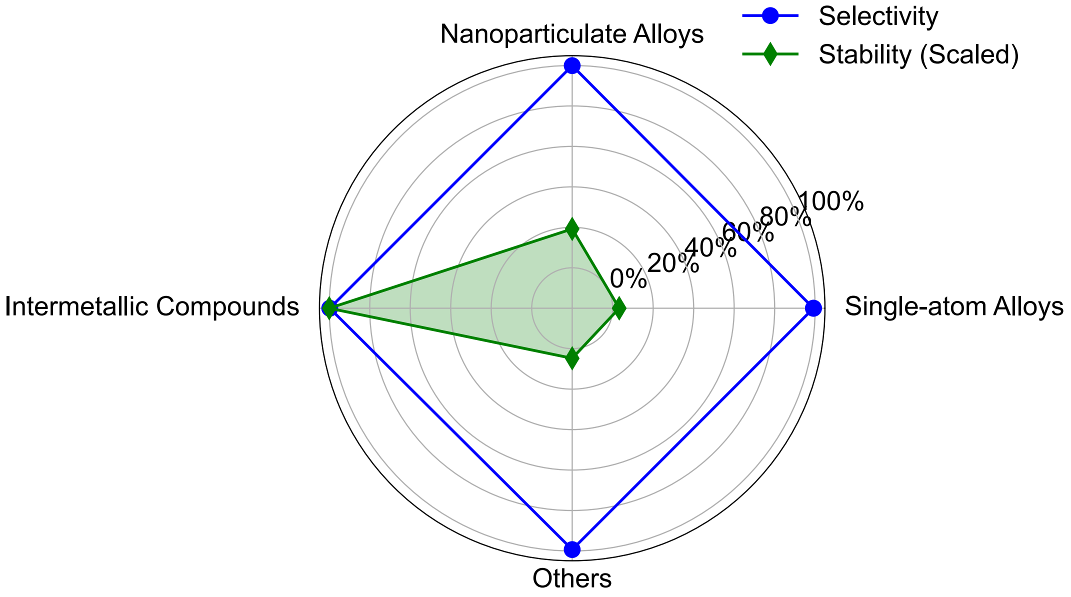

g. Alloy Preparation Methods

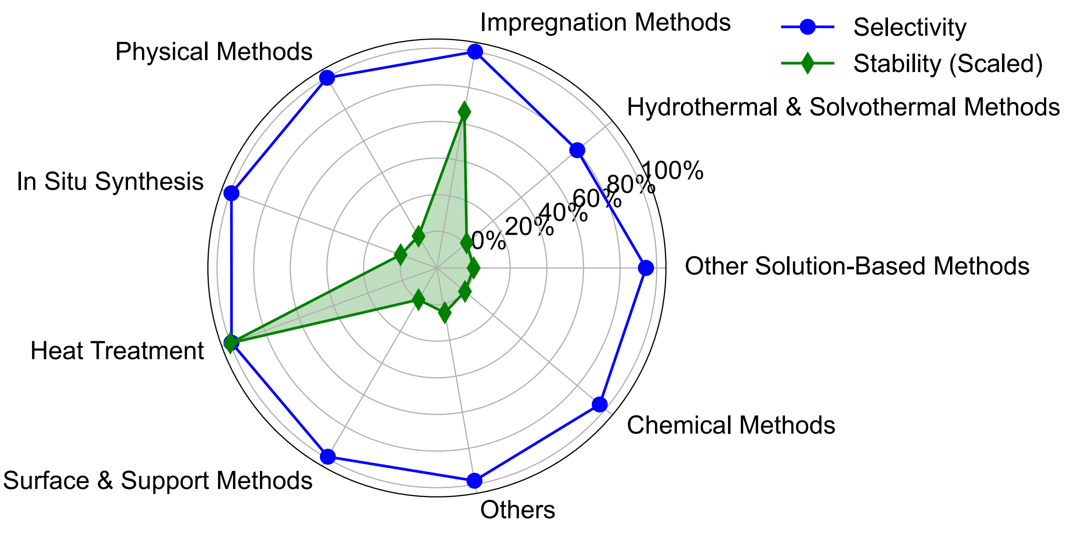

Fig.A2 Radar charts of single factor peak performance. a, Active species elements. **b,** Composition elements. **c,** Promoter elements. **d,** Catalyst type. **e,** Support materials. **f,** Alloy structure types. **g,** Alloy preparation methods. Stability values are scaled to percentages relative to their maximum values.

a. Type - Structure type

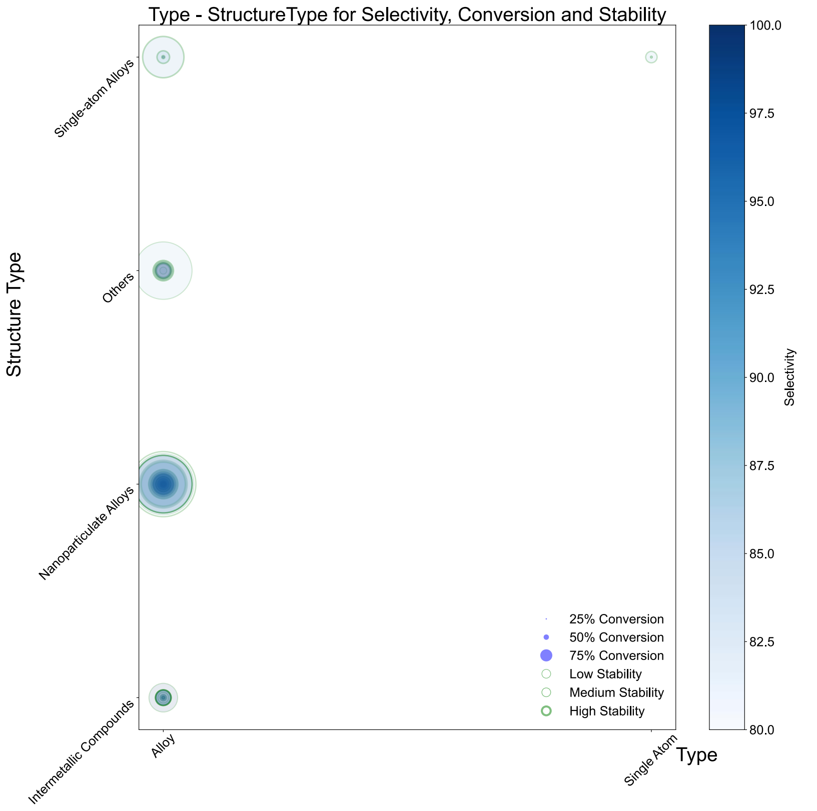

b. Type - Support material

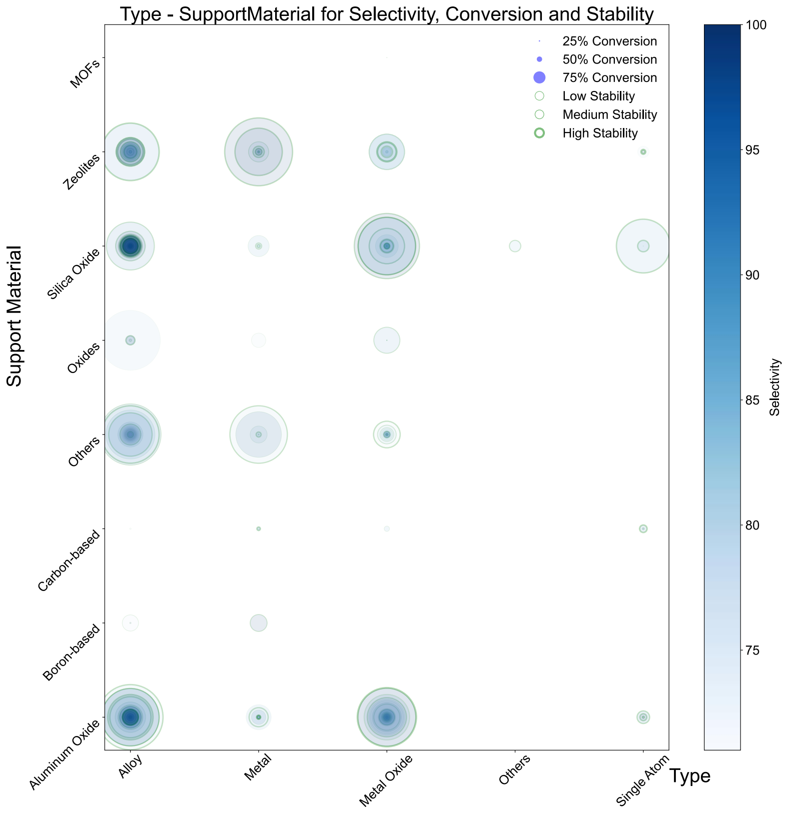

c. Type - Preparation method

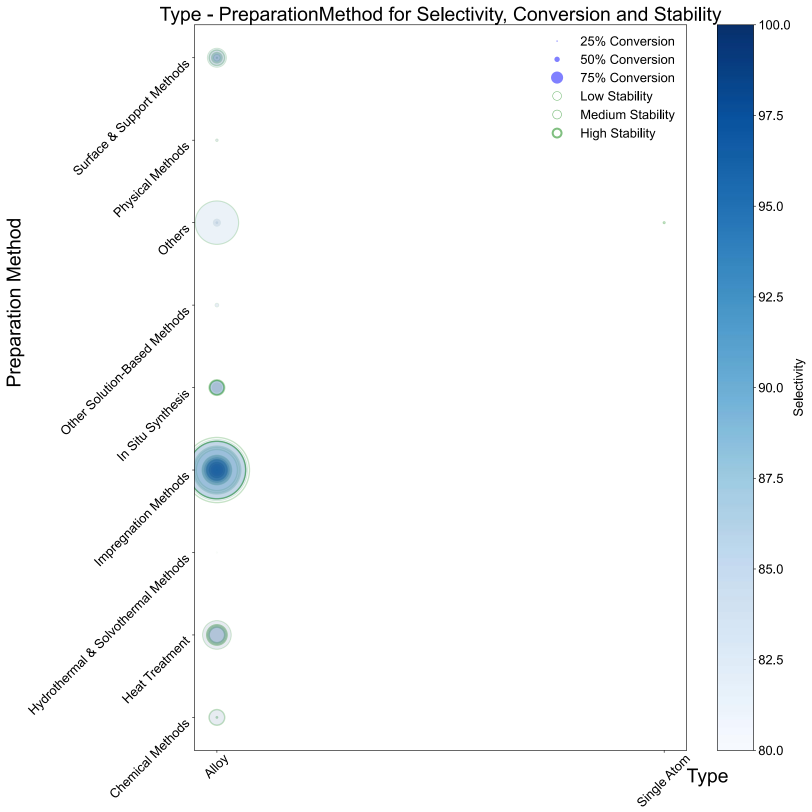

d. Type - Inlet flow rate

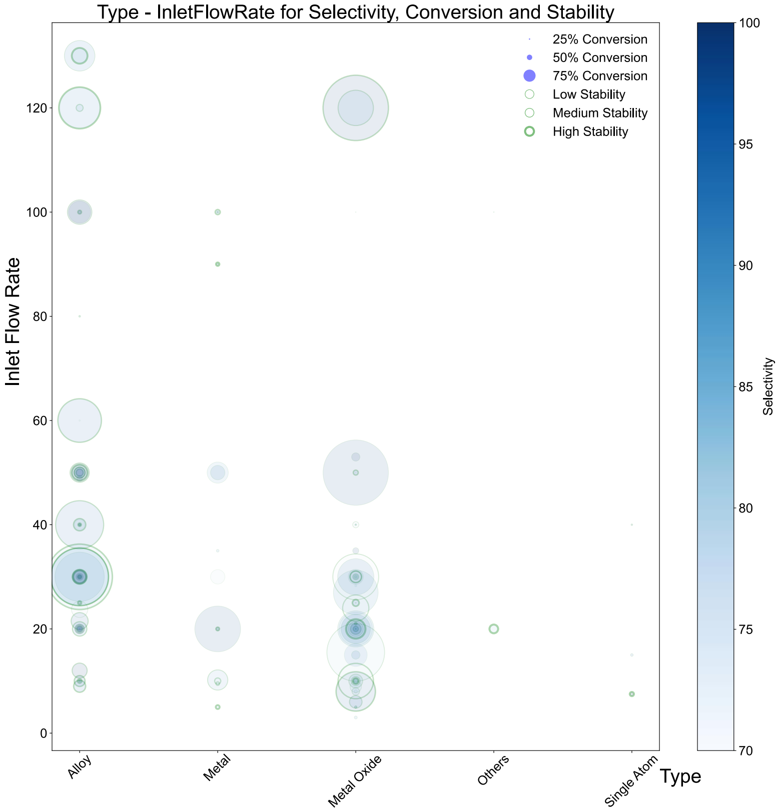

e. Type - Reaction temperature

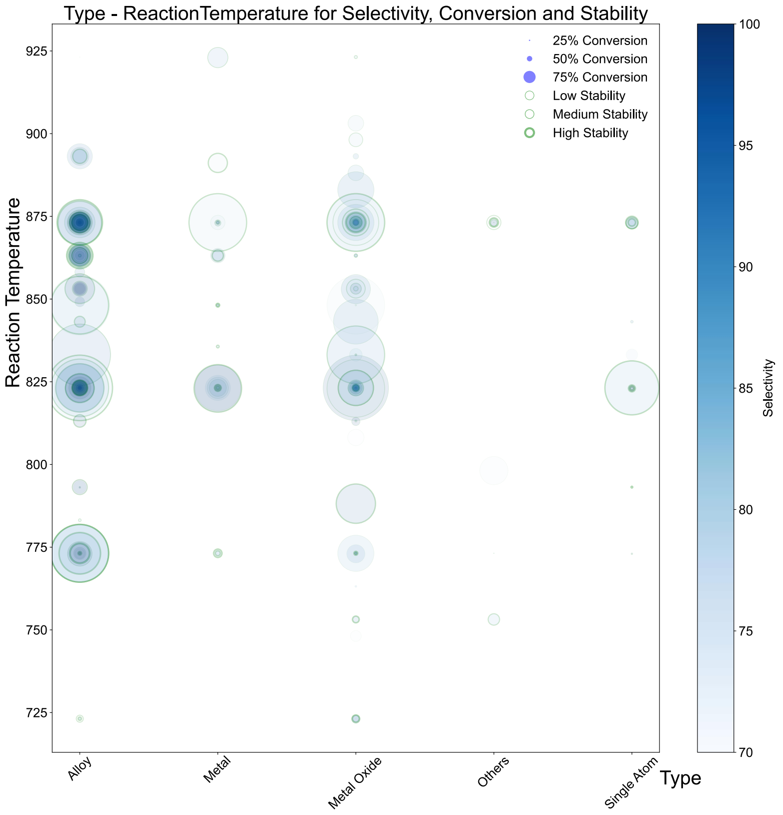

f. Type - Propane partial pressure

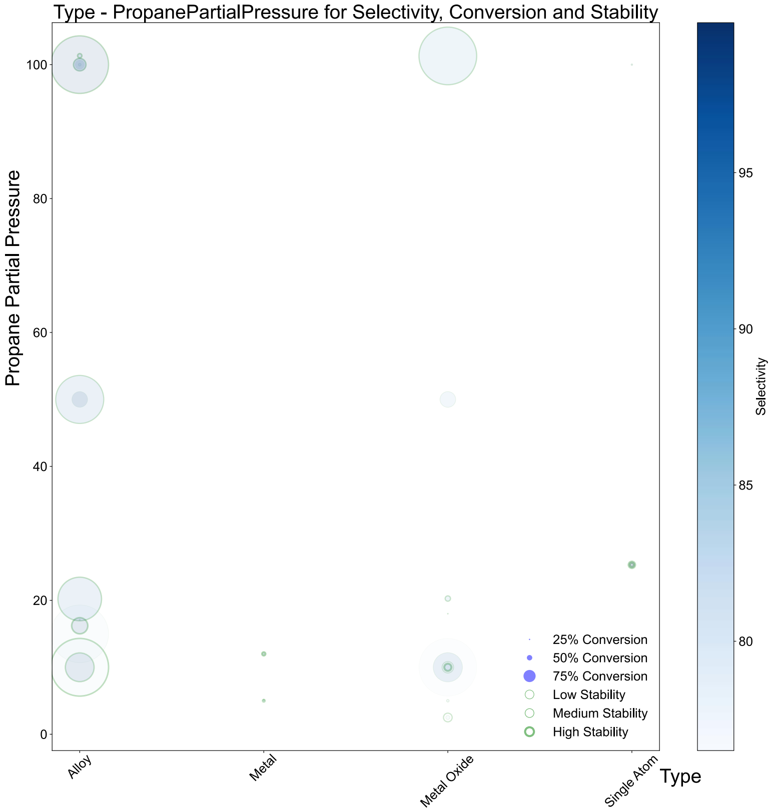

g. Type - Composition elements

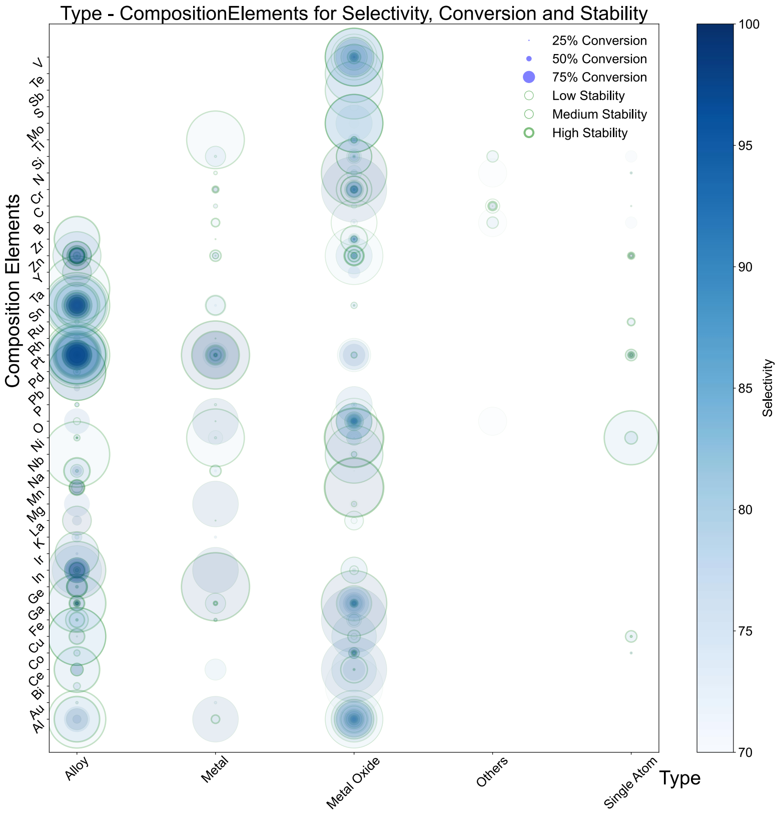

h. Type - Promoter elements

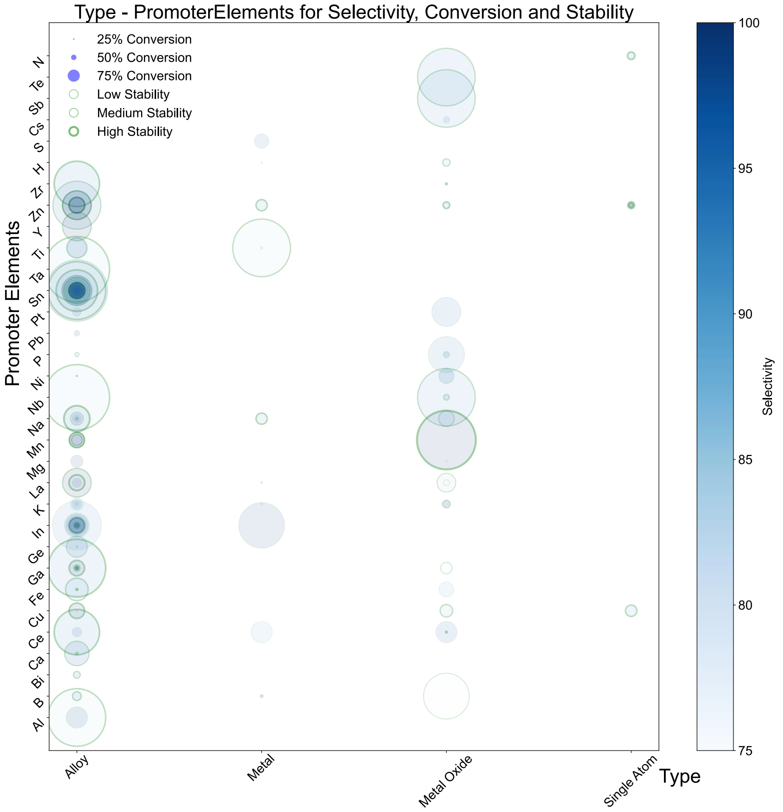

i. Type - Active species elements

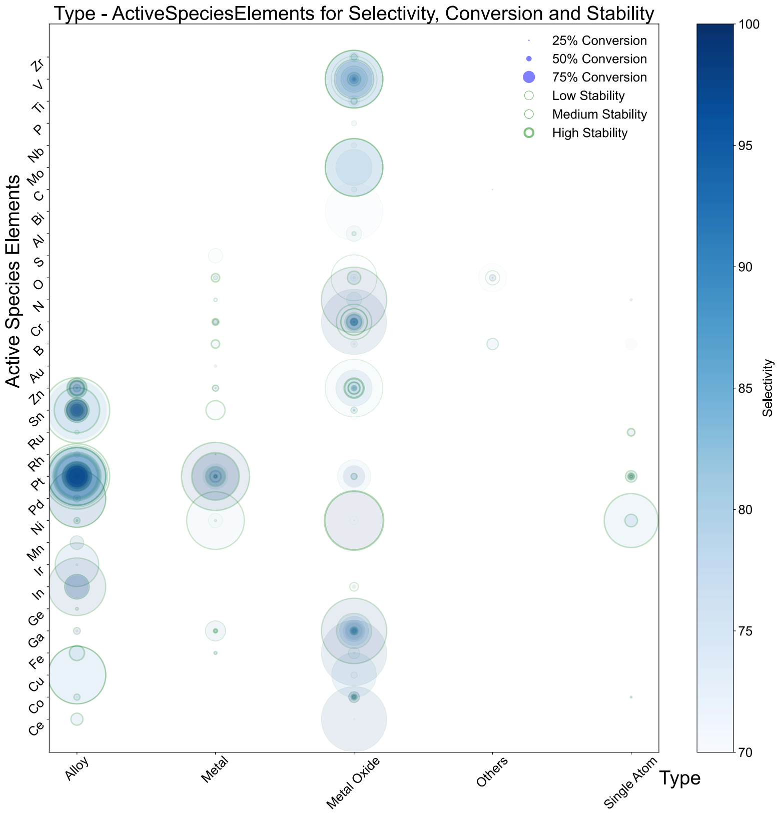

j. Structure type - Support material

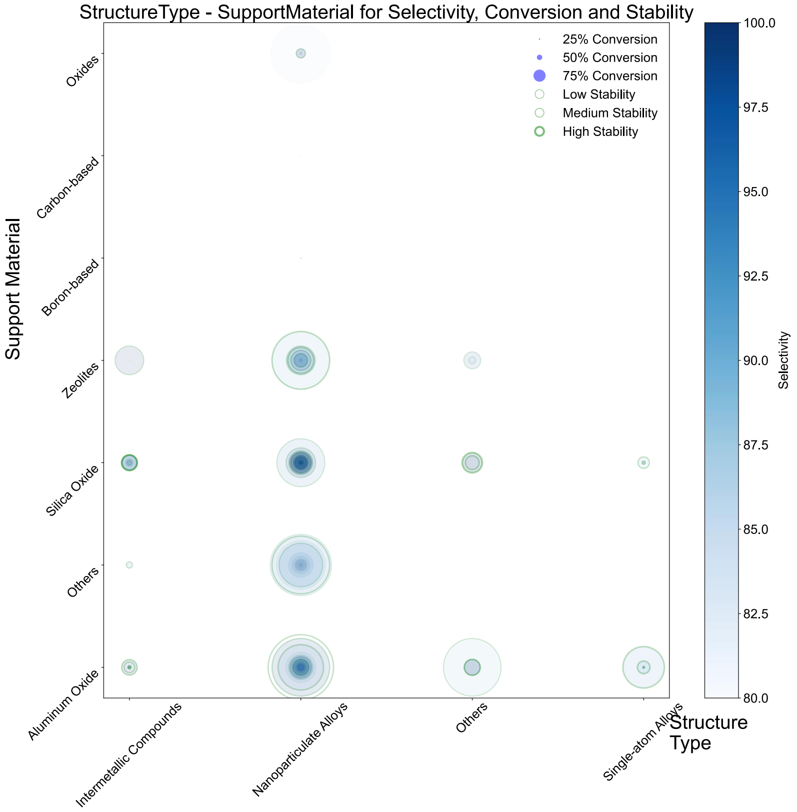

k. Structure type - Preparation method

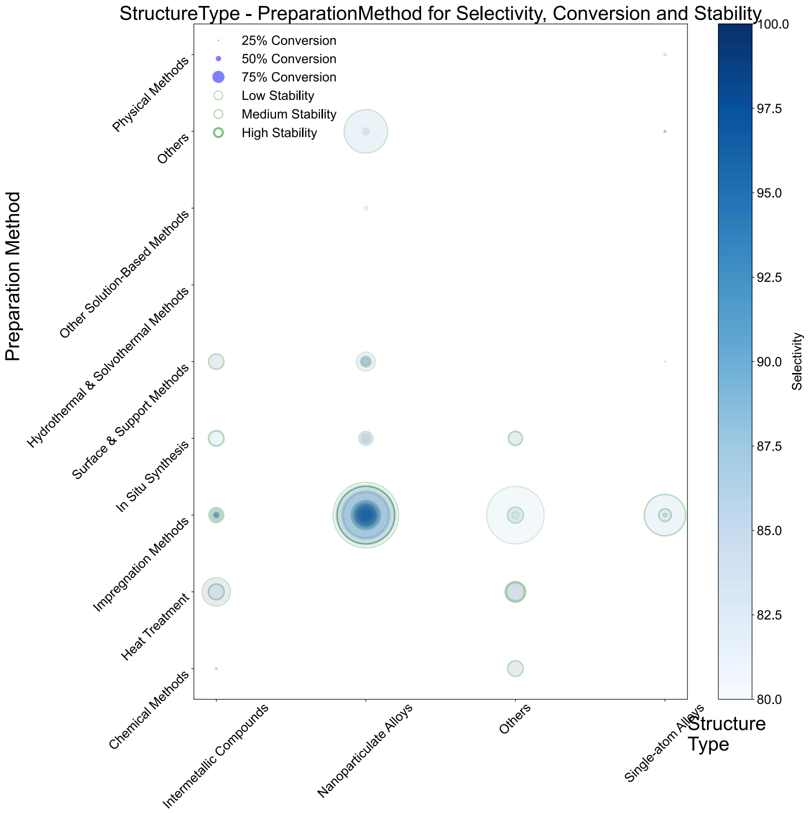

l. Structure type - Inlet flow rate

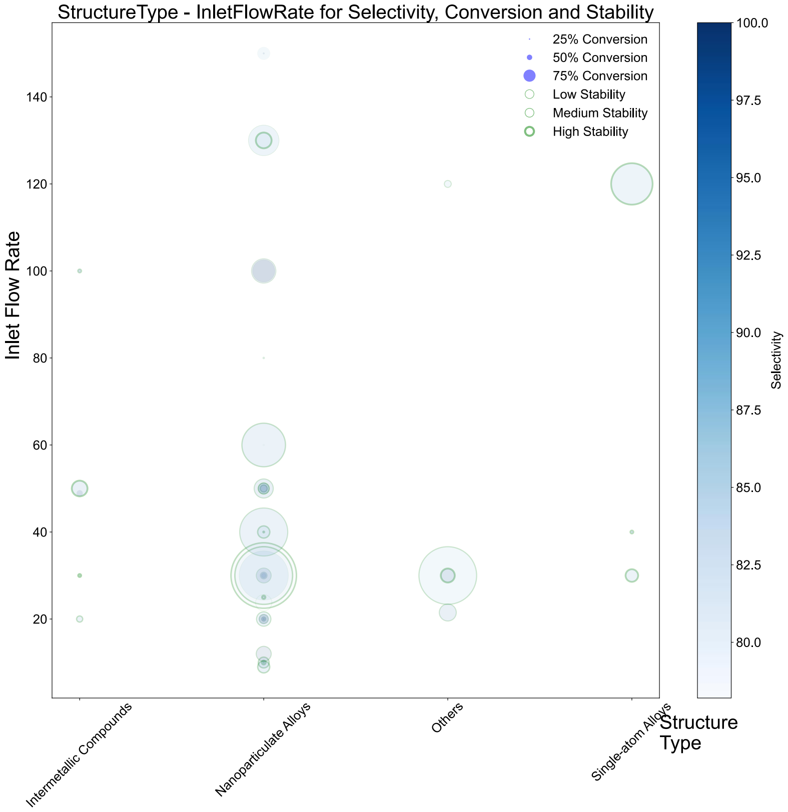

m. Structure type - Reaction temperature

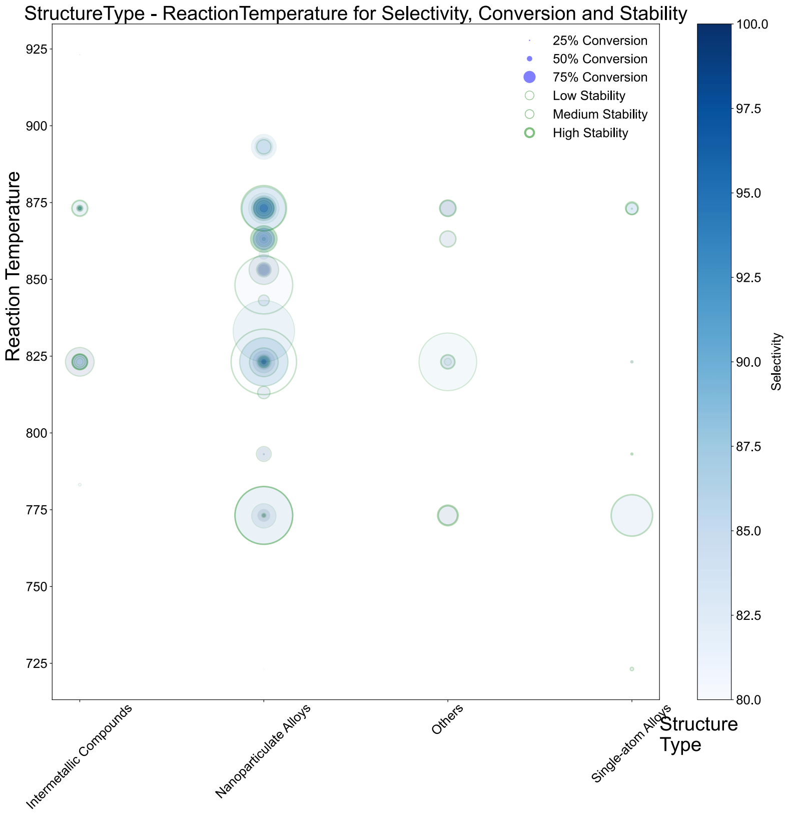

n. Structure type - Propane partial pressure

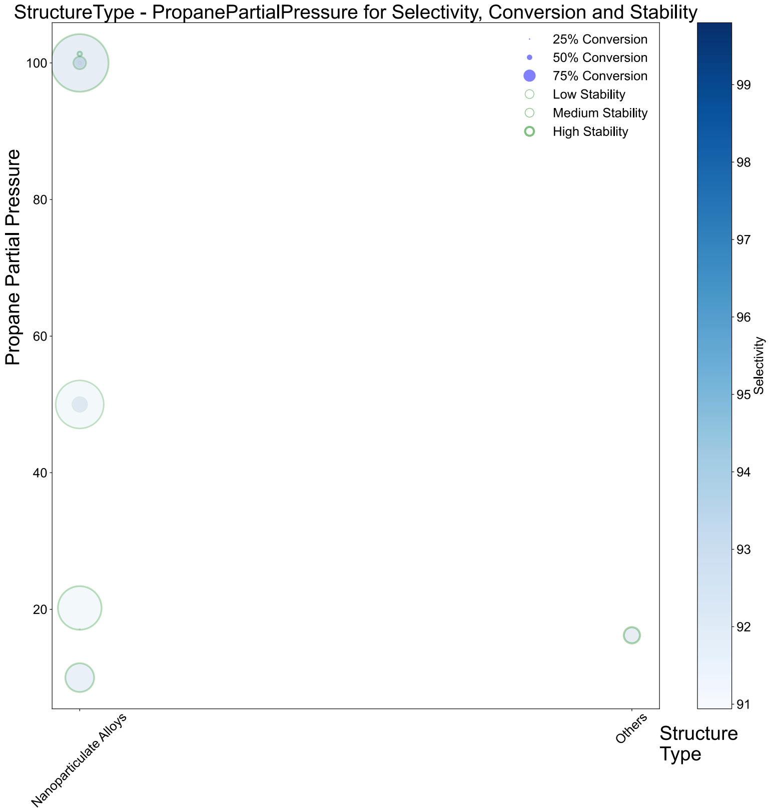

o. Structure type - Composition elements

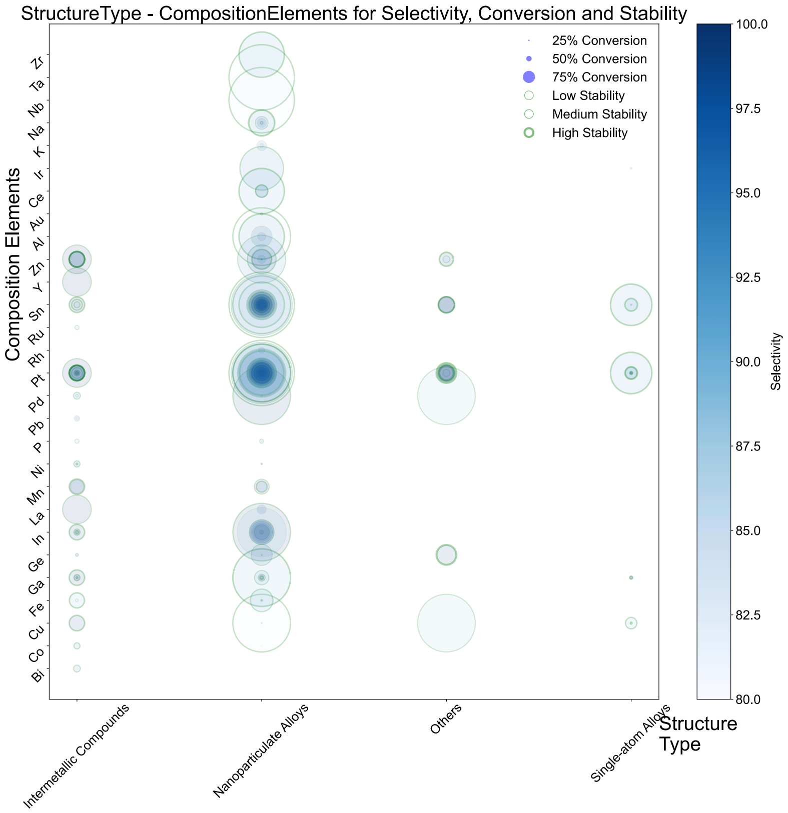

p. Structure type - Promoter elements

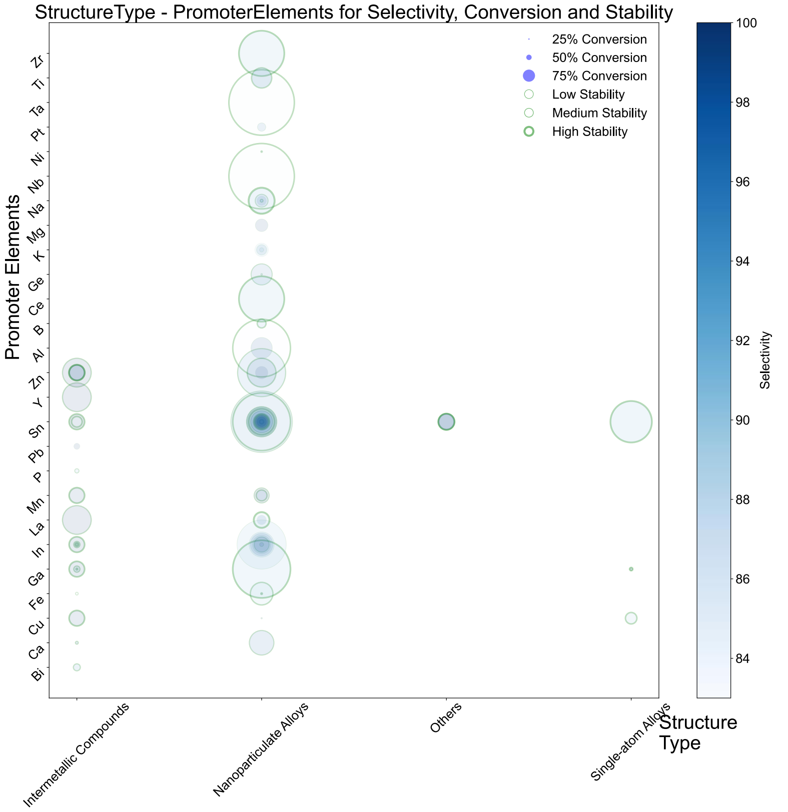

q. Structure type - Active species elements

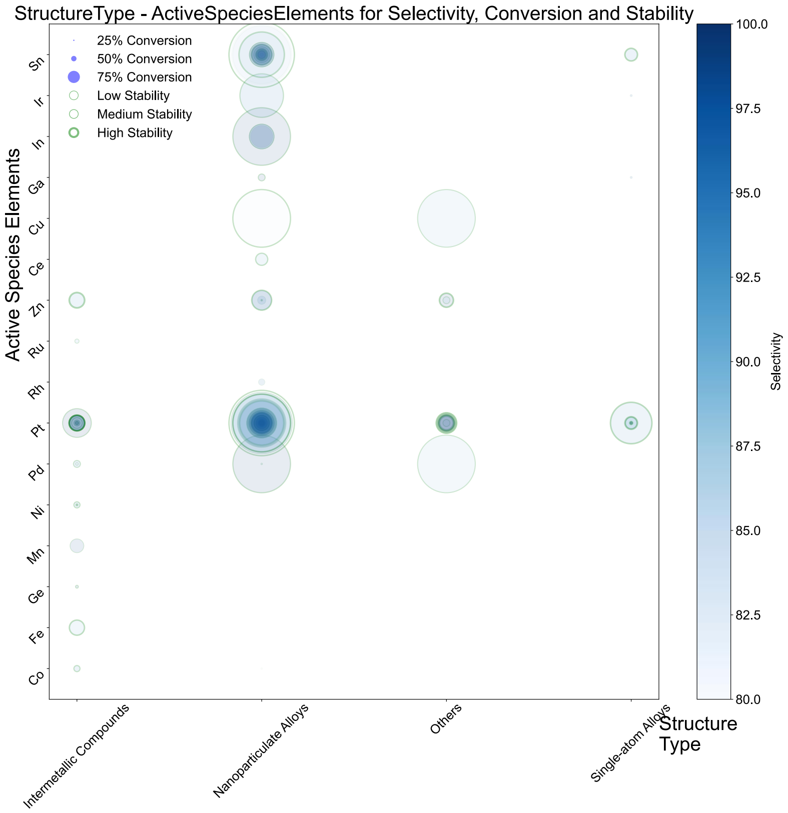

r. Support material - Preparation method

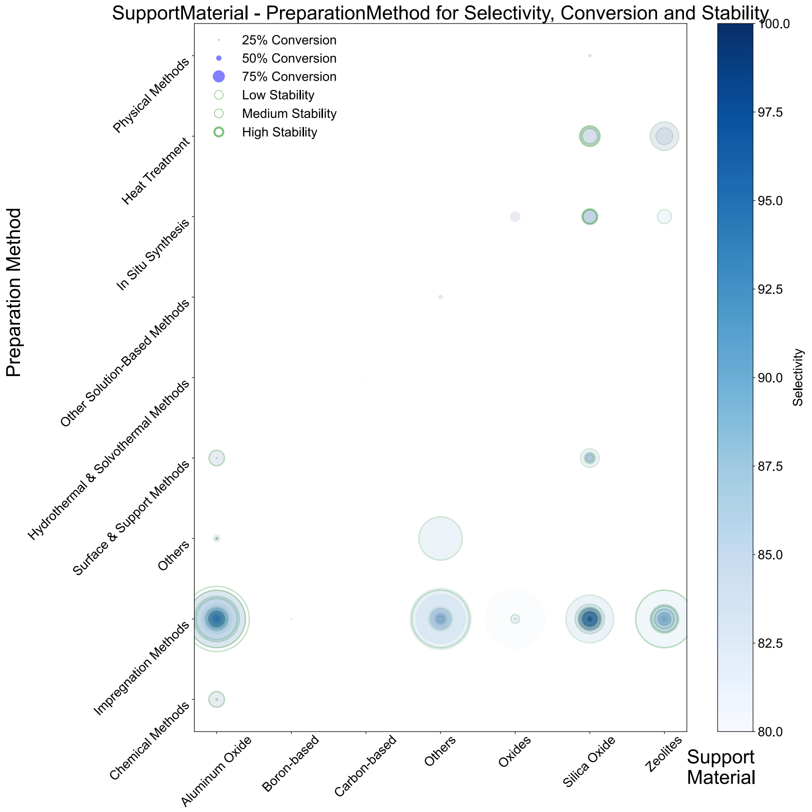

s. Support material - Inlet flow rate

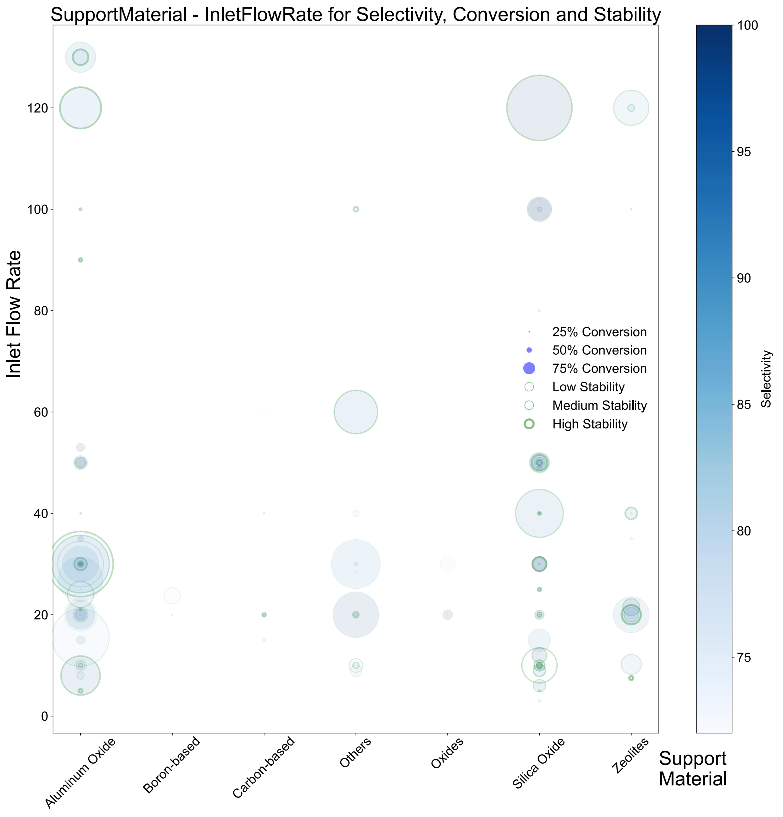

t. Support material - Reaction temperature

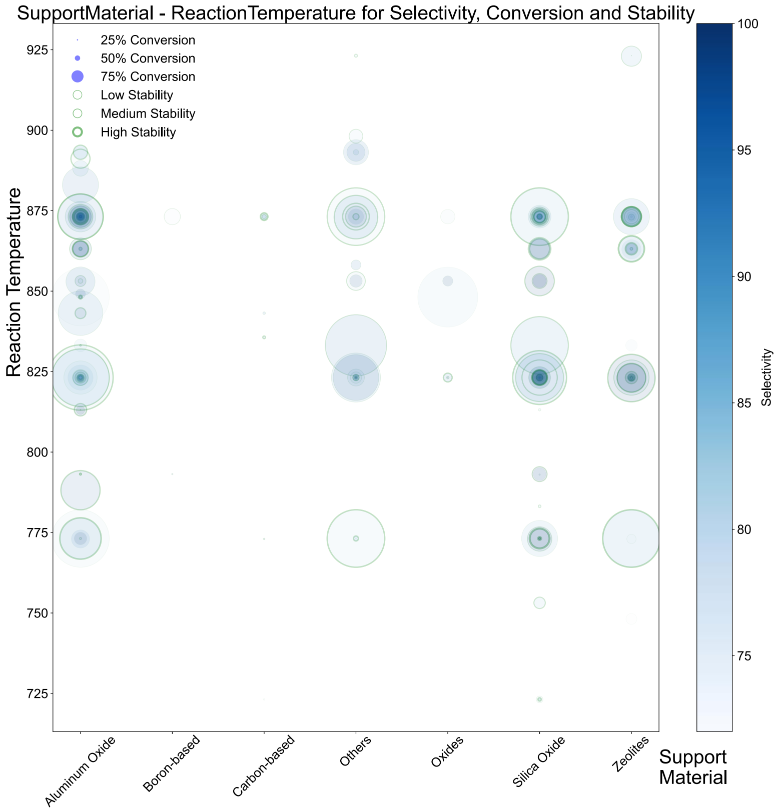

u. Support material - Propane partial pressure

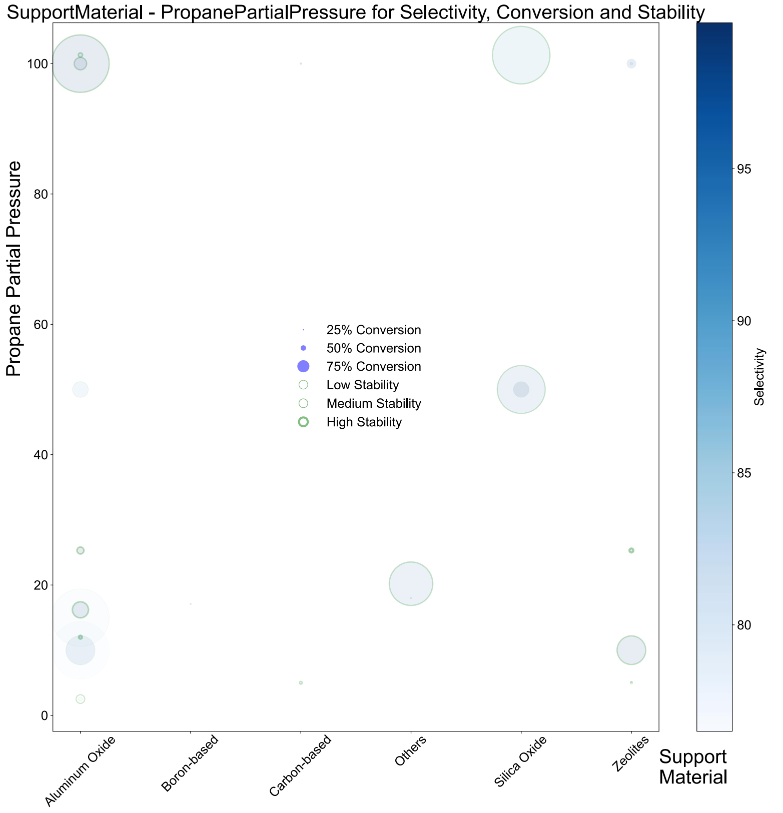

v. Support material - Composition elements

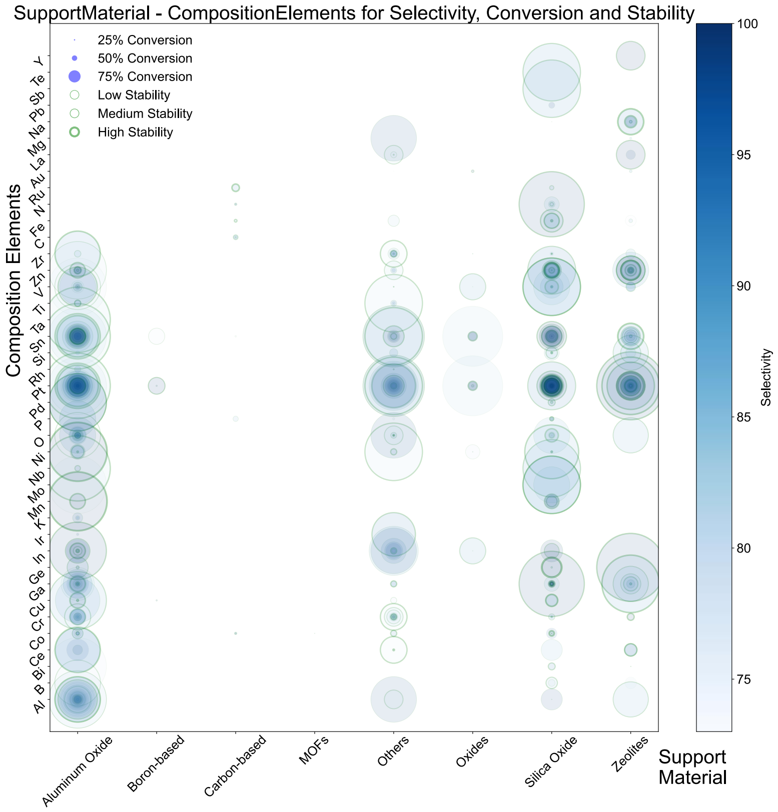

w. Support material - Promoter elements

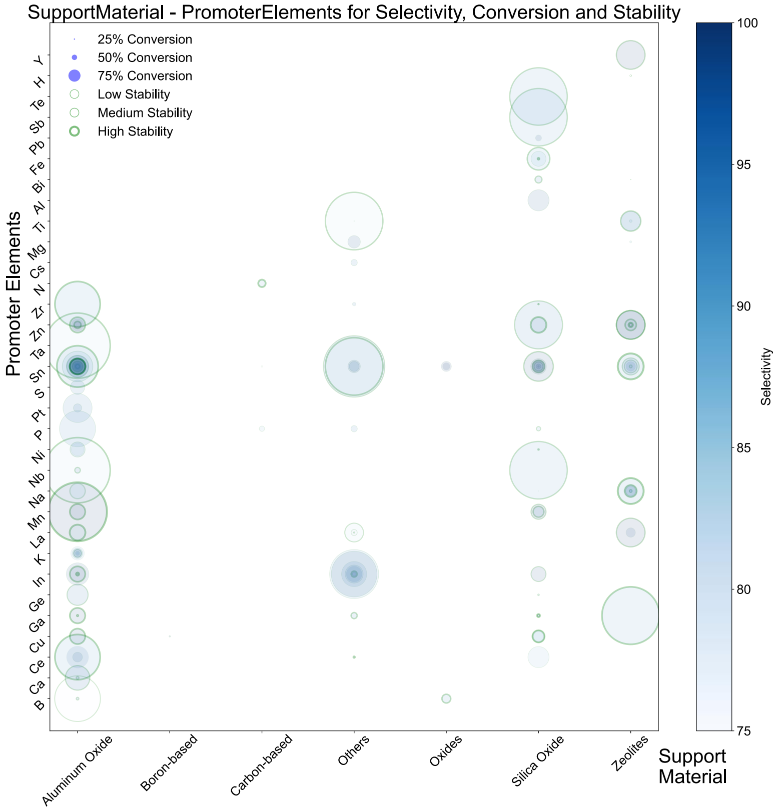

x. Support material - Active species elements

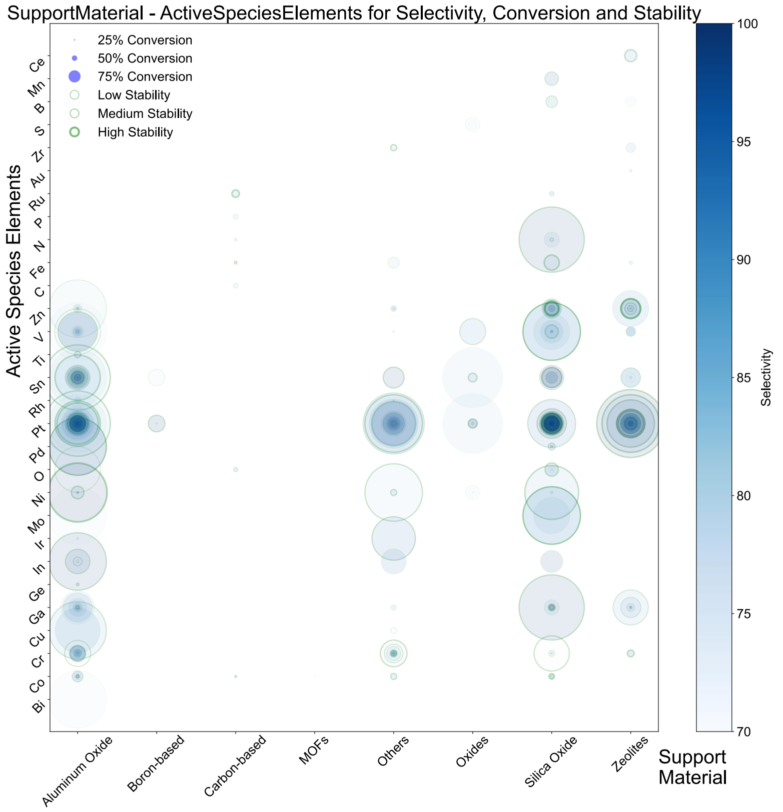

y. Preparation method - Inlet flow rate

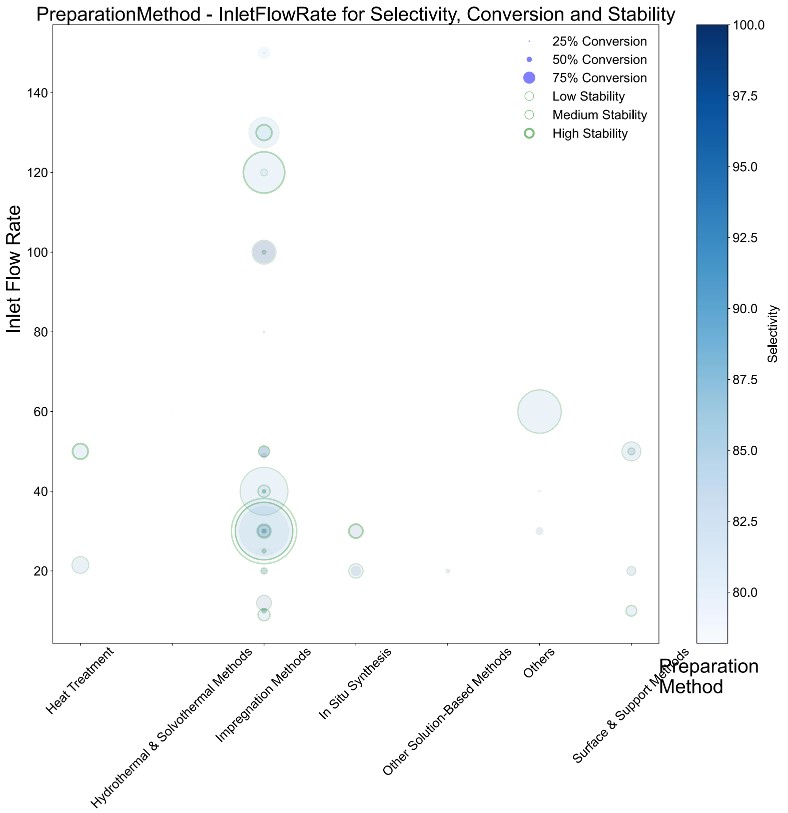

z. Preparation method - Reaction temperature

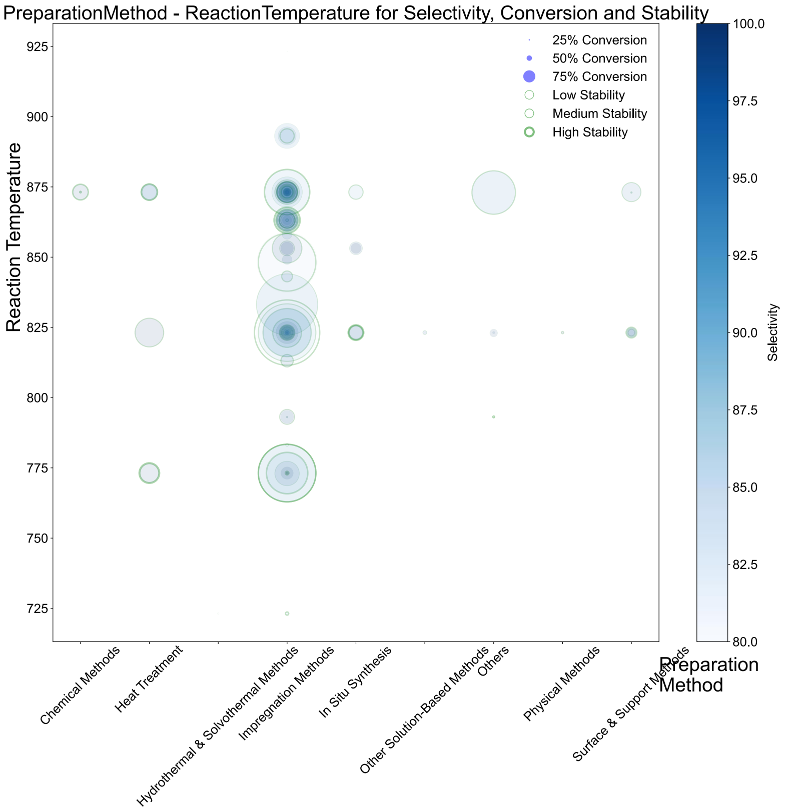

aa. Preparation method - Propane partial pressure

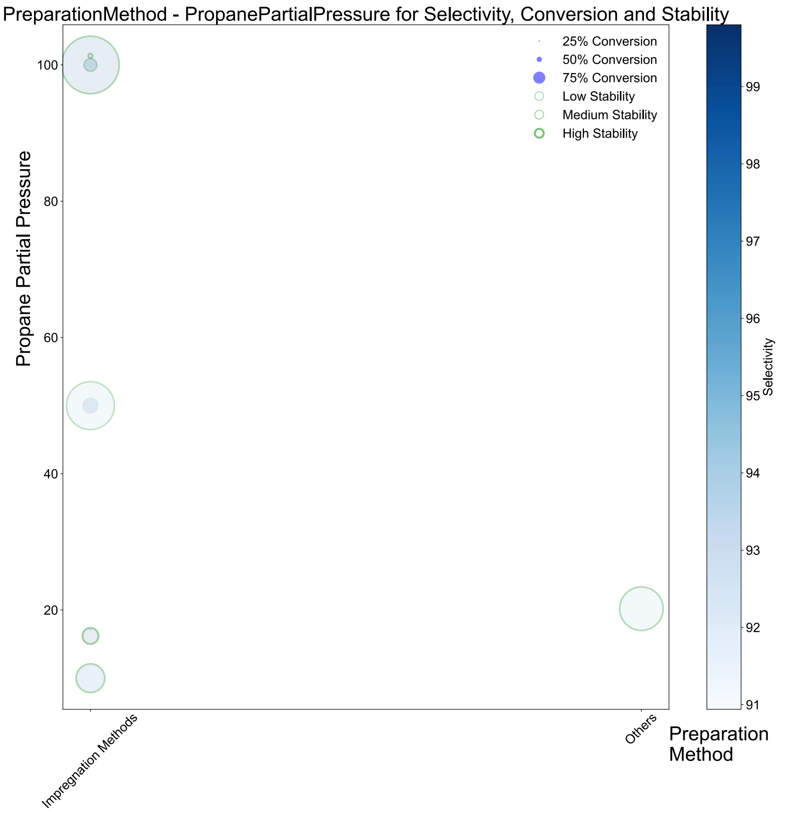

ab. Preparation method - Composition elements

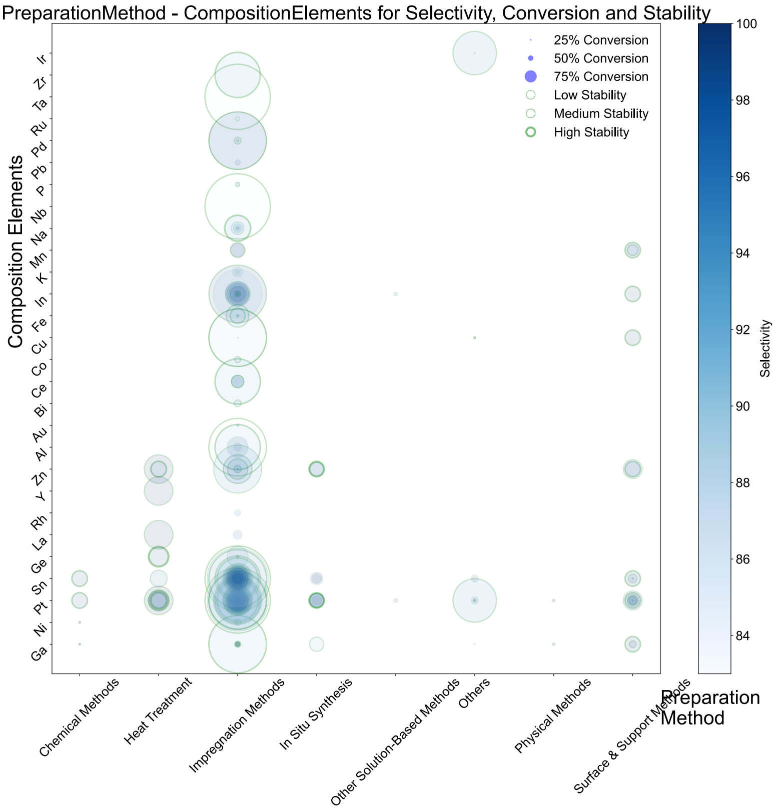

ac. Preparation method - Promoter elements

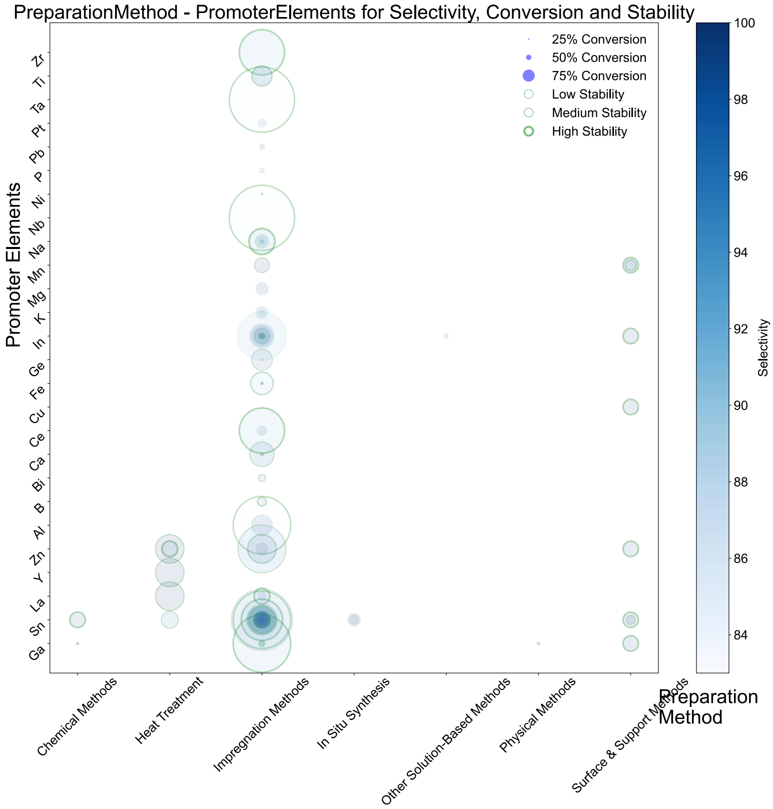

ad. Preparation method - Active species elements

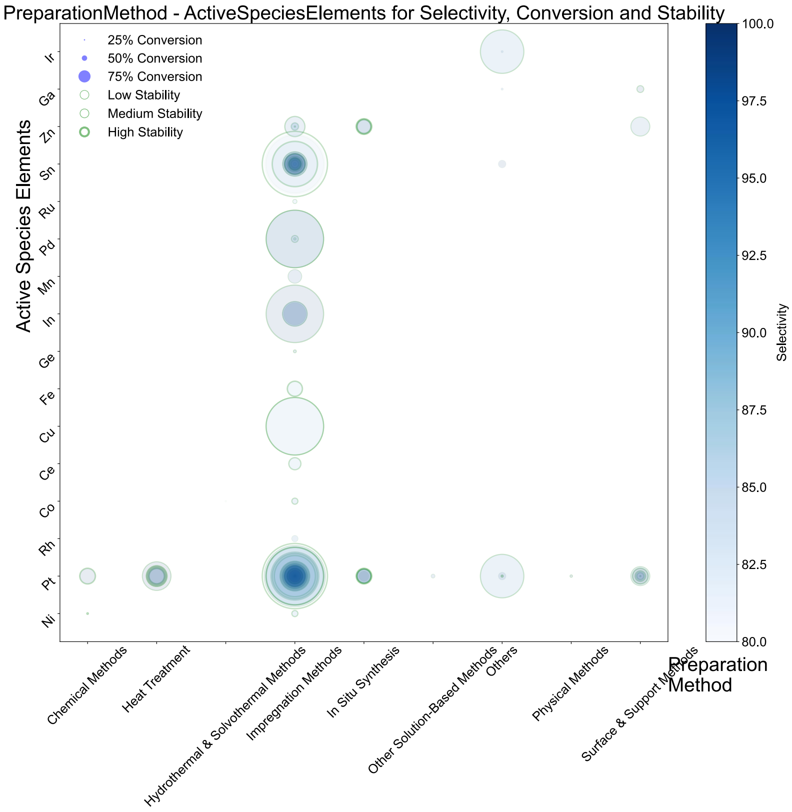

ae. Inlet flow rate - Reaction temperature

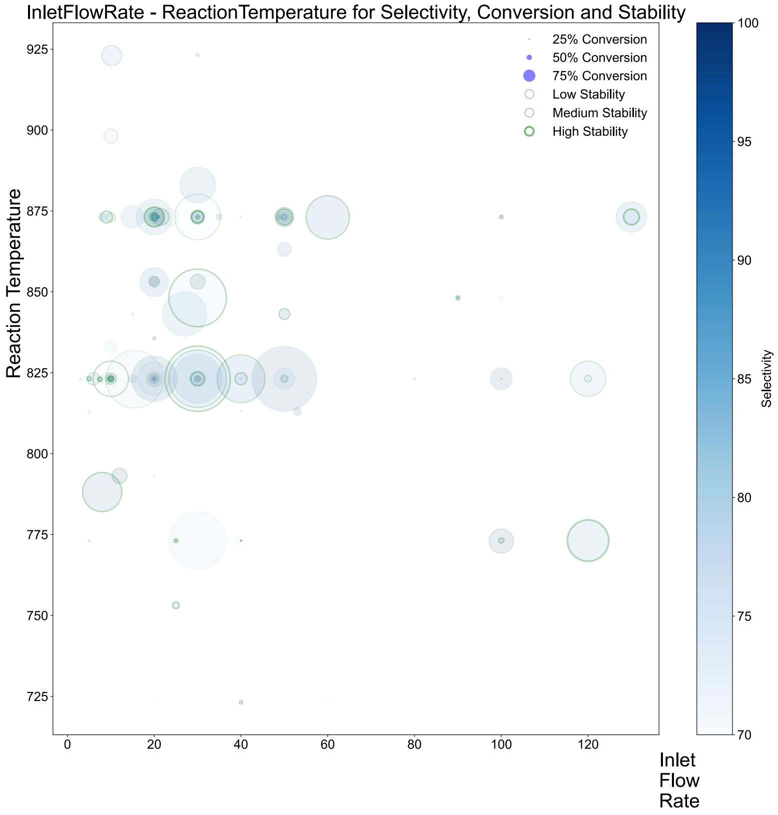

af. Inlet flow rate - Propane partial pressure

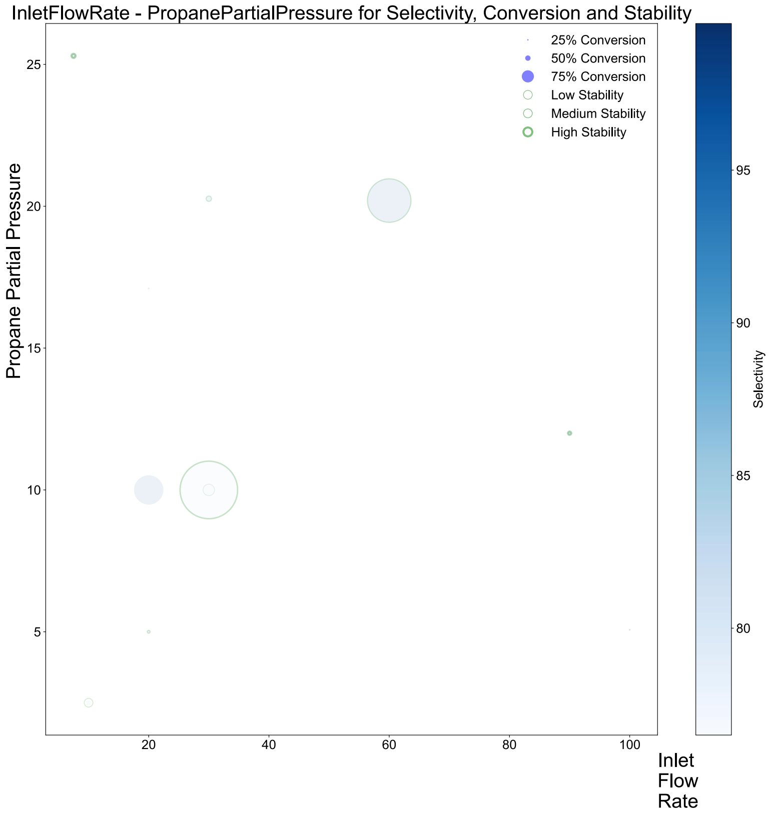

ag. Inlet flow rate - Composition elements

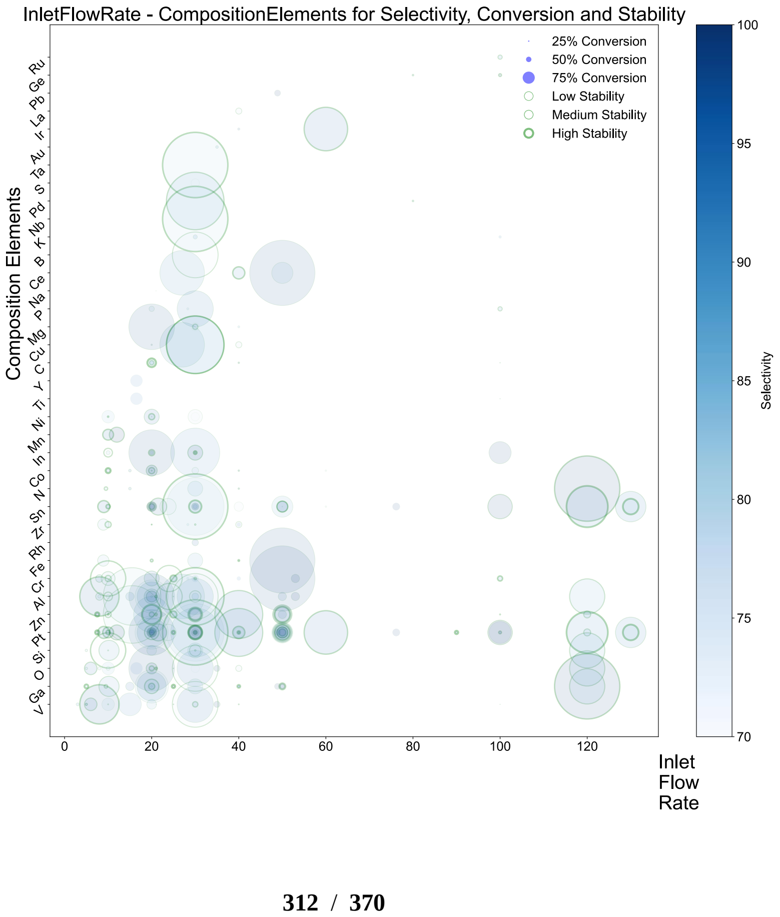

ah. Inlet flow rate - Promoter elements

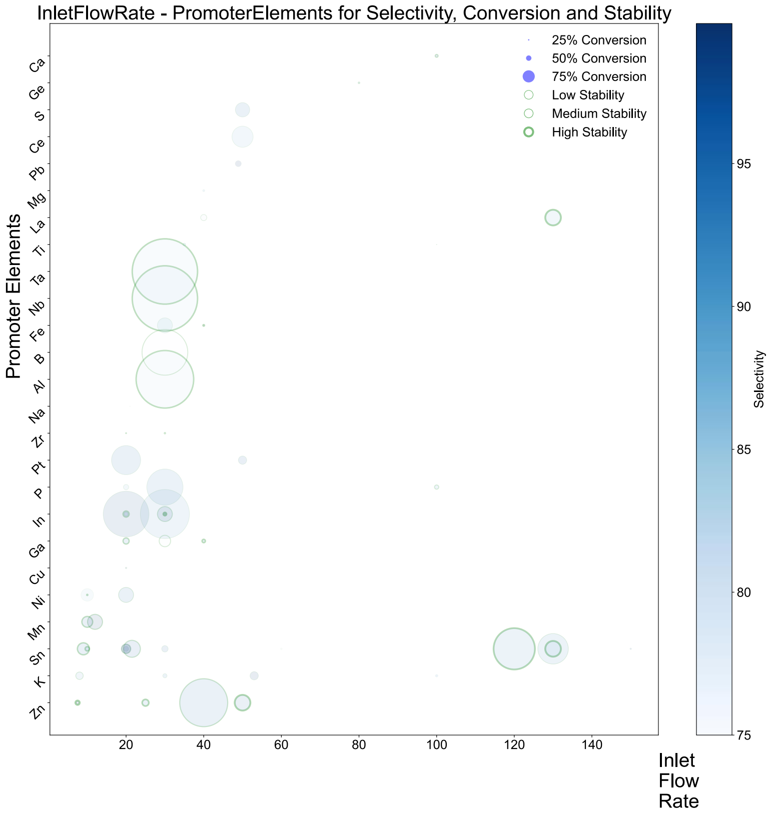

ai. Inlet flow rate - Active species elements

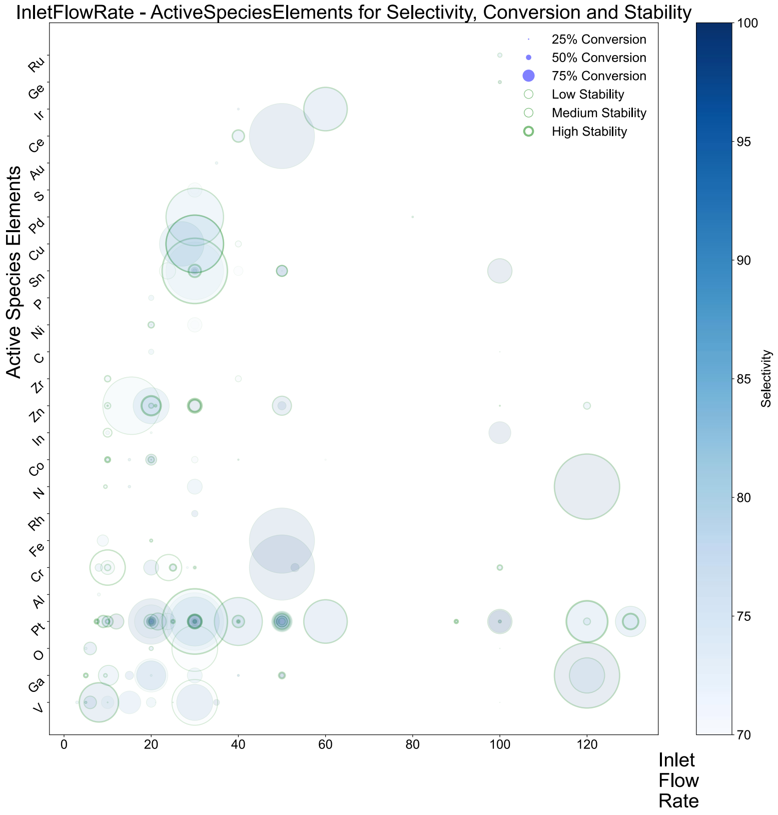

aj. Reaction temperature - Propane partial pressure

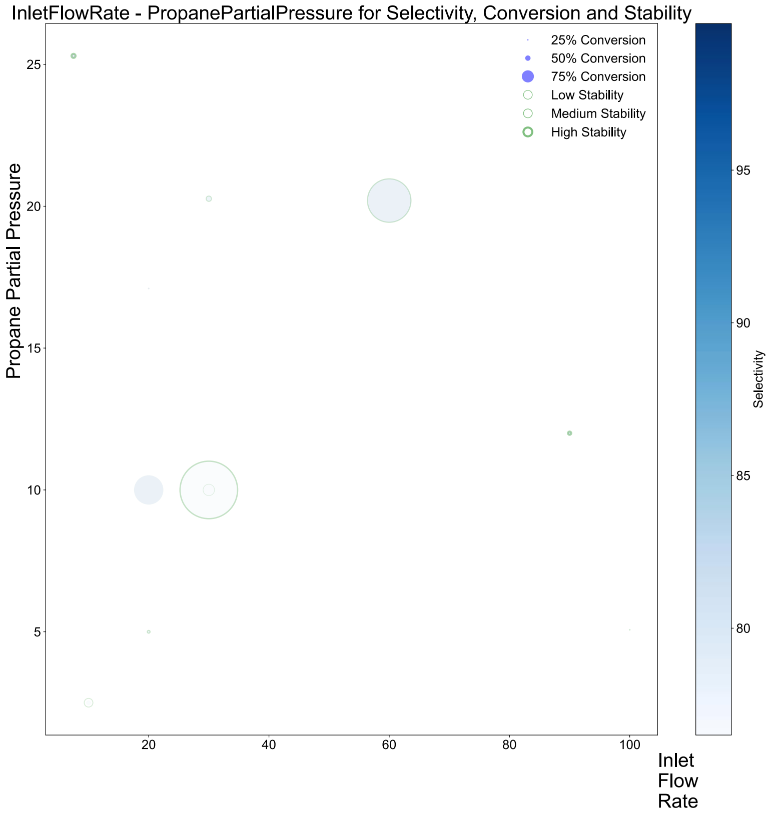

ak. Reaction temperature - Composition elements

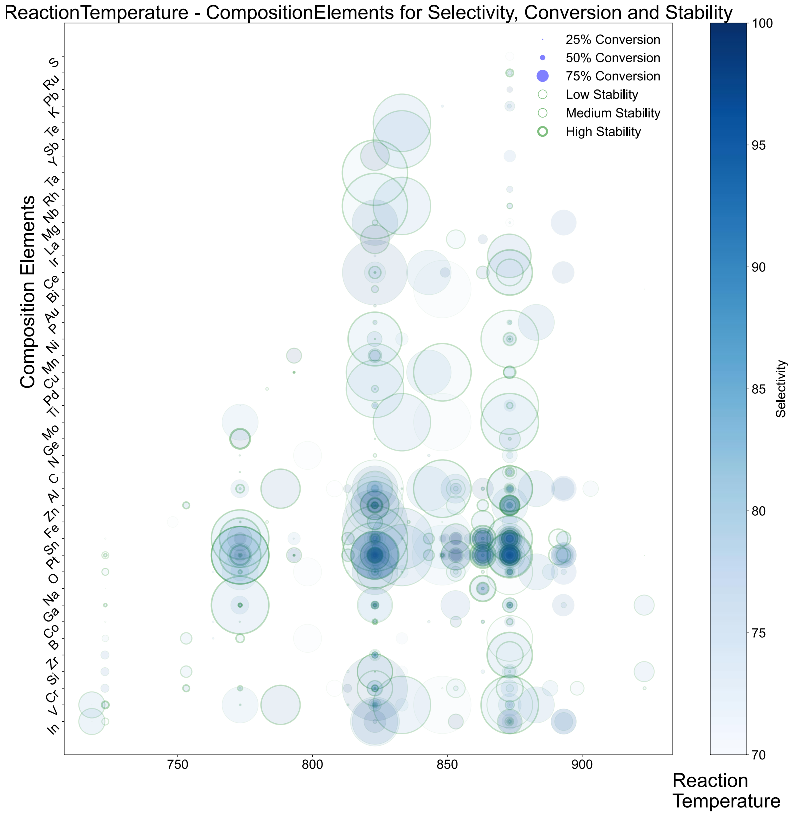

al. Reaction temperature - Promoter elements

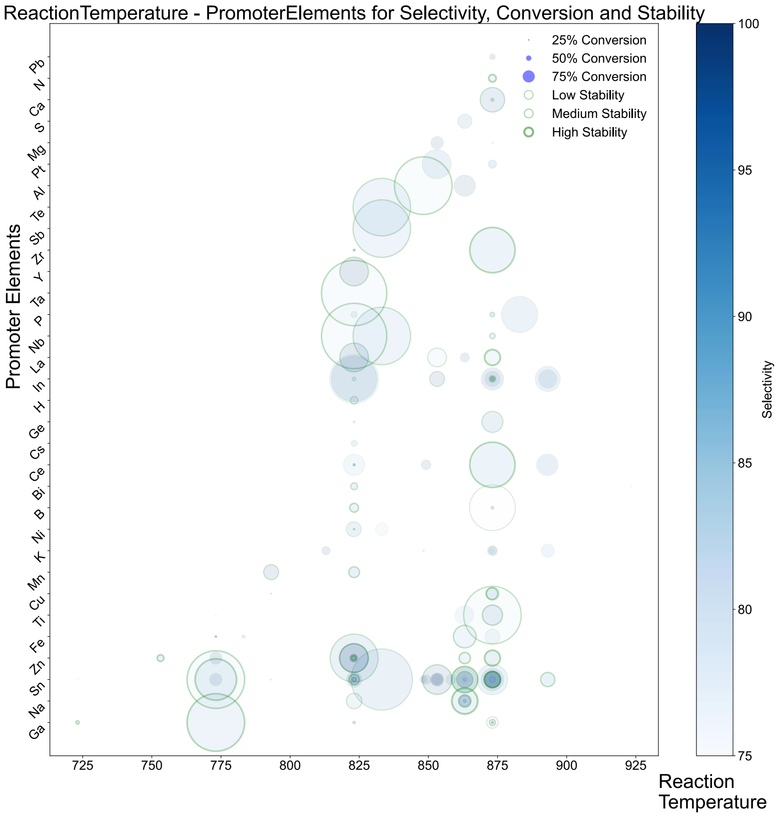

am. Reaction temperature - Active species elements

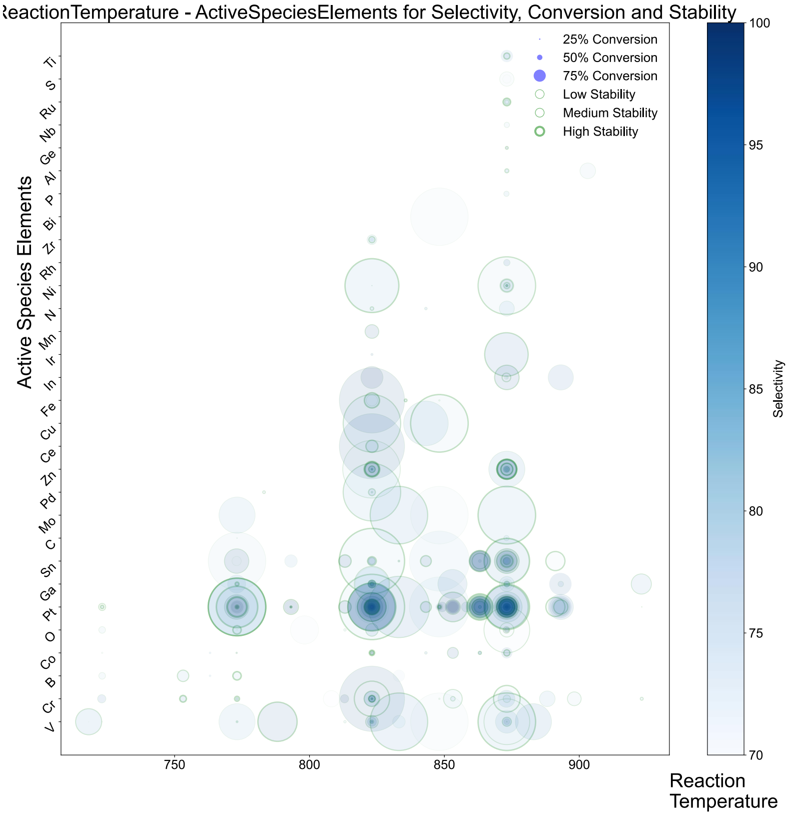

an. Propane partial pressure - Composition elements

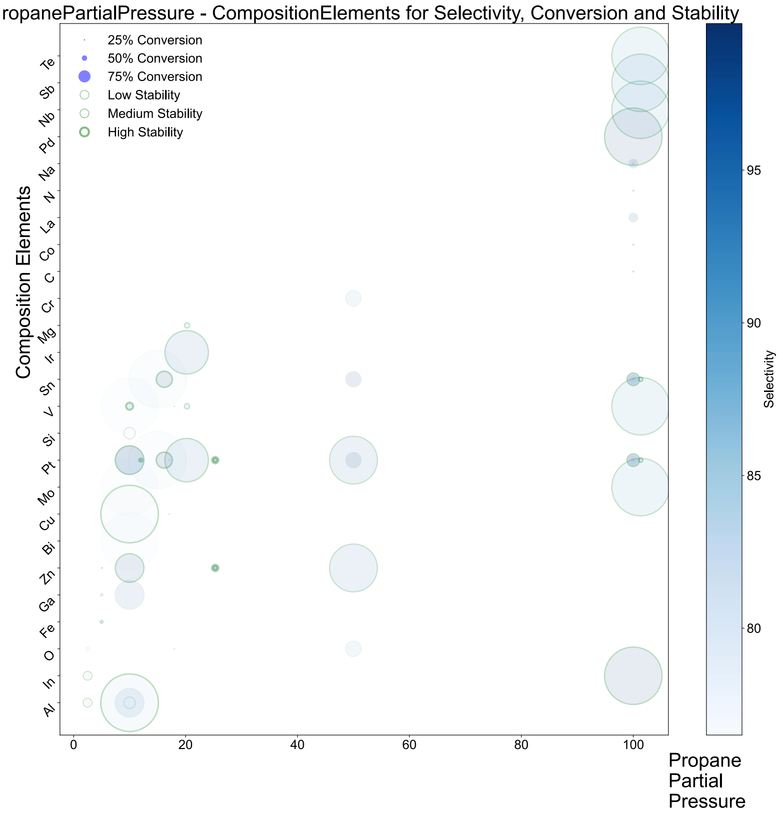

ao. Propane partial pressure - Promoter elements

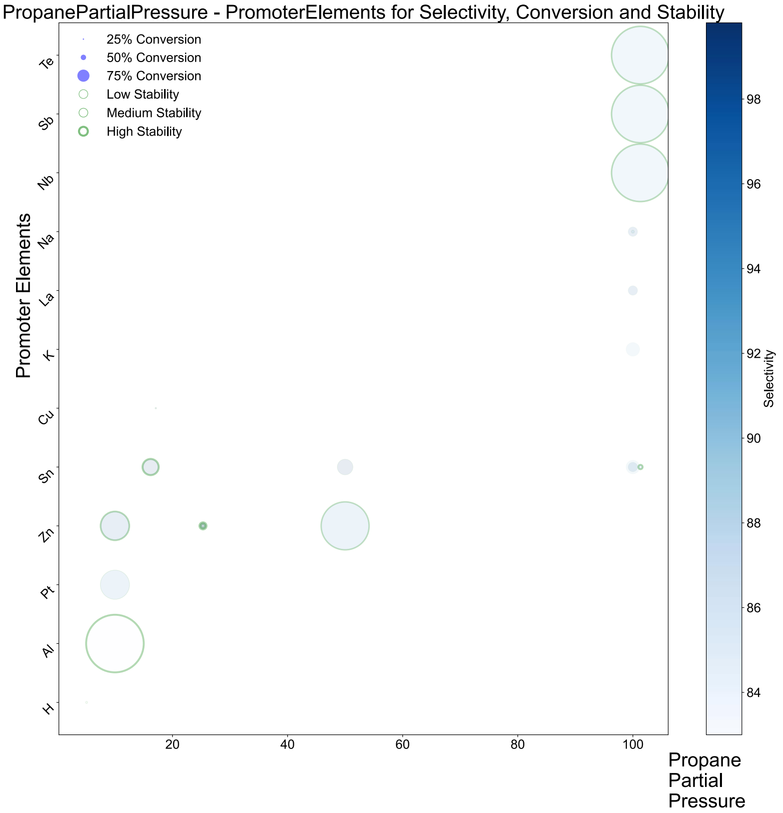

ap. Propane partial pressure - Active species elements

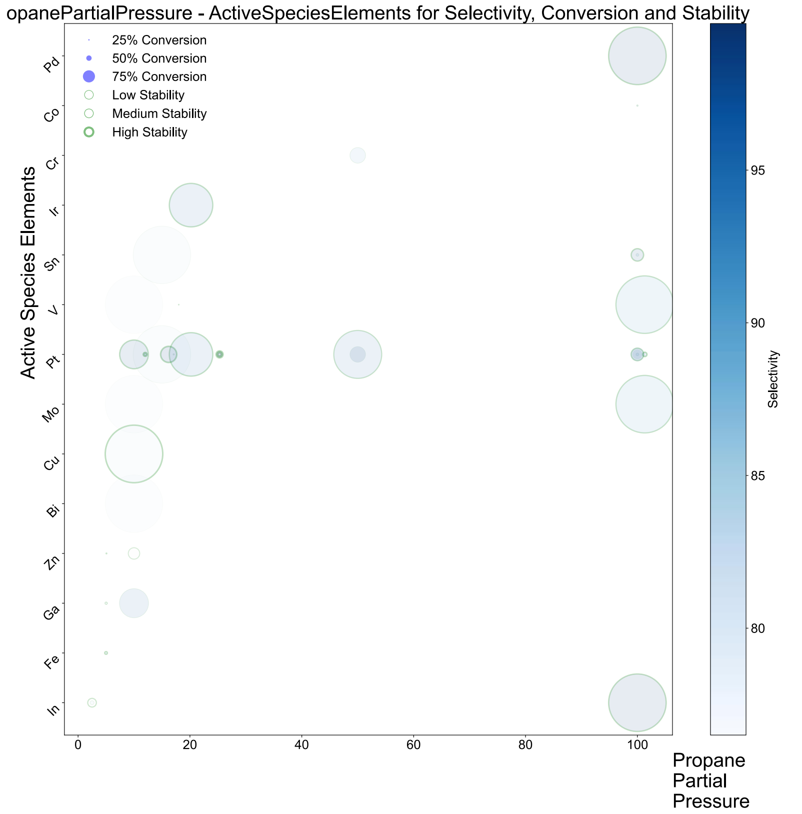

aq. Composition elements - Promoter elements

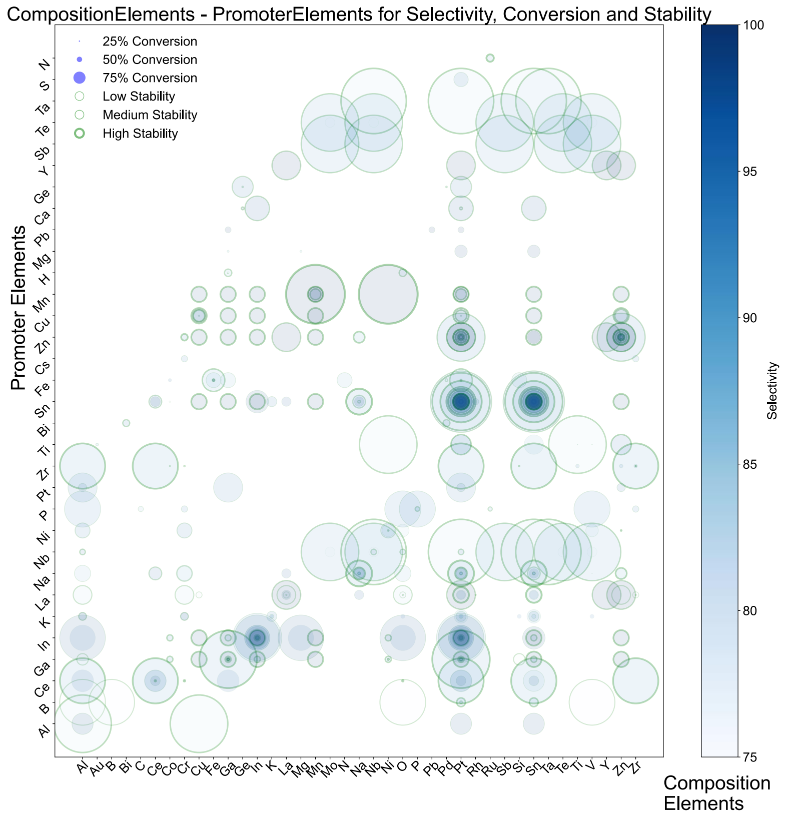

ar. Composition elements - Active species elements

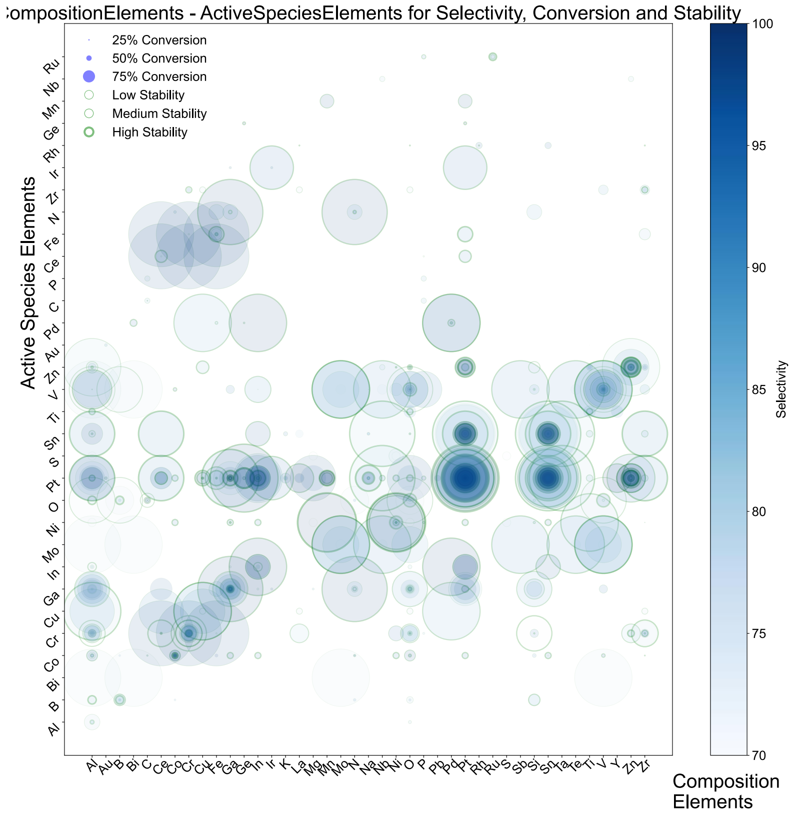

as. Promoter elements - Active species elements

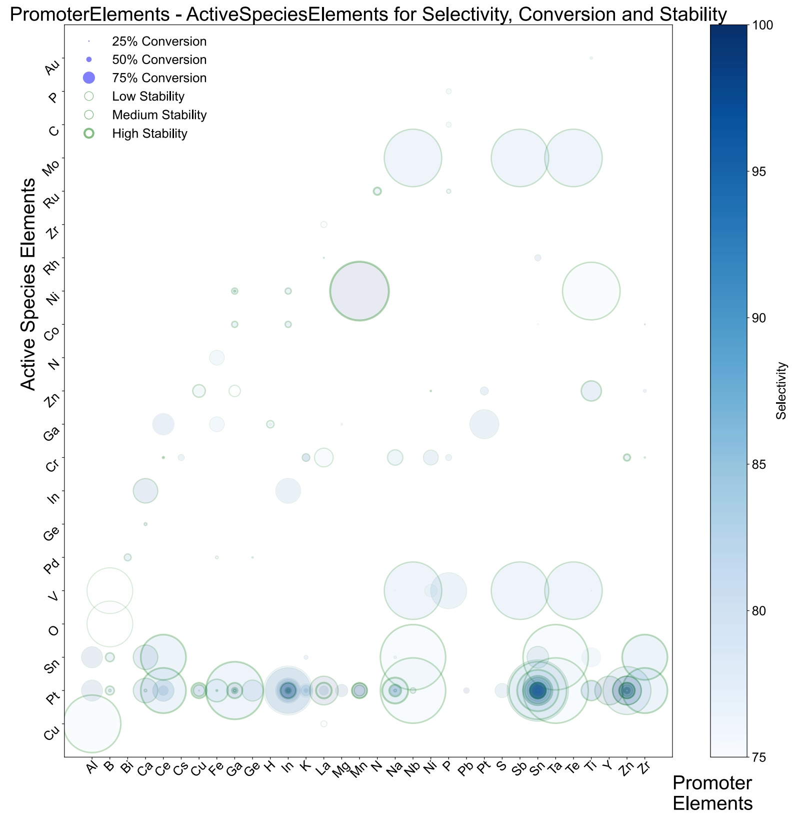

Fig.A3 Bivariate correlation bubble charts. a, Type - Structure type. **b,** Type - Support material. **c,** Type - Preparation method. **d,** Type - Inlet flow rate. **e,** Type - Reaction temperature. **f,** Type - Propane partial pressure. **g,** Type - Composition elements. **h,** Type - Promoter elements. **i,** Type - Active species elements. **j,** Structure type - Support material. **k,** Structure type - Preparation method. **l,** Structure type - Inlet flow rate. **m,** Structure type - Reaction temperature. **n,** Structure type - Propane partial pressure. **o,** Structure type - Composition elements. **p,** Structure type - Promoter elements. **q,** Structure type - Active species elements. **r,** Support material - Preparation method. **s,** Support material - Inlet flow rate. **t,** Support material - Reaction temperature. **u,** Support material - Propane partial pressure. **v,** Support material - Composition elements. **w,** Support material - Promoter elements. **x,** Support material - Active species elements. **y,** Preparation method - Inlet flow rate. **z,** Preparation method - Reaction temperature. **aa,** Preparation method - Propane partial pressure. **ab,** Preparation method - Composition elements. **ac,** Preparation method - Promoter elements. **ad,** Preparation method - Active species elements. **ae,** Inlet flow rate - Reaction temperature. **af,** Inlet flow rate - Propane partial pressure. **ag,** Inlet flow rate - Composition elements. **ah,** Inlet flow rate - Promoter elements. **ai,** Inlet flow rate - Active species elements. **aj,** Reaction temperature - Propane partial pressure. **ak,** Reaction temperature - Composition elements. **al,** Reaction temperature - Promoter elements. **am,** Reaction temperature - Active species elements. **an,** Propane partial pressure - Composition elements. **ao,** Propane partial pressure - Promoter elements. **ap,** Propane partial pressure - Active species elements. **aq,** Composition elements - Promoter elements. **ar,** Composition elements - Active species elements. **as,** Promoter elements - Active species elements. The depth of the bubble color indicates the level of selectivity, size of the bubble indicates the rate of conversion, and thickness of the bubble edge indicates the level of stability. The optimal catalyst should exhibit high selectivity, high conversion, and high stability, i.e., deep color, large bubble, and thick border.

Appendix III：Software Interface

The software interface is designed with simplicity and efficiency at its core to support rapid completion of review generation tasks. Implemented in Python3, the graphical user interface (GUI) is compatible with Windows systems and facilitates straightforward keyboard and mouse interactions. The interface allows users to visually observe the segmented processing workflow and track the generation progress in real time. Additionally, the software features a breakpoint resumption function, enhancing task processing flexibility.

Due to regulatory and legal requirements, users are required to provide their own search engine and LLM API keys. The software interface is meticulously designed to ensure efficient use of APIs while safeguarding user privacy and security. Sensitive parameters are displayed as quantities only, while non-sensitive parameters are shown in real-time.

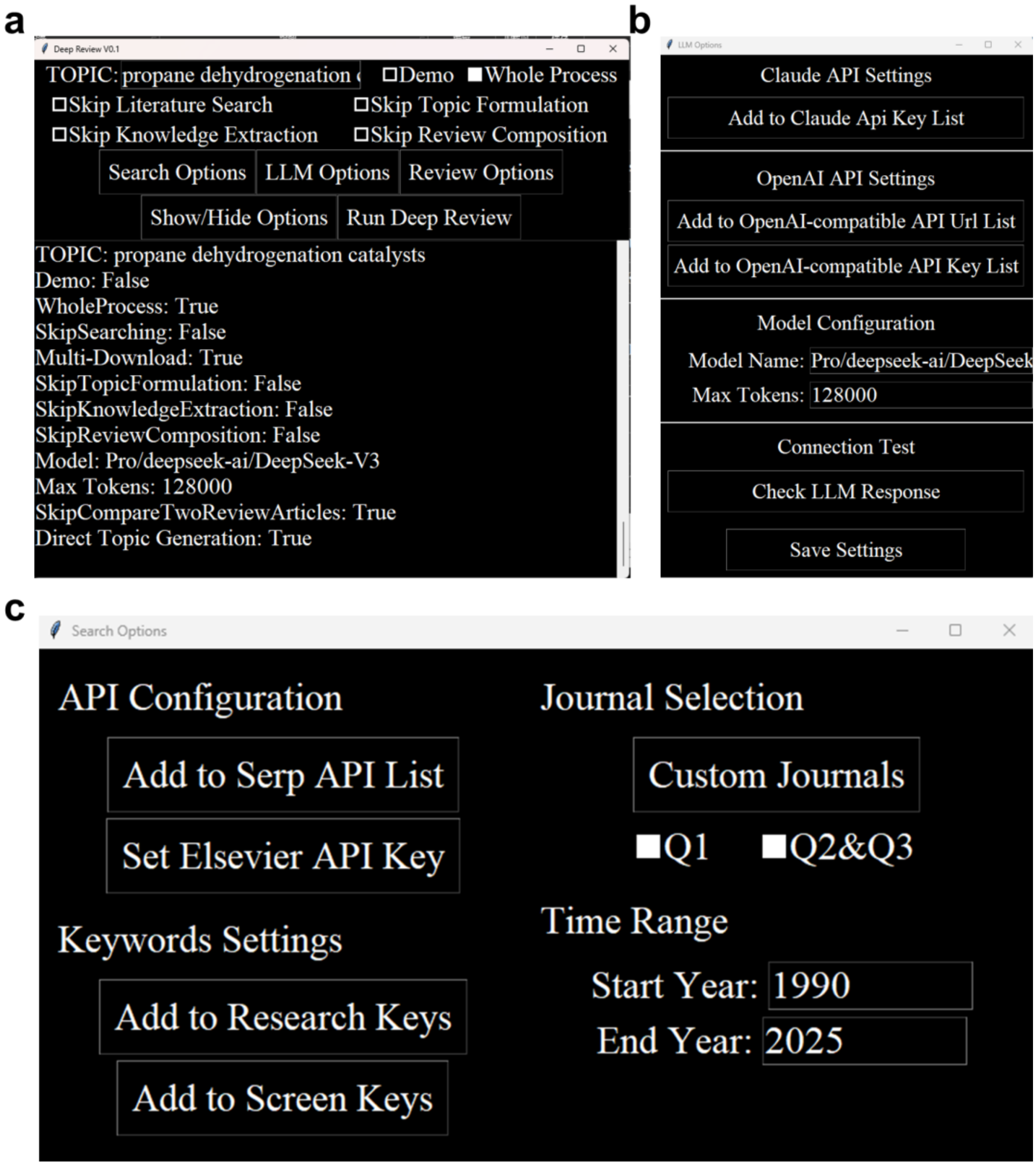

Fig.A4 Graphical user interface. a, Startup screen. **b,** Collapsible literature retrieval options interface.

The software's literature retrieval options support various parameter configurations, including search start and end years, Chinese Academy of Sciences (CAS) division classification (first division/second and third divisions), and keywords for literature search and preliminary filtering. To prevent accidental operations, the software includes a function to clear lists by entering a special character sequence. You can also type in your own defined journal name using the Custom Journals.

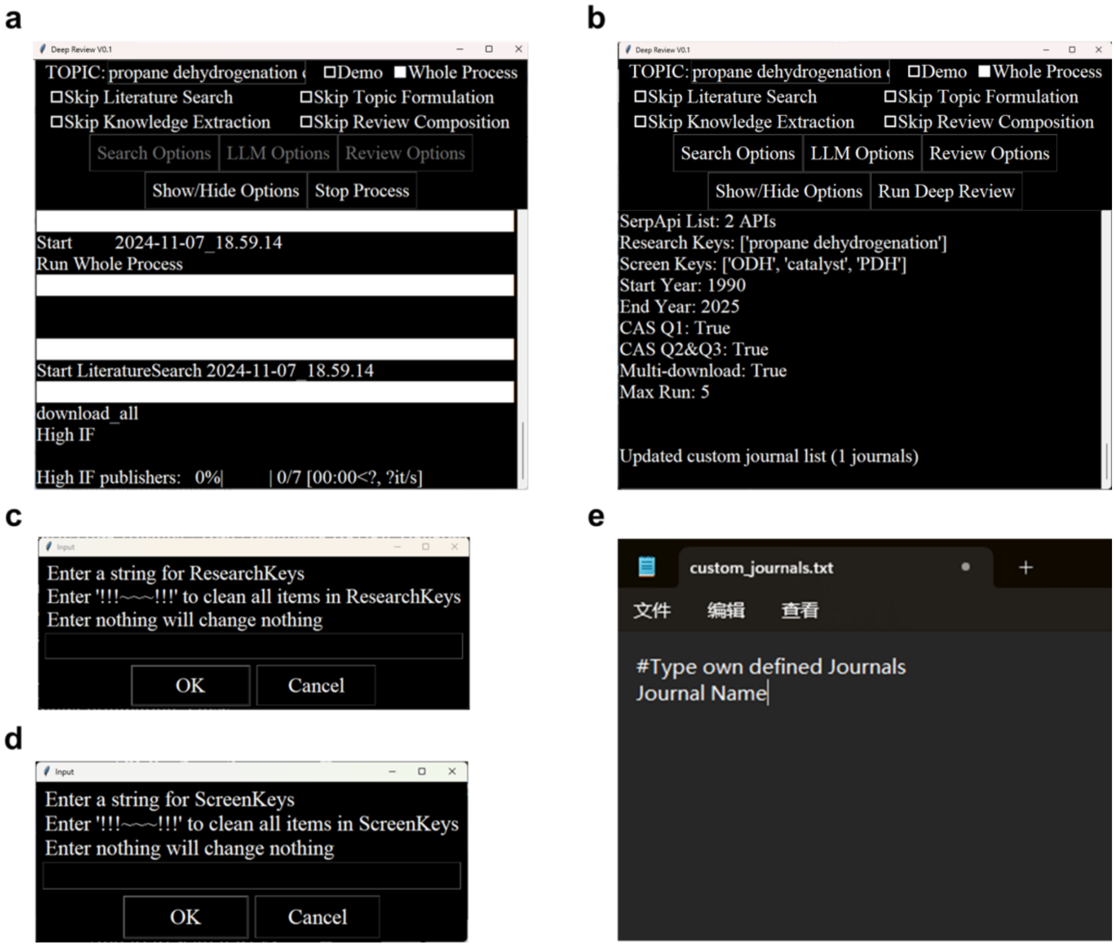

Fig.A5 Literature retrieval options interface. a, Real-time results output. **b,** Research interface output. **c,d,** Literature search options pop-up interface, window. **e,** Custom defined journal pop-up interface.

In terms of LLM options, the software supports Claude's native API and OpenAI formatted APIs, including locally deployed systems like FastChat-based ones. Users can easily test LLM connection status, and the software assumes by default that all LLMs are accessible.

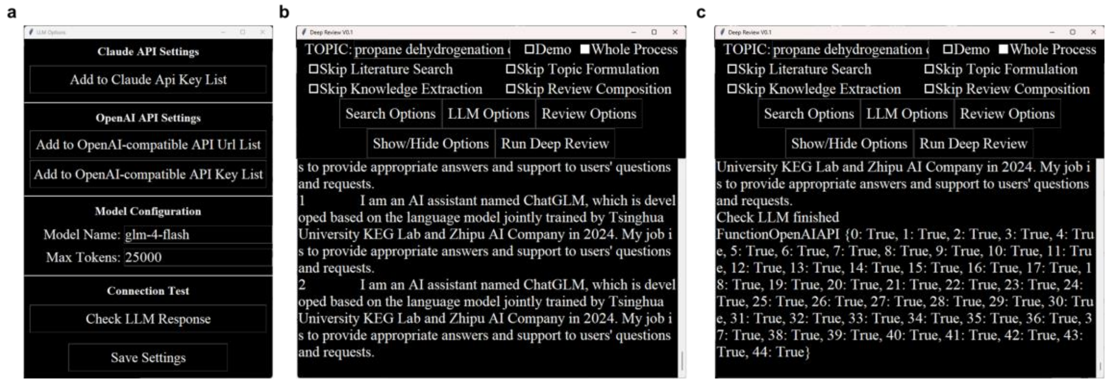

Fig.A6 LLM options interface. a, LLM options window. **b,** Configuration and successful connection test example. **c,** Final results of the connection test.

At the same time, you can set the options for review evaluation and generation and choose the method for generating review topics. You can choose to use directly generated review topics, as well as reduce costs and improve speed by skipping the literature comparison process.

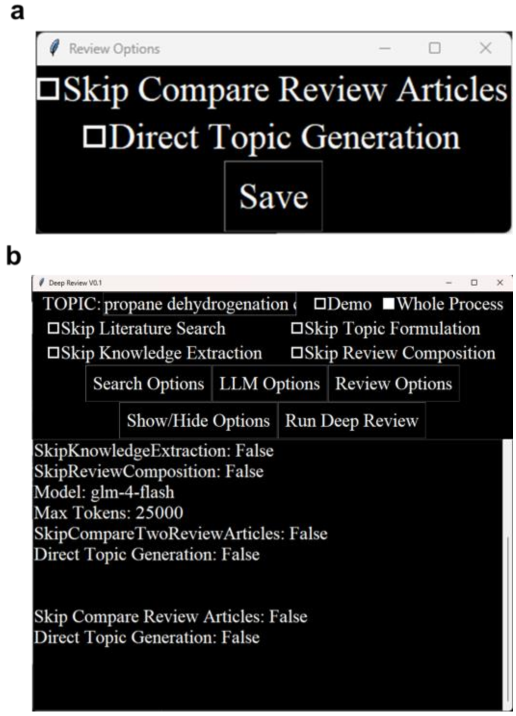

Fig.A7 Review options interface. a, Review options window. **b,** Configuration and successful connection test example. c, Final results of the connection test.

You can stop the process at any time, and this program will reload after a few seconds ago. The result will be shown in the new interface. You can drag the bar on the side of the window to view the program's history.

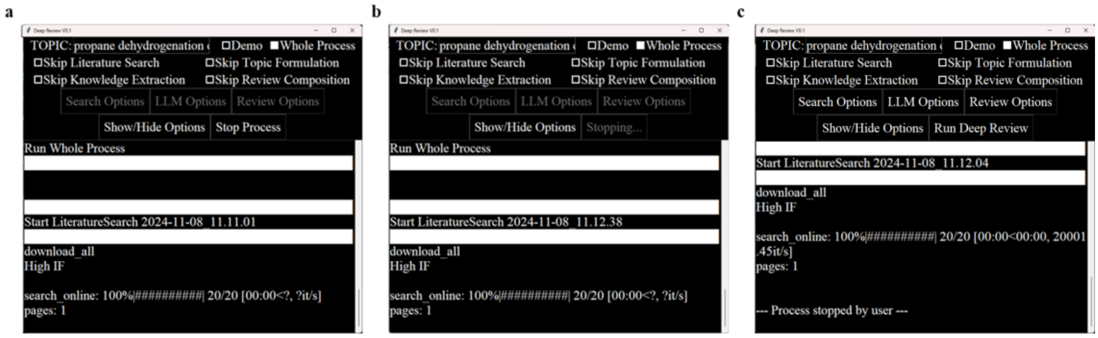

Fig.A8 Program running/stopping interface. a, Program Interrupt Button, **b,** Program Interrupting. **c,** The historical results of the program.

For user interaction, the software provides detailed error message prompts to help users quickly identify and resolve input or operational errors. These messages cover various scenarios from LLM connection failures to uncompleted prerequisite processes for each stage, ensuring smooth and effective user operation.

a:

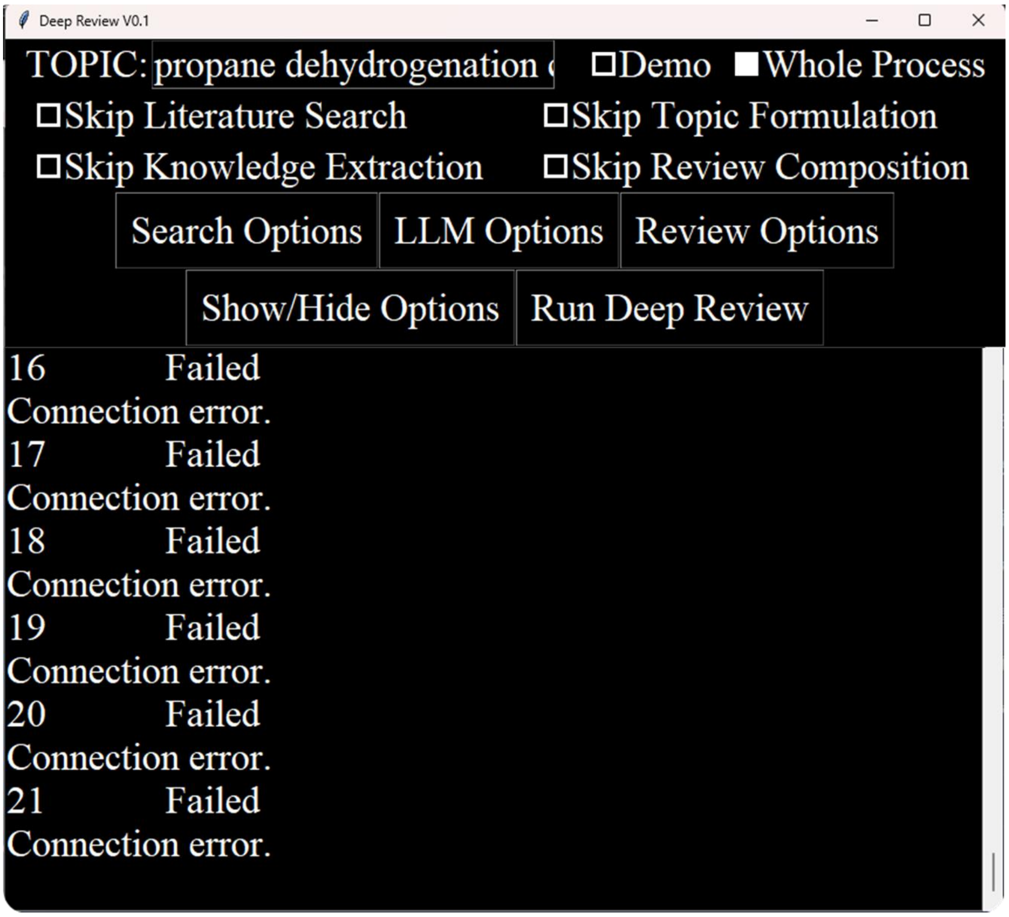

b:

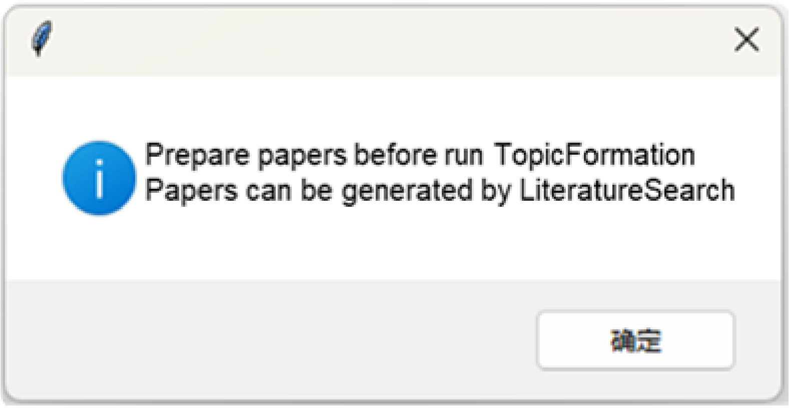

c:

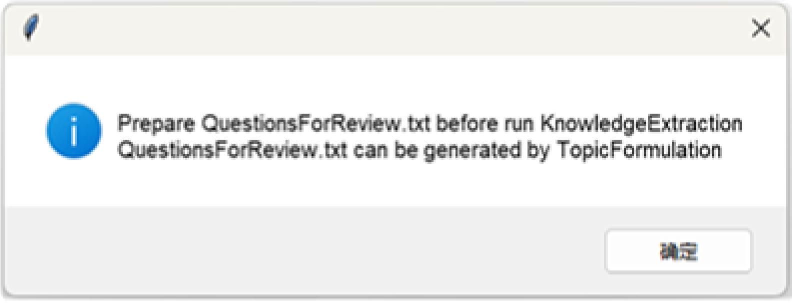

d:

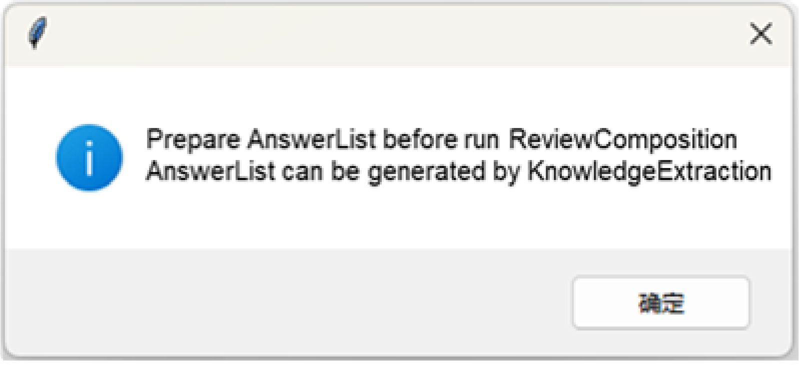

Fig.A9 Error message window. a, Example of LLM connection failure. **b,** Prompt for uncompleted literature search generation process. **c,** Prompt for uncompleted topic formulation process. **d,** Prompt for uncompleted knowledge extraction process.

Appendix IV：Manual Hallucination Verification Results

Our manual verification of hallucinations examined 50 peer-reviewed articles (25 per section). The verification results are presented in Table A2-5 (Tables.xlsx, Sheet: Table A2-5) for knowledge extraction and Table A6 (Tables.xlsx, Sheet: Table A6) for data mining applications.

Appendix V：Review Quality Assessment Scoring Criteria for LLM-Generated Scientific Reviews

Table A7 (Tables.xlsx, Sheet: Table A7) presents the scoring criteria developed for evaluating LLM-generated scientific reviews.

Appendix VI：Reliability Assessment of Qwen2-7b-Instruct

a, ICC heatmap for self-evaluation.

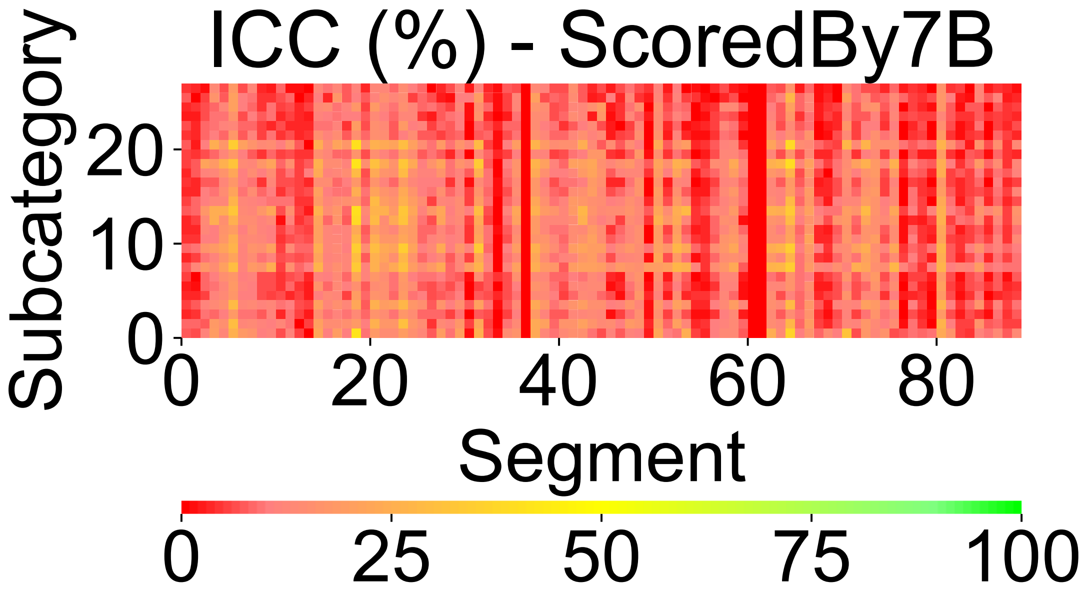

b, TCR heatmap for self-evaluation.

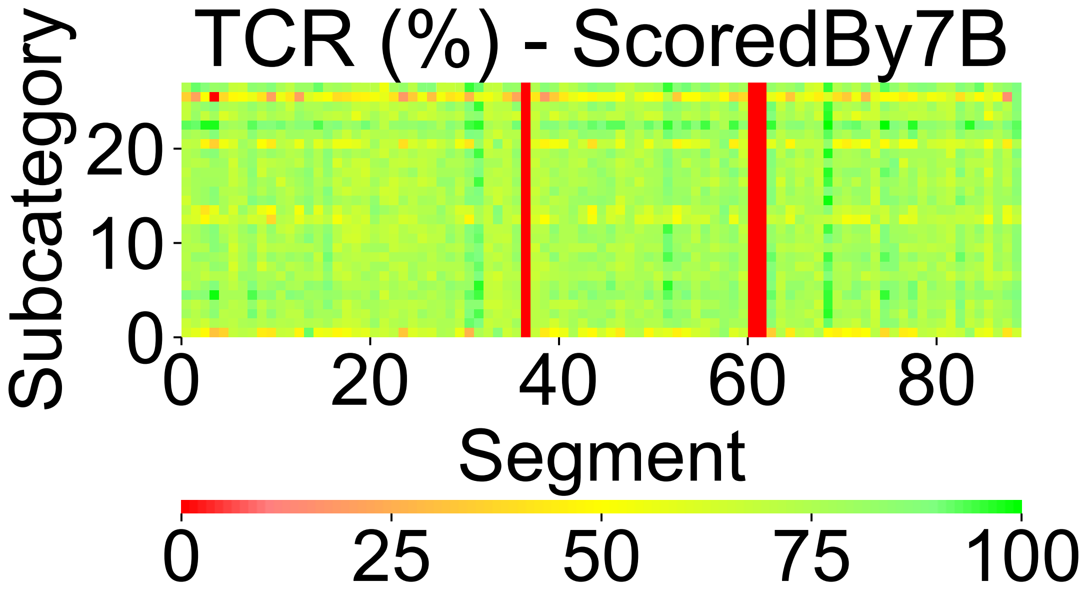

c, ICC heatmap by Qwen2-72b-Instruct.

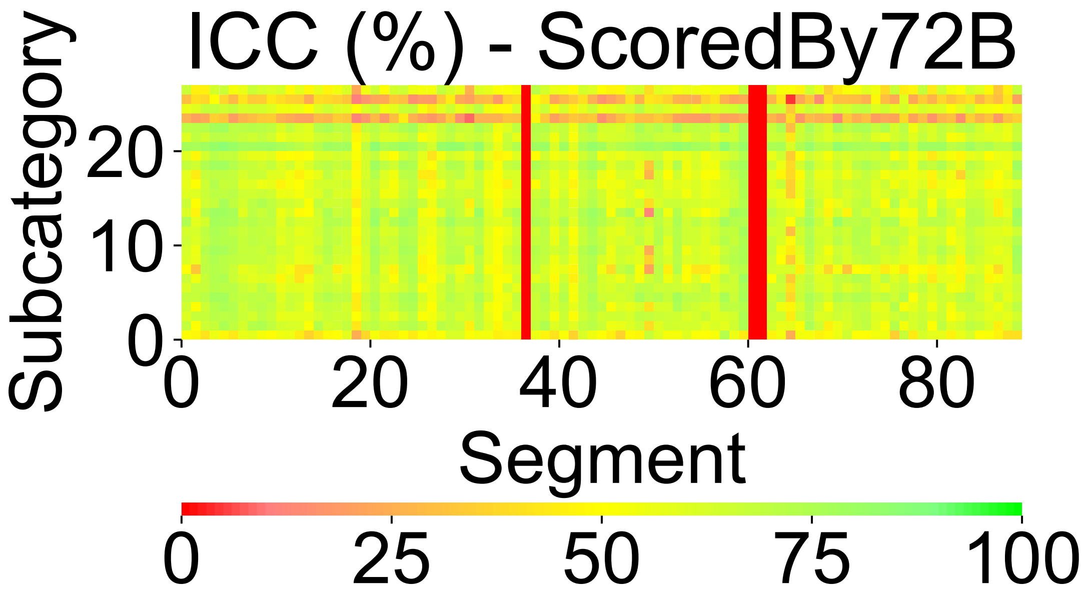

d, TCR heatmap by Qwen2-72b-Instruct.

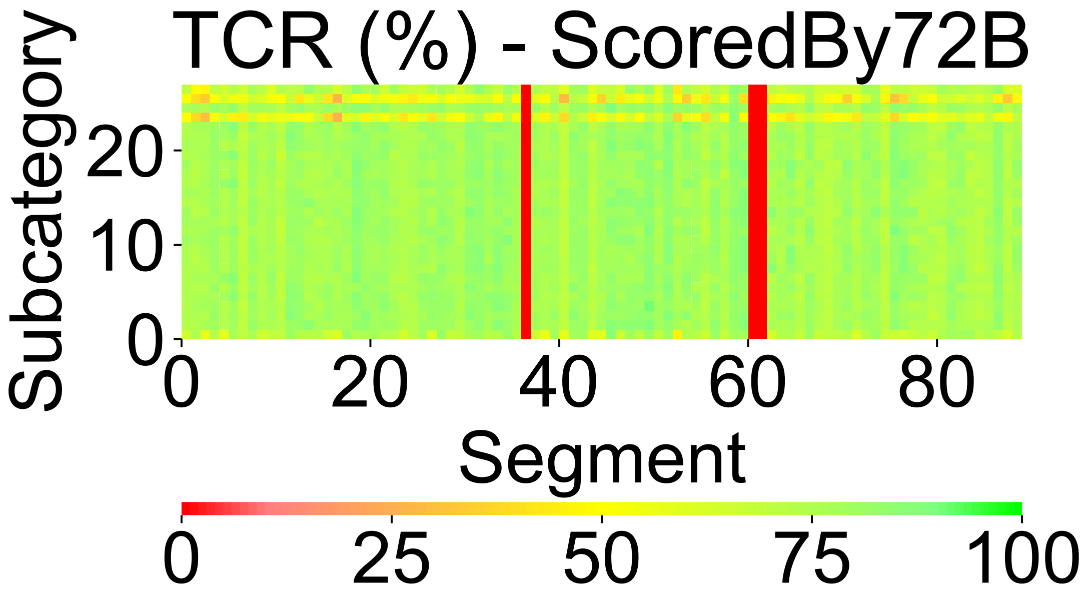

e, Histogram of TCR and ICC assessment scores for Qwen2-7b-Instruct and Qwen2-72b-Instruct models.

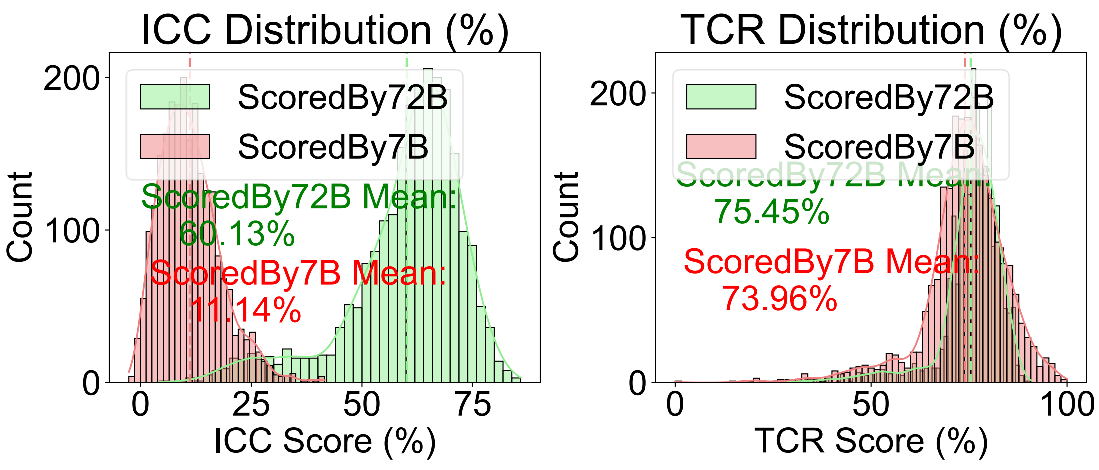

Fig.A10 Reliability assessment results of Qwen2-7b-Instruct. a, ICC heatmap for self-evaluation. **b,** TCR heatmap for self-evaluation. **c,** ICC heatmap by Qwen2-72b-Instruct. **d,** TCR heatmap by Qwen2-72b-Instruct. **e,** Histogram of TCR and ICC assessment scores for Qwen2-7b-Instruct and Qwen2-72b-Instruct models.

Appendix VII：Comprehensive Model Evaluation Results

The normalized self-assessment scores for Claude3.5Sonnet, Qwen2-72b-Instruct, and Qwen2-7b-Instruct are presented in Tables A8, 9, and 10, respectively (Tables.xlsx, Sheets: Table A8-10). For cross-model evaluation, Tables A11 and 12 (Tables.xlsx, Sheets: Table A11-12) contain the normalized assessment scores of Claude3.5Sonnet and Qwen2-7b-Instruct as evaluated by Qwen2-72b-Instruct.

a, Scaling law analysis comparing performance between Qwen2-7b and Qwen2-72b models.

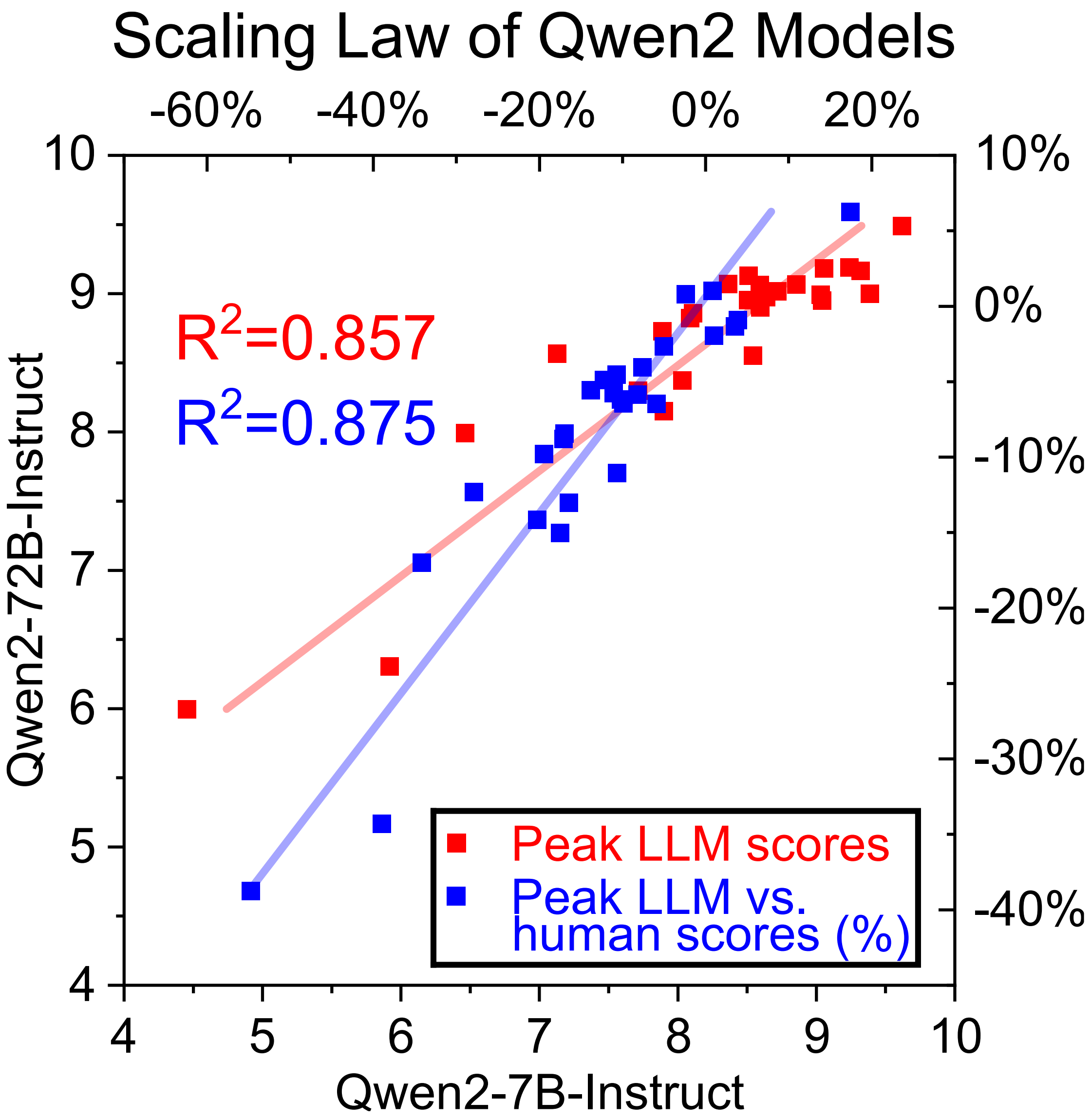

b, Three-dimensional visualization comparing performance metrics across different LLM models.

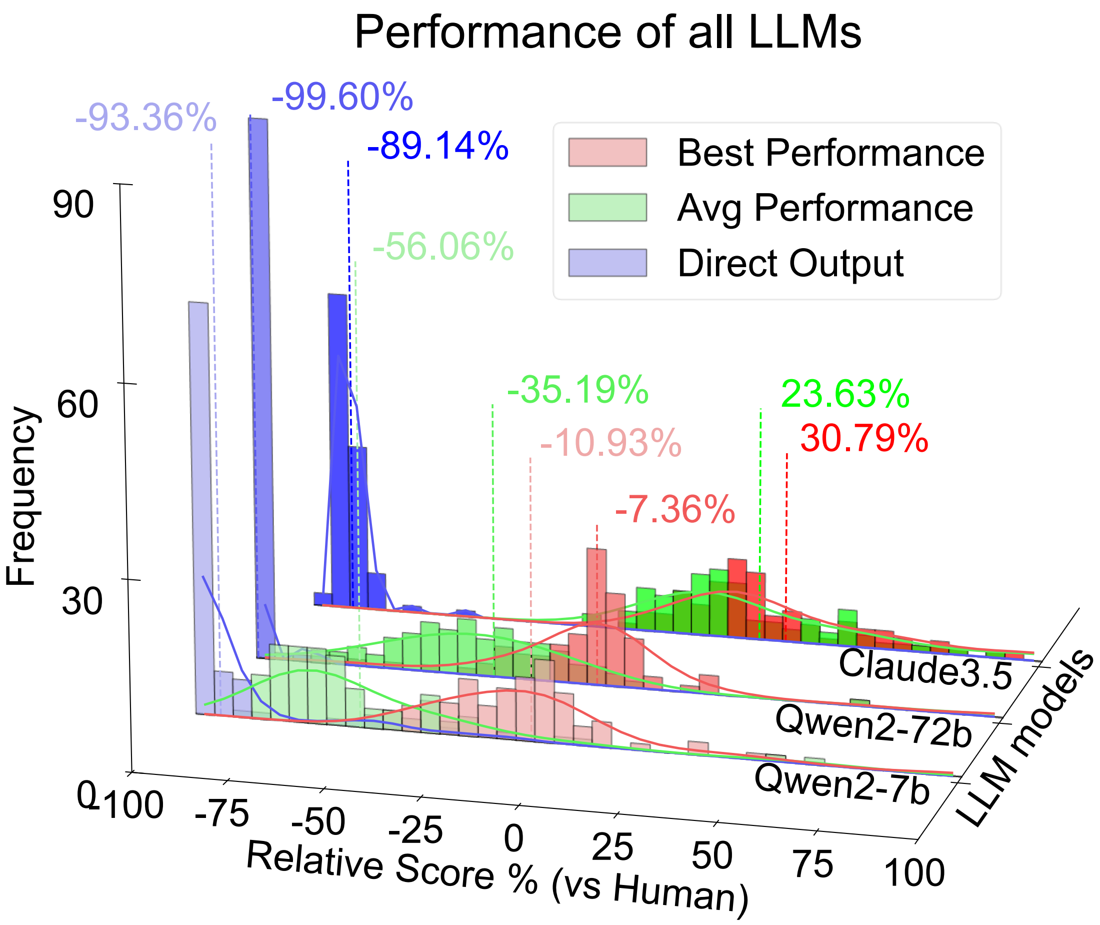

c, Distribution analysis of relative scores across models.

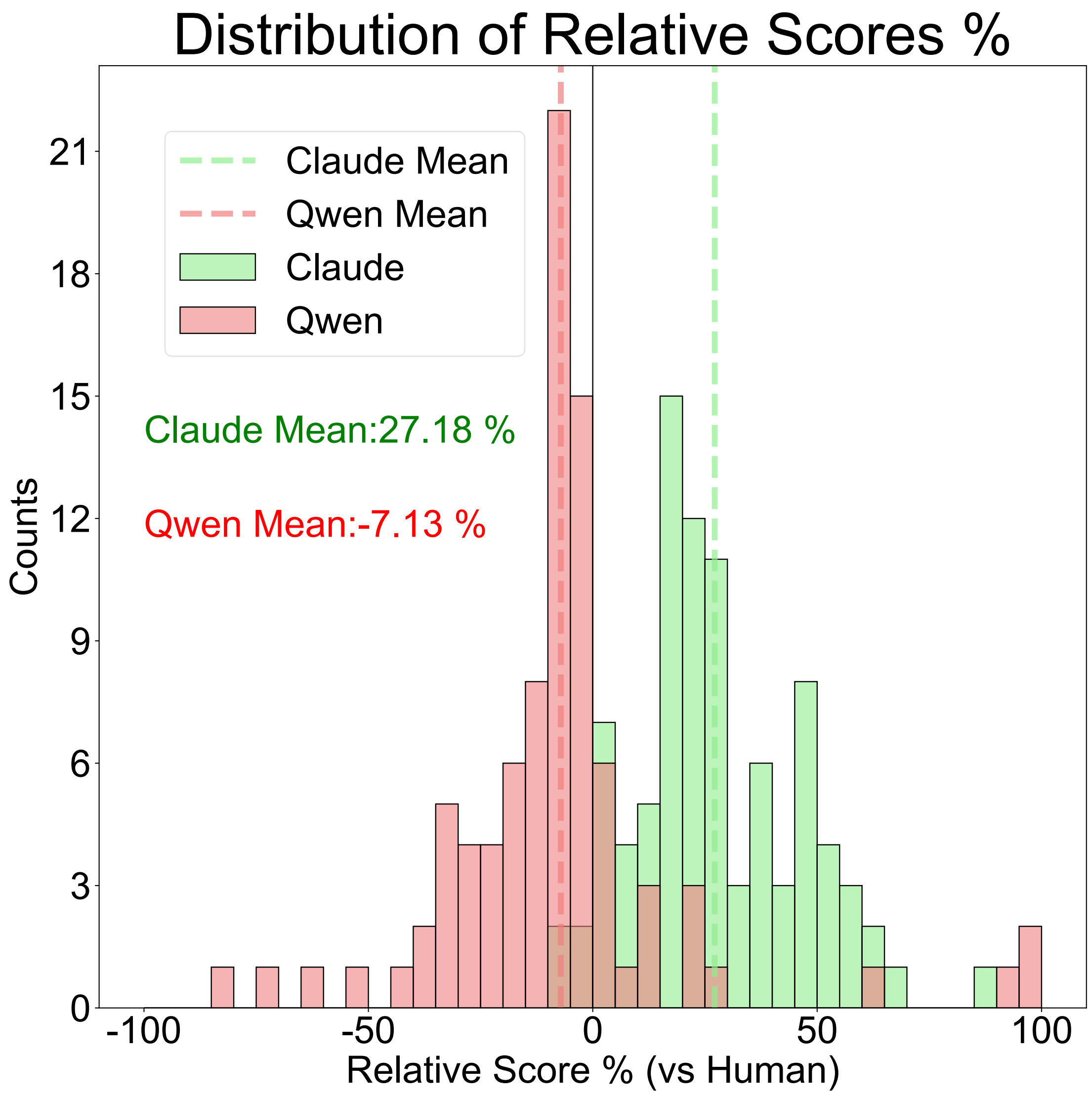

d, Histogram comparison of reliability between Claude3.5Sonnet and Qwen2-72b-Instruct.

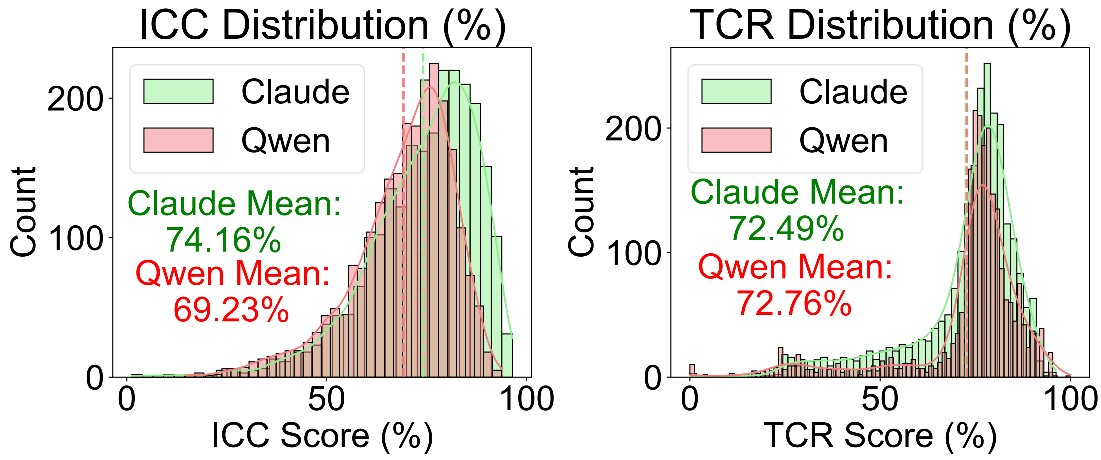

e, Comparative distribution analysis of maximum scores achieved by each model.

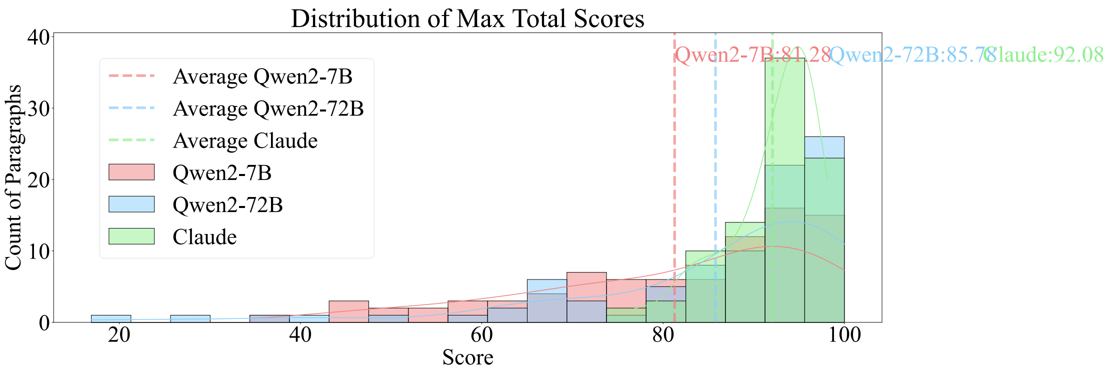

f, Total maximum score comparison across all models.

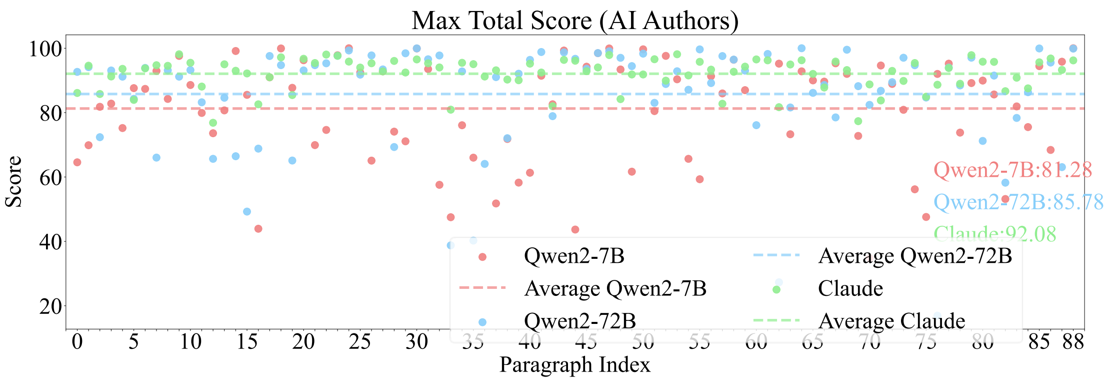

g, Comparative analysis of model scores relative to human performance.

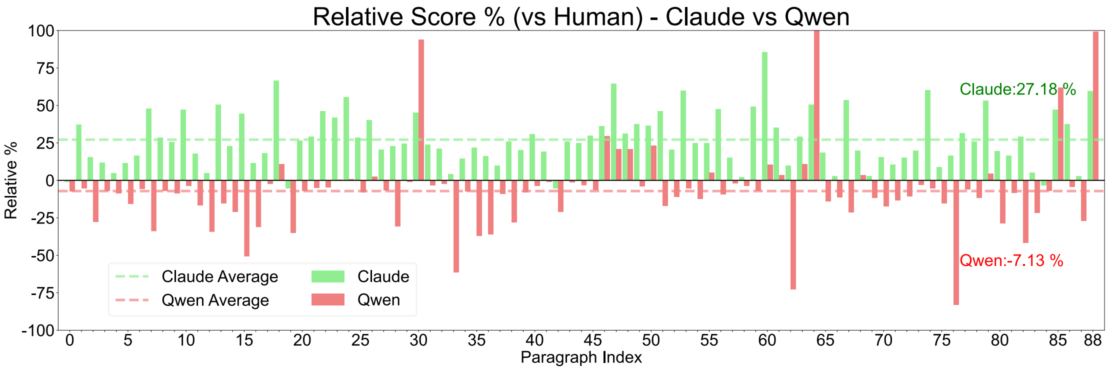

h, Intraclass correlation coefficient heatmap for Claude3.5Sonnet.

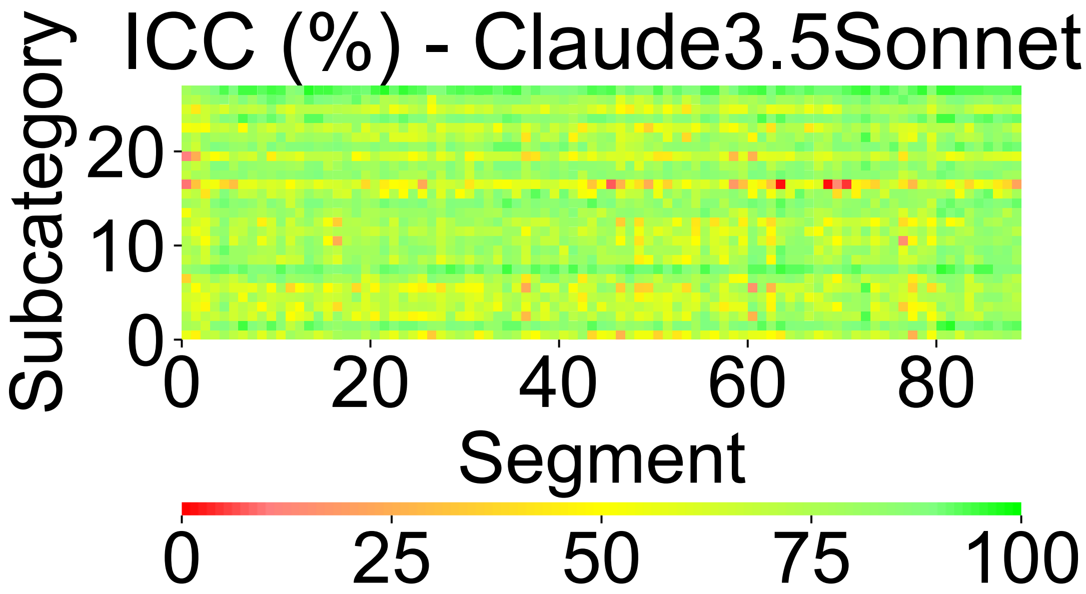

i, Transitive consistency ratio heatmap for Claude3.5Sonnet.

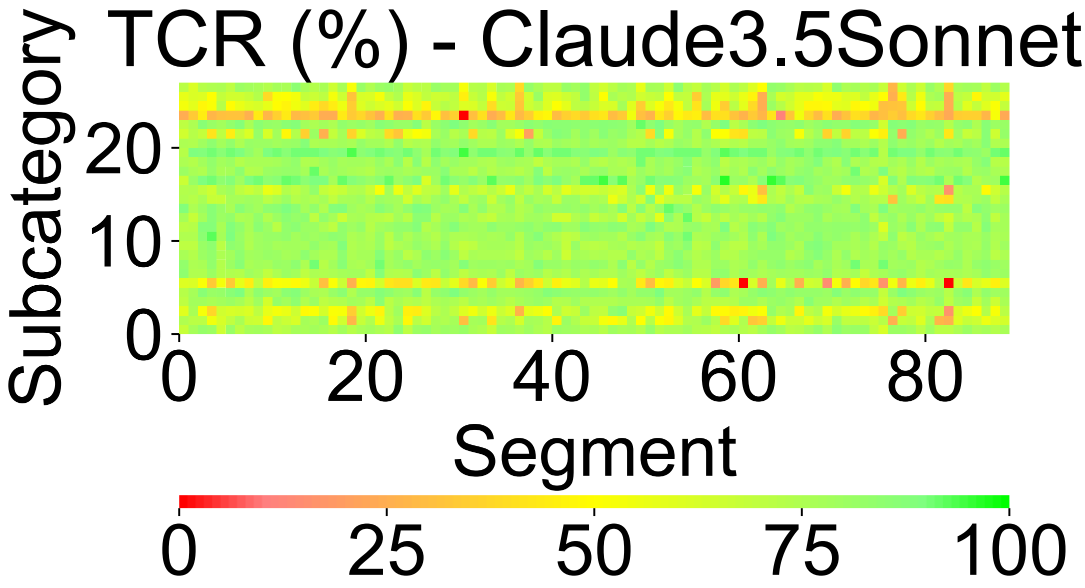

j, Average performance score heatmap for Claude3.5Sonnet.

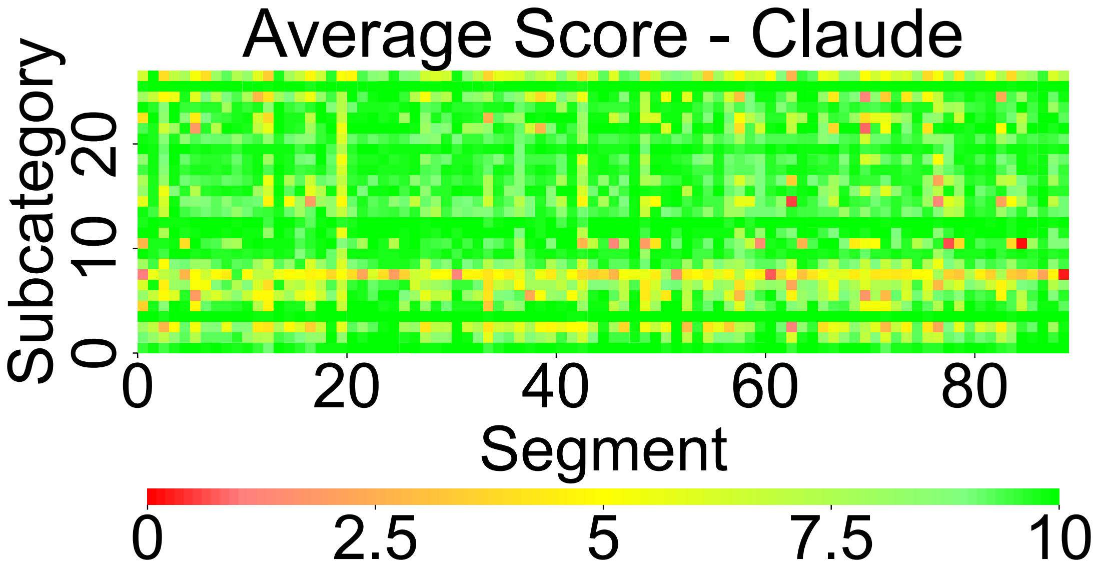

k, Peak performance score heatmap for Claude3.5Sonnet.

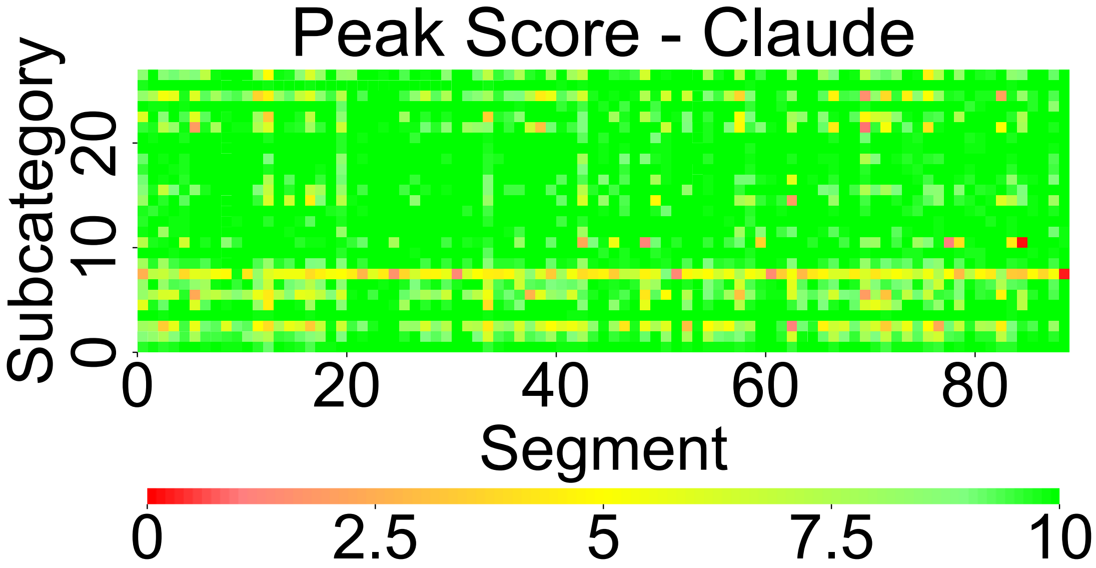

l, Total score distribution heatmap for Claude3.5Sonnet.

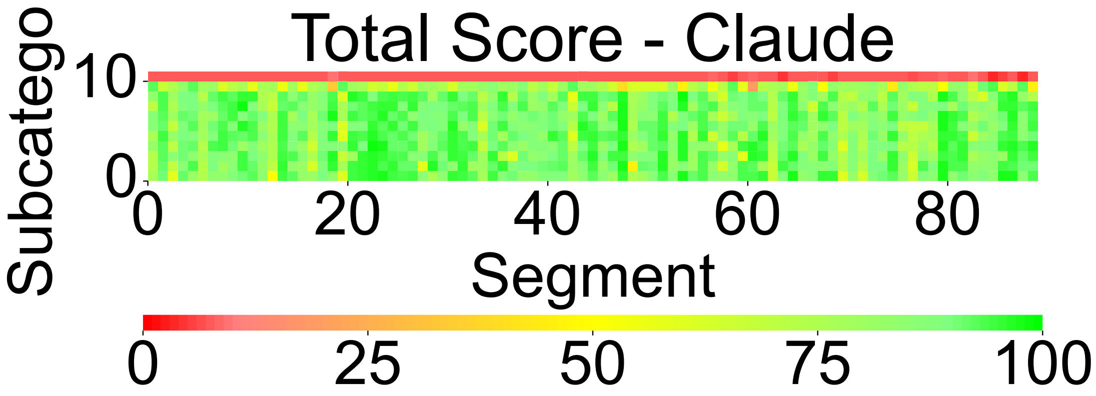

m, Percentage mean difference heatmap comparing Claude3.5Sonnet with baseline.

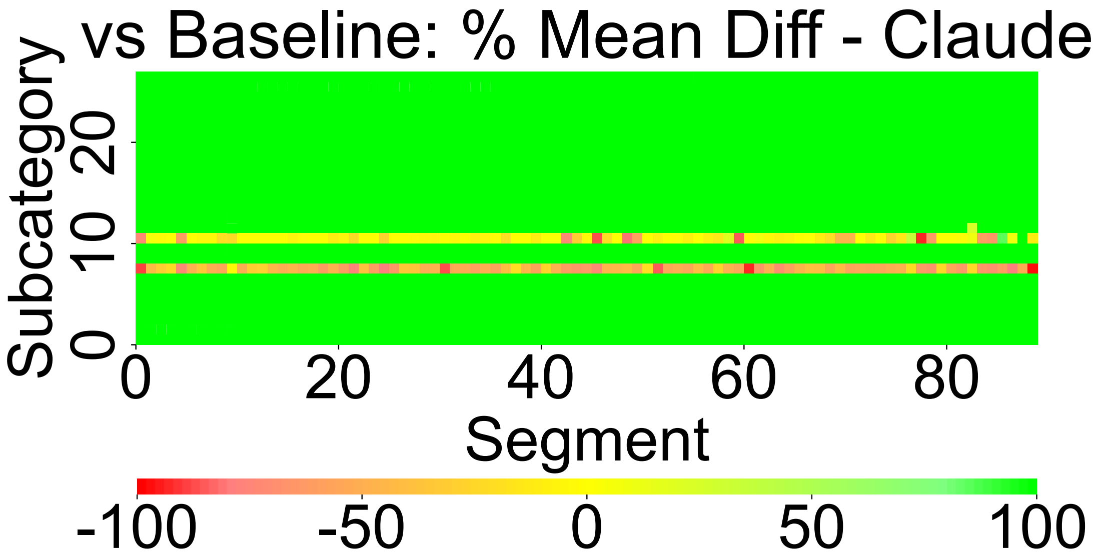

n, Percentage peak difference heatmap comparing Claude3.5Sonnet with baseline.

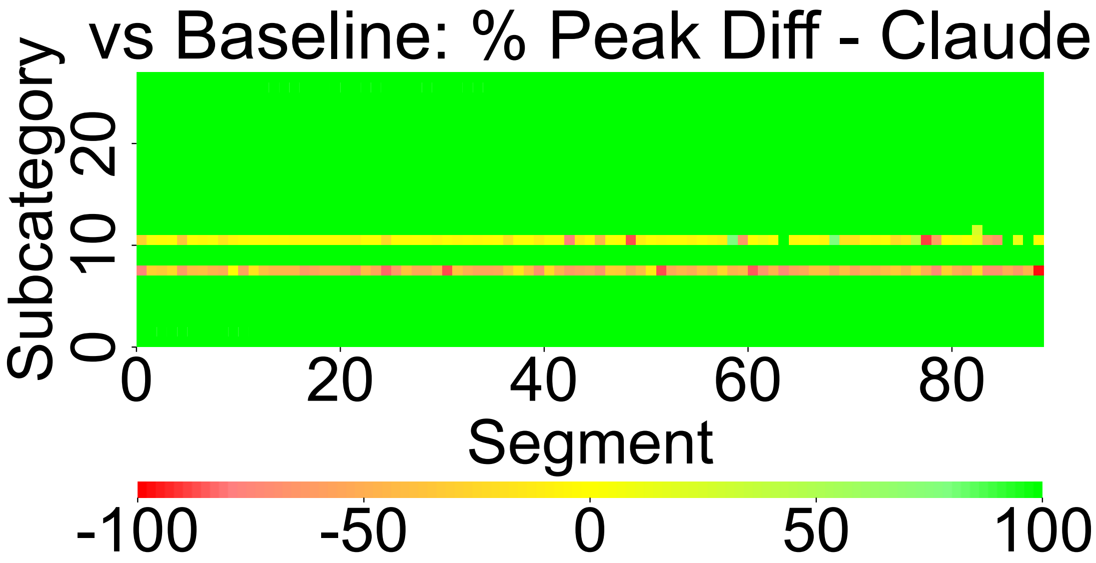

o, Percentage mean difference heatmap comparing Claude3.5Sonnet with human performance.

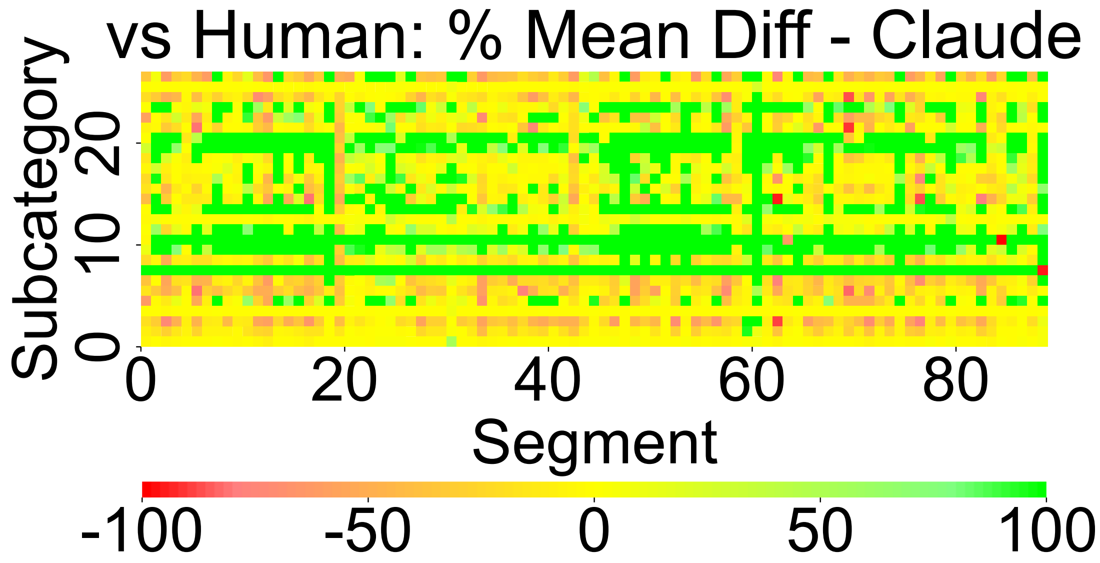

p, Percentage peak difference heatmap comparing Claude3.5Sonnet with human performance.

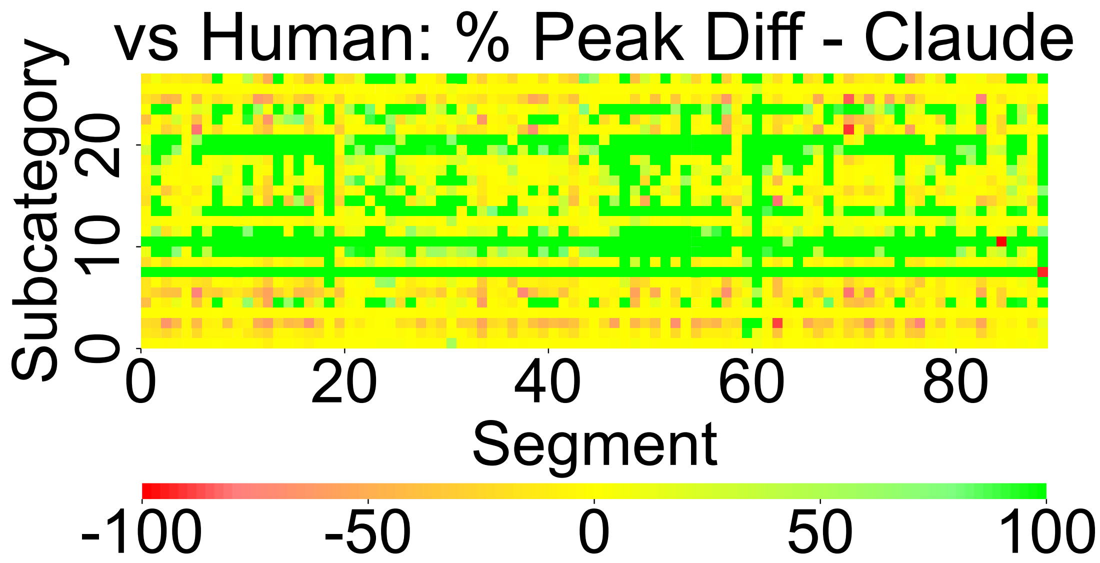

q, Intraclass correlation coefficient heatmap for Qwen2-72B.

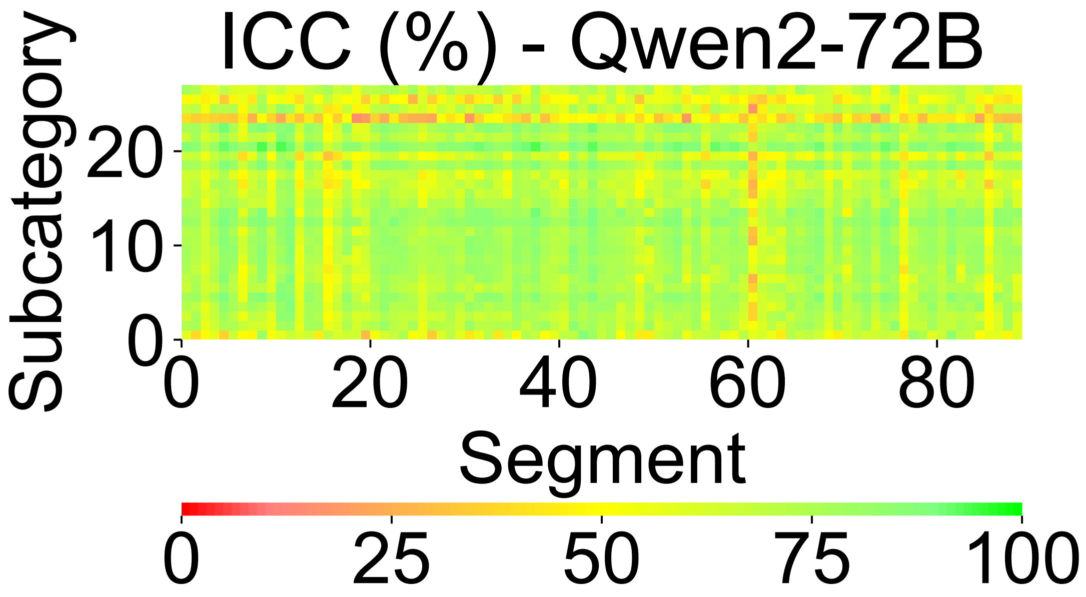

r, Transitive consistency ratio heatmap for Qwen2-72B.

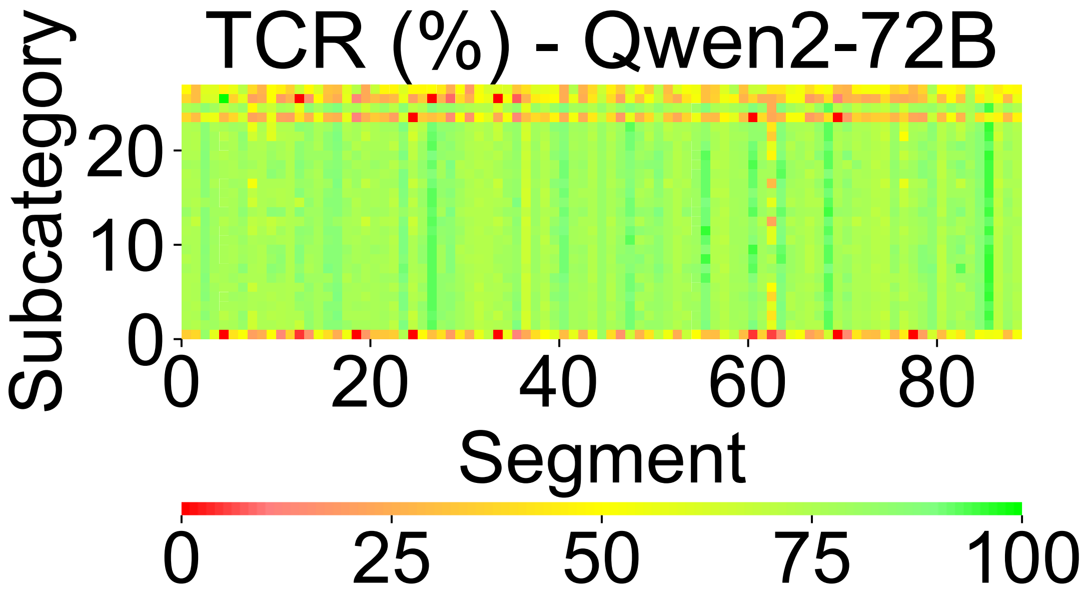

s, Average performance score heatmap for Qwen2-72B.

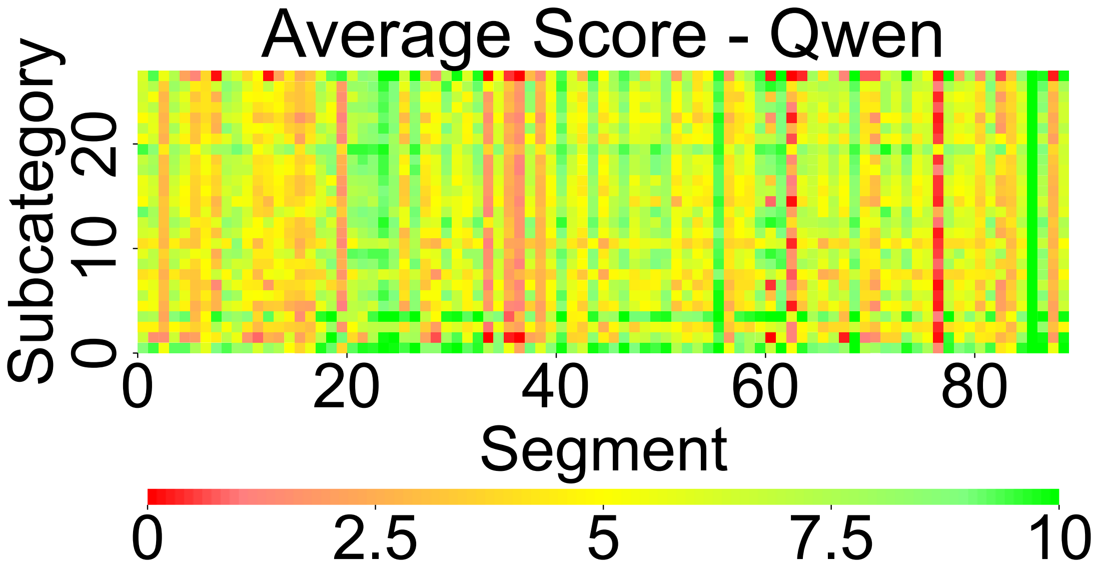

t, Peak performance score heatmap for Qwen2-72B.

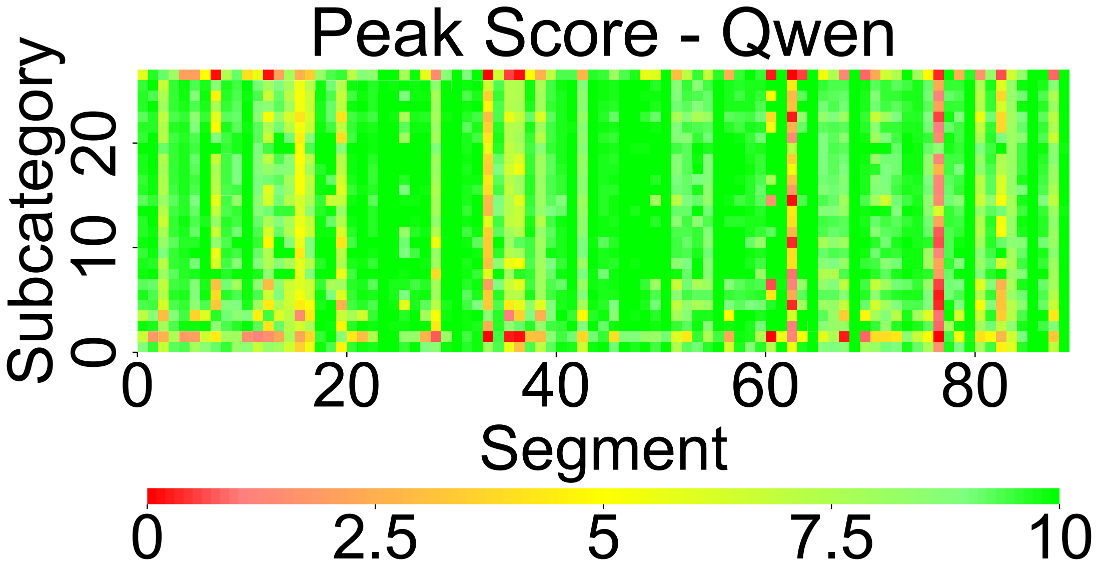

u, Total score distribution heatmap for Qwen2-72B.

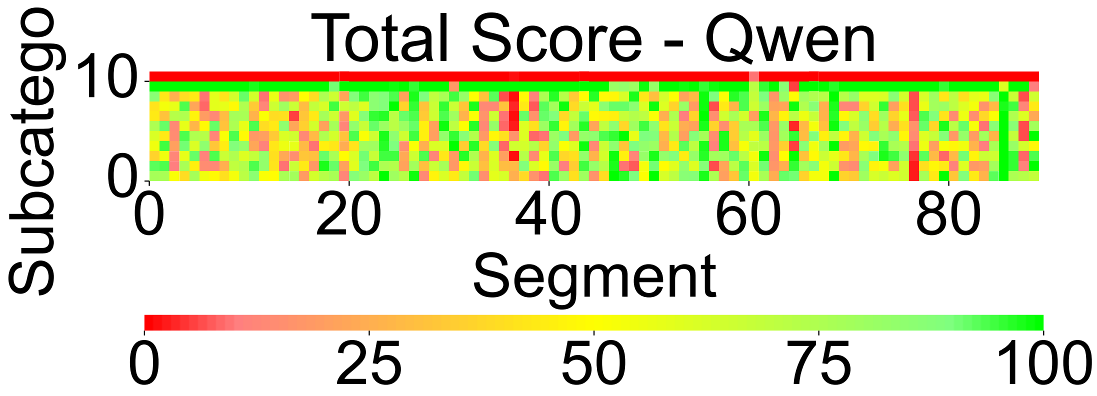

v, Percentage mean difference heatmap comparing Qwen2-72B with baseline.

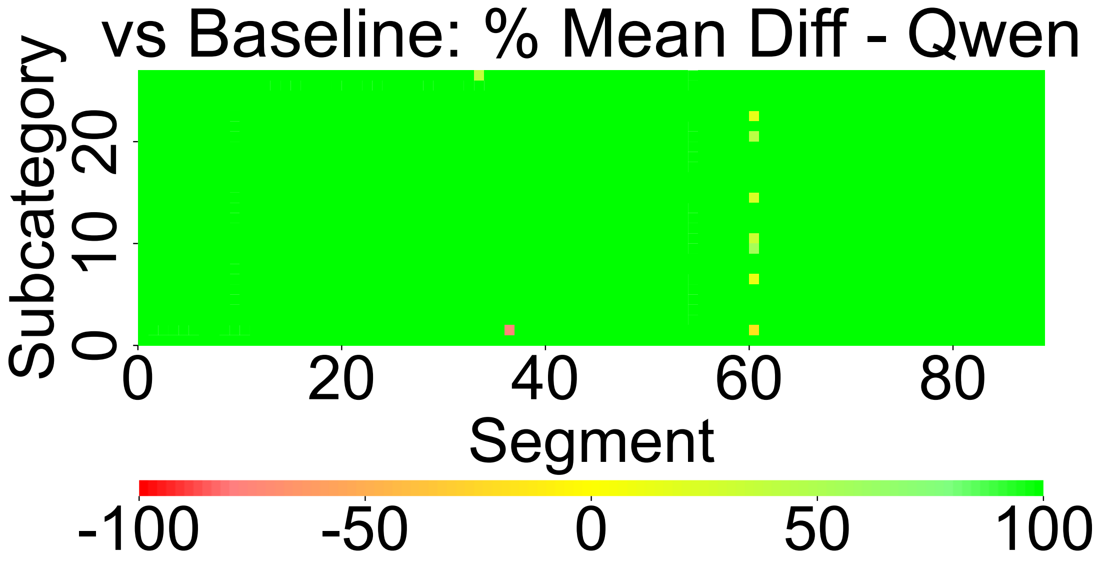

w, Percentage peak difference heatmap comparing Qwen2-72B with baseline.

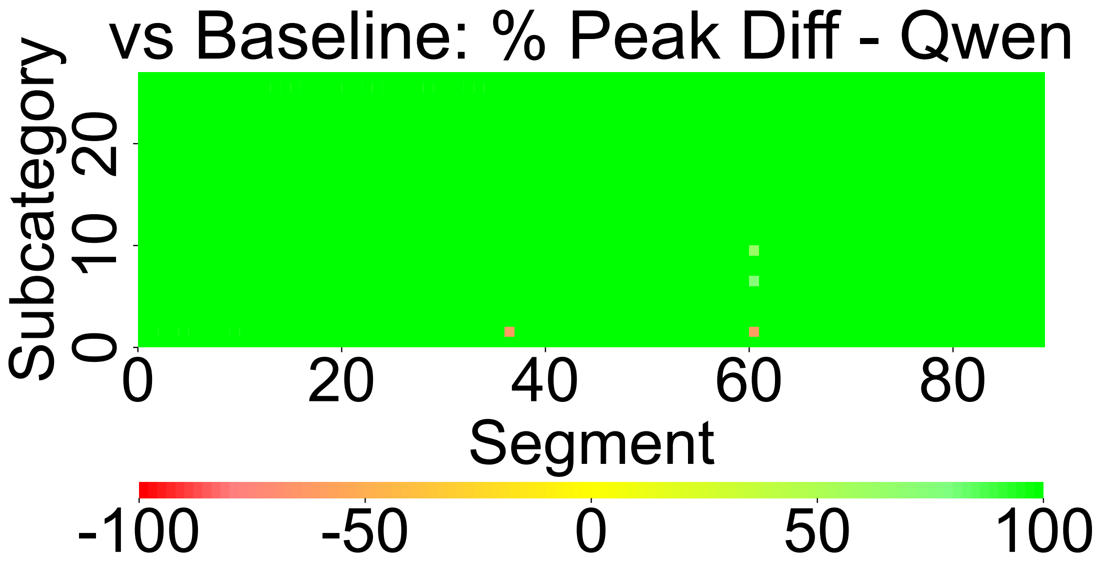

x, Percentage mean difference heatmap comparing Qwen2-72B with human performance.

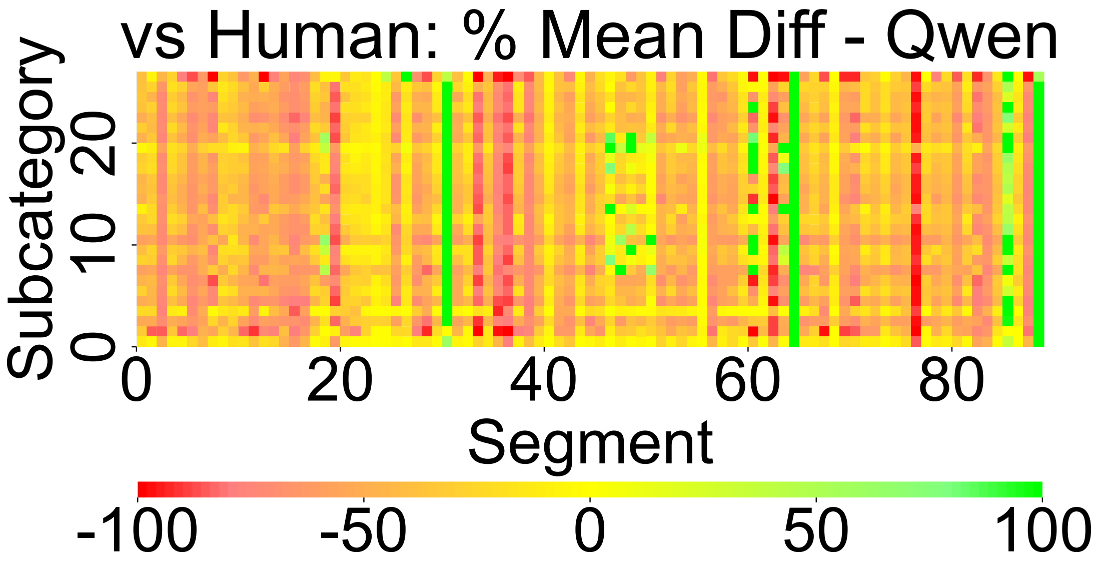

y, Percentage peak difference heatmap comparing Qwen2-72B with human performance.

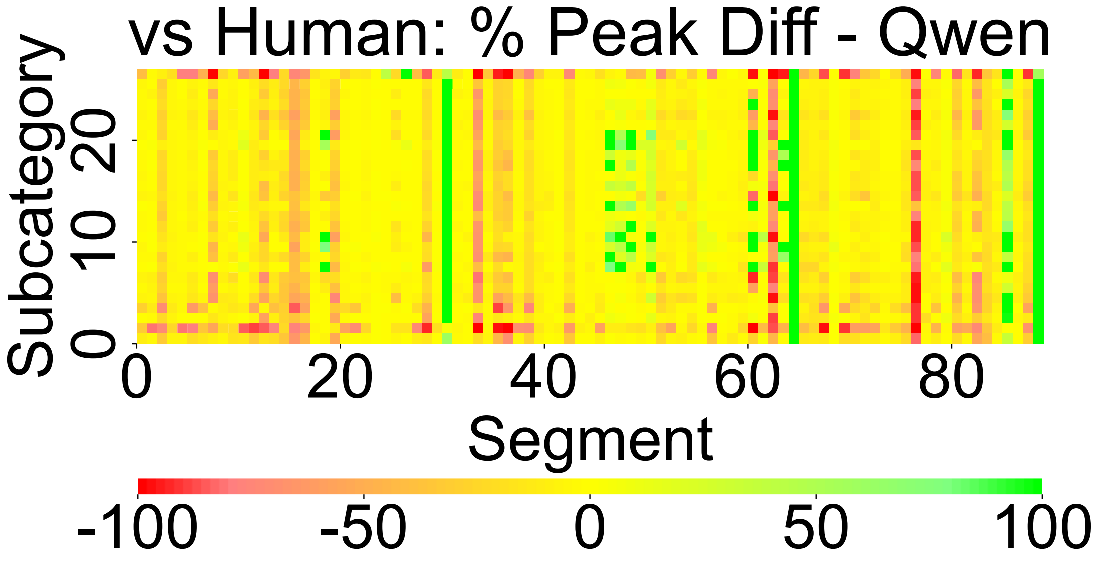

z, Intraclass correlation coefficient heatmap for Qwen2-7B.

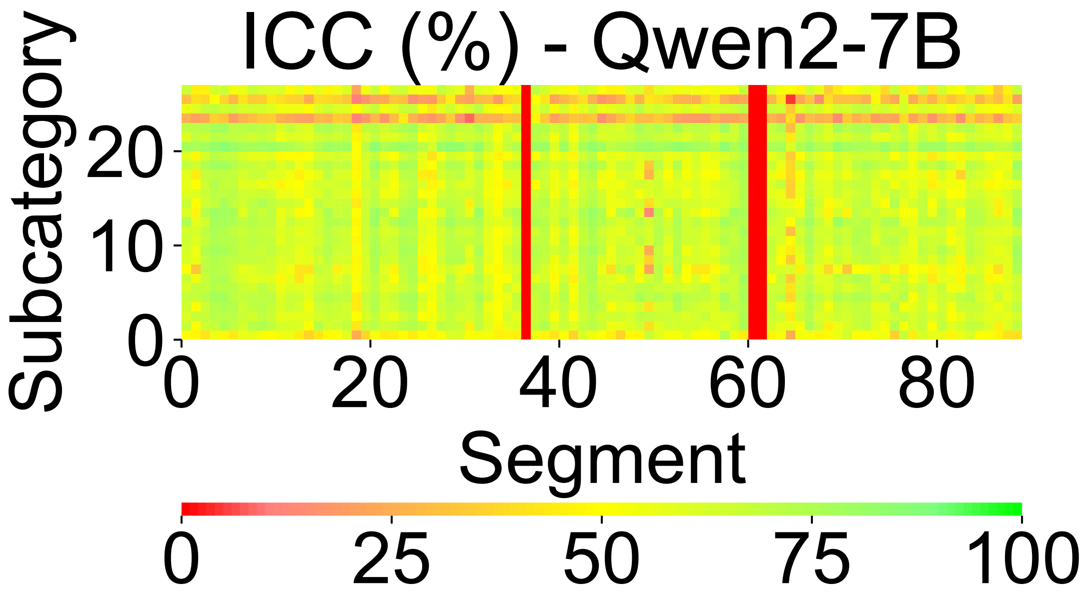

aa, Transitive consistency ratio heatmap for Qwen2-7B.

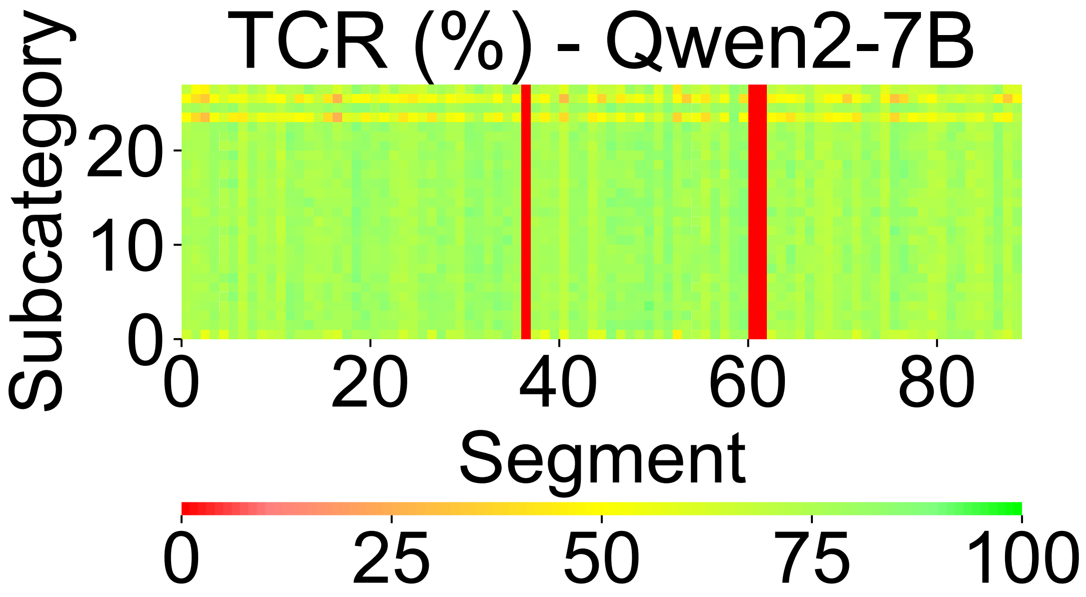

ab, Average performance score heatmap for Qwen2-7B.

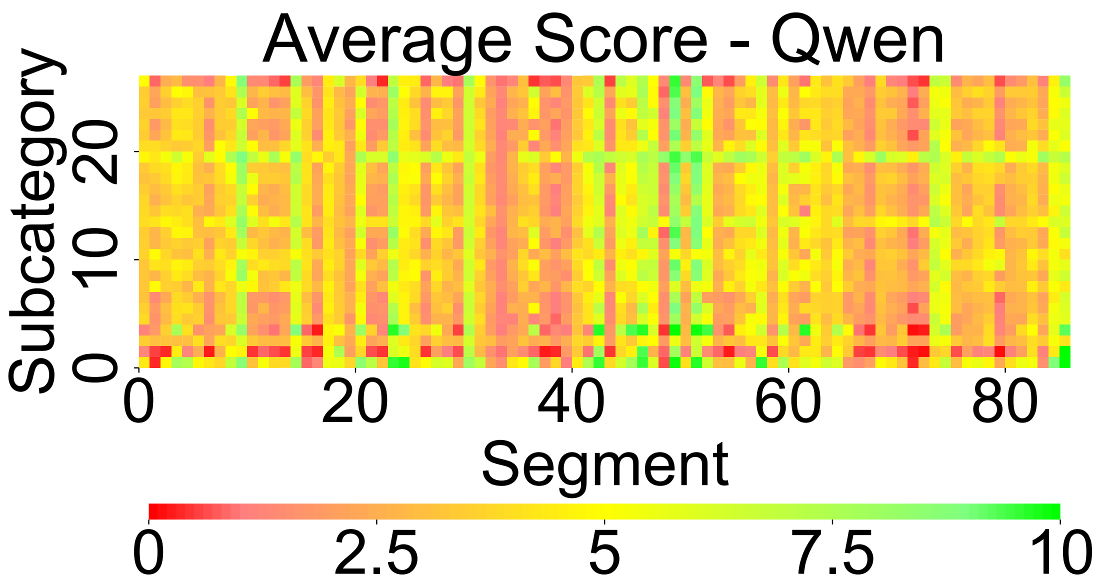

ac, Peak performance score heatmap for Qwen2-7B.

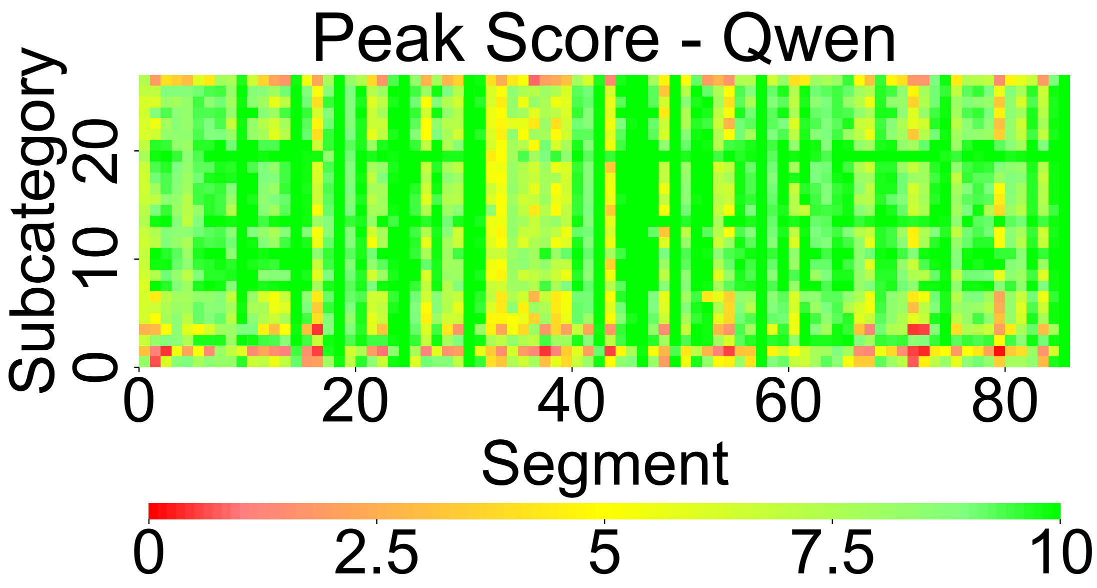

ad, Total score distribution heatmap for Qwen2-7B.

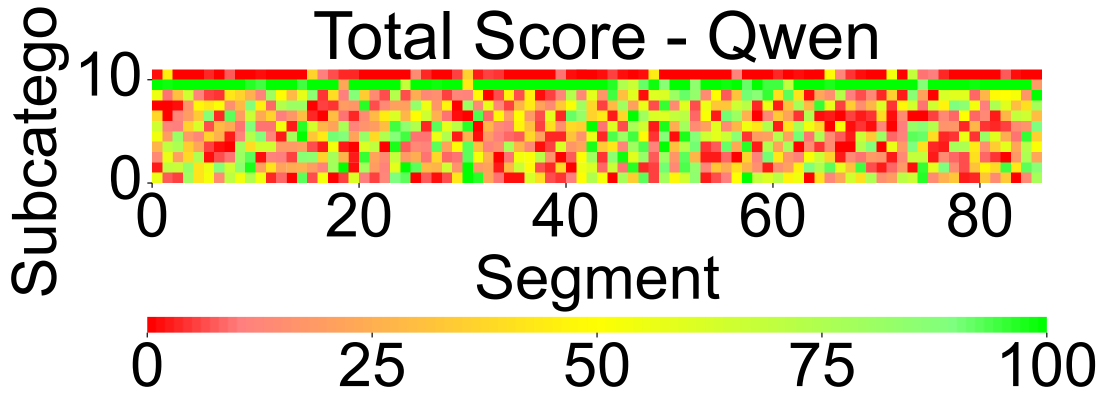

ae, Percentage mean difference heatmap comparing Qwen2-7B with baseline.

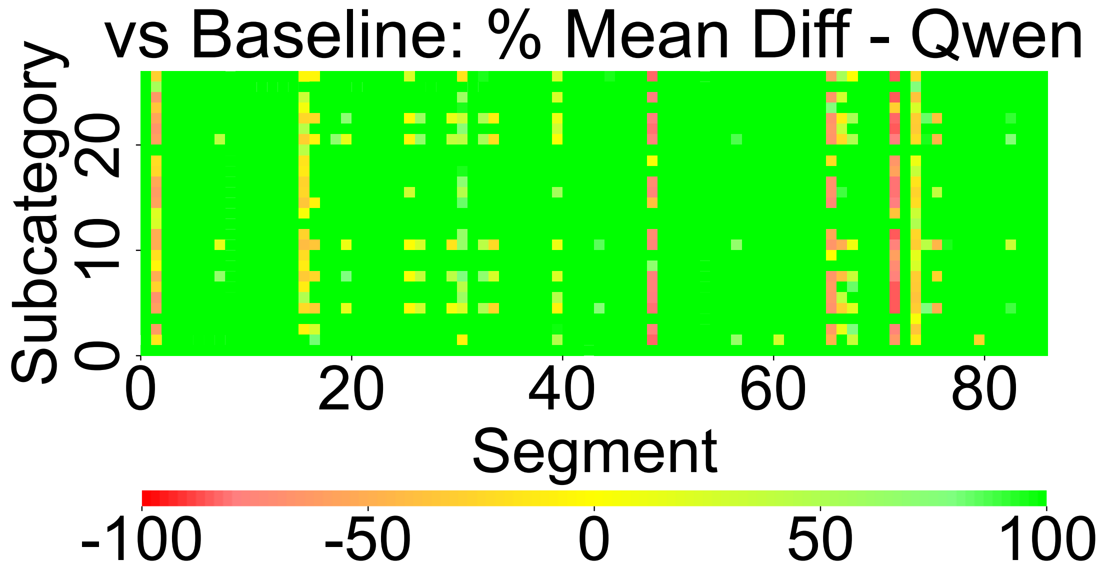

af, Percentage peak difference heatmap comparing Qwen2-7B with baseline.

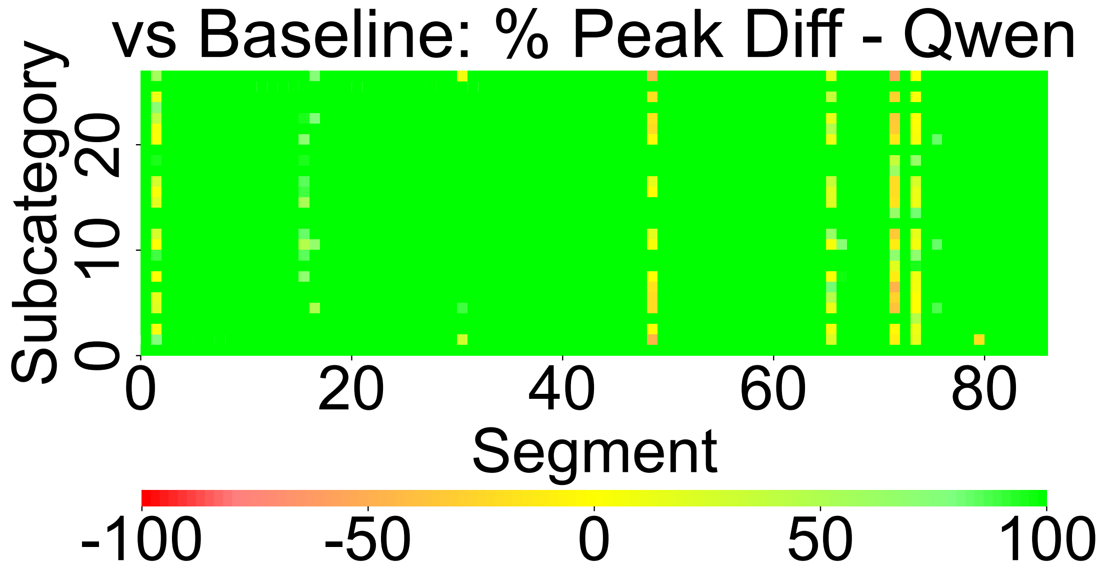

ag, Percentage mean difference heatmap comparing Qwen2-7B with human performance.

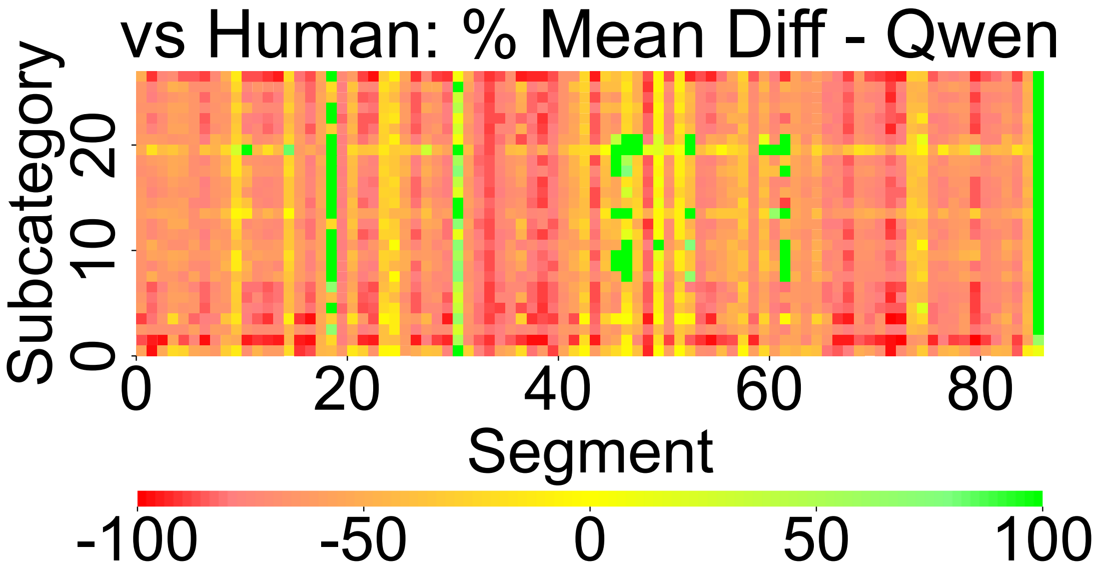

ah, Percentage peak difference heatmap comparing Qwen2-7B with human performance.

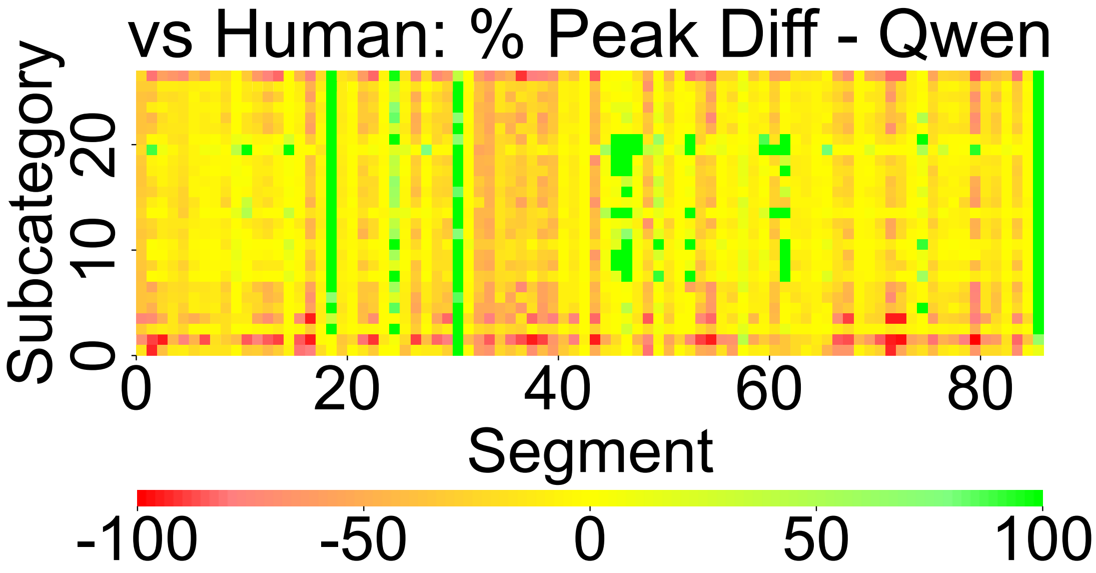

ai, Category-wise comparison of average scores between Claude3.5Sonnet and Qwen2-72B.

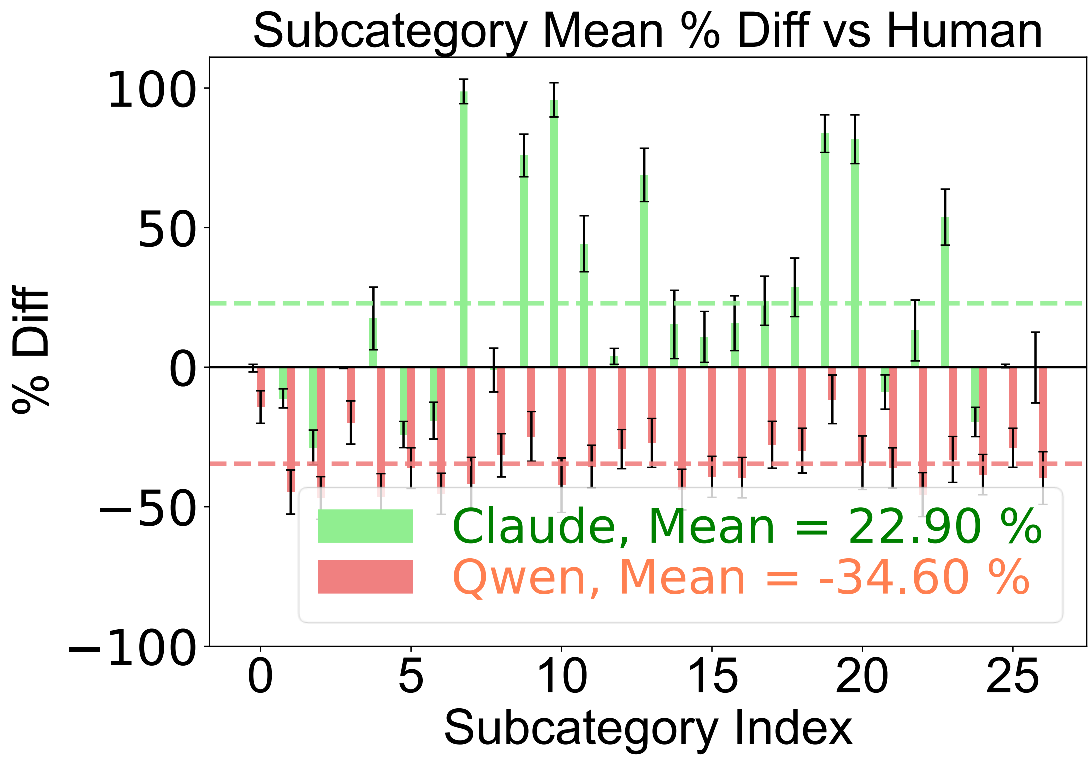

aj, Category-wise comparison of maximum scores between Claude3.5Sonnet and Qwen2-72B.

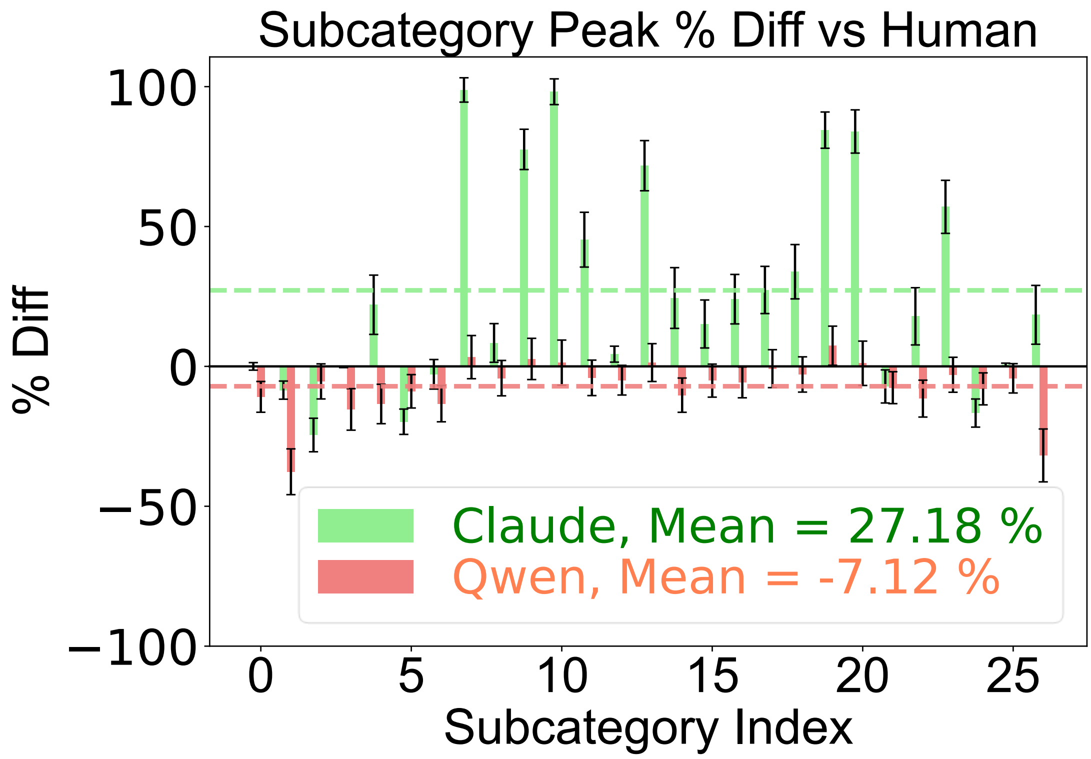

ak, Overall Bland-Altman plot comparing model performances.

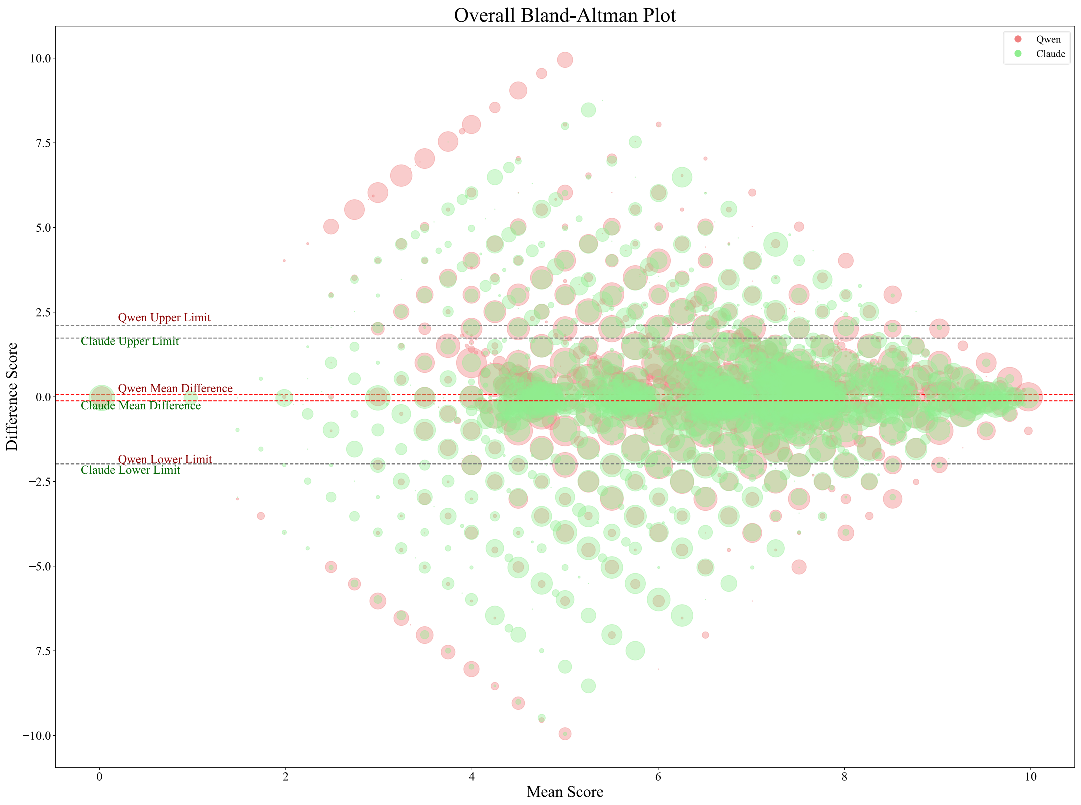

al, Bland-Altman plot for detailed performance comparison.

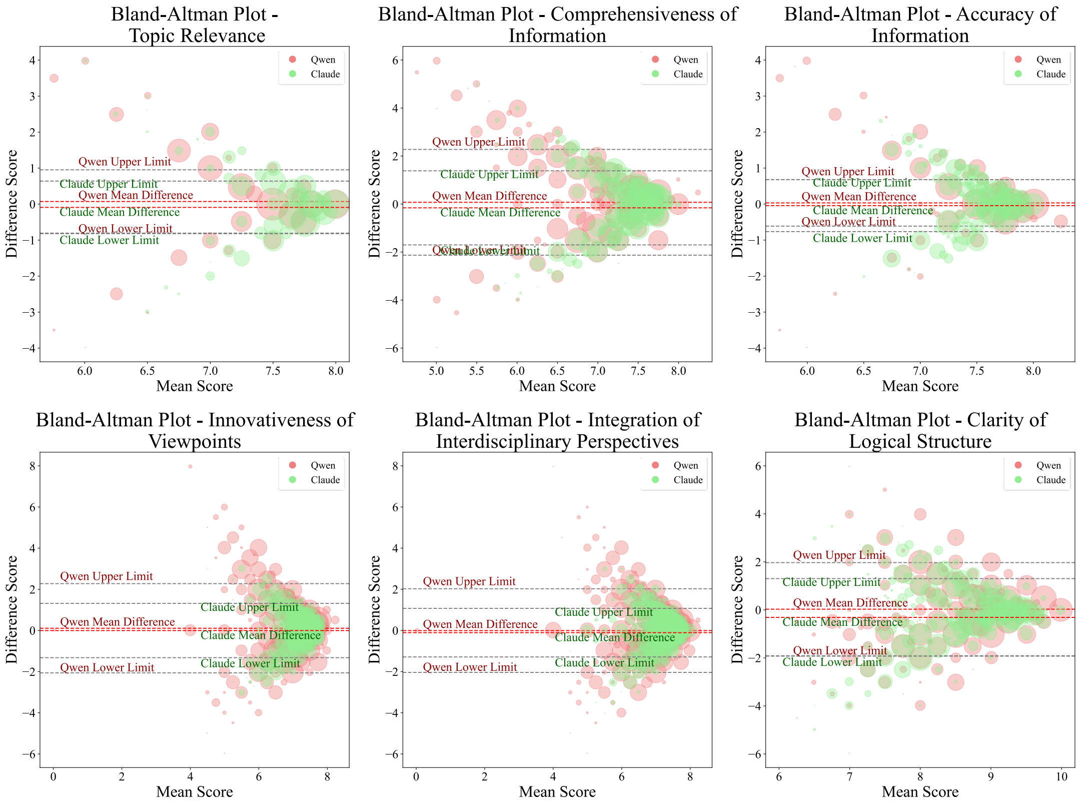

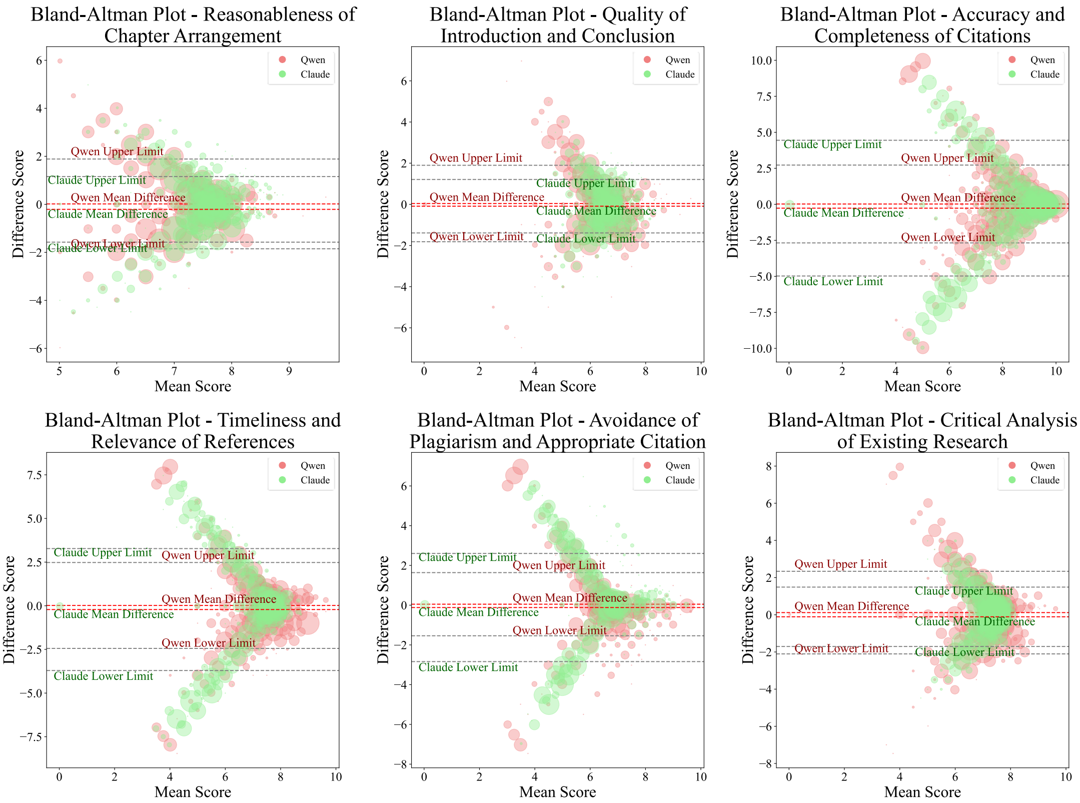
Bland-Altman Plot - Reasonableness of Chapter Arrangement
Bland-Altman Plot - Quality of Introduction and Conclusion
Bland-Altman Plot - Accuracy and Completeness of Citations
Bland-Altman Plot - Timeliness and Relevance of References
Bland-Altman Plot - Avoidance of Plagiarism and Appropriate Citation
Bland-Altman Plot - Critical Analysis of Existing Research
Difference Score
Mean Score
Qwen
Claude

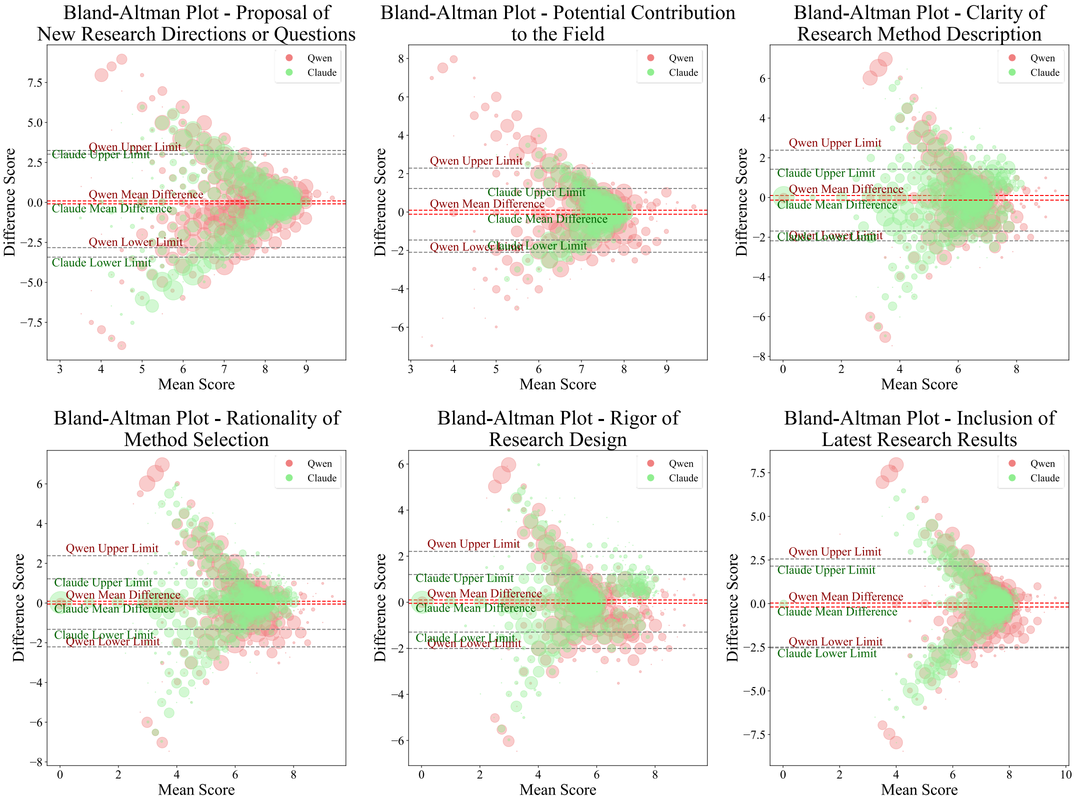
Bland-Altman Plot - Proposal of New Research Directions or Questions
Bland-Altman Plot - Potential Contribution to the Field
Bland-Altman Plot - Clarity of Research Method Description
Bland-Altman Plot - Rationality of Method Selection
Bland-Altman Plot - Rigor of Research Design
Bland-Altman Plot - Inclusion of Latest Research Results
Difference Score
Mean Score
Qwen
Claude

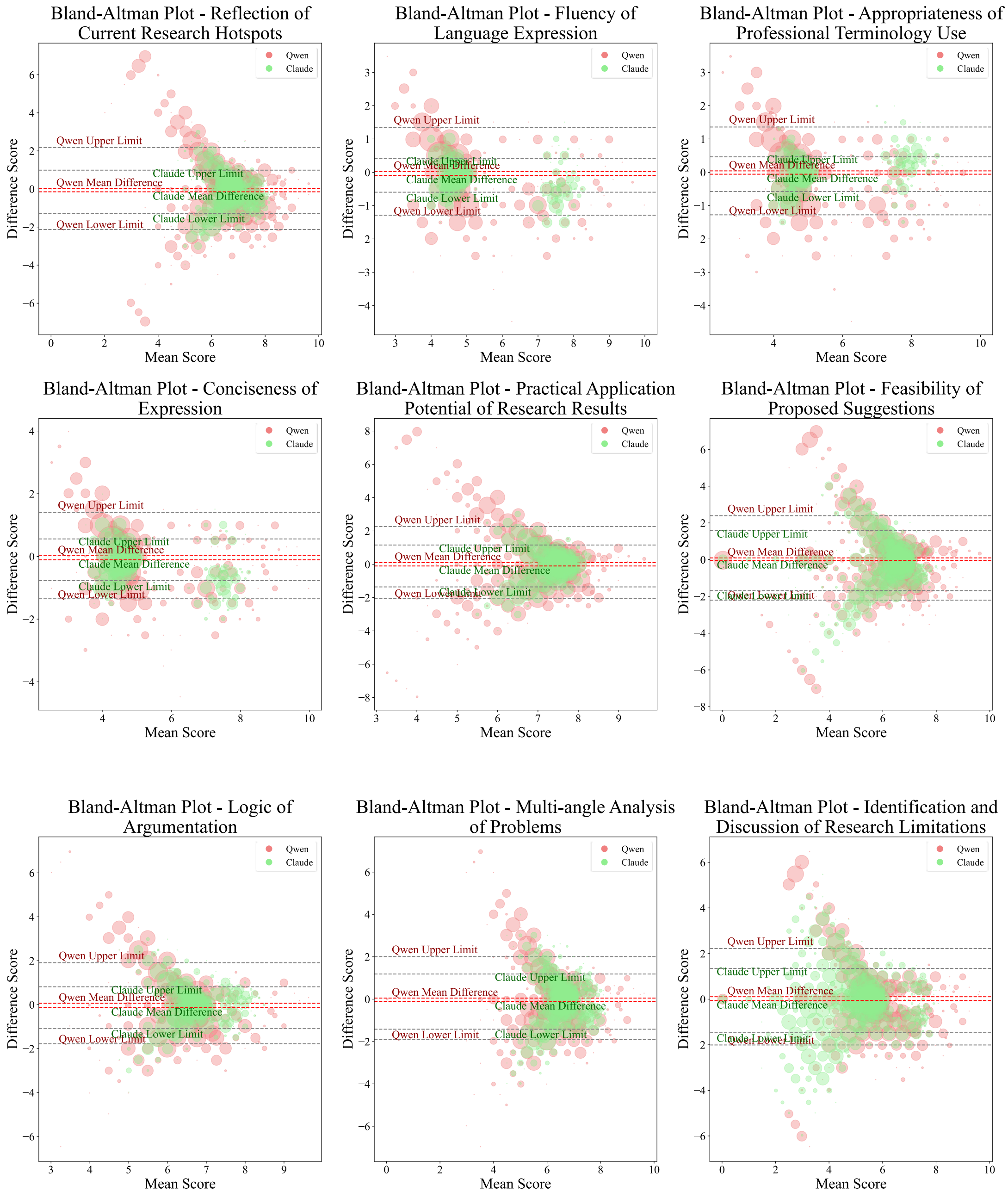

Fig.A11 Comprehensive performance analysis of LLM models in review generation. a, Scaling law analysis comparing performance between Qwen2-7b and Qwen2-72b models. **b,** Three-dimensional visualization comparing performance metrics across different LLM models. **c,** Distribution analysis of relative scores across all models. **d,** Histogram comparison of reliability between Claude3.5Sonnet and Qwen2-72b-Instruct. **e,** Comparative distribution analysis of maximum scores achieved by each model. **f,** Total maximum score comparison across all models. **g,** Comparative analysis of model scores relative to human performance. **h,** Intraclass correlation coefficient heatmap for Claude3.5Sonnet. **i,** Transitive consistency ratio heatmap for Claude3.5Sonnet. **j,** Average performance score heatmap for Claude3.5Sonnet. **k,** Peak

performance score heatmap for Claude3.5Sonnet. **l,** Total score distribution heatmap for Claude3.5Sonnet. **m,** Percentage mean difference heatmap comparing Claude3.5Sonnet with baseline. **n,** Percentage peak difference heatmap comparing Claude3.5Sonnet with baseline. **o,** Percentage mean difference heatmap comparing Claude3.5Sonnet with human performance. **p,** Percentage peak difference heatmap comparing Claude3.5Sonnet with human performance. **q,** Intraclass correlation coefficient heatmap for Qwen2-72B. **r,** Transitive consistency ratio heatmap for Qwen2-72B. **s,** Average performance score heatmap for Qwen2-72B. **t,** Peak performance score heatmap for Qwen2-72B. **u,** Total score distribution heatmap for Qwen2-72B. **v,** Percentage mean difference heatmap comparing Qwen2-72B with baseline. **w,** Percentage peak difference heatmap comparing Qwen2-72B with baseline. **x,** Percentage mean difference heatmap comparing Qwen2-72B with human performance. **y,** Percentage peak difference heatmap comparing Qwen2-72B with human performance. **z,** Intraclass correlation coefficient heatmap for Qwen2-7B. **aa,** Transitive consistency ratio heatmap for Qwen2-7B. **ab,** Average performance score heatmap for Qwen2-7B. **ac,** Peak performance score heatmap for Qwen2-7B. **ad,** Total score distribution heatmap for Qwen2-7B. **ae,** Percentage mean difference heatmap comparing Qwen2-7B with baseline. **af,** Percentage peak difference heatmap comparing Qwen2-7B with baseline. **ag,** Percentage mean difference heatmap comparing Qwen2-7B with human performance. **ah,** Percentage peak difference heatmap comparing Qwen2-7B with human performance. **ai,** Category-wise comparison of average scores between Claude3.5Sonnet and Qwen2-72B. **aj,** Category-wise comparison of maximum scores between Claude3.5Sonnet and Qwen2-72B. **ak,** Overall Bland-Altman plot comparing model performances. **al,** Bland-Altman plot for detailed performance comparison.

Appendix VIII：Prompt Templates

a, Topic extraction phase
i, Direct generation

Your task is to craft a detailed and logical question-based outline for a comprehensive review article about {TOPIC}. The outline should first be presented entirely in English, followed by its translation in Chinese after a line break. The detailed instructions are as follows:

Ensure that your outline adheres to the following criteria:

1. Develop at least 17 primary themes that are intricately linked to {TOPIC}, paving the way for a structured review article.

2. Under each primary theme, identify at least 7 detailed questions that probe deep into the respective themes, fostering detailed and insightful paragraphs in the final article.

3. Integrate both experimental and computational methodologies, including aspects from DFT and other theoretical approaches, evenly and coherently in the questions to offer a rounded perspective on {TOPIC}.

4. The progression of the primary themes should mirror a traditional review trajectory: starting with the foundational concepts, transitioning to advanced nuances, discussing prevailing challenges, and culminating with future prospects and visions.

5. Formulate the questions such that when answered, they collectively offer a wide-ranging view of the {TOPIC} landscape, without any significant overlap or omission of vital aspects.

Remember that this outline should function as a structured pathway for researchers, guiding them to draft a holistic review article on {TOPIC} without overlooking any critical details.

Start with the complete English version of the outline. After finishing it, provide the Chinese translation, maintaining the depth and essence of the English version, separated by a line break.

ii,Generation based on existing reviews

Based on the review about {TOPIC} presented in the attached file content, your task is to craft a detailed and logical question-based outline for a comprehensive review article. The outline should first be presented entirely in English, followed by its translation in Chinese after a line break. The detailed instructions are as follows:

<file-attachment-contents filename='[Name]'>
</file-attachment-contents>

Ensure that your outline adheres to the following criteria:

1. Develop at least 17 primary themes that are intricately linked to {TOPIC}, paving the way for a structured review article.

2. Under each primary theme, identify at least 7 detailed questions that probe deep into the respective themes, fostering detailed and insightful paragraphs in the final article.

3. Integrate both experimental and computational methodologies, including aspects from DFT and other theoretical approaches, evenly and coherently in the questions to offer a rounded perspective on {TOPIC}.

4. The progression of the primary themes should mirror a traditional review trajectory: starting with the foundational concepts, transitioning to advanced nuances, discussing prevailing challenges, and culminating with future prospects and visions.

5. Formulate the questions such that when answered, they collectively offer a wide-ranging view of the {TOPIC} landscape, without any significant overlap or omission of vital aspects.

Remember that this outline should function as a structured pathway for researchers, guiding them to draft a holistic review article on {TOPIC} without overlooking any critical details.

Start with the complete English version of the outline. After finishing it, provide the Chinese translation, maintaining the depth and essence of the English version, separated by a line break.

iii, Integration questions for review

Your task is to construct a structured, question-based outline for a comprehensive review article on the topic detailed in the provided attachment, with a significant emphasis on {TOPIC}. The focus should be on presenting a thorough exploration across various dimensions of {TOPIC}, smoothly transitioning from a foundational understanding to an in-depth analysis of specific instances related to {TOPIC}. The outline should facilitate a logical flow between a broad range of themes, consistently maintaining a focal emphasis on the core elements of {TOPIC}.

Instructions:

*1. **Introduction***

- Begin with a section that lays the groundwork for the {TOPIC}, detailing its background, historical developments, and the evolution of key elements or breakthroughs within this field.

*2. **Foundational Theories and Principles***

- Include a section on the fundamental theories and principles that form the basis of {TOPIC}, providing a foundation for a deeper exploration into specific areas or instances in later sections.

*3. **Detailed Examination of Specific Instances***

- Significantly expand the section dedicated to the specific instances or case studies of {TOPIC}, covering a broad spectrum of applications, variations, or case studies that illustrate the depth and breadth of the field.

*4. **Methodologies and Techniques***

- Delve into the diverse methodologies and techniques that are vital to researching and understanding {TOPIC}, maintaining an equitable focus on both theoretical and practical approaches.

*5. **Integrative Discussion Section***

- Include a single section that integrates discussions on related sub-topics, such as technological, economic, and sustainability aspects, highlighting their interconnections and impacts on the main {TOPIC}.

*6. **Conclusion and Future Directions***

- Conclude with a synthesis of insights from all sections, providing a comprehensive overview and identifying potential future research avenues in the realm of {TOPIC}.

*7. **Output Format**:*

- Present your outline in English as per the XML structure provided below. The outline should encompass all the aforementioned sections, each clearly identified and structured in a logical

manner.

The expected XML structure is:

```xml
<?xml version="1.0" encoding="UTF-8"?>
<outlines>
	<outline id="1">
		English outlines 1
	</outline>
	<outline id="2">
		English outlines 2
	</outline>
	...
	<outline id="n">
		English outlines n
	</outline>
</outlines>
```

Ensure that the outline serves as a rich source of information, seamlessly transitioning from theoretical underpinnings to a thorough exploration of {TOPIC}, and culminating in a cohesive conclusion that offers a vision for the future. The structure should be traditional yet detailed, emphasizing extensively on the key facets of {TOPIC}.

<file-attachment-contents filename='[Name]'>
</file-attachment-contents>

iv, Paper questions from paragraph questions for review

Utilizing the document provided, create a set of structured questions that correlate with the specific segments related to {TOPIC} as outlined in the literature. These questions are intended to extract precise information directly from the document to construct a comprehensive review article. It's essential to tailor each question to uncover the details specified in the segments of the document, ensuring an in-depth accumulation of data that aligns with the broader queries in the review's outline.

Construct each question by combining the main topic {TOPIC} with the pertinent questions from the document's individual subsections, then reorder them to present a logical sequence. For instance, if the document presents the following segment:

```
1. Background and Significance
    - What is spin and catalyst?
    - What is its industrial significance?
```

The corresponding questions tailored for information extraction should be formulated and ordered as:

```
1. Background and Significance - What are the core principles of spin and catalyst as described in the document?
2. Background and Significance - How has spin and catalyst impacted the industry according to the document?
```

Repeat this process for each segment, ensuring the questions are distinct and provide a clear framework for extracting detailed responses based on the document's specifics.

Your ultimate aim is to create a sequence of questions that will enable the gathering of rich, specific information from the document to develop a well-rounded perspective on each section related to {TOPIC}.

After drafting the detailed set of questions in English, translate them into Chinese, preserving the original depth and intention to support a bilingual information extraction process.

Output the complete English questions first, each clearly numbered. Follow this with the Chinese version, keeping each question separate and ensuring the two language sets are distinctly partitioned.

<file-attachment-contents filename='[Name]'>
</file-attachment-contents>

b, Knowledge extraction phase
i, Information extraction
I'm going to give you a scientific literature. Then I'm going to ask you some questions about it. I'd like you to first write down exact quotes of parts of the document word by word that would help answer the question, and then I'd like you to answer the question using facts from the quoted content. Do not omit any relevant information from the text, and avoid introducing any falsehoods or assumptions that aren't directly supported by the literature. Here is the literature, in <literature></literature> XML tags:

<literature>
</literature>

Here are the question lists, in <questions></questions>XML tags:
<questions>
</questions>

First, you need to sequentially extract any quotes in the literature that are most relevant to each question, and print them in numbered order, separated by newlines. Quotes should be relatively brief. Do not attempt to summarize or answer questions at this stage, but simply repeat exactly what the corresponding part of the literature says.

Please enclose the full list of quotes in <quotes></quotes> XML tags. If there are no relevant quotes, write "No relevant quotes" instead.

Then, answer the question, starting with "Answer:". Do not include or reference quoted content verbatim in the answer. Don't say "According to Quote [1]" when answering. Do not write reference number of quotes after answer. Put your answer to the user inside <EnglishVersionAnswer></EnglishVersionAnswer> XML tags. Output formatted text, with line breaks for each question.Separate quotes and answers with a blank line. Provide the answers to all questions in English. After completing the English answers, translate all those answers into Chinese and provide the Chinese version inside <ChineseVersionAnswer></ChineseVersionAnswer> XML tags.

Thus, the format of your overall response should look like what's shown between the <example></example> tags. Make sure to follow the formatting and spacing exactly.

<example>

<quotes>

[1] "Company X reported revenue of $12 million in 2021."

</quotes>

<EnglishVersionAnswer>

1.Company X earned $12 million in 2021.

</EnglishVersionAnswer>

<ChineseVersionAnswer>

1.X 公司在 2021 年赚了 1200 万美元。

</ChineseVersionAnswer>

<quotes>

[1] "Almost 90% of revenue came from widget sales, with gadget sales making up the remaining 10%."

</quotes>

<EnglishVersionAnswer>

2.Almost 90% of it came from widget sales.

</EnglishVersionAnswer>

<ChineseVersionAnswer>

2.几乎 90%的收入来自小部件销售。

</ChineseVersionAnswer>

</example>

If the question cannot be answered by the document, say so.If deemed necessary, the answer to the question can be extended entirely from the content of the document.

Answer all of the questions immediately without preamble.

ii, Answer integration
Read the questions and answers provided below. First, critically assess the overall relevance of the answers provided to the set of questions asked.

If, upon your assessment, you find that the answers do not contain information that is relevant to the questions asked, stop your review process immediately and respond with a single sentence: "※※※※※※※The provided answers are not relevant to the questions.※※※※※※※". Do not provide any additional explanation or background information, only this sentence should be given as a response in case of irrelevant answers.

If, however, the answers are relevant to the questions asked, proceed to compile answers for each question according to the instructions below. Ensure to aggregate all the relevant answers from the multiple answer results provided in the document, and organize them sequentially by their order number, compiling the corresponding quotes, English answers, and Chinese answers for each question.

If the provided answers' quotes are not differentiated by question, ensure to break them down and

assign the quotes to each respective question, outputting them separately within each question's section.

To provide a comprehensive review, differentiate the responses into quotes, English answers, and Chinese answers for each question based on the details given in the 'Answer' XML tags. Structure your review using the XML format showcased below if the answers are relevant to the questions asked:

<?xml version="1.0" encoding="UTF-8"?>
<Questions>
<Question number="1">
<Quotes>
Quotes for question 1 from all the answer results
</Quotes>
<English>
Aggregated English answer for question 1 from all the answer results
</English>
<Chinese>
所有答案结果中的汇总中文答案 1
</Chinese>
</Question>
<Question number="2">
<Quotes>
Quotes for question 2 from all the answer results
</Quotes>
<English>
Aggregated English answer for question 2 from all the answer results
</English>
<Chinese>
所有答案结果中的汇总中文答案 2
</Chinese>
</Question>
</Questions>

Here are the question lists, in <questions></questions>XML tags:
<questions>
</questions>

Here are the answer lists, in <Answer></Answer>XML tags:
<Answer>
</Answer>

c, Review composition phase

i, Text organization

Based on the in-depth details extracted from the file related to '[question]', construct an analytical and comprehensive review section on [Theme], emphasizing '[Topic]'.

While developing the content, adhere to the following protocols:

1. **Accurate Citations**: Reference specific content from the file by embedding the actual DOI numbers furnished, without any alterations. Utilize the format '\[Placeholder_Of_DOI\]' right after the sentence where the reference is applied.

2. **Strict Adherence**: Stick to the particulars and DOI details from the file; avoid integrating external or speculative data.

3. **Scientific Language**: Uphold a technical and scholarly diction akin to chemical

engineering literature.

4. **Format & Translation**: After creating the main review content, append an 'integrative understanding and prospective outlook' section within the same <English></English> and <Chinese></Chinese> XML tags, demarcated with '※※※'. This segment should transcend a mere summation and foster a forward-thinking discussion, potentially elucidating future directions and broader horizons grounded in the file's content.

The content structure should resemble:

<example>
<English>
Detailed analysis established from the study of reference \[Placeholder_Of_DOI1\]. Synthesized comprehension stemming from references \[Placeholder_Of_DOI2\] and \[Placeholder_Of_DOI3\].
※※※
Integrative understanding and prospective outlook: Taking into consideration the advancements and findings discussed in the file, there lies an opportunity to explore emerging fields and innovative methodologies. Future research endeavors might focus on ...
</English>
<Chinese>
基于\[Placeholder_Of_DOI1\]参考文献的深度分析。从\[Placeholder_Of_DOI2\]和\[Placeholder_Of_DOI3\]的参考文献中获得的综合理解。
※※※
综合理解与未来展望: 考虑到文件中讨论的先进成果和发现，我们有机会探索新兴领域和创新方法。未来的研究努力可能会集中在...
</Chinese>
<References>
Placeholder_Of_DOI1
Placeholder_Of_DOI2
Placeholder_Of_DOI3
</References>
</example>

In the 'integrative understanding and prospective outlook' segment, aspire to:

- **Offer an expansive perspective**: Illuminate potential pathways and pioneering research opportunities, grounded in the details divulged in the file.
- **Propose forward-thinking suggestions**: Advocate for innovative angles and burgeoning domains that might take center stage in future explorations, while rooted in the file's details.

Finally, compile all the cited DOIs in the 'References' compartment, adhering to the <References></References> XML tag, using the exact DOIs designated in the file.

<file-attachment-contents filename="Paragraph1Info.txt">
</file-attachment-contents>

ii, Text compression
Text Compression Task

You will be provided with the following information:

Topic: {TOPIC}
Question: {QUESTION}
Quotes: {QUOTES}
English Text: {ENGLISH}

Understanding the inputs:
1. Topic: This provides the overall context or subject area of the text. Use this to understand the broader theme and to ensure your compressed text remains relevant to the main subject.

2. Question: This is the specific query that the English text is attempting to answer. Your compressed text should focus on addressing this question effectively.

3. Quotes: These are relevant excerpts that may provide key information or support the main points of the text. Consider integrating these into your compressed version if they are crucial to answering the question.

4. English Text: This is the main body of text that you need to compress. It contains the detailed information addressing the question within the context of the topic.

Based on this information, create a compressed version of the English paragraph. Follow these guidelines:

1. Carefully read and analyze all provided inputs, understanding how they relate to each other.

2. Use the Chain of Thought (COT) method to compress the text:
a. Identify the main themes and key information in the paragraph, considering how they relate to the topic and question.
b. Determine which information is core to answering the question and which is secondary.
c. Consider how to express the same meanings using more concise language.
d. Think about how to combine related ideas to reduce redundancy.
e. Evaluate how the quotes can be integrated to support key points.

3. Create a compressed version ensuring that you:
- Retain all key concepts and main ideas relevant to the topic and essential for answering the question
- Maintain the logical flow of information
- Preserve important details and examples, especially those from the quotes that directly support the answer
- Remove redundant or less critical information that doesn't directly contribute to addressing the question
- Use concise language without altering the original meaning
- Maintain the original tone and writing style

4. The compressed text should stand alone as a coherent summary that effectively addresses the question within the context of the topic.

5. Do not introduce new information not present in the original text or quotes.

Output format:
```json
{
  "thought_process": [
    "Step 1: ...",
    "Step 2: ...",
    "Step 3: ..."
  ],
  "compressed_text": "Your compressed English text here"
}
```

In the "thought_process", detail your compression process, explaining:

- How you used the topic and question to guide your compression
- Why you chose to keep certain information while removing others
- How you integrated relevant quotes
- How you ensured the core meaning of the original text was preserved while effectively addressing the question and staying relevant to the topic

iii, Paragraph scoring

Based on the attached content below containing multiple review paragraphs focused on the topic of "[Topic]", evaluate each paragraph on the following dimensions:

1. **Clarity** (10 points): Is the paragraph clearly written and easy to understand?
2. **Depth** (10 points): Does the paragraph delve deep into the topic and offer nuanced insights?
3. **Relevance** (10 points): Is the content of the paragraph directly related to the topic "The introduction, principles, and application of tandem catalysis in chemical engineering processes, with a focus on improving efficiency and reducing costs, exemplified by a novel nanoscale porous overcoating catalyst for coupling catalytic propane dehydrogenation with selective H2 combustion.?
4. **Coherence** (10 points): Does the paragraph maintain a logical flow and coherence in its discussion?
5. **Originality** (10 points): Does the paragraph offer new perspectives or original ideas related to the topic?
6. **Evidence-based** (10 points): Is the content backed by relevant evidence or references?
7. **Structure** (10 points): Is the paragraph well-structured with a clear beginning, middle, and end?
8. **Text Length** (20 points): Does the paragraph maintain an appropriate length? The longer, the better.
9. **DistinctNumberOfDOIs** (20 points): Count the distinct DOIs (format '10\.\d{4,9}\[/_\]+\[-._;()/:A-Za-z0-9\]+') in each paragraph and compare. Assign a relative score based on the number of unique references compared to other paragraphs; the paragraph with the most unique references gets the highest score.
10. **Comprehensiveness** (10 points): Does the paragraph cover all pertinent aspects related to the topic in a comprehensive manner?
11. **Relatedness** (20 points): Does the paragraph exhibit thematic consistency with other paragraphs when discussing similar or identical DOI references? Is the paragraph's explanation and context concerning a specific DOI analogous to that in other paragraphs that cite the same DOI?

Regarding the new **Relatedness** criterion:
- Analyze the paragraphs that share common DOI references, assessing the degree of similarity in the contextual use and discussion surrounding those references.
- Evaluate whether the paragraph aligns with or diverges from the shared thematic discussions related to the DOI in question when compared to other paragraphs that cite the same DOI.
- Ensure that the relatedness is not merely surface-level or lexical but digs deeper into the thematic and contextual consistency across different paragraphs that cite the same DOI.

Using these criteria, evaluate each paragraph methodically, ensuring that each dimension is assessed with rigor and impartiality. Subsequently, the paragraph that amasses the highest cumulative score across all dimensions should be selected as the one that most effectively addresses the topic at hand, while also maintaining cohesion, depth, and thematic alignment with related discussions in different paragraphs. Remember to be meticulous and transparent in the scoring to ensure that the selection is justifiable and replicable.

<file-attachment-contents filename="Paragraphs.txt">
<Paragraphs>
</Paragraphs>
</file-attachment-contents>
After evaluating all paragraphs for each dimension, provide scores for each dimension individually.

For example:

<Scores>
<Paragraph id="1">
<Clarity>8</Clarity>
<Depth>7</Depth>
<Relevance>9</Relevance>
<Coherence>7</Coherence>
<Originality>6</Originality>
<Evidence-based>8</Evidence-based>
<Structure>9</Structure>
<TextLength>16</TextLength>
<DistinctNumberOfDOIs>18</DistinctNumberOfDOIs>
<Comprehensiveness>8</Comprehensiveness>
<TotalScore>88</TotalScore>
</Paragraph>
<!-- Scores for additional paragraphs -->
</Scores>

Finally, identify and present the paragraph that achieved the highest combined score:

<BestParagraphResult>
<ParagraphID>1</ParagraphID>
<Content>
{Raw content of the top-scoring paragraph}
<References>
{Raw references from the top-scoring paragraph}
</References>
</Content>
</BestParagraphResult>

iv, Full text refinement

With the draft of the chemical engineering review article at hand, proceed to refine and enhance the content in its entirety. Here are your instructions:

*1. **Scientific Language**: Apply rigorous and academic language suitable for the chemical engineering community, ensuring precision in the technical details.*
*2. **Content Enhancement**: Without altering the original meaning or data, elevate the clarity, fluency, and expression of ideas throughout the document.*
*3. **Structure Preservation**: Maintain the draft's paragraph structure, seamlessly integrating enhancements within the existing framework.*
*4. **Citation Accuracy**: Embed the provided DOI numbers accurately and appropriately in the format '[actual DOI from the file]', directly following the referenced information.*
*5. **Abstract Creation**: Compose a comprehensive abstract that concisely summarizes the article's primary focus and findings.*
*6. **Title Formulation**: Devise a fitting academic title that encapsulates the essence of the review.*
*7. **Reference Listing**: Keep all original references intact, listing them with their exact DOIs in the prescribed format at the end of the main text after all sections done.*
*8. **Complete Output**: Deliver the polished review in one continuous output, ensuring the content is uninterrupted and cohesive from start to finish.*

Focus on producing a detailed and complete polished review, presenting it as one continuous text that aligns with the original draft's guidance and the academic standards of chemical engineering literature.

continue, Deliver the polished review in one continuous output, ensuring the content is uninterrupted and cohesive from section 10 to finish. Focus on producing a detailed and complete

polished review, presenting it as one continuous text that aligns with the original draft's guidance and the academic standards of chemical engineering literature.

Give the new DOI list of references after rewriting, requiring a one-to-one correspondence with the actual citation order in the text, with "[index]"

d, Review evaluation phase
i, Topic extraction
# Paragraph Topic Extractor and Question Generator

You are an expert in academic writing and text analysis. Your task is to extract the main topic from a given paragraph of an academic review and then generate a single comprehensive question based on that topic. Follow these steps carefully:

1. Read the provided paragraph carefully.
2. Analyze its content to identify the main topic.
3. Formulate a concise topic statement.
4. Based on the extracted topic, create a single comprehensive question that can guide the extraction of detailed information from the paragraph.
5. Translate the question into Chinese.

Output your results in the following XML format:

```xml
<analysis>
  <original_paragraph>
    [Insert the original paragraph here]
  </original_paragraph>

  <topic_extraction>
    <step1_initial_thoughts>
      [Provide your initial thoughts about the paragraph's content]
    </step1_initial_thoughts>

    <step2_key_concepts>
      [List the key concepts or ideas present in the paragraph]
    </step2_key_concepts>

    <step3_topic_formulation>
      [Explain how you're formulating the topic based on the key concepts]
    </step3_topic_formulation>

    <extracted_topic>
      [State the extracted topic in a clear, concise manner]
    </extracted_topic>
  </topic_extraction>

  <question_generation>
    <step1_question_formulation>
      [Explain how you're formulating the question based on the extracted topic]
    </step1_question_formulation>

    <generated_question>
      <english>[Present the final version of your generated question in English]</english>
      <chinese>[Present the Chinese translation of the generated question]</chinese>
    </generated_question>
  </question_generation>
```

```
</analysis>
```

Remember to:
- Be thorough in your analysis and explanation.
- Ensure that the extracted topic accurately represents the main idea of the paragraph.
- Create a single, comprehensive question that can elicit detailed information about the topic from the paragraph.
- Use academic language appropriate for the field of study.
- Provide both English and Chinese versions of the generated question.

<file-attachment-contents filename="Paragraphs.txt">
<Paragraphs>
[]
</Paragraphs>
</file-attachment-contents>

ii, Topic Integration

Integration of Multiple Topic Extractions and Questions

You are an expert in academic writing, text analysis, and information synthesis. Your task is to integrate multiple topic extractions and generated questions from the same document into a cohesive and comprehensive summary. Follow these steps carefully:

1. Review all provided topic extractions and generated questions.
2. Identify common themes, key concepts, and overlapping ideas across all inputs.
3. Synthesize this information into a unified topic and comprehensive question.
4. Ensure the final output reflects a deep understanding of the entire document, not just individual sections.

Use the following XML template for your output, integrating all relevant information into a single, cohesive entry:

```xml
<integrated_analysis>
  <synthesized_topic_extraction>
    <common_themes>
      [List the common themes identified across all inputs]
    </common_themes>

    <key_concepts>
      [Enumerate the key concepts that appear consistently across inputs]
    </key_concepts>

    <integration_process>
      [Explain your process of synthesizing the various topics and ideas]
    </integration_process>

    <final_extracted_topic>
      [Present the final, synthesized topic that encompasses all inputs]
    </final_extracted_topic>
  </synthesized_topic_extraction>

  <synthesized_question_generation>
    <question_integration_approach>
      [Describe how you're combining and refining the individual questions]
    </question_integration_approach>
```

<final_generated_question>
<english>[Present the final, comprehensive question in English that covers all aspects of the synthesized topic]</english>
<chinese>[Present the Chinese translation of the final generated question]</chinese>
</final_generated_question>
</synthesized_question_generation>
</integrated_analysis>
```

Remember to:
- Focus on creating a cohesive narrative that captures the essence of all inputs.
- Avoid simply listing or concatenating individual items.
- Ensure the final topic and question are broad enough to encompass all key points from the inputs, yet specific enough to guide a focused discussion.
- Use academic language appropriate for the field of study.
- Provide both English and Chinese versions of the final generated question.

<file-attachment-contents filename="Analysis.txt">
<Analysis>
<analysis_0>
</analysis_0>
</Analysis>
</file-attachment-contents>

iii, Direct generation
Write a paragraph around the following topic:
[Topic]

**iii, Pairwise scoring**

You are a professional literature review expert. Your task is to evaluate and compare two review articles on the topic of "The introduction, principles, and application of tandem catalysis in chemical engineering processes, with a focus on improving efficiency and reducing costs, exemplified by a novel nanoscale porous overcoating catalyst for coupling catalytic propane dehydrogenation with selective H2 combustion." based on the provided scoring criteria. Please carefully read the following instructions and scoring criteria, then evaluate the two provided articles.

The scoring criteria are as follows:

{
"Content Quality": {
"Topic Relevance": {
"Description": "Relevance and importance to the topic",
"Maximum Score": 8
},
"Comprehensiveness of Information": {
"Description": "Breadth and depth of topic coverage",
"Maximum Score": 8
},
"Accuracy of Information": {
"Description": "Correctness of data and statements",
"Maximum Score": 8
},
"Innovativeness of Viewpoints": {
"Description": "Novel insights and unique perspectives",
"Maximum Score": 8
```

```
    },
    "Integration of Interdisciplinary Perspectives": {
        "Description": "Degree of multidisciplinary knowledge integration",
        "Maximum Score": 8
    }
},
"Structure and Organization": {
    "Clarity of Logical Structure": {
        "Description": "Coherence of argumentation process",
        "Maximum Score": 10
    },
    "Reasonableness of Chapter Arrangement": {
        "Description": "Balance and sequence of content distribution",
        "Maximum Score": 8
    },
    "Quality of Introduction and Conclusion": {
        "Description": "Effectiveness of opening and closing",
        "Maximum Score": 7
    }
},
"Academic Standards": {
    "Accuracy and Completeness of Citations": {
        "Description": "Correctness of citation format and content",
        "Maximum Score": 10
    },
    "Timeliness and Relevance of References": {
        "Description": "Recency and relevance of literature",
        "Maximum Score": 8
    },
    "Avoidance of Plagiarism and Appropriate Citation": {
        "Description": "Originality and appropriateness of citations",
        "Maximum Score": 7
    }
},
"Innovation and Contribution": {
    "Critical Analysis of Existing Research": {
        "Description": "Depth of analysis and insight",
        "Maximum Score": 8
    },
    "Proposal of New Research Directions or Questions": {
        "Description": "Foresight and inspirational quality",
        "Maximum Score": 9
    },
    "Potential Contribution to the Field": {
        "Description": "Degree of theoretical or practical advancement",
        "Maximum Score": 8
    }
},
"Methodology Assessment": {
    "Clarity of Research Method Description": {
        "Description": "Clarity of methodological explanation",
        "Maximum Score": 7
    },
    "Rationality of Method Selection": {
        "Description": "Match between method and research question",
        "Maximum Score": 7
    },
```

```
        "Rigor of Research Design": {
            "Description": "Scientific nature and reliability of design",
            "Maximum Score": 6
        }
    },
    "Timeliness and Cutting-edge Nature": {
        "Inclusion of Latest Research Results": {
            "Description": "Degree of citation of latest literature",
            "Maximum Score": 8
        },
        "Reflection of Current Research Hotspots": {
            "Description": "Coverage of hot issues",
            "Maximum Score": 7
        }
    },
    "Writing Quality": {
        "Fluency of Language Expression": {
            "Description": "Smooth writing, easy to understand",
            "Maximum Score": 5
        },
        "Appropriateness of Professional Terminology Use": {
            "Description": "Accuracy and consistency of terminology use",
            "Maximum Score": 5
        },
        "Conciseness of Expression": {
            "Description": "Degree of language refinement",
            "Maximum Score": 5
        }
    },
    "Practicality and Application Value": {
        "Practical Application Potential of Research Results": {
            "Description": "Guiding significance for practice",
            "Maximum Score": 8
        },
        "Feasibility of Proposed Suggestions": {
            "Description": "Realism and operability of suggestions",
            "Maximum Score": 7
        }
    },
    "Critical Thinking": {
        "Logic of Argumentation": {
            "Description": "Rationality of reasoning process",
            "Maximum Score": 7
        },
        "Multi-angle Analysis of Problems": {
            "Description": "Diversity and comprehensiveness of perspectives",
            "Maximum Score": 7
        },
        "Identification and Discussion of Research Limitations": {
            "Description": "Recognition and analysis of limiting factors",
            "Maximum Score": 6
        }
    }
}
```


Please follow these steps for the evaluation:

1. Carefully read the two provided review articles.
2. Score each article based on every evaluation dimension and specific item in the scoring criteria.
3. Provide a concise scoring rationale for each point, and indicate the strengths and weaknesses of both articles.
4. Calculate the overall score for each article.
5. Determine which review is superior based on the overall scores and analysis.
6. Output the evaluation results in the specified JSON format.

During the evaluation process, please note the following:

- Maintain objectivity: Base your evaluation on facts and article content, avoiding subjective bias.
- Provide concise scoring rationales: Summarize the basis for scoring in brief sentences or phrases.
- Consider disciplinary characteristics: Judge the quality and contribution of the reviews based on the characteristics of their respective disciplines.
- Note subtle differences: Even if the overall quality is similar, strive to identify and articulate the differences between the two articles.
- Weigh pros and cons: When drawing final conclusions, consider performance across all dimensions comprehensively.

<file-attachment-contents filename="Review A.txt">
</file-attachment-contents>

<file-attachment-contents filename="Review B.txt">
</file-attachment-contents>

Please output the evaluation results in the following JSON format:

```
{
  "Content Quality": {
    "Topic Relevance": {
      "Requirement": "Relevance and importance to the topic",
      "Scoring Rationale": "",
      "Review A Score": 0,
      "Review B Score": 0,
      "Superior Article": ""
    },
    "Comprehensiveness of Information": {
      "Requirement": "Breadth and depth of topic coverage",
      "Scoring Rationale": "",
      "Review A Score": 0,
      "Review B Score": 0,
      "Superior Article": ""
    },
    "Accuracy of Information": {
      "Requirement": "Correctness of data and statements",
      "Scoring Rationale": "",
      "Review A Score": 0,
      "Review B Score": 0,
      "Superior Article": ""
    },
    "Innovativeness of Viewpoints": {
      "Requirement": "Novel insights and unique perspectives",
      "Scoring Rationale": "",
      "Review A Score": 0,
      "Review B Score": 0,
      "Superior Article": ""
    },
```

```
    "Integration of Interdisciplinary Perspectives": {
      "Requirement": "Degree of multidisciplinary knowledge integration",
      "Scoring Rationale": "",
      "Review A Score": 0,
      "Review B Score": 0,
      "Superior Article": ""
    }
  },
  "Structure and Organization": {
    "Clarity of Logical Structure": {
      "Requirement": "Coherence of argumentation process",
      "Scoring Rationale": "",
      "Review A Score": 0,
      "Review B Score": 0,
      "Superior Article": ""
    },
    "Reasonableness of Chapter Arrangement": {
      "Requirement": "Balance and sequence of content distribution",
      "Scoring Rationale": "",
      "Review A Score": 0,
      "Review B Score": 0,
      "Superior Article": ""
    },
    "Quality of Introduction and Conclusion": {
      "Requirement": "Effectiveness of opening and closing",
      "Scoring Rationale": "",
      "Review A Score": 0,
      "Review B Score": 0,
      "Superior Article": ""
    }
  },
  "Academic Standards": {
    "Accuracy and Completeness of Citations": {
      "Requirement": "Correctness of citation format and content",
      "Scoring Rationale": "",
      "Review A Score": 0,
      "Review B Score": 0,
      "Superior Article": ""
    },
    "Timeliness and Relevance of References": {
      "Requirement": "Recency and relevance of literature",
      "Scoring Rationale": "",
      "Review A Score": 0,
      "Review B Score": 0,
      "Superior Article": ""
    },
    "Avoidance of Plagiarism and Appropriate Citation": {
      "Requirement": "Originality and appropriateness of citations",
      "Scoring Rationale": "",
      "Review A Score": 0,
      "Review B Score": 0,
      "Superior Article": ""
    }
  },
  "Innovation and Contribution": {
    "Critical Analysis of Existing Research": {
      "Requirement": "Depth of analysis and insight",
```

"Scoring Rationale": "",
"Review A Score": 0,
"Review B Score": 0,
"Superior Article": ""
},
"Proposal of New Research Directions or Questions": {
"Requirement": "Foresight and inspirational quality",
"Scoring Rationale": "",
"Review A Score": 0,
"Review B Score": 0,
"Superior Article": ""
},
"Potential Contribution to the Field": {
"Requirement": "Degree of theoretical or practical advancement",
"Scoring Rationale": "",
"Review A Score": 0,
"Review B Score": 0,
"Superior Article": ""
}
},
"Methodology Assessment": {
"Clarity of Research Method Description": {
"Requirement": "Clarity of methodological explanation",
"Scoring Rationale": "",
"Review A Score": 0,
"Review B Score": 0,
"Superior Article": ""
},
"Rationality of Method Selection": {
"Requirement": "Match between method and research question",
"Scoring Rationale": "",
"Review A Score": 0,
"Review B Score": 0,
"Superior Article": ""
},
"Rigor of Research Design": {
"Requirement": "Scientific nature and reliability of design",
"Scoring Rationale": "",
"Review A Score": 0,
"Review B Score": 0,
"Superior Article": ""
}
},
"Timeliness and Cutting-edge Nature": {
"Inclusion of Latest Research Results": {
"Requirement": "Degree of citation of latest literature",
"Scoring Rationale": "",
"Review A Score": 0,
"Review B Score": 0,
"Superior Article": ""
},
"Reflection of Current Research Hotspots": {
"Requirement": "Coverage of hot issues",
"Scoring Rationale": "",
"Review A Score": 0,
"Review B Score": 0,
"Superior Article": ""

```
    }
},
"Writing Quality": {
    "Fluency of Language Expression": {
        "Requirement": "Smooth writing, easy to understand",
        "Scoring Rationale": "",
        "Review A Score": 0,
        "Review B Score": 0,
        "Superior Article": ""
    },
    "Appropriateness of Professional Terminology Use": {
        "Requirement": "Accuracy and consistency of terminology use",
        "Scoring Rationale": "",
        "Review A Score": 0,
        "Review B Score": 0,
        "Superior Article": ""
    },
    "Conciseness of Expression": {
        "Requirement": "Degree of language refinement",
        "Scoring Rationale": "",
        "Review A Score": 0,
        "Review B Score": 0,
        "Superior Article": ""
    }
},
"Practicality and Application Value": {
    "Practical Application Potential of Research Results": {
        "Requirement": "Guiding significance for practice",
        "Scoring Rationale": "",
        "Review A Score": 0,
        "Review B Score": 0,
        "Superior Article": ""
    },
    "Feasibility of Policy Suggestions": {
        "Requirement": "Realism and operability of suggestions",
        "Scoring Rationale": "",
        "Review A Score": 0,
        "Review B Score": 0,
        "Superior Article": ""
    }
},
"Critical Thinking": {
    "Logic of Argumentation": {
        "Requirement": "Rationality of reasoning process",
        "Scoring Rationale": "",
        "Review A Score": 0,
        "Review B Score": 0,
        "Superior Article": ""
    },
    "Multi-angle Analysis of Problems": {
        "Requirement": "Diversity and comprehensiveness of perspectives",
        "Scoring Rationale": "",
        "Review A Score": 0,
        "Review B Score": 0,
        "Superior Article": ""
    },
    "Identification and Discussion of Research Limitations": {
```

"Requirement": "Recognition and analysis of limiting factors",
"Scoring Rationale": "",
"Review A Score": 0,
"Review B Score": 0,
"Superior Article": ""
}
},
"Overall Evaluation": {
"Review A Total Score": 0,
"Review B Total Score": 0,
"Superior Review": "",
"Summary": ""
}
}

In your output, please ensure:
1. The scoring rationale for each point is concise yet substantive, not exceeding 20 words.
2. Scores are precise to one decimal place and show differentiation.
3. Indicate the superior article for each scoring point (output "A" or "B", if scores are equal, mark as "AB").
4. The total score is the sum of all scoring point scores.
5. Provide a brief comparison and conclusion in the overall evaluation, explaining why one review is superior, not exceeding 50 words.

When evaluating, please note:
- Maintain objectivity and conciseness.
- Provide accurate scores based on the content of the articles.
- Only provide the most critical observations or judgments in the scoring rationales.
- When comparing the two articles, focus on their significant differences.

Now, please begin your evaluation work. Remember, your goal is to provide a concise yet comprehensive comparison, highlighting the key differences between the two reviews.
