# Supplementary material for: Automated literature research and review-generation method based on large language models
Source: Natl Sci Rev. 2025 Apr 25;12(6):nwaf169. doi: 10.1093/nsr/nwaf169 (PMC12125968; doi:10.1093/nsr/nwaf169)
Supplement: nwaf169_Supplemental_Files [file nwaf169_supplemental_files.zip › Supplementary data.pdf]

Automated literature research and review generation method based on large language models

Shican Wu[1,#], Xiao Ma[1,#], Dehui Luo[1], Lulu Li[1], Xiangcheng Shi[2], Xin Chang[2], Xiaoyun Lin[1], Ran Luo[2], Chunlei Pei[1,3], Changyin Du[4], Zhi-Jian Zhao[1,5,*] and Jinlong Gong[1,2,3,5,6,7,8,*]

[1]School of Chemical Engineering and Technology; Key Laboratory for Green Chemical Technology of Ministry of Education, Tianjin University; Collaborative Innovation Center of Chemical Science and Engineering (Tianjin); Tianjin 300072, China;

[2]Joint School of National University of Singapore and Tianjin University, International Campus of Tianjin University, Binhai New City, Fuzhou 350207, China;

[3]Zhejiang Institute of Tianjin University, Ningbo 315201, China;

[4]AIStrucX Technologies, Beijing 100000, China;

[5]International Joint Laboratory of Low-carbon Chemical Engineering, Tianjin 300192, China;

[6]Haihe Laboratory of Sustainable Chemical Transformations, Tianjin 300192, China;

[7]National Industry-Education Platform of Energy Storage, Tianjin University, Tianjin 300350, China;

[8]Tianjin Normal University, Tianjin 300387, China

***Corresponding authors**. E-mails: zjzhao@tju.edu.cn; jlgong@tju.edu.cn

[#]Eqaully contributed to this work.

Supplementary Information

1: Example of Generated Content: Appendix I 3
2: Comprehensive Charts from Data Mining Module: Appendix II 3
3: Software Interface: Appendix III 3
4: Manual Verification of Hallucinations: Appendix IV 3
5: Scoring Criteria for Generated Paragraphs 4
6: Factors Influencing the Selection of LLMs 6
7: Comparison of Automated and Manual Web of Science (WoS) Retrieval 7
8: Standard Metrics for Evaluating the Performance of Classification Models 10
9: Calculation of Theoretical Accuracy Improvement via Self-Consistency Aggregation 10
10: Details for Intraclass Correlation Coefficient (ICC) tests and Transitive Consistency Ratio (TCR) calculations 12
11: Review Quality Assessment Scoring Criteria for LLM-Generated Scientific Reviews: Appendix V 13
12: Reliability Assessment of Qwen2-7b-Instruct: Appendix VI 13
13: Comprehensive Model Evaluation Results: Appendix VII 13
14: Prompt Templates: Appendix VIII 14
15: Human evolution of Review Generated by Deep Seek and Claude 14
16: Multi-Evaluator Assessment Reliability 17

All supplementary files have been uploaded to our GitHub pages:

Appendix1.pdf:
https://github.com/TJU-ECAT-AI/AutomaticReviewGenerationData/blob/main/Appendix1.pdf

Appendix2.xlsx:
https://github.com/TJU-ECAT-AI/AutomaticReviewGenerationData/blob/main/Appendix2.xlsx

SI.pdf:
https://github.com/TJU-ECAT-AI/AutomaticReviewGenerationData/blob/main/SI.pdf

1: Example of Generated Content: Appendix I

To review a comprehensive sample of automatically generated content on propane dehydrogenation catalyst advancements produced by the large language model, refer to the 35-chapter review paper included in Appendix I. This will showcase the system's text synthesis capabilities on this topic. The content of the review is unaltered in format for authenticity.

Additionally, we have also provided the intermediate results of five other reviews, which include the paragraphs before integration, as well as direct DOI link references that can be used for precise tracing of knowledge sources. Similarly, the content of the review is unaltered in format for authenticity.

2: Comprehensive Charts from Data Mining Module: Appendix II

Appendix II contains extensive graphical overviews analyzing publication trends and performance correlations related to propane dehydrogenation catalysts, generated through an integrated data mining module. Consult the multi-factor charts here for a data-driven perspective.

Fig.A1 Annual publication number charts: Granular perspectives on publication volumes over time across salient catalyst factors are presented through Gantt charts in Fig.A1.

Fig.A2 Peak performance radar charts: For radar visualizations contrasting maximum recorded performance between key catalyst traits like structure type, preparation method and active species.

Fig.A3 Bivariate correlation charts: Fig.A3 statistically examines interrelationships and correlations between catalyst formulation aspects and resultant performance through bivariate bubble charts.

3: Software Interface: Appendix III

Screenshots demonstrating and outlining capabilities of the software system's visual interface are exhibited in Appendix III (Fig.A4-9). Refer here for an overview of key features and options.

4: Manual Verification of Hallucinations: Appendix IV

Detailed results from manual verification of potential factual inaccuracies and hallucinations in the generated text are compiled in tabular format within Appendix IV. Consult this appendix for robustness assessments.

5: Scoring Criteria for Generated Paragraphs

To ensure the quality and scientific value of the automatically generated review content, this study has designed a multi-dimensional automatic evaluation system for the generated paragraphs. This system encompasses the following key aspects:

Table S1 Criteria for automatic paragraph scoring

Assessment Dimension	Description
Clarity	Ensures textual clarity and ease of comprehension.
Depth	Focuses on whether the text provides in-depth discussion and insights on the topic.
Relevance and Comprehensiveness	Ensures content is closely related and comprehensively covers the topic.
Coherence	Guarantees logical flow and overall consistency in the text.
Originality	Emphasizes whether the text presents new viewpoints or insights.
Evidence-Based	Ensures viewpoints are well-supported by academic references.
Structure	Concentrates on the text's organizational logic and integration of various parts.
Text Length	Reflects the thoroughness of the text.
Unique DOI Count	Indicates the diversity of citations.
Consistency	Ensures paragraphs referencing the same DOI are consistent in theme and content.

The consistency metric also embodies the concept of self-consistency: a description found consistent across all paragraphs referencing the same DOI is more likely to be accurate. This includes not only literal or lexical consistency but also a deeper exploration of thematic and contextual consistency across different paragraphs.

Using this approach, each paragraph is rigorously and impartially evaluated across all dimensions, ensuring the selection of the most effective paragraphs for the current topic while maintaining cohesion, depth, and thematic consistency in discussions across different paragraphs.

In terms of paragraph scoring, analysis of the distribution histogram revealed that scores generally follow a normal distribution. This implies that even a randomly selected result would

maintain a relatively stable level of performance. The design of this evaluation system, while ensuring accuracy and transparency of assessments, achieves rationalization and replicability of results, providing an efficient and scientific quality assurance mechanism for the generation of fully automated reviews.

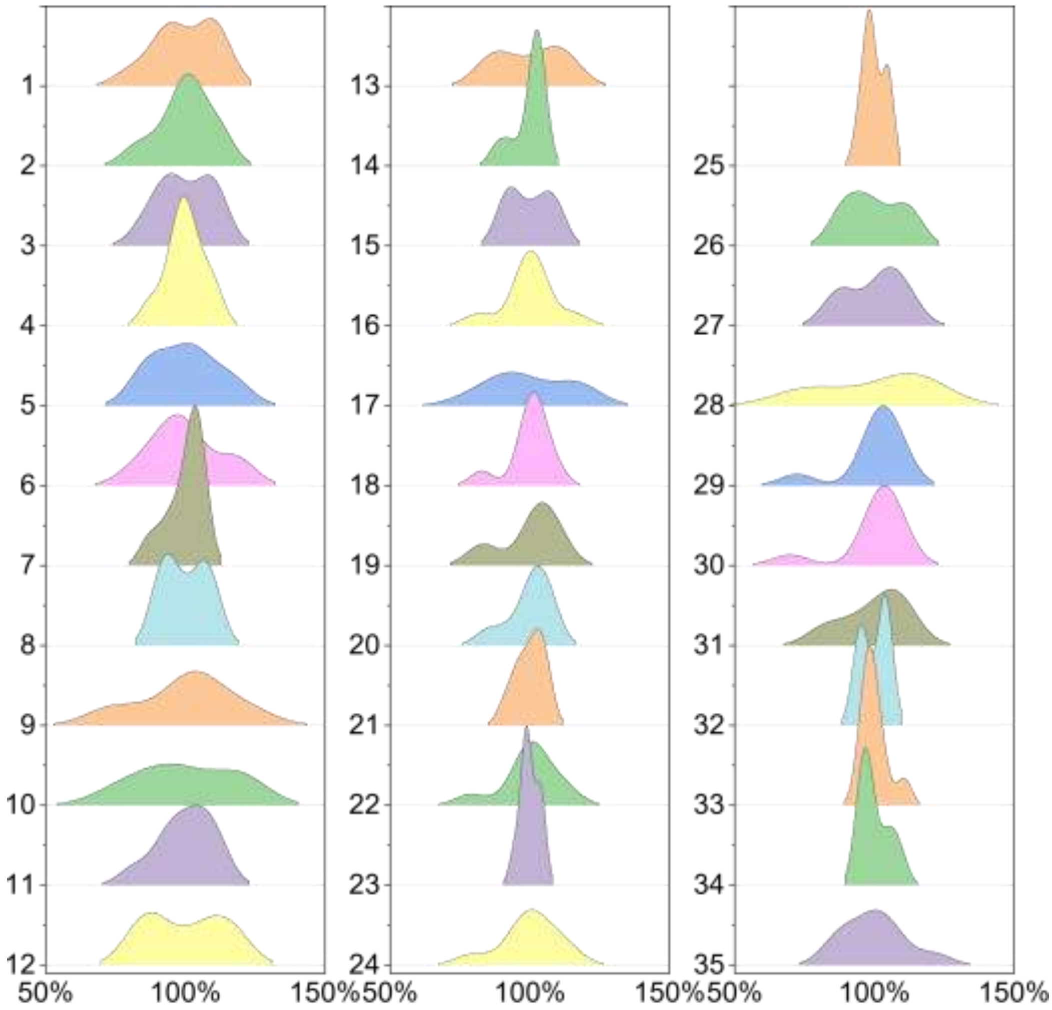

Fig.S1 Distribution of paragraph scores. The horizontal axis represents the ratio of paragraph scores relative to the average score of all paragraphs in the section, and the vertical axis lists the numbers of the 35 guiding questions used in the case study.

6: Factors Influencing the Selection of LLMs

While the essence of our method lies in its unique procedural design and implementation, the final outcome and breadth of application are significantly influenced by the inherent performance of the chosen LLM. Theoretically, our method is highly adaptable, capable of utilizing various LLMs. However, in practice, differences in how LLMs handle natural language understanding and generation tasks directly impact the quality and efficiency of review generation. We considered several key factors, including supported context length, model size and parameter count, as well as multimodal and chart handling capabilities, leading to the selection of the Claude2 model as the LLM for this study.

Before delving into context length considerations, it's crucial to understand the length distribution of the target literature. We analyzed the text length of 343 documents entering the information extraction phase using Claude's API, resulting in a histogram of literature length distribution. It was observed that 69.1% of the documents exceeded 8k tokens in length, and 6.1% exceeded 32k tokens. Moreover, during the process of full-text polishing, it becomes necessary to input the entire text into the model, and LLMs limited to processing only parts of the text may struggle to grasp the article's overall context. These exceedingly long texts pose a challenge in handling context, as texts exceeding a model's processing capacity are typically truncated or ignored, potentially leading to the loss of crucial information. Existing methods, such as reducing model parameters, downsampling context, focusing solely on recent input, or segmenting the entire content, alleviate computational and storage pressures but often at the cost of sacrificing the model's understanding and generative capabilities. For example, methods like downsampling, focusing only on recent input, or segmenting all content may prevent the model from fully comprehending the global context of the text, leading to potential information loss or even more severe hallucinations. Given the tendency of large models to lose middle-section information when processing texts near their contextual limits (as per arXiv:2307.03172), choosing an LLM with the longest possible context window is advisable for optimal performance.

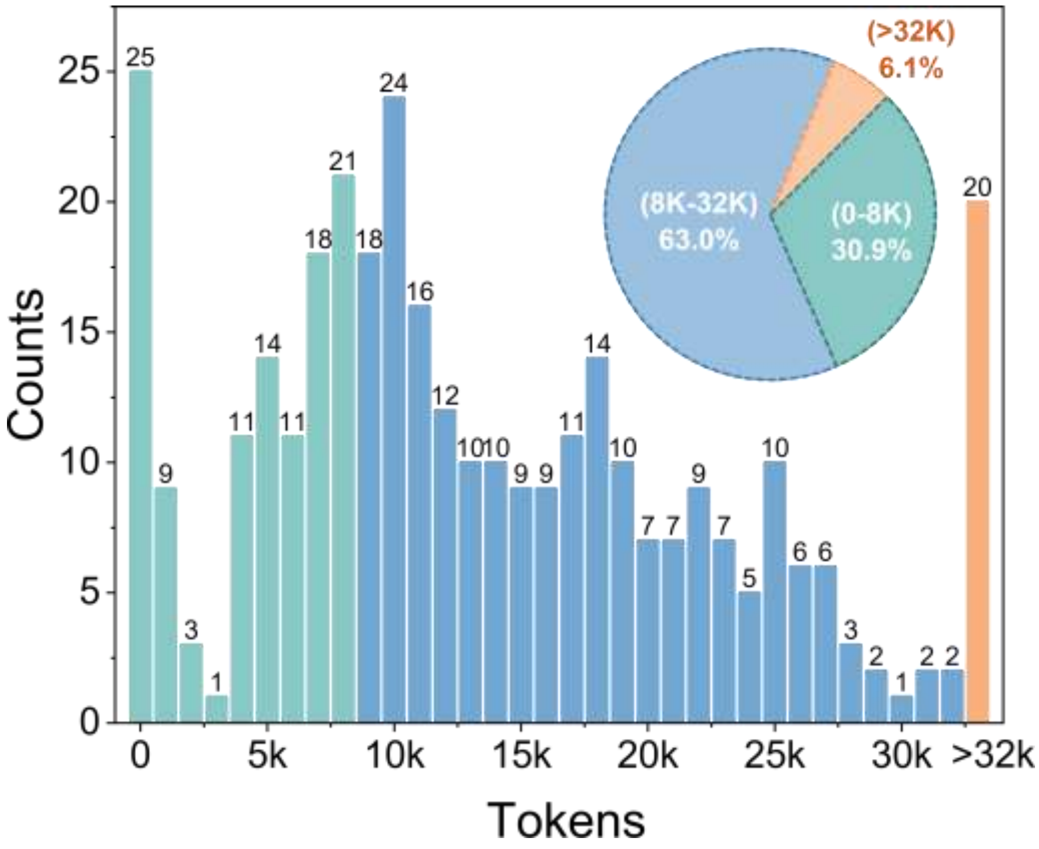

Fig.S2 Token count of literature entering the information extraction phase.

For a detailed breakdown of factors influencing LLM selection, please refer to Table A1 in the accompanying excel file (Appendix2.xlsx, Sheet: Table A1). In summary, the Claude2 model was chosen as the preferred LLM for our automated review generation method due to its superior context processing ability, extensive parameter count, and robust capability in handling table information. This makes Claude2 an ideal choice for automating the generation of scientific literature reviews, ensuring the accuracy and coherence of processed information.

7: Comparison of Automated and Manual Web of Science (WoS) Retrieval

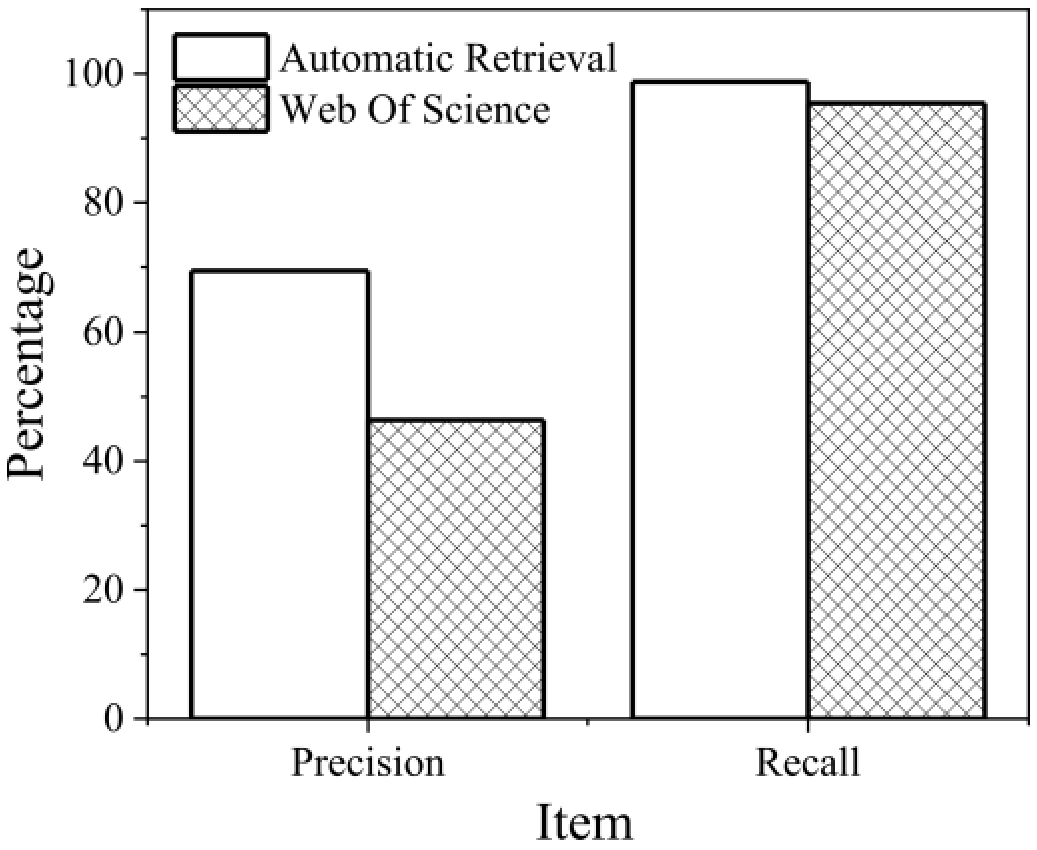

Fig.S3 Bar chart illustrating precision and recall rates for manual WoS retrieval compared to automated retrieval.

Table S2 Comparison of Automated vs. Manual Retrieval Outcomes

Metric	Automated Retrieval	Manual Retrieval (WoS)
Total Number of Retrieved Results	1420 (before quick filter)<br>343 (after quick filter)	496
Total Number of Documents Deemed Relevant by LLM	238	
Precision Rate	69.39%	46.37%
Recall Rate	98.76%	95.44%
Number of Documents Not Appearing in Retrieval Results	11 (3 related to PDH)	11 (all related to PDH)

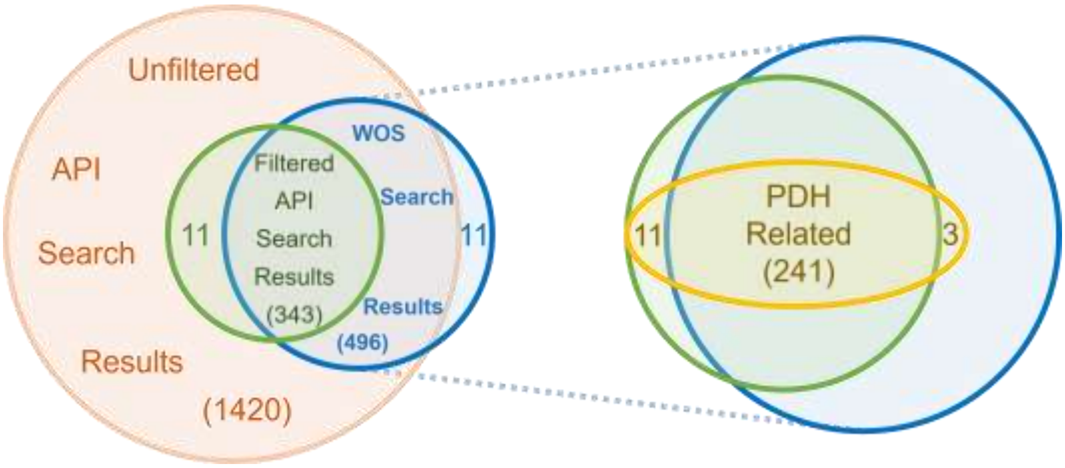

Fig.S4 Venn diagram illustrating the intersection and union of documents retrieved through manual WoS retrieval and automated retrieval systems.

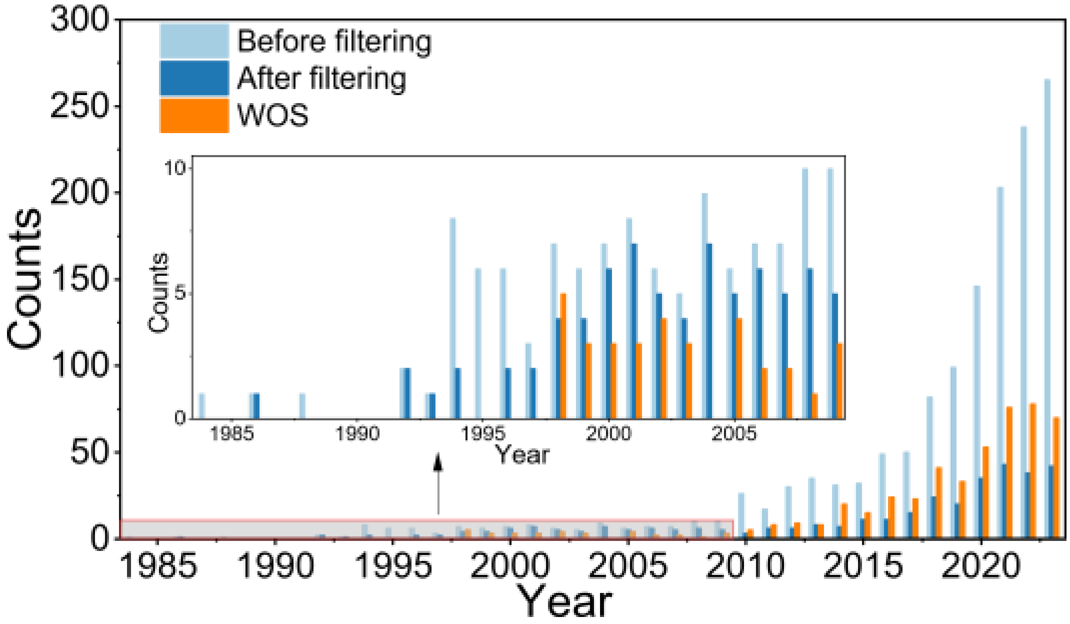

Fig.S5 Comparison of the number of publications retrieved via manual WoS retrieval and automated retrieval.

8: Standard Metrics for Evaluating the Performance of Classification Models

The formulas provided are standard metrics for evaluating the performance of classification models and algorithms in machine learning and data analysis, refer to Table A2-6 (Appendix2.xlsx, Sheet: Table A2-6) in Appendix IV. Here's what each term and formula represent:

TP (True Positives): Correctly predicted positive observations

TN (True Negatives): Correctly predicted negative observations

FP (False Positives): Incorrectly predicted positive observations

FN (False Negatives): Incorrectly predicted negative observations

Accuracy: Measures the proportion of true results (both true positives and true negatives) among the total number of cases examined.

Accuracy=(TP+TN)/(TP+TN+FP+FN)

False Positive Rate: The proportion of negative cases that were incorrectly classified as positive.

False Positive Rate (FPR)=FP/(TN+FP)

Precision (P): The ratio of correctly predicted positive observations to the total predicted positives.

Precision (P)=TP/(TP+FP)

Recall (R): The ratio of correctly predicted positive observations to all observations in the actual class.

Recall (R)=TP/(TP+FN)

F1 Score: The weighted average of Precision and Recall, used when you seek a balance between Precision and Recall.

F1 Score=2 ×P ×R/(P+R)

Example python code snippet to calculate the 95% confidence interval for the False Positive Rate:

```
from statsmodels.stats.proportion import proportion_confint
confidence_level = 0.95
FPR_confint = proportion_confint(count=FP, nobs=TN + FP, alpha=(1 - confidence_level), method='wilson')
```

9: Calculation of Theoretical Accuracy Improvement via Self-Consistency Aggregation

We aimed to calculate the probability of achieving accurate final predictions using Model A (with an initial accuracy rate of 79.09%) by independently predicting five times and aggregating these predictions. Assuming that incorrect answers are random and the correct

answer is unique, we determined the final accuracy rate by calculating the probability of at least three correct predictions out of five trials.

To simplify this calculation, we employed the binomial probability formula. In this scenario:

The probability of success (i.e., Model A providing the correct answer) is p = 0.7909.

The probability of failure (i.e., Model A providing an incorrect answer) is q = 1 - p = 0.2091.

A total of n = 5 independent trials are conducted, where n is any odd number greater than 3.

We are interested in the probability of having at least k = 3 successes, where k = (n + 1) / 2. This can be calculated using the following binomial probability formula:

$$P\left(x \geq \frac{n+1}{2}\right) = \sum_{i=\frac{n+1}{2}}^{n} \frac{n!}{i!\,(n-i)!} p^{i}(1-p)^{n-i}$$

Substituting into the formula, the probability of a correct final prediction when aggregating five independent predictions using Model A is approximately 93.49%.

Similarly calculated, the probability of a correct final prediction when aggregating seven independent predictions using Model A is approximately 96.12%.

Similarly calculated, the probability of a correct final prediction when aggregating nine independent predictions using Model A is approximately 97.64%.

10: Details for Intraclass Correlation Coefficient (ICC) tests and Transitive Consistency Ratio (TCR) calculations

For ICC:

$$ICC = \frac{MS_B - MS_W}{(MS_B + (k-1) * MS_W)}$$

Where:

$$MS_B = \frac{k \sum (\bar{x_i} - \bar{x})^2}{n-1}$$

$$MS_E = \frac{\sum \sum (x_{ij} - \bar{x_i})^2}{n(k-1)}$$

MSB: Mean square between paragraph pairs

MSE: Mean square error within comparisons

k: Number of comparisons made

n: number of paragraph pairs

$\bar{x_i}$: mean difference score for pair i

$\bar{x}$: grand mean of all difference scores

x_{ij}: difference score of pair i in comparison j

The detailed calculation step is as follows:

1. Standardize pairs by sorting paragraph IDs and calculating score differences
2. Apply sign correction based on pair ordering
3. Group differences by paragraph pairs across comparisons
4. Calculate MSB from mean differences between pairs
5. Calculate MSE from variance within pair comparisons
6. Apply formula with k being number of comparisons

For TCR:

$$T = \frac{T - I}{T} * 100\%$$

Where:

- Comparison Matrix M: M[x,y] = sign(mean(Score_x - Score_y))
  - Score_x: score of paragraph x
  - Score_y: score of paragraph y
  - M[x,y] takes values in {-1, 0, 1}:
    - 1: x is preferred over y
    - -1: y is preferred over x
    - 0: tie between x and y
- T (Total Valid Triplets):
  - Total number of combinations selecting 3 paragraphs from all paragraphs
  - Excludes combinations containing ties (M[x,y]=0)
  - Formula: T = C(n,3) - t_ties
    - n: total number of paragraphs
    - C(n,3): number of combinations selecting 3 from n paragraphs
    - t_ties: number of triplets containing ties
- I (Intransitive Triplets): For any triplet (a,b,c), it is counted as intransitive if either:

Condition 1: M[a,b] > 0 AND M[b,c] > 0 AND M[a,c] ≤ 0
Condition 2: M[a,b] < 0 AND M[b,c] < 0 AND M[a,c] ≥ 0

Calculation Steps:

1. Create comparison matrix with mean score differences
2. Convert to sign matrix (-1,0,1) for each pair
3. Find all possible triplet combinations
4. Count triplets violating transitivity (A>B>C but A≤C)
5. Exclude triplets containing ties
6. Calculate ratio and convert to percentage

And all these following calculations are achieved in our python files present on GitHub Pages: https://github.com/TJU-ECAT-AI/AutomaticReviewGeneration/blob/main/QualityEvaluation/ComparedScore.py

11: Review Quality Assessment Scoring Criteria for LLM-Generated Scientific Reviews: Appendix V

For a comprehensive framework evaluating the quality of LLM-generated scientific reviews, refer to Table A7 (Appendix2.xlsx, Sheet: Table A7) in Appendix V. This scoring system provides structured criteria for assessing various aspects of automatically generated scientific reviews, ensuring consistent and objective evaluation across different review outputs.

12: Reliability Assessment of Qwen2-7b-Instruct: Appendix VI

Appendix VI presents detailed reliability assessment results for the Qwen2-7b-Instruct model through various analytical visualizations including ICC (Intraclass Correlation Coefficient) and TCR (Transitive Consistency Ratio) heatmaps. Fig.A10 provides comprehensive visualization of the model's reliability metrics, including comparative analyses between self-evaluation and Qwen2-72b-Instruct evaluation results.

13: Comprehensive Model Evaluation Results: Appendix VII

For an extensive analysis of model performance comparisons, consult Appendix VII. This section contains normalized assessment scores for Claude3.5Sonnet, Qwen2-72b-Instruct, and Qwen2-7b-Instruct (Appendix2.xlsx, Sheets: Tables A8-10), along with detailed performance visualizations in Fig.A11. These analyses include scaling law comparisons, three-dimensional performance metrics, distribution analyses, and various performance heatmaps across different evaluation dimensions. For cross-model evaluation, Tables A11 and 12 (Appendix2.xlsx, Sheets: Table A11-12) contain the normalized assessment scores of Claude3.5Sonnet and Qwen2-7b-Instruct as evaluated by Qwen2-72b-Instruct.

14: Prompt Templates: Appendix VIII

The complete collection of prompt templates utilized in this study is documented in Appendix VIII. These templates serve as standardized frameworks for generating consistent and high-quality review content across different topics and sections.

15: Human evolution of Review Generated by Deep Seek and Claude

To demonstrate the impact of LLM development on the reviews generated by our method, we used the same 343 PDH-related publications as in the main text to generate a review article. For this demonstration, we specifically chose DeepSeek models as they represent affordable inference model options that remain accessible for research purposes. Initially, we used DeepSeek-R1; however, due to its maximum token limit (16K), we had to delete paragraphs 2, 4, 9, 10, 16, and 24, as these paragraphs were too long and reached the token limit. To make our testing more rigorous and comprehensive, we also conducted experiments with DeepSeek-V3, which belongs to the same model family but offers a longer context length (128K). Based on the knowledge extraction results from DeepSeek-R1, we further used DeepSeek-V3 to generate another edition. You can see both editions in the following link:

Generated by DeepSeek-R1:

Catalytic Advances in Propane Dehydrogenation: Mechanistic Insights and Material Design Strategies (DeepSeek R1)

GitHub Link:

https://github.com/TJU-ECAT-AI/AutomaticReviewGenerationData/blob/main/ExampleOfReview/Catalytic%20Advances%20in%20Propane%20Dehydrogenation%20Mechanistic%20Insights%20and%20Material%20Design%20Strategies-DeepSeek%20R1.txt

Generated by DeepSeek-R1 and DeepSeek-V3:

Recent Advances in Propane Dehydrogenation Catalysis: From Molecular Understanding to Industrial Application (Deep Seek R1+V3)

GitHub Link:

https://github.com/TJU-ECAT-AI/AutomaticReviewGenerationData/blob/main/ExampleOfReview/Recent%20Advances%20in%20Propane%20Dehydrogenation%20Catalysis%20From%20Molecular%20Understanding%20to%20Industrial%20Application-DeepSeek%20R1%2BV3.txt

Additionally, we selected 14 experts and PhD students from relevant fields to conduct anonymous peer reviews. Without prior knowledge of the model used, they read two review articles and provided evaluations and comments based on their personal preferences, as listed below. Among these, the review written by the DeepSeek-R1 model outperformed the review written by Claude with an advantage of 11:3. (Claude: Review 1, Deep Seek: Review 2).

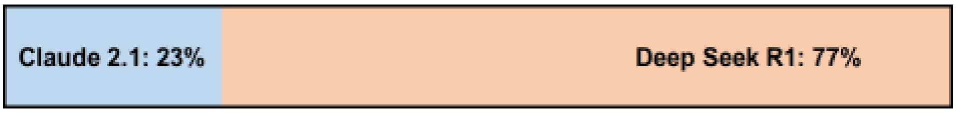

Fig.S6 Preference Distribution Between Deep Seek R3 and Claude in Expert Review Evaluations

Table S3 Comparative Comments from Expert Reviewers on Two Generated Review Articles

Index	Comments	Review 1	Review 2
1	Rev2 offers clearer comprehension and stronger summarization compared to Rev1. The article in Rev2 more closely resembles a review-type format and presents the content with greater effectiveness.		√
2	I recommend Rev1 more. Rev1 better conforms to the structure of a review article. Rev2 provides more dynamic descriptions of cited literature, and Rev2 presents clearer logic. The summary section of Rev2 contains more substantive content compared to Rev1's impressive-sounding statements.	√	
3	I recommend Rev2 more. Rev2 has better readability and is more fluid to read. Rev2 is more innovative, analyzing articles while demonstrating understanding. If I had to choose, I would select the second one, and then add my own understanding to it later.		√
4	Rev2 is more comprehensive and systematic, with advantages in: covering a broader domain, including catalyst design, theoretical modeling, industrial applications, and process optimization; having a more rigorous structure with logical progression between chapters, forming a complete "fundamentals-applications-outlook" chain; and providing richer technical details with both depth and breadth in the analysis of key issues, making it suitable as an authoritative reference in the PDH field. Specifically: Rev2 appears to have many chapters		√

Index	Comments	Review 1	Review 2
	covering different categories of catalysts, theoretical calculations, reactor design, future outlook, etc. Each section has detailed subheadings, such as metal oxide catalysts, bimetallic catalysts, zeolite supports, etc., and cites numerous references. Rev1 is also a review with a concise structure, mainly divided into introduction, theoretical principles, catalyst synthesis and characterization, reactor and process considerations, conclusions and future outlook. In comparison, Rev2 has a more detailed structure with clearer points, such as independent chapters for each catalyst category, allowing for more in-depth discussion.		
5	the second one might be relatively deeper and seems more comprehensive		√
6	I feel the second one is better. The second one offers more clarity and focuses on essential points compared to the first one. Personal opinion		√
7	I like the second one, it's probably a matter of style.		√
8	After just studying and taking a look, both are quite good, but I personally lean more toward the second one.		√
9	The first one is clearly better than the second one. On one hand, it has its own approach to catalyst design, and the subsequent discussion on reactor design, economic viability, and safety aspects is not commonly seen in reviews on dehydrogenation. The overall structure is more novel than the second one.	√	
10	I feel that the introduction sequence in rev1 is better. It first introduces the theory and reaction mechanisms, then discusses catalyst types, characterization, and reactors, before reaching conclusions. Additionally, I think it would be better to move the theory-related	√	

Index	Comments	Review 1	Review 2
	content forward. The other one focuses more on introducing various catalyst categories and industrial applications.		
11	To me, both are at similar levels in terms of writing, but Version 2 is better structured. Version 2 stands out with its clear expression backed by a numbers approach, while Version 1 takes more of an assertive "I said so" approach.		√
12	I feel rev2 is a bit better. The overall logic is somewhat better. Rev1 seems more like summarizing one paper after another.		√
13	I feel that 1 is concise, while 2 offers more depth and comprehensiveness. Overall, I think 2 would be better.		√
14	I didn't read it very carefully, just browsed through it overall. My general impression is that the 1st article is better in terms of structural organization, but regarding the text, the second article is relatively more concise and easier to understand, with more comprehensive content. Each has its advantages. If I were to rank them overall, I still think the second one is a bit better.		√
Total		3	11

16: Multi-Evaluator Assessment Reliability

Our method's reliability was critically assessed through a carefully designed cross-validation process. Table S4 presents the comparative performance metrics across different evaluation scenarios: Reviewer 0 analyzed a set of 25 papers (875 data points), while Reviewers 1-3 independently assessed an identical set of 20 papers (700 data points each), resulting in a comprehensive evaluation totaling 2975 data points.

Table S4 Comparison of Results Between Different Reviewers

Stage	Data Points	Accu-racy	False Positive Rate	95% CI of FPR	Preci-sion	Recall	F1 Score

Reviewer 0	875	95.77%	0.000%	0.000% - 0.485%	100.0%	57.47%	72.99%
Reviewer 1	700	96.71%	0.32%	0.090% - 1.160%	96.43%	72.00%	82.44%
Reviewer 2	700	95.71%	0.000%	0.000% - 0.610%	100.0%	60.53%	75.41%
Reviewer 3	700	95.86%	0.16%	0.030% - 0.900%	97.96%	63.16%	76.80%
**Total**	**2975**	**96.19%**	**0.090%**	**0.030% - 0.270%**	**98.82%**	**64.78%**	**78.26%**

The data demonstrates remarkable consistency across both different paper sets and different evaluators. Accuracy values range narrowly from 95.71% to 96.71%, suggesting that our assessment framework produces reliable results regardless of the specific evaluator or the particular papers being analyzed. This consistency is particularly significant given that Reviewer 0 assessed a completely different set of papers than Reviewer 1-3, yet produced comparable results.

Especially noteworthy is the consistently low false positive rate observed across all evaluations (0.000%-0.32%), with a combined rate of 0.090% (95% CI: 0.030%-0.270%). The fact that this critical metric remains extremely low across different paper sets and different evaluators provides strong evidence that our approach effectively addresses the risk of hallucination in LLM-generated content.

The cross-validation approach combining evaluation of different paper sets with multiple independent assessments of the same papers provides comprehensive evidence that our methodology produces reliable, consistent results with minimal risk of hallucination (below 0.5% with 95% confidence) across varying conditions, strengthening confidence in the robustness of our method.
